# Divergent reaction pathways in gold-catalyzed cycloisomerization of 1,5-enynes containing a cyclopropane ring: dramatic *ortho* substituent and temperature effects†

**DOI:** 10.1039/c6sc00058d

**Published:** 2016-03-04

**Authors:** Gen-Qiang Chen, Wei Fang, Yin Wei, Xiang-Ying Tang, Min Shi

**Affiliations:** a State Key Laboratory of Organometallic Chemistry, Shanghai Institute of Organic Chemistry, Chinese Academy of Sciences 345 Lingling Road Shanghai 200032 P. R. China weiyin@sioc.ac.cn siocxiangying@mail.sioc.ac.cn mshi@mail.sioc.ac.cn

## Abstract

A gold(i)-catalyzed cycloisomerization of easily available 1,5-enynes containing a cyclopropane ring has been developed, efficiently providing cyclobutane-fused 1,4-cyclohexadiene, tricyclic cyclobutene, biscyclopropane and 1,3-cyclohexadiene derivatives in moderate to excellent yields. When the phenyl group was not *ortho* substituted, 1,4-cyclohexadienes could be produced. With an *ortho* substituent, three different products could be selectively synthesized by control of the temperature and the used gold(i) catalyst. The 1,5-enyne substrate first undergoes a classical enyne cycloisomerization to form a tricyclic cyclobutene key intermediate, which undergoes subsequent transformation to produce the desired products. A plausible reaction mechanism was proposed according to deuterium labeling experiments and intermediate trapping experiments, as well as DFT calculations. In our current reaction, the *ortho* substituent on the phenyl group controls the reaction outcome and the *ortho* substituent effect was found to originate from steric and electronic factors.

## Abstract 2

Gold-catalyzed cycloisomerization of 1,5-enynes containing a cyclopropane ring provides access to cyclobutane-fused 1,4-cyclohexadiene, 1,3-cyclohexadiene, tricyclic cyclobutene and biscyclopropane derivatives.

## Graphical abstract

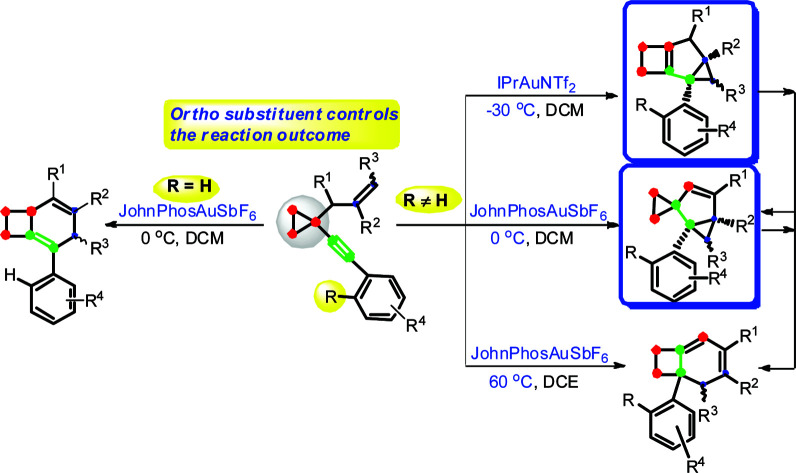

Transition metal-catalyzed enyne cycloisomerization^1^ is one of the most important strategies for the construction of cyclic structures from simple acyclic enyne substrates, of which 1,4-,^2^ 1,5-,^3^ 1,6-^4^ and 1,7-enynes^4*k*,5^ have been extensively examined. Among a range of transition metal catalysts for enyne cycloisomerization, gold(i) complexes are the most active and selective catalysts, probably due to relativistic effects.^6*a*,*b*^ Reports on homogeneous gold catalysis have been increasing explosively during the last decade^6,7^ and 1,5-enynes have always been the proving ground for gold catalysis. In 2004, Malacria^3*o*^ and Fürstner^3*p*^ reported their pioneering work on 1,5-enyne cycloisomerization, affording bicyclo[3.1.0]hexenes from 1,5-enynes with hydroxy or acyloxy groups at the propargylic position in the presence of PtCl_2_ or gold(i), respectively. Subsequently, Toste's group found that gold(i)-catalyzed isomerization of 1,5-enynes could efficiently produce bicyclo[3.1.0]hexane^3*q*^ or tetracyclic^3*h*^ compounds. Kozmin’s^3*j*,3*n*^ and Zhang's^3*k*^ groups disclosed that cyclohexadiene derivatives could be synthesized from siloxy-1,5-enynes or 3-carboxy-1,5-enynes under gold(i) catalysis.

On the other hand, cyclopropanes are versatile building blocks in organic synthesis.^8^ Their unique structure and intrinsic strain endow cyclopropanes with high reactivity. Thus far, it has been well known that a cyclopropyl group can effectively stabilize carbocations adjacent to it due to its π-character,^9^ and the cyclopropylmethyl carbocation is a stable non-classical carbocation that has several resonance structures: homoallyl carbocation, cyclobutyl carbocation, *etc.*^9*a*,10,11^ In 2010, Liu's group disclosed a novel PtCl_2_-catalyzed cycloisomerization of cyclopropyl group-tethered 1,4-enynes, efficiently providing eight-membered carbocycles (Scheme 1, eqn (1a)).^2*h*^ Subsequently, we found that cyclopropyl-tethered 1,4-enynes could undergo a tandem Pauson–Khand type reaction in the presence of a Rh(i) catalyst under CO atmosphere (Scheme 1, eqn (1b)).^2*c*^ However, the reaction of 1,5-enynes tethered by a cyclopropane has been rarely reported, to the best of our knowledge (Scheme 1, eqn (2)).^3*c*^

**Scheme 1:**
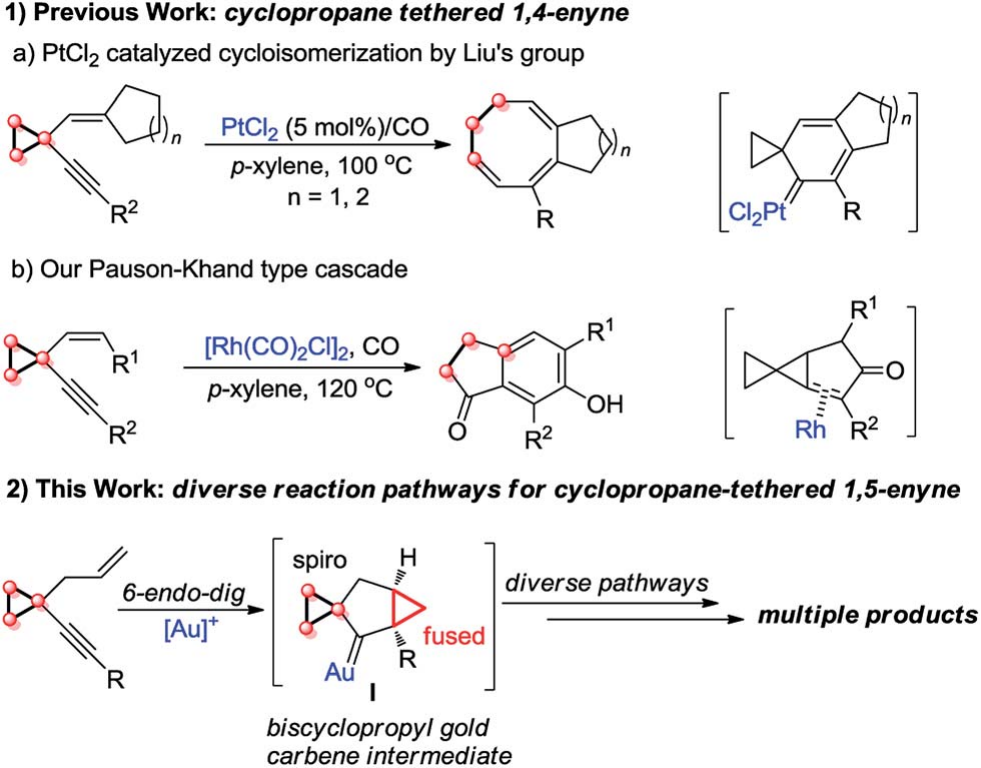
Previous work and this work.

On the basis of the research work of Liu's group, as well as gold-catalyzed cyclopropane chemistry from Schmalz^12*g*^ and other groups^12^ and our long-term exploration of cyclopropane chemistry,^13^ we envisaged that when the 1,5-enyne was tethered with a cyclopropane moiety, the reaction should be different. We postulate that a biscyclopropane gold carbene intermediate I can be produced in the presence of a gold catalyst. The intermediate I has a spiro and a fused cyclopropane moiety adjacent to the carbene center, and this unique structure makes it undergo diverse reaction pathways, affording multiple kinds of products (Scheme 1, eqn (2)).

The cyclopropane-tethered 1,5-enyne substrate 1a was synthesized and its reactivity was examined. We screened various gold catalysts to find the optimal catalyst. In the presence of [PPh_3_AuCl]/AgSbF_6_, product 3a could be afforded in 82% NMR yield. After further optimization of the reaction conditions, we found that when [JohnPhosAu·MeCN]SbF_6_ was used as the catalyst, product 3a could be produced in 92% isolated yield after 20 h at 0 °C in DCM (dichloromethane). Other gold catalysts, such as [(*p*-F-Ph)_3_PAu·MeCN]SbF_6_, [(*p*-CF_3_-Ph)_3_PAu·MeCN]SbF_6_, [P(OAr)_3_Au·MeCN]SbF_6_, [(*t*-Bu)_3_PAu·MeCN]SbF_6_, [JackiePhosAu·MeCN]SbF_6_, [XPhosAu·MeCN]SbF_6_, and [IPrAu·MeCN]SbF_6_ were also evaluated, but no better result was obtained. Therefore, [JohnPhosAu·MeCN]SbF_6_ was identified as the best catalyst for the current reaction (see Table SI-1 in the ESI† for the detailed optimization of the reaction conditions).

With the optimal reaction conditions in hand, we next turned our efforts to examine the substrate scope of the reaction. We found that when R^1^ was an aromatic group with an electron-donating or electron-withdrawing substituent (Table 1, entries 1–9), the corresponding products could be obtained in good to excellent yields. Only when a strongly electron-withdrawing group such as CF_3_ or NO_2_ was introduced (R^1^ = *p*-CF_3_-Ph or *p*-NO_2_-Ph), the reaction proceeded sluggishly and elevation of temperature was required for the complete conversion (Table 1, entries 10 and 11). When the substituents were heteroaromatic groups, such as thienyl or 5-indolyl groups, the reaction went smoothly to produce 3l and 3m in excellent yields (Table 1, entries 12 and 13). The reaction also worked very well when the substituent was a 2-naphthyl group or 6-methoxyl-2-naphthyl group (Table 1, entries 14 and 15). In addition, R^2^ could also be an alkyl group, and the corresponding product 3p could be obtained in 69% yield in the presence of IPrAuNTf_2_ (ref. 14) (Table 1, entry 16). The structure of 3 was unambiguously determined by the X-ray diffraction of compound 3o.^15^

**Table 1:** Au(i)-catalyzed cycloisomerization of 1 leading to 3

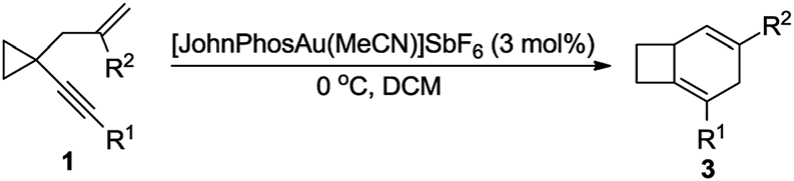			
Entry^a^	R^1^, R^2^	Time (h)	Yield^b^ (%)
1	1a, R^1^ = Ph, R^2^ = H	20	3a, 92
2	1b, R^1^ = *p*-Me-Ph, R^2^ = H	17	3b, 90
3	1c, R^1^ = 3,5-di-Me-Ph, R^2^ = H	14	3c, 91
4	1d, R^1^ = *p*-MeO-Ph, R^2^ = H	12	3d, 93
5	1e, R^1^ = *m*-MeO-Ph, R^2^ = H	17	3e, 88
6	1f, R^1^ = *p*-Ph-Ph, R^2^ = H	16.5	3f, 92
7	1g, R^1^ = *p*-Br-Ph, R^2^ = H	18	3g, 85
8	1h, R^1^ = *p*-Cl-Ph, R^2^ = H	20	3h, 84
9	1i, R^1^ = *p*-F-Ph, R^2^ = H	19	3i, 82
10^c^	1j, R^1^ = *p*-CF_3_-Ph, R^2^ = H	19	3j, 82
11^d^	1k, R^1^ = *p*-NO_2_-Ph, R^2^ = H	36	3k, 72
12	1l, R^1^ = 2-thienyl, R^2^ = H	14	3l, 86
13	1m, R^1^ = 1-boc-5-indoly, R^2^ = H	14	3m, 95
14	1n, R^1^ = 2-naphthyl, R^2^ = H	14	3n, 87
15	1o, R^1^ = 6-MeO-2-naphthyl, R^2^ = H	18	3o, 94
16^e^	1p, R^1^ = 9-phen, R^2^ = Me	12	3p, 69

a To a 25 mL flame- and vacuum-dried Schlenk tube was added 1 (0.2 mmol), then the tube was evacuated and backfilled with Ar. The catalyst (3 mol%) was dissolved in 2.5 mL DCM and then the solution was degassed with Ar. The catalyst solution was added to the Schlenk tube. The reaction was allowed to stir at the indicated temperature until TLC indicated complete conversion of 1.

b Isolated yield.

c The reaction was conducted at 10 °C.

d The reaction was conducted at 60 °C for 36 h, the product contained about 20% of 1,3-cyclohexadiene 9k and the total yield was 91%.

e IPrAuNTf_2_ was used as the catalyst instead of JohnPhosAuSbF_6_.

It was a great surprise that compound 2a, in which Ar is a 9-phenanthrenyl group, could not produce 3 in the presence of [JohnPhosAu·MeCN]SbF_6_ and two new compounds 5a and 6a were obtained in 37% and 54% yields, respectively (Table 2, entry 1). Inspired by this discovery, we optimized the reaction conditions for the gold-catalyzed cycloisomerization of compound 2a. With JohnPhosAuOAc, the reaction could not proceed at all (Table 2, entry 2). When the temperature was lowered to 0 °C, compound 5a was obtained in 84% yield, combined with a trace amount of compound 4a and a small amount of compound 6a, in the presence of [JohnPhosAu·MeCN]SbF_6_ (Table 2, entry 3). By elevation of the reaction temperature, compound 6a could be obtained in higher yield in the presence of IPrAuNTf_2_ or [JohnPhosAu·MeCN]SbF_6_ (Table 2, entries 4 and 5) in DCE. When the reaction of 2a was conducted at −20 °C using [JohnPhosAu·MeCN]SbF_6_ as the catalyst, 4a could be afforded in 5% yield combined with a trace amount of 5a (Table 2, entry 6). When the reaction was conducted at −30 °C in the presence of IPrAuNTf_2_, 4a was produced as the major product in 75% yield along with a trace amount of 5a, and 6a could not be detected at all (Table 2, entry 7). Entries 3, 5 and 7 were identified as the optimal conditions for the formation of 5a, 6a and 4a, respectively.

**Table 2:** Optimization of reaction conditions for the gold-catalyzed cycloisomerization of 2a

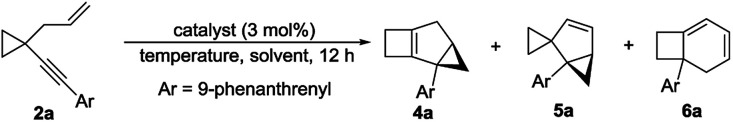						
Entry	Catalyst (3 mol%)	Solvent	Temperature (°C)	Yield^a^ (%)		
				4a	5a	6a
1^b^	[JohnPhosAu(MeCN)]SbF_6_	DCM	rt	N.D.	37	54
2	JohnPhosAuOAc	DCE	rt		N.R.	
**3** b	**[JohnPhosAu(MeCN)]SbF**_**6**_	**DCM**	**0**	**Trace**	**84**	**<5**
4	IPrAuNTf_2_	DCE	80	N.D.	N.D.	90
**5**	**[JohnPhosAu(MeCN)]SbF**_**6**_	**DCE**	**60**	**N.D.**	**N.D.**	**95**
6^b^	[JohnPhosAu(MeCN)]SbF_6_	DCM	−20	5^d^	Trace	N.D.
**7** b ^,^ c	**IPrAuNTf**_**2**_	**DCM**	**−30**	**75**	**Trace**	**N.D.**

a Isolated yield.

b The reaction was quenched with DMS (dimethyl sulfide).

c The reaction was conducted for 8 hours.

d NMR yield.

The substrate scope for the formation of 4 was also explored. When the aryl group was a substituted naphthyl group or a derivative such as pyrene or anthracene, the products 4 could be produced in moderate to good yields, ranging from 43% to 75% (Table 3, 4a–4h). The structure of 4a was determined by 2D-NMR spectroscopy (for details see the ESI†).

**Table 3:** Au(i)-catalyzed cycloisomerization of 2 leading to 4

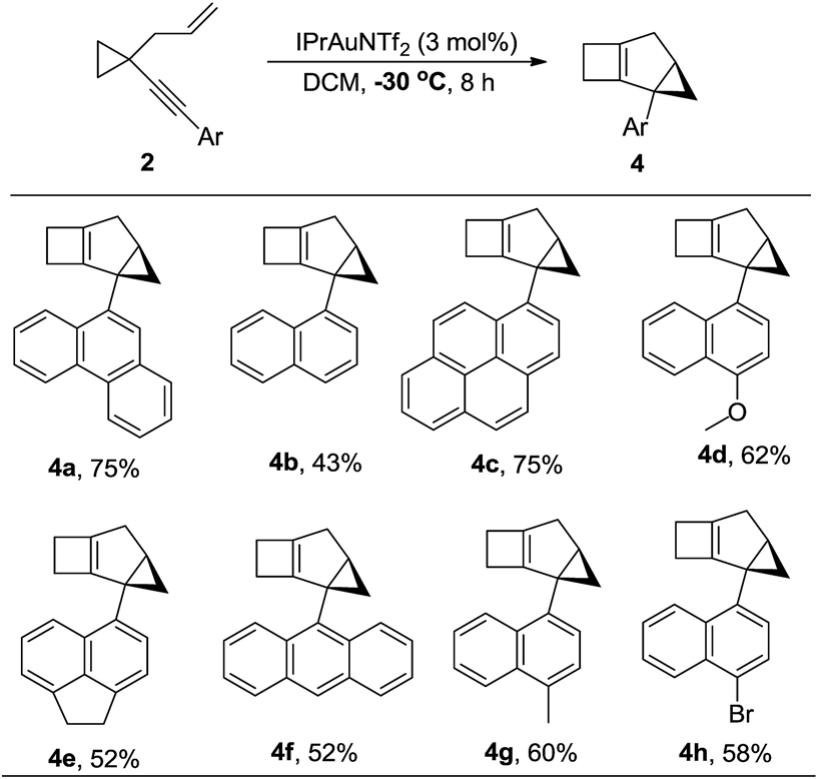

The substrate generality for the formation of the biscyclopropane product 5 was also examined. When the alkynyl substituent was a naphthyl or substituted naphthyl group or its derivatives, the reaction proceeded smoothly to produce products 5 in high yields (Table 4, 5a–5c and 5e–5h); H_4_-naphthyl substrate 2j also worked well (Table 4, 5j); the desired products 5 could also be produced in moderate to high yields when the phenyl group was substituted at the *ortho* position (Table 4, 5k–5m). In the case that the substituent on the allylic position was a methyl group, the reaction also proceeded efficiently to produce the desired product 5p in 78% yield. The structure of 5a was unambiguously determined by X-ray diffraction.^15^ Interestingly, the product 5 was detected as mixtures of two diastereomers. Due to the following reasons, we believe that compound 5 exists as a pair of rotamers: (1) compounds 5f and 5l had no dr value; (2) four peaks could be found in the chiral HPLC resolution (for the details see the ESI†); (3) when the bromine atom in compound 5m was removed, the dr value disappeared (Scheme 12); (4) when the ^1^H-NMR spectrum of compound 5m was recorded at 65 °C in CDCl_3_, the two peaks converged into one peak, indicating that the dr value disappeared at higher temperature (for details, see the ESI†).

**Table 4:** Au(i)-catalyzed cycloisomerization of 2 leading to 5

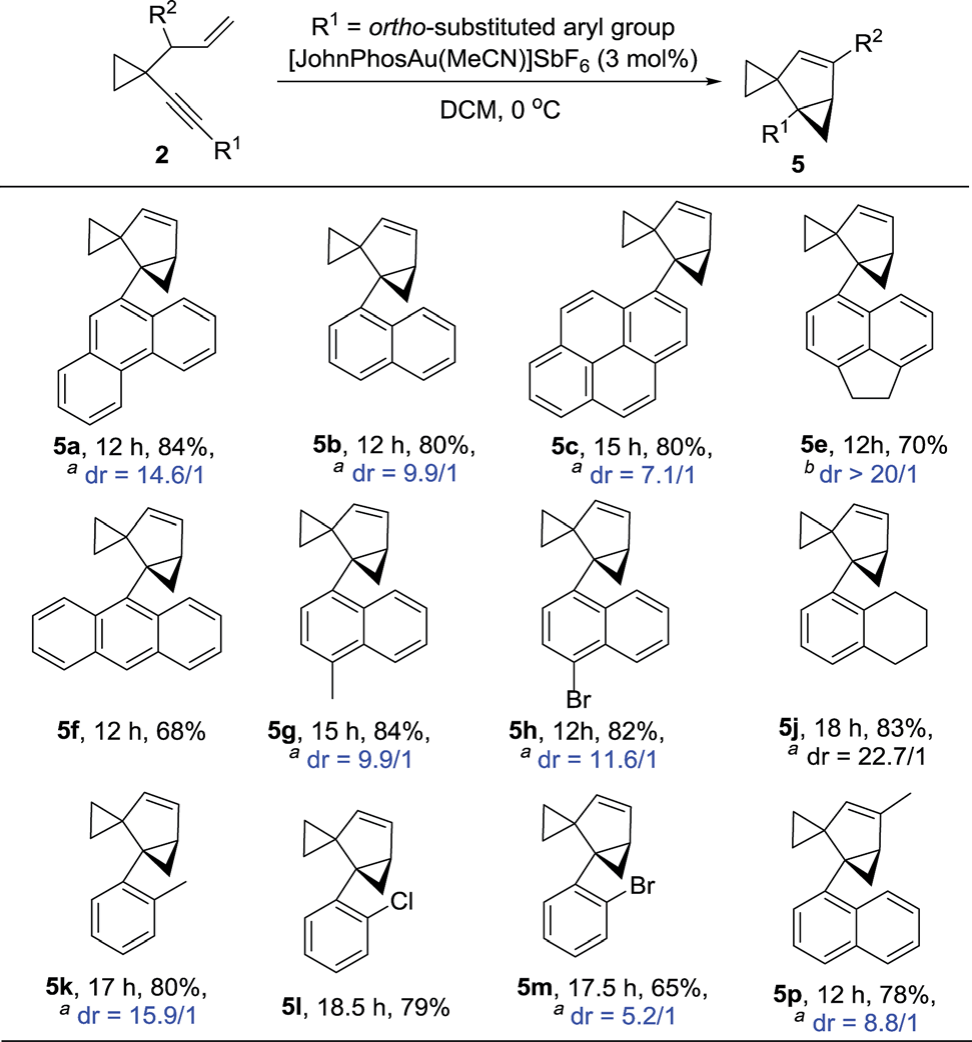

a The dr value was determined by ^1^H-NMR spectroscopy in CDCl_3_.

b The dr value was determined by ^1^H-NMR spectroscopy in CDCl_3_ as well as HPLC resolution.

After further exploration of the substrate scope for the formation of 6, we found that when the substituent on the alkyne group was 1-naphthyl or a substituted 1-naphthyl group or its derivatives, the corresponding products 6b–6i could be obtained in good to excellent yields. The reaction also proceeded smoothly when the phenyl group was substituted at the *ortho* position (Table 5, 6j–6n). In the case that the substituent on the alkyne was a benzyl group, the reaction also proceeded efficiently to produce the desired product 6o in 83% yield. For the substrate in which the allylic position was substituted with an alkyl group, the corresponding product 6p could also be obtained in 91% yield (Table 5). If the alkene unit was substituted at the terminal position, the desired product 6q was produced as a mixture of two diastereomers (dr = 6.4/1) in 90% yield (Table 5). The structure of compound 6 was further confirmed by the X-ray diffraction of compound 6c.^15^

**Table 5:** Au(i)-catalyzed cycloisomerization of 2 leading to 6

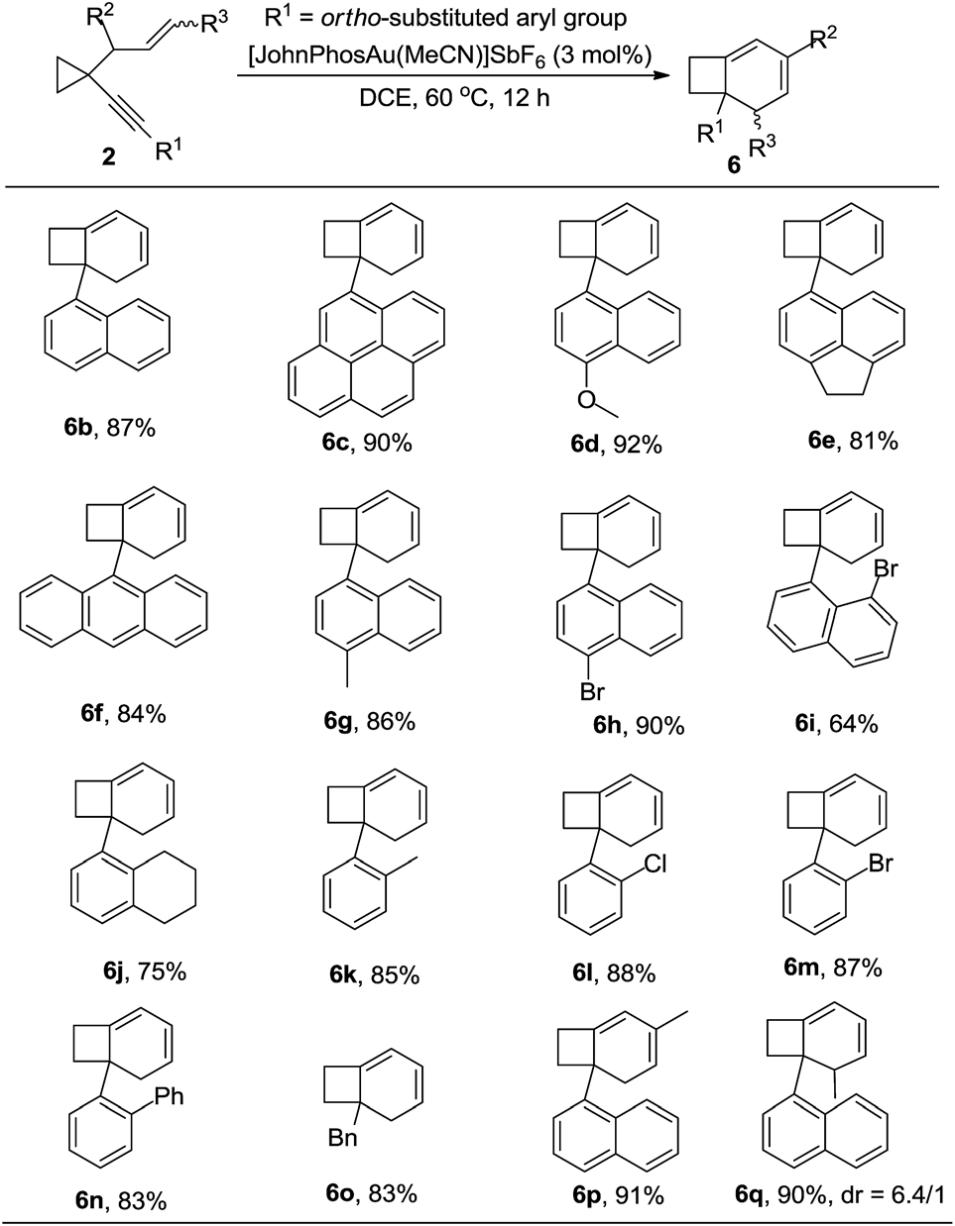

## Deuterium labeling experiments

**Scheme 2:**
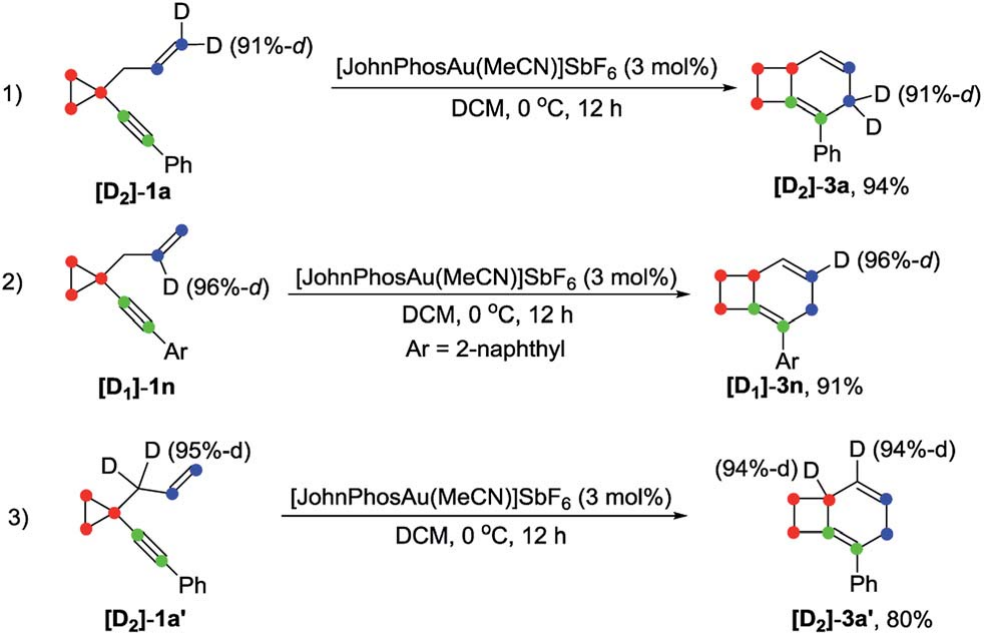
Deuterium labeling experiment for formation of 3.

Deuterium labeling experiments were conducted to gain further insights into the reaction mechanism. For deuterium-labeled substrates [D_2_]-1a and [D_1_]-1n, the corresponding products [D_2_]-3a and [D_1_]-3n were obtained without a deuterium shift, suggesting that there is no carbon rearrangement of the allyl group during the reaction (Scheme 2, eqn (1) and (2)). When the allylic hydrogen atoms were deuterated, compound [D_2_]-3a′ was produced in 80% yield along with one deuterium shift (Scheme 2, eqn (3)).

**Scheme 3:**
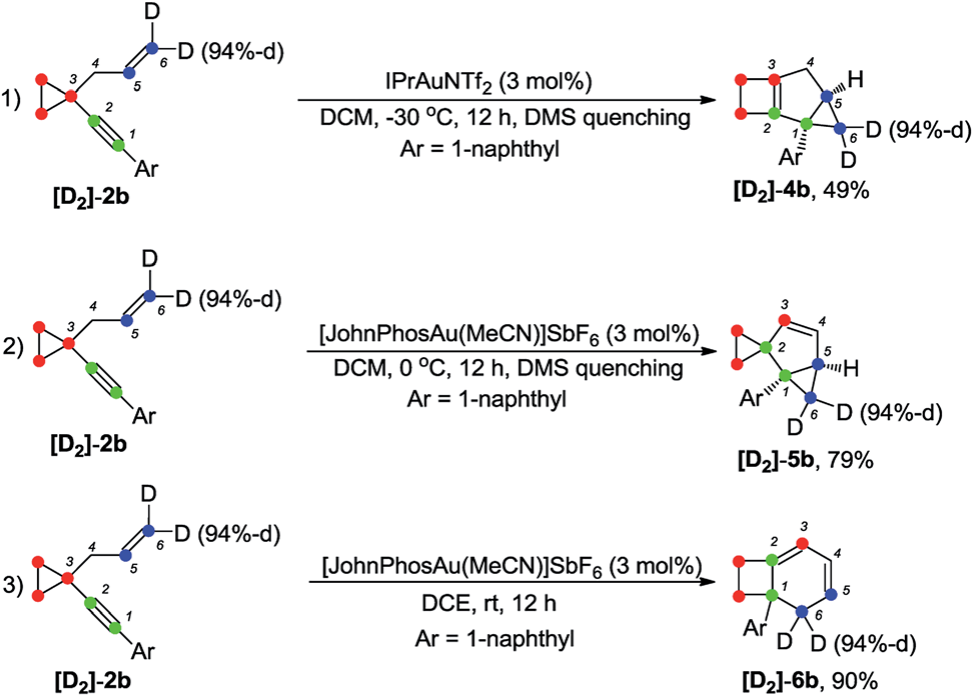
Deuterium labeling experiment ([D_2_]-2b).

**Scheme 4:**
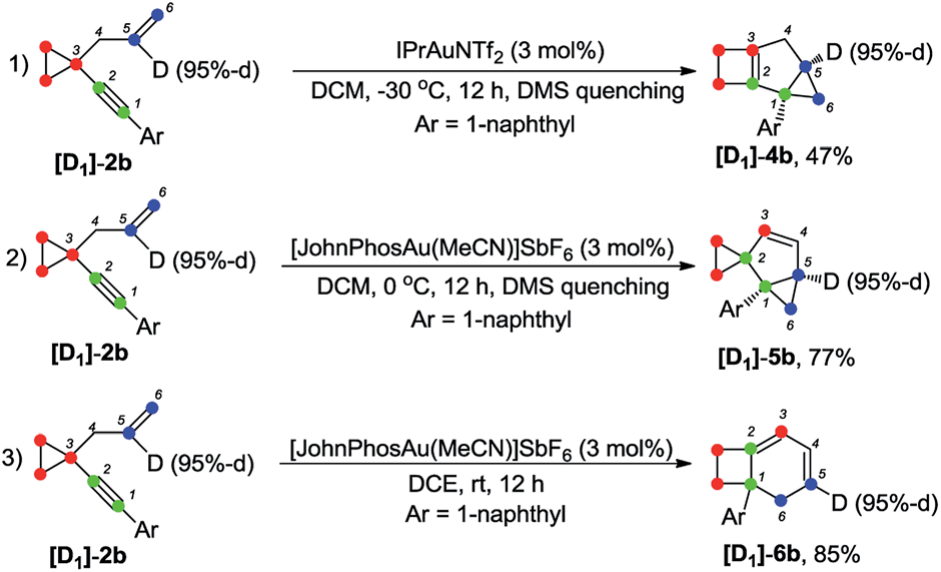
Deuterium labeling experiment ([D_1_]-2b).

The deuterium-labeled compounds [D_2_]-2b and [D_1_]-2b were also synthesized and subjected to the standard reaction conditions for the formation of products 4b, 5b and 6b. The corresponding products [D_2_]-4b, [D_2_]-5b and [D_2_]-6b and [D_1_]-4b, [D_1_]-5b and [D_1_]-6b were produced in high yields with retention of the deuterium content (Scheme 3 and 4). As can be seen from the deuterium labeling experiment, the deuterated C–H bond was not disturbed during the reaction process. Apparently, for the formation of 4b, only the carbon skeleton was rearranged and all the hydrogen atoms remained unaltered during the reaction process; an allylic hydrogen shift was involved during the formation of 5b and 6b.

**Scheme 5:**
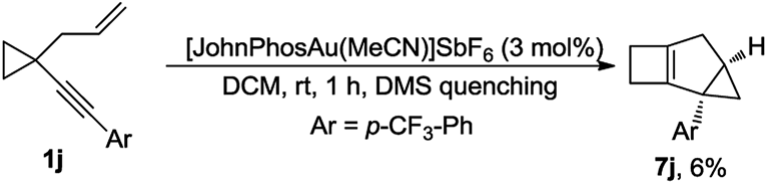
Capture of compound 7j.

It appears that the reactions leading to 3 and 5 or 6 are controlled by the nature of substituent R^1^. The inherent relationship between them stimulated our further investigations. Fortunately, by quenching the reaction of substrate 1j with DMS after 1 h at room temperature, we were able to capture 7j in 6% yield, indicating that 7j was a key intermediate in the reaction that leads to 1,4-cyclohexadiene 3j (Scheme 5; for the details, see the ESI†).

**Scheme 6:**
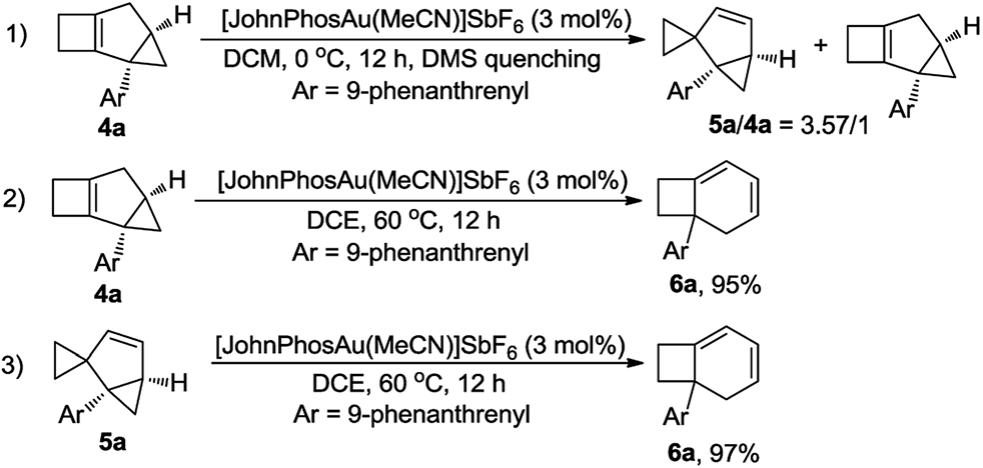
Reactions of 4a and 5a under standard conditions.

Furthermore, the control experiment showed that 5a could be afforded from 4a at 0 °C in the presence of [JohnPhosAu·MeCN]SbF_6_ (Scheme 6, eqn (1)). Under the standard reaction conditions, both compounds 4a and 5a could be transformed to product 6a in almost quantitative yields, indicating that both compounds 4a and 5a are the intermediates for the formation of product 6a (Scheme 6, eqn (2) and (3)).

## Proposed reaction pathways

**Scheme 7:**
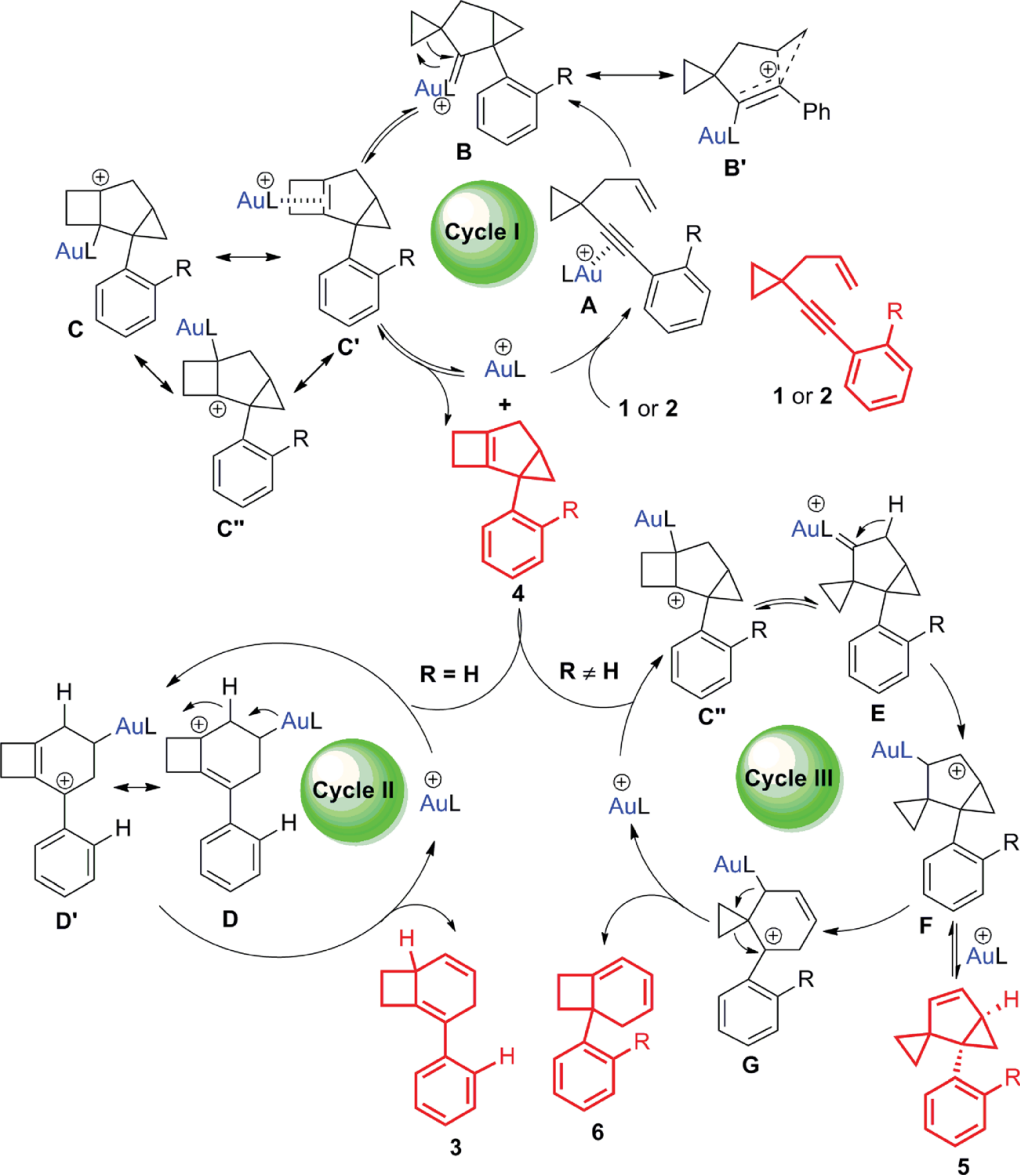
A plausible mechanism for the formation of 3, 4, 5 and 6.

Based on the deuterium labeling experiments, intermediate trapping experiments and theoretical investigations (Schemes 9 and 10), some possible reaction pathways are ruled out and the most reasonable mechanism is proposed in Scheme 7 (for details, see Schemes S9 and S10 in the ESI†). Product 4 is formed through a classical 1,5-enyne cyclization. Coordination of gold(i) to the alkyne moiety of substrate 1 or 2 forms intermediate A. Nucleophilic attack of the alkyne by the alkene unit gives intermediate B or its resonance structure B′. Gold(i) carbenoid-initiated ring expansion produces intermediate C, which probably has other resonance structures illustrated as C′ and C′′. Release of the catalyst from intermediate C affords tricyclic cyclobutene 4 (Scheme 7, cycle I).^17^ An equilibrium probably exists between tricyclic cyclobutene 4 and the gold catalyst to give intermediate C′, which can further undergo subsequent transformations to generate other products. For substrates having an aryl group without an *ortho* substituent, the corresponding intermediate C′ undergoes cleavage of the cyclopropyl ring^16^ to form cationic intermediates D and D′, which are in resonance with each other. Subsequent 1,2-*H* shift followed by release of the cationic Au(i) species results in product 3 (Scheme 7, cycle II). On the other hand, for the substrates having an aryl group with an *ortho* substituent or having a benzyl group, the corresponding intermediate C′ probably has another resonance structure depicted as cationic intermediate C′′, which is probably more favorable.^20^ The cationic intermediate C′′ undergoes ring contraction to form carbenoid intermediate E.^4*p*,18^ 1,2-*H* shift of intermediate E produces intermediate F, which is in equilibrium with compound 5. Carbocation-initiated cyclopropane ring opening of intermediate F forms intermediate G. Finally, compound 6 is obtained after the ring expansion process (Scheme 7, cycle III).

## Rationalization of *ortho*-substituent effects

**Scheme 8:**
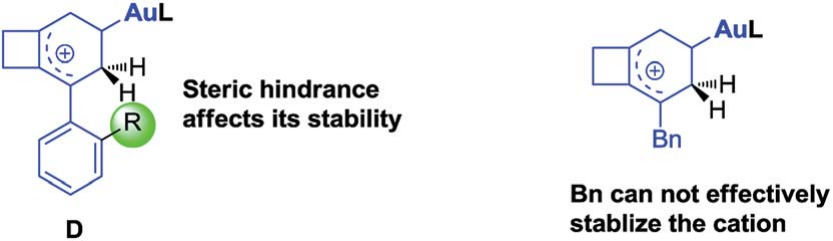
Our speculation for the reaction path divergence.

For substrate 1 having an aryl group without an *ortho*-substituent, 1,4-cyclohexadiene 3 could be produced. In contrast, substrate 2 having an aryl group with an *ortho*-substituent afforded three different products. Based on the proposed reaction mechanism, we speculate that the stability of intermediate D is the key point to affect the reaction path. As depicted in Scheme 8, when the phenyl group is substituted at the *ortho* position, it cannot effectively stabilize the cationic intermediate since the steric hindrance makes the coplanar conformation of the phenyl ring and the allylic carbocation become unfavorable in energy. Furthermore, the benzyl group is also not good enough to stabilize the carbocation intermediate. Thus, in all these cases, the energy level of intermediate D is high and the reaction probably prefers to undergo cycle III.

**Scheme 9:**
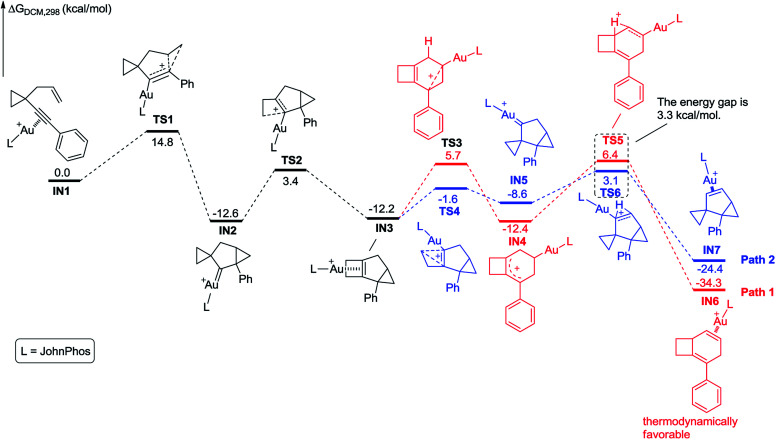
Calculated reaction pathway for the reaction of 1a having an aryl group without an *ortho* substituent.

To understand these *ortho*-substituent effects, we performed DFT calculations on the possible reaction pathways using substrates 1a and 2m. For substrate 1a, the reaction energy profile is depicted in Scheme 9. Initially, coordination of the Au(i) catalyst to the alkyne moiety of substrate 1a generates gold complex IN1. The gold complex IN1 undergoes a 6-*endo*-dig cyclization to give a gold carbene intermediate IN2*via* transition state TS1 with an energy barrier of 14.8 kcal mol^−1^. Subsequently, the intermediate IN2 undergoes ring enlargement *via* transition state TS2 with an energy barrier of 16.0 kcal mol^−1^, producing another intermediate IN3. The intermediate IN3 can undergo two possible reaction pathways to obtain products 3 and 5. In Path 1, the cleavage of the cyclopropane ring *via*TS3 with an energy barrier of 17.9 kcal mol^−1^ leads to the carbocation intermediate IN4. Subsequently, 1,2-*H* shift of the intermediate *via*TS5 leads to intermediate IN6, which undergoes deauration to give product 3. Transition state TS5 is located 18.6 kcal mol^−1^ above intermediate IN3 and 0.7 kcal mol^−1^ above transition state TS3, indicating that the 1,2-*H* shift step is the rate-limiting step for Path 1. Another possible reaction pathway (Path 2) for the carbocation intermediate IN3 is skeletal rearrangement *via*TS4 with an energy barrier of 10.6 kcal mol^−1^, leading to gold carbene intermediate IN5. Intermediate IN5 also undergoes 1,2-*H* shift *via*TS6, affording intermediate IN7, which undergoes deauration to give product 5. Transition state TS6 is located 15.3 kcal mol^−1^ above intermediate IN3 and 4.7 kcal mol^−1^ above transition state TS4, indicating that the 1,2-*H* shift step is also the rate-limiting step for Path 2. Transition state TS5 is higher in energy than transition state TS6 by 3.3 kcal mol^−1^, and intermediates IN4 and IN6 along Path 1 are thermodynamically more stable than intermediates IN5 and IN7 along Path 2, by 3.8 kcal mol^−1^ and 9.9 kcal mol^−1^, respectively. These calculation results indicate that Path 1 is thermodynamically favorable and the reaction of 1a is thermodynamically controlled, which may account for the fact that product 3 is experimentally obtained as the major product if using 1a as substrate. For comparison, we switched the ligand to PPh_3_, and also investigated the possible reaction pathways of substrate 1a (for details, see Scheme S11 in the ESI†). The DFT calculation results indicate that the phosphorus ligand does not significantly affect the reaction energy profile. This is in line with the experimental findings that the product 3 can be obtained in acceptable yield in the presence of [PPh_3_AuCl]/AgSbF_6_. From a practical use point of view, the JohnPhos ligand is more stable due to its steric bulkiness in our reaction conditions for gold catalysis. Moreover, for the comparison with the substrates having an *ortho*-substituent, JohnPhos ligand is chosen as the primary ligand for the gold catalysis.

**Scheme 10:**
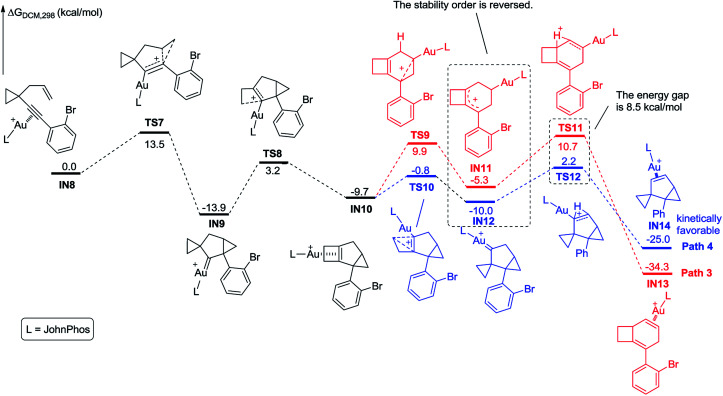
Calculated reaction pathway for the reaction of 2m having an aryl group with an *ortho* substituent.

**Scheme 11:**
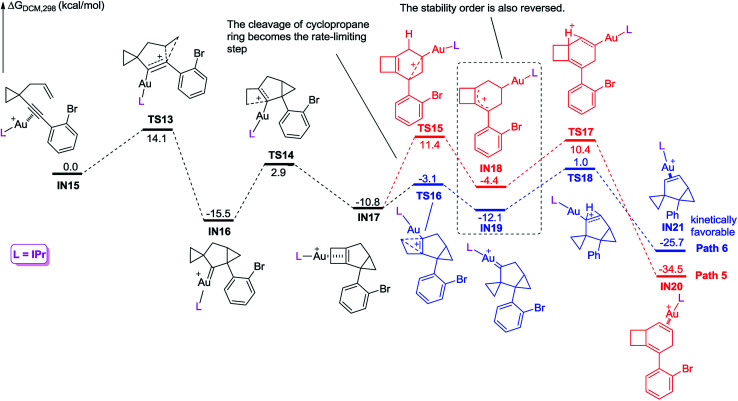
Calculated reaction pathway for the reaction of 2m having an aryl group with an *ortho* substituent, using IPr as the ligand.

We further investigated the reaction energy profiles for the reaction of substrate 2m having an aryl group with *ortho*-substituent Br, and the results are shown in Scheme 10. In a similar manner, coordination of the Au(i) catalyst to the alkyne moiety of substrate 2m generates gold complex IN8. The gold complex IN8 undergoes a 6-*endo*-dig cyclization to give a gold carbene intermediate IN9*via* transition state TS7, with an energy barrier of 13.5 kcal mol^−1^. Subsequently, the intermediate IN9 undergoes ring enlargement *via* transition state TS8 with an energy barrier of 17.1 kcal mol^−1^, producing another intermediate IN10. The intermediate IN10 can also undergo two possible reaction pathways to obtain products 3 and 5. In Path 3, the cleavage of the cyclopropane ring *via*TS9 with an energy barrier of 19.6 kcal mol^−1^ leads to the carbocation intermediate IN11. Subsequently, 1,2-*H* shift of the intermediate *via*TS11 leads to intermediate IN13, which undergoes deauration to give product 3. Transition state TS11 is located 20.4 kcal mol^−1^ above intermediate IN10 and 0.8 kcal mol^−1^ above transition state TS9, indicating that the 1,2-*H* shift step is the rate-limiting step for Path 3. Another possible reaction pathway (Path 4) for the carbocation intermediate IN10 is skeletal rearrangement *via*TS10 with an energy barrier of 8.9 kcal mol^−1^, leading to gold carbene intermediate IN12. Intermediate IN12 also undergoes 1,2-*H* shift *via*TS12, affording intermediate IN14, which undergoes deauration to give product 5. Transition state TS12 is located 11.9 kcal mol^−1^ above intermediate IN10 and 3.0 kcal mol^−1^ above transition state TS10, indicating that the 1,2-*H* shift step is also the rate-limiting step for Path 4. Due to the *ortho* substituent effect, the carbocation intermediate IN11 is less stable than the gold carbene intermediate IN12 by 4.7 kcal mol^−1^; the energy gap between transition states TS11 and TS12 is increased to 8.5 kcal mol^−1^, which is significantly larger than that between TS5 and TS6 (3.3 kcal mol^−1^), indicating that the intermediate IN11 can less easily cross over this energy barrier. The reaction using substrate 2m is probably kinetically controlled, thus the kinetically favorable product 5 is obtained. The calculation results can explain why using a substrate having an aryl group with an *ortho*-substituent can give product 5 as the major product in experiments. For comparison, we switched the ligand to IPr, and also investigated the possible reaction pathways of substrate 2m, and the results are shown in Scheme 11. In general, the calculated reaction energy profiles using IPr as the ligand are similar to those using JohnPhos as the ligand. The carbocation intermediate IN18 is less stable than the gold carbene intermediate IN19 by 7.7 kcal mol^−1^, which is similar to their analogues IN11 and IN12, indicating that the key intermediates' stabilities are hardly influenced by the phosphorus ligand. It is notable that the cleavage of the cyclopropane ring *via*TS15 has an energy barrier of 22.2 kcal mol^−1^, which is slightly higher than that of the 1,2-*H* shift step *via*TS17 in Path 5, thus the cleavage of the cyclopropane ring becomes the rate-limiting step. Moreover, the energy barrier (22.2 kcal mol^−1^) of the rate-limiting step along Path 5 is higher than that (20.4 kcal mol^−1^) of the rate-limiting step along Path 3, indicating that it is more difficult to obtain product 3 using IPr as the ligand. This result partially agrees with the experimental finding that a poor yield of product 3 was obtained using IPr as the ligand. There is no significant difference between the reaction energy profile along Path 4 and that along Path 6. The experimental results in Table 2 show that the catalysts affect the product selectivity, probably mainly due to the temperature effect, and are not significantly influenced by the ligand effect.

## Rationalization of the temperature effect

The sterically bulky and electron-rich IPr ligand was crucial for the selective formation of product 4 at −30 °C because the gold complex IPrAuNTf_2_ also becomes sterically bulky and electron-rich and thus will be less reactive to activate the alkene unit in 4. On the other hand, JohnPhos is a bulky phosphine ligand as well; however, it is not as electron-rich as IPr. Thus, compound 4 could be transformed into compound 5 at 0 °C. At higher temperature, compound 6 was produced as the final product.

**Scheme 12:**
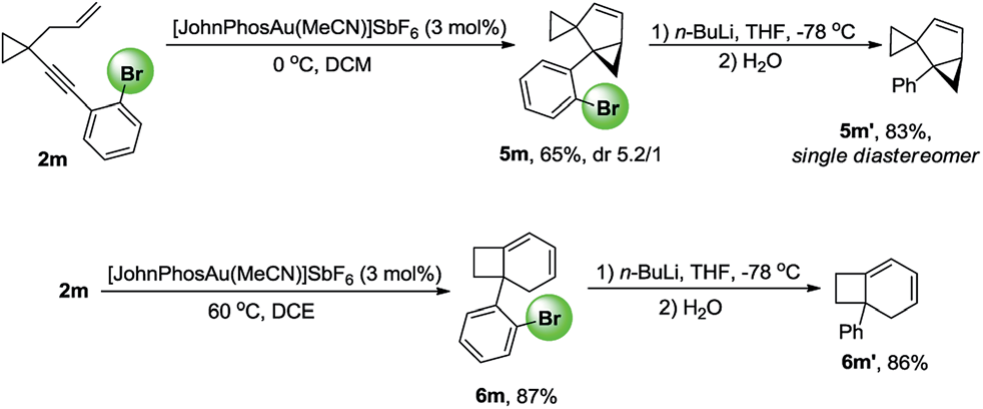
Removable bromine atom controls the reaction outcome.

Compounds 5m and 6m could be transformed to compounds 5m′ and 6m′ by halogen–lithium exchange and subsequent quenching with water, indicating that the bromine atom at the *ortho* position can serve as a removable directing group to control the reaction pathway, and it can be easily removed when the reaction is complete (Scheme 12).

In conclusion, a novel gold(i)-catalyzed cycloisomerization of 1,5-enynes containing a cyclopropane ring has been developed. The cyclopropane functionality in the substrates has a great influence on the reaction pathway,^19^ and the suggested gold carbene intermediate I involving two cyclopropyl moieties is critical to result in the divergent reaction pathways. With this methodology, cyclobutane-fused 1,4-cyclohexadiene, 1,3-cyclohexadiene, tricyclic cyclobutene and biscyclopropane derivatives can be selectively synthesized in high yields. A plausible mechanism has been proposed according to the deuterium labeling and intermediate trapping experiments and theoretical investigations. The dramatic *ortho*-substituent effects have been investigated through DFT calculations, which rationalized the experimental findings. Further efforts to expand the application of this novel gold(i)-catalyzed reaction are underway, and the results will be published in due course.

## Computational methods

All DFT calculations were performed with the Gaussian 09 program.^21^ The geometries of all minima and transition states have been optimized using the PBE1PBE functional.^23^ The SDD basis set and pseudopotential were used for the gold atom, and the 6-31G(d) basis set was used for other atoms. The subsequent frequency calculations on the stationary points were carried out at the same level of theory to ascertain the nature of the stationary points as minima or first-order saddle points on the respective potential energy surfaces. All transition states were characterized by one and only one imaginary frequency pertaining to the desired reaction coordinate. The intrinsic reaction coordinate (IRC) calculations were carried out at the same level of theory to further authenticate the transition states. The conformational space of the flexible systems has first been searched manually. Thermochemical corrections to 298.15 K have been calculated for all minima from unscaled vibrational frequencies obtained at this same level. The solvent effect was estimated by the IEFPCM method^22^ with radii and nonelectrostatic terms for the SMD solvation model^24^ in dichloromethane (*ε* = 8.93). Solution-phase single point energy calculations (SDD basis set and pseudopotential used for the gold atom, and the 6-31+G(d,p) basis set used for other atoms) were performed based on the gas-phase optimized structures.

## Supplementary Material

SC-007-C6SC00058D-s001

SC-007-C6SC00058D-s002
