# Supplementary material for: Divergent reaction pathways in gold-catalyzed cycloisomerization of 1,5-enynes containing a cyclopropane ring: dramatic ortho substituent and temperature effects
Source: Chem Sci. 2016 Mar 4;7(7):4318–28. doi: 10.1039/c6sc00058d (PMC6013813; doi:10.1039/c6sc00058d)
Supplement: SC-007-C6SC00058D-s001 [file SC-007-C6SC00058D-s001.pdf]

Electronic Supplementary Material (ESI) for Chemical Science.
This journal is © The Royal Society of Chemistry 2016

Supporting Information

Divergent Reaction Pathways in Gold Catalyzed Cycloisomerization of 1,5-enynes Containing a Cyclopropane: Dramatic Ortho Substituent and Temperature Effects

Gen-Qiang Chen, Wei Fang, Yin Wei, Xiang-Ying Tang,* and Min Shi**

State Key Laboratory of Organometallic Chemistry,

Shanghai Institute of Organic Chemistry,

Chinese Academy of Sciences, 345 Lingling Road, Shanghai, 200032, China.

weiyin@sioc.ac.cn, siocxiangying@sioc.ac.cn, mshi@sioc.ac.cn. *Fax* 86-21-64166128

CONTENTS

1. General Remarks……………………………………………….……………………………S2
2. General or Representative Reaction Procedure.……………….…………………………….….S3-S7
3. Procedures for the Synthesis of the Deuterated Compounds.………………….….…………S8-S11
4. Optimization of the Reaction Conditions..……………………………………..…….………S12-S13
5. Spectroscopic Data.……….………………………………………………….………..….......S14-S137
6. Evidences for the Existence of Rotamer……………………………….………...…...........S138
7. ^{1}H and ^{13}C NMR Spectra of the Compound **7j**……………………………………...……..S139
8. ^{1}H NMR Spectra of the Deuterated Compounds…………………………………..…...….S140-S146
9. Crystallographic Information……….…………………….…………………….……......S147-S149
10. 2D-NMR Data of Compounds **3a**, **4b**, **5a** and **6a**…………………………..………..….......S150-165
11. Three possible routes for the formation of product **3** from **4**……………...…………………......S166
12. Another mechanism for the formation of products **5b** and **6b** through intermediate **4b'**……..S167
13. Computational Details…………………………..………..…...S168-169
14. Archive Entries………………………………………………………………………...S170-220
15. Reference………………………………………………………………………..…….....S221

1. General Remarks. ^{1}H and ^{13}C NMR spectra were recorded at the 300 and 75 MHz or the 400 and 100 MHz, respectively. Mass and HRMS spectra were recorded by EI or ESI method. Organic solvents used were dried by standard methods when necessary. 1,2-Dichloroethane and dichloromethane were distilled from CaH_2 under argon (Ar) atmosphere. Commercially obtained reagents were used without further purification. Flash column chromatography was performed using 300-400 mesh silica gel. For thin-layer chromatography (TLC), silica gel plates (Huanghai GF254) were used.

2. General or Representative Reaction Procedure.

Representative procedures for the preparation of substrates:

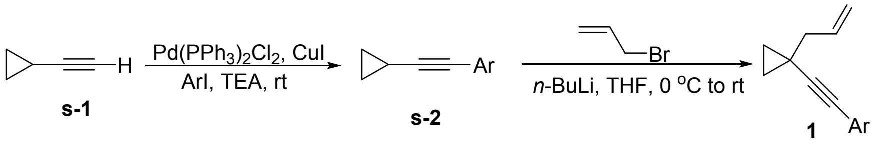

s-1 **s-2** **1**

Scheme S1

The substrate **1a** was synthesized according to Scheme S1. The compound **s-2** was synthesized according to the previously reported procedure.[1]

Synthesis of substrate **1a**: To a 100 mL flame and vacuum dried flask was added cyclopropylphenylacetylene **s-2** (4.33 g, 31.16 mmol), then the flask was evacuated and backfilled with Ar and then *n*-BuLi (2.5 M, 15 mL, 37.5 mmol) was added slowly at 0 °C. The reaction mixture was warmed to rt gradually and allowed to be stirred at rt for 1 h, and then allyl bromide (3.0 mL, 37.4 mmol) was added slowly at 0 °C. The reaction mixture was stirred overnight. After that, the reaction was quenched with water, and then extracted with ethyl acetate (30 mL*3). The combined organic phase was dried over Na_2SO_4 (anhydrous) and evaporated under reduced pressure. The crude product was purified by column chromatography using PE as an eluent. The product was afforded as a colorless oil (3.12 g, yield = 58%).

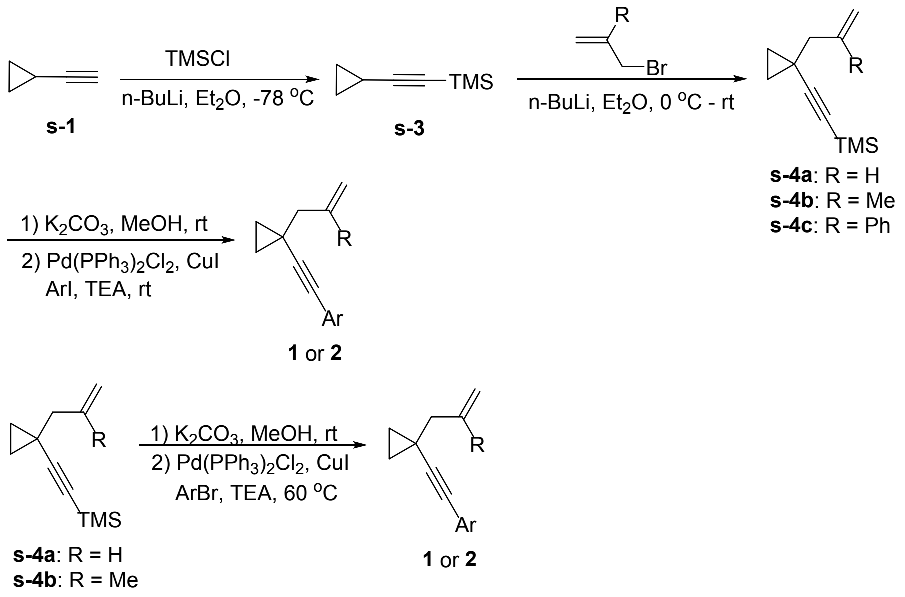

Scheme S2

The substrates **1b**, **1c**, **1d**, **1e**, **1f**, **1g**, **1h**, **1i**, **1j**, **1k**, **1l**, **1m**, **1n**, **1o**, **1p**, **2a**, **2b**, **2c**, **2d**, **2e**, **2f**, **2g**, **2h**, **2i**, **2j**, **2k**, **2l**, **2m** and **2n** were synthesized according to Scheme S2. The corresponding iodide was synthesized according to the previously reported procedures.[2]

The compound **s-3** was synthesized according to the previously reported procedure:[3] To a 500 mL flame and vacuum dried three-neck flask was added compound **s-1** (20.3 mL, 240 mmol) and 100 mL THF under Ar, then *n*-BuLi (2.5 M, 97 mL, 240 mmol) was added slowly at -78 °C. After the addition was complete, the reaction mixture was allowed to stir at -78 °C for 2 h. TMSCl (33 mL, 240 mmol) was added to the flask slowly at -78 °C. The reaction mixture was allowed to stir at -78 °C for 2 h, and then warmed to rt gradually. After 5 h, the reaction was quenched with water. The reaction mixture was extracted with Et_2O, and the combined organic phase was dried over anhydrous sodium sulfate. The crude product was purified by vacuum distillation, and the product **s-3** (100 mbar, 80 °C fraction) was obtained as a colorless oil (23.0 g, yield = 69%).

Synthesis of compound **s-4**: To a 250 mL flame and vacuum dried three-neck flask was added compound **s-3** (21.36 g, 154.5 mmol) and 100 mL Et_2O under Ar, then *n*-BuLi (1.6 M, 100 mL, 160 mmol). After the addition was complete, the reaction mixture was allowed to stir at rt for 14 h. Allyl bromide (14.0 mL, 160 mmol) was added slowly to the reaction mixture to maintain gentle reflux. After the addition was complete, the reaction mixture was warmed to rt. The reaction was quenched with water and the reaction mixture was extracted with Et_2O, and the combined organic phase was dried over anhydrous sodium sulfate. The product **s-4** was purified by vacuum distillation (50 mbar, 90-100 °C) and the desired product was afforded as a colorless oil (17.2 g, yield = 63%).

Synthesis of compound **1f**: To a 50 mL flask was added **s-4a** (850 mg, 4.5 mmol) and K_2CO_3 (700 mg, 5.0 mmol) and 2.5 mL methanol, the reaction mixture was allowed to stir at rt for 2 hours. Then *p*-Ph-PhI (1.12 g, 4.0 mmol) and 10 mL TEA (triethylamine) were added into the reaction system, respectively. The reaction mixture was solidified by liquid N_2, and then evacuated and backfilled with Ar for 3 times. $Pd(PPh_3)_2Cl_2$ (70 mg, 0.01 mmol) and CuI (38 mg, 0.02 mmol) were added under Ar. The reaction mixture was allowed to stir at rt overnight. The product was purified by column chromatography using PE as an eluent. The corresponding product **1f** was afforded as a colorless oil (1.053 g, yield = 91%).

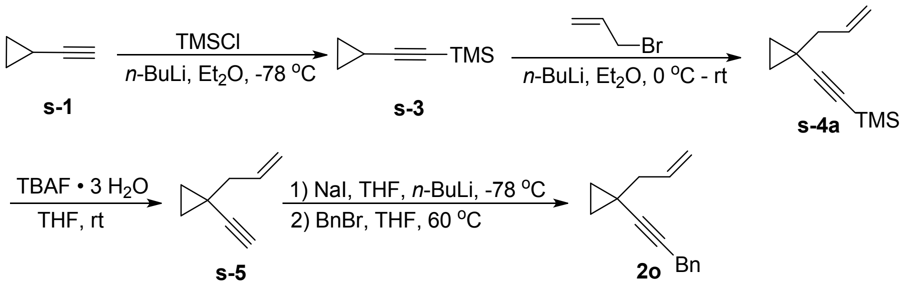

Scheme S3

The substrate **2o** was synthesized according to Scheme S3.

Synthesis of compound **s-5**: To a 50 mL flask was added **s-4a** (5.35 g, 30 mmol), THF (20 mL) and $TBAF{\cdot}3H_2O$ (11.025 g, 35 mmol), the reaction mixture was allowed to stir at rt overnight. The reaction mixture was filtered through a short pad of silica gel using *n*-pentane as an eluent. The product was purified by distillation under ambient pressure, and the 60 ^{o}C fraction was collected as mixtures of THF, DCM and **s-5** (4.315 g, yield = 72%, determined by ^{1}H NMR). The mixture was used in the next step without further purification.

Synthesis of compound **2o**: To a 100 mL flask was added NaI (1.09 g), then the flask was flame and vacuum dried and **s-5** (mixtures of DCM and THF, 1.5 g, 7.5 mmol) was added and then THF (20 mL) and *n*-BuLi (2.5 M, 3.25 mL, 8.0 mmol) were added at -78 ^{o}C. The reaction mixture was allowed to stir at -78 ^{o}C for 2.5 h. BnBr (1.10 mL, 9.0 mmol) was added and the reaction mixture was allowed to stir at 60 ^{o}C with an oil bath overnight. The product was purified by column chromatography using PE as an eluent. Product **2o** was afforded as a colorless oil (0.863 g, yield = 59%).

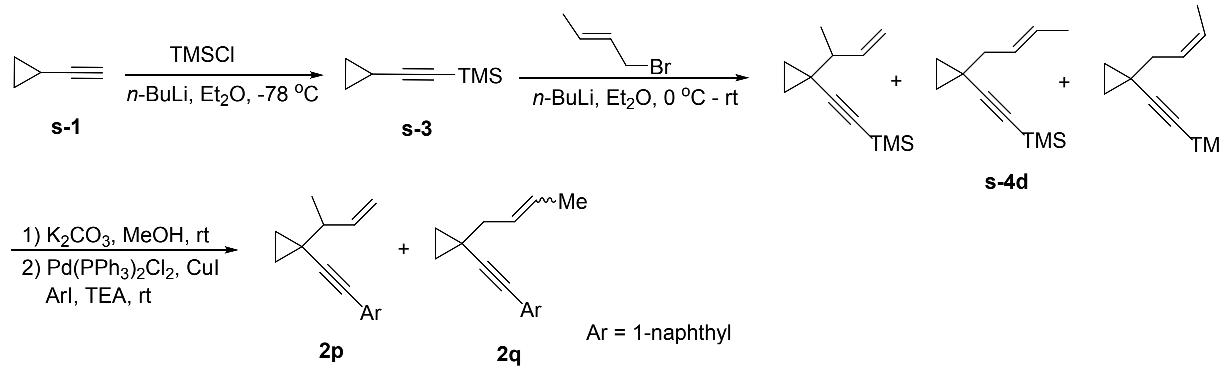

Scheme S4

The substrates **2p** and **2q** were synthesized according to Scheme S4.

Synthesis of compound **s-4d**: To a 250 mL flame and vacuum dried three neck flask was added **s-3** (16.22 g, 118 mmol) and Et_2O (50 mL), then *n*-BuLi (50 mL, 125 mmol) was added at 0 ^{o}C. The reaction mixture was warmed to rt and allowed to stir at rt overnight. (E)-1-bromobut-2-ene (19 g, 125 mmol) was added slowly at 0 ^{o}C. The reaction was quenched with H_2O and the crude product was purified by vacuum distillation. The product **s-4d** (50 mbar, 100-120 ^{o}C fraction) was afforded as a colorless oil (10.053 g, yield = 44%, mixtures of three compounds).

Synthesis of compounds **2p** and **2q**: To a 50 mL flask was added **s-4d** (1.00 g, 5 mmol) and K_2CO_3 (1.00 g, 7.2 mmol) and 2.5 mL methanol, the reaction mixture was allowed to stir at rt for 2 hours. Then 2-iodo-naphthalene (1.27 g, 5.0 mmol) and 10 mL TEA (triethylamine) were added. The reaction mixture was solidified by liquid N_2, then evacuated and backfilled with Ar for 3 times. $Pd(PPh_3)_2Cl_2$ (70 mg, 0.01 mmol) and CuI (38 mg, 0.02 mmol) were added under Ar. The reaction mixture was allowed to stir at rt overnight. The product was purified by column chromatography using PE as an eluent. The product was afforded as a colorless oil (**2p**, 0.103 g, yield = 8%; **2q**, 0.563 g, yield = 46%).

Representative procedure for the gold(I) catalyzed cascade reactions of substrates 1:

To a 25 mL flame and vacuum dried Schlenk tube was added substrate **1a** (37 mg, 0.2 mmol), the reaction tube was evacuated and backfilled with Ar. [JohnPhosAu·MeCN]SbF_6 (3.5 mg, 3 mol%) was dissolved in 2.0 mL DCM, and the air was bubbled out with Ar. Then the catalyst solution was added to the Schlenk tube at 0 ^{o}C. The reaction mixture was allowed to stir at 0 ^{o}C and the reaction

was quenched with DMS (dimethyl sulfide) when the reaction was complete. The product was purified by column chromatography using PE as an eluent. The product **3a** was afforded as a colorless oil (34 mg, yield = 92%).

Representative procedure for the gold(I) catalyzed cascade reactions of substrates 2 leading to 4:

To a 25 mL flame and vacuum dried Schlenk tube was added substrate **2a** (57 mg, 0.2 mmol) and 2.0 mL dry DCM under Ar and the reaction was allowed to stired at -30 ^{o}C for 30 min. Then $IPrAuNTf_2$ (4.0 mg, 3.0 mol%) was added under Ar. The reaction mixture was allowed to stir at -30 ^{o}C for 8 h. The reaction was quenched with DMS (dimethyl sulfide). The product was purified by column chromatography using PE as an eluent. The product **4a** was afforded as a white solid (42 mg, yield = 75%).

Representative procedure for the gold(I) catalyzed cascade reactions of substrates 2 leading to 5:

To a 25 mL flame and vacuum dried Schlenk tube was added substrate **2a** (57 mg, 0.2 mmol) and 2.0 mL dry DCM under Ar and the reaction was allowed to stired at -0 ^{o}C for 30 min. Then [JohnPhosAu·MeCN]SbF_6 (3.5 mg, 3.0 mol%) was added under Ar. The reaction mixture was allowed to stir at 0 ^{o}C for 12 h. The reaction was quenched with DMS (dimethyl sulfide). The product was purified by column chromatography using PE as an eluent. The product **5a** was afforded as a white solid (47 mg, yield = 84%).

Representative procedure for the gold(I) catalyzed cascade reactions of substrates 2 leading to 6:

To a 25 mL flame and vacuum dried Schlenk tube was added substrate **2a** (57 mg, 0.2 mmol) and [JohnPhosAu·MeCN]SbF_6 (3.5 mg, 3.0 mol%), then the reaction tube was evacuated and backfilled with Ar. 2.0 mL dry DCE was added to the reaction tube, and the reaction mixture was allowed to stir at 60 ^{o}C with an oil bath for 12 h. The product was purified by column chromatography using PE as an eluent. The product **6a** was afforded as a white solid (54 mg, yield = 95%).

3. Procedures for the Synthesis of the Deuterated Compounds.

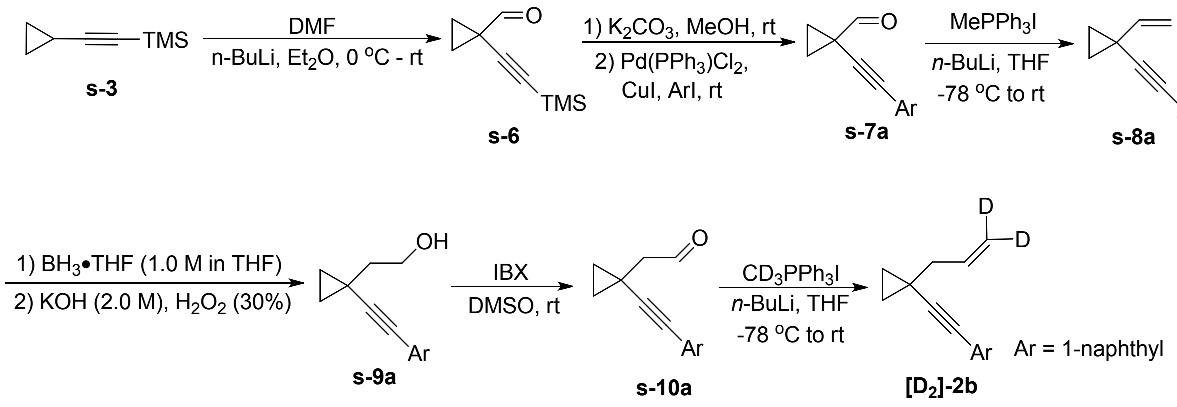

Scheme S5

The deuterated compound **[D$_2$]-2b** was synthesized according to Scheme S5.

Compound **s-7a** was synthesized according to the previously reported procedure:[1] To a 100 mL flask was added **s-6** (1.66 g, 10 mmol), K_2CO_3 (1.38 g, 10 mmol) and 4 mL MeOH, the reaction mixture was allowed to stir at rt for 1 h. 1-iodo-naphthalene (2.54 g, 10 mmol) and 15 mL TEA (triethylamine) were added. The reaction mixture was solidified by liquid nitrogen, then evacuated and backfilled with Ar for 3 times. $Pd(PPh_3)_2Cl_2$ (100 mg, 0.014 mmol) and CuI (59 mg, 0.031 mmol) were added under Ar. The reaction mixture was allowed to stir at rt overnight. The compound **s-7a** was purified by column chromatography using PE as an eluent (1.418 g, yield = 64%).

Synthesis of compound **s-8a**: To a 100 mL flame and vacuum dried flask was added $MePPh_3I$ (4.04 g, 10 mmol), then the flask was evacuated and backfilled with Ar. THF (20 mL) was added, and then *n*-BuLi (2.5 M, 4 mL, 10 mmol) was added slowly at -78 ^oC. After 20 min., **s-7a** (1.10 g) dissolved in 10 mL THF was added slowly. The reaction mixture was warmed to rt gradually, and allowed to stir at rt for 6 h. The product was purified by column chromatography using PE as an eluent. The compound **s-8a** was afforded as a colorless oil (880 mg, yield = 81%).

Synthesis of compound **s-9a**: To a 100 mL flame and vacuum dried flask (containing 218 mg **s-8a**) was added THF (15 mL), then BH_3 (1.0 M in THF,) was added slowly at 0 ^oC. After 30 min., NaOH (2.0 M, 2.5 mL) was added. After 5 min., H_2O_2 (30%, 2.5 mL) was added. The reaction was quenched with sodium thiosulfate. The product was purified by column chromatography (eluent: PE/EA = 4/1). The compound **s-9a** was afforded as a colorless oil (108 mg, yield = 46%).

Synthesis of compound **s-10a**: To a 50 mL flask was added **s-9a** (914 mg, 3.87 mmol) and 10 mL DMSO, then 1.4 g IBX was added. The reaction mixture was allowed to stir at rt overnight. The product was purified by column chromatography (eluent: PE/EA = 20/1). The product was afforded as a colorless oil (624 mg, yield = 69%).

Synthesis of compound **[D$_2$]-2b**: To a 100 mL flame and vacuum dried flask was added CD_3PPh_3I (2.04 g, 5 mmol), then the flask was evacuated and backfilled with Ar. THF (20 mL) was added, and then *n*-BuLi (2.5 M, 2.0 mL, 5 mmol) was added slowly at -78 ^oC. After 20 min., **s-10a** (624 mg, 2.66 mmol) dissolved in 10 mL dry THF was added to the flask. The reaction mixture was allowed to warm to rt gradually and stirred at rt for 6 hours. The product **[D$_2$]-2b** was purified by column chromatography using PE as an eluent. The product was afforded as colorless oil (578 mg, yield = 92%).

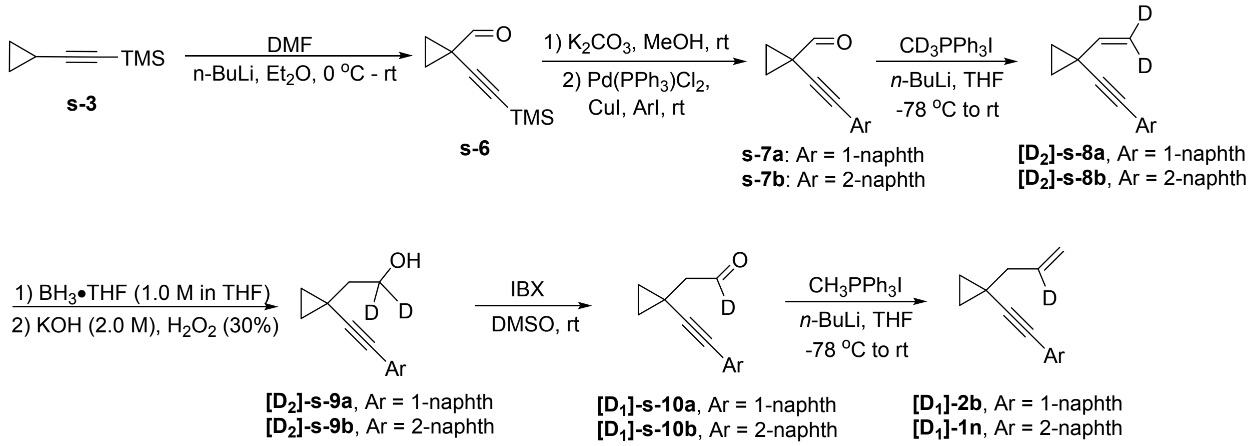

Scheme S6

The deuterium labeling compounds **[D$_1$]-2b** and **[D$_1$]-1n** was synthesized according to Scheme S6. The synthetic route was very similar as that of compound **[D$_2$]-2b**.

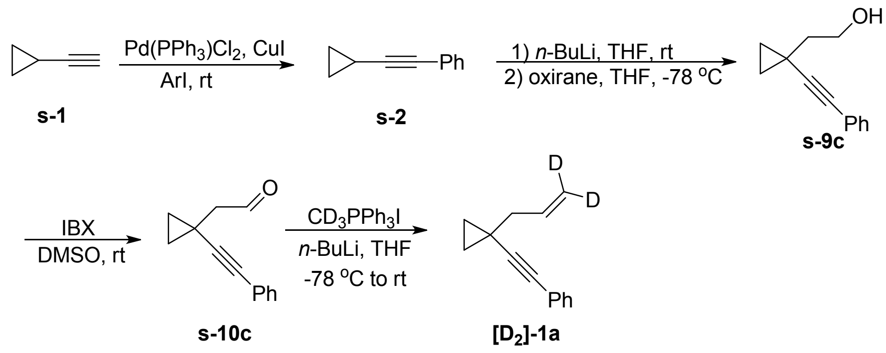

Scheme S7

The deuterium labeling compound **[D$_2$]-1a** was synthesized according to Scheme S7.

Synthesis of compound **s-9c**: To a 100 mL flame and vacuum dried flask was added compound **s-2** (7.11 g, 50 mmol), then the flask was evacuated and backfilled with Ar. THF (40 mL) was added and then *n*-BuLi (2.5 M, 20.0 mL, 50 mmol) was added slowly at -78 °C. The reaction mixture was allowed to stir at rt for 1.0 hour, then cooled to -78 °C. Epoxyethane dissolved in THF was added slowly until the color of reaction mixture disappeared. After 20 min., the reaction was quenched with saturated NH_4Cl solution. The product was purified by silica gel column chromatography (eluent: PE/EA = 10/1 to 4/1). The product was afforded as a colorless oil (7.018 g, yield = 75%).

Synthesis of compound **s-10c**: To a 100 mL flask was added compound **s-9c** (3.969 g, 21.3 mmol), then 7.16 g IBX was added. The reaction mixture was allowed to stir at rt overnight. The product was purified by silica gel column chromatography (eluent: PE/EA = 30/1). The product was afforded as a colorless oil (3.625 g, yield = 92%).

Synthesis of compound **[D$_2$]-1a**: To a 50 mL flame and vacuum dried flask was added CD_3PPh_3I (4.5 g, 11 mmol), then the flask was evacuated and backfilled with Ar. THF (20 mL) was added, and then *n*-BuLi (2.5 M, 4.4.0 mL, 11 mmol) was added slowly at -78 °C. After 20 min., **s-10c** (1.84 g, 10 mmol) dissolved in 10 mL dry THF was added to the flask. The reaction mixture was allowed to warm to rt gradually and stirred at rt for 6 hours. The product was purified by silica gel column chromatography using PE as an eluent. The product was afforded as a colorless oil (1.5 g, yield = 82%).

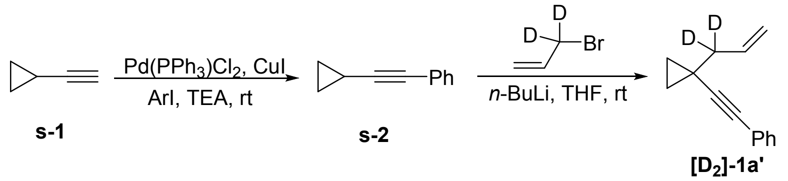

Scheme S8

The deuterium labeling compound **[D_2]-1a’** was synthesized according to Scheme S8. The deuterated allyl bromide was prepared according to the previously reported procedure.[4]

Synthesis of compound **[D_2]-1a’**: To a 100 L flame and vacuum dried flask was added compound **s-2** (1.42 g, 10 mmol) and 20 mL THF under Ar, then *n*-BuLi (2.5M, 4.5 mL, 11.25 mmol) was added slowly at rt. The deuterated allyl bromide (Et_2O solution) was added slowly until the brown color of the reaction mixture disappeared. Compound **[D_2]-1a’** and the compound **s-2** can not be separated by silica gel column chromatography. The product was purified by GPC (gel permeation chromatography). The product was afforded as a colorless oil (0.105 g, yield = 6%).

4. Optimization of the Reaction Conditions.

Table SI-1. Optimization of the reaction conditions for the gold catalyzed cycloisomerization of **1a**.

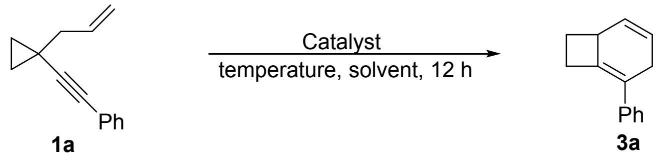

entry	catalyst	solvent	temperature (^oC)	yield (%)
1	$PPh_3AuCl/AgSbF_6$ (5 mol%)	DCM	rt	82[a]
2[b]	$PPh_3AuCl/AgSbF_6$ (5 mol%)	DCE	rt	27[a]
3[b]	$PPh_3AuCl/AgSbF_6$ (5 mol%)	CH_3CN	rt	N.R.
4	[JohnPhosAu(MeCN)]SbF_6 (3 mol%)	DCE	rt	81[a]
5	[JohnPhosAu(MeCN)]SbF_6 (3 mol%)	DCM	-20	35[c,e]
6[d]	***[JohnPhosAu(MeCN)]SbF_6 (3 mol%)***	***DCM***	***0***	***92[e]***
7	[JohnPhosAu(MeCN)]SbF_6 (3 mol%)	DCE	60	61[a]
8	[P(*p*-F-C_6H_4)$_3$Au(MeCN)]SbF_6 (3 mol%)	DCM	0	72[a]
9	[P(*p*-CF_3-C_6H_4)$_3$Au(MeCN)]SbF_6 (3 mol%)	DCM	0	73[a]
10	[P(OAr)$_3$Au(MeCN)]SbF_6 (3 mol%)	DCM	0	N.R.
11	[P(*t*-Bu)$_3$Au(MeCN)]SbF_6 (3 mol%)	DCM	0	36[a]
12	[JackiePhosAu(MeCN)]SbF_6 (3 mol%)	DCM	0	N.R.
13	[XPhosAu(MeCN)]SbF_6 (3 mol%)	DCM	0	22[a]
14	[IPrAu(MeCN)]SbF_6 (3 mol%)	DCM	0	21[a]

[a] NMR yield. The product contains unidentified byproduct. [b] The reaction was conducted under O_2 atmosphere. [c] Compound **1a** can be recovered quantitatively. [d] The solvent was degassed with Ar. [e] Isolated yield. Ar = 2,4-di-*t*-Bu-Ph.

We screened various reaction conditions to find out the optimal reaction conditions for the gold catalyzed cycloisomerization of compound **1a**. We found that the product **3a** can be obtained in 82% yield using $PPh_3AuCl/AgSbF_6$ as catalyst (Table SI-1, entry 1). The yield of **3a** dropped to 27% when the reaction was conducted under O_2 atmosphere (Table SI-1, entry 2). No reaction occurred when the reaction was conducted in acetonitrile (Table SI-1, entry 3). The product can be obtained in 81% yield using [JohnPhosAu·MeCN]SbF_6 as a catalyst (Table SI-1, entry 4). When the temperature was lowered to -20 ^oC, the desired product was obtained in 35% yield and the compound **1a** can be recovered quantitatively (Table SI-1, entry 5). When the reaction was conducted at 0 ^oC and the solvent was degassed with Ar, the desired product can be obtained in 92% yield, and the unidentified byproduct could not be detected (Table SI-1, entry 6). When the reaction was conducted at 60 ^oC in DCE, the yield dropped to 61% (Table SI-1, entry 7). Other gold catalysts such as [(*p*-F-Ph)$_3$PAu·MeCN]SbF_6, [(*p*-CF_3-Ph)$_3$PAu·MeCN]SbF_6, [P(OAr)$_3$Au·MeCN]SbF_6, [(*t*-Bu)$_3$PAu·MeCN]SbF_6, [JackiePhosAu·MeCN]SbF_6, [XPhosAu·MeCN]SbF_6, and [IPrAu·MeCN]SbF_6 can only gave inferior results (Table SI-1, entries 8-14).

The primary ligand effect was not obvious. Electron-deficient $P(OAr)_3$ ligand was not effective for our reaction. PPh_3, P(*p*-F-Ph)$_3$, and P(*p*-CF_3-Ph)$_3$ gave similar result, indicating that electronic effect in the ligand was not obvious. Poor yield was obtained with electron-rich P(*t*-Bu)$_3$ or IPr ligand. The JohnPhos ligand was found to be the best ligand, and other Buchwald type ligands such as XPhos or JackiePhos gave poor result.

5. Spectroscopic Data.

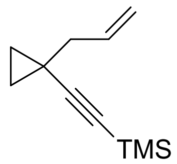

((1-Allylcyclopropyl)ethynyl)trimethylsilane s-4a:

17.2 g, yield = 63%. Colorless oil. ^{1}H NMR (400 MHz, $CDCl_3$, TMS): δ 0.12 (s, 9H, CH_3), 0.63 (dd, J_1 = 6.4 Hz, J_2 = 4.4 Hz, 2H, CH_2), 0.91 (dd, J_1 = 6.8 Hz, J_2 = 4.4 Hz, 2H, CH_2), 2.12 (d, J = 7.2 Hz, 2H, CH_2), 5.05-5.10 (m, 2H, =CH_2), 5.85-5.96 (m, 1H, =CH). ^{13}C NMR (100 MHz, $CDCl_3$, TMS): δ 0.24, 12.2, 15.0, 41.8, 80.6, 111.7, 116.5, 135.3. IR (CH_2Cl_2) ν 3081, 2960, 2159, 1249, 912, 837, 758, 697 cm^{-1}. MS (%) m/z 178 (M^+, 0.51), 163 (47.68), 145 (5.28), 135 (26.62), 123 (43.77), 104 (20.68), 73 (100.00), 59 (56.78), 43 (19.16). HRMS (EI) calcd. for $C_{11}H_{18}Si$: 178.1178, found: 178.1174.

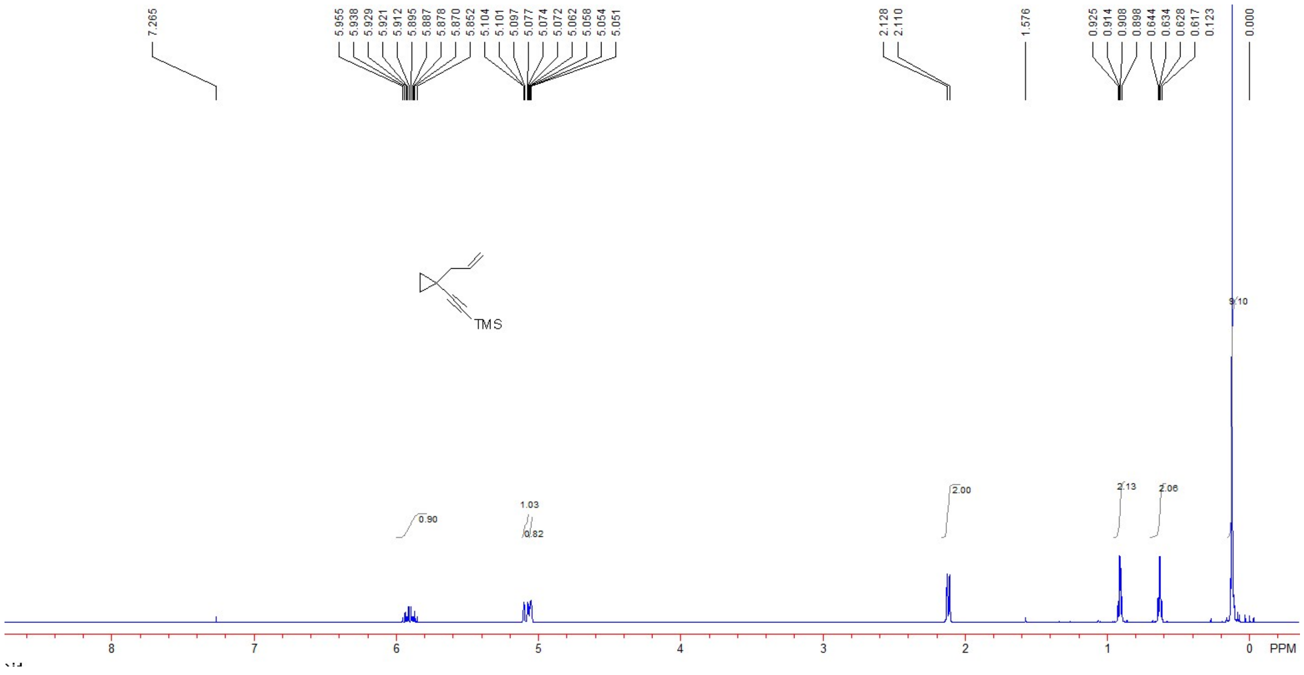

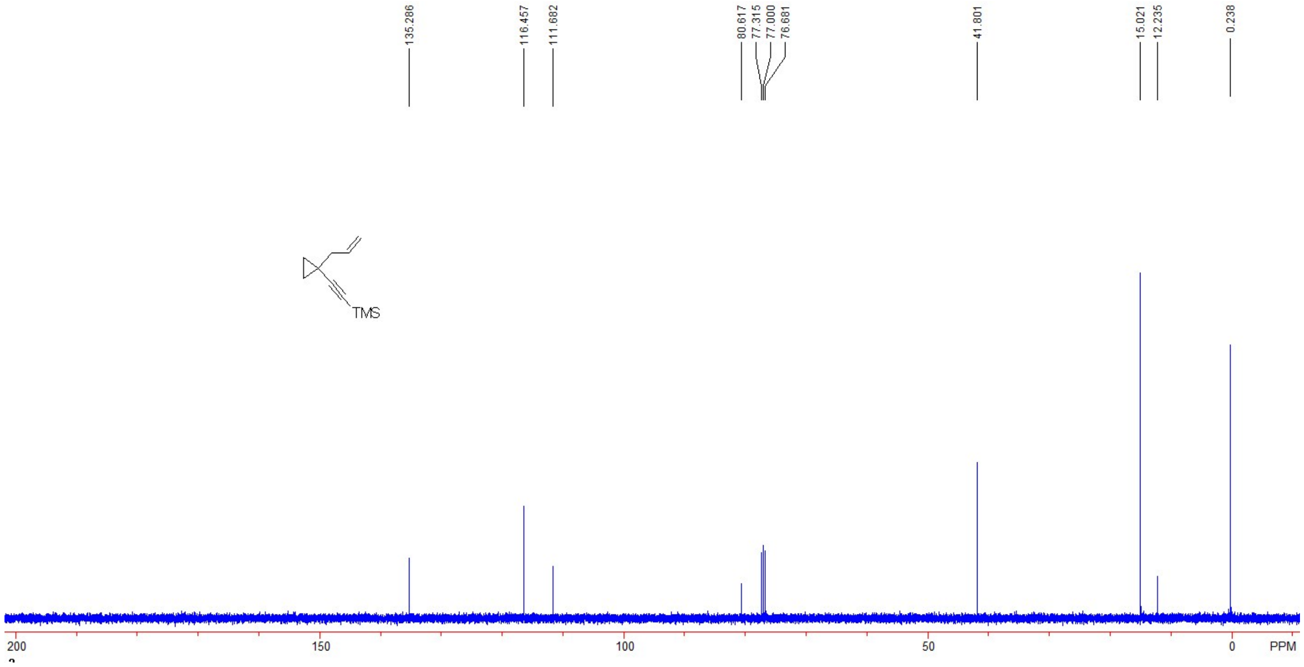

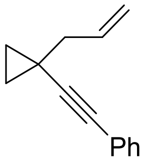

((1-allylcyclopropyl)ethynyl)benzene 1a:

This is a known compound.[5] Colorless oil. ^{1}H NMR (400 MHz, $CDCl_3$, TMS): δ 0.73 (dd, J_1 = 6.4 Hz, J_2 = 4.0 Hz, 2H, CH_2), 1.01 (dd, J_1 = 6.4 Hz, J_2 = 4.0 Hz, 2H, CH_2), 2.21 (d, J = 6.8 Hz, 2H, CH_2), 5.09-5.17 (m, 2H, =CH_2), 5.93-6.03 (m, 1H, CH_2), 7.24-7.28 (m, 3H, Ar), 7.35-7.38 (m, 2H, Ar). ^{13}C NMR (100 MHz, $CDCl_3$, TMS): δ 12.0, 15.0, 42.0, 77.0, 94.7, 116.5, 123.9, 127.4, 128.1, 131.6, 135.5.

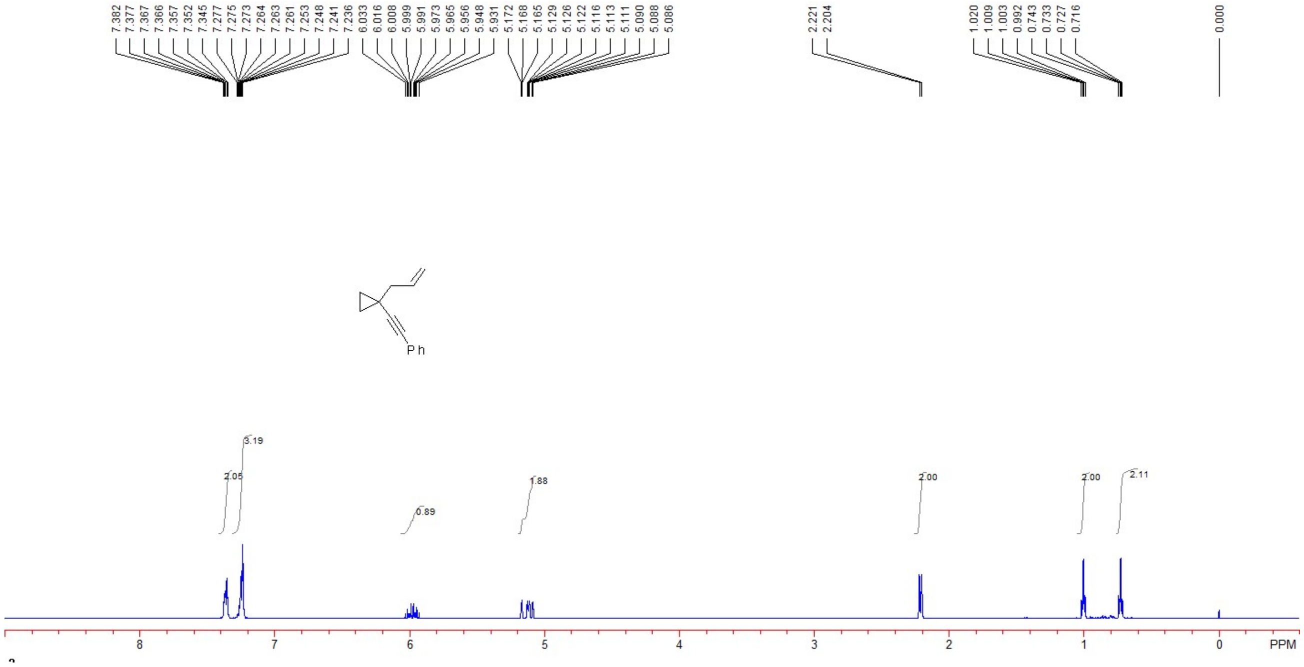

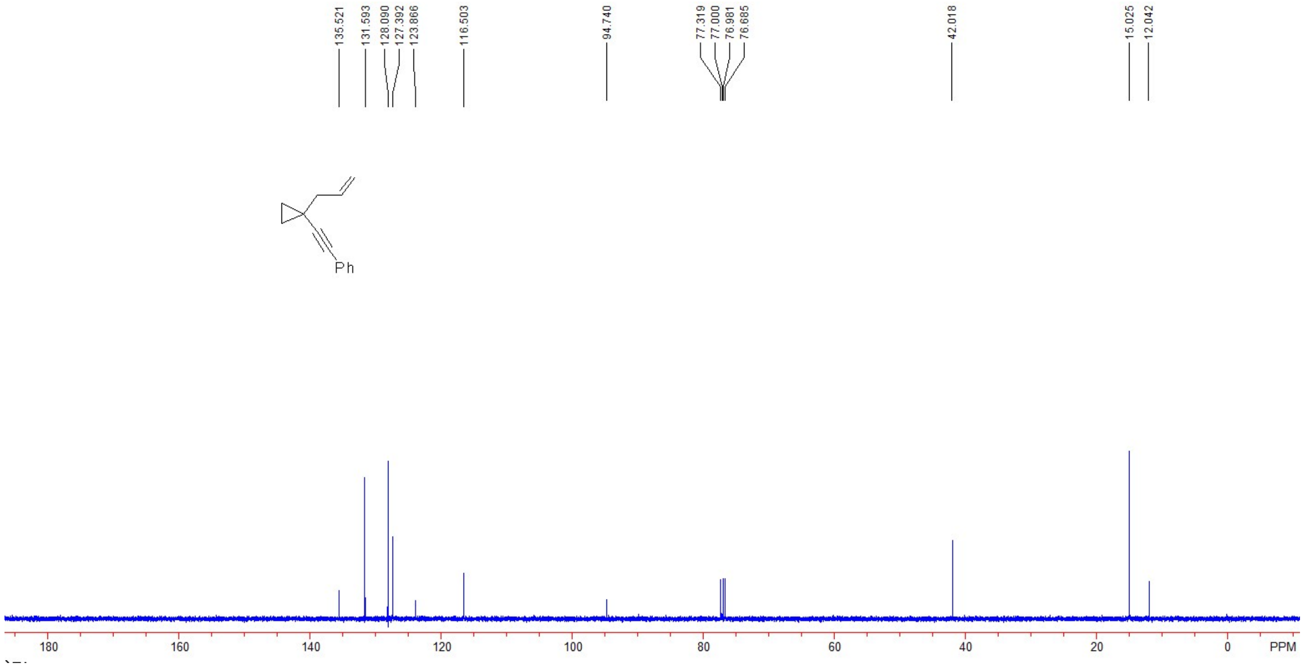

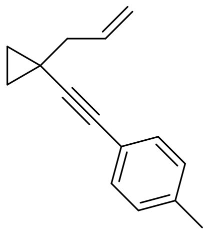

1-((1-Allylcyclopropyl)ethynyl)-4-methylbenzene 1b:

0.350 g, yield = 89%. Colorless oil. ^{1}H NMR (400 MHz, $CDCl_3$, TMS): δ 0.71 (dd, J_1 = 6.8 Hz, J_2 = 4.4 Hz, 2H, CH_2), 0.99 (dd, J_1 = 6.8 Hz, J_2 = 4.4 Hz, 2H, CH_2), 2.20 (d, J = 6.8 Hz, 2H, CH_2), 2.31 (s, 3H, CH_3), 5.09 (dd, J_1 = 10.4 Hz, J_2 = 1.6 Hz, 1H, =CH_2), 5.14 (dd, J_1 = 17.2 Hz, J_2 = 1.6 Hz, 1H,

=CH_2), 5.93-6.03 (m, 1H, =CH), 7.05 (d, *J* = 8.0 Hz, 2H, Ar), 7.26 (d, *J* = 8.0 Hz, 2H, Ar). ^{13}C NMR (100 MHz, $CDCl_3$, TMS): δ 12.0, 15.0, 21.4, 42.1, 93.9, 116.4, 120.8, 128.8, 131.5, 135.6, 137.3. IR (CH_2Cl_2) ν 3088, 3006, 2921, 1610, 1423, 996, 914, 815 cm^{-1}. MS (%) m/z 196 (M^+, 19.52), 181 (100.00), 155 (98.40), 128 (63.35), 115 (84.27), 105 (44.33), 89 (29.70), 77 (27.34), 63 (17.97). HRMS (EI) calcd. for $C_{15}H_{16}$: 196.1252, found: 196.1256.

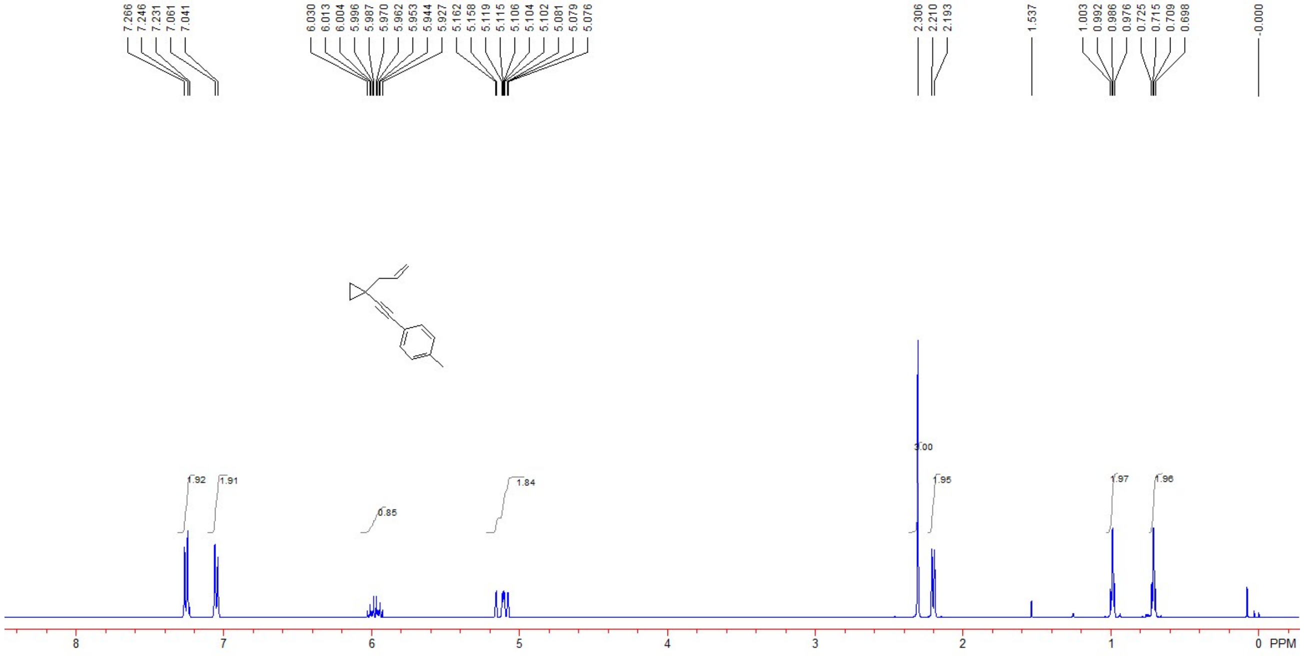

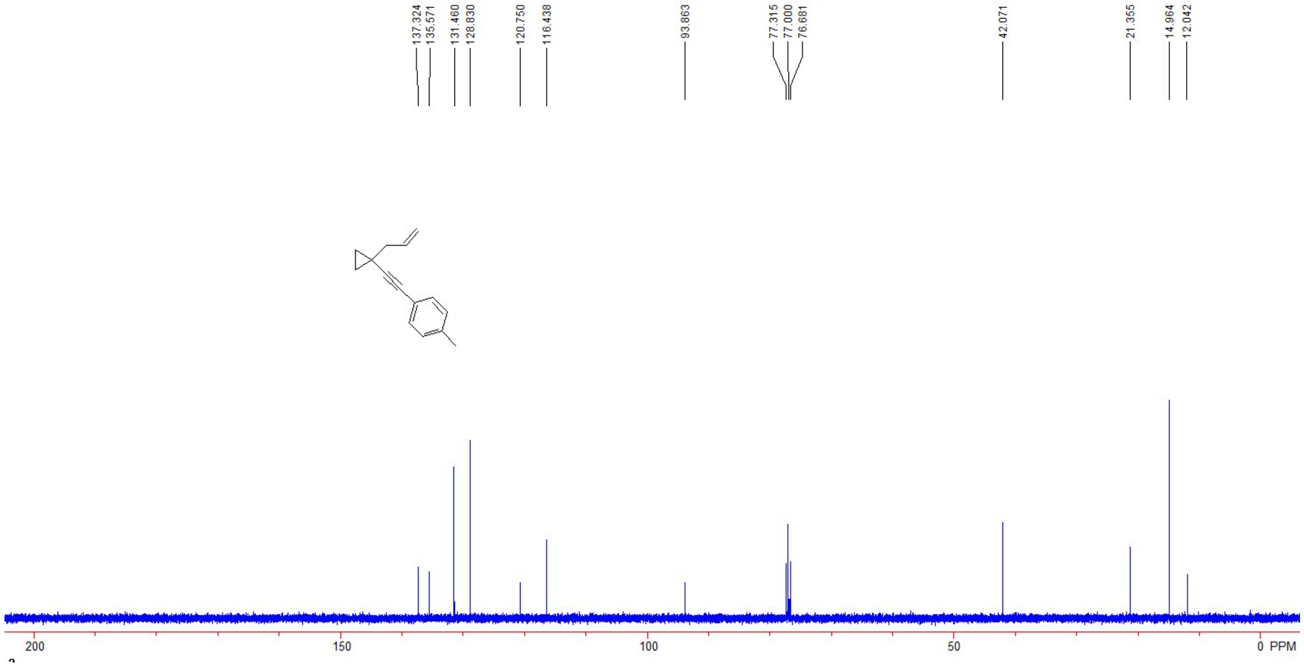

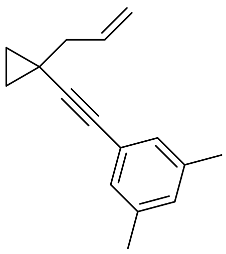

1-((1-Allylcyclopropyl)ethynyl)-3,5-dimethylbenzene 1c:

0.703 g, yield = 96%. Colorless oil. ^{1}H NMR (400 MHz, $CDCl_3$, TMS): δ 0.70 (dd, J_1 = 6.8 Hz, J_2 = 4.4 Hz, 2H, CH_2), 0.98 (dd, J_1 = 6.8 Hz, J_2 = 4.4 Hz, 2H, CH_2), 2.19 (d, J = 6.8 Hz, 2H, CH_2), 2.23 (s, 6H, CH_3), 5.09 (d, J = 10.4 Hz, 1H, =CH_2), 5.14 (d, J = 17.2 Hz, 1H, =CH_2), 5.92-6.02 (m, 1H, =CH), 6.85 (s, 1H, Ar), 6.99 (s, 2H, Ar). ^{13}C NMR (100 MHz, $CDCl_3$, TMS): δ 12.0, 15.0, 21.0, 42.0, 76.7, 93.9, 116.4, 123.4, 129.3, 135.5, 137.5. IR (CH_2Cl_2) ν 3078, 3006, 2918, 1597, 1423, 913, 996, 689 cm^{-1}. MS (%) m/z 210 (M^+, 83.76), 195 (100.00), 165 (73.18), 153 (55.25), 128 (40.44), 115 (38.91), 89 (28.54), 77 (28.47), 63 (13.40). HRMS (EI) calcd. for $C_{16}H_{18}$: 210.1409, found: 210.1411.

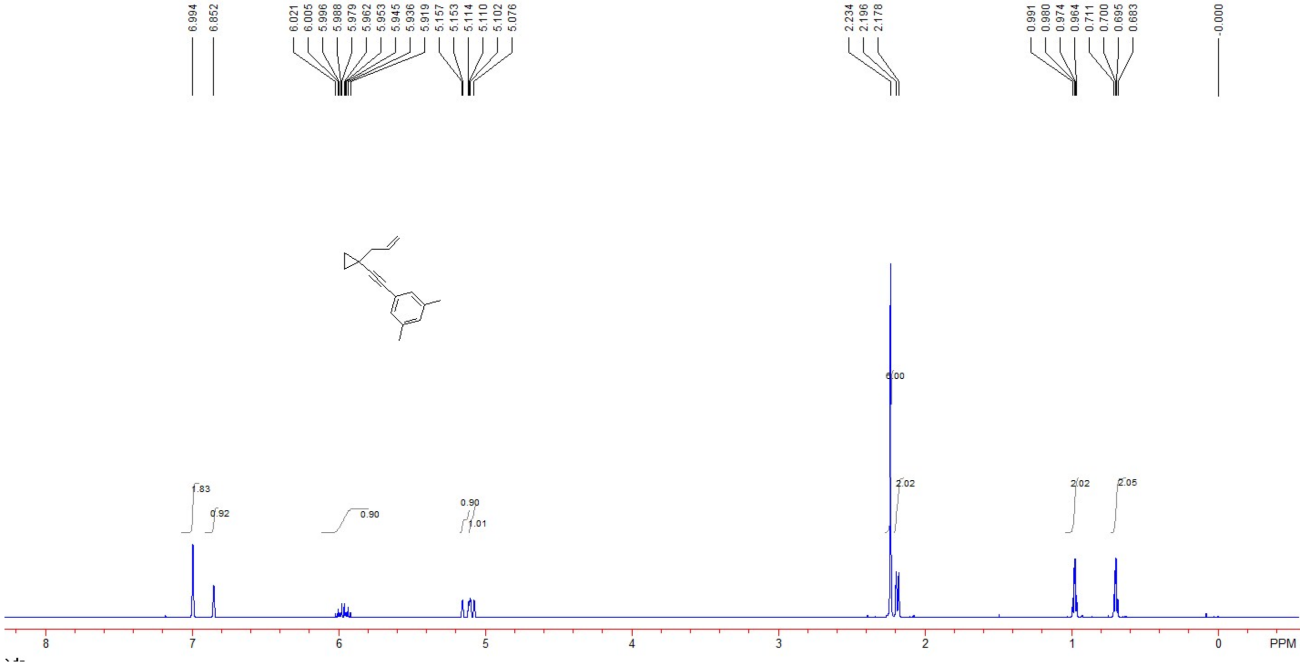

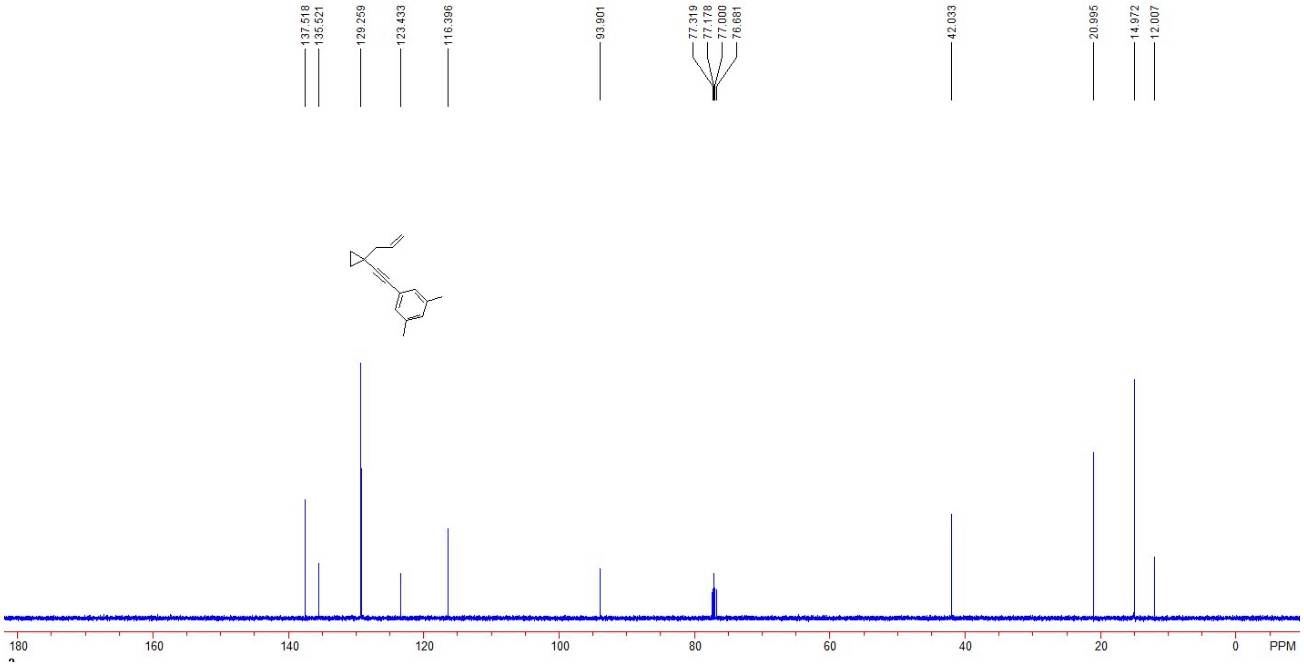

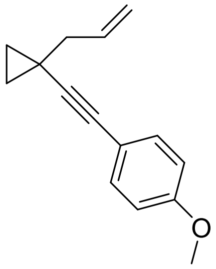

1-((1-Allylcyclopropyl)ethynyl)-4-methoxybenzene 1d:

0.585 g, yield = 92%. Colorless oil. ^{1}H NMR (400 MHz, $CDCl_3$, TMS): δ 0.70 (dd, J_1 = 6.4 Hz, J_2 = 4.4 Hz, 2H, CH_2), 0.98 (dd, J_1 = 6.4 Hz, J_2 = 4.4 Hz, 2H, CH_2), 2.20 (d, J = 6.8 Hz, 2H, CH_2), 5.09 (d, J = 10.4 Hz, 1H, =CH_2), 5.14 (d, J = 17.2 Hz, 1H, =CH_2), 5.93-6.03 (m, 1H, =CH), 6.77 (d, J = 8.8 Hz, 2H, Ar), 7.30 (d, J = 8.8 Hz, 2H, Ar). ^{13}C NMR (100 MHz, $CDCl_3$, TMS): δ 12.0, 14.9, 42.1, 55.1, 76.7, 93.0, 113.7, 116.0, 116.4, 132.9, 135.6, 158.9. IR (CH_2Cl_2) ν 3076, 3004, 2836, 1606, 1508, 1245, 1169, 1032, 830 cm^{-1}. MS (%) m/z 212 (M^+, 76.92), 197 (37.70), 184 (18.08), 171 (89.76), 141 (42.96), 128 (100.00), 115 (77.76), 102 (30.33), 89 (20.75), 71 (18.03). HRMS (EI) calcd. for $C_{15}H_{16}O$: 212.1201, found: 212.1198.

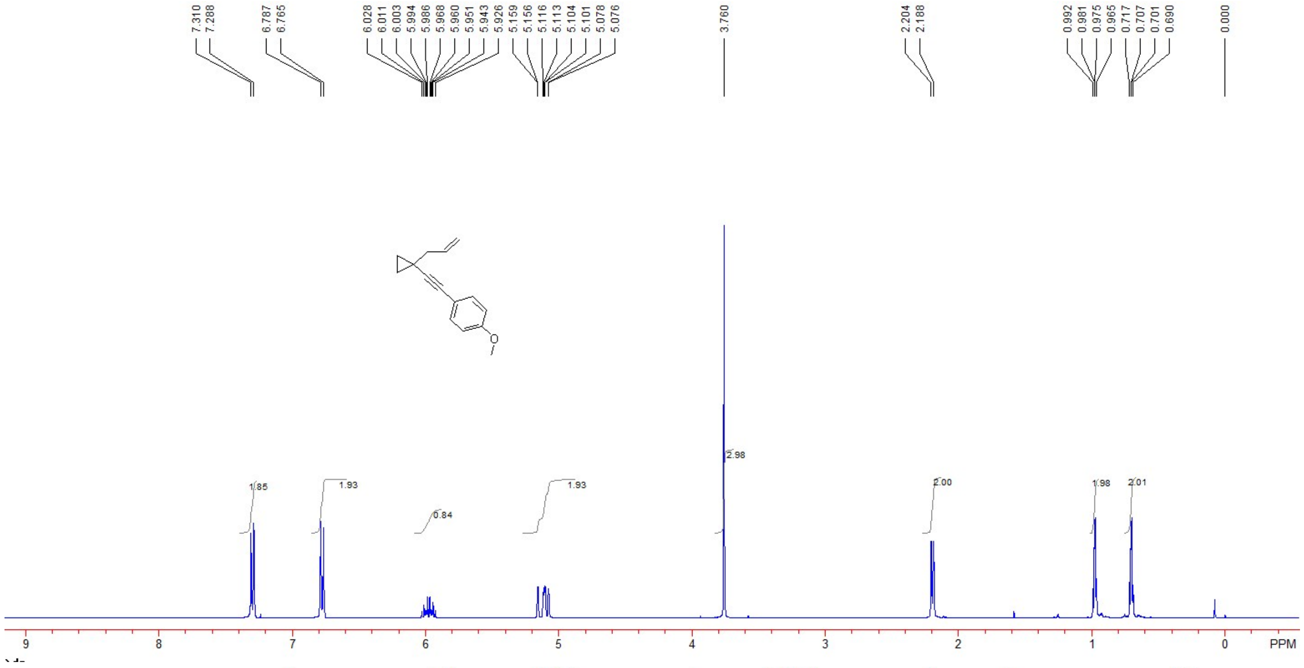

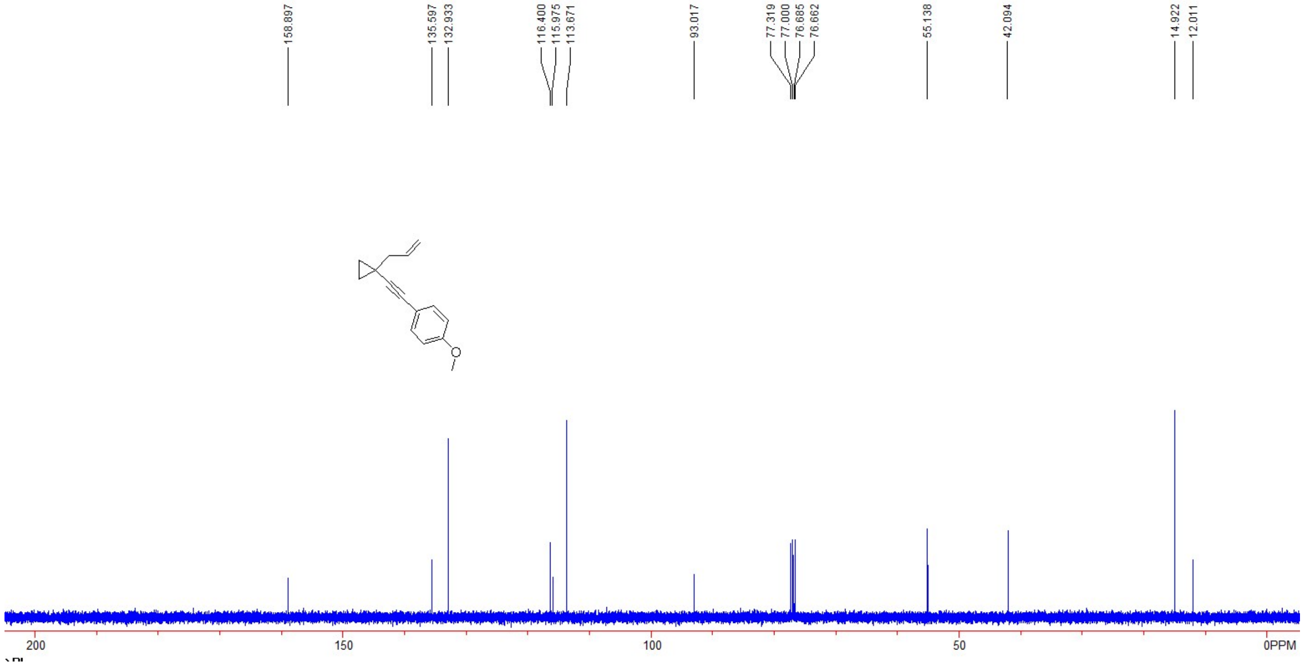

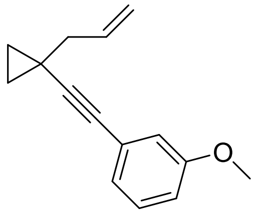

1-((1-Allylcyclopropyl)ethynyl)-3-methoxybenzene 1e:

0.783, yield = 74%. Colorless oil. ^{1}H NMR (400 MHz, $CDCl_3$, TMS): δ 0.77 (dd, J_1 = 6.4 Hz, J_2 = 4.4 Hz, 2H, CH_2), 1.00 (dd, J_1 = 6.4 Hz, J_2 = 4.4 Hz, 2H, CH_2), 2.19 (d, J = 6.8 Hz, 2H, CH_2), 3.72 (s, 3H, CH_3), 5.07-5.17 (m, 2H, =CH_2), 5.93-6.00 (m, 1H, =CH), 6.77-6.79 (m, 1H, Ar), 6.89-6.90 (m,

1H, Ar), 6.94-6.97 (m, 1H, Ar), 7.11-7.20 (m, 1H, Ar). ^{13}C NMR (100 MHz, $CDCl_3$, TMS): δ 11.9, 14.9, 41.9, 55.0, 76.9, 94.5, 113.9, 116.3, 116.4, 124.1, 124.8, 129.1, 135.4, 159.1. IR (CH_2Cl_2) ν 3076, 3004, 2907, 2834, 1596, 1481, 1247, 1042, 777 cm^{-1}. MS (%) m/z 212 (M^+, 91.60), 197 (34.68), 171 (57.53), 165 (38.08), 141 (32.19), 128 (100.00), 115 (77.17), 102 (32.79), 91 (21.77). HRMS (EI) calcd. for $C_{15}H_{16}O$: 212.1201, found: 212.1205.

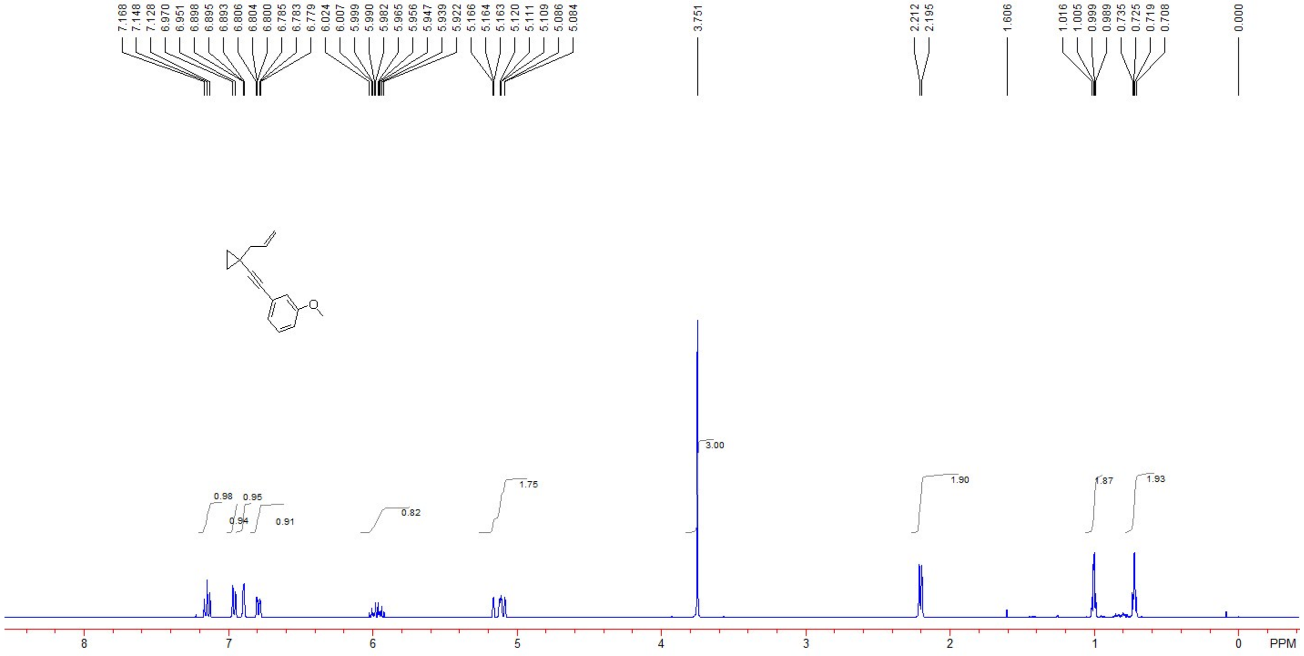

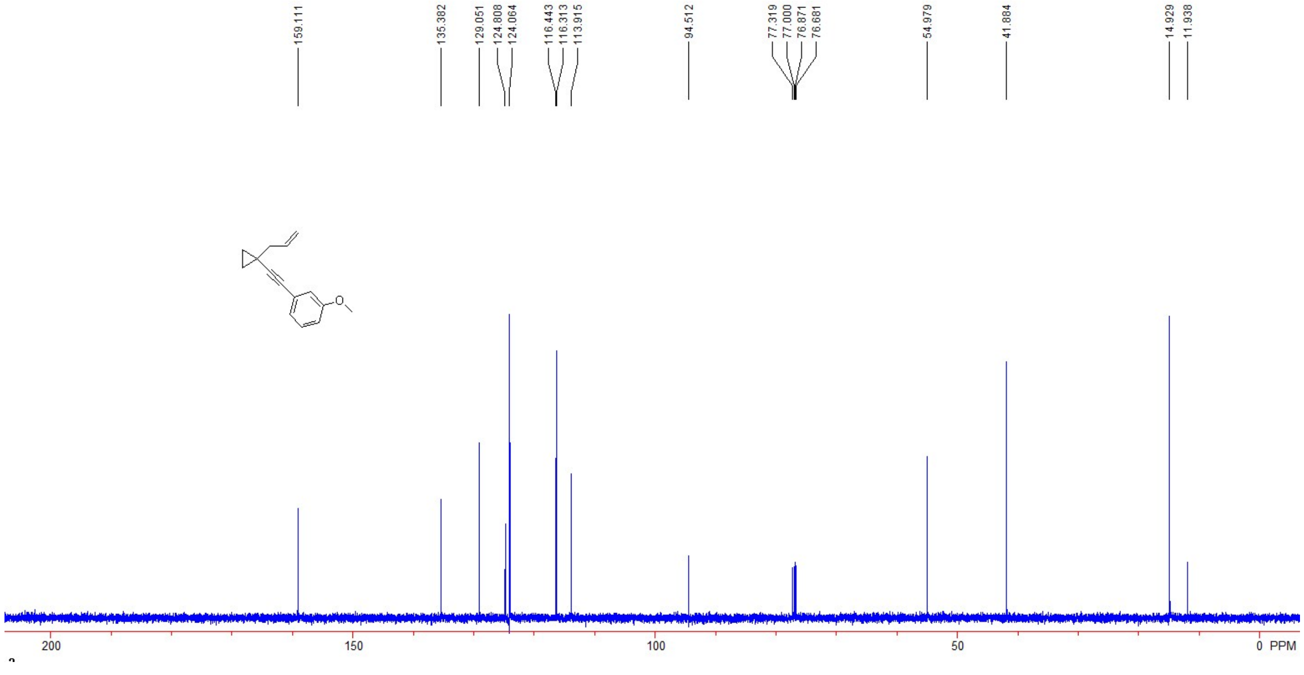

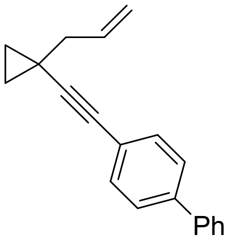

4-((1-Allylcyclopropyl)ethynyl)-1,1'-biphenyl 1f:

1.053 g, yield = 91%. Colorless oil. ^{1}H NMR (400 MHz, $CDCl_3$, TMS): δ 0.71 (dd, J_1 = 6.8 Hz, J_2 = 4.4 Hz, 2H, CH_2), 1.01 (dd, J_1 = 6.8 Hz, J_2 = 4.4 Hz, 2H, CH_2), 2.20 (d, J = 6.8 Hz, 2H, CH_2), 5.11 (d, J = 10.8 Hz, 1H, =CH_2), 5.13 (dd, J_1 = 17.2 Hz, J_2 = 1.6 Hz, 1H, =CH_2), 5.93-6.03 (m, 1H, =CH), 7.27 (dd, J_1 = 7.6 Hz, J_2 = 7.2 Hz, 1H, Ar), 7.35 (d, J = 8.0 Hz, 1H, Ar), 7.38-7.46 (m, 4H, Ar), 7.51 (d, J = 7.2 Hz, 2H, Ar). ^{13}C NMR (100 MHz, $CDCl_3$, TMS): δ 12.1, 15.0, 42.0, 76.9, 95.4, 116.5, 122.8, 126.7, 126.8, 127.3, 128.7, 132.0, 135.5, 140.0, 140.3. IR (CH_2Cl_2) ν 3077, 3030, 3005, 1486, 996, 914, 838, 761, 695 cm^{-1}. MS (%) m/z 258 (M^+, 100.00), 243 (45.51), 230 (29.14), 217 (76.57), 202 (57.86), 189 (17.97), 165 (26.95), 115 (19.84), 91 (30.55). HRMS (EI) calcd. for $C_{20}H_{18}$: 258.1409, found: 258.1411.

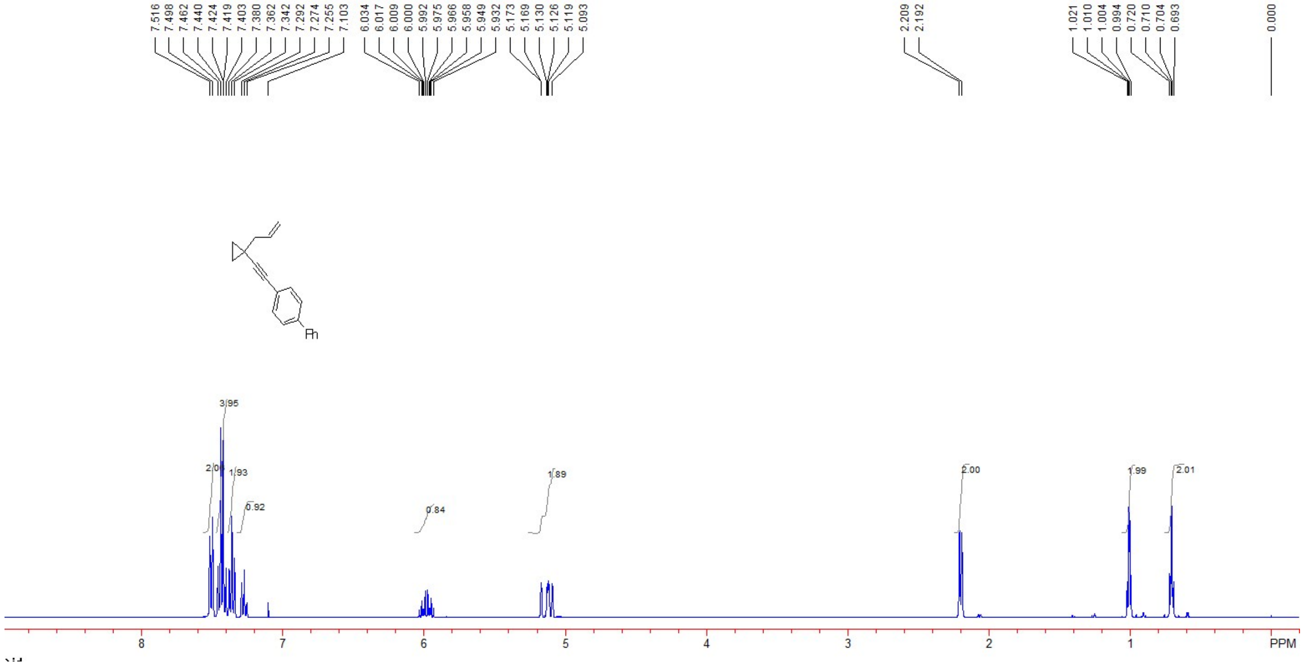

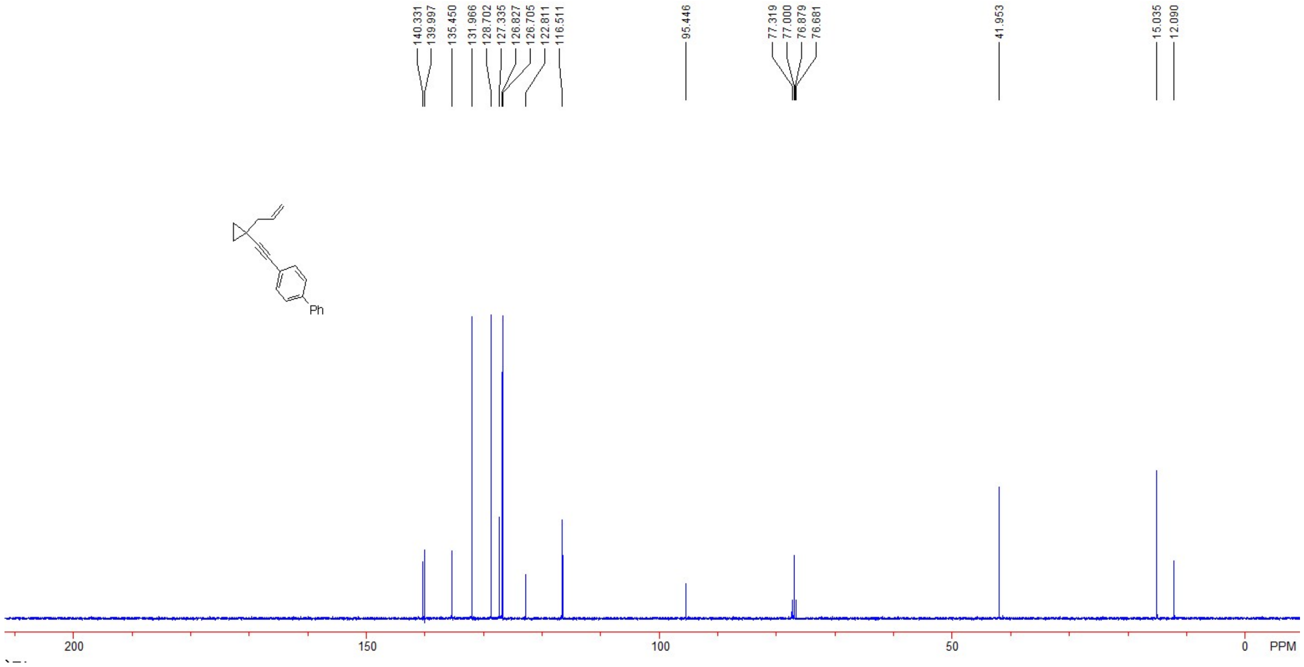

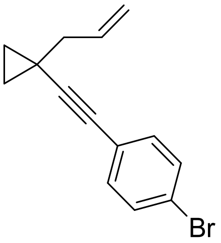

1-((1-Allylcyclopropyl)ethynyl)-4-bromobenzene 1g:

0.780 g, yield = 75%. Colorless oil. ^{1}H NMR (400 MHz, $CDCl_3$, TMS): δ 0.72 (dd, J_1 = 6.4 Hz, J_2 = 4.4 Hz, 2H, CH_2), 0.99 (dd, J_1 = 6.4 Hz, J_2 = 4.4 Hz, 2H, CH_2), 2.19 (d, J = 6.4 Hz, 2H, CH_2), 5.10 (d, J = 10.8 Hz, 1H, =CH_2), 5.13 (d, J = 18.8 Hz, 1H, =CH_2), 5.90-6.00 (m, 1H, =CH), 7.20 (d, J = 7.6 Hz, 2H, Ar), 7.36 (d, J = 7.6 Hz, 2H, Ar). ^{13}C NMR (100 MHz, $CDCl_3$, TMS): δ 12.0, 15.0, 41.8, 76.0, 96.0, 116.6, 121.4, 122.8, 131.2, 133.0, 135.3. IR (CH_2Cl_2) ν 3078, 3005, 2971, 1486, 1069, 940, 915, 821 cm^{-1}. MS (%) m/z 260 (M^+, 29.65), 219 (32.04), 181 (34.86), 166 (100.00), 139 (71.51), 114 (25.68), 99 (14.57), 89 (20.29), 76 (28.65). HRMS (EI) calcd. for $C_{14}H_{13}Br$: 260.0201, found: 260.0203.

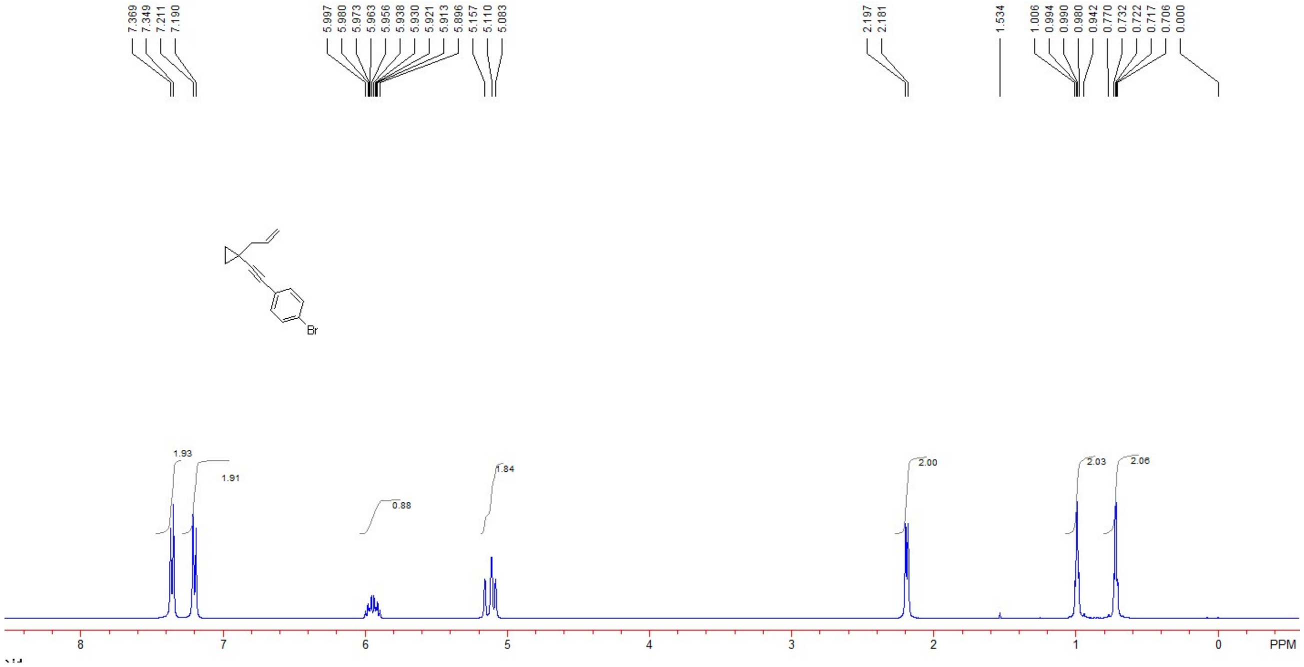

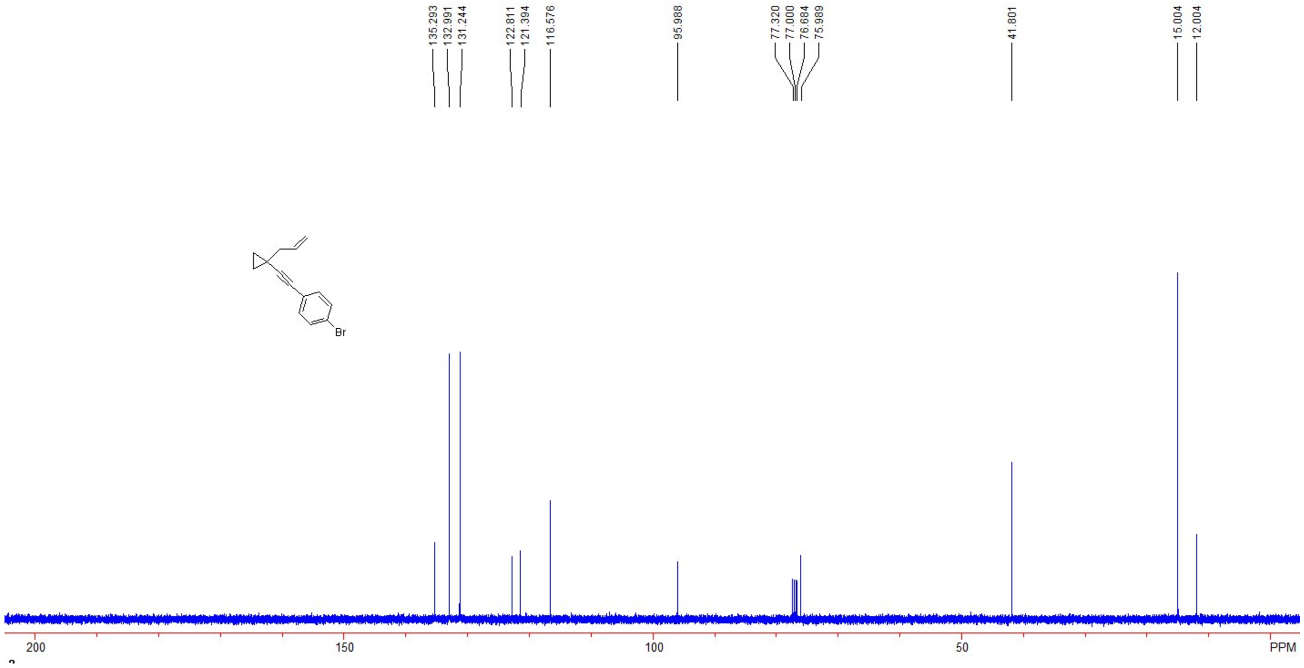

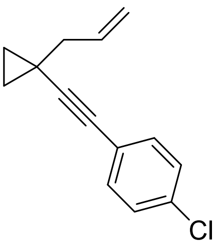

1-((1-Allylcyclopropyl)ethynyl)-4-chlorobenzene 1h:

0.737 g, yield = 97%. Colorless oil. ^{1}H NMR (400 MHz, $CDCl_3$, TMS): δ 0.70 (dd, J_1 = 6.4 Hz, J_2 = 4.4 Hz, 2H, CH_2), 0.99 (dd, J_1 = 6.4 Hz, J_2 = 4.4 Hz, 2H, CH_2), 2.18 (d, J = 6.8 Hz, 2H, CH_2), 5.09 (d, J = 11.2 Hz, 1H, =CH_2), 5.13 (d, J = 17.6 Hz, 1H, =CH_2), 5.89-5.97 (m, 1H, =CH), 7.19 (d, J =

8.4 Hz, 2H, Ar), 7.26 (d, *J* = 8.4 Hz, 2H, Ar). ^{13}C NMR (100 MHz, $CDCl_3$, TMS): δ 12.0, 15.0, 41.8, 75.9, 95.7, 116.5, 122.3, 128.3, 132.7, 133.2, 135.3. IR (CH_2Cl_2) ν 3078, 3006, 2903, 2225, 1489, 1090, 915, 825, 711 cm^{-1}. MS (%) m/z 216 (M^+, 57.15), 201 (14.16), 181 (76.26), 166 (100.00), 149 (55.02), 139 (82.24), 125 (52.89), 99 (23.57), 75 (35.70). HRMS (EI) calcd. for $C_{14}H_{13}Cl$: 216.0706, found: 216.0707.

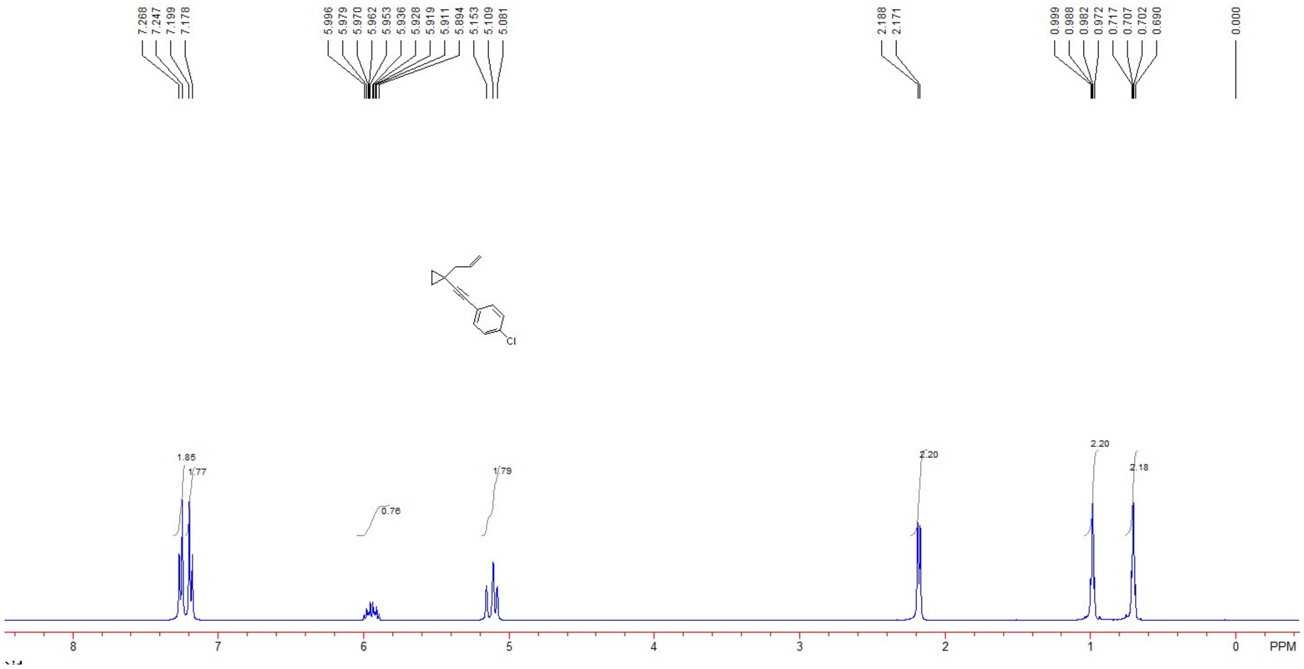

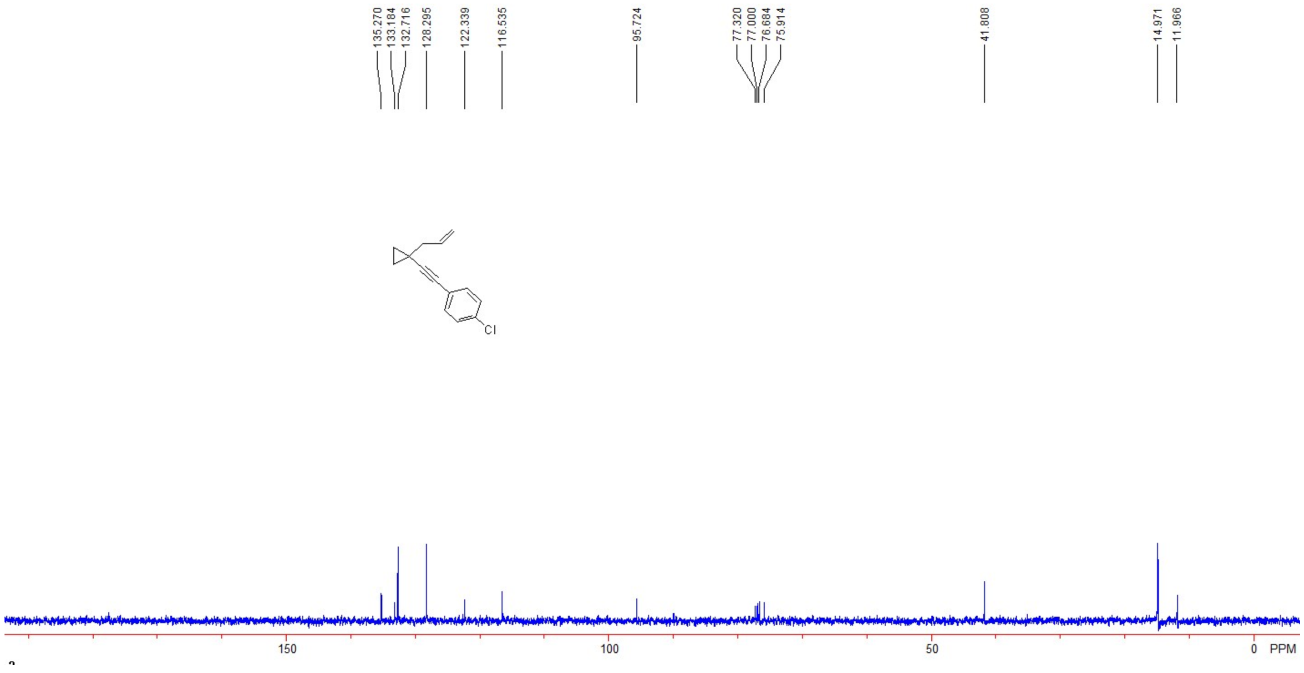

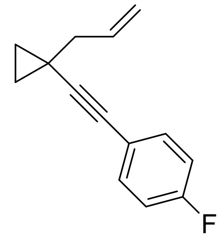

1-((1-Allylcyclopropyl)ethynyl)-4-fluorobenzene 1i:

0.599 g, yield = 86%. Colorless oil. ^{1}H NMR (400 MHz, $CDCl_3$, TMS): δ 0.71 (dd, J_1 = 6.4 Hz, J_2 = 4.4 Hz, 2H, CH_2), 0.98 (dd, J_1 = 6.4 Hz, J_2 = 4.4 Hz, 2H, CH_2), 2.19 (d, J = 6.8 Hz, 2H, CH_2), 5.10 (d, J = 11.2 Hz, 1H, $=CH_2$), 5.14 (d, J = 18.8 Hz, 1H, $=CH_2$), 5.91-6.01 (m, 1H, =CH), 6.90-6.95 (m, 2H, Ar), 7.30-7.33 (m, 2H, Ar). ^{13}C NMR (100 MHz, $CDCl_3$, TMS): δ 11.9, 14.9, 41.9, 75.9, 94.3 (d, J = 1.5 Hz), 115.2 (d, J = 21.6 Hz), 116.5, 119.9 (d, J = 3.3 Hz), 133.3 (d, J = 8.2 Hz), 135.4, 161.9, (d, J = 246.9 Hz). ^{19}F NMR (376 MHz, $CDCl_3$, $CFCl_3$): δ -112.2. IR (CH_2Cl_2) ν 3078, 3007, 2904, 1507, 1229, 916, 833, 762 cm^{-1}. MS (%) m/z 200 (M^+, 51.61), 185 (48.50), 172 (25.76), 159 (75.50), 133 (100.00), 125 (13.41), 109 (30.56), 75 (16.90), 63 (12.77). HRMS (EI) calcd. for $C_{14}H_{13}F$: 200.1001, found: 200.1003.

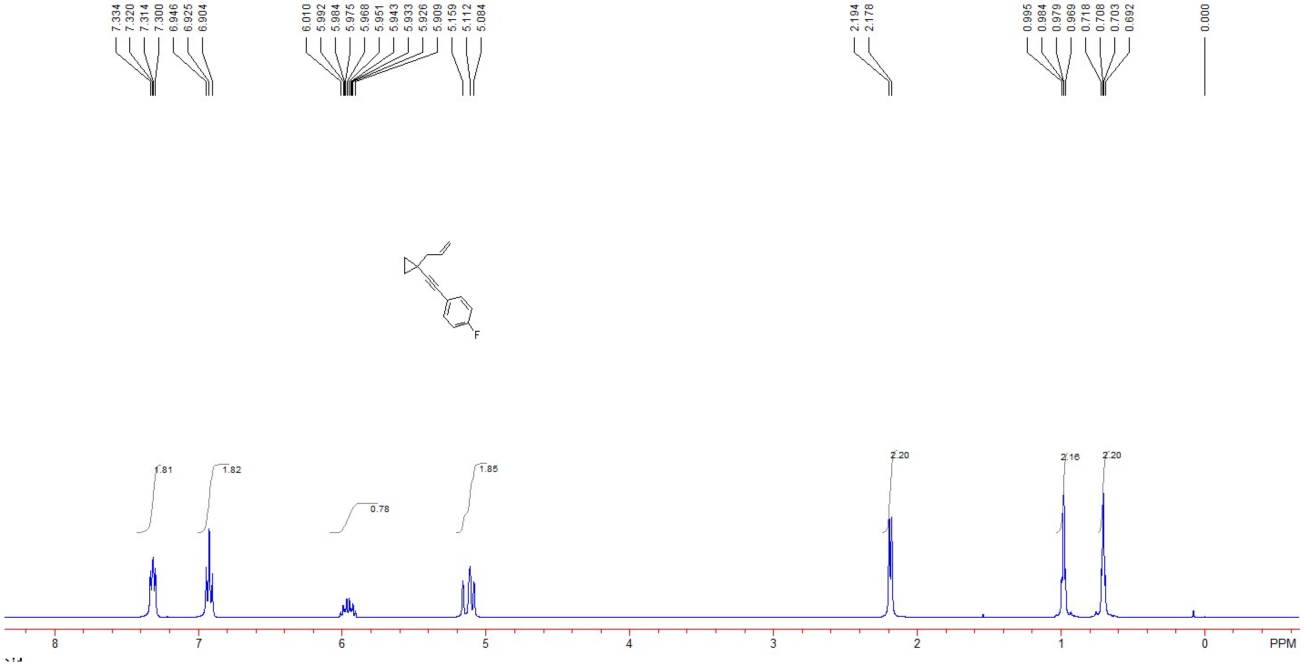

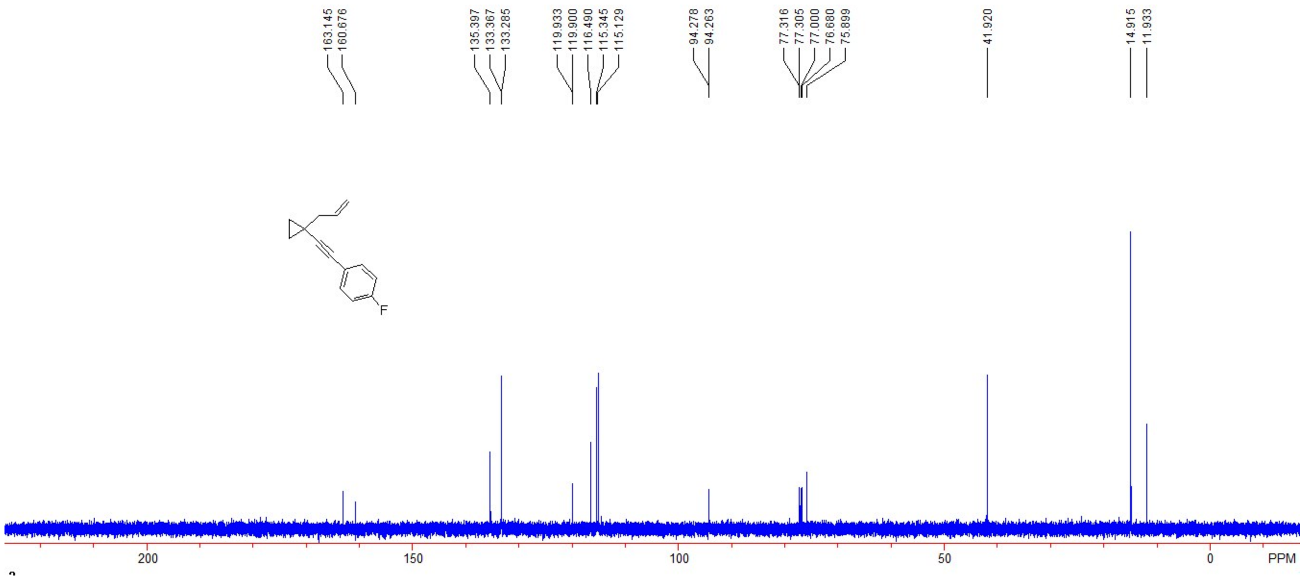

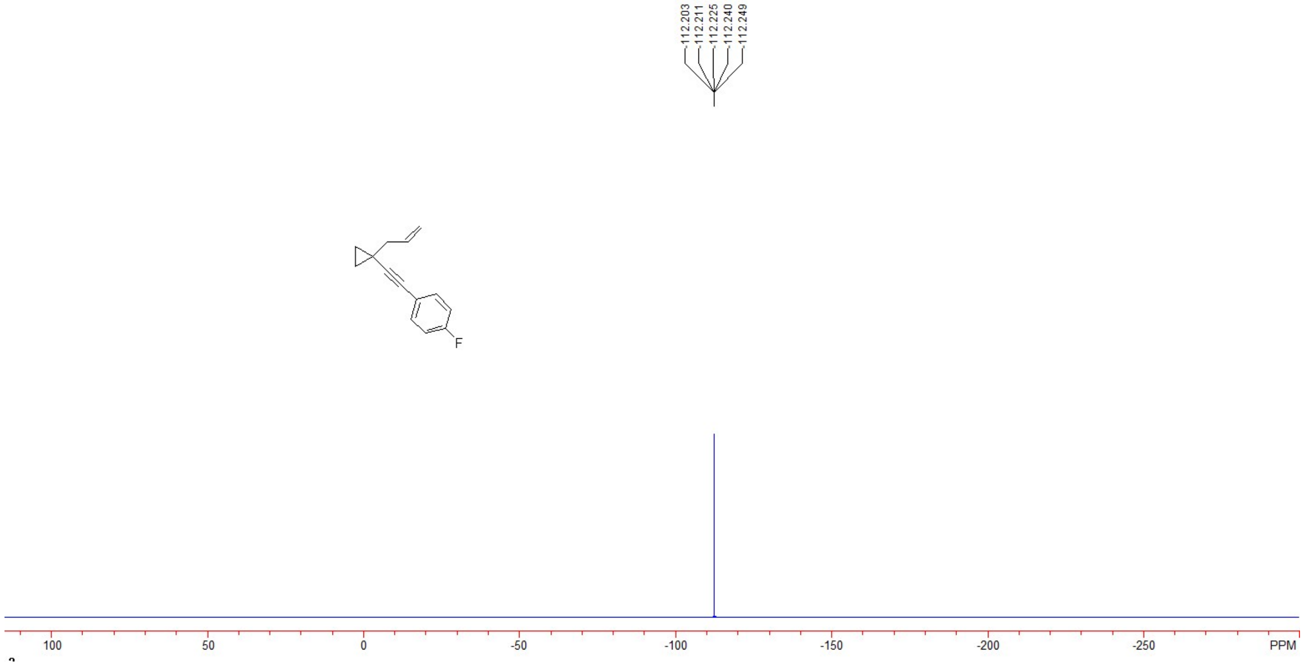

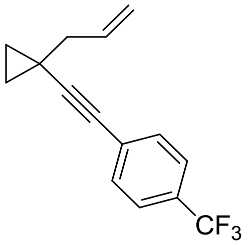

1-((1-Allylcyclopropyl)ethynyl)-4-(trifluoromethyl)benzene 1j:

1.093 g, yield = 87%. Colorless oil. ^{1}H NMR (400 MHz, $CDCl_3$, TMS): δ 0.75 (dd, J_1 = 6.8 Hz, J_2 = 4.4 Hz, 2H, CH_2), 1.02 (dd, J_1 = 6.8 Hz, J_2 = 4.4 Hz, 2H, CH_2), 2.21 (d, J = 6.8 Hz, 2H, CH_2), 5.10-5.13 (m, 1H, =CH_2), 5.16 (dd, J_1 = 15.6 Hz, J_2 =1.6 Hz, 1H, =CH_2), 5.91-6.01 (m, 1H, =CH), 7.43 (d, J = 8.4 Hz, 2H, Ar), 7.48 (d, J = 8.4 Hz, 2H, Ar). ^{13}C NMR (100 MHz, $CDCl_3$, TMS): δ 12.1, 15.1,

41.8, 76.0, 97.7, 120.0, 124.1 (q, J = 270.3 Hz), 125.1 (q, J = 3.8 Hz), 127.9 (q, J = 1.5 Hz), 129.1 (q, J = 32.6 Hz), 131.8, 135.3. ^{19}F NMR (376 MHz, $CDCl_3$, $CFCl_3$): δ -62.8. IR (CH_2Cl_2) ν 3080, 2901, 2225, 1614, 1320, 1166, 1104, 1065, 839, 687 cm^{-1}. MS (%) m/z 250 (M^+, 47.75), 235 (71.68), 222 (39.24), 209 (100.00), 181 (70.63), 165 (63.22), 152 (30.33), 133 (23.78), 91 (25.61), 78 (28.10). HRMS (EI) calcd. for $C_{15}H_{13}F_3$: 250.0969, found: 250.0972.

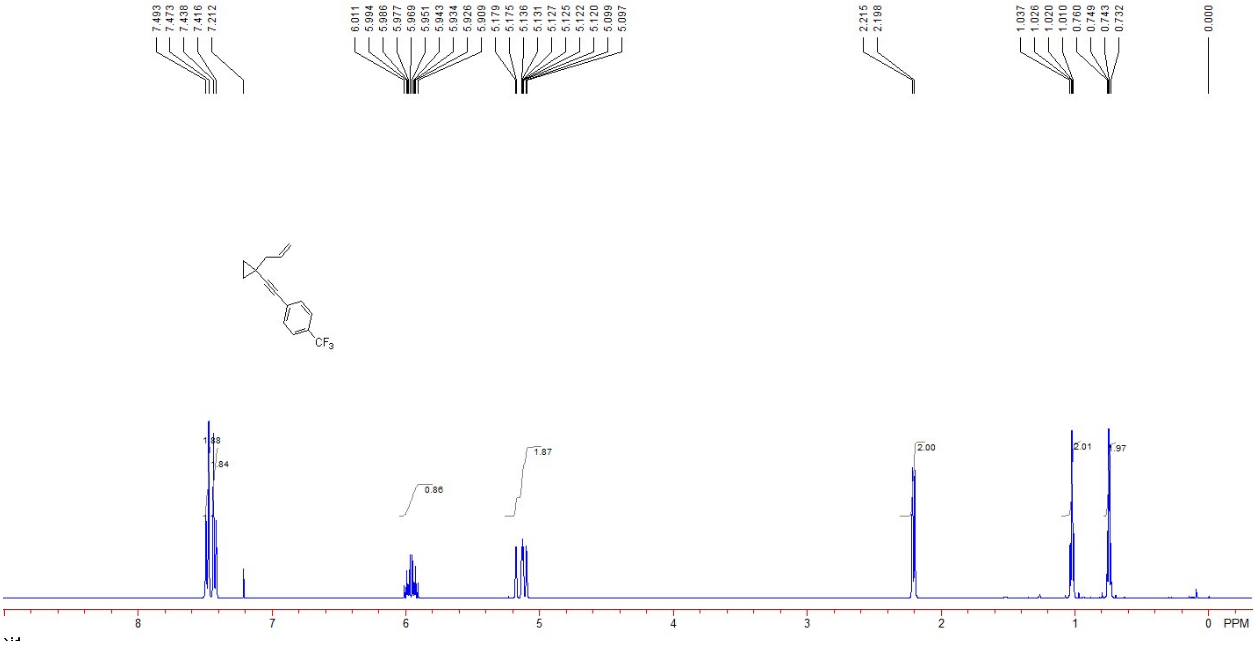

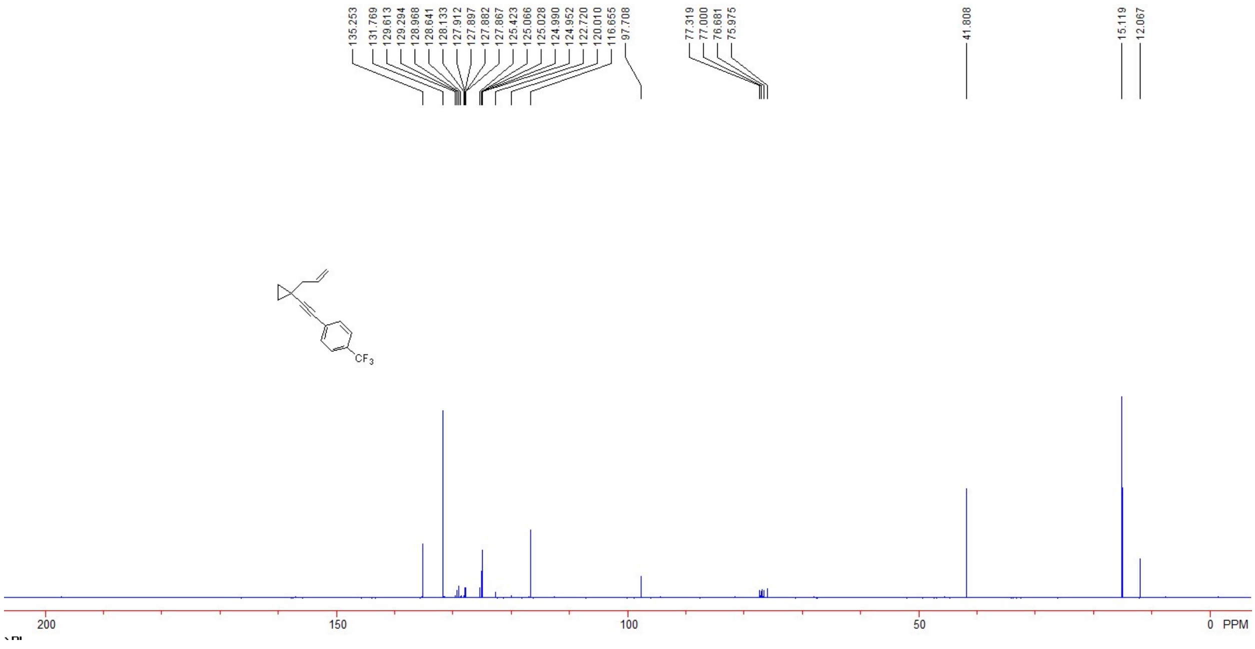

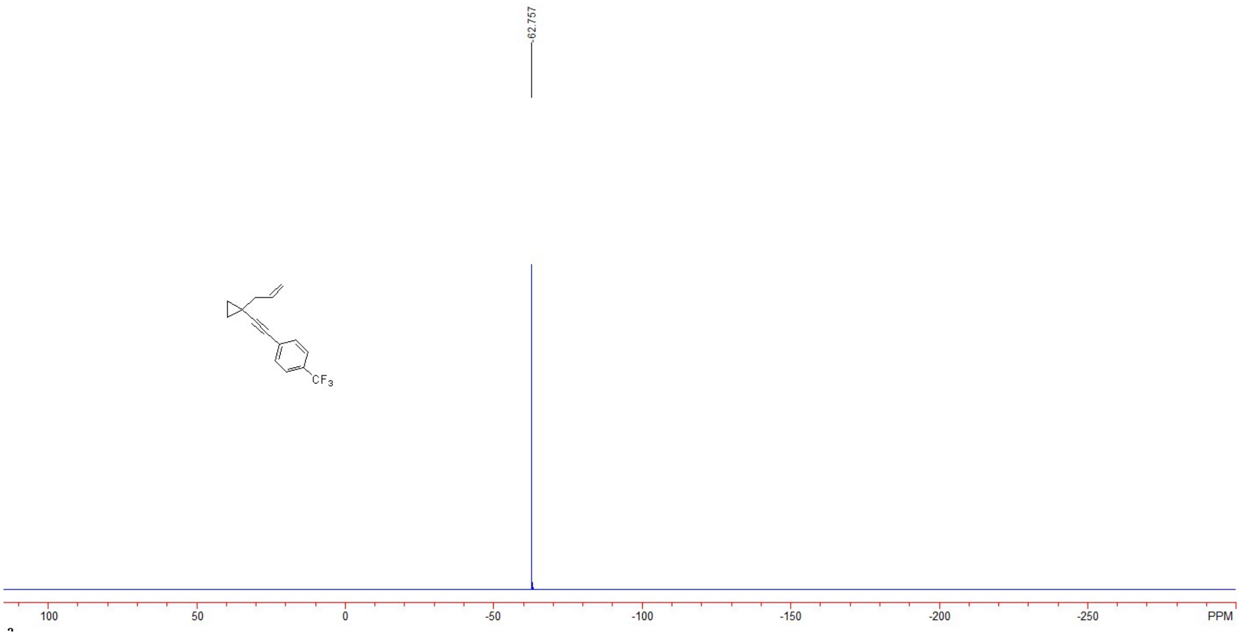

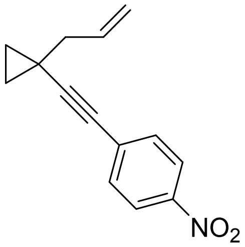

1-((1-Allylcyclopropyl)ethynyl)-4-nitrobenzene 1k:

0.905 g, yield = 99%. Brown oil. ^{1}H NMR (400 MHz, $CDCl_3$, TMS): δ 0.81 (dd, J_1 = 6.8 Hz, J_2 = 4.4 Hz, 2H, CH_2), 1.06 (dd, J_1 = 6.8 Hz, J_2 = 4.4 Hz, 2H, CH_2), 2.23 (d, J = 6.8 Hz, 2H, CH_2), 5.12 (dt, J_1 = 10.4 Hz, J_2 = 0.8 Hz, 1H, $=CH_2$), 5.17 (dd, J_1 = 17.6 Hz, J_2 = 1.2 Hz, 1H, $=CH_2$), 5.90-6.00 (m, 1H, =CH), 7.45 (d, J = 8.8 Hz, 2H, Ar), 8.10 (d, J = 8.8 Hz, 2H, Ar). ^{13}C NMR (100 MHz, $CDCl_3$, TMS): δ 12.0, 15.2, 41.3, 75.6, 101.1, 116.6, 123.1, 130.8, 131.9, 134.9, 146.1. IR (CH_2Cl_2) ν 3078, 3007, 2903, 2220, 1593, 1514, 1338, 850, 749 cm^{-1}. MS (%) m/e 227 (M^+, 29.87), 212 (13.69), 186 (42.62), 180 (46.12), 165 (100.00), 115 (31.26), 91 (27.05), 78 (29.29). HRMS (EI) calcd. for $C_{14}H_{13}NO_2$: 227.0946, found: 227.0942.

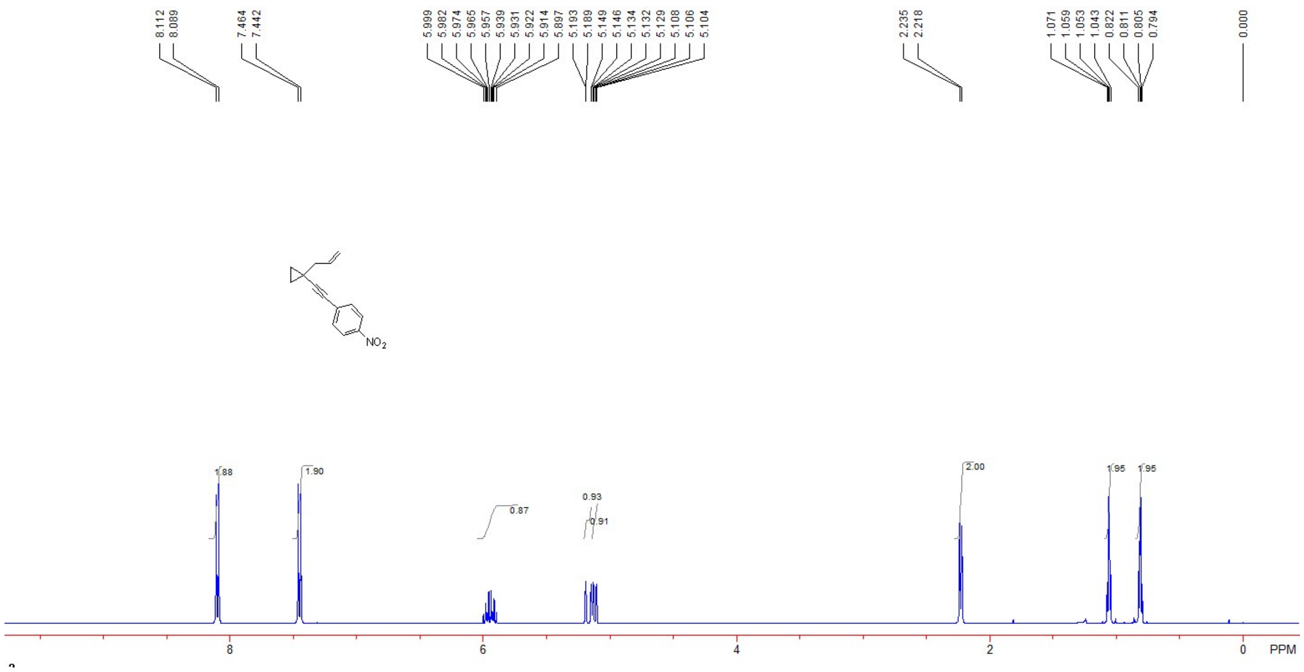

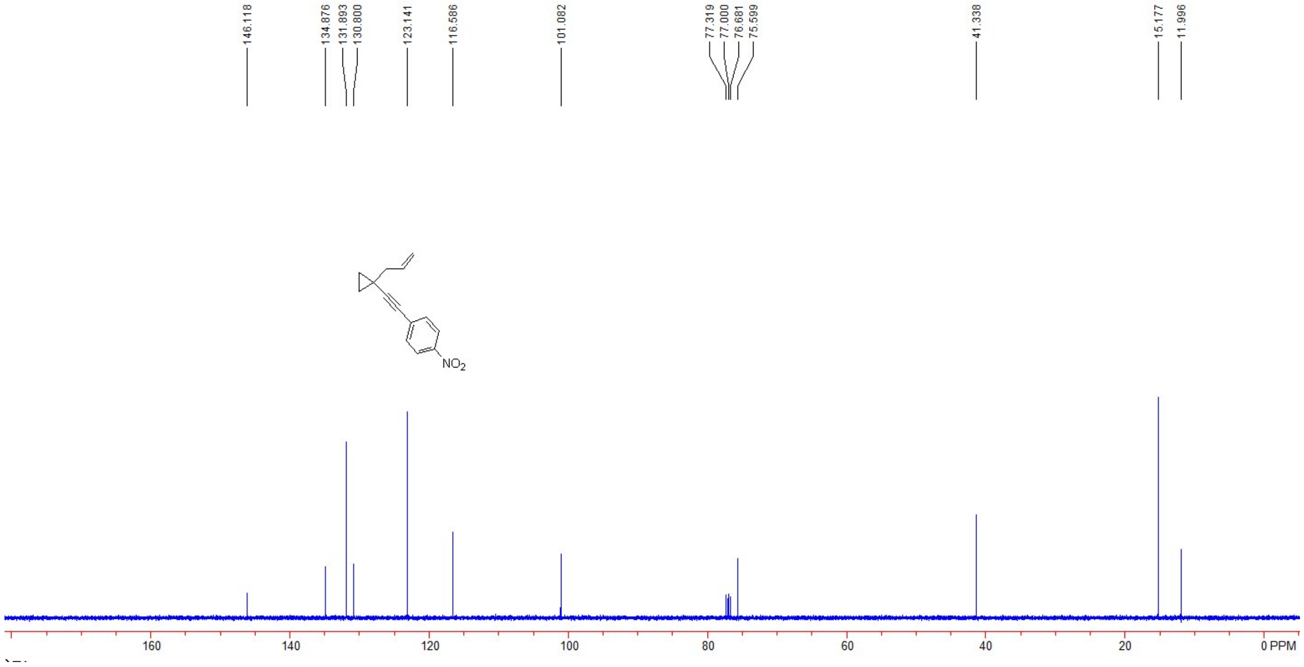

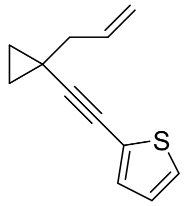

2-((1-Allylcyclopropyl)ethynyl)thiophene 1l:

0.313 g, yield = 83%. Colorless oil. ^{1}H NMR (400 MHz, $CDCl_3$, TMS): δ 0.73 (dd, J_1 = 6.4 Hz, J_2 = 4.4 Hz, 2H, CH_2), 1.01 (dd, J_1 = 6.4 Hz, J_2 = 4.4 Hz, 2H, CH_2), 2.20 (d, J = 6.8 Hz, 2H, CH_2), 5.09 (d, J = 11.2 Hz, 1H, =CH_2), 5.13 (d, J = 17.6 Hz, 1H, =CH_2), 5.89-5.98 (m, 1H, =CH), 6.89 (dd, J_1 =

5.6 Hz, J_2 = 3.6 Hz, 1H, Ar), 7.08 (d, J = 3.6 Hz, 1H, Ar), 7.12 (d, J = 5.6 Hz, 1H, Ar). ^{13}C NMR (100 MHz, $CDCl_3$, TMS): δ 12.1, 15.1, 41.7, 69.9, 98.7, 116.6, 124.0, 125.9, 126.7, 131.1, 135.3. IR (CH_2Cl_2) ν 3302, 3077, 2927, 1424, 1228, 1094, 915, 838, 695 cm^{-1}. MS (%) m/z 188 (M^+, 68.85), 173 (46.01), 160 (27.55), 147 (100.00), 128 (33.73), 115 (71.44), 103 (43.09), 77 (35.83), 45 (36.38). HRMS (EI) calcd. for $C_{12}H_{12}S$: 188.0660, found: 188.0659.

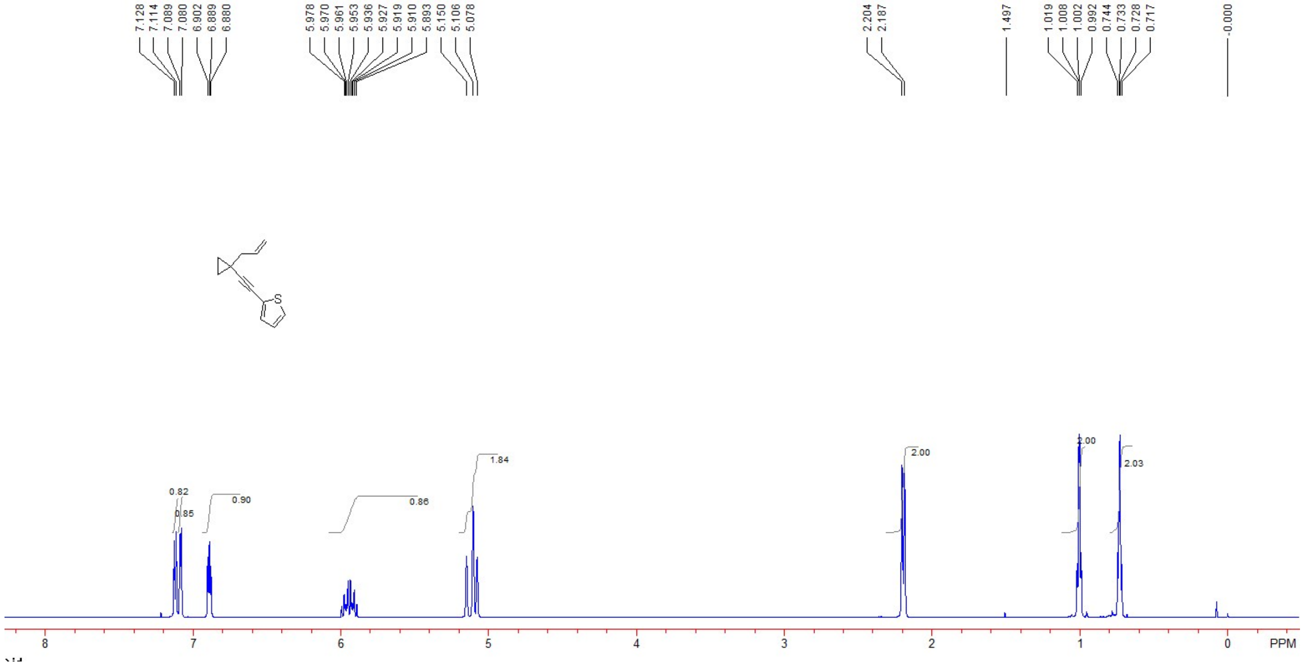

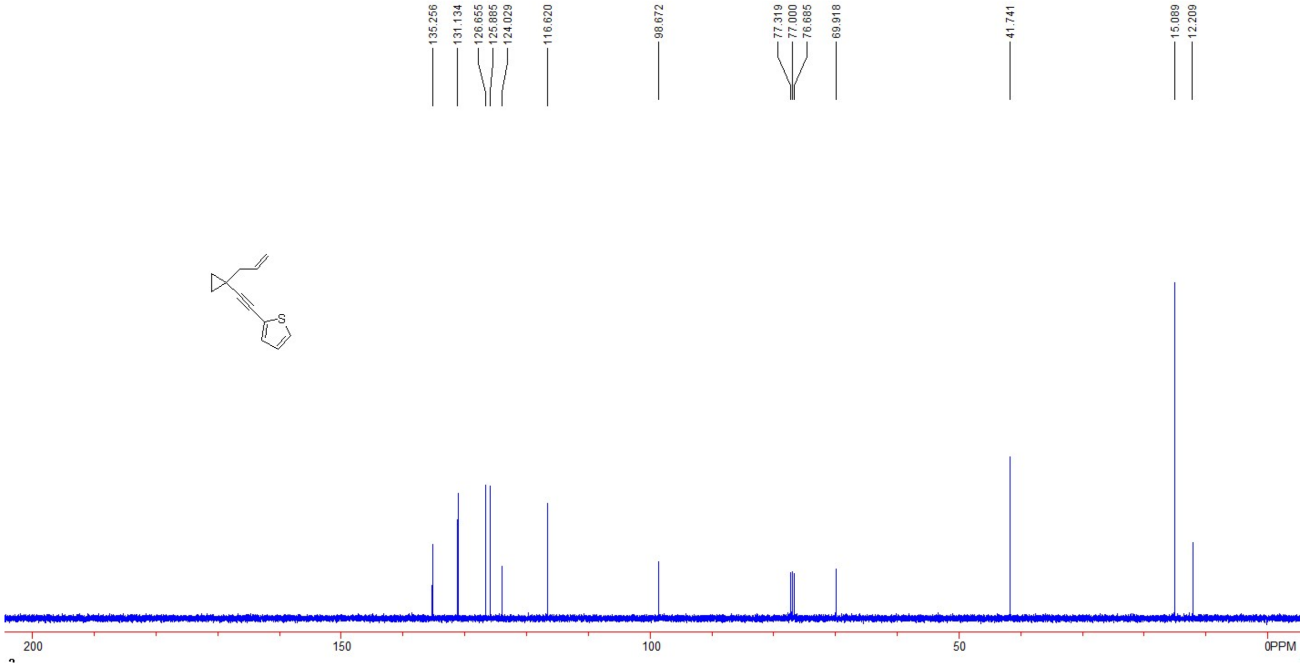

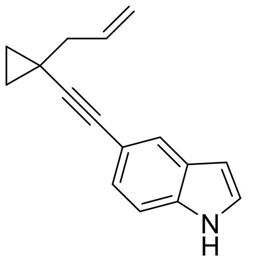

5-((1-Allylcyclopropyl)ethynyl)-1*H*-indole 1m':

0.450 g, yield = 68%. Colorless oil. ^{1}H NMR (400 MHz, $CDCl_3$, TMS): δ 0.71 (dd, J_1 = 6.4 Hz, J_2 = 4.4 Hz, 2H, CH_2), 1.00 (dd, J_1 = 6.4 Hz, J_2 = 4.4 Hz, 2H, CH_2), 2.22 (d, J = 6.4 Hz, 2H, CH_2), 5.08-5.18 (m, 2H, =CH_2), 5.96-6.07 (m, 1H, =CH), 6.43-6.44 (m, 1H, Ar), 7.04-7.07 (m, 1H, Ar), 7.14-7.21 (m, 2H, Ar), 7.70 (s, 1H, Ar), 7.98 (br, 1H, NH). ^{13}C NMR (100 MHz, $CDCl_3$, TMS): δ 12.1, 14.9, 42.2, 78.2, 92.0, 102.4, 110.9, 114.8, 116.4, 124.3, 124.9, 125.6, 127.6, 134.9, 135.7. IR (CH_2Cl_2) ν 3416, 3077, 2921, 1470, 1307, 1054, 909, 763 cm^{-1}. MS (%) m/z 221 (M^+, 100.00), 206 (37.97), 191 (22.70), 180 (76.54), 152 (36.24), 130 (13.65), 117 (9.44), 103 (2.98), 89 (11.96), 77 (12.19). HRMS (EI) calcd. for $C_{16}H_{15}N$: 221.1204, found: 221.1202.

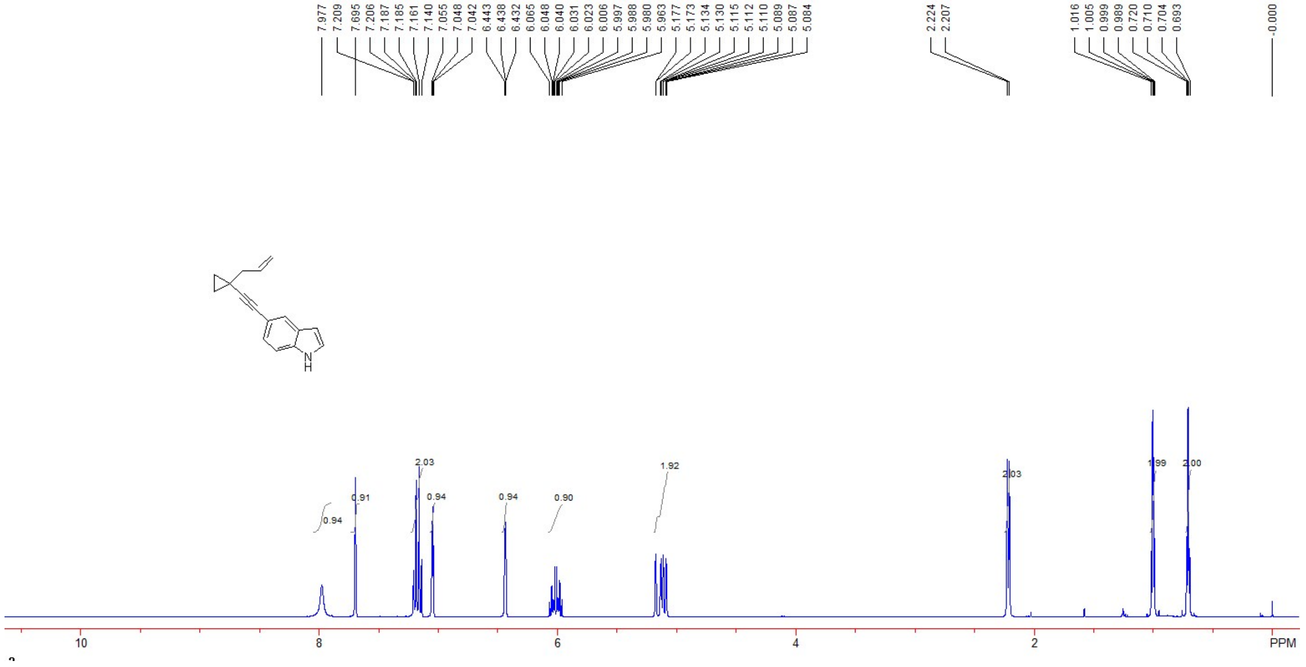

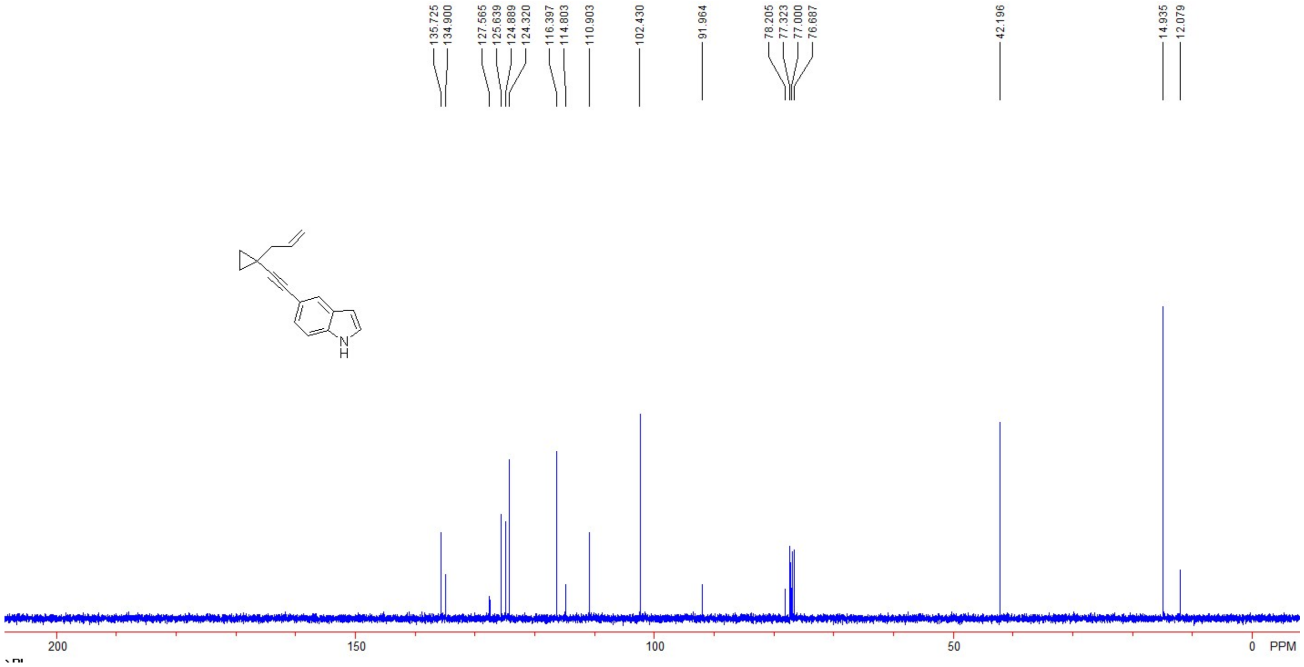

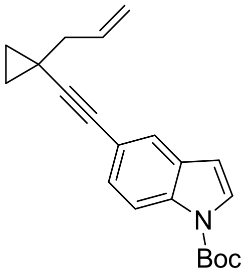

***tert*-Butyl 5-((1-allylcyclopropyl)ethynyl)-1*H*-indole-1-carboxylate 1m**:

0.232 g, yield = 23%. A white solid. Mp: 57-59 °C. ^{1}H NMR (400 MHz, $CDCl_3$, TMS): δ 0.71 (dd, J_1 = 6.0 Hz, J_2 = 4.4 Hz, 2H, CH_2), 1.01 (dd, J_1 = 6.0 Hz, J_2 = 4.4 Hz, 2H, CH_2), 1.62 (s, 9H, CH_3), 2.21 (d, J = 6.8 Hz, 2H, CH_2), 5.11 (d, J = 10.4 Hz, 1H, =CH_2), 5.17 (d, J = 17.6 Hz, 1H, =CH_2), 5.95-6.05 (m, 1H, =CH), 7.33 (d, J = 8.4 Hz, 1H, Ar), 7.53 (d, J = 2.0 Hz, 1H, Ar), 7.58 (s, 1H, Ar), 8.04 (d, J = 7.6 Hz, 1H, Ar). ^{13}C NMR (100 MHz, $CDCl_3$, TMS): δ 12.1, 15.0, 28.1, 42.1, 83.8, 93.1, 107.0, 114.9, 116.5, 118.0, 124.3, 126.5, 127.8, 130.3, 135.6, 149.5. IR (CH_2Cl_2) ν 2978, 2930, 1732, 1467, 1365, 1158, 1127, 1022, 836, 724 cm^{-1}. HRMS (ESI) Calcd. for $C_{21}H_{24}NO_2$ (M^++H) requires 322.1802, found: 322.1797.

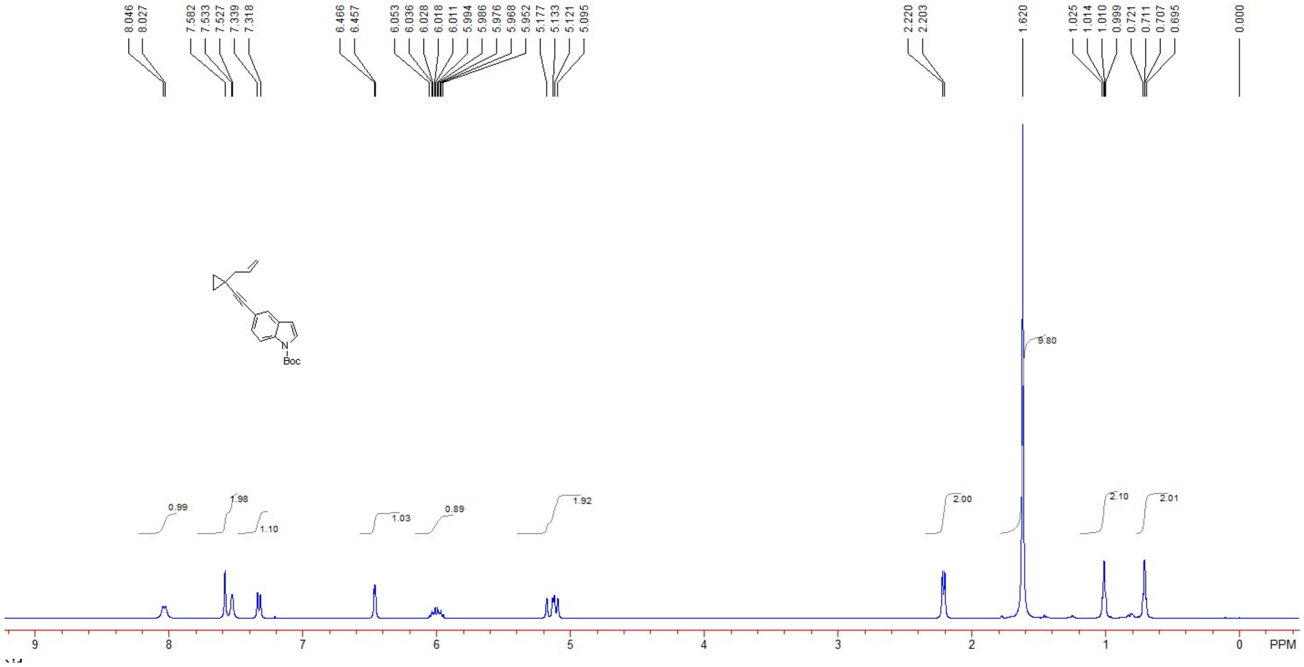

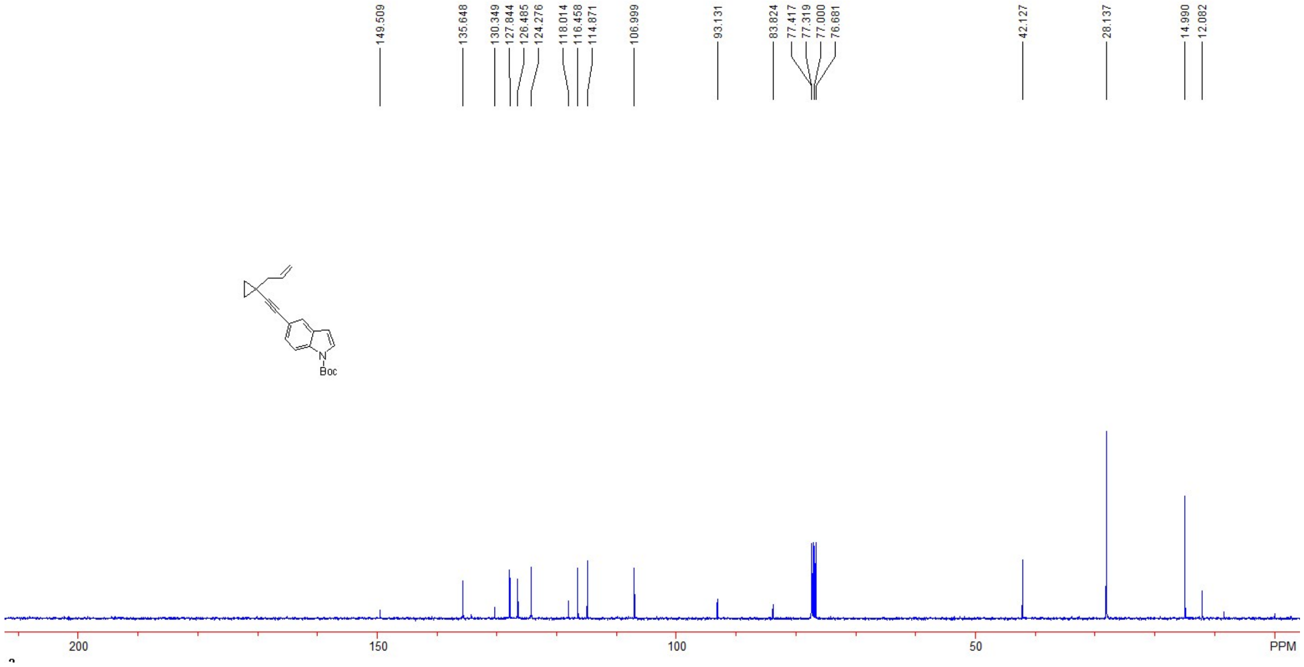

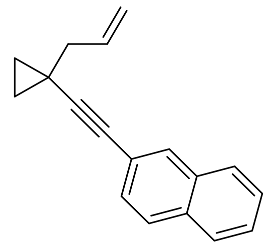

2-((1-Allylcyclopropyl)ethynyl)naphthalene 1n:

0.844 g, yield = 91%. Colorless oil. ^{1}H NMR (400 MHz, $CDCl_3$, TMS): δ 0.74 (dd, J_1 = 6.4 Hz, J_2 = 4.4 Hz, 2H, CH_2), 1.04 (dd, J_1 = 6.4 Hz, J_2 = 4.4 Hz, 2H, CH_2), 2.29 (d, J = 6.8 Hz, 2H, CH_2), 5.12 (d, J = 10.4 Hz, 1H, =CH_2), 5.17 (d, J = 17.2 Hz, 1H, =CH_2), 5.96-6.06 (m, 1H, =CH), 7.37-7.42 (m,

3H, Ar), 7.67-7.74 (m, 3H, Ar), 7.87 (s, 1H, Ar). ^{13}C NMR (100 MHz, $CDCl_3$, TMS): δ 12.1, 15.1, 42.0, 77.4, 95.2, 116.6, 121.2, 126.1, 126.3, 127.5, 127.6, 127.7, 128.8, 131.0, 132.3, 133.0, 135.5. IR (CH_2Cl_2) ν 3057, 3005, 2218, 1597, 1422, 916, 815, 744 cm^{-1}. MS (%) m/z 232 (M$^+$, 100.00), 217 (66.44), 202 (71.81), 191 (81.54), 165 (40.50), 152 (20.11), 128 (8.62), 115 (12.58), 101 (22.47). HRMS (EI) calcd. for $C_{18}H_{16}$: 232.1252, found: 232.1257.

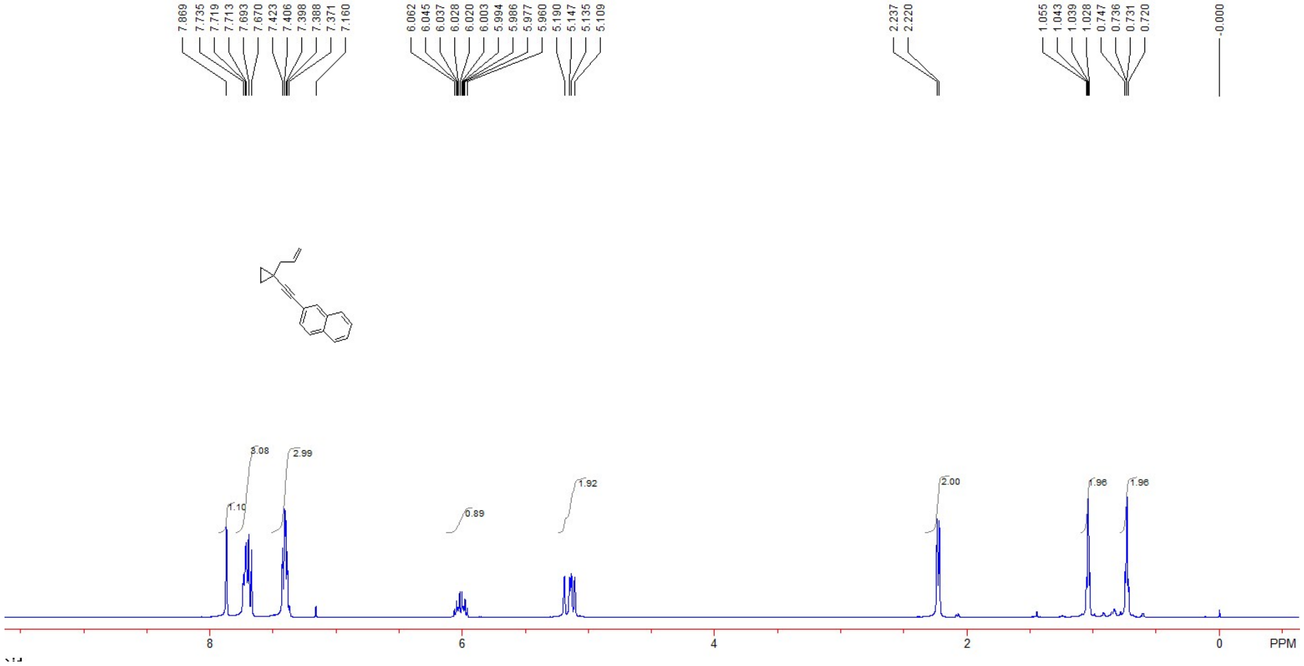

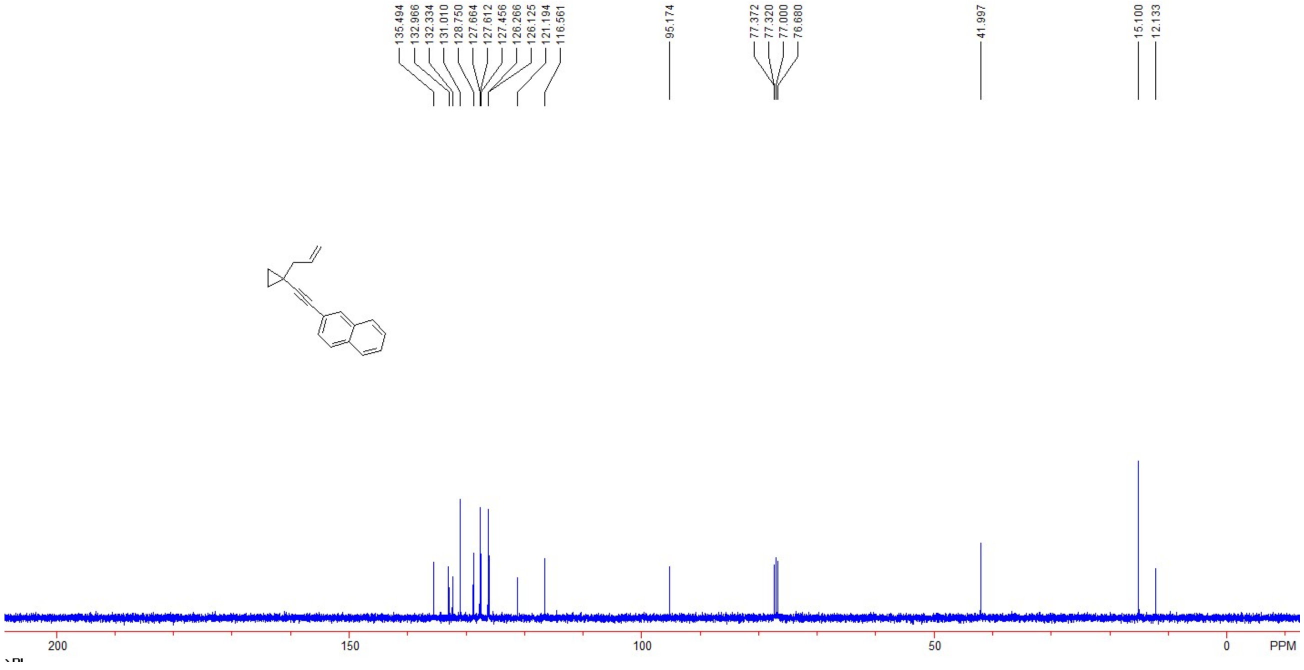

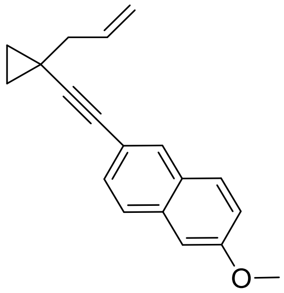

2-((1-Allylcyclopropyl)ethynyl)-6-methoxynaphthalene 1o:

0.953 g, yield = 91%. A white solid. Mp: 90-93 ^oC. ^1H NMR (400 MHz, $CDCl_3$, TMS): δ 0.75 (dd, J_1 = 6.4 Hz, J_2 = 4.4 Hz, 2H, CH_2), 1.04 (dd, J_1 = 6.4 Hz, J_2 = 4.4 Hz, 2H, CH_2), 2.24 (d, J = 6.8 Hz, 2H, CH_2), 3.89 (s, 3H, CH_3), 5.12 (dd, J_1 = 10.4 Hz, J_2 = 0.8 Hz, 1H, =CH_2), 5.17 (d, J = 17.2 Hz, 1H, =CH_2), 5.97-6.05 (m, 1H, =CH), 7.06 (d, J = 2.0 Hz, 1H, Ar), 7.11 (dd, J_1 = 8.8 Hz, J_2 = 2.4 Hz, 1H, Ar), 7.38-7.40 (m, 1H, Ar), 7.60 (d, J = 8.8 Hz, 1H, Ar), 7.64 (d, J = 8.8 Hz, 1H, Ar), 7.80 (s, 1H, Ar). ^{13}C NMR (100 MHz, $CDCl_3$, TMS): δ 12.1, 15.1, 42.1, 55.2, 77.4, 94.3, 105.7, 116.5, 118.8, 119.1, 126.5, 128.5, 129.1, 130.9, 133.6, 135.6, 157.9. IR (CH_2Cl_2) ν 3059. 2922, 2849, 1629, 1601, 1498, 1388, 1265, 1031, 850, 806 cm^{-1}. MS (%) m/z 262 (M^+, 100.00), 247 (24.61), 231 (19.16), 221 (62.69), 189 (32.20), 178 (40.62), 163 (24.07), 152 (20.32), 139 (9.58), 115 (7.05). HRMS (EI) calcd. for $C_{19}H_{18}O$: 262.1358, found: 262.1353.

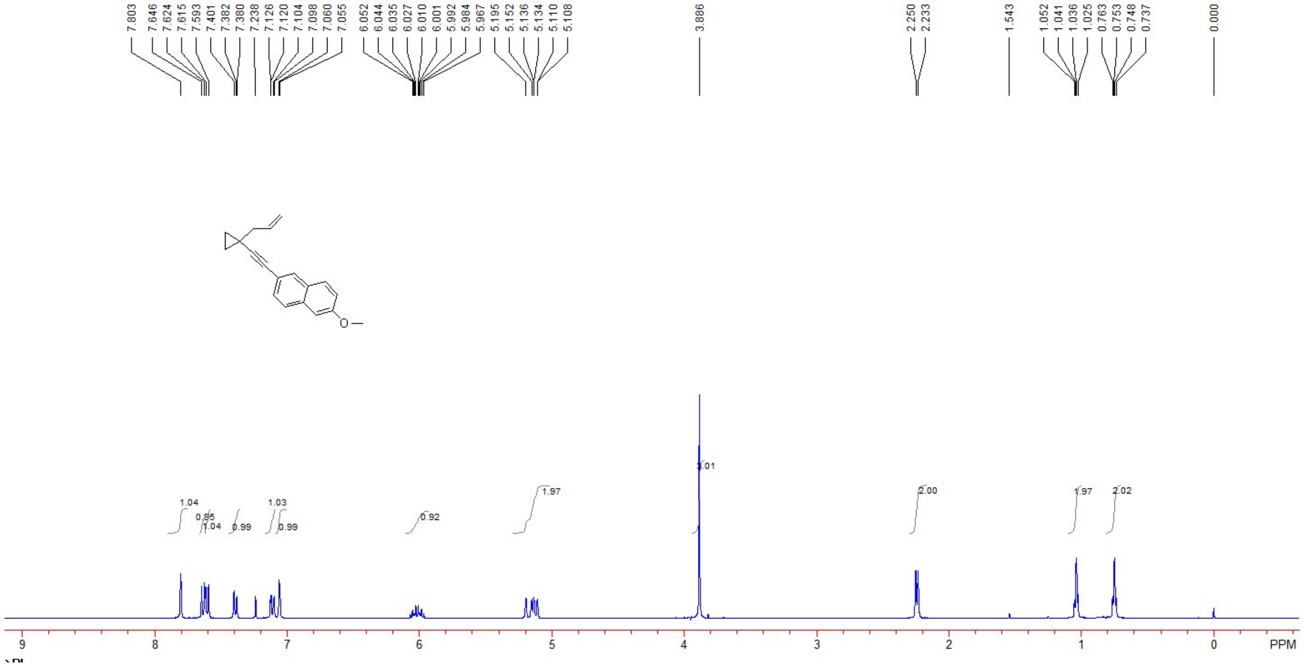

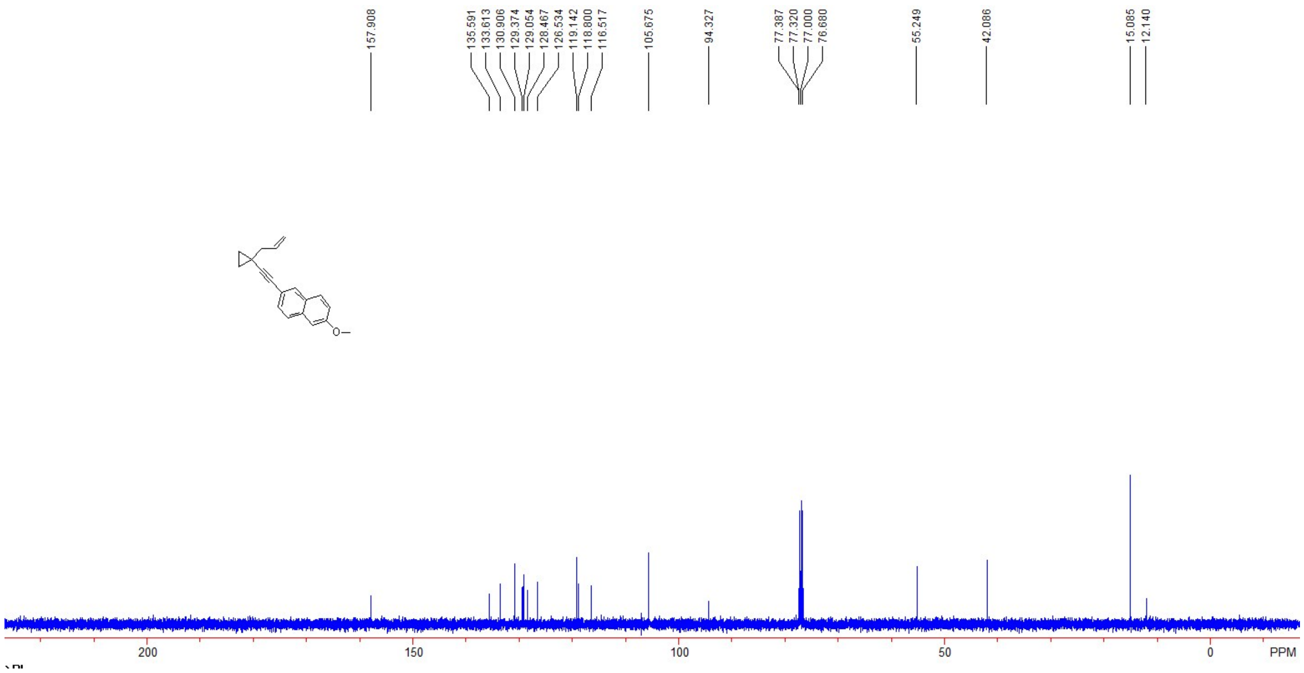

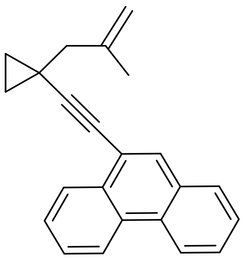

9-((1-(2-Methylallyl)cyclopropyl)ethynyl)phenanthrene 1p:

0.897 g, yield = 61%. Colorless oil. ^{1}H NMR (400 MHz, $CDCl_3$, TMS): δ 0.80-0.86 (m, 2H, CH_2), 1.18 (dd, J_1 = 6.4 Hz, J_2 = 4.4 Hz, 2H, CH_2), 1.92 (s, 3H, CH_3), 2.24 (s, 2H, CH_2), 4.94 (s, 1H, =CH_2), 5.04 (s, 1H, =CH_2), 7.46-7.57 (m, 2H, Ar), 7.58-7.62 (m, 2H, Ar), 7.72-7.74 (m, 1H, Ar), 7.85 (s, 1H, Ar), 8.44 (dd, J_1 = 6.4 Hz, J_2 = 3.2 Hz, 1H, Ar), 8.50-8.56 (m, 2H, Ar). ^{13}C NMR (100 MHz, $CDCl_3$, TMS): δ 11.6, 16.1, 22.9, 46.0, 75.0, 99.8, 112.4, 120.3, 122.5, 122.6, 126.71, 126.75, 126.9, 127.1, 128.1, 129.8, 130.0, 130.9, 131.3, 131.5, 143.7. IR (CH_2Cl_2) ν 3073, 2967, 2915, 2217, 1649, 1450, 1380, 888, 746, 723 cm^{-1}. MS (%) m/z 296 (M^+, 96.33), 281 (90.81), 266 (56.90), 253 (67.80), 239 (100.00), 226 (40.74), 215 (37.02), 202 (22.89), 165 (9.27), 126 (14.46). HRMS (EI) calcd. for $C_{23}H_{20}$: 296.1565, found: 296.1570.

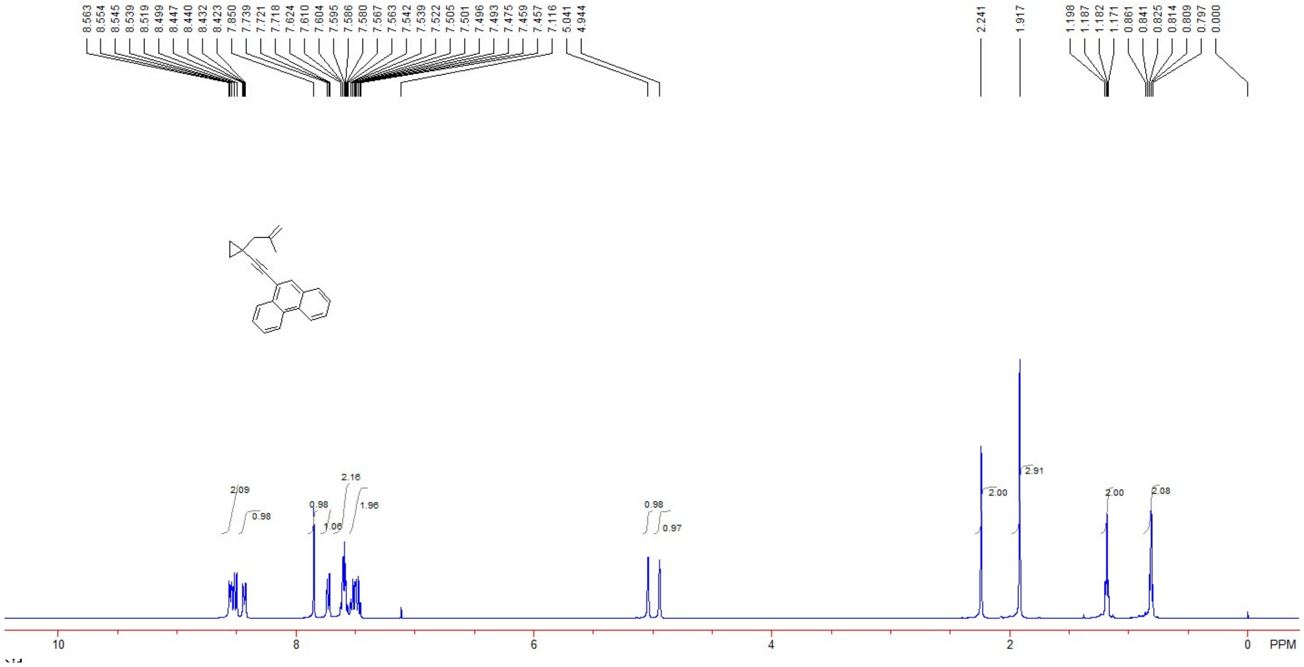

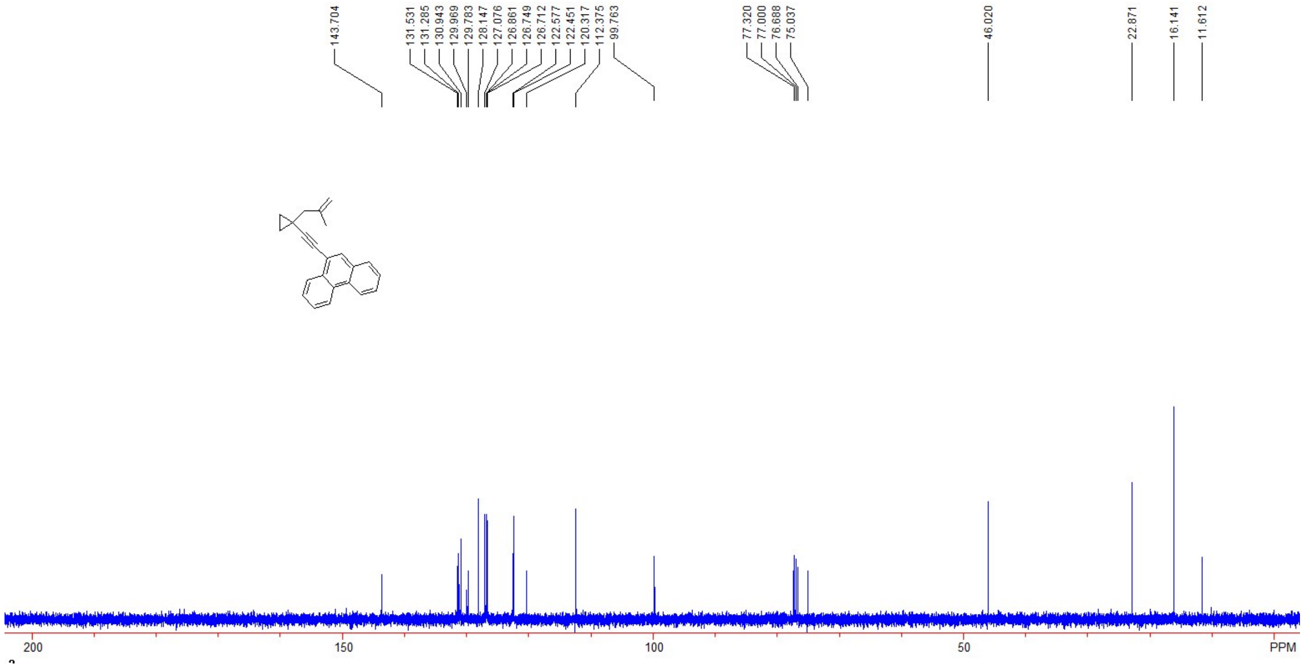

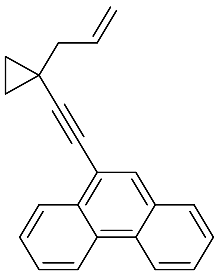

9-((1-Allylcyclopropyl)ethynyl)phenanthrene 2a:

0.757 g, yield = 93%. A white solid. Mp: 60-62 ^{o}C. ^{1}H NMR (400 MHz, $CDCl_3$, TMS): δ 0.79 (dd, J_1 = 6.8 Hz, J_2 = 4.4 Hz, 2H, CH_2), 1.13 (dd, J_1 = 6.8 Hz, J_2 = 4.4 Hz, 2H, CH_2), 2.29 (d, J = 6.8 Hz,

2H, CH_2), 5.16-5.26 (m, 2H, $=CH_2$), 6.04-6.11 (m, 1H, =CH), 7.46-7.49 (m, 2H, Ar), 7.53-7.63 (m, 2H, Ar), 7.87 (s, 1H, Ar), 8.41-8.43 (m, 1H, Ar), 8.50-8.56 (m, 2H, Ar). ^{13}C NMR (100 MHz, $CDCl_3$, TMS): δ 12.4, 15.4, 42.1, 75.2, 99.6, 116.7, 120.2, 122.5, 122.6, 126.7, 126.8, 126.9, 127.0, 128.2, 129.8, 130.0, 131.1, 131.3, 131.5, 135.6. IR (CH_2Cl_2) ν 3074, 3005, 2900, 2217, 1451, 915, 746, 723 cm^{-1}. MS (%) m/z 282 (M^+, 100.00), 267 (35.68), 252 (54.98), 239 (82.16), 226 (31.43), 215 (22.58), 202 (14.84), 126 (15.99), 113 (11.97). HRMS (EI) calcd. for $C_{22}H_{18}$: 282.1409, found: 282.1412.

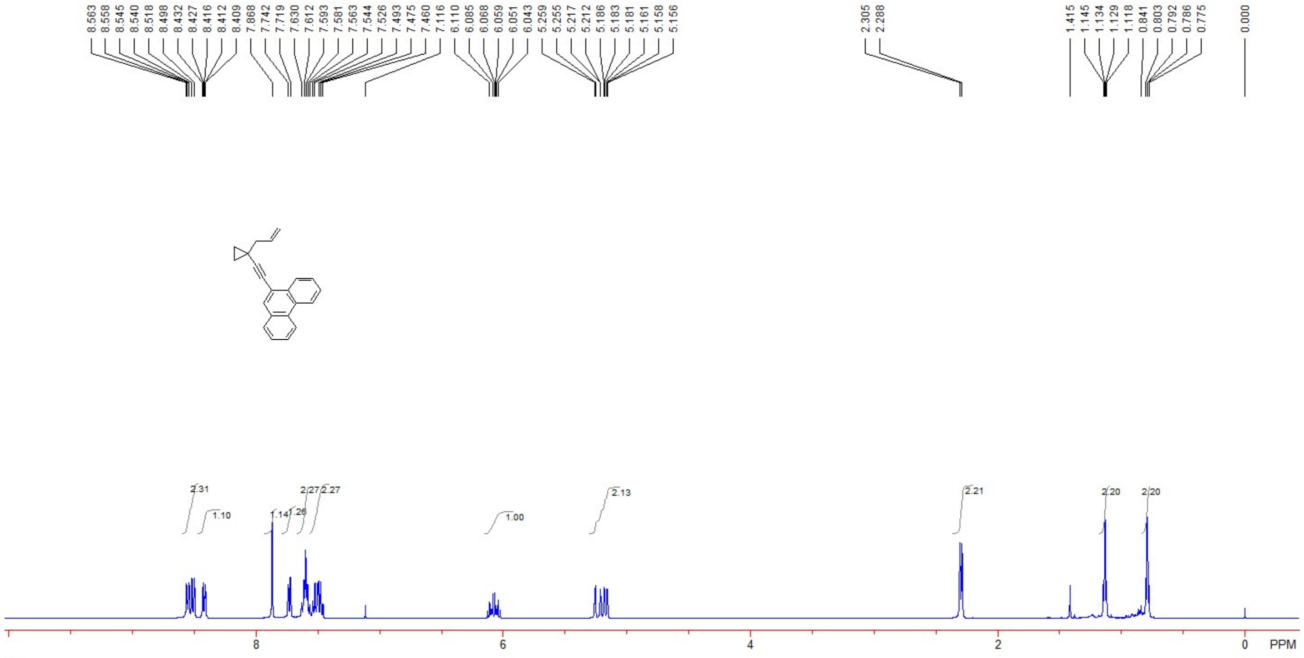

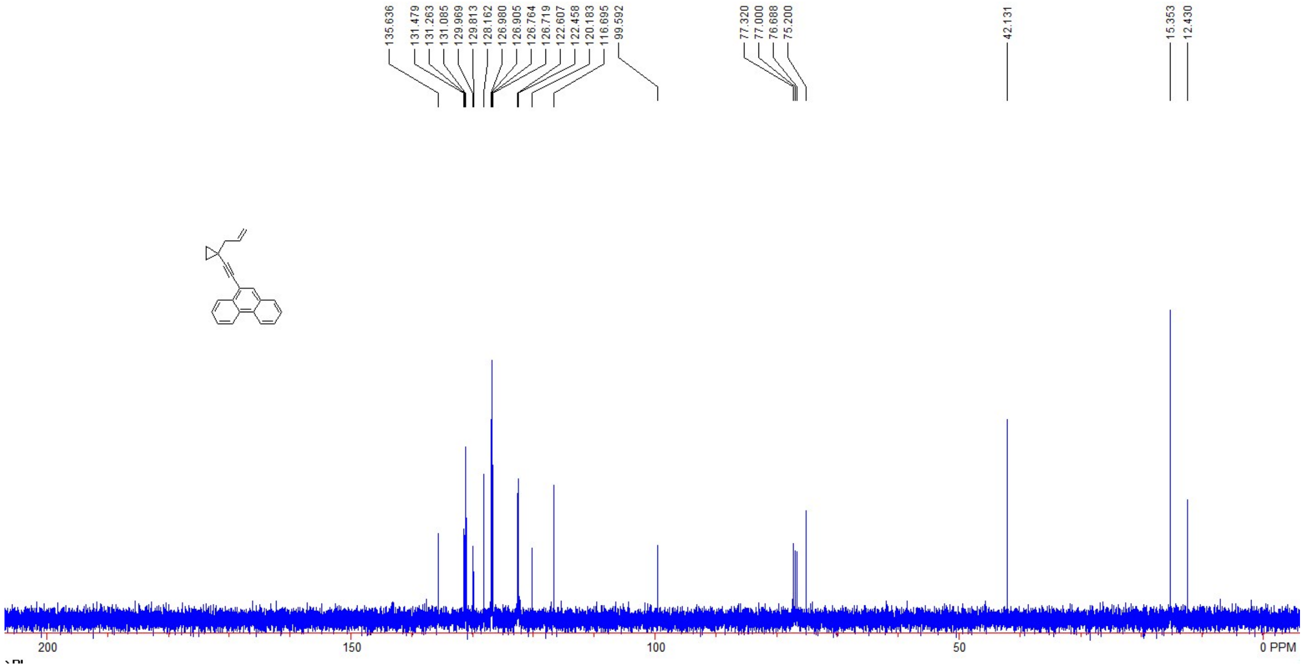

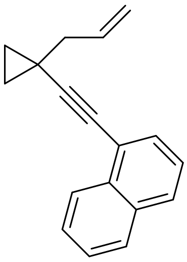

1-((1-Allylcyclopropyl)ethynyl)naphthalene 2b:

0.757 g, yield = 93%. Colorless oil. ^{1}H NMR (400 MHz, $CDCl_3$, TMS): δ 0.75 (dd, J_1 = 6.0 Hz, J_2 = 4.4 Hz, 2H, CH_2), 1.10 (dd, J_1 = 6.0 Hz, J_2 = 4.4 Hz, 2H, CH_2), 2.26 (d, J = 6.8 Hz, 2H, CH_2), 5.15 (d, J = 10.4 Hz, 1H, =CH_2), 5.21 (d, J = 17.2 Hz, 1H, =CH_2), 6.00-6.10 (m, 1H, =CH), 7.28-7.32 (m, 1H, Ar), 7.42 (dd, J_1 = 7.6 Hz, J_2 = 7.2 Hz, 1H, Ar), 7.48-7.52 (m, 1H, Ar), 7.57 (d, J = 7.2 Hz, 1H, Ar), 7.67 (d, J = 8.0 Hz, 1H, Ar), 7.74 (d, J = 8.0 Hz, 1H, Ar), 8.33 (d, J = 7.2 Hz, 1H, Ar). ^{13}C NMR (100 MHz, $CDCl_3$, TMS): δ 12.4, 15.3, 42.1, 75.0, 99.9, 116.6, 121.5, 125.1, 126.1, 126.2, 126.4, 127.7, 128.1, 129.9, 133.1, 133.5, 135.6. IR (CH_2Cl_2) ν 3058, 3005, 2901, 1641, 1398, 996, 915, 797, 771 cm^{-1}. MS (%) m/z 232 (M^+, 100.00), 217 (51.12), 202 (77.32), 189 (89.58), 176 (29.02), 165 (36.71), 152 (25.87), 126 (7.43), 101 (30.64). HRMS (EI) calcd. for $C_{18}H_{16}$: 232.1252, found: 232.1253.

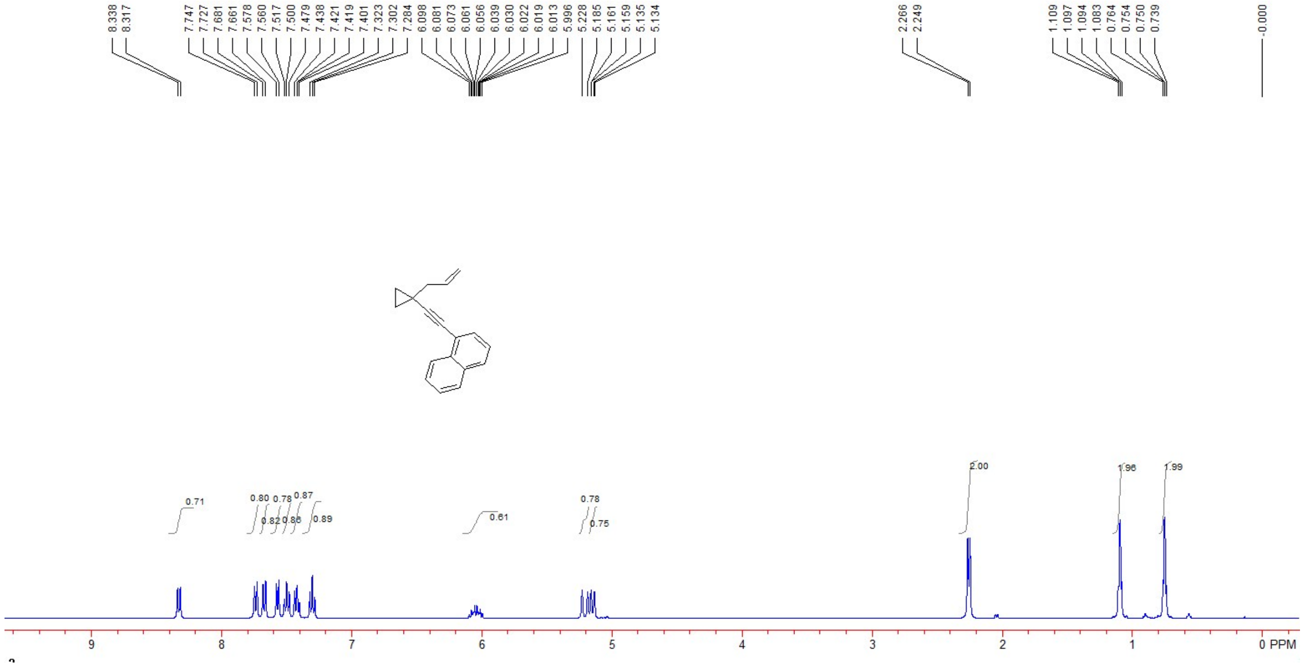

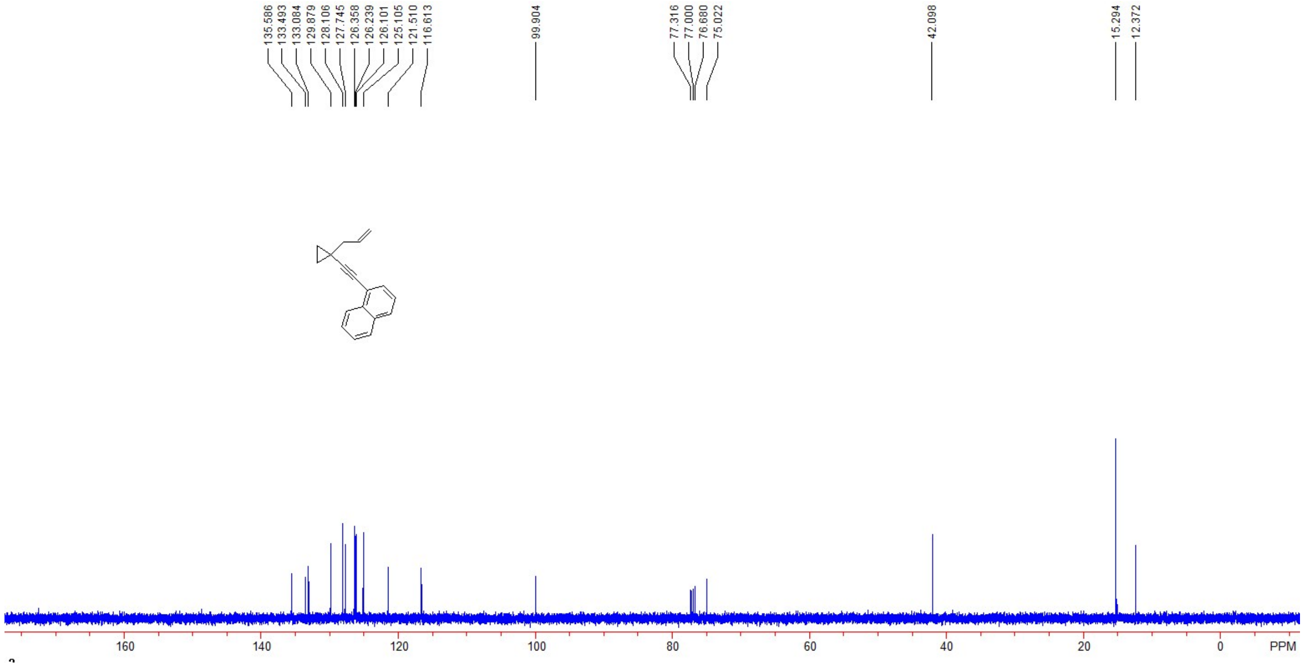

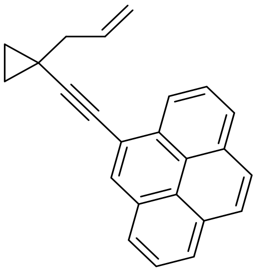

4-((1-Allylcyclopropyl)ethynyl)pyrene 2c:

0.858 g, yield = 70%. A white solid. Mp: 95-98 ^{o}C. ^{1}H NMR (400 MHz, $CDCl_3$, TMS): δ 0.87 (dd, J_1 = 6.8 Hz, J_2 = 4.4 Hz, 2H, CH_2), 1.20 (dd, J_1 = 6.8 Hz, J_2 = 4.4 Hz, 2H, CH_2), 2.37 (d, J = 7.2 Hz, 2H, CH_2), 5.20-5.31 (m, 2H, =CH_2), 6.10-6.17 (m, 1H, =CH), 7.94-7.99 (m, 3H, Ar), 8.01-8.02 (m, 2H, Ar), 8.08-8.16 (m, 3H, Ar), 8.51 (d, J = 8.8 Hz, 1H, Ar). ^{13}C NMR (100 MHz, $CDCl_3$, TMS): δ 12.6, 15.5, 42.3, 76.1, 100.8, 116.7, 118.7, 124.4, 125.2, 125.7, 126.0, 127.2, 127.6, 127.9, 129.5, 130.5, 131.1, 131.2, 131.9, 135.7. IR (CH_2Cl_2) ν 3040, 2921, 2850, 2214, 1434, 915, 841, 716 cm^{-1}. MS (%) m/z 306 (M^+, 100.00), 291 (21.83), 276 (36.76), 263 (47.49), 250 (24.38), 239 (19.26), 145 (7.49), 138 (14.77), 125 (7.42). HRMS (EI) calcd. for $C_{24}H_{18}$: 306.1409, found: 306.1408.

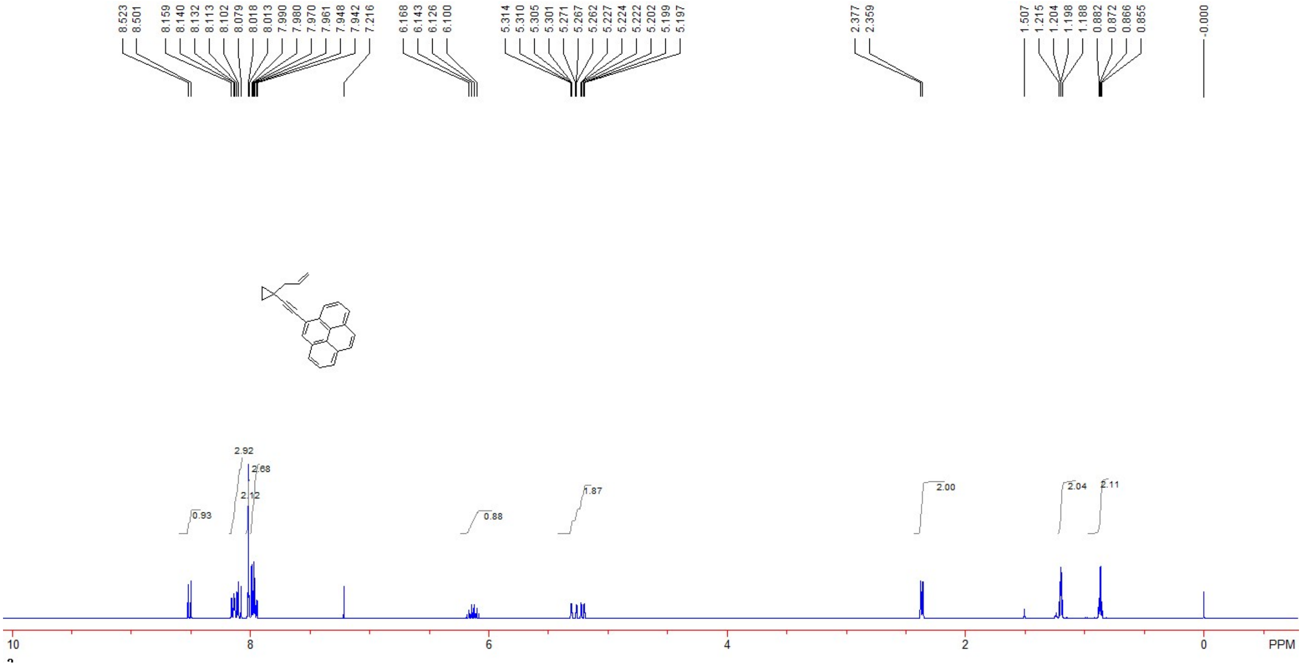

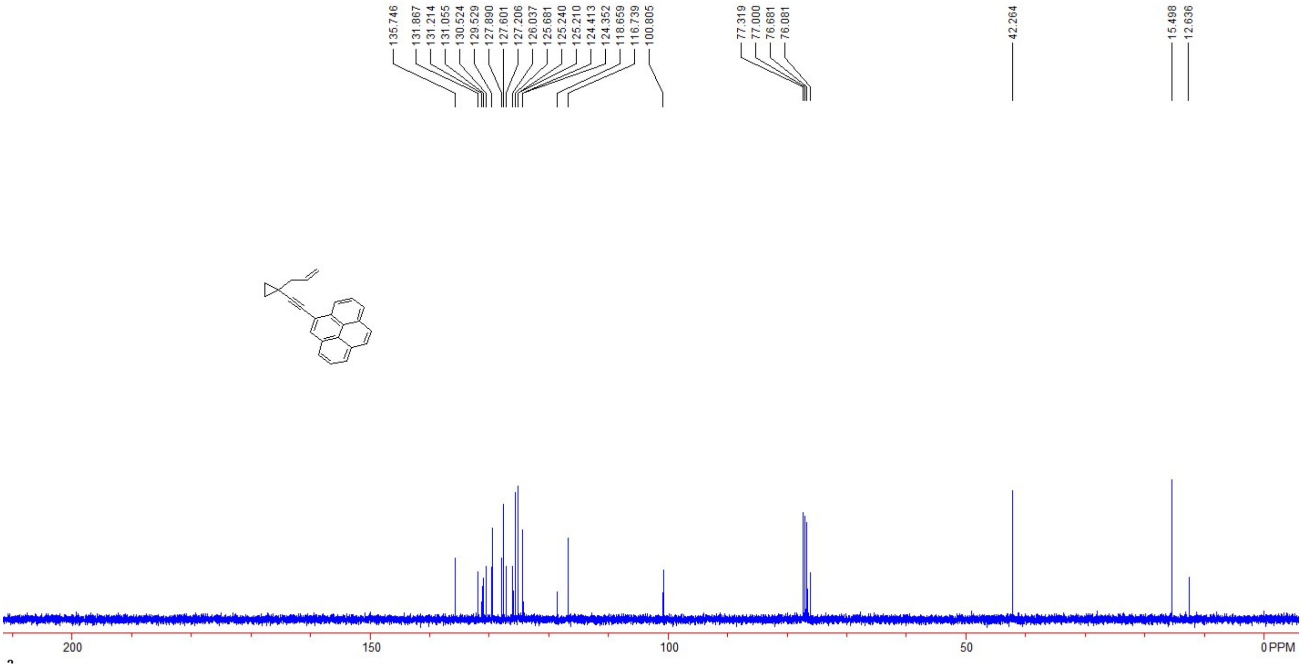

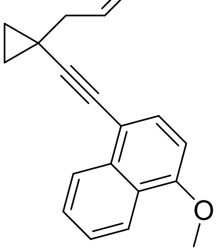

1-((1-Allylcyclopropyl)ethynyl)-4-methoxynaphthalene 2d:

0.910 g, yield = 87%. Colorless oil. ^{1}H NMR (400 MHz, $CDCl_3$, TMS): δ 0.79 (dd, J_1 = 6.8 Hz, J_2 = 4.4 Hz, 2H, CH_2), 1.10 (dd, J_1 = 6.8 Hz, J_2 = 4.4 Hz, 2H, CH_2), 2.29 (d, J = 6.8 Hz, 2H, CH_2), 3.95 (s,

3H, CH_3), 5.13-5.24 (m, 2H, $=CH_2$), 6.04-6.10 (m, 1H, =CH), 6.86 (d, J = 8.4 Hz, 2H, Ar), 7.45-7.57 (m, 3H, Ar), 8.25 (dd, J_1 = 9.6 Hz, J_2 = 9.2 Hz, 2H, Ar). ^{13}C NMR (100 MHz, $CDCl_3$, TMS): δ 12.4, 15.2, 42.3, 55.5, 75.0, 97.9, 103.4, 113.6, 116.5, 122.1, 125.3, 125.5, 126.1, 126.9, 130.4, 134.4, 135.8, 155.2. IR (CH_2Cl_2) ν 3066, 3004, 2839, 2220, 1583, 1317, 1094, 914, 762 cm^{-1}. MS (%) m/z 262 (M^+, 100.00), 247 (24.24), 231 (32.69), 221 (37.61), 203 (16.85), 189 (32.74), 178 (27.08), 163 (18.82), 152 (16.34), 139 (8.38), 115 (6.44). HRMS (EI) calcd. for $C_{19}H_{18}O$: 262.1358, found: 262.1355.

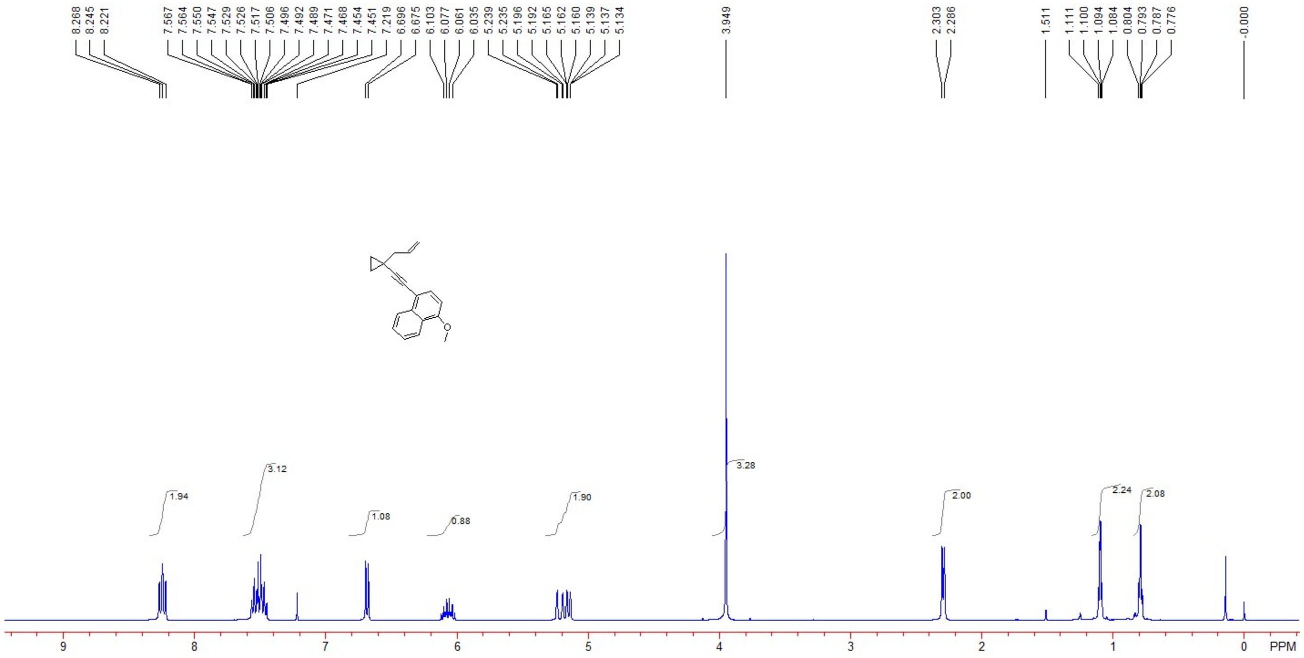

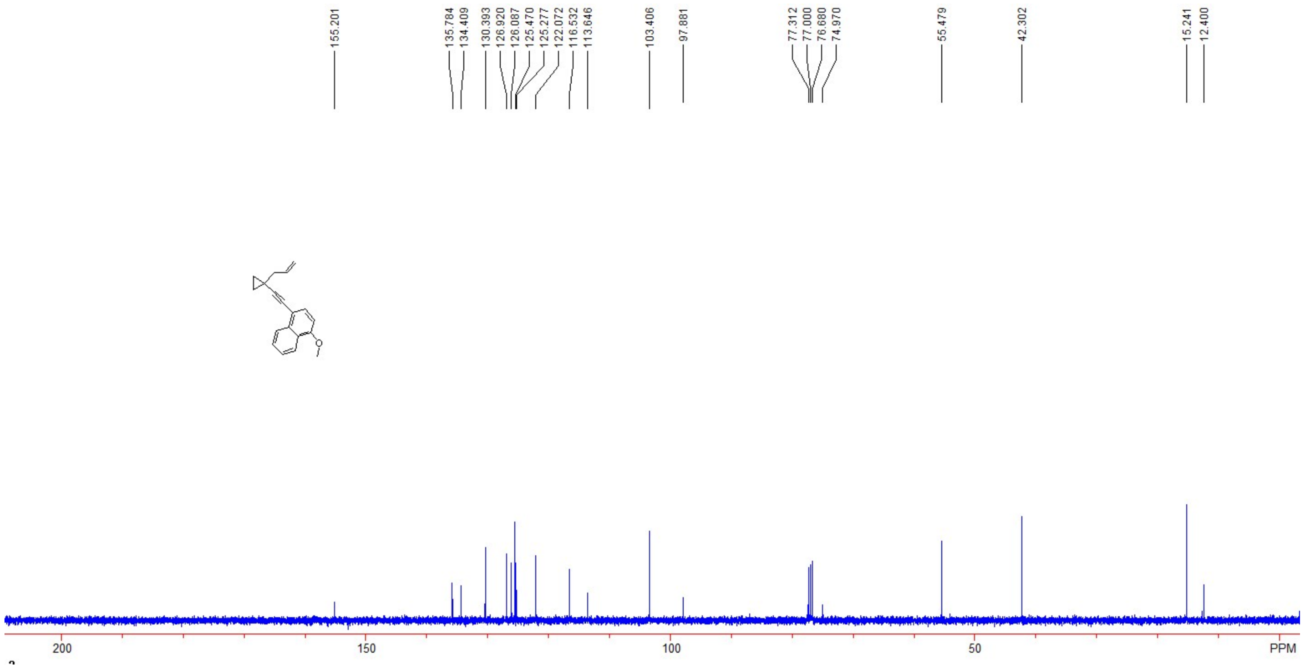

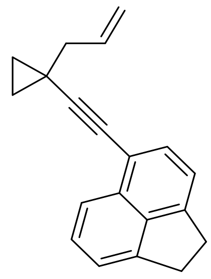

5-((1-Allylcyclopropyl)ethynyl)-1,2-dihydroacenaphthylene 2e:

0.758 g, yield = 73%. A white solid. Mp: 65-67 °C. ^{1}H NMR (400 MHz, $CDCl_3$, TMS): δ 0.78 (dd, J_1 = 6.4 Hz, J_2 = 4.4 Hz, 2H, CH_2), 1.10 (dd, J_1 = 6.4 Hz, J_2 = 4.4 Hz, 2H, CH_2), 2.28 (d, J = 6.8 Hz, 2H, CH_2), 3.28-3.34 (m, 4H, CH_2), 5.14 (d, J = 10.0 Hz, 1H, =CH_2), 5.21 (dd, J_1 = 17.2 Hz, J_2 = 1.6 Hz, 1H, CH_2), 6.01-6.12 (m, 1H, =CH), 7.13 (d, J = 7.2 Hz, 1H, Ar), 7.24 (d, J = 6.8 Hz, 1H, Ar), 7.44-7.51 (m, 2H, Ar), 7.85 (d, J = 8.4 Hz, 1H, Ar). ^{13}C NMR (100 MHz, $CDCl_3$, TMS): δ 12.4, 15.3, 30.3, 30.4, 42.3, 74.8, 98.4, 116.5, 118.8, 119.6, 121.1, 128.2, 131.6, 132.0, 135.7, 138.8, 146.0. IR (CH_2Cl_2) ν 3061, 2920, 2836, 2217, 1607, 1422, 913, 836, 774 cm^{-1}. MS (%) m/z 258 (M^+, 100.00), 243 (28.91), 229 (29.53), 217 (39.03), 202 (60.77), 189 (24.67), 152 (10.40), 113 (6.94), 101 (10.68). HRMS (EI) calcd. for $C_{20}H_{18}$: 258.1409, found: 258.1411.

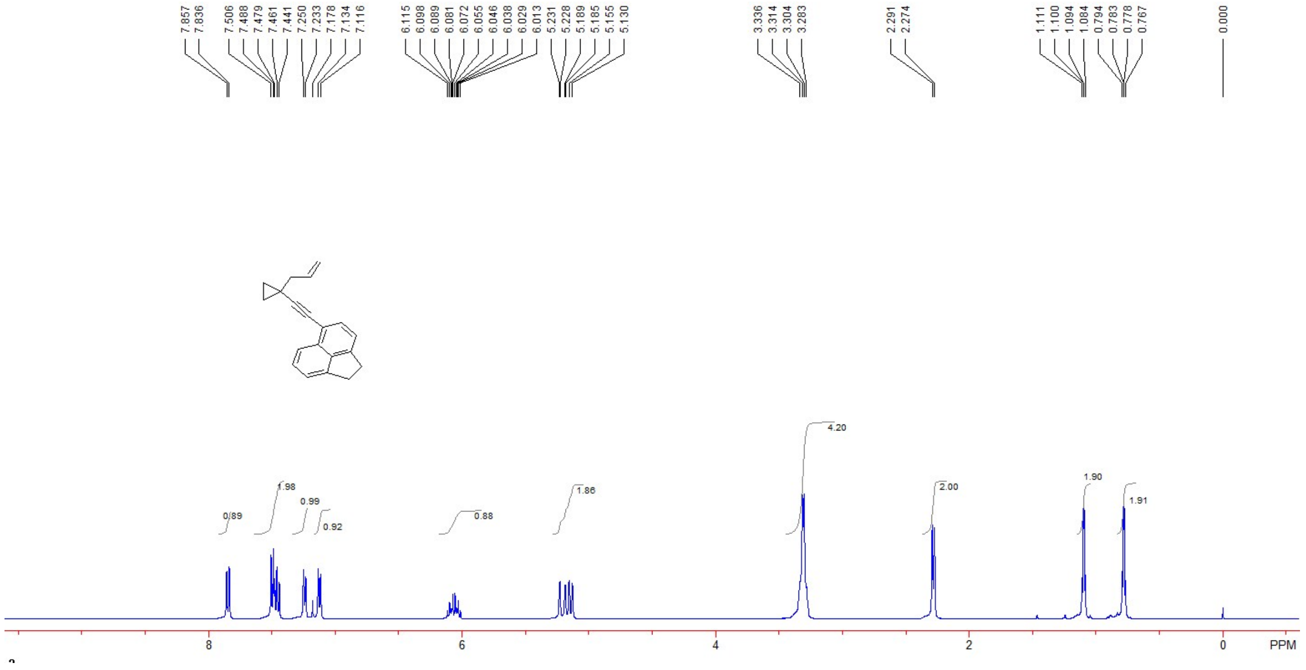

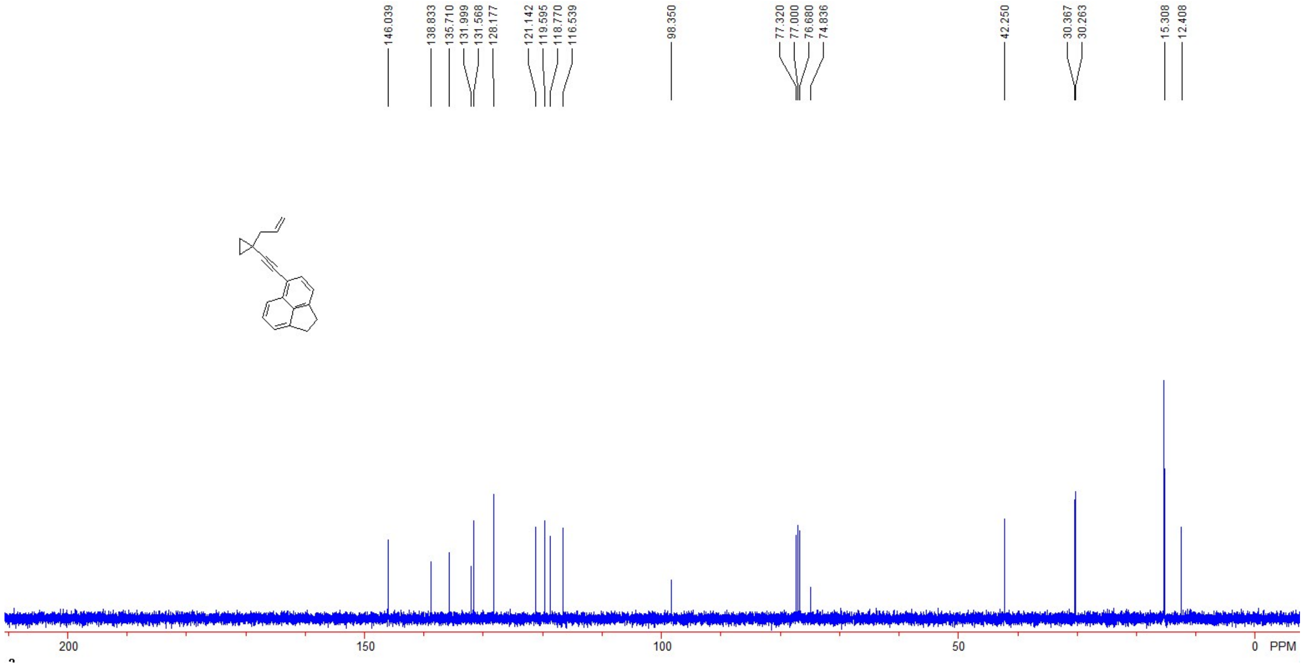

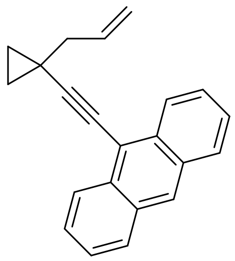

9-((1-Allylcyclopropyl)ethynyl)anthracene 2f:

0.913 g, yield =81%. A white solid. Mp: 49-51 °C. ^{1}H NMR (400 MHz, $CDCl_3$, TMS): δ 0.90 (dd, J_1 = 6.4 Hz, J_2 = 4.4 Hz, 2H, CH_2), 1.24 (dd, J_1 = 6.4 Hz, J_2 = 4.4 Hz, 2H, CH_2), 2.39 (d, J = 6.8 Hz, 2H, CH_2), 5.20-5.23 (m, 1H, =CH_2), 5.27-5.33 (m, 1H, =CH_2), 6.10-6.18 (m, 1H, =CH), 7.42-7.46 (m, 2H, Ar), 7.49-7.53 (m, 2H, Ar), 7.93 (dd, J_1 = 8.0 Hz, J_2 =0.4 Hz, 2H, Ar), 8.29 (s, 1H, Ar), 8.50 (dd, J_1 = 8.0 Hz, J_2 =0.8 Hz, 2H, Ar). ^{13}C NMR (100 MHz, $CDCl_3$, TMS): δ 12.9, 15.8, 42.3, 106.7, 116.8, 118.2, 125.5, 126.1, 126.5, 126.9, 128.5, 131.1, 132.6, 135.8. IR (CH_2Cl_2) ν 3053, 3004, 2922, 2211, 1440, 1417, 916, 843, 734 cm^{-1}. MS (%) m/z 282 (M^+, 100.00), 267 (23.44), 252 (33.29), 239 (52.21), 226 (39.38), 215 (19.37), 126 (12.47), 113 (11.18). HRMS (EI) calcd. for $C_{22}H_{18}$: 282.1409, found: 282.1415.

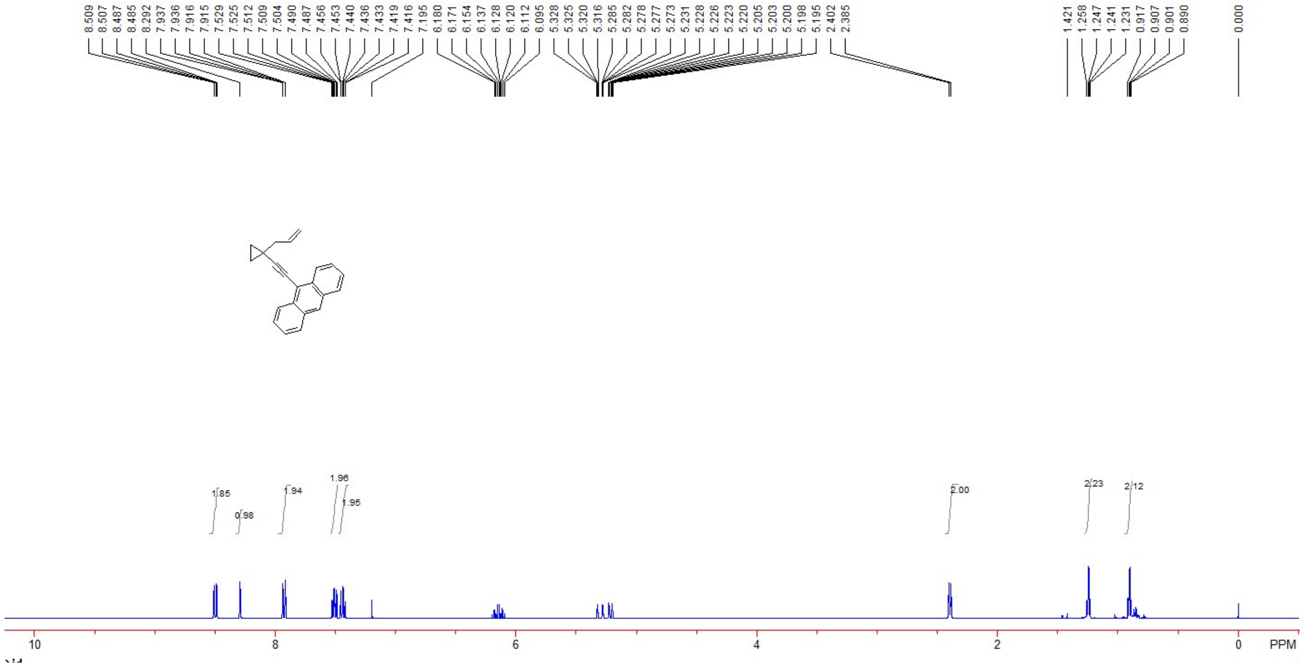

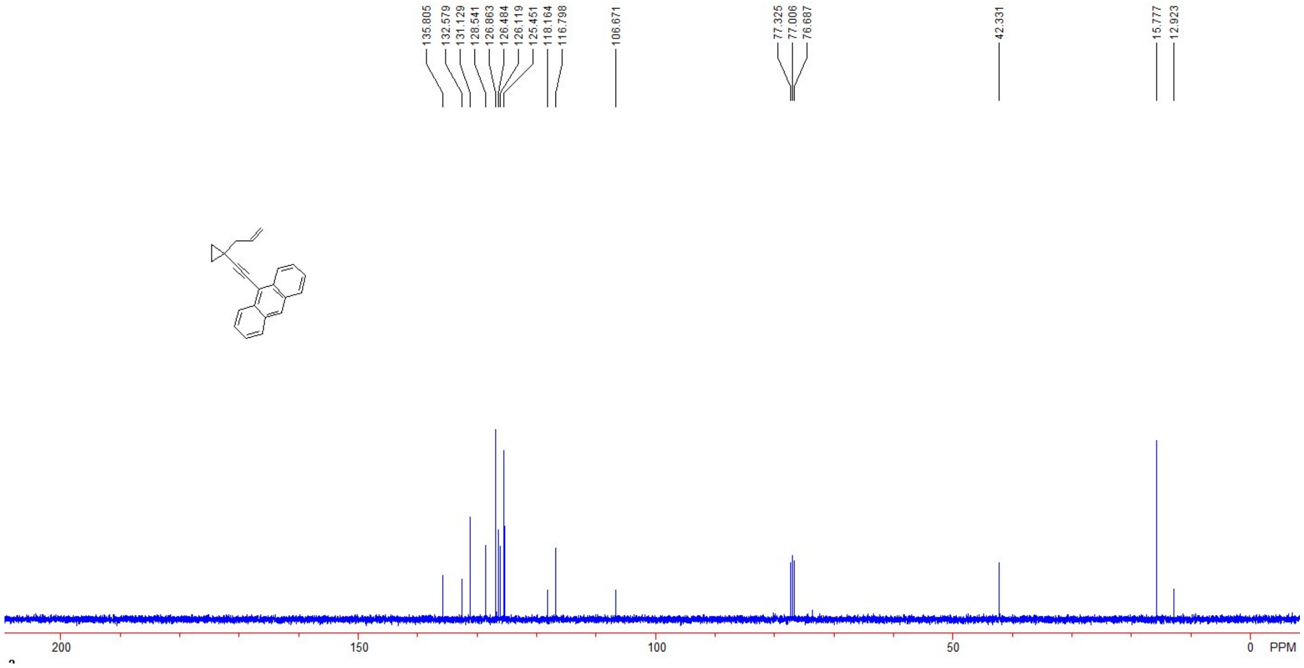

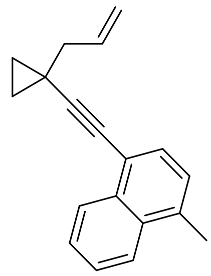

1-((1-Allylcyclopropyl)ethynyl)-4-methylnaphthalene 2g:

0.853 g, yield = 87%. Colorless oil. ^{1}H NMR (400 MHz, $CDCl_3$, TMS): δ 0.79 (dd, J_1 = 6.8 Hz, J_2 = 4.4 Hz, 2H, CH_2), 1.10 (dd, J_1 = 6.8 Hz, J_2 = 4.4 Hz, 2H, CH_2), 2.29 (d, J = 6.8 Hz, 2H, CH_2), 2.62 (s,

3H, CH_3), 5.13-5.24 (m, 2H, =CH_2), 6.03-6.10 (m, 1H, =CH), 7.18 (d, J = 7.2 Hz, 1H, Ar), 7.46-7.54 (m, 3H, Ar), 7.93 (dd, J_1 = 7.2 Hz, J_2 = 2.0 Hz, 1H, Ar), 8.35 (dd, J_1 = 6.8 Hz, J_2 = 1.6 Hz, 1H, Ar). ^{13}C NMR (100 MHz, $CDCl_3$, TMS): δ 12.4, 15.3, 19.5, 42.2, 75.2, 99.2, 116.6, 119.8, 124.3, 125.95, 126.02, 126.9, 129.7, 132.3, 133.5, 134.4, 135.7. IR (CH_2Cl_2) ν 3077, 3006, 2922, 1580, 1421, 1394, 915, 830, 758 cm^{-1}. MS (%) m/z 246 (M^+, 100.00), 231 (71.87), 216 (43.72), 202 (47.21), 189 (78.84), 179 (13.55), 165 (17.25), 152 (8.75), 101 (16.13), 94 (3.61). HRMS (EI) calcd. for $C_{19}H_{18}$: 246.1409, found: 246.1406.

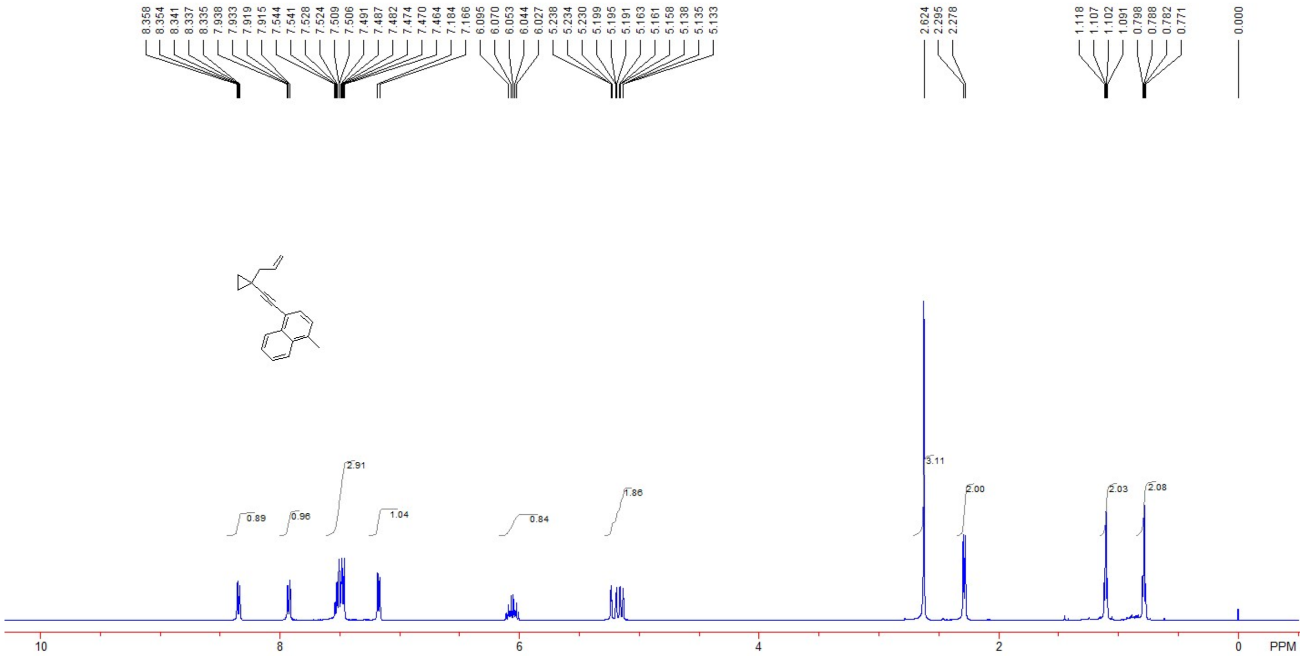

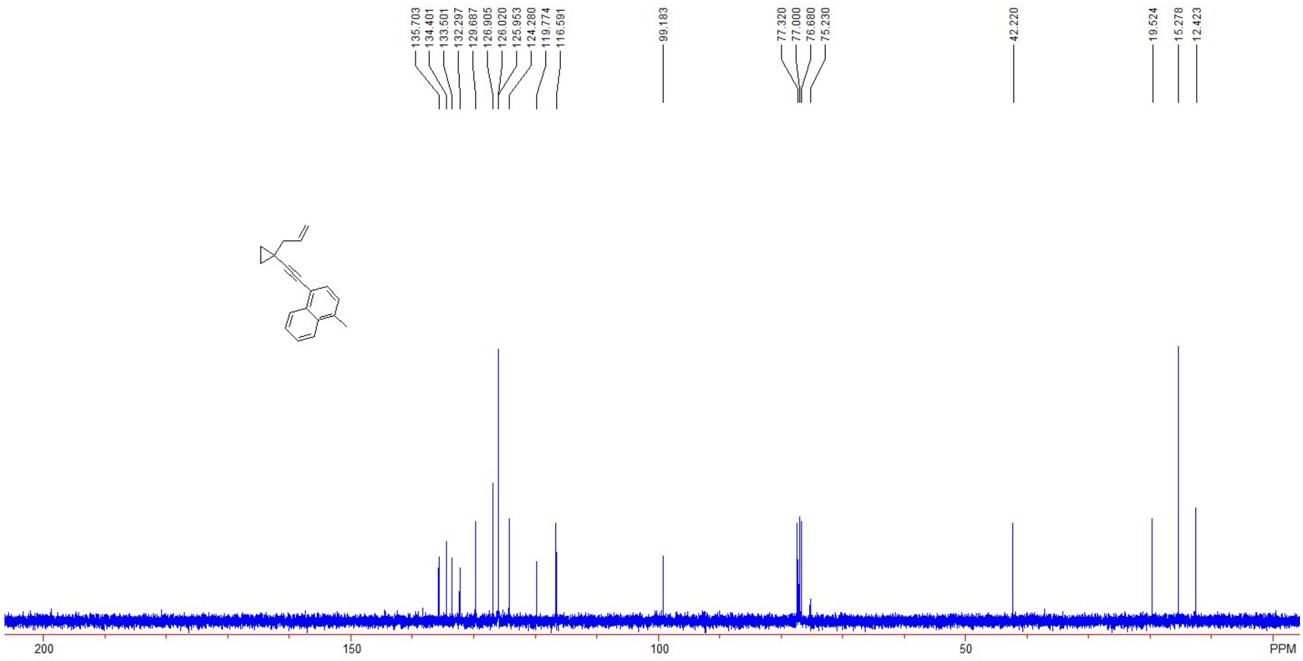

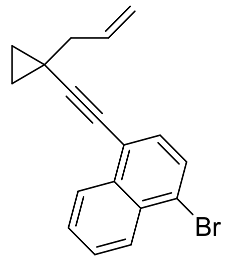

1-((1-Allylcyclopropyl)ethynyl)-4-bromonaphthalene 2h:

1.059 g, yield = 85%. Colorless oil. ^{1}H NMR (400 MHz, $CDCl_3$, TMS): δ 0.81 (dd, J_1 = 6.4 Hz, J_2 = 4.4 Hz, 2H, CH_2), 1.12 (dd, J_1 = 6.4 Hz, J_2 = 4.4 Hz, 2H, CH_2), 2.30 (d, J = 6.8 Hz, 2H, CH_2), 5.14-5.25 (m, 2H, $=CH_2$), 5.99-6.10 (m, 1H, =CH), 7.38 (d, J = 7.6 Hz, 1H, Ar), 7.53-7.59 (m, 2H, Ar), 7.65 (d, J = 7.6 Hz, 1H, Ar), 8.17-8.21 (m, 1H, Ar), 8.29-8.32 (m, 1H, Ar). ^{13}C NMR (100 MHz, $CDCl_3$, TMS): δ 12.4, 15.4, 42.0, 74.5, 101.1, 116.7, 121.7, 122.4, 126.9, 127.2, 127.4, 127.5, 129.3, 129.9, 131.6, 134.5, 135.5. IR (CH_2Cl_2) ν 3077, 3004, 2924, 2220, 1501, 1384, 943, 828, 759 cm^{-1}. MS (%) m/z 310 (M^+, 40.10), 295 (6.88), 269 (17.14), 231 (33.33), 215 (67.47), 202 (59.98), 189 (100.00), 176 (17.17), 163 (19.86), 101 (25.48). HRMS (EI) calcd. for $C_{18}H_{15}Br$: 310.0357, found: 310.0362.

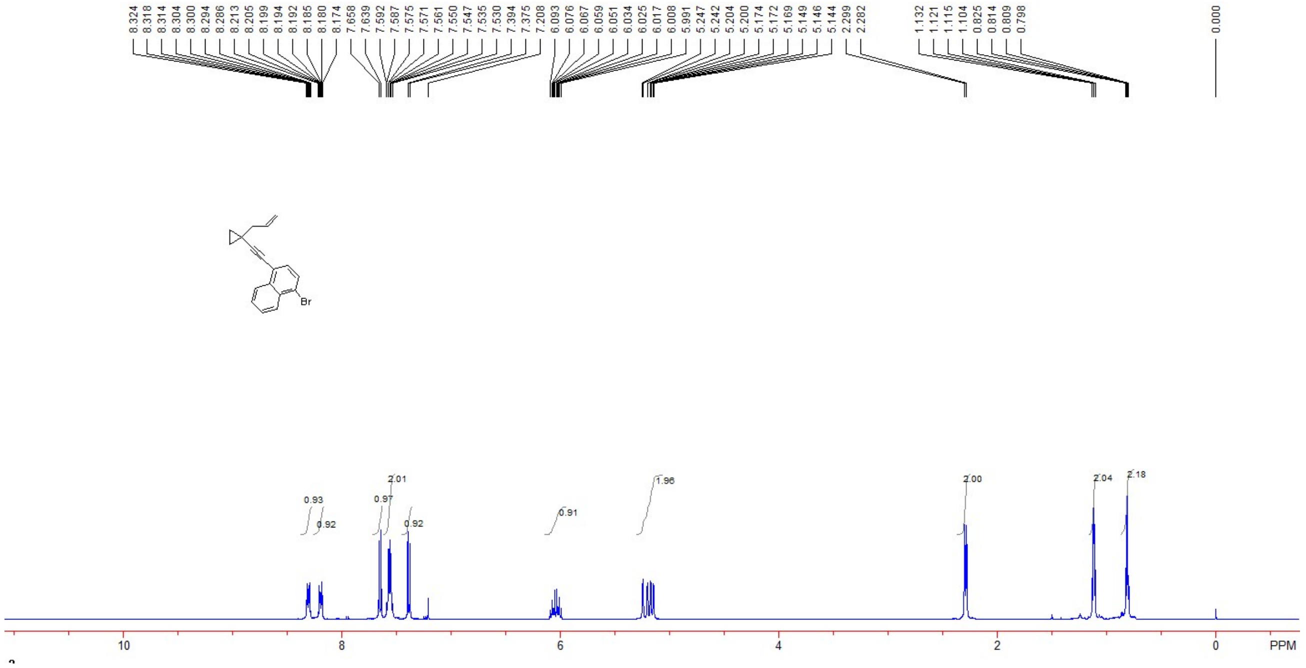

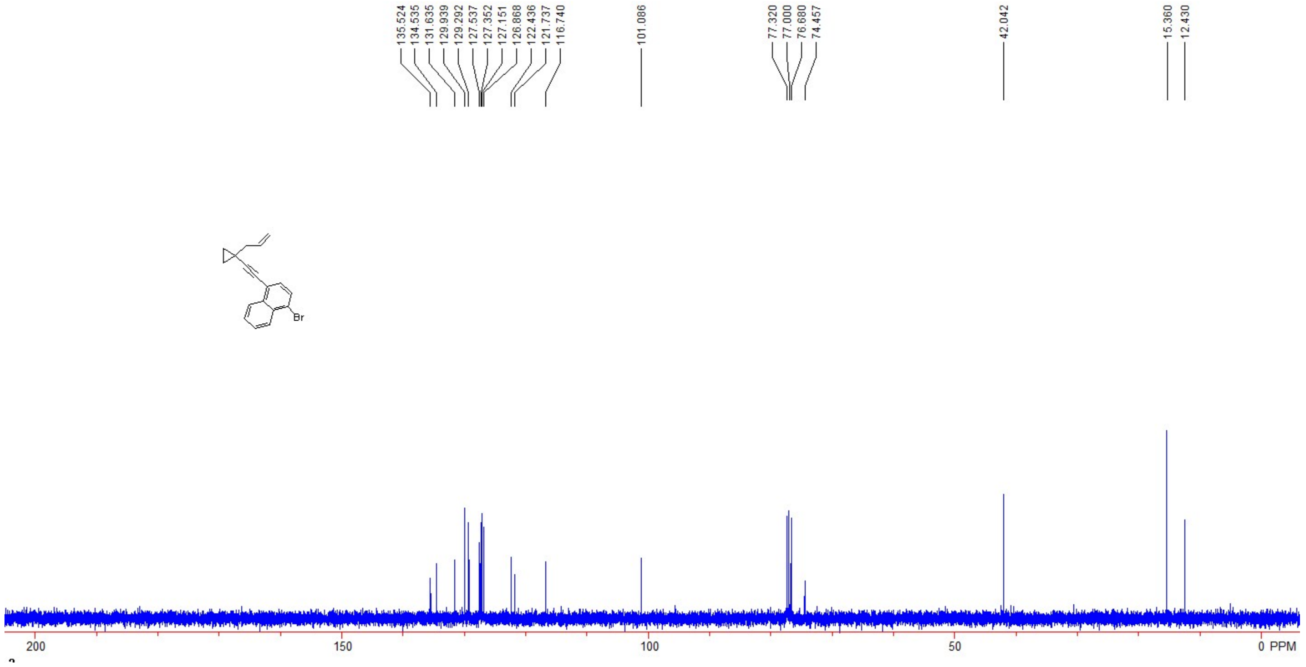

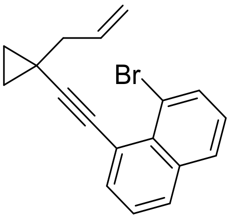

1-((1-Allylcyclopropyl)ethynyl)-8-bromonaphthalene 2i:

0.979 mg, yield = 79%. Colorless oil. ^{1}H NMR (400 MHz, $CDCl_3$, TMS): δ 0.74 (dd, J_1 = 6.8 Hz, J_2 = 4.4 Hz, 2H, CH_2), 1.12 (dd, J_1 = 6.8 Hz, J_2 = 4.4 Hz, 2H, CH_2), 2.28 (d, J = 6.4 Hz, 2H, CH_2), 5.11 (d, J = 10.4 Hz, 1H, =CH_2), 5.18 (dd, J_1 = 17.2 Hz, J_2 = 1.6 Hz, 1H, =CH_2), 6.01-6.08 (m, 1H, =CH), 7.16-7.22 (m, 1H, Ar), 7.30-7.34 (m, 1H, Ar), 7.67-7.72 (m, 3H, Ar), 7.74-7.78 (m, 1H, Ar). ^{13}C NMR (100 MHz, $CDCl_3$, TMS): δ 12.8, 14.3, 41.5, 76.7, 103.2, 116.4, 120.6, 121.6, 125.5, 126.1, 128.9, 129.0, 130.6, 133.6, 135.6, 135.8. IR (CH_2Cl_2) ν 3058, 3003, 2213, 1557, 1195, 815, 755 cm^{-1}. MS (%) m/z 310 (M^+, 28.34), 231 (55.82), 215 (72.96), 202 (66.48), 189 (100.00), 176 (15.88), 163 (19.88), 150 (9.62), 101 (26.83), 88 (15.90). HRMS (EI) calcd. for $C_{18}H_{15}Br$: 310.0357, found: 310.0364.

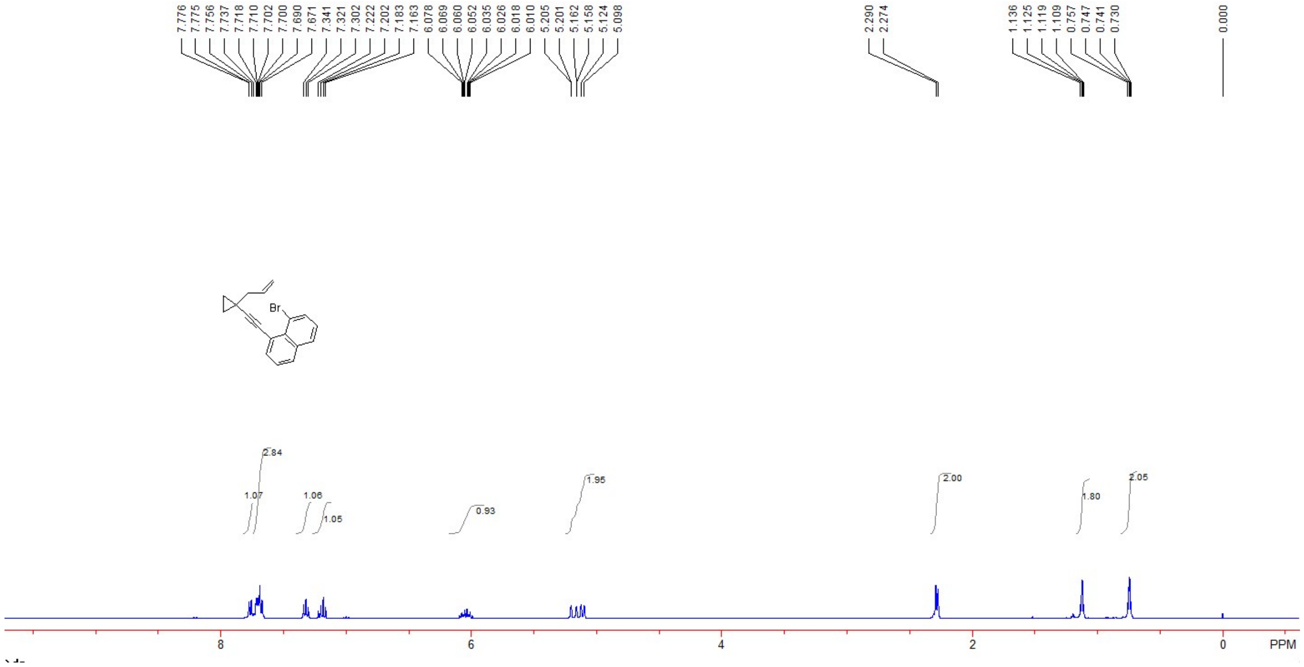

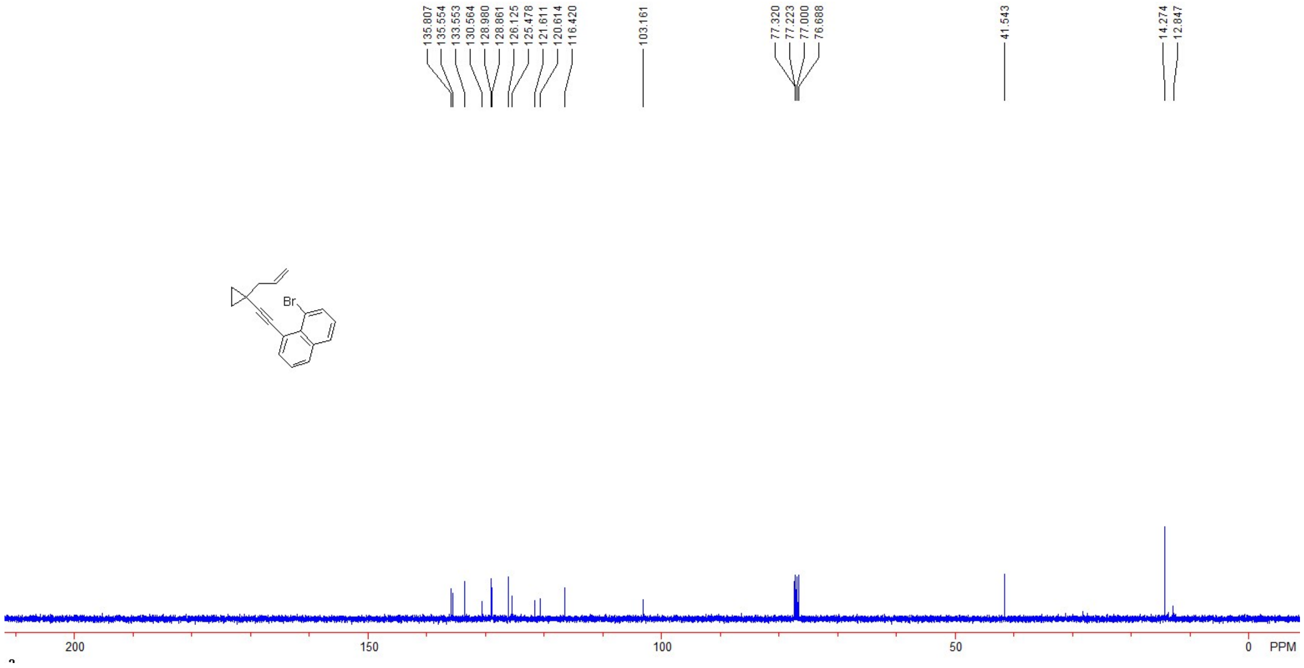

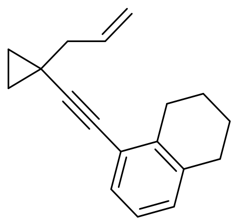

5-((1-Allylcyclopropyl)ethynyl)-1,2,3,4-tetrahydronaphthalene 2j:

0.756 g, yield = 80%. Colorless oil. ^{1}H NMR (400 MHz, $CDCl_3$, TMS): δ 0.73 (dd, J_1 = 6.8 Hz, J_2 = 4.4 Hz, 2H, CH_2), 1.00 (dd, J_1 = 6.8 Hz, J_2 = 4.4 Hz, 2H, CH_2), 1.71-1.82 (m, 4H, CH_2), 2.21 (d, J = 6.8 Hz, 2H, CH_2), 2.71 (dd, J_1 = 6.4 Hz, J_2 = 5.6 Hz, 2H, CH_2), 2.80 (dd, J_1 = 6.4 Hz, J_2 = 6.0 Hz,

2H, CH_2), 5.07-5.17 (m, 2H, =CH_2), 5.93-6.02 (m, 1H, =CH), 6.93-6.99 (m, 2H, Ar), 7.16 (dd, J_1 = 7.2 Hz, J_2 = 1.6 Hz, 1H, Ar). ^{13}C NMR (100 MHz, $CDCl_3$, TMS): δ 12.3, 15.2, 22.8, 23.1, 28.1, 29.7, 42.2, 75.7, 99.3, 116.4, 123.5, 124.8, 128.4, 129.1, 135.6, 137.1, 138.9. IR (CH_2Cl_2) ν 3078, 2926, 2858, 1641, 1451, 996, 913, 774, 709 cm^{-1}. MS (%) m/z 236 (M^+, 64.04), 221 (25.25), 207 (33.92), 193 (43.24), 179 (65.72), 165 (100.00), 152 (46.92), 128 (20.88), 115 (23.11), 89 (10.51). HRMS (EI) calcd. for $C_{18}H_{20}$: 236.1565, found: 236.1560.

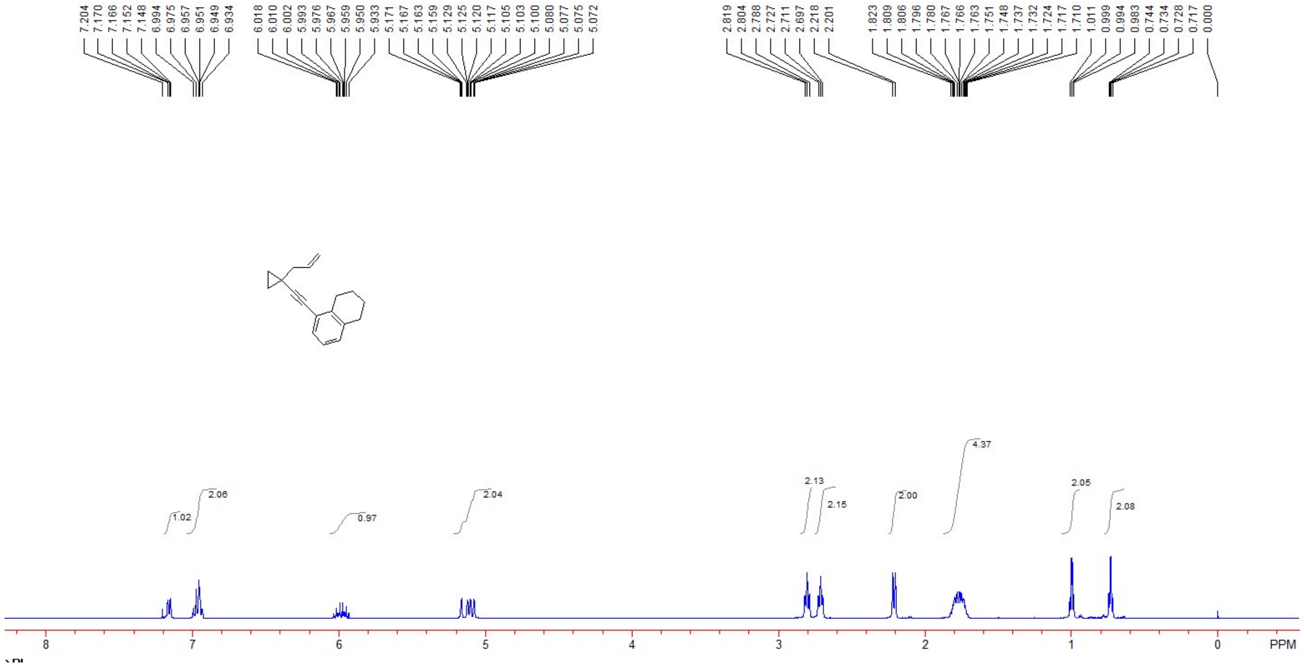

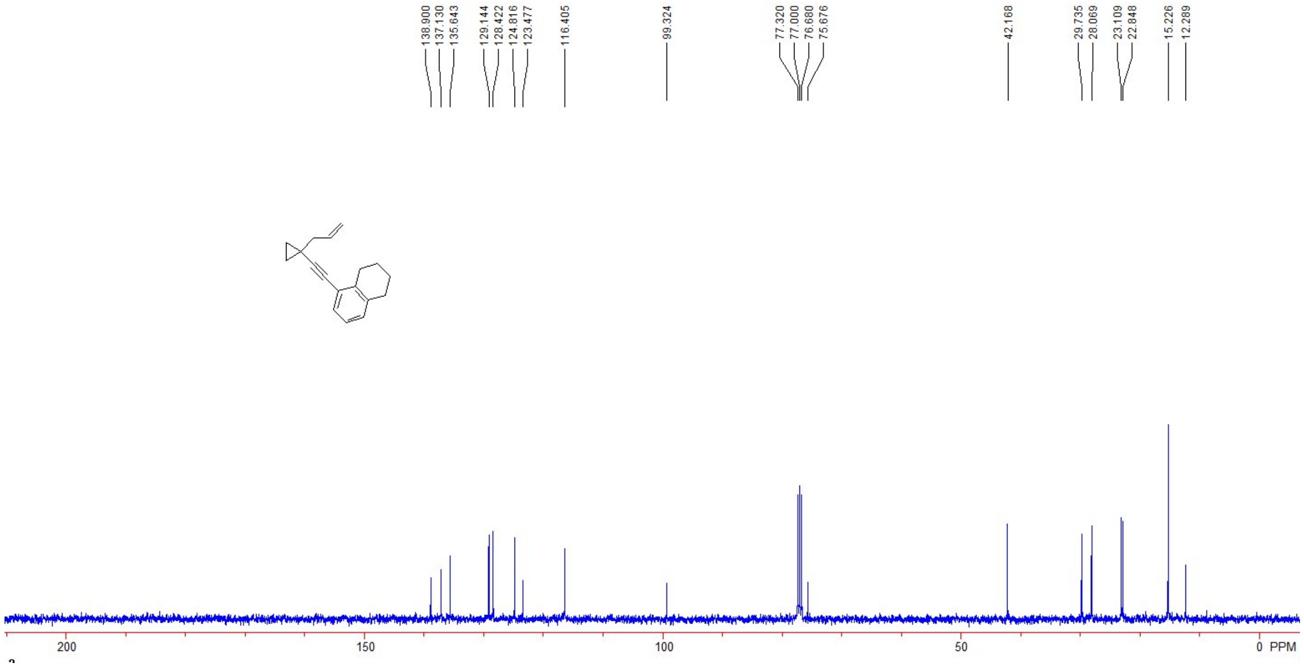

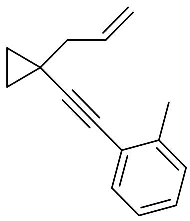

1-((1-Allylcyclopropyl)ethynyl)-2-methylbenzene 2k:

0.651 g, yield = 83%. Colorless oil. ^{1}H NMR (400 MHz, $CDCl_3$, TMS): δ 0.74 (dd, J_1 = 6.4 Hz, J_2 = 4.4 Hz, 2H, CH_2), 1.01 (dd, J_1 = 6.4 Hz, J_2 = 4.4 Hz, 2H, CH_2), 2.22 (d, J = 6.8 Hz, 2H, CH_2), 5.10 (dd, J_1 = 10.0 Hz, J_2 =0.8 Hz, 1H, =CH_2), 5.16 (dd, J_1 = 16.0 Hz, J_2 =0.8 Hz, 1H, =CH_2), 5.94-6.03 (m, 1H, =CH), 7.05-7.09 (m, 1H, Ar), 7.11-7.14 (m, 2H, Ar), 7.32 (d, J = 7.6 Hz, 1H, Ar). ^{13}C NMR (100 MHz, $CDCl_3$, TMS): δ 12.3, 15.2, 20.7, 42.2, 75.8, 98.9, 116.5, 123.6, 125.3, 127.3, 129.2, 131.7, 135.6, 140.0. IR (CH_2Cl_2) ν 3075, 3006, 2921, 2221, 1642, 1486, 914, 753, 715 cm^{-1}. MS (%) m/z 196 (M^+, 57.33), 181 (76.24), 165 (74.38), 153 (76.62), 128 (68.24), 115 (100.00), 105 (26.31), 89 (21.98), 77 (23.41). HRMS (EI) calcd. for $C_{15}H_{16}$: 196.1252, found: 196.1246.

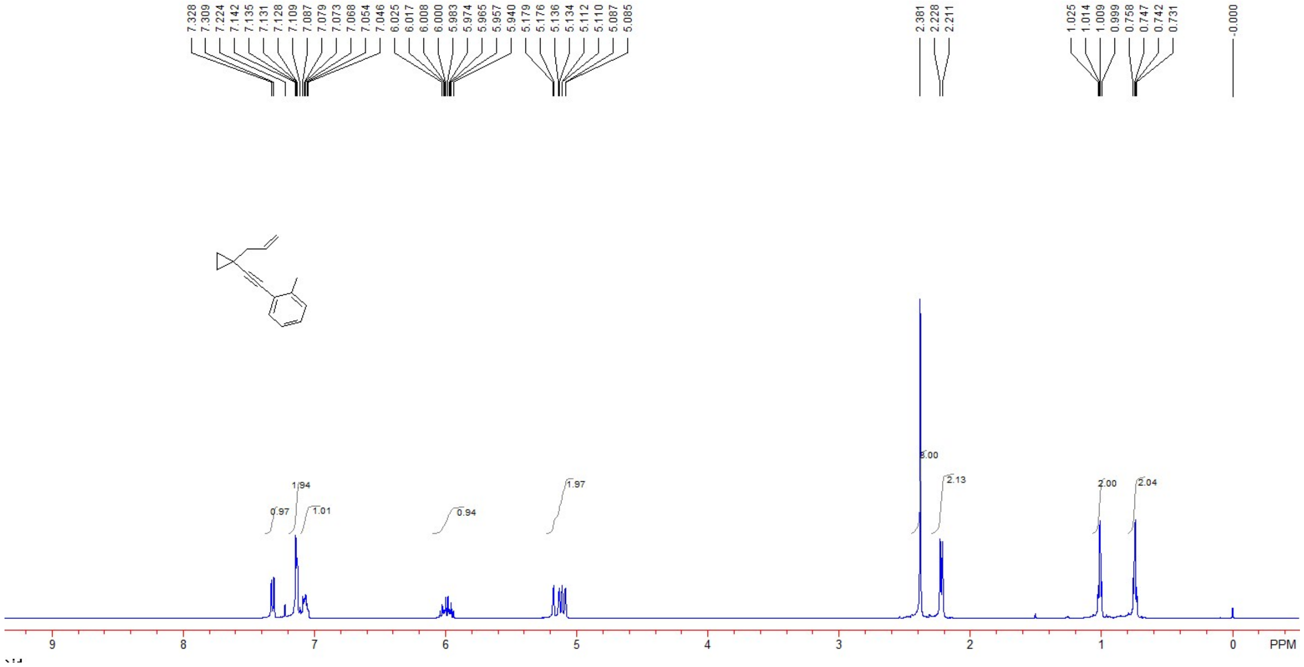

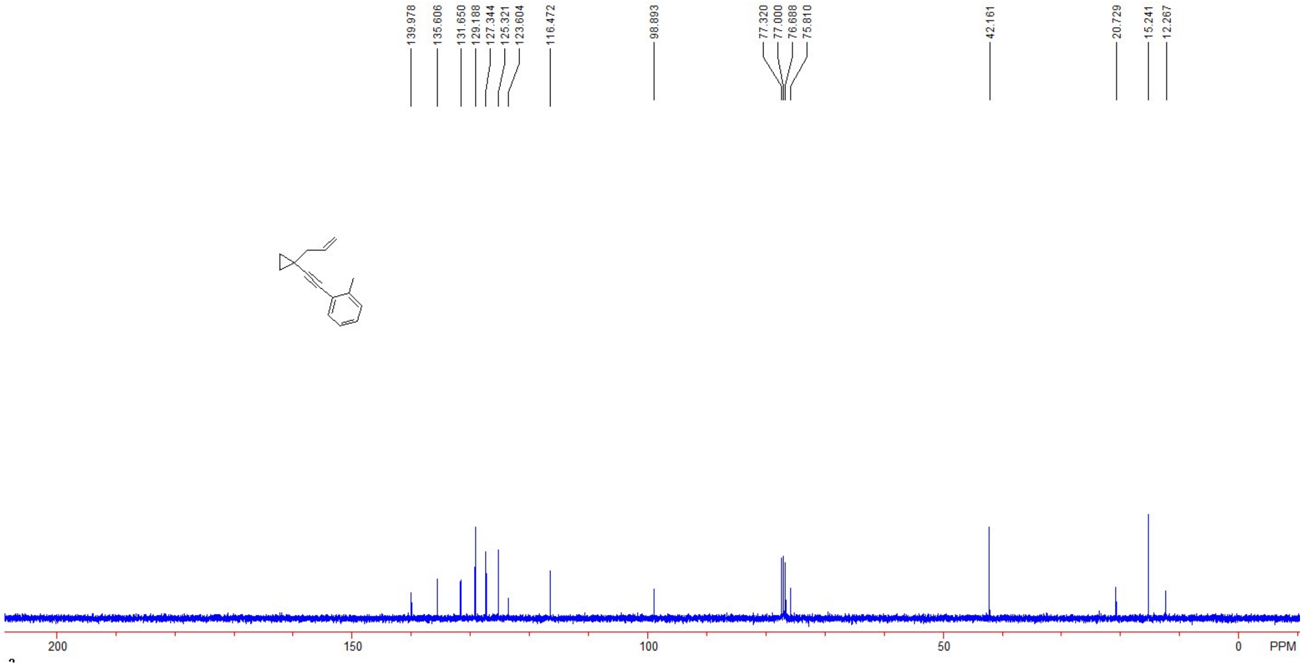

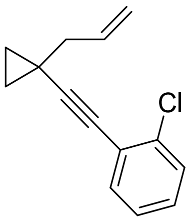

1-((1-allylcyclopropyl)ethynyl)-2-chlorobenzene 2l:

0.653 g, yield = 76%. Colorless oil. ^{1}H NMR (400 MHz, $CDCl_3$, TMS): δ 0.76 (dd, J_1 = 6.4 Hz, J_2 = 4.4 Hz, 2H, CH_2), 1.05 (dd, J_1 = 6.4 Hz, J_2 = 4.4 Hz, 2H, CH_2), 2.23 (d, J = 6.8 Hz, 2H, CH_2), 5.11 (d, J = 10.4 Hz, 1H, =CH_2), 5.16 (d, J = 17.2 Hz, 1H, =CH_2), 5.98-6.06 (m, 1H, =CH), 7.11-7.24 (m, 2H, Ar), 7.32-7.35 (m, 1H, Ar), 7.37-7.41 (m, 1H, Ar). ^{13}C NMR (100 MHz, $CDCl_3$, TMS): δ 12.3, 15.3, 41.8, 73.8, 100.6, 116.6, 123.7, 126.2, 128.3, 129.0, 133.1, 135.3, 135.7. IR (CH_2Cl_2) ν 3076, 3006, 2903, 2225, 1475, 1058, 911, 751, 708 cm^{-1}. MS (%) m/z 216 (M^+, 33.78), 201 (11.85), 181 (92.28), 165 (100.00), 152 (48.07), 139 (68.25), 125 (36.91), 99 (17.76), 89 (21.15), 75 (20.26). HRMS (EI) calcd. for $C_{14}H_{13}Cl$: 216.0706, found: 216.0710.

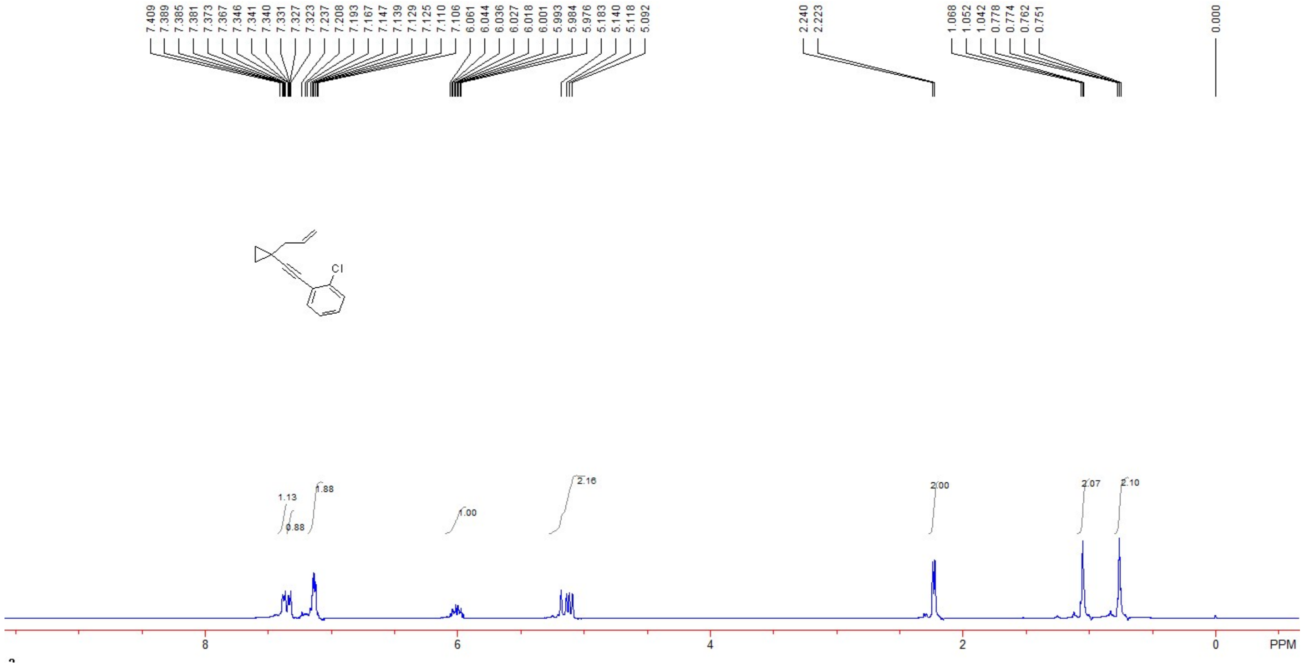

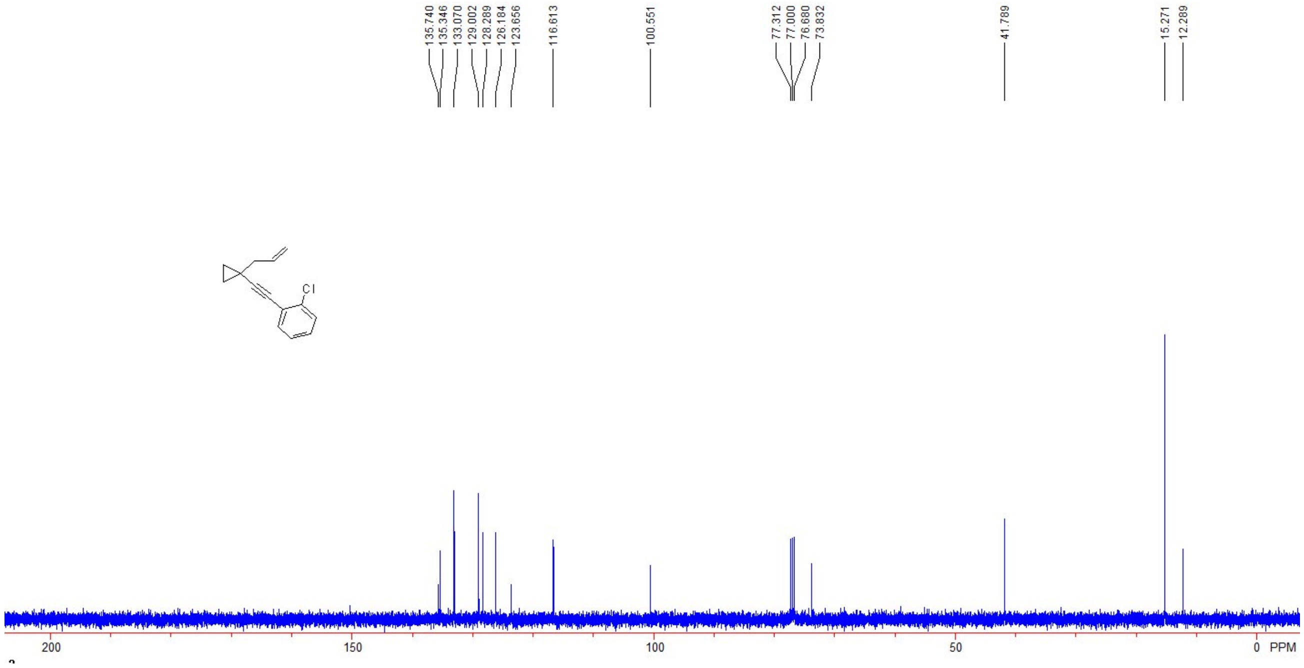

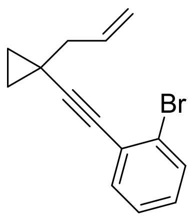

1-((1-Allylcyclopropyl)ethynyl)-2-bromobenzene 2m:

0.444 g, yield = 34%. Colorless oil. ^{1}H NMR (400 MHz, $CDCl_3$, TMS): δ 0.77 (dd, J_1 = 6.4 Hz, J_2 = 4.4 Hz, 2H, CH_2), 1.06 (dd, J_1 = 6.4 Hz, J_2 = 4.4 Hz, 2H, CH_2), 2.24 (d, J = 7.2 Hz, 2H, CH_2), 5.11 (dd, J_1 = 10.0 Hz, J_2 = 1.2 Hz, 1H, =CH_2), 5.17 (dd, J_1 = 17.2 Hz, J_2 = 1.2 Hz, 1H, =CH_2), 5.97-6.07

(m, 1H, =CH), 7.05-7.09 (m, 1H, Ar), 7.16-7.20 (m, 1H, Ar), 7.37 (dd, J_1 = 8.0 Hz, J_2 = 1.2 Hz, 1H, Ar), 7.52 (d, J = 8.0 Hz, 1H, Ar). ^{13}C NMR (100 MHz, $CDCl_3$, TMS): δ 12.3, 15.3, 41.7, 75.6, 100.0, 116.6, 125.5, 125.8, 126.8, 128.4, 132.1, 133.0, 135.4. IR (CH_2Cl_2) ν 3076, 3006, 2903, 2225, 1471, 1433, 1026, 917, 751 cm^{-1}. MS (%) m/z 260 (M^+, 19.29), 232 (7.07), 219 (22.21), 181 (43.74), 166 (100.00), 152 (42.97), 140 (73.28), 115 (23.17), 89 (25.08), 76 (35.62). HRMS (EI) calcd. for $C_{14}H_{13}Br$: 260.0201, found: 260.0203.

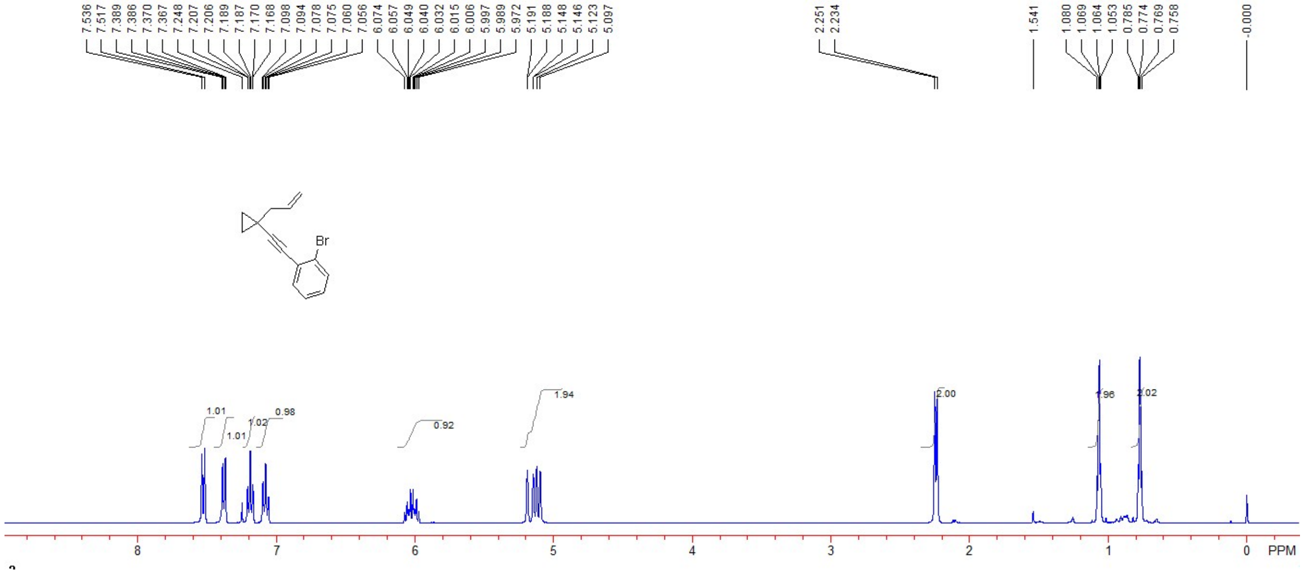

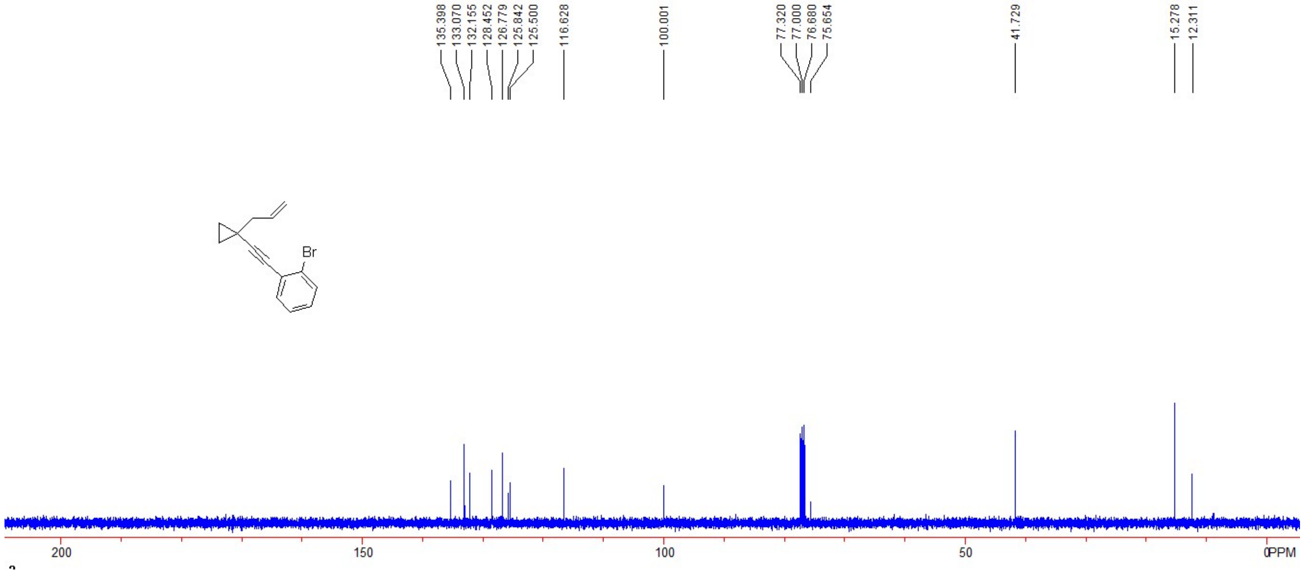

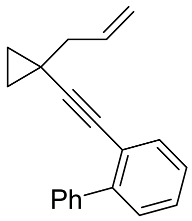

2-((1-Allylcyclopropyl)ethynyl)-1,1'-biphenyl 2n:

0.302 g, yield = 29%. Colorless oil. ^{1}H NMR (400 MHz, $CDCl_3$, TMS): δ 0.62 (dd, J_1 = 6.8 Hz, J_2 = 4.4 Hz, 2H, CH_2), 0.83 (dd, J_1 = 6.8 Hz, J_2 = 4.4 Hz, 2H, CH_2), 2.08 (d, J = 6.8 Hz, 2H, CH_2), 4.99-5.05 (m, 2H, =CH_2), 5.77-5.84 (m, 1H, =CH), 7.20-7.22 (m, 1H, Ar), 7.20-7.22 (m, 1H, Ar), 7.24-7.31 (m, 1H, Ar), 7.32-7.34 (m, 2H, Ar), 7.37-7.41 (m, 2H, Ar), 7.45-7.47 (m, 1H, Ar), 7.55-7.57 (m, 2H, Ar). ^{13}C NMR (100 MHz, $CDCl_3$, TMS): δ 12.2, 14.8, 41.6, 76.4, 97.8, 116.2, 122.2, 126.8, 127.2, 127.6, 127.7, 129.2, 132.8, 135.6, 140.7, 143.6. IR (CH_2Cl_2) ν 3063, 3005, 2922, 2222, 1641, 1449, 915, 775, 698 cm^{-1}. MS (%) m/z 258 (M^+, 20.11), 243 (38.44), 229 (61.65), 215 (91.03), 202 (100.00), 189 (23.98), 178 (16.67), 165 (58.67), 93 (38.62), 77 (10.51). HRMS (EI) calcd. for $C_{20}H_{18}$: 258.1409, found: 258.1406.

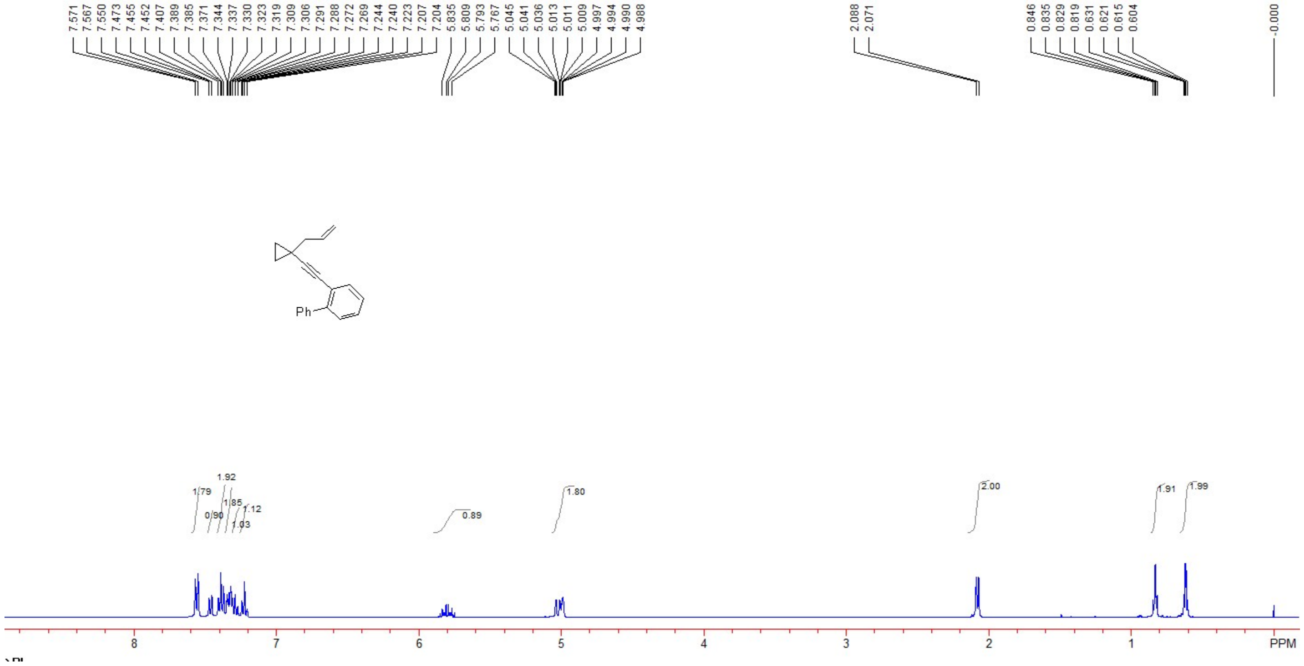

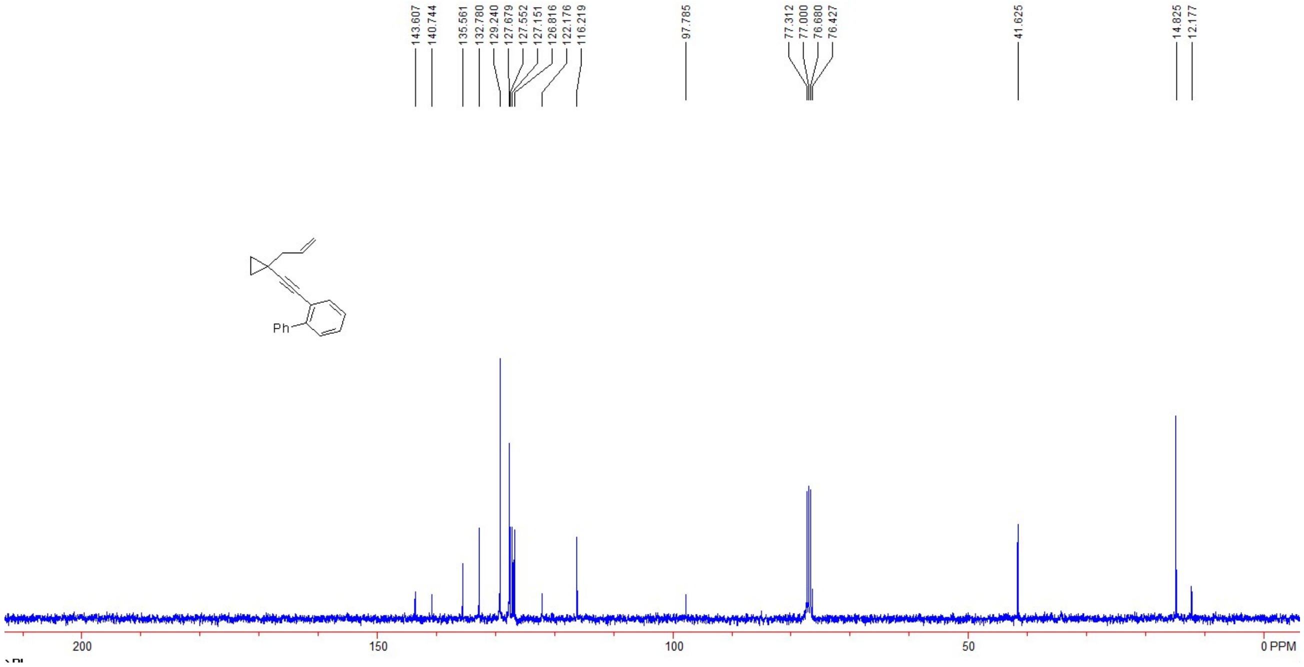

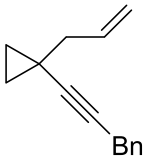

(3-(1-Allylcyclopropyl)prop-2-yn-1-yl)benzene 2o:

0.863 g, yield = 59%. Colorless oil. ^{1}H NMR (400 MHz, $CDCl_3$, TMS): δ 0.61-0.63 (m, 2H, CH_2), 0.89 (dd, J_1 = 5.6 Hz, J_2 = 4.4 Hz, 2H, CH_2), 2.14 (d, J = 6.8 Hz, 2H, CH_2), 3.56 (s, 2H, CH_2), 5.07-5.14 (m, 2H, =CH_2), 5.90-5.99 (m, 1H, =CH), 7.19-7.23 (m, 1H, Ar), 7.24-7.34 (m, 4H, Ar). ^{13}C NMR (100 MHz, $CDCl_3$, TMS): δ 11.6, 14.56, 14.57, 25.0, 42.2, 74.0, 87.1, 116.3, 126.3, 127.8, 128.3, 135.8, 137.5. IR (CH_2Cl_2) ν 3064, 3005, 2921, 2201, 1689, 1275, 913, 750, 696 cm^{-1}. MS (%) m/z 196 (M^+, 4.07), 195 (15.33), 181 (49.65), 165 (64.78), 153 (70.59), 128 (58.23), 115 (100.00), 105 (56.39), 91 (77.47), 77 (69.49). HRMS (EI) calcd. for $C_{15}H_{16}$: 196.1252, found: 196.1257.

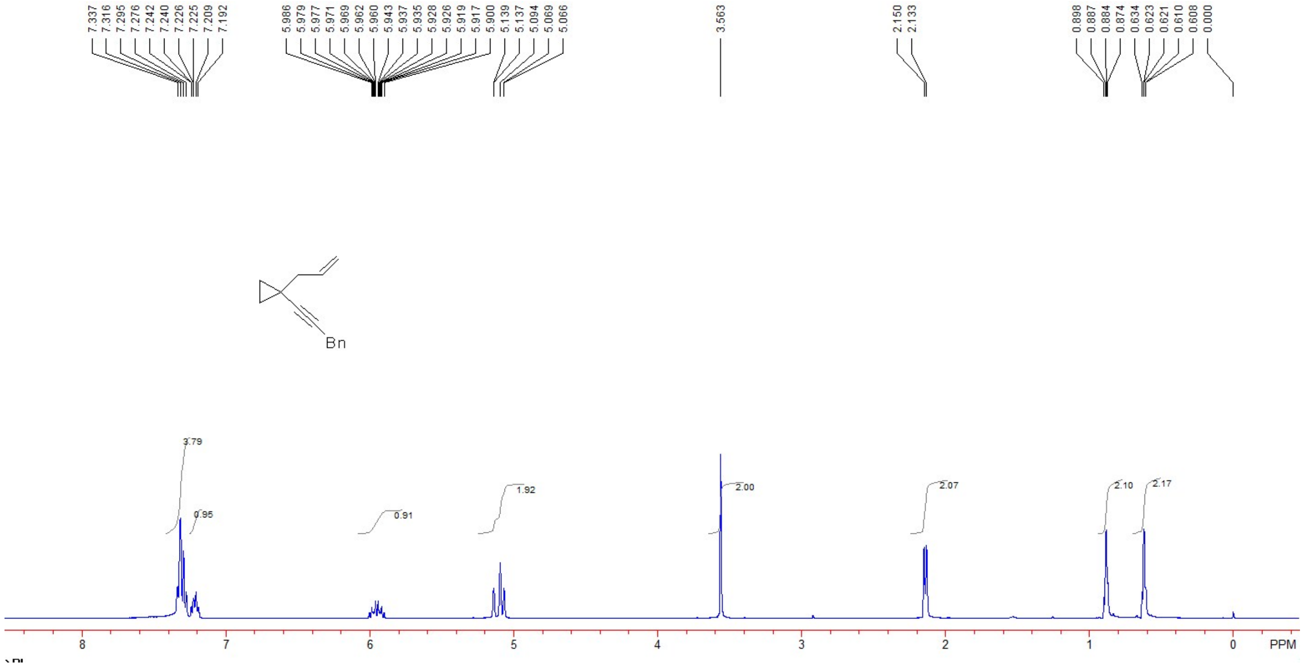

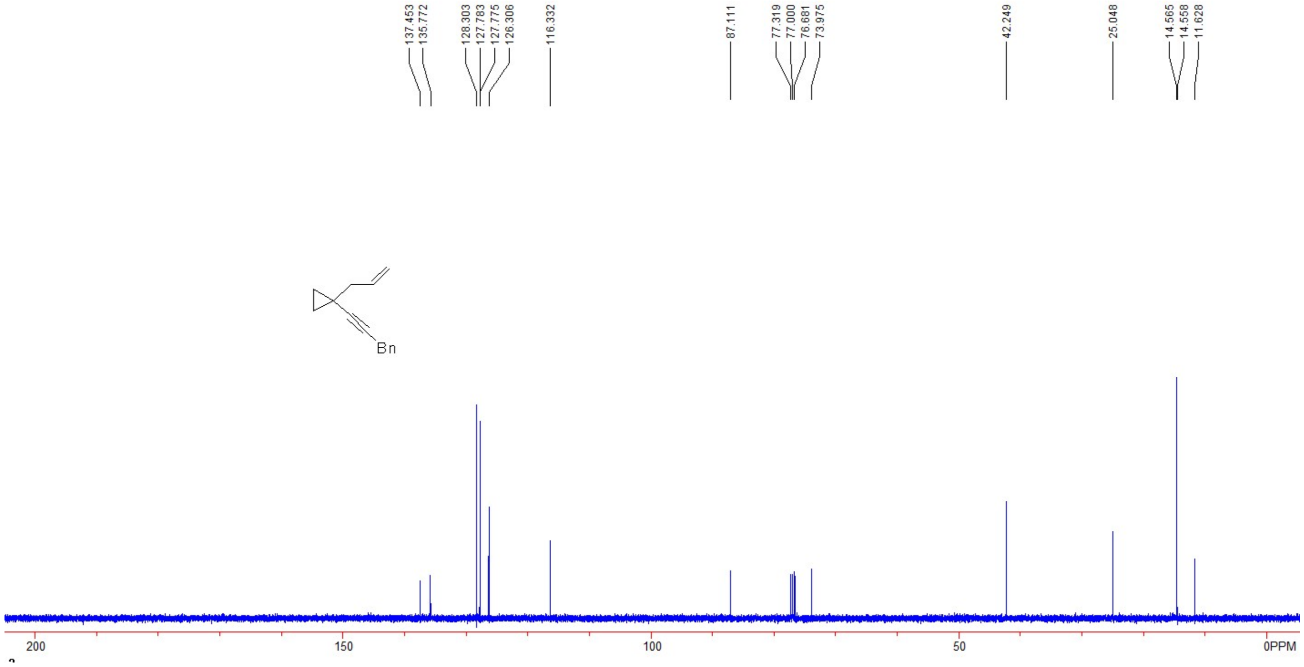

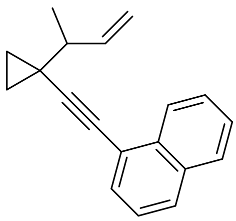

1-((1-(But-3-en-2-yl)cyclopropyl)ethynyl)naphthalene 2p:

103 mg, yield = 8%. Colorless oil. ^{1}H NMR (400 MHz, $CDCl_3$, TMS): δ 0.78-0.81 (m, 2H, CH_2), 1.08-1.14 (m, 2H, CH_2), 1.34 (d, J = 6.8 Hz, 3H, CH_3), 1.60-1.67 (m, 1H, Ar), 5.08-5.16 (m, 2H, $=CH_2$), 6.04-6.12 (m, 1H, =CH), 7.33-7.37 (m, 1H, Ar), 7.45-7.47 (m, 1H, Ar), 7.48-7.51 (m, 1H,

Ar), 7.53-7.55 (m, 1H, Ar), 7.57-7.59 (m, 1H, Ar), 7.72-7.80 (m, 1H, Ar), 8.32 (d, *J* = 8.4 Hz, 1H, Ar). ^{13}C NMR (100 MHz, $CDCl_3$, TMS): δ 15.2, 15.5, 18.0, 18.3, 45.2, 75.9, 98.4, 114.1, 121.7, 125.2, 126.1, 126.3, 126.4, 127.7, 128.2, 130.0, 133.1, 133.6, 141.4. IR (CH_2Cl_2) ν 3058, 2964, 2926, 2218, 1638, 1399, 913, 797, 771 cm^{-1}. MS (%) m/z 246 (M^+, 93.41), 231 (54.61), 215 (51.62), 202 (57.15), 189 (100.00), 165 (44.53), 152 (24.59), 101 (14.80), 94 (11.57). HRMS (EI) calcd. for $C_{19}H_{18}$: 246.1409, found: 246.1415.

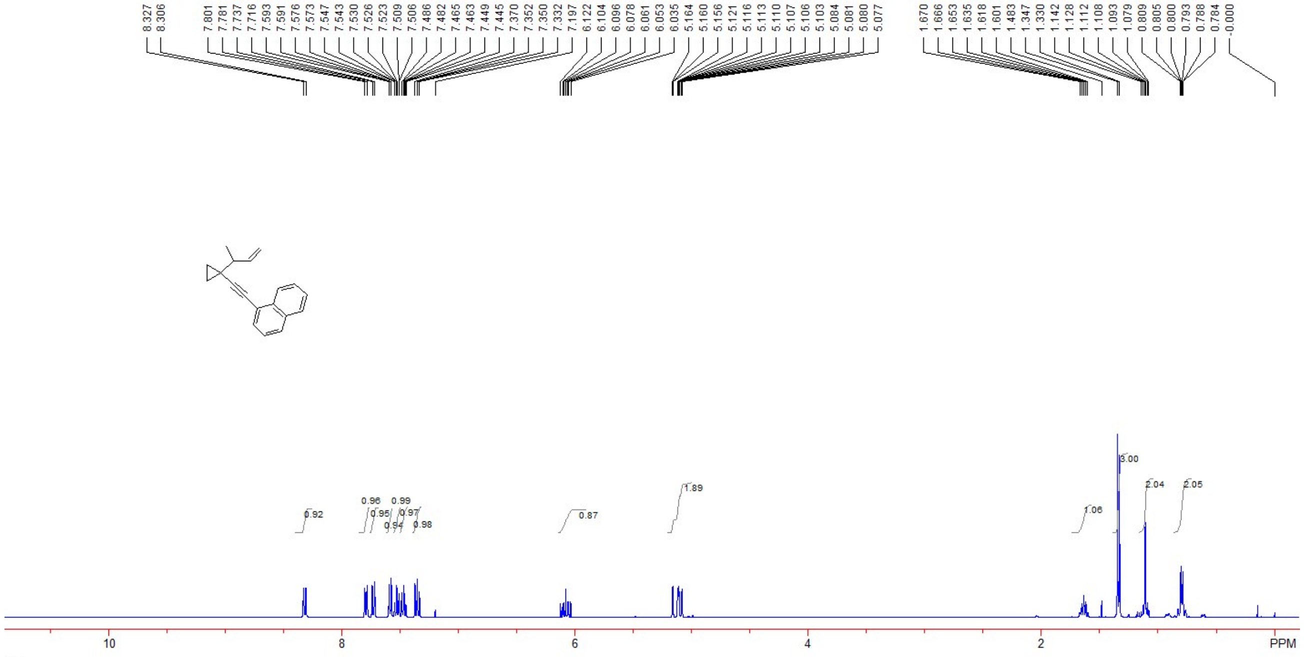

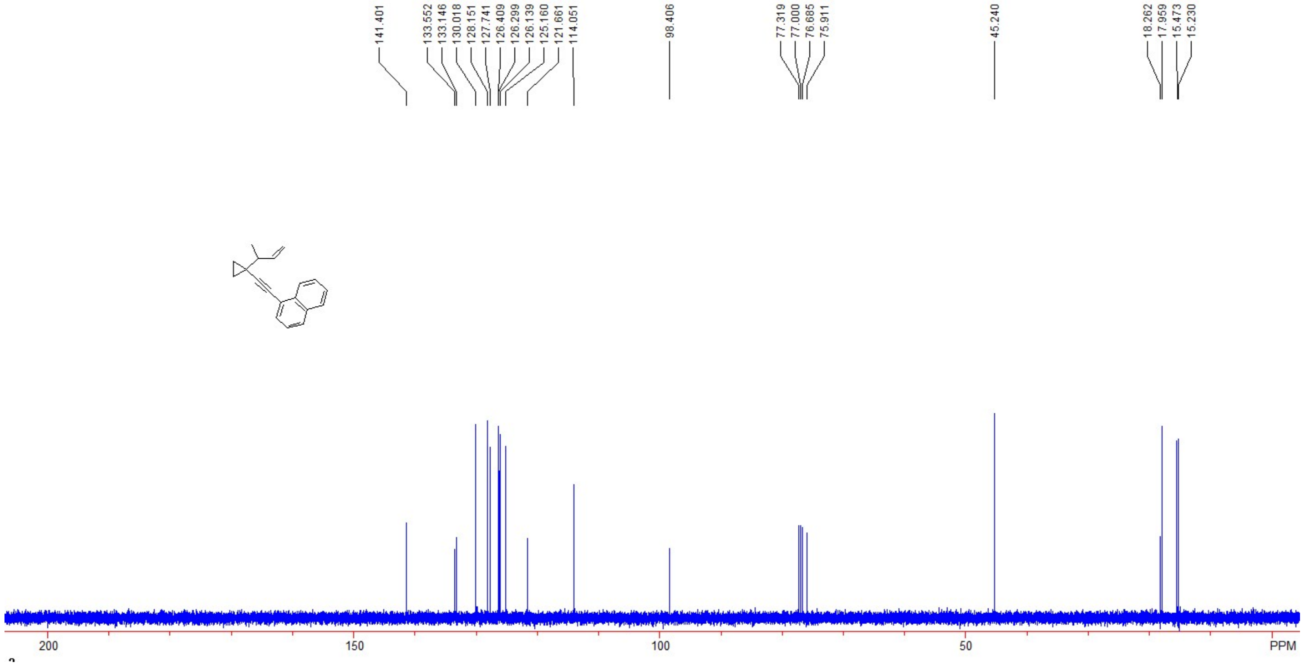

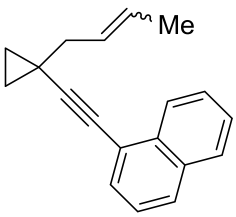

1-((1-(But-2-en-1-yl)cyclopropyl)ethynyl)naphthalene 2q:

563 mg, yield = 46%. Colorless oil (mixtures of two isomers, E/Z = 4.4/1). ^{1}H NMR (400 MHz, $CDCl_3$, TMS): δ 0.78-0.83 (m, 2H, CH_2), 1.08-1.11 (m, 2H, CH_2), 1.68 (d, J = 5.6 Hz, 0.51H, CH_3), 1.75 (d, J = 5.6 Hz, 2.26H, CH_3), 2.24 (d, J = 5.6 Hz, 1.58H, CH_2), 2.34 (d, J = 5.6 Hz, 0.36H, CH_2), 5.61-5.73 (m, 2H, =CH), 7.34-7.38 (m, 1H, Ar), 7.46-7.53 (m, 2H, Ar), 7.55-7.58 (m, 1H, Ar), 7.74 (d, J = 8.0 Hz, 1H, Ar), 7.80 (d, J = 8.0 Hz, 1H, Ar), 8.30-8.34 (m, 1H, Ar). ^{13}C NMR (100 MHz, $CDCl_3$, TMS): δ 12.9, 13.1, 15.19, 15.22, 18.0, 35.0, 41.0, 74.7, 75.0, 100.3, 100.4, 121.7, 125.2, 125.8, 126.1, 126.30, 126.35, 126.39, 127.35, 127.71, 127.73, 128.13, 129.80, 129.87, 133.1, 133.6. IR (CH_2Cl_2) ν 3055, 2916, 2850, 2216, 1585, 1398, 565, 797, 771 cm^{-1}. MS (%) m/z 246 (M^+, 84.66), 231 (68.61), 215 (56.98), 202 (64.58), 189 (100.00), 176 (29.70), 165 (38.94), 152 (23.62), 115 (7.87), 101 (10.84). HRMS (EI) calcd. for $C_{19}H_{18}$: 246.1409, found: 246.1402.

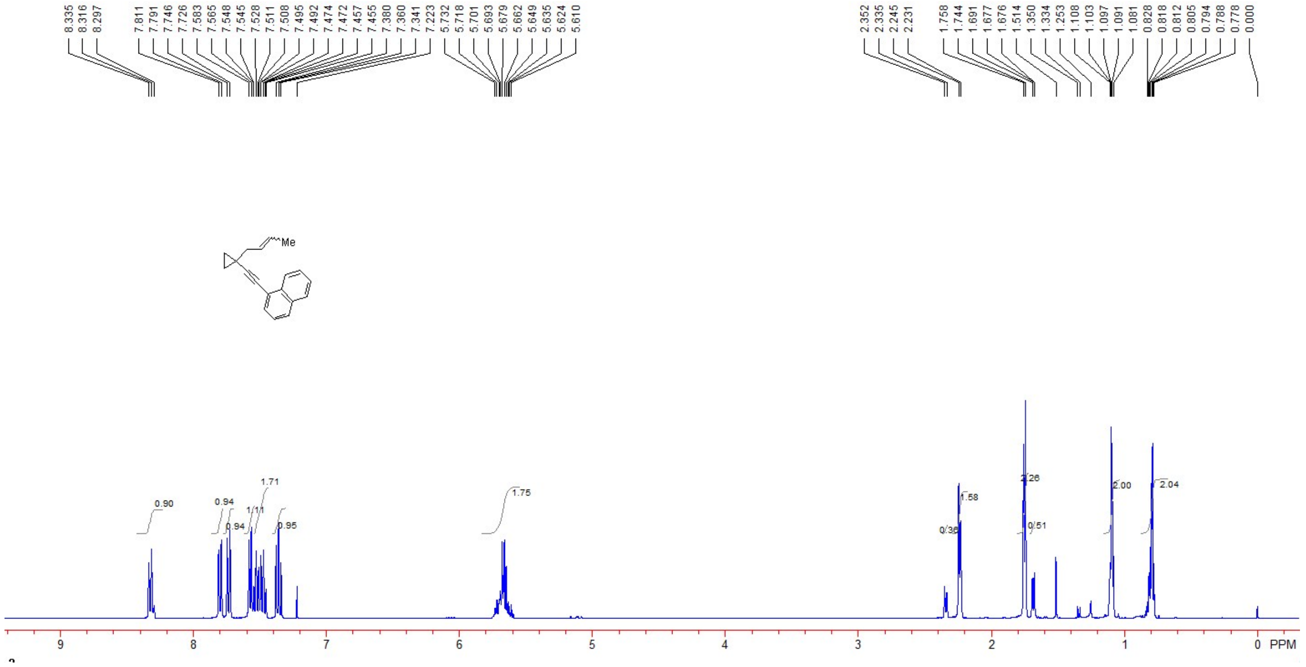

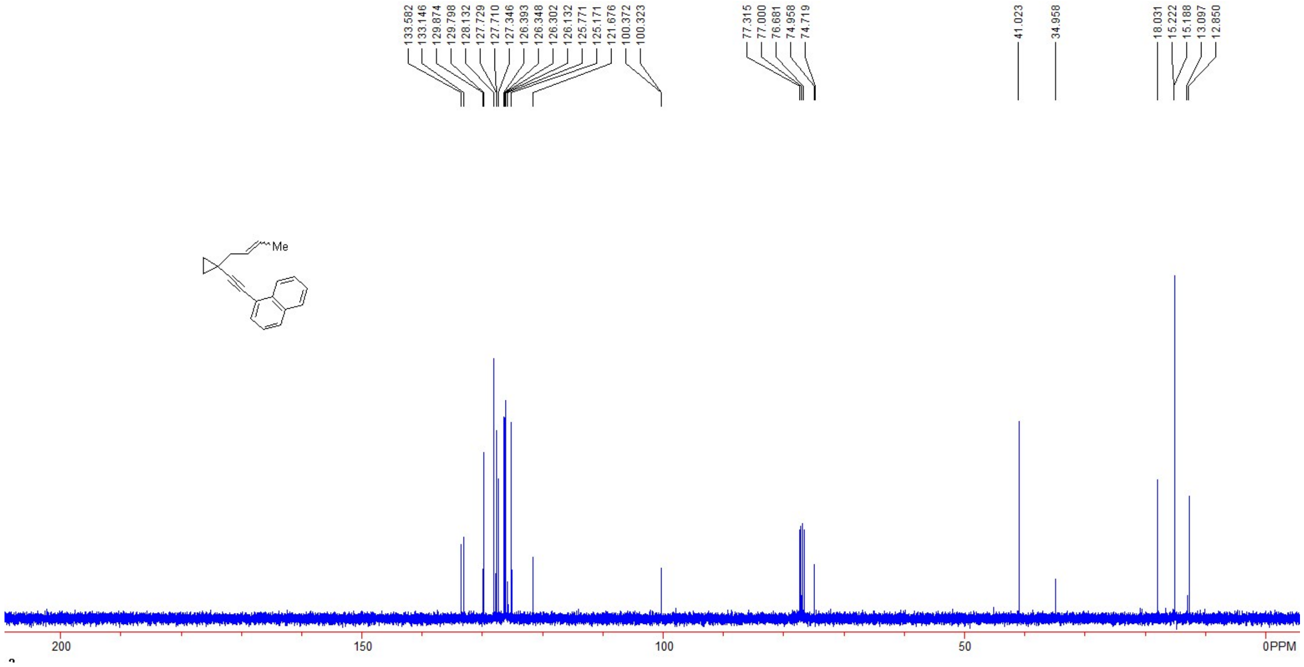

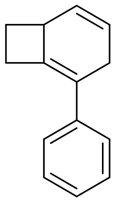

2-Phenylbicyclo[4.2.0]octa-1,4-diene 3a:

34 mg, yield = 92%. Colorless oil. ^{1}H NMR (400 MHz, $CDCl_3$, TMS): δ 1.79-1.86 (m, 1H, CH_2), 2.26-2.32 (m, 1H, CH_2), 2.66-2.82 (m, 2H, CH_2), 3.07-3.15 (m, 1H, CH_2), 3.22-2.29 (m, 1H, CH_2), 3.54-3.55 (m, 1H, CH), 5.83-5.90 (m, 1H, =CH), 5.91-5.93 (m, 1H, =CH), 7.18-7.21 (m, 1H, Ar), 7.25-7.33 (m, 4H, Ar). ^{13}C NMR (100 MHz, $CDCl_3$, TMS): δ 27.4, 28.6, 32.6, 44.0, 123.2, 126.2, 126.7, 128.2, 128.3, 138.9, 140.1. IR (CH_2Cl_2) ν 3024, 2938, 2852, 1683, 1495, 908, 761, 694, 676 cm^{-1}. MS (%) m/z 182 (M^+, 45.82), 167 (55.47), 154 (100.00), 128 (18.04), 115 (23.54), 91 (32.38), 77 (27.67), 63 (12.20). HRMS (EI) calcd. for $C_{14}H_{14}$: 182.1096, found: 182.1101.

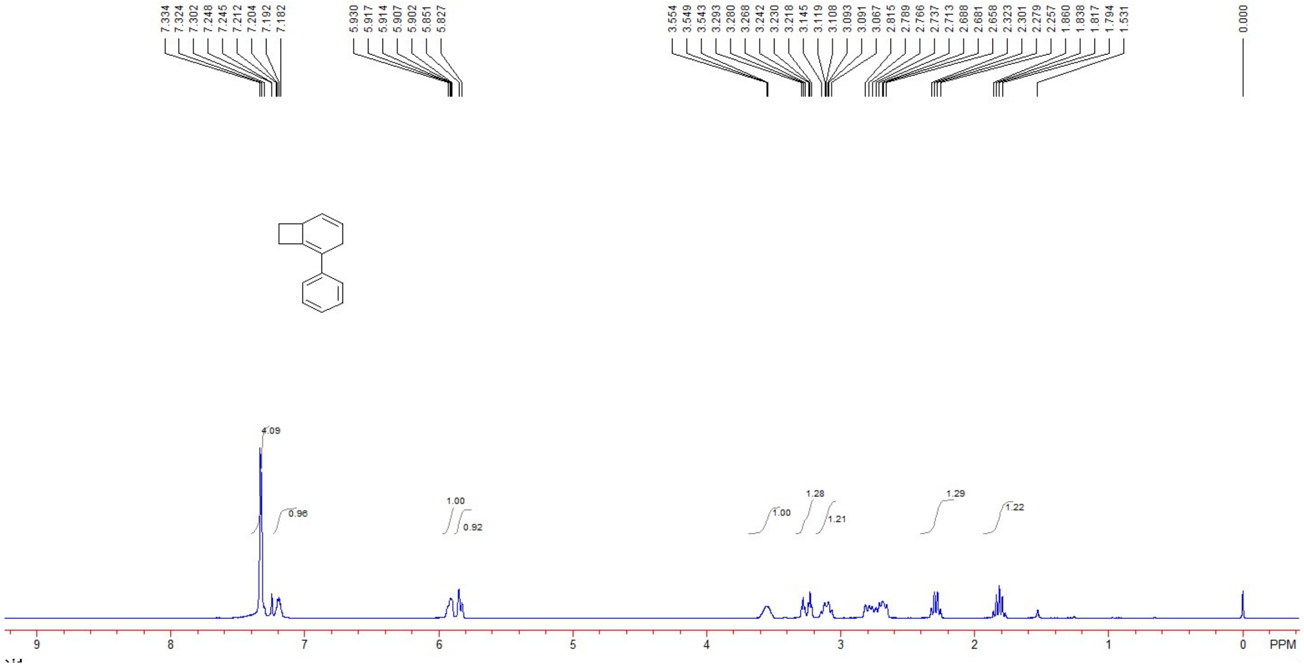

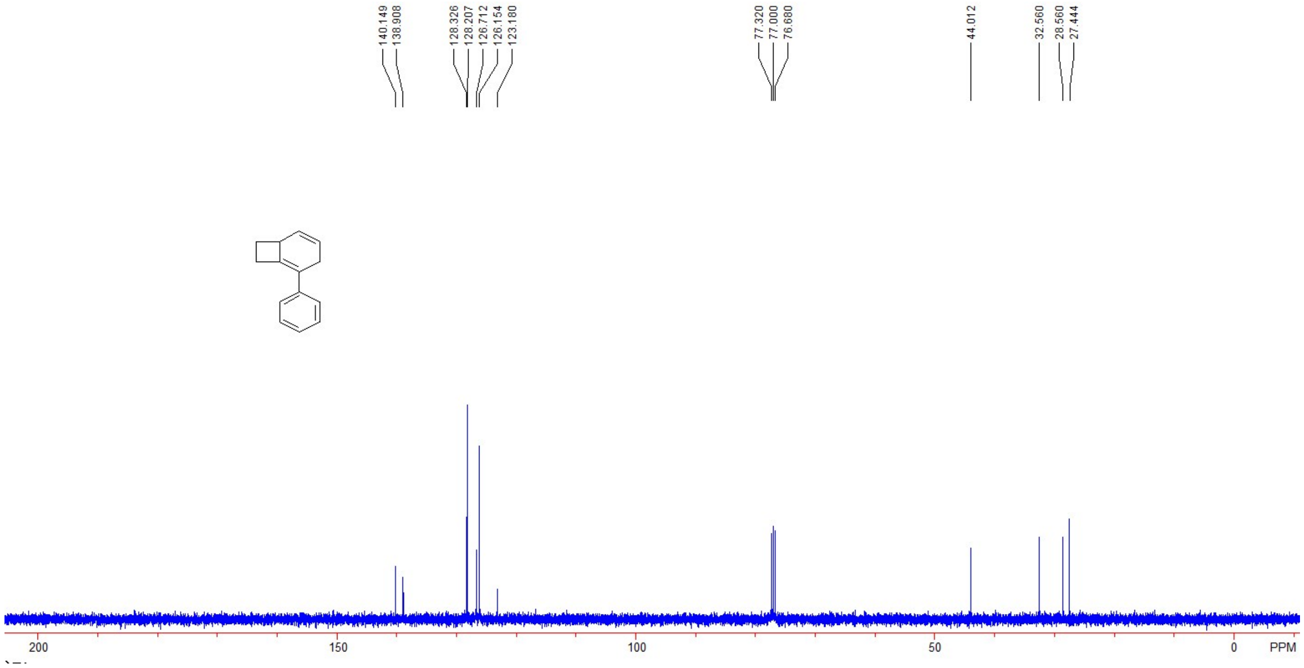

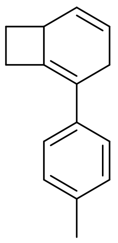

2-(*p*-Tolyl)bicyclo[4.2.0]octa-1,4-diene 3b:

36 mg, yield = 90%. Colorless oil. ^{1}H NMR (400 MHz, $CDCl_3$, TMS): δ 1.76-1.85 (m, 1H, CH_2), 2.26-2.31 (m, 1H, CH_2), 2.33 (s, 3H, CH_3), 2.64-2.70 (m, 2H, CH_2), 2.72-2.80 (m, 1H, CH_2), 3.05-3.13 (m, 1H, CH_2), 3.20-2.27 (m, 1H, CH_2), 3.50-3.57 (m, 1H, CH), 5.82-5.84 (m, 1H, =CH), 5.89-

5.92 (m, 1H, =CH), 7.13 (d, J = 8.0 Hz, 2H, Ar), 7.23 (d, J = 8.0 Hz, 2H, Ar). ^{13}C NMR (100 MHz, $CDCl_3$, TMS): δ 21.1, 27.5, 28.6, 32.5, 44.0, 123.0, 126.1, 126.8, 128.4, 128.9, 135.8, 136.0, 139.2. IR (CH_2Cl_2) ν 3024, 2921, 1514, 1468, 1034, 814, 785, 708 cm^{-1}. MS (%) m/z 196 (M^+, 49.29), 181 (100.00), 165 (54.84), 152 (20.96), 128 (13.63), 115 (19.06), 105 (22.60), 89 (20.62), 77 (14.03). HRMS (EI) calcd. for $C_{15}H_{16}$: 196.1252, found: 196.1260.

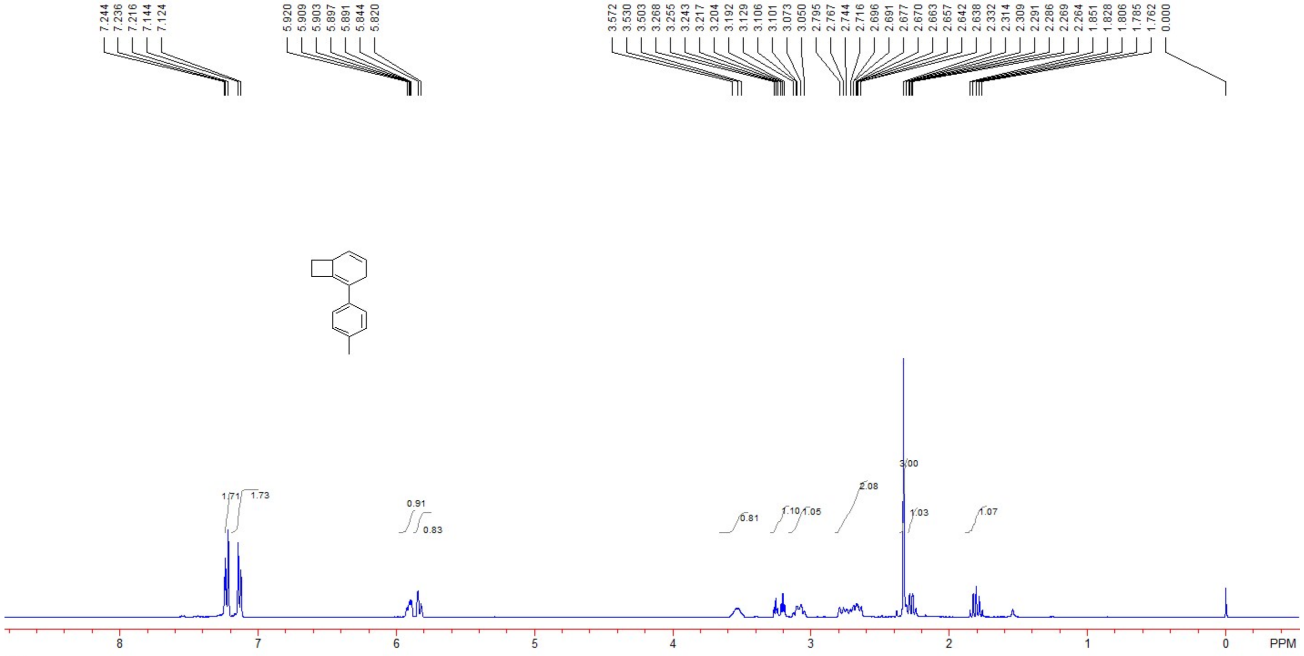

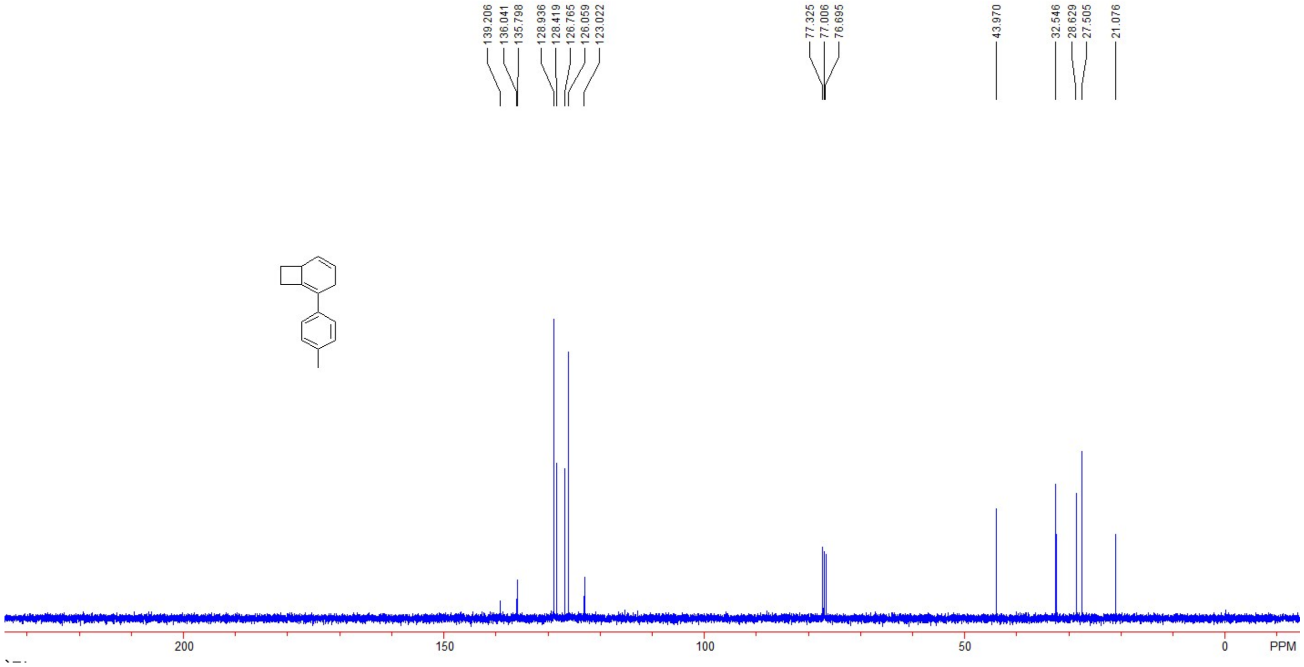

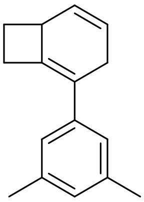

2-(3,5-Dimethylphenyl)bicyclo[4.2.0]octa-1,4-diene 3c:

38 mg, yield = 91%. Colorless oil. ^{1}H NMR (400 MHz, $CDCl_3$, TMS): δ 1.76-1.81 (m, 1H, CH_2), 2.25-2.30 (m, 1H, CH_2), 2.36 (s, 6H, CH_3), 2.63-2.78 (m, 2H, CH_2), 3.06-3.09 (m, 1H, CH_2), 3.19-2.26 (m, 1H, CH_2), 3.51-3.53 (m, 1H, CH), 5.80-5.83 (m, 1H, =CH), 5.87-5.91 (m, 1H, =CH), 6.84 (s, 1H, Ar), 6.94 (s, 2H, Ar). ^{13}C NMR (100 MHz, $CDCl_3$, TMS): δ 21.4, 27.6, 28.6, 32.6, 44.0, 123.3, 124.1, 126.8, 127.9, 128.3, 137.6, 138.9, 139.1. IR (CH_2Cl_2) ν 3023, 2939, 2857, 1599, 1463, 845, 787, 709 cm^{-1}. MS (%) m/z 210 (M^+, 34.63), 195 (100.00), 182 (57.18), 167 (54.10), 152 (17.91), 128 (14.69), 115 (18.93), 89 (27.48), 77 (19.97). HRMS (EI) calcd. for $C_{16}H_{18}$: 210.1409, found: 210.1414.

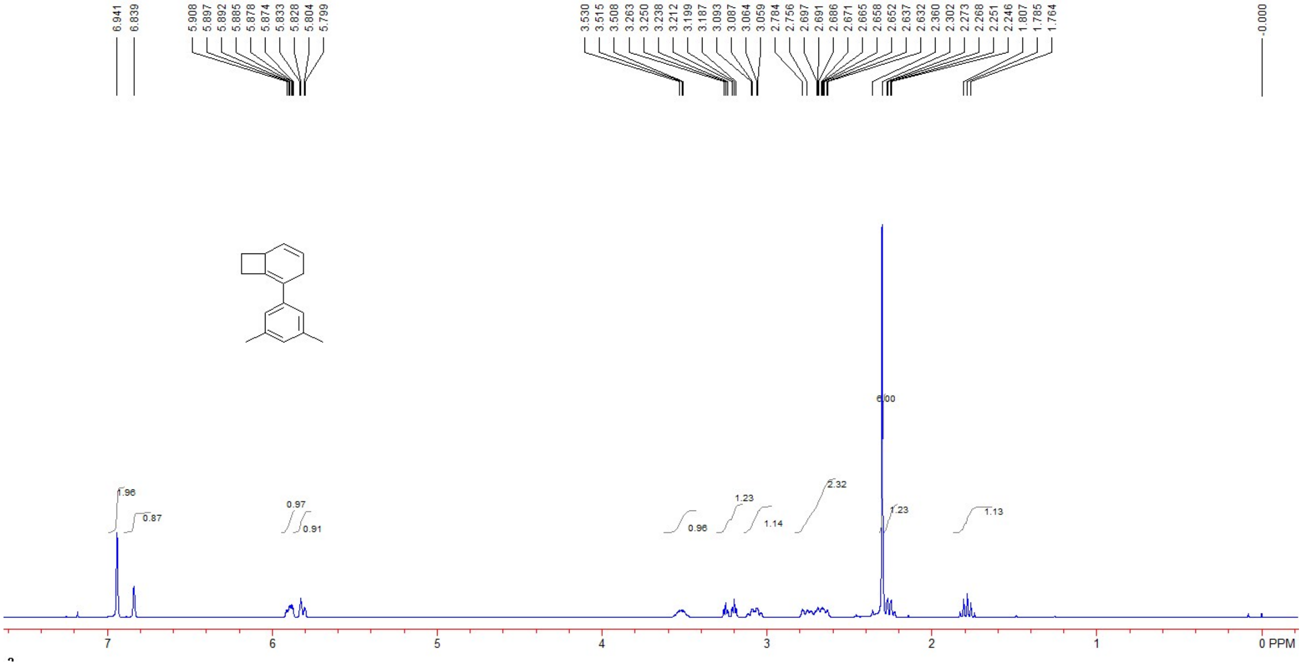

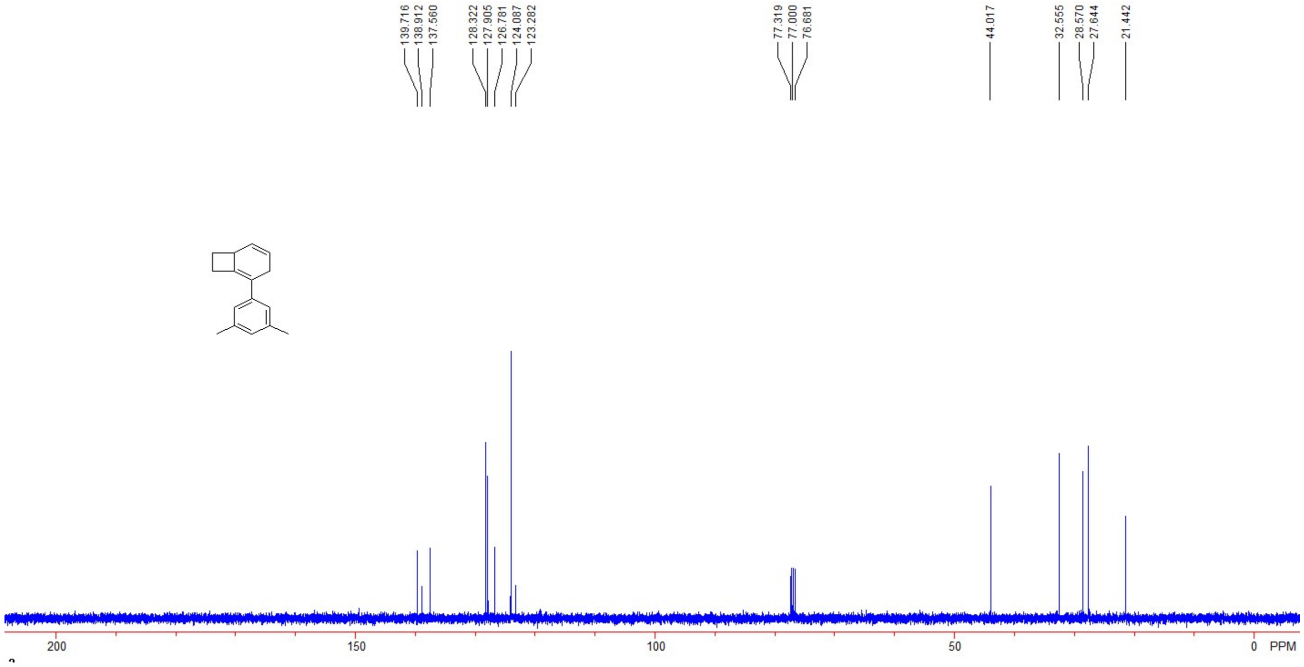

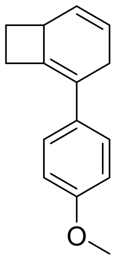

2-(4-Methoxyphenyl)bicyclo[4.2.0]octa-1,4-diene 3d:

40 mg, yield = 93%. White solid, mp: 85-88 °C. ^{1}H NMR (400 MHz, $CDCl_3$, TMS): δ 1.76-1.85 (m, 1H, CH_2), 2.23-2.31 (m, 1H, CH_2), 2.62-2.68 (m, 1H, CH_2), 2.72-2.78 (m, 1H, CH_2), 3.04-3.12 (m, 1H, CH_2), 3.16-2.24 (m, 1H, CH_2), 3.51-3.54 (m, 1H, CH), 3.79 (s, 3H, CH_3), 5.82-5.84 (m, 1H, =CH), 5.89-5.92 (m, 1H, =CH), 6.86-6.88 (m, 2H, Ar), 7.26 (d, J = 8.8 Hz, 2H, Ar). ^{13}C NMR (100 MHz, $CDCl_3$, TMS): δ 27.5, 28.6, 32.4, 43.9, 55.2, 113.6, 122.5, 126.7, 127.2, 128.5, 131.5, 138.1, 157.9. IR (CH_2Cl_2) ν 3022, 2935, 2834, 1606, 1510, 1245, 1177, 1035, 829 cm^{-1}. MS (%) m/z 212 (M^+, 100.00), 197 (34.02), 184 (40.86), 169 (38.30), 165 (30.79), 141 (37.92), 128 (13.79), 115 (39.22), 91 (19.21), 77 (18.89). HRMS (EI) calcd. for $C_{15}H_{16}O$: 212.1201, found: 212.1197.

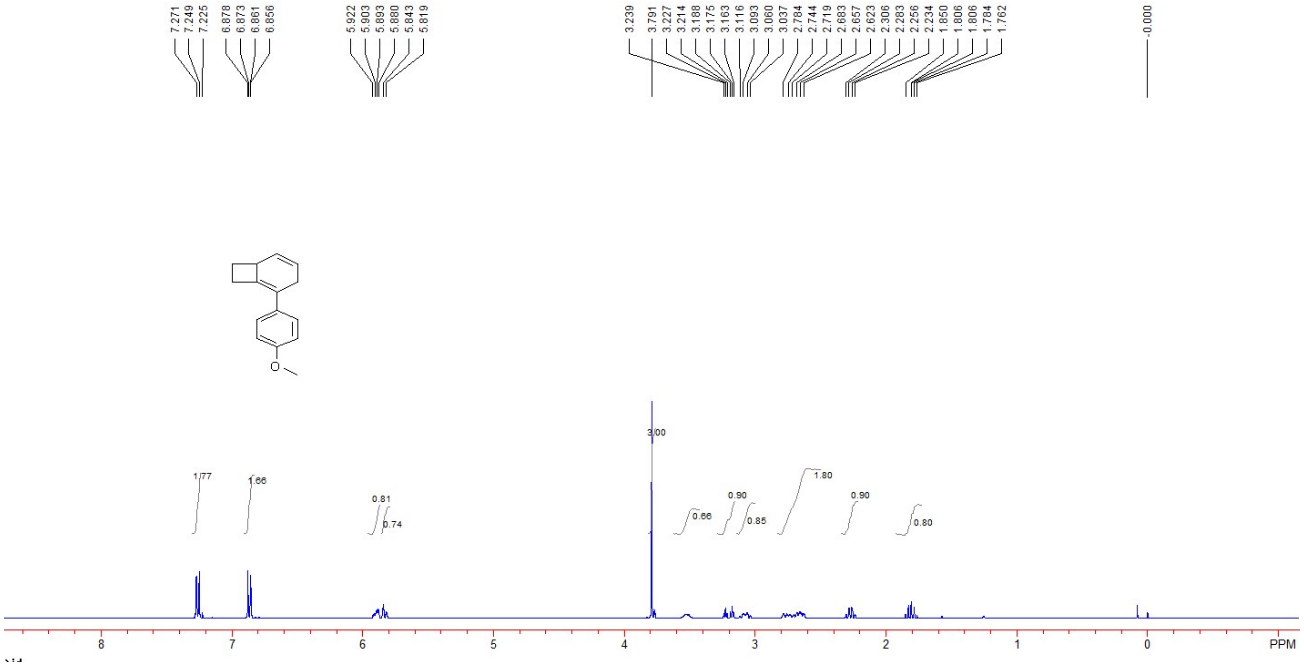

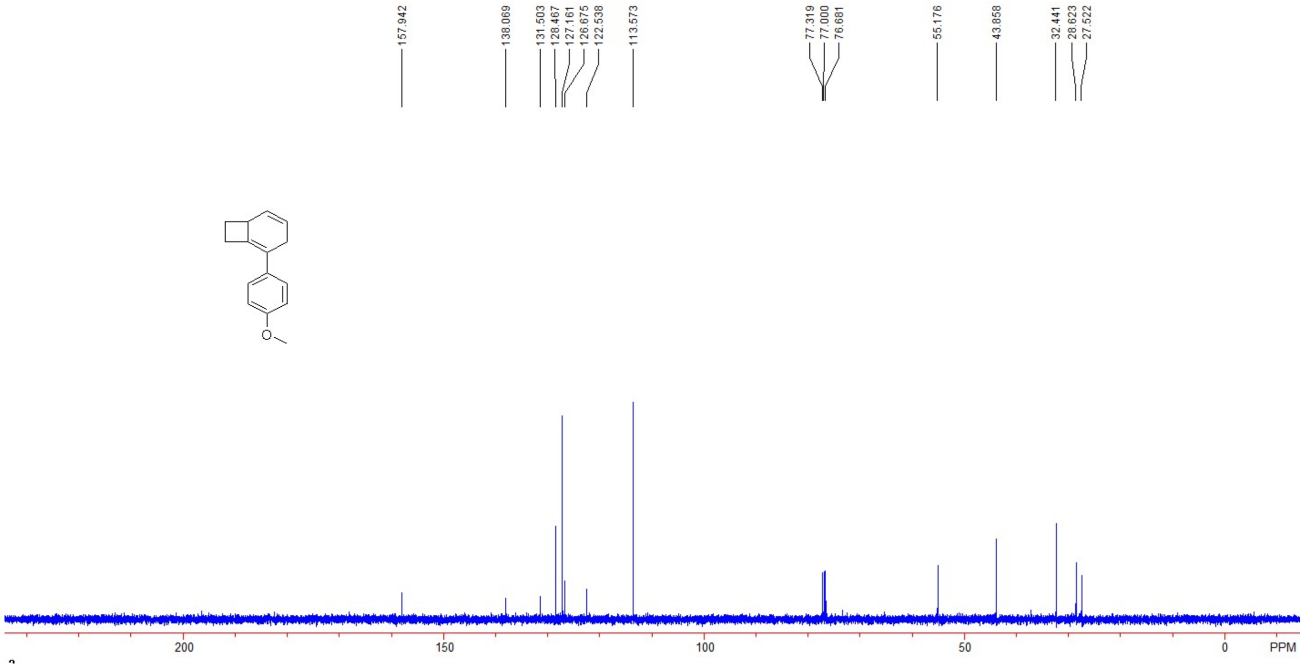

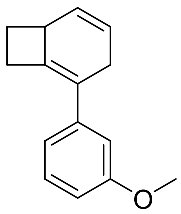

2-Phenylbicyclo[4.2.0]octa-1,4-diene 3e:

37 mg, yield = 88%. Colorless oil. ^{1}H NMR (400 MHz, $CDCl_3$, TMS): δ 1.77-1.83 (m, 1H, CH_2), 2.24-2.32 (m, 1H, CH_2), 2.67-2.80 (m, 2H, CH_2), 3.06-3.13 (m, 1H, CH_2), 3.18-2.21 (m, 1H, CH_2), 3.50-3.58 (m, 1H, CH), 3.80 (s, 3H, CH_3), 5.81-5.84 (m, 1H, =CH), 5.90-5.93 (m, 1H, =CH), 6.75

(dd, J_1 = 8.0 Hz, J_2 = 2.4 Hz, 1H, Ar), 6.87 (dd, J_1 = 2.4 Hz, J_2 = 1.6 Hz, 1H, Ar), 6.93 (d, J = 8.0 Hz, 1H, Ar), 7.23 (d, J = 8.0 Hz, 1H, Ar). ^{13}C NMR (100 MHz, $CDCl_3$, TMS): δ 27.5, 28.5, 32.6, 44.0, 55.1, 111.5, 112.0, 118.7, 123.1, 126.7, 128.2, 129.1, 140.4, 140.5, 159.5. IR (CH_2Cl_2) ν 3023, 2937, 2832, 1597, 1575, 1484, 1210, 1041, 774, 692 cm^{-1}. MS (%) m/z 212 (M^+, 53.91), 197 (26.24), 184 (100.00), 181 (63.27), 165 (41.70), 141 (38.20), 115 (45.38), 91 (18.19), 77 (17.19). HRMS (EI) calcd. for $C_{15}H_{16}O$: 212.1201, found: 212.1204.

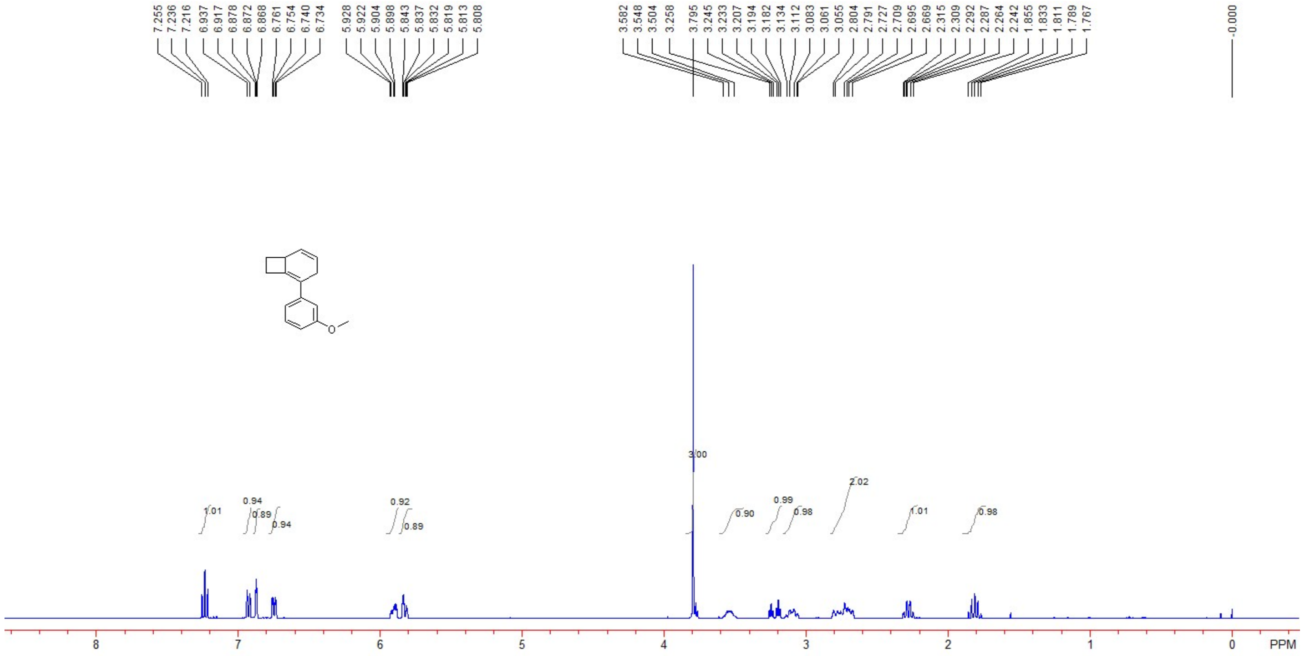

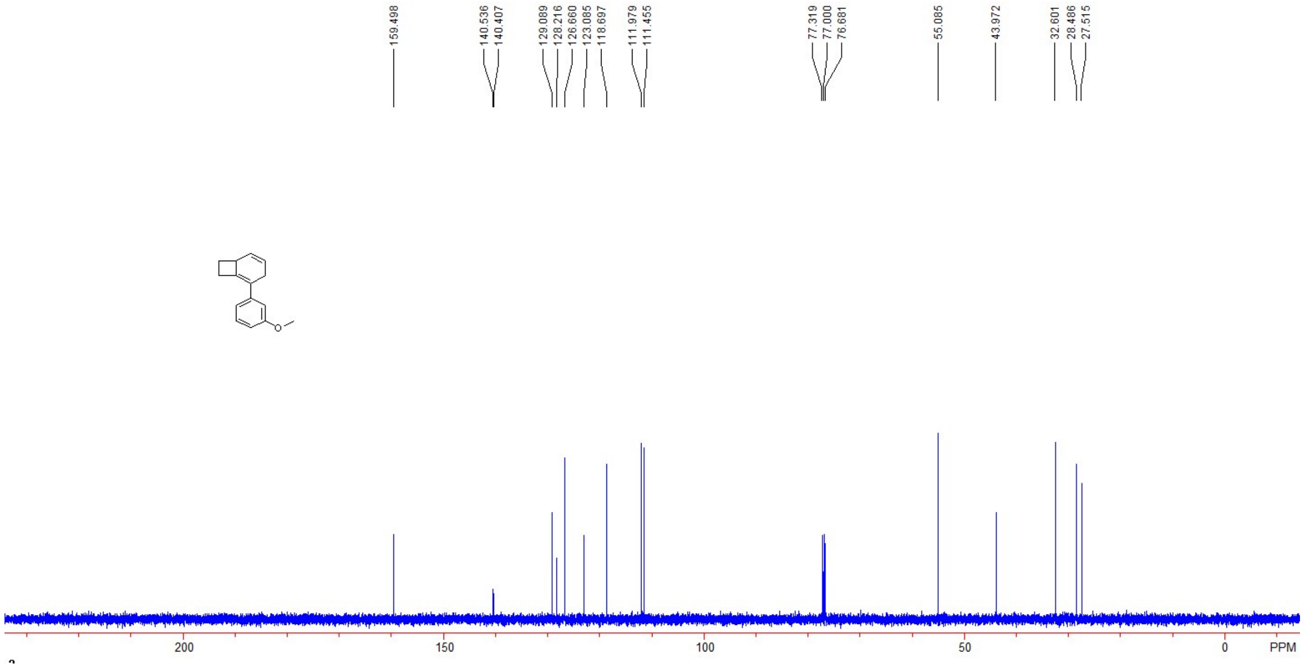

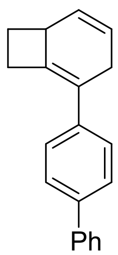

2-([1,1'-Biphenyl]-4-yl)bicyclo[4.2.0]octa-1,4-diene 3f:

48 mg, yield = 92%. White solid. Mp: 80-83 ^oC. ^1H NMR (400 MHz, $CDCl_3$, TMS): δ 1.78-1.87 (m, 1H, CH_2), 2.26-2.32 (m, 1H, CH_2), 2.70-2.83 (m, 2H, CH_2), 3.10-3.14 (m, 1H, CH_2), 3.25-3.30 (m, 1H, CH_2), 3.56-3.57 (m, 1H, CH), 5.83-5.85 (m, 1H, =CH), 5.91 (br, 1H, =CH), 7.29-7.35 (m, 1H, Ar), 7.40-7.48 (m, 4H, Ar), 7.55-7.60 (m, 4H, Ar). ^{13}C NMR (100 MHz, $CDCl_3$, TMS): δ 27.4, 28.6, 32.7, 44.1, 122.8, 126.5, 126.7, 126.9, 127.0, 128.3, 128.7, 137.9, 138.8, 140.5, 140.8. IR (CH_2Cl_2) ν 3020, 2941, 2846, 1628, 1486, 835, 764, 724, 692 cm^{-1}. MS (%) m/z 258 (M^+, 100.00), 243 (29.87), 230 (84.04), 215 (13.30), 167 (26.12), 152 (14.74), 115 (23.18), 91 (24.96), 77 (13.45). HRMS (EI) calcd. for $C_{20}H_{18}$: 258.1409, found: 258.1403.

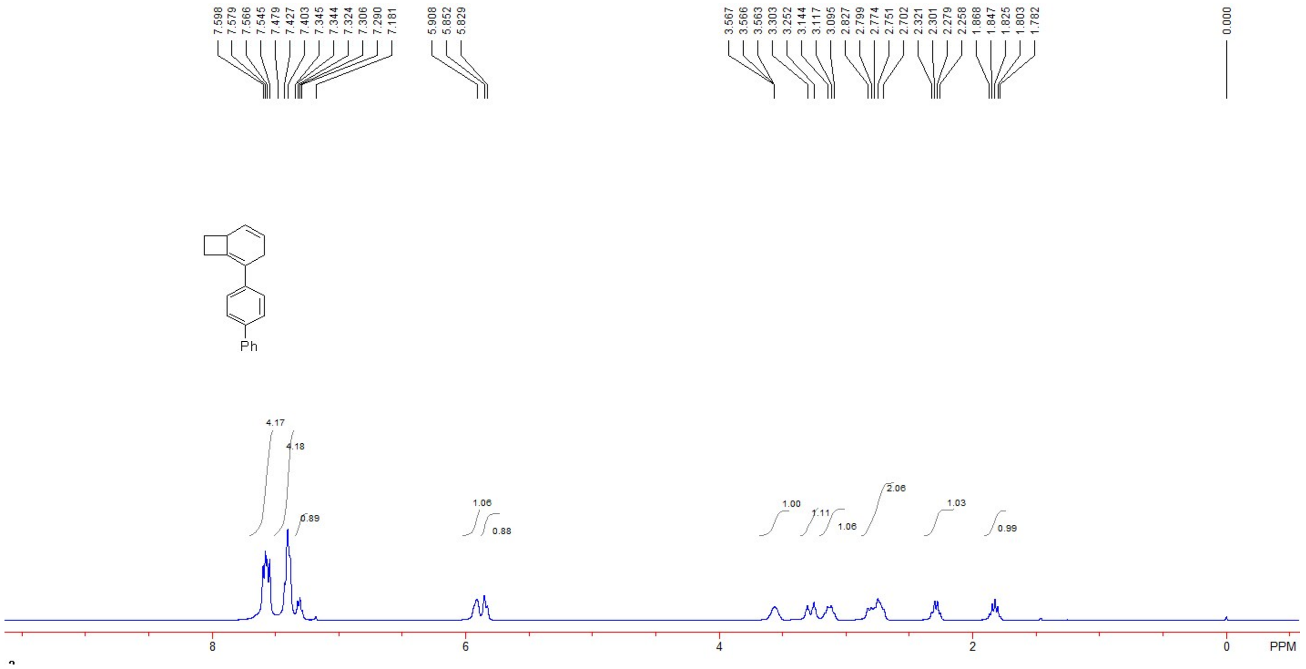

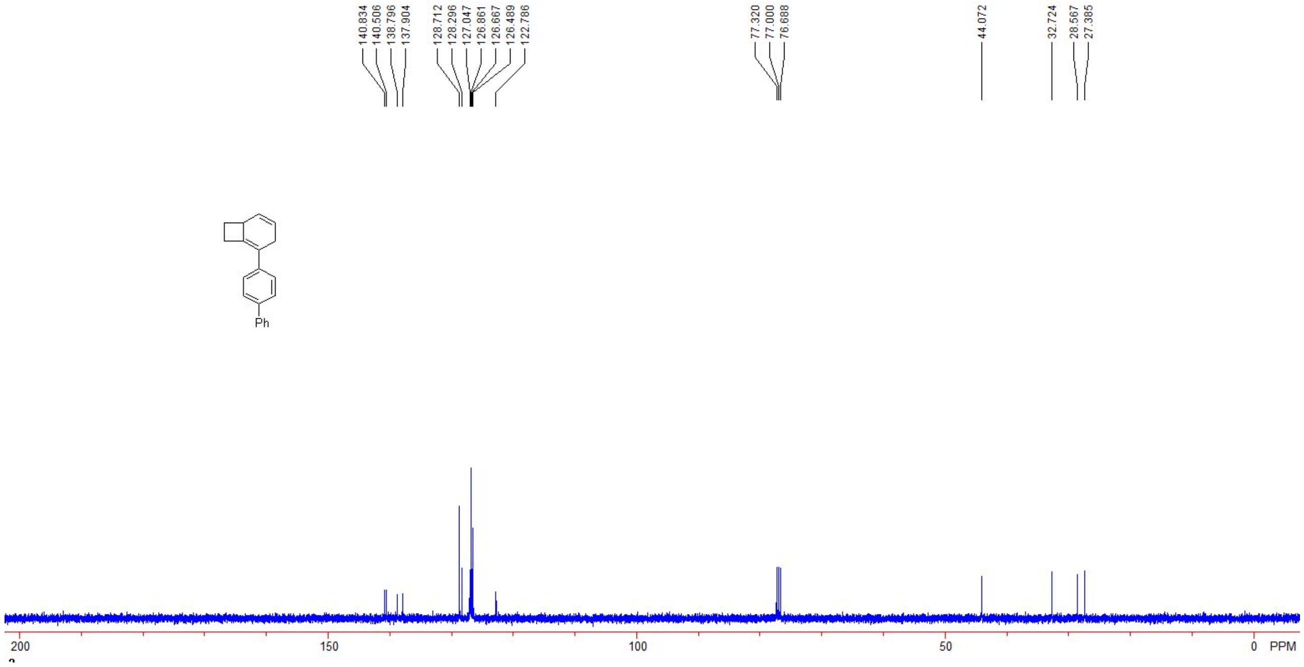

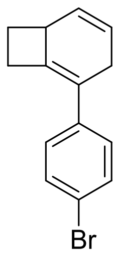

2-(4-Bromophenyl)bicyclo[4.2.0]octa-1,4-diene 3g:

44 mg, yield = 85%. Colorless oil. ^{1}H NMR (400 MHz, $CDCl_3$, TMS): δ 1.79-1.83 (m, 1H, CH_2), 2.25-2.32 (m, 1H, CH_2), 2.60-2.66 (m, 1H, CH_2), 2.71-2.79 (m, 1H, CH_2), 3.00-3.09 (m, 1H, CH_2), 3.13-2.21 (m, 1H, CH_2), 3.48-3.57 (m, 1H, CH), 5.81-5.84 (m, 1H, =CH), 5.88-5.89 (m, 1H, =CH), 7.17 (d, J = 8.4 Hz, 2H, Ar), 7.42 (d, J = 8.4 Hz, 2H, Ar). ^{13}C NMR (100 MHz, $CDCl_3$, TMS): δ 27.3, 28.5, 32.5, 44.0, 119.7, 122.3, 126.4, 127.7, 128.2, 131.2, 137.8, 141.0. IR (CH_2Cl_2) ν 3024, 2939, 2853, 1682, 1489, 1072, 1008, 821, 696 cm^{-1}. MS (%) m/z 260 (M^+, 7.37), 245 (6.92), 232 (48.93), 181 (100.00), 165 (58.85), 152 (44.11), 115 (11.60), 89 (31.58), 76 (30.67). HRMS (EI) calcd. for $C_{14}H_{13}Br$: 260.0201, found: 260.0195.

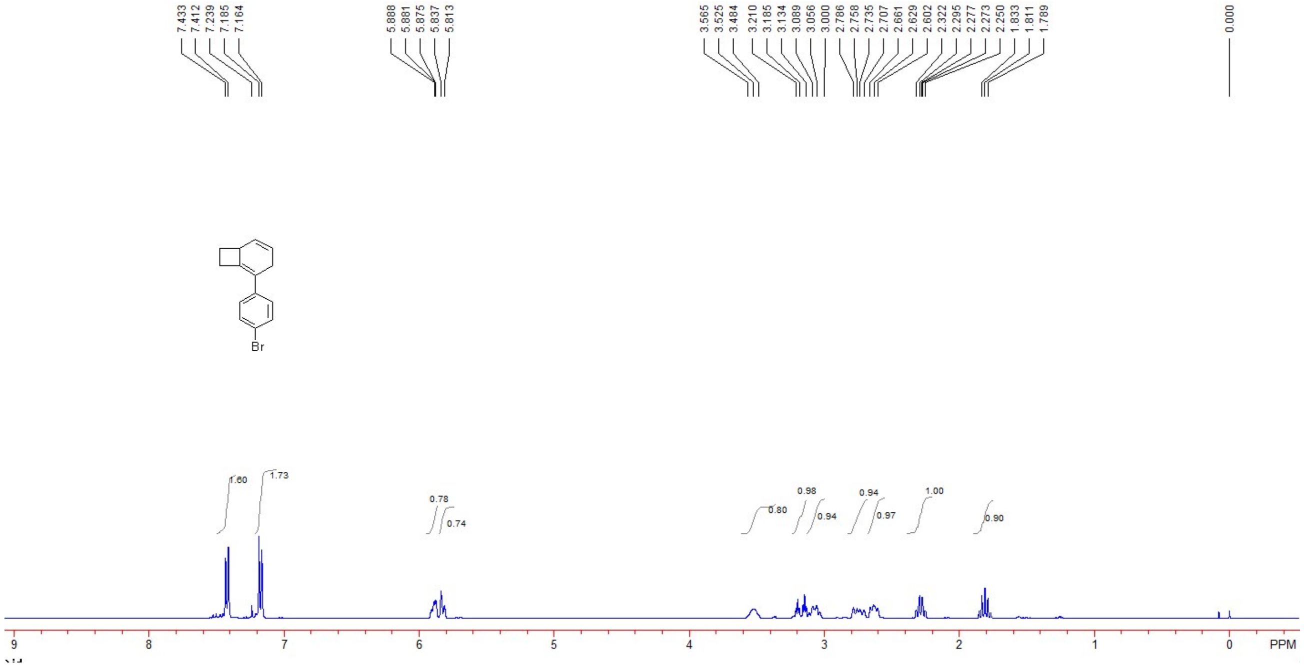

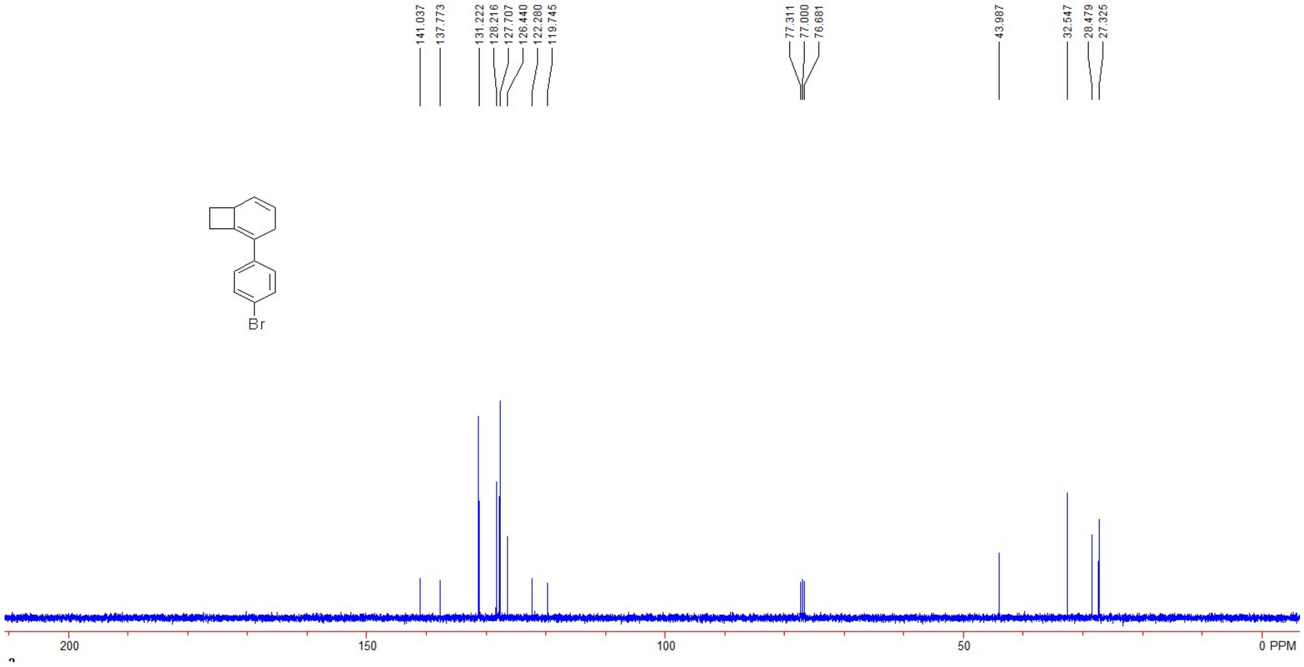

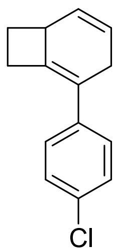

2-(4-Chlorophenyl)bicyclo[4.2.0]octa-1,4-diene 3h:

38 mg, yield = 84%. Colorless oil. ^{1}H NMR (400 MHz, $CDCl_3$, TMS): δ 1.79-1.86 (m, 1H, CH_2), 2.26-2.33 (m, 1H, CH_2), 2.62-2.67 (m, 1H, CH_2), 2.71-2.79 (m, 1H, CH_2), 3.04-3.10 (m, 1H, CH_2),

3.12-3.22 (m, 1H, CH_2), 3.52-3.55 (m, 1H, CH), 5.82-5.84 (m, 1H, =CH), 5.88-5.92 (m, 1H, =CH), 7.24 (d, J = 8.8 Hz, 2H, Ar), 7.28 (d, J = 8.8 Hz, 2H, Ar). ^{13}C NMR (100 MHz, $CDCl_3$, TMS): δ 27.4, 28.5, 32.5, 44.0, 122.3, 126.5, 127.4, 128.27, 128.30, 131.7, 137.3, 140.9. IR (CH_2Cl_2) ν 3026, 2942, 1682, 1491, 1092, 1013, 785, 706 cm^{-1}. MS (%) m/z 216 (M^+, 55.20), 181 (83.17), 166 (100.00), 152 (54.05), 139 (73.49), 125 (45.21), 99 (17.19), 75 (27.42). HRMS (EI) calcd. for $C_{14}H_{13}Cl$: 216.0706, found: 216.0708.

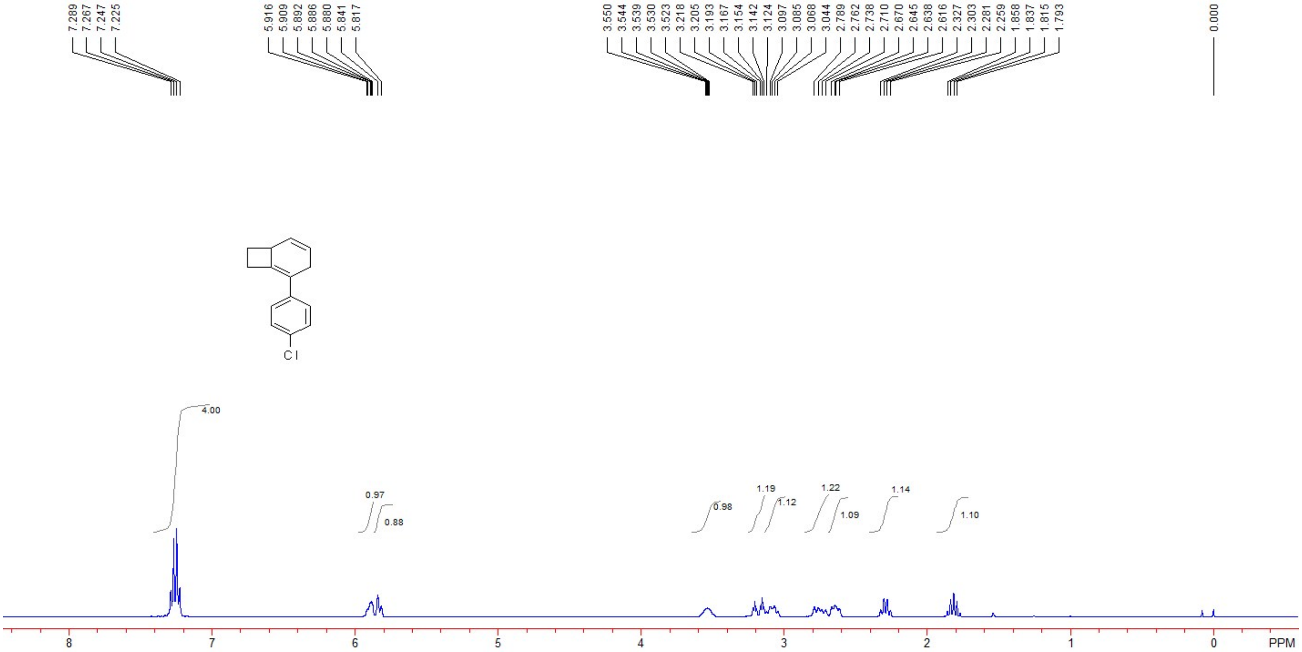

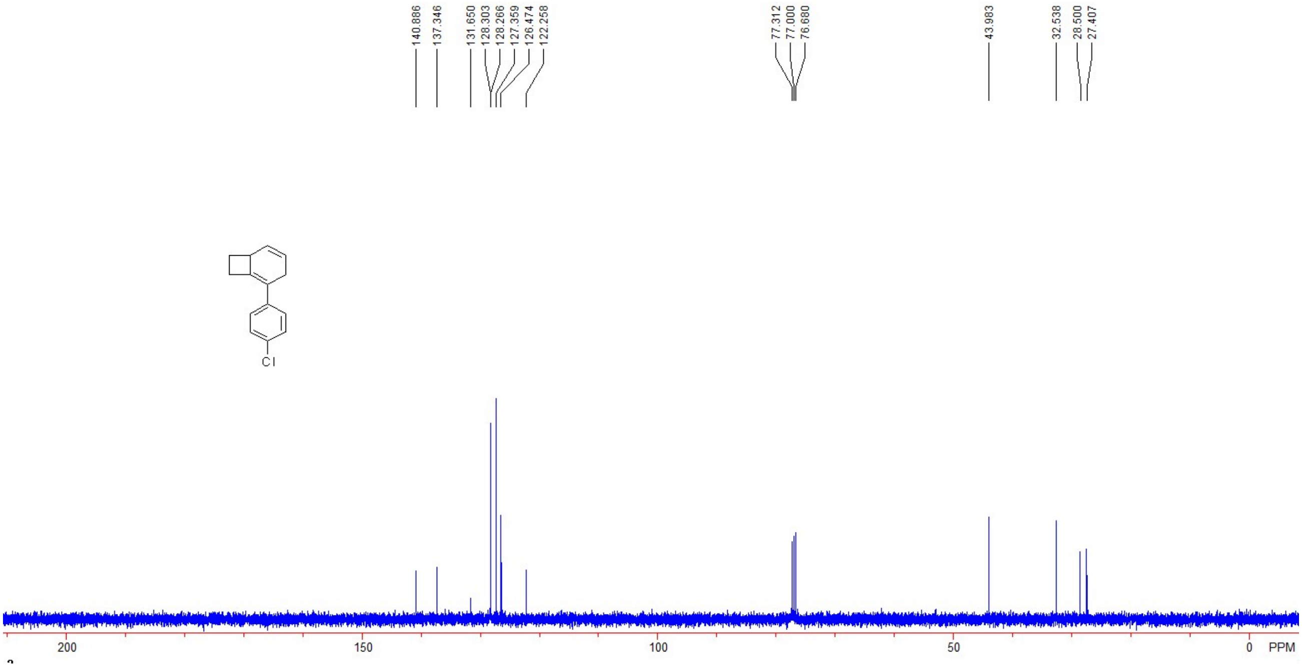

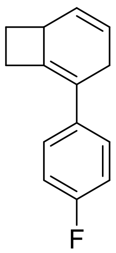

2-(4-Fluorophenyl)bicyclo[4.2.0]octa-1,4-diene 3i:

33 mg, yield = 82%. Colorless oil. ^{1}H NMR (400 MHz, $CDCl_3$, TMS): δ 1.77-1.86 (m, 1H, CH_2), 2.25-2.32 (m, 1H, CH_2), 2.60-2.66 (m, 1H, CH_2), 2.71-2.80 (m, 1H, CH_2), 3.02-3.11 (m, 1H, CH_2), 3.14-3.22 (m, 1H, CH_2), 3.51-3.55 (m, 1H, CH), 5.82-5.85 (m, 1H, =CH), 5.88-5.90 (m, 1H, =CH), 6.98-7.03 (m, 2H, Ar), 7.26-7.29 (m, 2H, Ar). ^{13}C NMR (100 MHz, $CDCl_3$, TMS): δ 27.7, 28.5, 32.4, 43.9, 115.0 (d, J = 20.5 Hz), 122.3, 126.5, 127.6 (d, J = 7.6 Hz), 128.4, 134.9 (d, J = 3.8 Hz), 139.7, 161.3 (d, J = 243.7 Hz). ^{19}F NMR (376 MHz, $CDCl_3$, $CFCl_3$): δ -116.5. IR (CH_2Cl_2) ν 3025, 2940, 2854, 1507, 1231, 1158, 830, 719, 657 cm^{-1}. MS (%) m/z 200 (M^+, 47.93), 185 (44.83), 172 (100.00), 165 (26.35), 146 (12.50), 133 (19.23), 109 (12.37), 91 (30.84), 77 (13.86). HRMS (EI) calcd. for $C_{14}H_{13}F$: 200.1001, found: 200.1002.

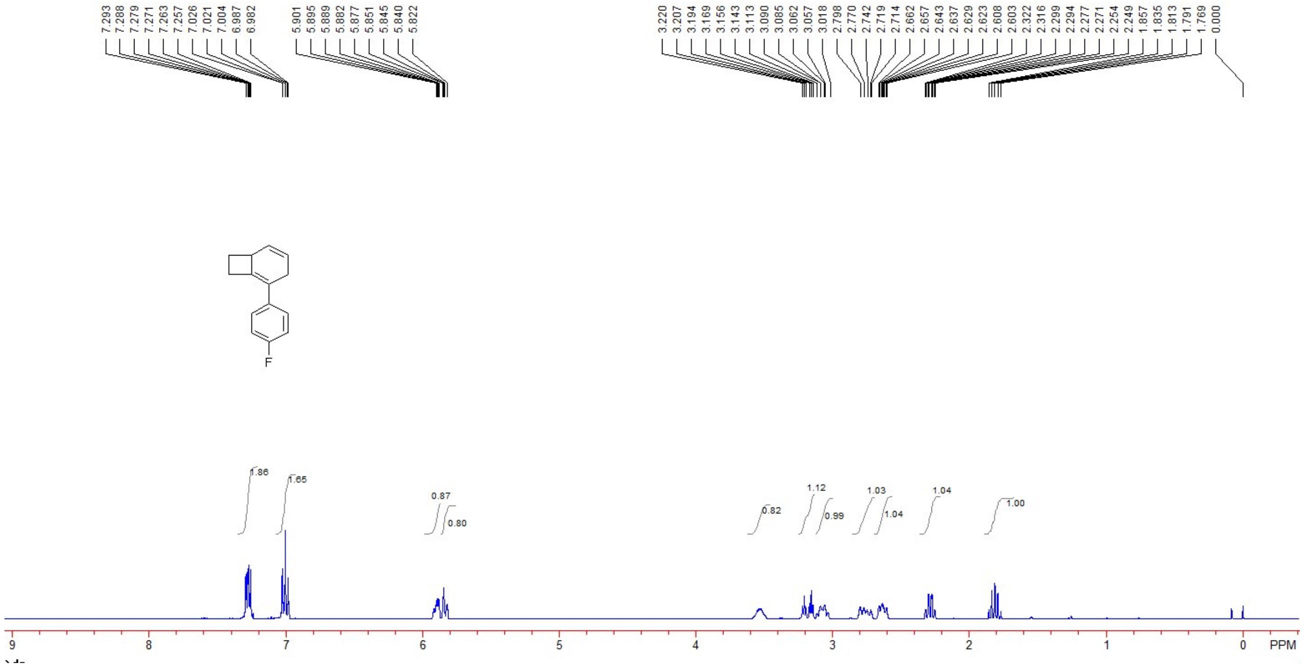

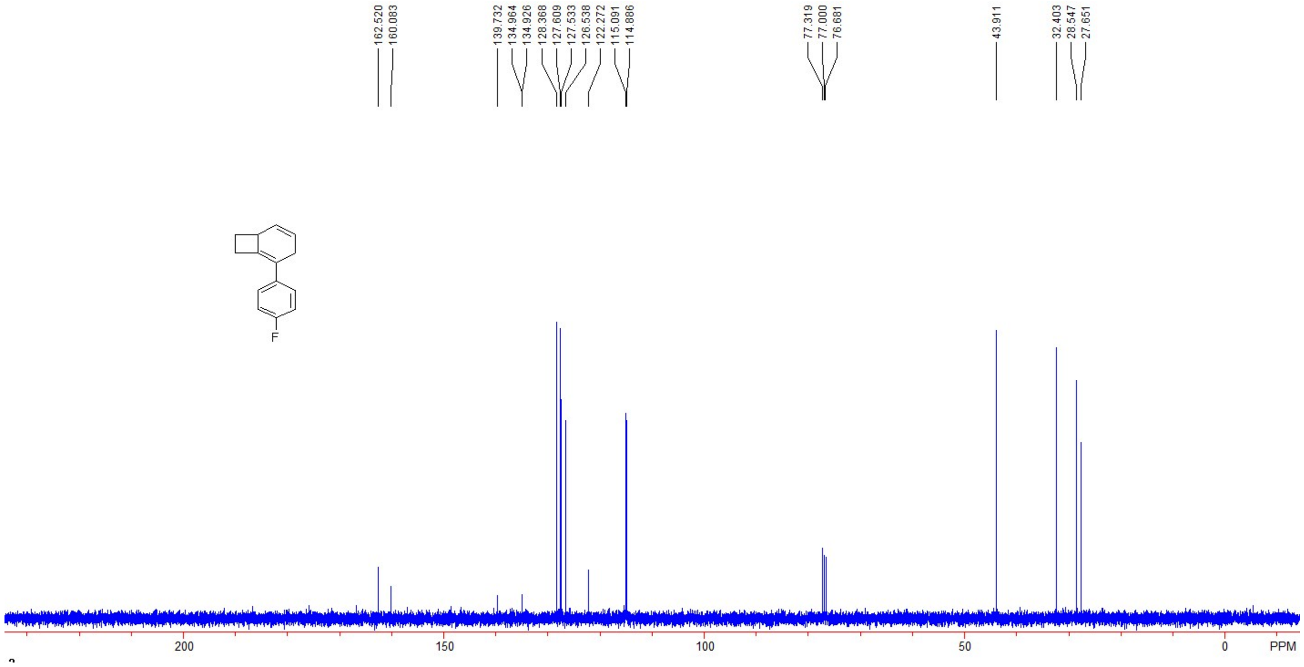

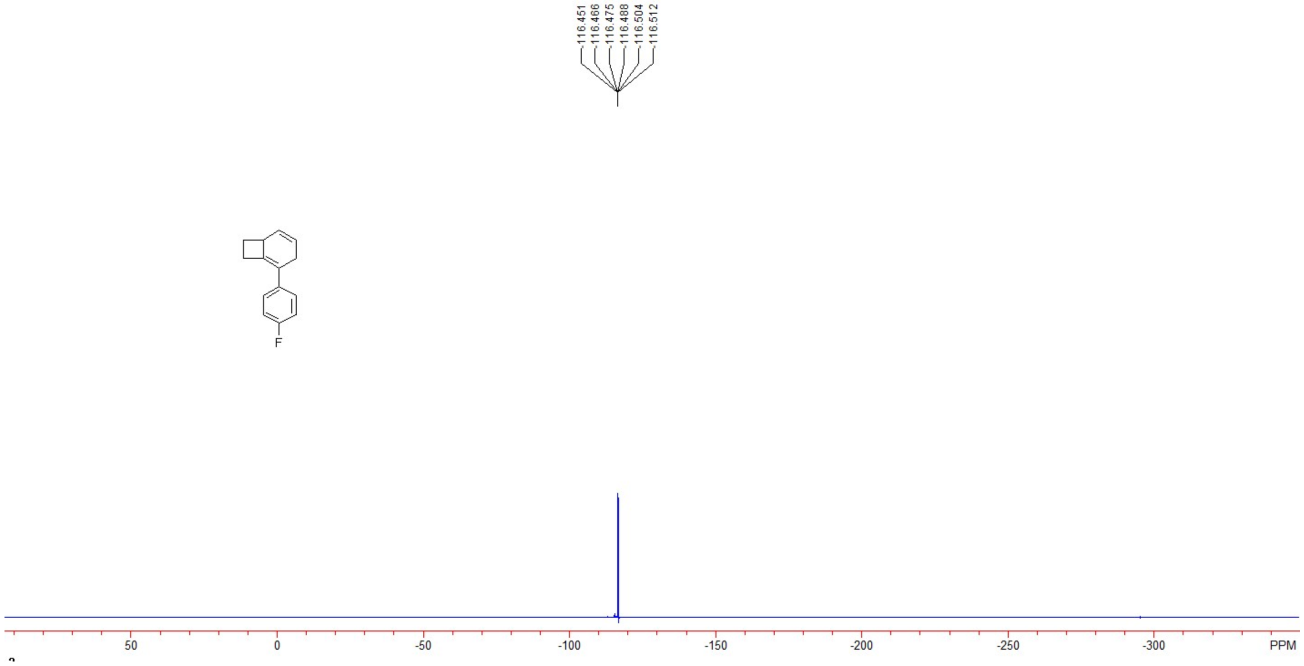

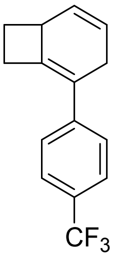

2-(4-(Trifluoromethyl)phenyl)bicyclo[4.2.0]octa-1,4-diene 3j:

33 mg, yield = 82%. Colorless oil. ^{1}H NMR (400 MHz, $CDCl_3$, TMS): δ 1.57-1.88 (m, 1H, CH_2), 2.28-2.35 (m, 1H, CH_2), 2.64-2.70 (m, 1H, CH_2), 2.75-2.82 (m, 1H, CH_2), 3.09-3.17 (m, 1H, CH_2),

3.20-3.28 (m, 1H, CH_2), 3.56-3.59 (m, 1H, CH), 5.82-5.86 (m, 1H, =CH), 5.90-5.94 (m, 1H, =CH), 7.41 (d, J = 8.4 Hz, 2H, Ar), 7.56 (d, J = 8.4 Hz, 2H, Ar). ^{13}C NMR (100 MHz, $CDCl_3$, TMS): δ 27.3, 28.4, 32.7, 44.1, 122.4, 124.4 (q, J = 270.7 Hz), 125.1 (q, J = 3.7 Hz), 126.2, 126.4, 127.9 (q, J = 32.0 Hz), 128.1, 142.5, 143.0. ^{19}F NMR (376 MHz, $CDCl_3$, $CFCl_3$): δ -62.4. IR (CH_2Cl_2) ν 3029, 2943, 2862, 1614, 1322, 1109, 1068, 1014, 837, 719 cm^{-1}. MS (%) m/z 250 (M^+, 56.82), 235 (76.22), 222 (40.51), 209 (100.00), 183 (66.48), 165 (83.26), 152 (42.73), 133 (29.38), 91 (25.23), 78 (24.42). HRMS (EI) calcd. for $C_{15}H_{13}F_3$: 250.0969, found: 250.0970.

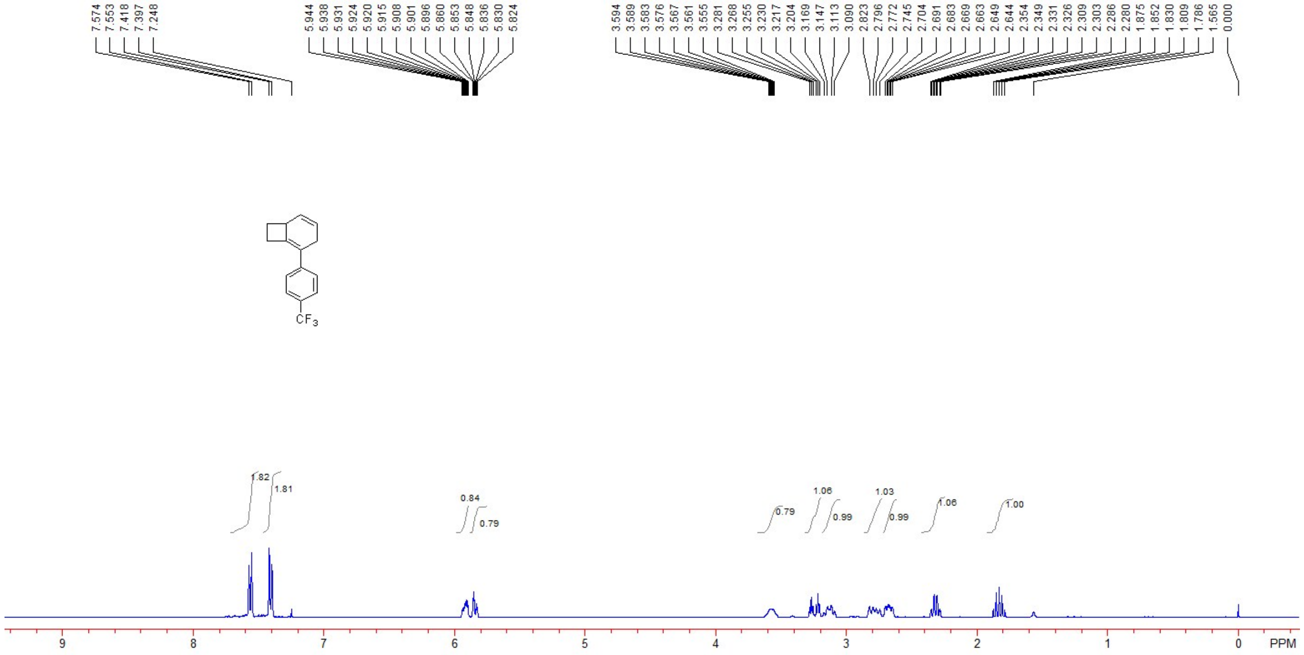

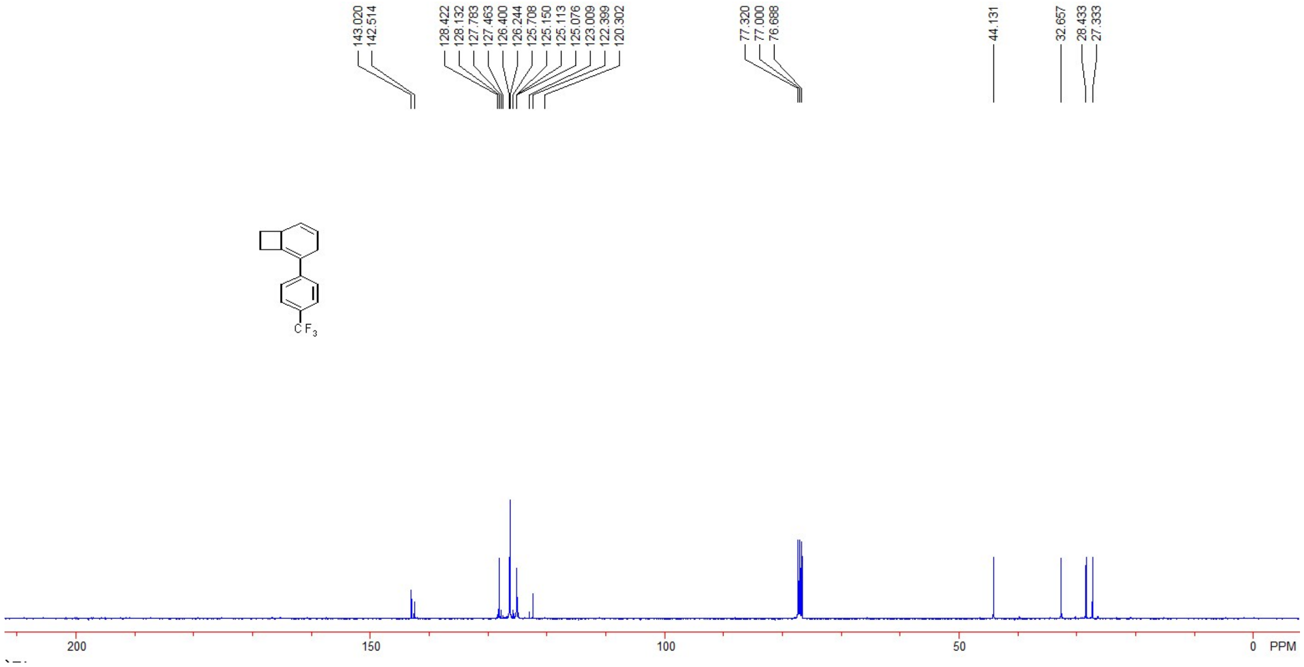

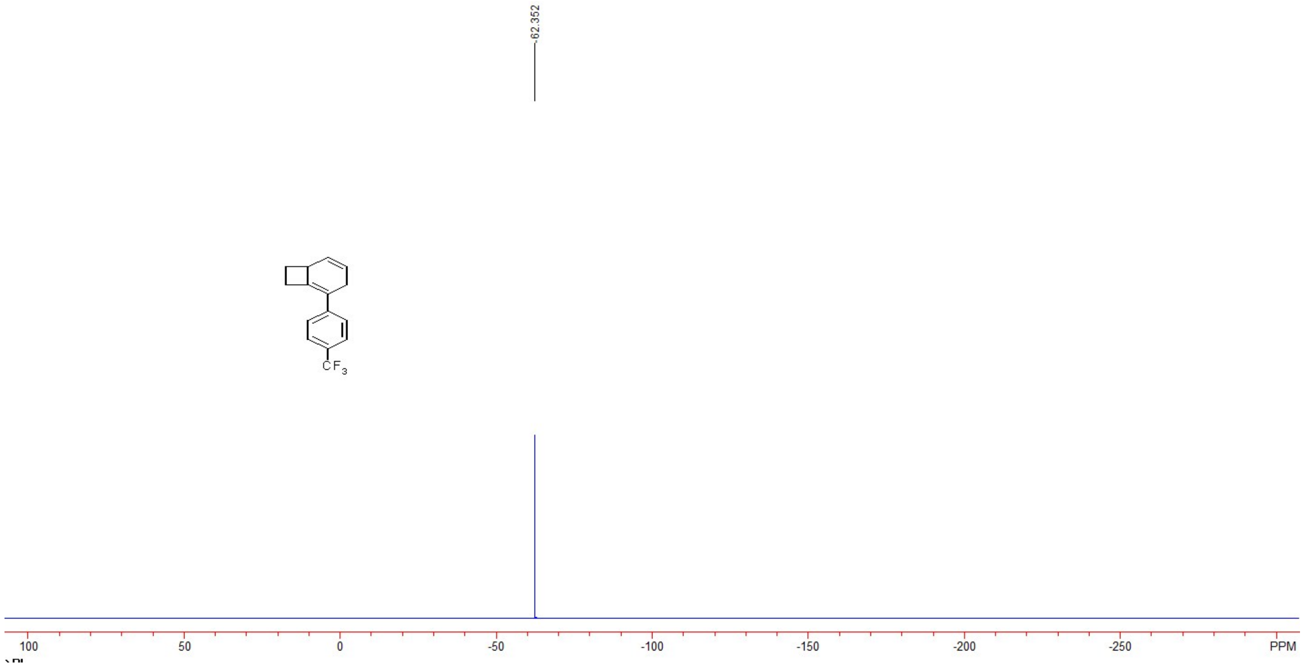

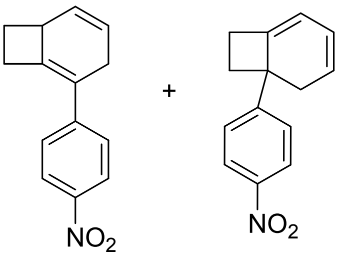

3k **9k**

2-(4-nitrophenyl)bicyclo[4.2.0]octa-1,4-diene 3k and 6-(4-nitrophenyl)bicyclo[4.2.0]octa-1,3-diene 9k:

41 mg, yield = 91%. Mixtures of compound **3k** and **9k** (**3k**:**9k** = 5:1). Colorless oil. ^{1}H NMR (400 MHz, $CDCl_3$, TMS): δ 1.83-1.90 (m, 1.08H, CH_2), 2.21-2.37 (m, 1.82H, CH_2), 2.68-2.79 (m, 1.97H, CH_2), 2.81-2.84 (m, 0.86H, CH_2), 3.17-3.20 (m, 1.02H, CH_2), 3.23-3.30 (m, 1.21H, CH_2), 3.59-3.62 (m, 0.99H, CH), 5.41-5.47 (m, 0.21H, =CH), 5.83-5.86 (m, 1.00H, =CH), 5.90-5.94 (m, 1.20H, =CH), 6.02-6.07 (m, 0.21H, =CH), 7.43-7.49 (m, 2.54H, Ar), 8.15-8.18 (m, 2.45H, Ar). ^{13}C NMR (100 MHz, $CDCl_3$, TMS): δ 27.2, 28.2, 29.9, 33.0, 35.6, 37.4, 44.3, 122.1, 122.5, 123.5, 123.6, 126.1, 126.4, 127.8, 127.9, 145.58, 145.64, 145.9, 153.3. IR (CH_2Cl_2) ν 3028, 2940, 2853, 1756, 1694, 1591, 1511, 1339, 1262, 1205, 1109, 1013, 852, 744, 699 cm^{-1}. MS (%) m/z 227 (M^+, 9.62), 199 (45.85), 179 (50.90), 165 (100.00), 152 (88.70), 141 (35.39), 115 (34.71), 91 (35.85), 77 (34.62). HRMS (EI) calcd. for $C_{14}H_{13}NO_2$: 227.0946, found: 227.0950.

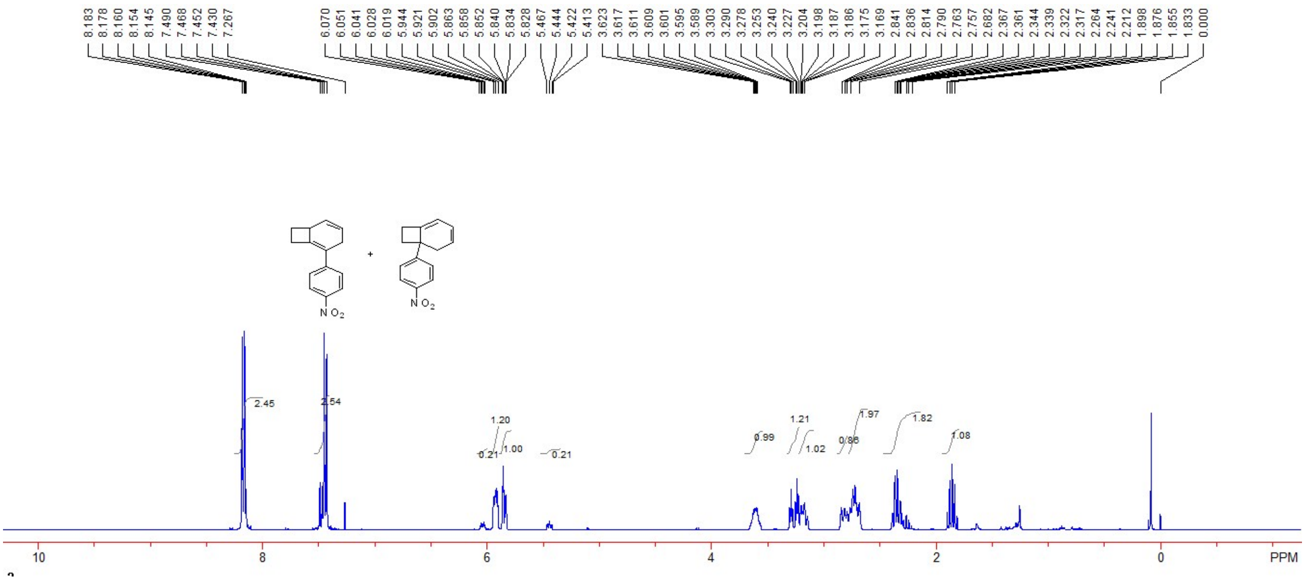

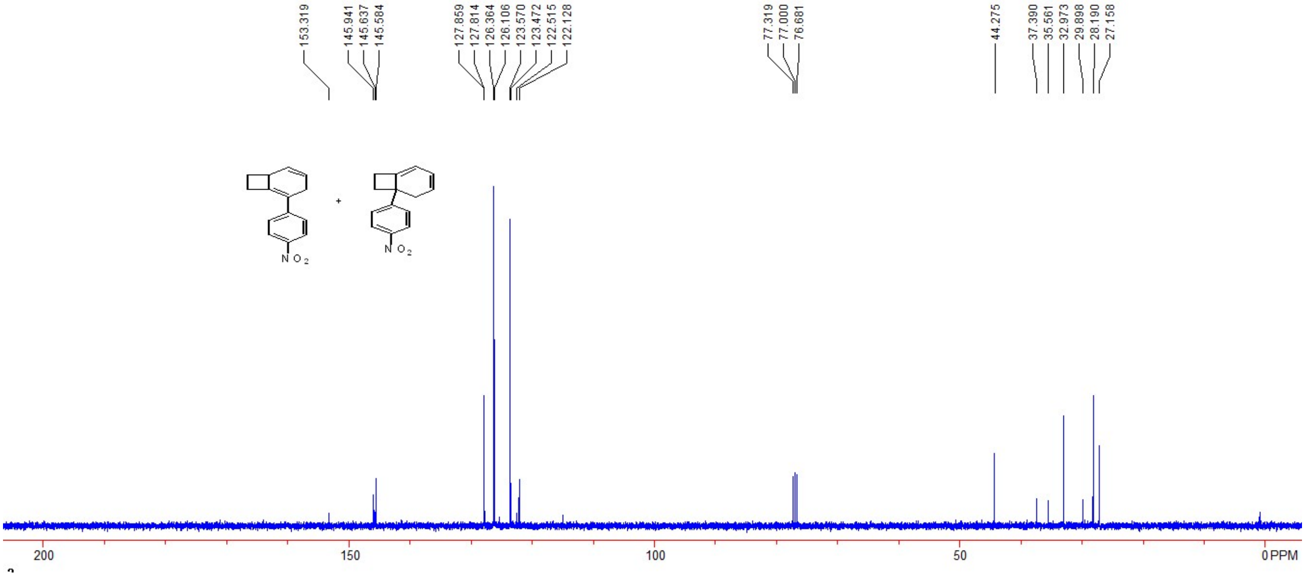

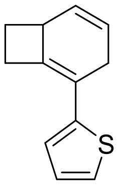

2-(Bicyclo[4.2.0]octa-1,4-dien-2-yl)thiophene 3l:

31 mg, yield = 86%. Colorless oil. ^{1}H NMR (400 MHz, $CDCl_3$, TMS): δ 1.79-1.88 (m, 1H, CH_2), 2.30-2.34 (m, 1H, CH_2), 2.83-2.91 (m, 2H, CH_2), 3.10-3.12 (m, 1H, CH_2), 3.15-3.18 (m, 1H, CH_2), 3.50-3.51 (m, 1H, CH), 5.82-5.90 (m, 2H, =CH), 6.91 (d, J = 3.6 Hz, 1H, Ar), 7.00 (dd, J_1 = 5.2 Hz, J_2 = 3.6 Hz, 1H, Ar), 7.16 (d, J = 5.2 Hz, 1H, Ar). ^{13}C NMR (100 MHz, $CDCl_3$, TMS): δ 28.2, 28.5, 32.6, 43.5, 117.9, 121.6, 123.0, 126.1, 126.8, 128.3, 139.1, 143.3. IR (CH_2Cl_2) ν 3023, 2938, 2850, 1626, 1423, 1249, 816, 660 cm^{-1}. MS (%) m/z 188 (M^+, 57.62), 187 (100.00), 173 (22.80), 160

(86.51), 153 (38.56), 128 (57.04), 115 (66.32), 97 (51.96), 77 (28.71), 45 (27.86). HRMS (EI) calcd. for $C_{12}H_{12}S$: 188.0660, found: 188.0665.

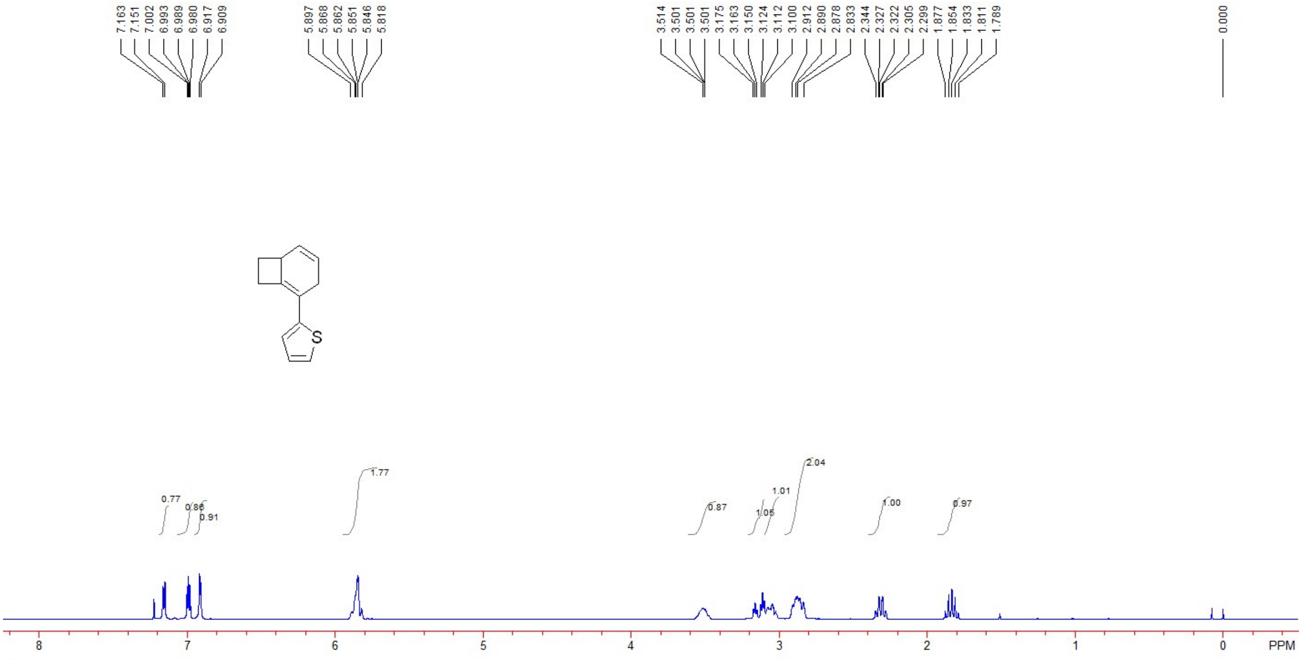

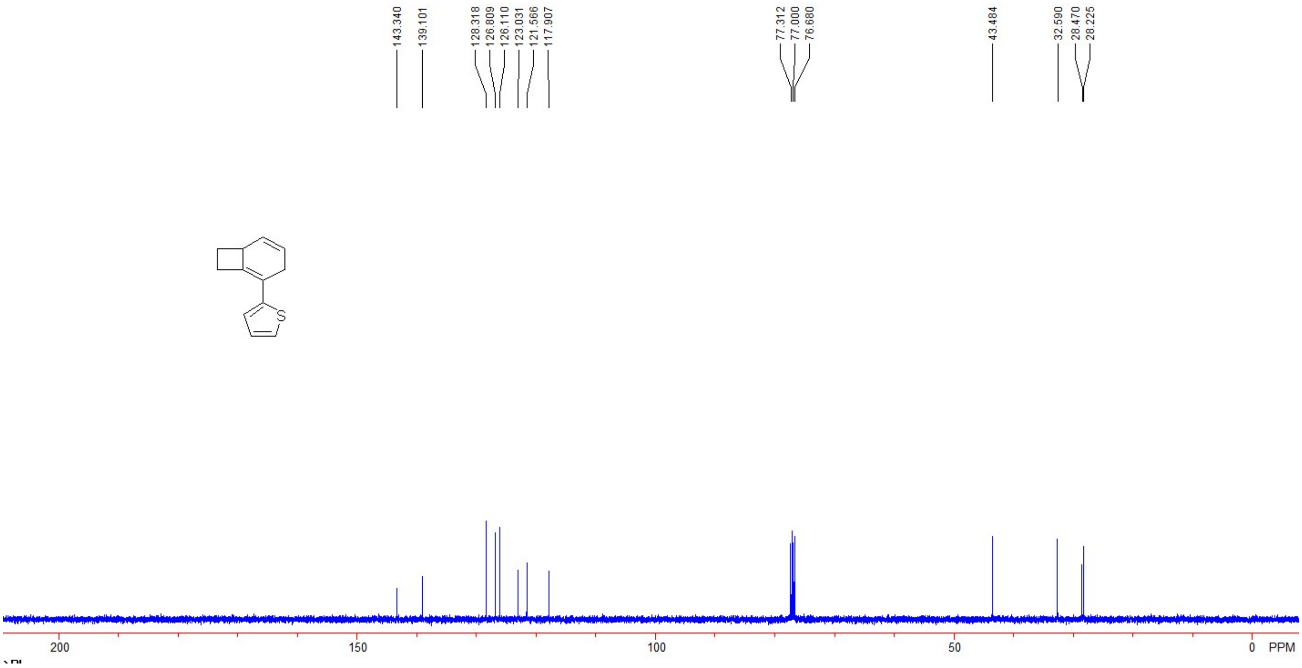

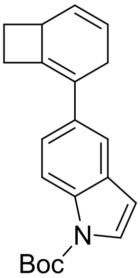

***tert*-Butyl 5-(bicyclo[4.2.0]octa-1,4-dien-2-yl)-1H-indole-1-carboxylate 3m**:

61 mg, yield = 95%. White solid, mp: 69-71 °C. ^{1}H NMR (400 MHz, $CDCl_3$, TMS): δ 1.62 (s, 9H, CH_3), 1.66-1.86 (m, 1H, CH_2), 2.25-2.31 (m, 1H, CH_2), 2.66-2.71 (m, 1H, CH_2), 2.78-2.86 (m, 1H, CH_2), 3.08-3.16 (m, 1H, CH_2), 3.27-3.34 (m, 1H, CH_2), 3.54-3.57 (m, 1H, CH), 5.83-5.86 (m, 1H, =CH), 5.91-5.95 (m, 1H, =CH), 6.53 (d, J = 3.6 Hz, 1H, Ar), 7.31 (d, J = 8.8 Hz, 1H, Ar), 7.49 (s, 1H, Ar), 7.56 (d, J = 2.8 Hz, 1H, Ar), 7.59 (s, 1H, Ar), 8.06 (d, J = 7.6 Hz, 1H, Ar). ^{13}C NMR (100 MHz, $CDCl_3$, TMS): δ 27.9, 28.1, 28.5, 32.5, 43.9, 83.5, 107.4, 114.7, 118.1, 123.2, 123.4, 126.0, 126.8, 128.4, 130.6, 133.7, 138.8, 149.7. IR (CH_2Cl_2) ν 2977, 2936, 1730, 1469, 1367, 1134, 1022, 766, 726 cm^{-1}. HRMS (ESI) Calcd. for $C_{21}H_{24}NO_2$ (M^++H) requires 322.1802, found: 322.1791.

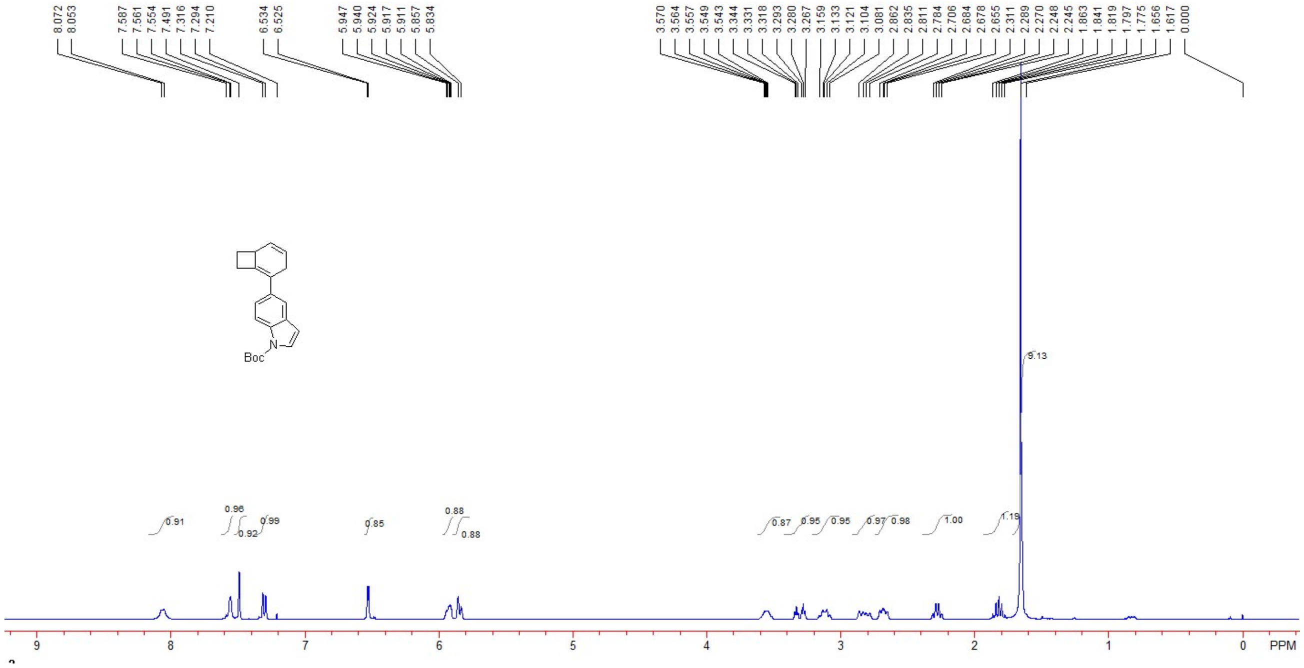

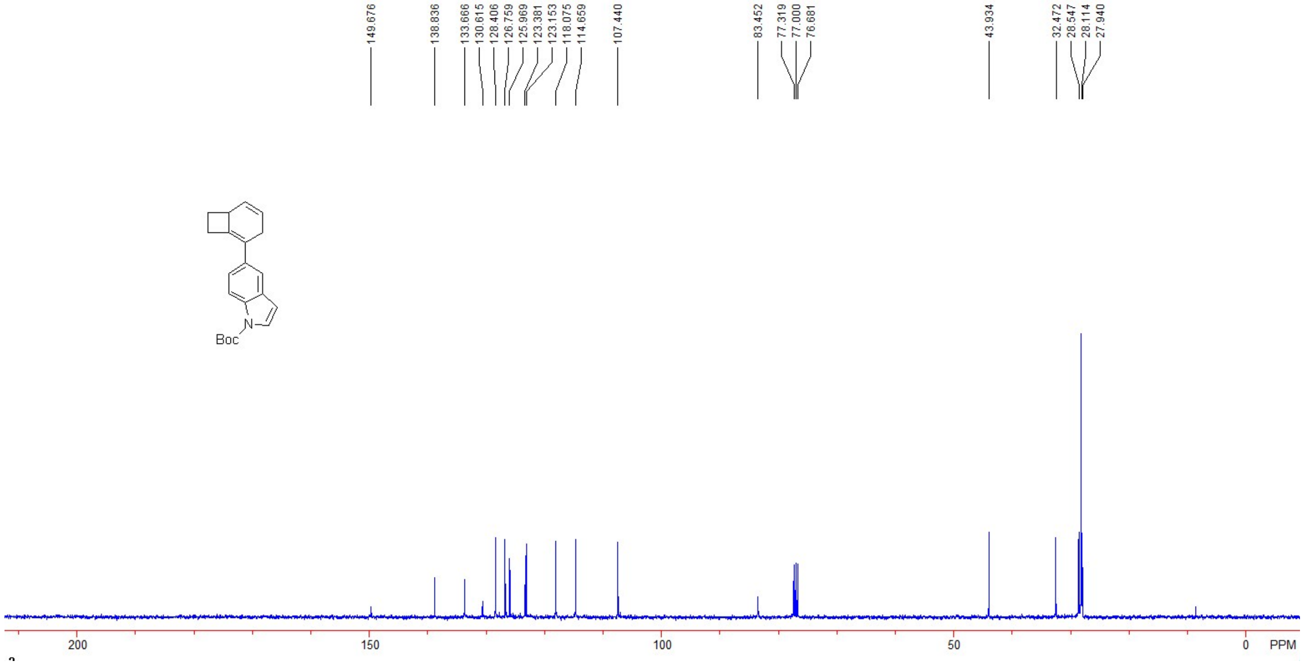

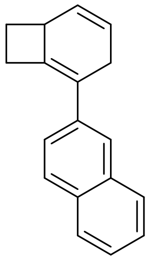

2-(Bicyclo[4.2.0]octa-1,4-dien-2-yl)naphthalene 3n:

40 mg, yield = 87%. A white solid. Mp: 79-82 ^{o}C. ^{1}H NMR (400 MHz, $CDCl_3$, TMS): δ 1.82-1.86 (m, 1H, CH_2), 2.29-2.32 (m, 1H, CH_2), 2.72-2.78 (m, 1H, CH_2), 2.87-2.89 (m, 1H, CH_2), 3.17-3.20 (m, 1H, CH_2), 3.36-3.42 (m, 1H, CH_2), 3.58-3.59 (m, 1H, CH), 5.85-5.88 (m, 1H, =CH), 5.95-5.97 (m, 1H, =CH), 7.39-7.45 (m, 2H, Ar), 7.49-7.51 (m, 1H, Ar), 7.73-7.80 (m, 4H, Ar). ^{13}C NMR (100 MHz, $CDCl_3$, TMS): δ 27.6, 28.4, 32.7, 44.1, 123.3, 124.0, 125.3, 125.4, 125.9, 126.7, 127.46, 127.52, 127.9, 128.3, 132.1, 133.6, 136.5, 140.9. IR (CH_2Cl_2) ν 3053, 2938, 2851, 1626, 1504, 853, 814, 743, 657 cm^{-1}. MS (%) m/z 232 (M^+, 44.26), 215 (38.28), 204 (100.00), 165 (7.80), 141 (32.84), 115 (13.13), 101 (23.36), 89 (8.84), 77 (8.30). HRMS (EI) calcd. for $C_{18}H_{16}$: 232.1252, found: 232.1258.

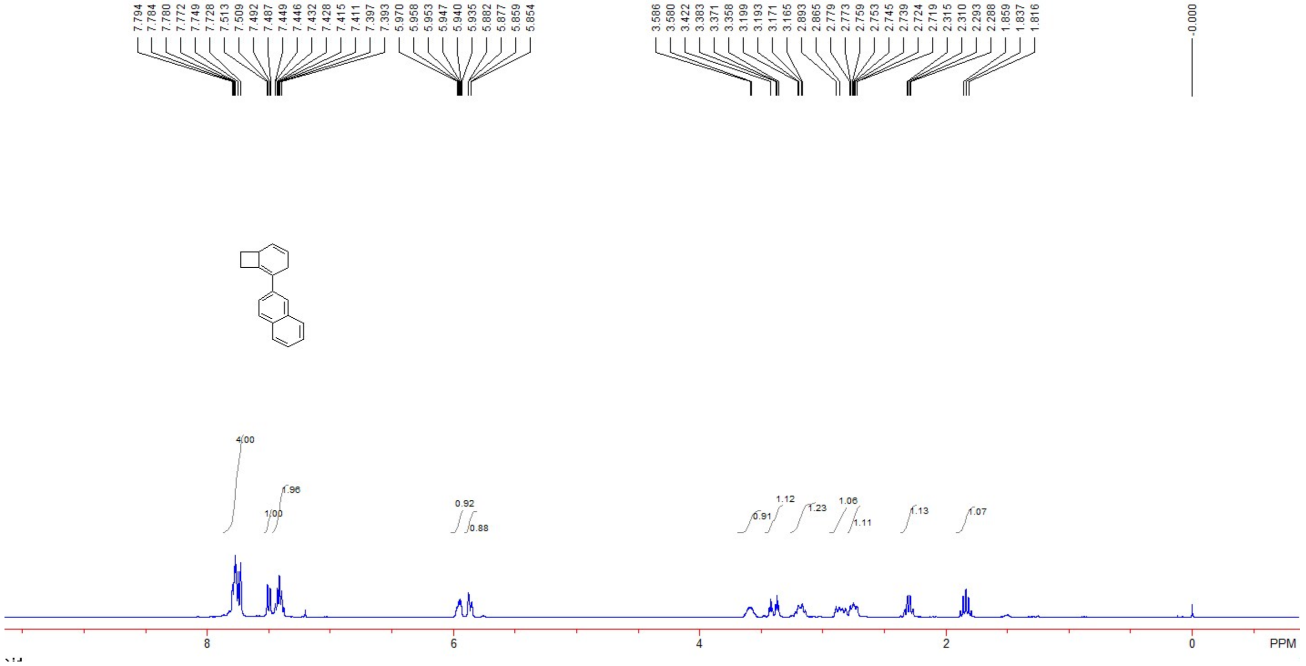

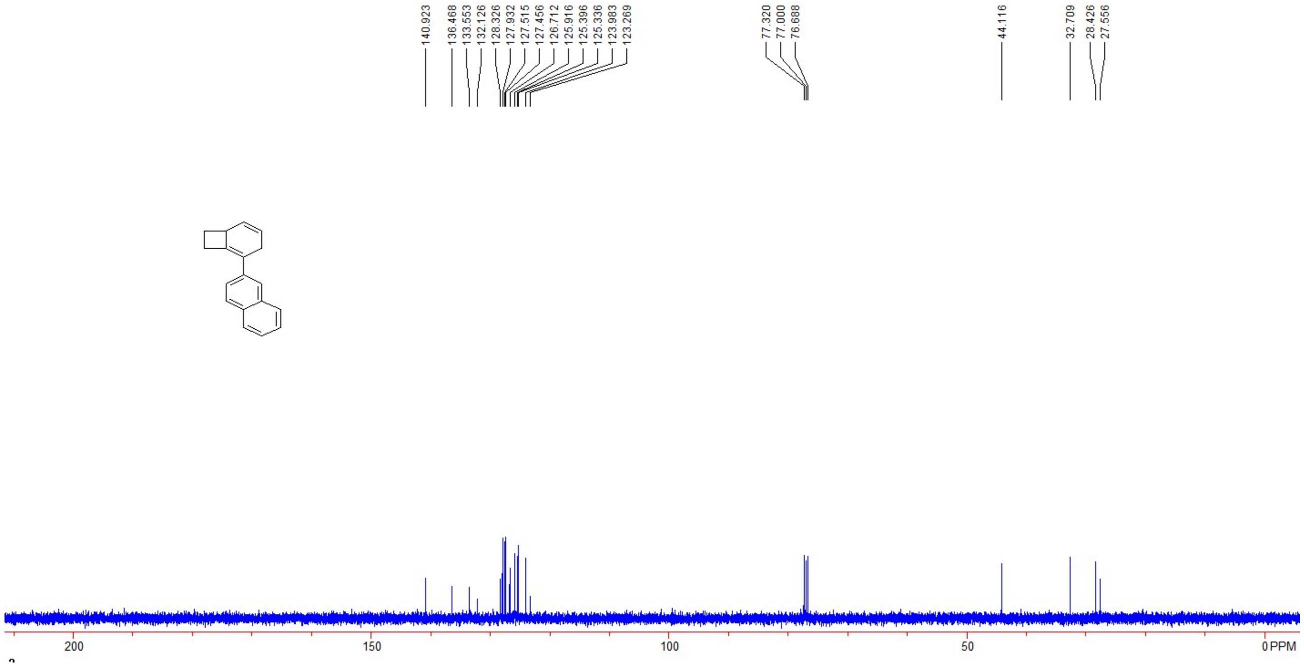

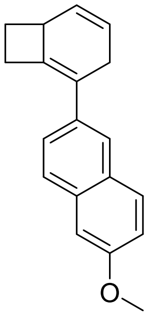

2-(Bicyclo[4.2.0]octa-1,4-dien-2-yl)-6-methoxynaphthalene 3o:

49 mg, yield = 94%. White solid, mp: 98-100 ^{o}C. ^{1}H NMR (400 MHz, $CDCl_3$, TMS): δ 1.81-1.85 (m, 1H, CH_2), 2.25-2.32 (m, 1H, CH_2), 2.71-2.87 (m, 2H, CH_2), 3.12-3.20 (m, 1H, CH_2), 3.24-3.58 (m, 1H, CH_2), 3.57-3.58 (m, 1H, CH), 3.86 (s, 3H, CH_3), 5.84-5.87 (m, 1H, =CH), 5.92-5.93 (m, 1H, =CH), 7.07 (s, 1H, Ar), 7.10 (d, J = 8.4 Hz, 1H, Ar), 7.46 (d, J = 8.8 Hz, 1H, Ar), 7.63-7.68 (m, 3H, Ar). ^{13}C NMR (100 MHz, $CDCl_3$, TMS): δ 27.5, 28.4, 32.6, 44.0, 55.2, 105.5, 118.7, 123.1, 125.8, 126.4, 126.7, 128.4, 129.0, 129.4, 133.1, 134.3, 139.9, 157.3. IR (CH_2Cl_2) ν 3022, 2937, 2838, 1601, 1484, 1201, 1031, 850, 654 cm^{-1}. MS (%) m/z 262 (M^+, 100.00), 247 (28.37), 234 (72.22), 215 (26.88), 202 (23.29), 191 (34.75), 171 (33.65), 117 (17.52), 101 (16.81). HRMS (EI) calcd. for $C_{19}H_{18}O$: 262.1358, found: 262.1356.

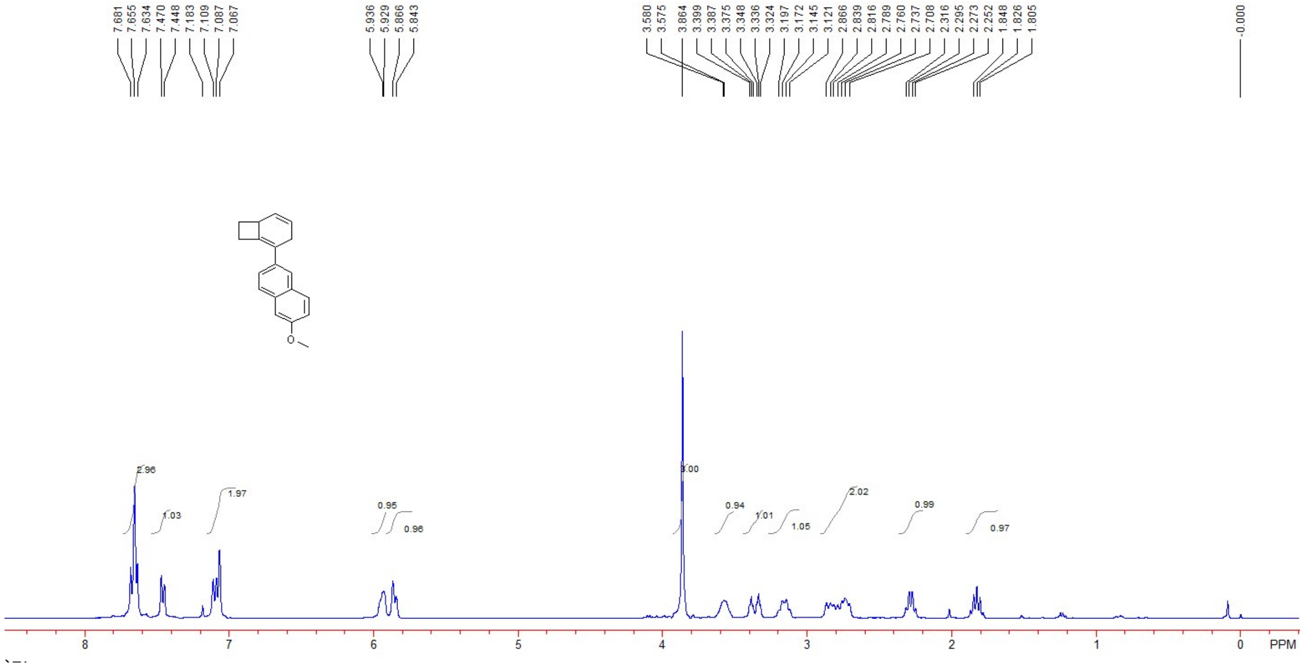

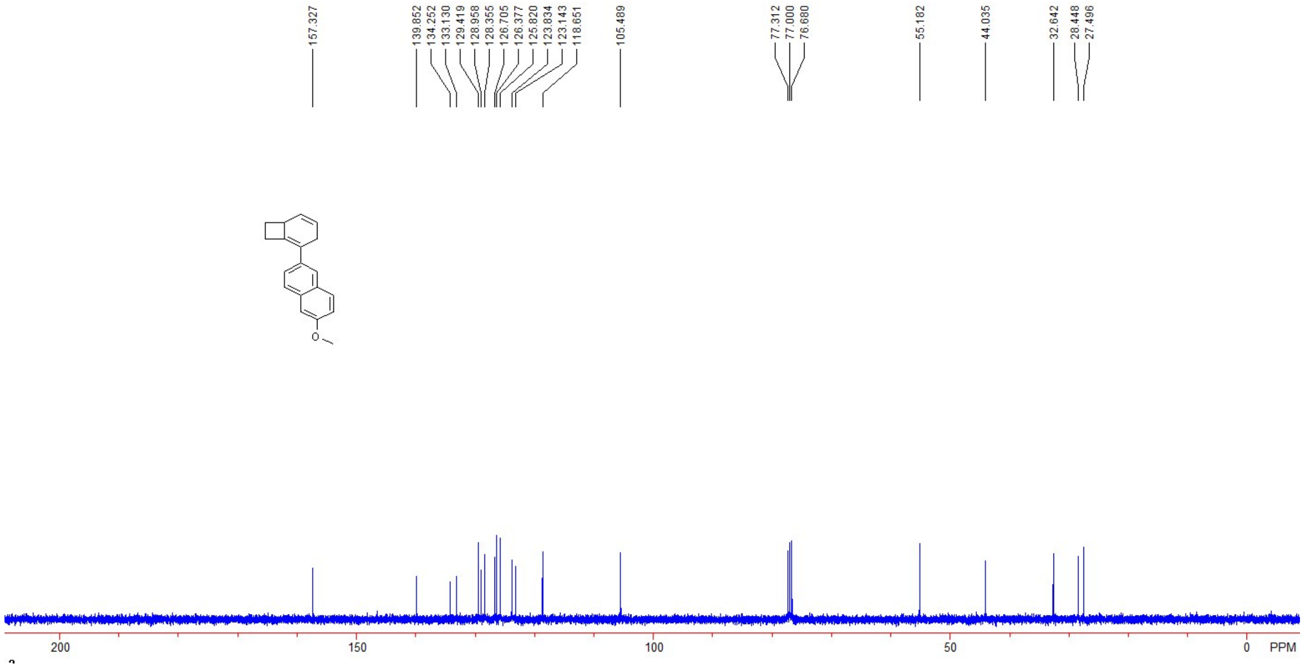

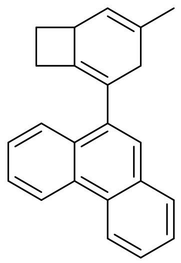

9-(4-Methylbicyclo[4.2.0]octa-1,4-dien-2-yl)phenanthrene 3p:

41 mg, yield = 69%. Colorless oil. ^{1}H NMR (400 MHz, CDCl$_3$, TMS): δ 1.24-1.28 (m, 3H, CH$_3$), 2.05-2.13 (m, 1H, CH$_2$), 2.42-2.47 (m, 3H, CH$_2$), 2.57-2.69 (m, 2H, CH$_2$), 2.84-2.89 (m, 1H, CH),

5.68 (d, J = 2.4 Hz, 1H, =CH), 7.55-7.66 (m, 5H, Ar), 7.85 (d, J = 8.0 Hz, 2H, Ar), 8.09 (d, J = 8.0 Hz, 2H, Ar), 8.66 (d, J = 8.4 Hz, 2H, Ar), 8.70 (d, J = 8.0 Hz, 2H, Ar). ^{13}C NMR (100 MHz, $CDCl_3$, TMS): δ 21.5, 29.2, 30.4, 30.5, 31.3, 122.5, 122.7, 125.8, 126.2, 126.3, 126.6, 127.0, 128.4, 129.9, 130.4, 131.0, 131.8, 133.9, 136.1, 139.7, 140.4. IR (CH_2Cl_2) ν 2951, 2920, 2826, 1698, 1450, 1235, 906, 746, 726 cm^{-1}. MS (%) m/z 296 (M$^+$, 100.00), 281 (46.73), 265 (47.96), 253 (44.49), 239 (25.02), 215 (17.20), 133 (24.74), 126 (24.11), 120 (13.20), 79 (5.88). HRMS (EI) calcd. for $C_{23}H_{20}$: 296.1565, found: 296.1563.

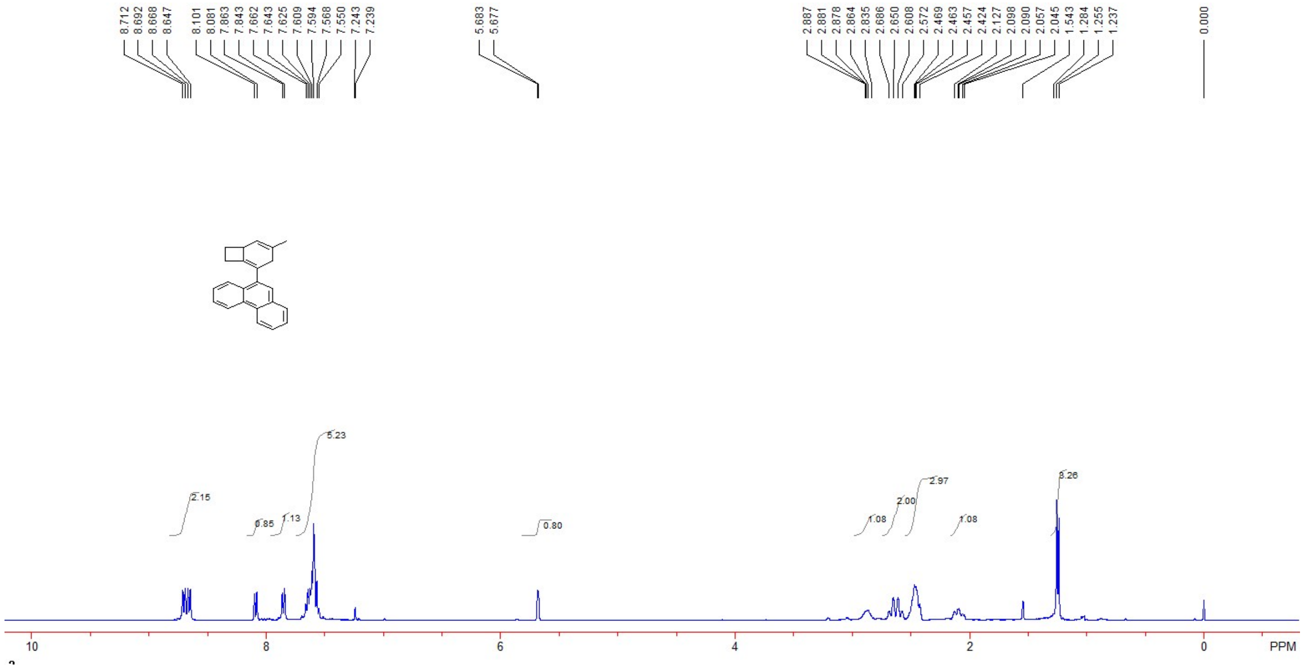

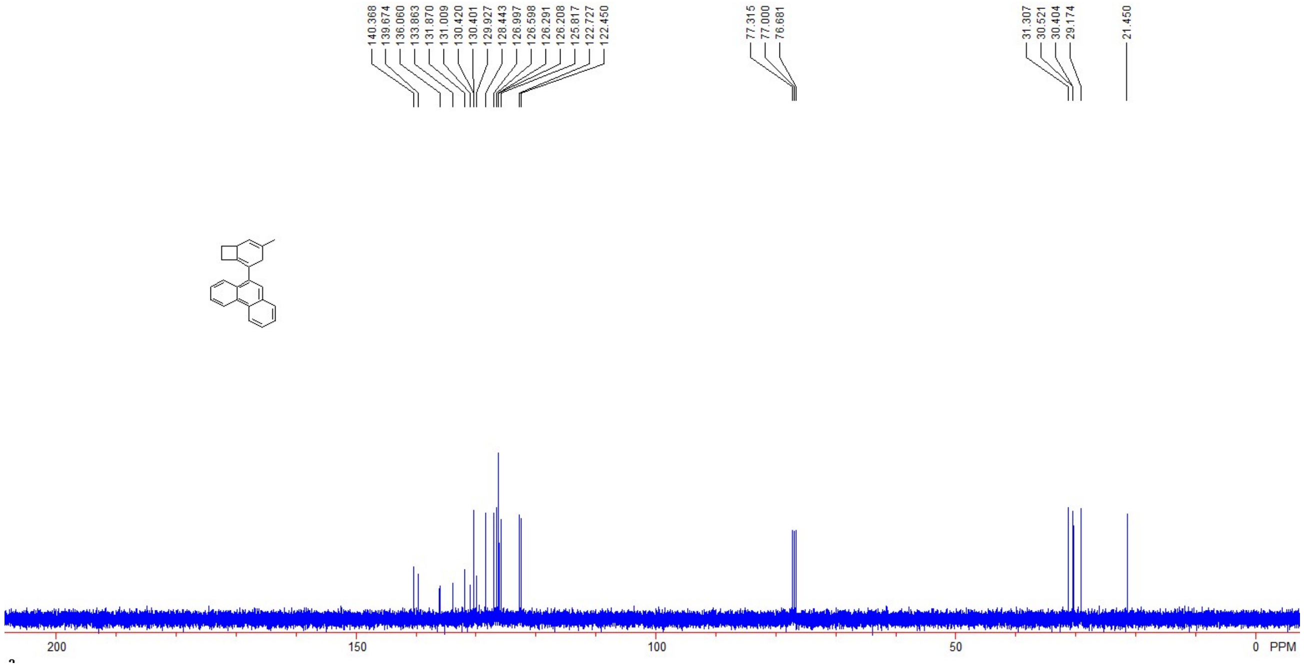

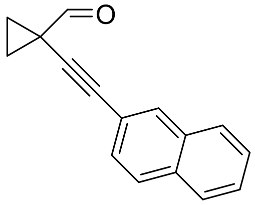

1-(Naphthalen-2-ylethynyl)cyclopropane-1-carbaldehyde s-7b:

Colorless oil. ^{1}H NMR (400 MHz, $CDCl_3$, TMS): δ 1.56-1.60 (m, 2H, CH_2), 1.62-1.66 (m, 2H, CH_2), 7.44-7.48 (m, 3H, Ar), 7.74-7.80 (m, 3H, Ar), 7.97 (s, 1H, Ar), 9.46 (s, 1H, CH=O). ^{13}C NMR (100 MHz, $CDCl_3$, TMS): δ 20.8, 24.4, 81.2, 87.9, 120.0, 126.5, 127.6, 127.7, 127.9, 128.5, 131.7, 132.7, 133.8, 198.1. IR (CH_2Cl_2) ν 3056, 2827, 1715, 1257, 1180, 1126, 895, 817, 746 cm^{-1}. MS (%) m/z 220 (M^+, 100.00), 191 (84.42), 176 (7.68), 165 (33.08), 152 (10.08), 115 (5.13), 110 (3.51), 82 (10.58), 63 (6.64). HRMS (EI) calcd. for $C_{16}H_{12}O$: 220.0888, found: 220.0892.

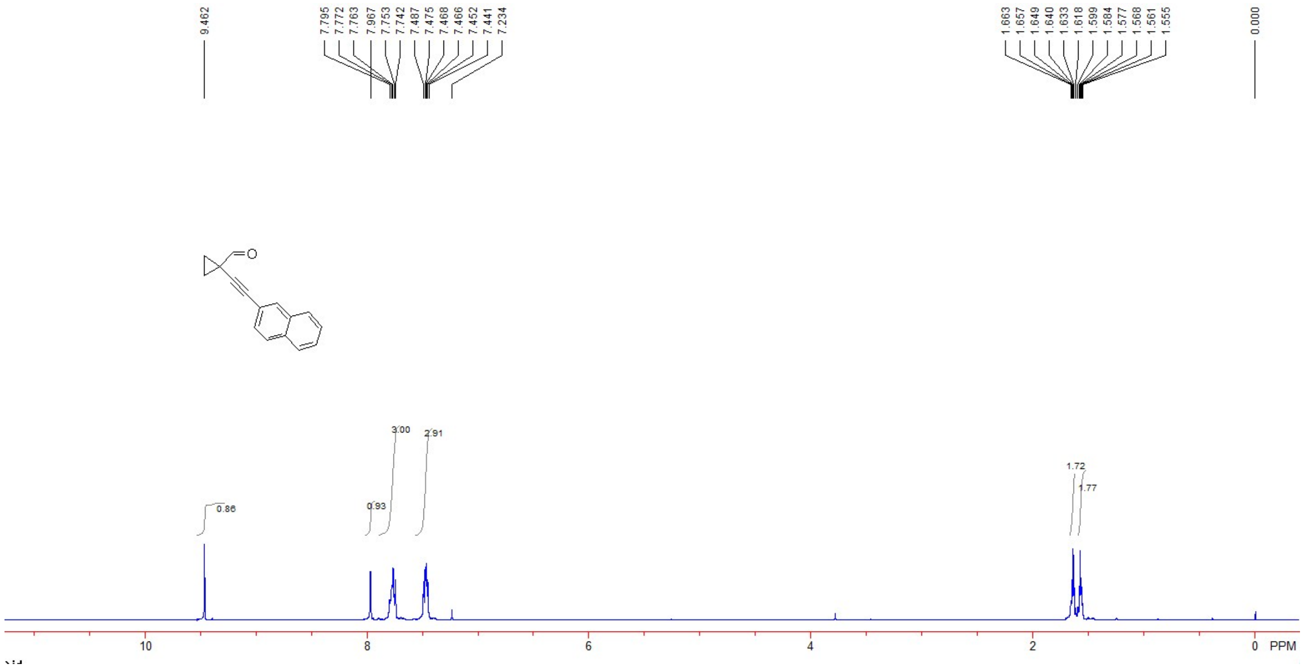

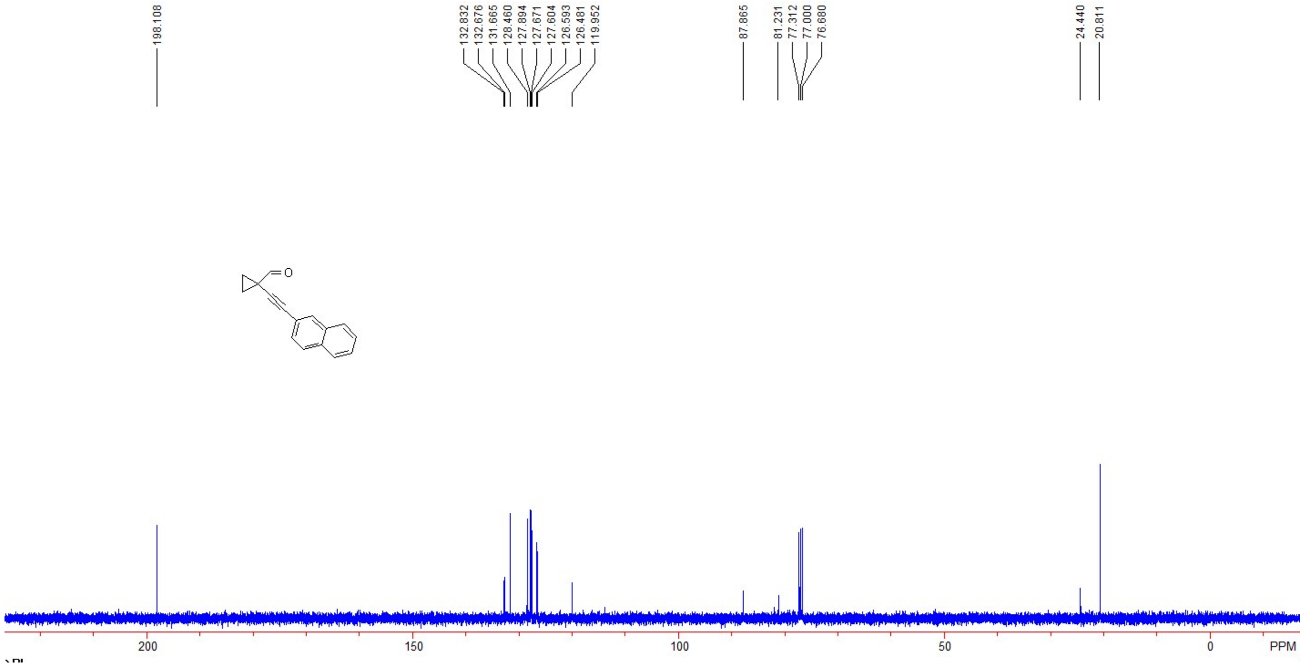

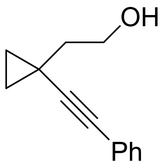

2-(1-(Phenylethynyl)cyclopropyl)ethan-1-ol s-9c:

Colorless oil. ^{1}H NMR (400 MHz, $CDCl_3$, TMS): δ 0.74 (dd, J_1 = 6.4 Hz, J_2 = 4.4 Hz, 2H, CH_2), 1.02 (dd, J_1 = 6.4 Hz, J_2 = 4.4 Hz, 2H, CH_2), 1.69 (t, J = 6.4 Hz, 2H, CH_2), 2.22 (s, 1H, OH), 3.93 (t, J = 6.4 Hz, 2H, CH_2), 7.24-7.28 (m, 3H, Ar), 7.34-7.37 (m, 2H, Ar). ^{13}C NMR (100 MHz, $CDCl_3$, TMS): δ 9.6, 15.3, 40.6, 61.9, 77.4, 94.1, 123.4, 127.6, 128.1, 131.5. IR (CH_2Cl_2) ν 3059. 2922, 2849, 1629, 1601, 1498, 1388, 1265, 1031, 850, 806 cm^{-1}. MS (%) m/z 186 (M^+, 26.28), 171 (36.63), 155 (94.88), 141 (66.23), 128 (73.51), 115 (100.00), 102 (22.93), 91 (26.91), 77 (58.21). HRMS (EI) calcd. for $C_{13}H_{14}O$: 186.1045, found: 186.1044.

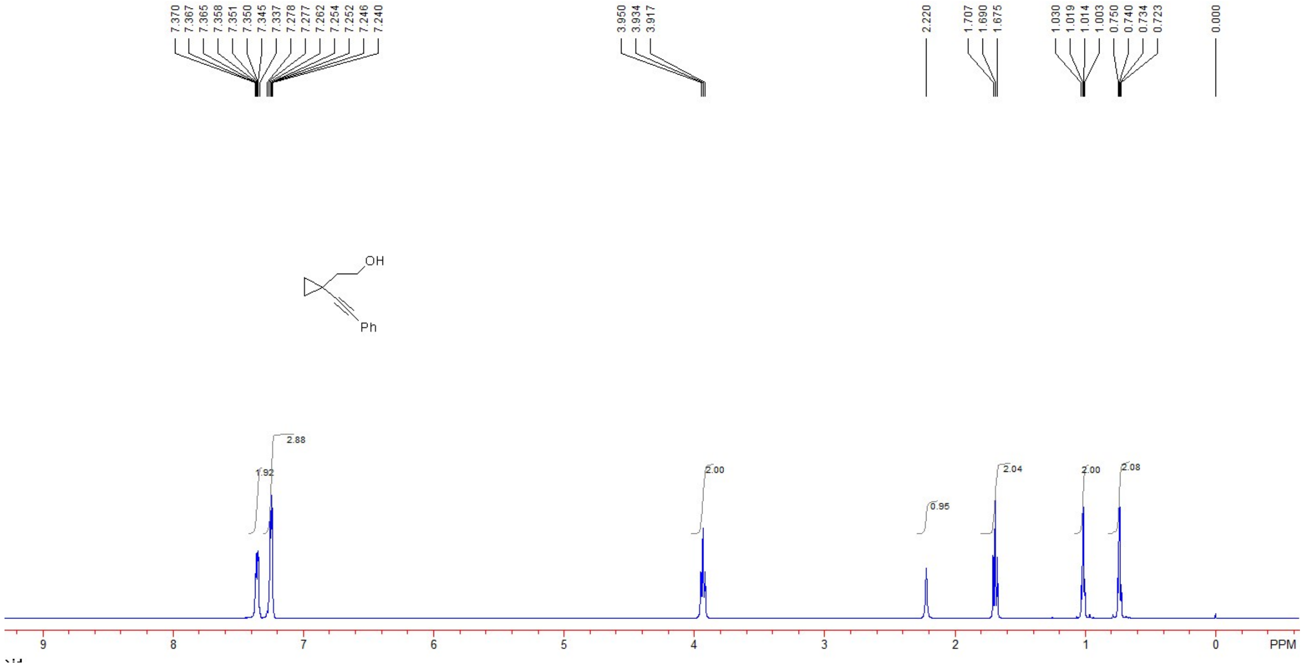

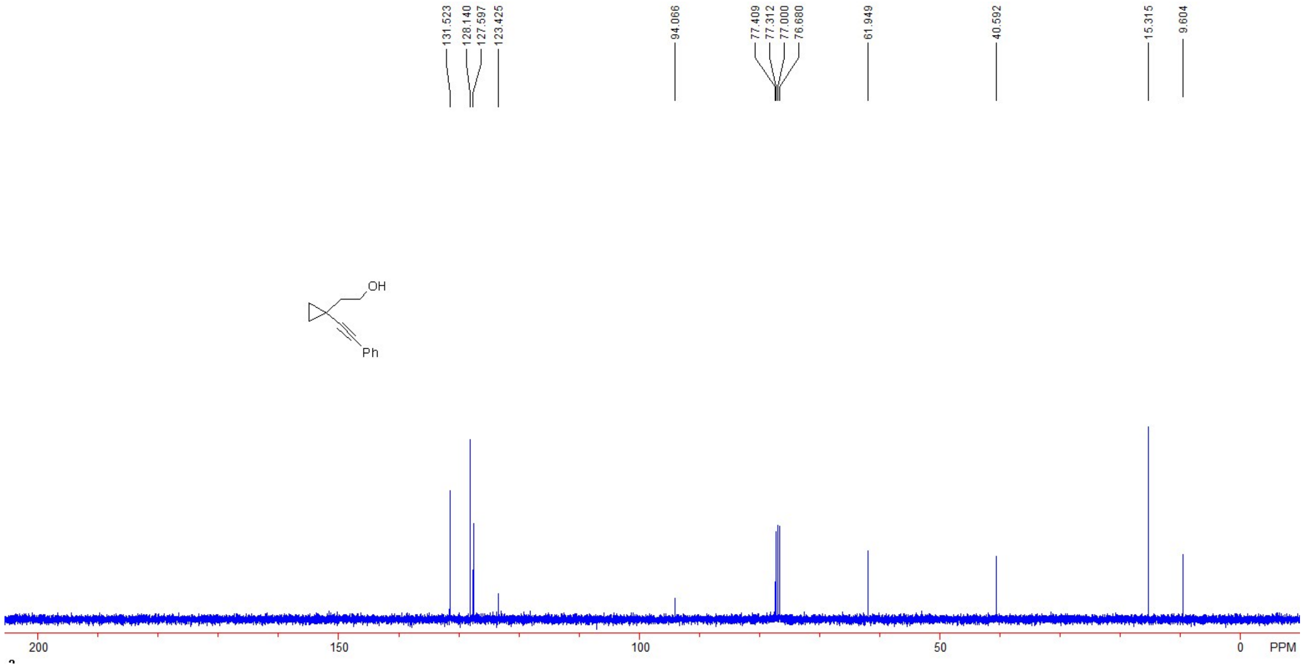

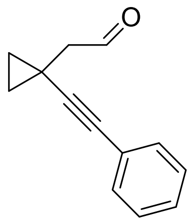

2-(1-(Phenylethynyl)cyclopropyl)acetaldehyde s-10c:

Colorless oil. ^{1}H NMR (400 MHz, $CDCl_3$, TMS): δ 0.84 (dd, J_1 = 6.0 Hz, J_2 = 4.4 Hz, 2H, CH_2), 1.17 (dd, J_1 = 6.4 Hz, J_2 = 4.8 Hz, 2H, CH_2), 2.44 (s, 2H, CH_2), 7.25-7.26 (m, 3H, Ar), 7.35-7.37 (m, 2H, Ar), 9.96 (t, J = 2.0 Hz, 1H, CH=O). ^{13}C NMR (100 MHz, $CDCl_3$, TMS): δ 7.6, 15.2, 51.0, 77.7,

92.7, 123.1, 122.8, 128.1, 131.6, 201.5. IR (CH_2Cl_2) ν 3059, 3011, 2919, 1703, 1491, 1280, 915, 755, 691 cm^{-1}. MS (%) m/z 184 (M^+, 22.96), 155 (81.54), 141 (100.00), 127 (51.33), 115 (77.22), 102 (26.95), 91 (17.22), 77 (46.51), 63 (13.49). HRMS (EI) calcd. for $C_{13}H_{12}O$: 184.0888, found: 184.0885.

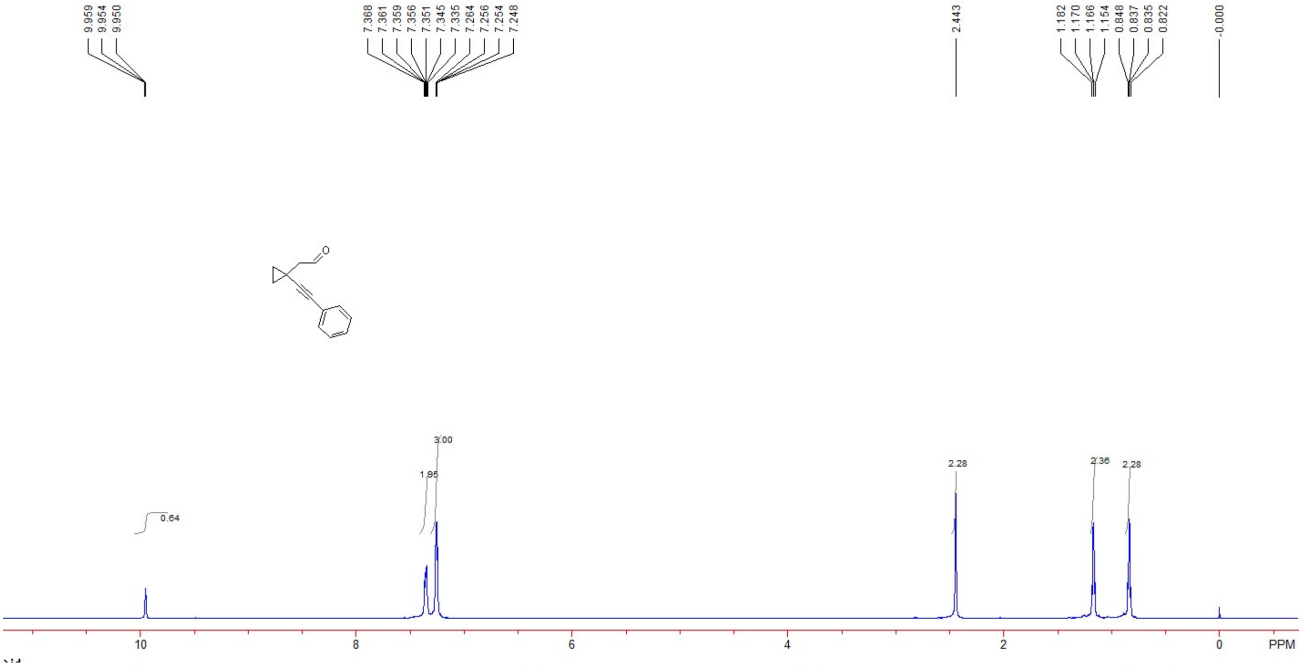

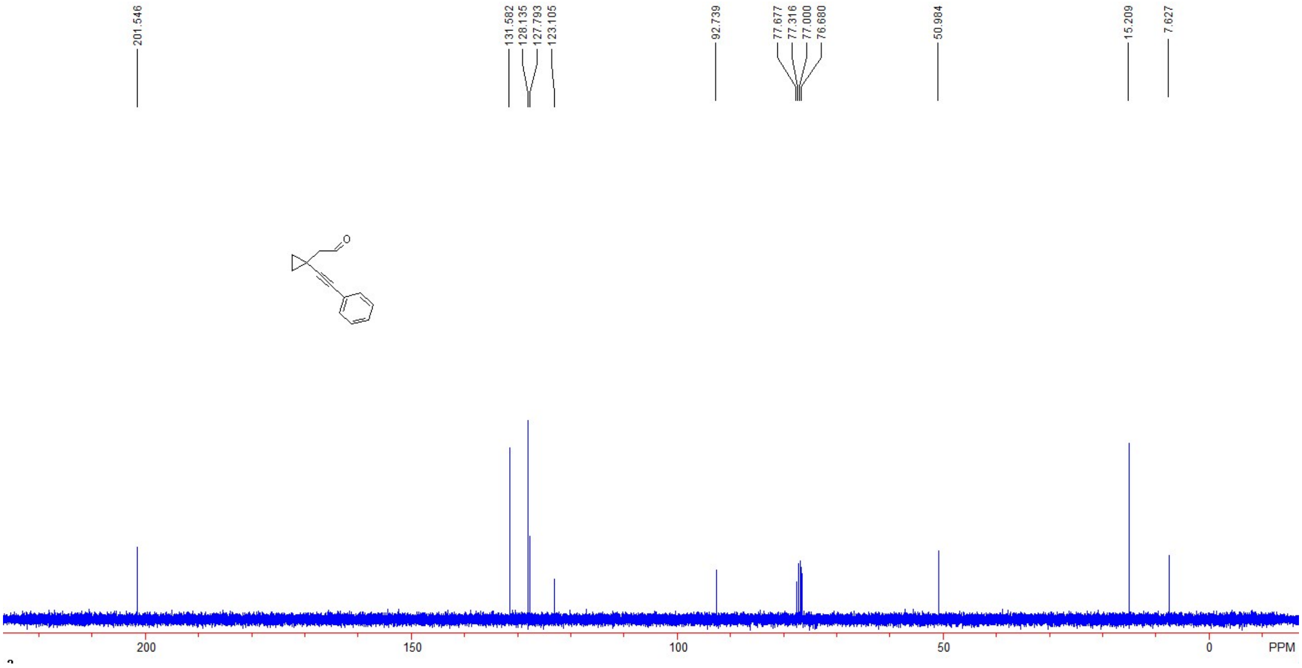

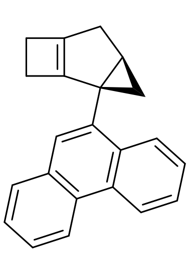

9-(Tricyclo[4.2.0.0^{2,4}]oct-1(6)-en-2-yl)phenanthrene 4a:

42 mg, yield = 75%. A white solid. Mp: 75-78 °C. ^{1}H NMR (400 MHz, $CDCl_3$, TMS): δ 0.82-0.87 (m, 1H, CH_2), 1.25-1.45 (m, 1H, CH_2), 2.10-2.15 (m, 1H, CH), 2.40-2.63 (m, 5H, CH_2), 2.90-2.94 (m, 1H, CH_2), 7.12-7.56 (m, 2H, Ar), 7.59-7.63 (m, 2H, Ar), 7.66 (s, 1H, Ar), 7.72-7.83 (m, 1H, Ar), 8.31-8.34 (m, 1H, Ar), 8.59 (d, J = 8.0 Hz, 1H, Ar), 8.65-8.68 (m, 1H, Ar). ^{13}C NMR (100 MHz, $CDCl_3$, TMS): δ 24.1, 26.7, 28.3, 28.5, 35.2, 37.9, 122.4, 122.8, 126.1, 126.45, 126.54, 126.7, 128.2, 129.8, 130.6, 131.8, 132.8, 135.5, 145.1, 156.2. IR (CH_2Cl_2) ν 3056, 2917, 2836, 1705, 1264, 895, 735, 726, 703 cm^{-1}. MS (%) m/z 282 (100.00), 267 (29.68), 254 (100.00), 239 (12.58), 191 (73.03), 178 (7.43), 165 (10.31), 126 (32.74), 120.12.18. HRMS (EI) calcd. for $C_{22}H_{18}$: 282.1409, found: 282.1414.

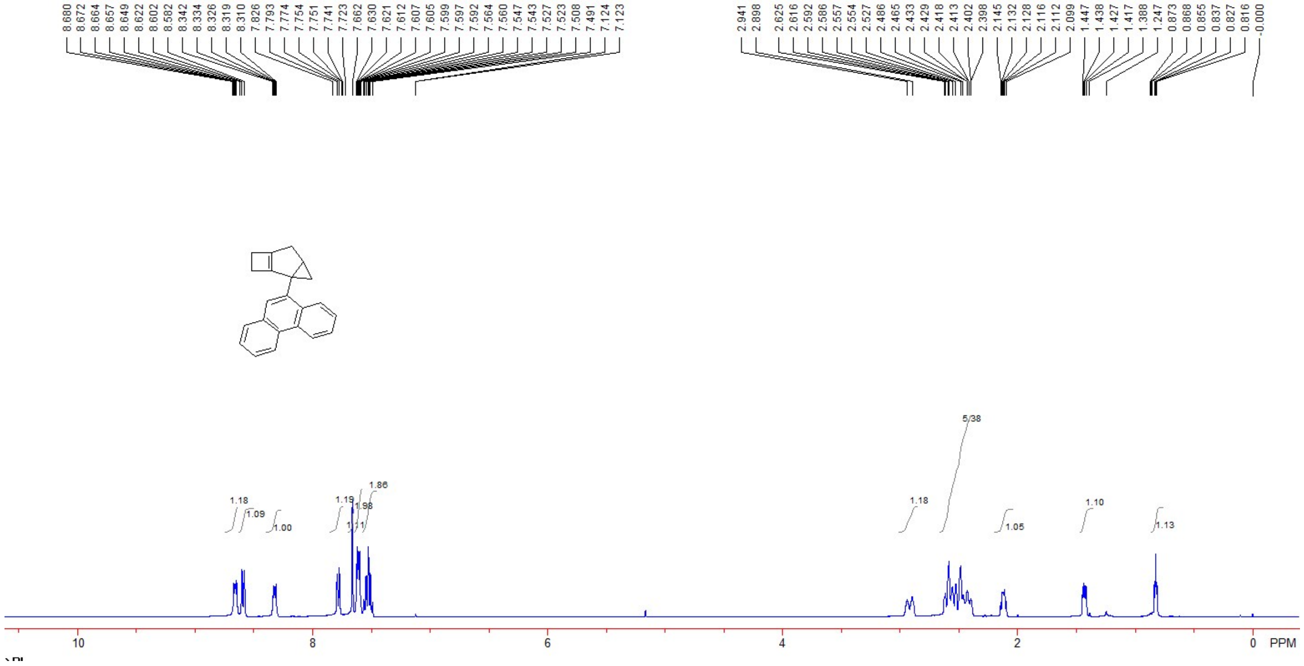

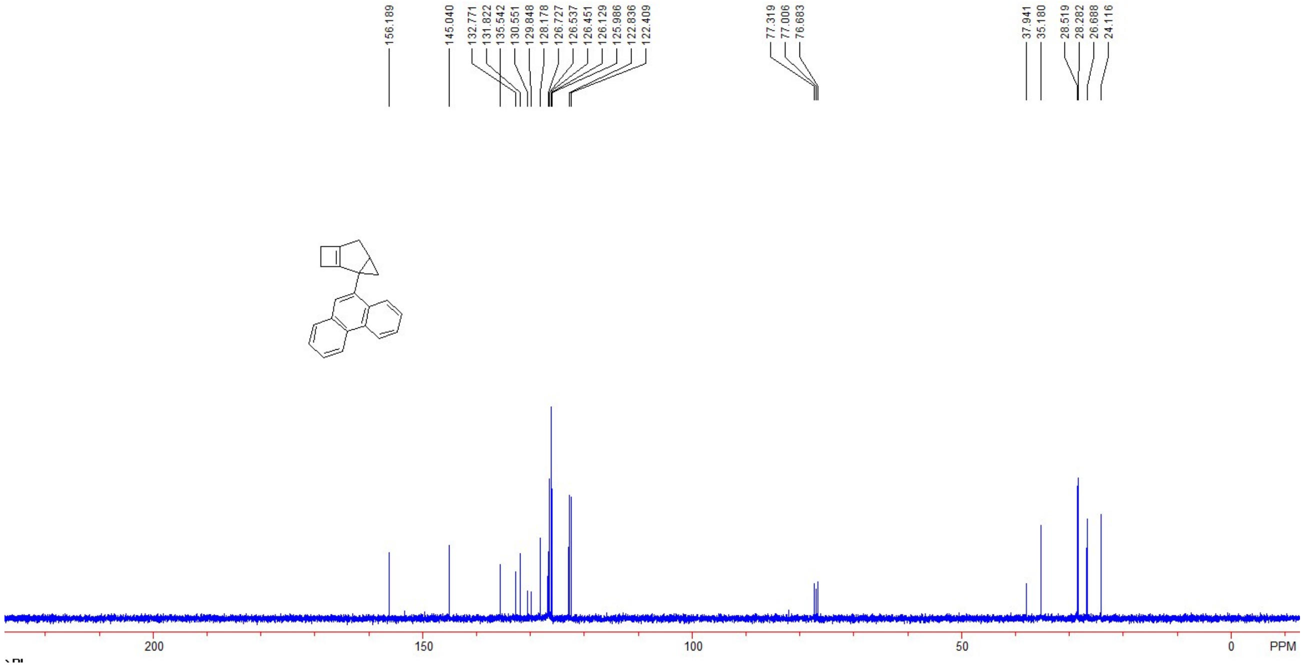

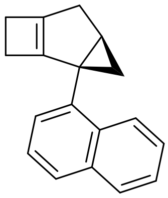

2-(Naphthalen-1-yl)tricyclo[4.2.0.0^{2,4}]oct-1(6)-ene 4b:

20 mg, yield = 43%. Colorless oil. ^{1}H NMR (400 MHz, $CDCl_3$, TMS): δ 0.78-0.80 (m, 1H, CH_2), 1.39-1.42 (m, 1H, CH_2), 2.03-2.07 (m, 1H, CH), 2.39-2.63 (m, 5H, CH_2), 2.87-2.92 (m, 1H, CH_2), 7.23-7.42 (m, 2H, Ar), 7.45-7.52 (m, 2H, Ar), 7.72 (d, J = 8.0 Hz, 1H, Ar), 7.83-7.85 (m, 1H, Ar), 8.22-8.24 (m, 1H, Ar). ^{13}C NMR (100 MHz, $CDCl_3$, TMS): δ 23.9, 26.7, 28.3, 28.4, 35.2, 37.6, 125.35, 125.37, 125.5, 125.7, 126.2, 127.1, 128.3, 133.7, 133.8, 137.3, 144.8, 156.4. IR (CH_2Cl_2) ν 3044, 2917, 2836, 1707, 1594, 1402, 793, 776 cm^{-1}. MS (%) m/z 232 (51.60), 215 (59.42), 204 (100.00), 189 (12.28), 141 (60.65), 128 (8.32), 117 (12.50), 101 (33.90), 95 (12.76). HRMS (EI) calcd. for $C_{18}H_{16}$: 232.1252, found: 232.1251.

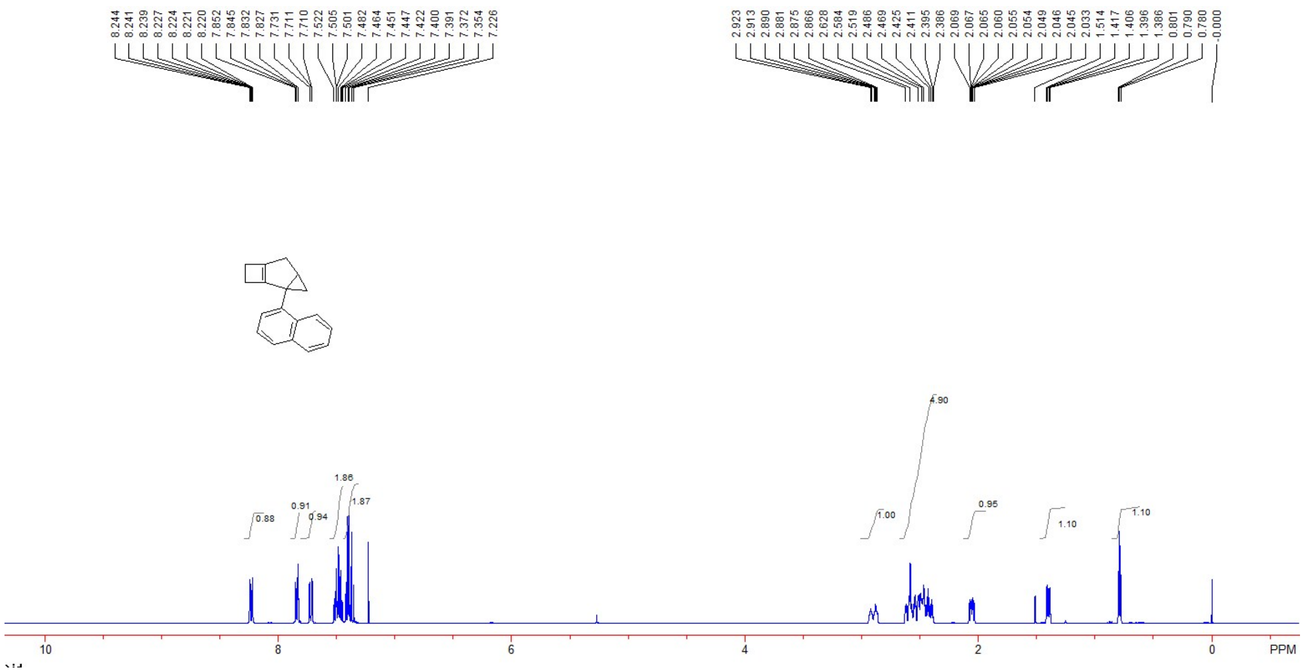

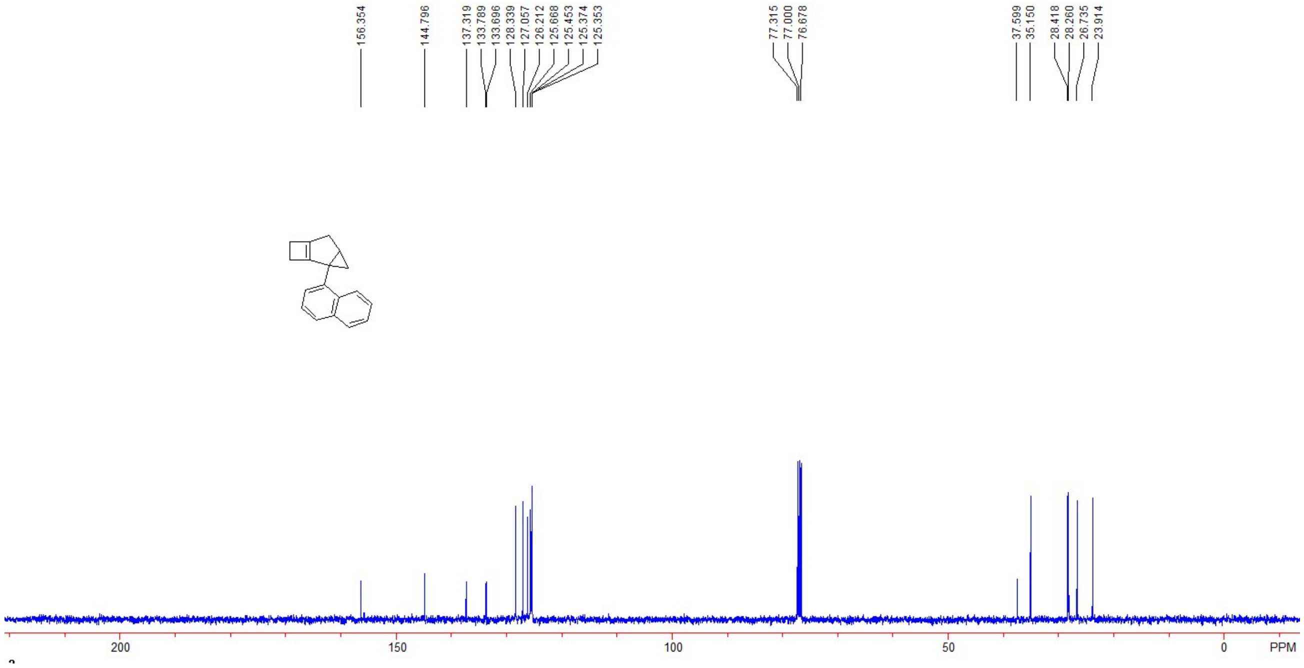

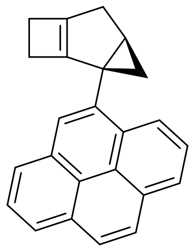

4-(tricyclo[4.2.0.0^{2,4}]oct-1(6)-en-2-yl)pyrene 4c:

46 mg, 75%. White solid, mp: 91-93 ^{o}C. ^{1}H NMR (400 MHz, $CDCl_3$, TMS): δ 0.92-0.95 (m, 1H, CH_2), 1.54-1.57 (m, 1H, CH_2), 2.17-2.22 (m, 1H, CH), 2.41-2.45 (m, 1H, CH_2), 2.50-2.67 (m, 4H,

CH$_2$), 2.97-3.01 (m, 1H, CH$_2$), 7.93-7.98 (m, 4H, Ar), 8.05-8.15 (m, 4H, Ar), 8.48 (d, J = 9.2 Hz, 1H, Ar). ^{13}C NMR (100 MHz, CDCl$_3$, TMS): δ 24.4, 27.5, 28.2, 28.4, 35.3, 38.1, 124.5, 124.74, 124.76, 124.85, 125.8, 126.8, 127.1, 127.2, 127.4, 130.2, 130.9, 131.0, 131.3, 135.0, 144.8, 156.6. IR (CH_2Cl_2) ν 3039, 2916, 2835, 1435, 1080, 845, 801, 718, 682 cm^{-1}. MS (%) m/z 306 (M$^+$, 100.00), 289 (56.23), 277 (61.14), 263 (25.25), 239 (33.43), 226 (13.19), 145 (33.92), 138 (32.92), 132 (13.46). HRMS (EI) calcd. for $C_{24}H_{18}$: 306.1409, found: 306.1406.

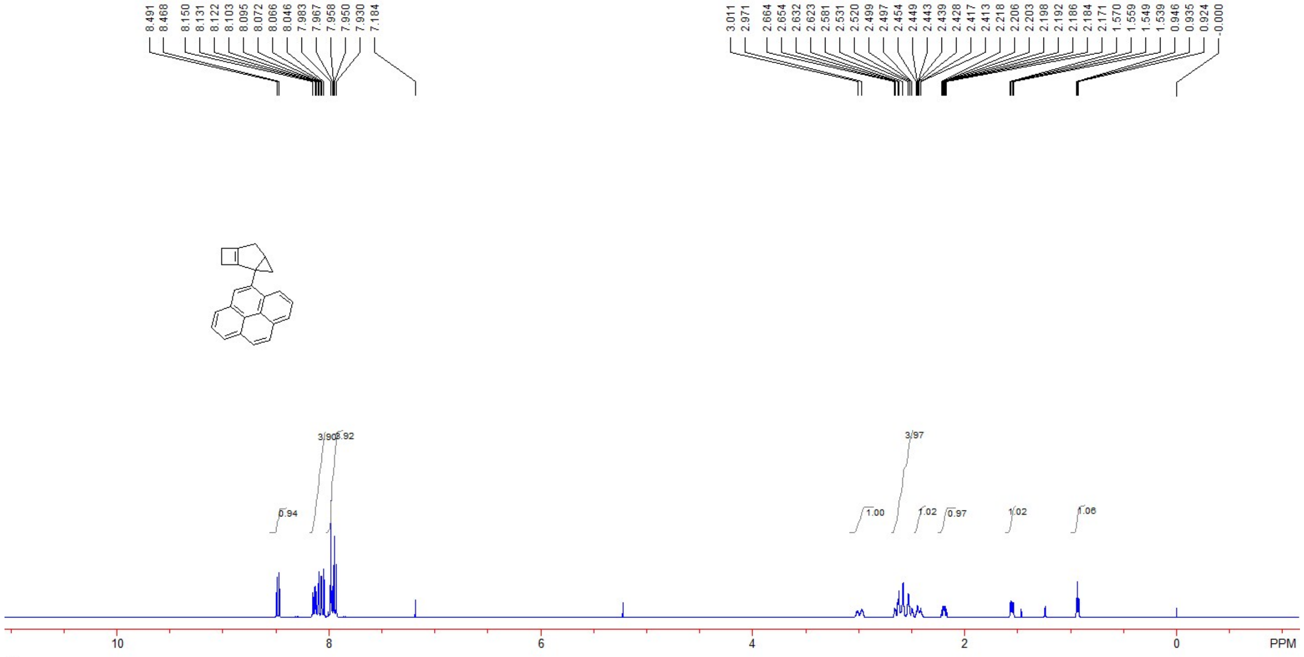

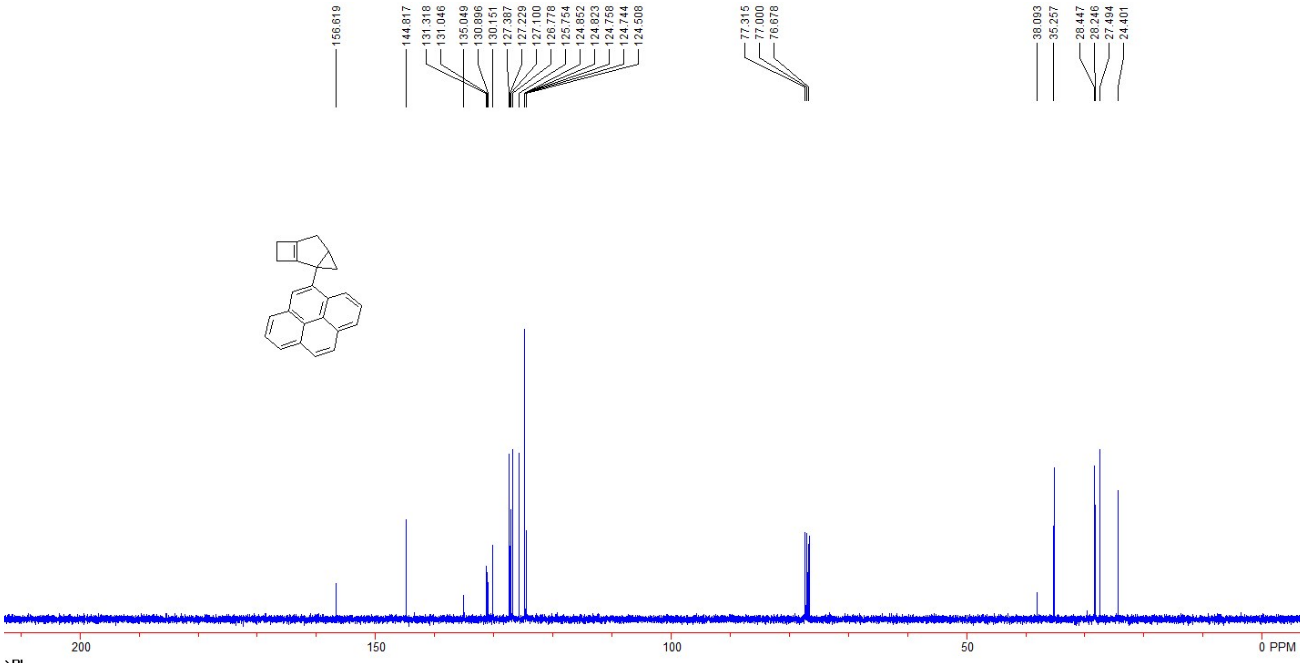

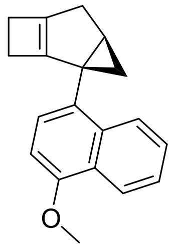

2-(4-Methoxynaphthalen-1-yl)tricyclo[4.2.0.02,4]oct-1(6)-ene 4d:

32 mg, yield = 62%. Colorless oil. ^{1}H NMR (400 MHz, $CDCl_3$, TMS): δ 0.74 (br, 1H, CH_2), 1.34-1.37 (m, 1H, CH_2), 1.96-2.00 (m, 1H, CH), 2.39-2.60 (m, 5H, CH_2), 2.86-2.90 (m, 1H, CH_2), 3.92 (s, 3H, CH_3), 6.66 (d, J = 8.0 Hz, 1H, Ar), 7.28 (d, J = 8.0 Hz, 1H, Ar), 7.44-7.52 (m, 2H, Ar), 8.17 (d, J = 8.4 Hz, 1H, Ar), 8.27 (d, J = 8.4 Hz, 1H, Ar). ^{13}C NMR (100 MHz, $CDCl_3$, TMS): δ 23.9, 26.6, 28.2, 28.4, 35.1, 37.1, 55.4, 103.1, 122.2, 124.8, 125.1, 125.7, 126.1, 126.2, 129.2, 134.5, 144.4, 154.5, 156.6. IR (CH_2Cl_2) ν 3071, 2916, 2835, 1585, 1463, 1234, 1083, 1026, 763 cm^{-1}. MS (%) m/z 262 (M^+, 93.59), 247 (39.29), 231 (100.00), 215 (37.92), 202 (31.67), 189 (19.55), 178 (13.94), 116 (12.57), 101 (15.52). HRMS (EI) calcd. for $C_{19}H_{18}O$: 262.1358, found: 262.1352.

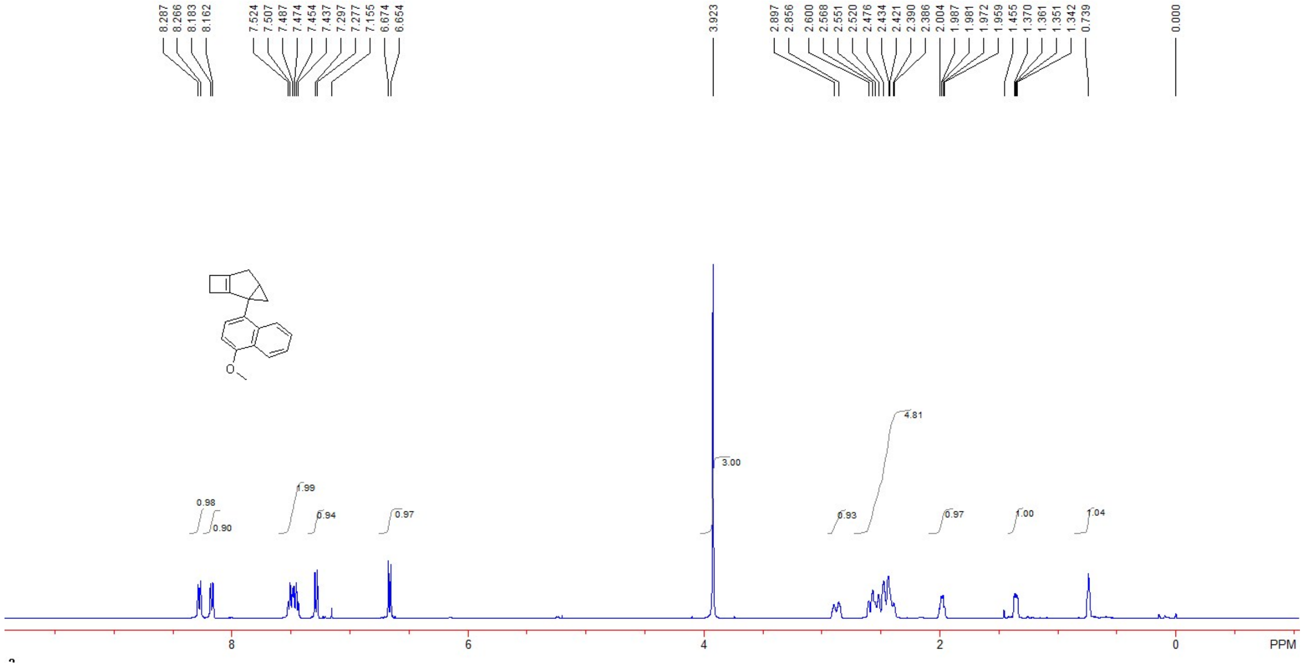

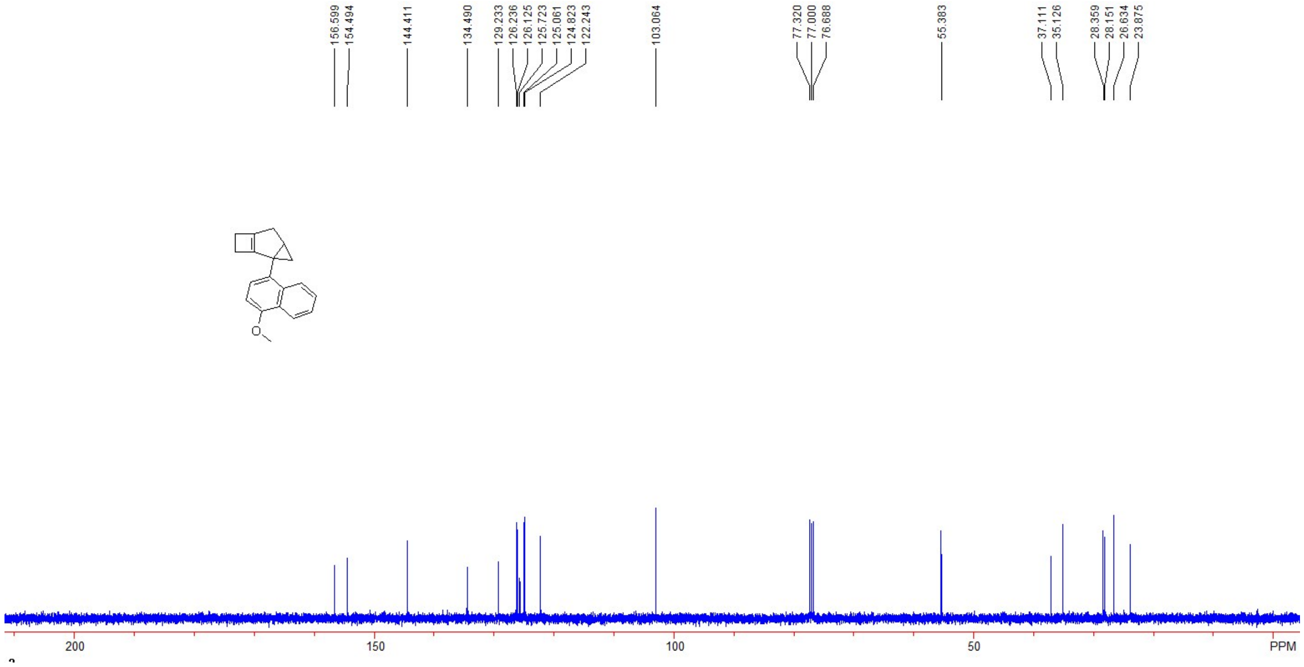

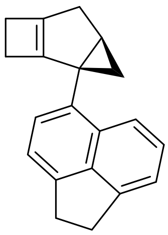

5-(tricyclo[4.2.0.0^{2,4}]oct-1(6)-en-2-yl)-1,2-dihydroacenaphthylene 4e:

27 mg, yield = 52%. Colorless oil. ^{1}H NMR (400 MHz, $CDCl_3$, TMS): δ 0.71-0.74 (m, 1H, CH_2), 1.42-1.45 (m, 1H, CH_2), 1.94-1.99 (m, 1H, CH), 2.43-2.49 (m, 3H, CH_2), 2.53-2.63 (m, 2H, CH_2), 2.86-2.91 (m, 1H, CH_2), 3.32-3.28 (m, 4H, CH_2), 7.16 (d, J = 7.2 Hz, 1H, Ar), 7.26 (d, J = 6.8 Hz, 1H, Ar), 7.36 (d, J = 6.8 Hz, 1H, Ar), 7.44 (dd, J_1 = 8.0 Hz, J_2 = 7.6 Hz, 1H, Ar), 7.82 (d, J = 8.0 Hz, 1H, Ar). ^{13}C NMR (100 MHz, $CDCl_3$, TMS): δ 23.3, 26.9, 28.3, 28.4, 29.9, 30.6, 35.2, 36.9, 118.8, 119.1, 120.6, 127.47, 127.49, 132.1, 133.0, 139.5, 144.72, 144.73, 146.1, 156.3. IR (CH_2Cl_2) ν 3030, 2915, 2836, 1608, 1424, 1075, 907, 838, 770, 729 cm^{-1}. MS (%) m/z 258 (M^+, 87.95), 257 (100.00), 243 (47.74), 229 (75.40), 215 (28.04), 202 (21.62), 189 (20.93), 152 (15.00), 120 (13.80), 101 (12.10). HRMS (EI) calcd. for $C_{20}H_{18}$: 258.1409, found: 258.1404.

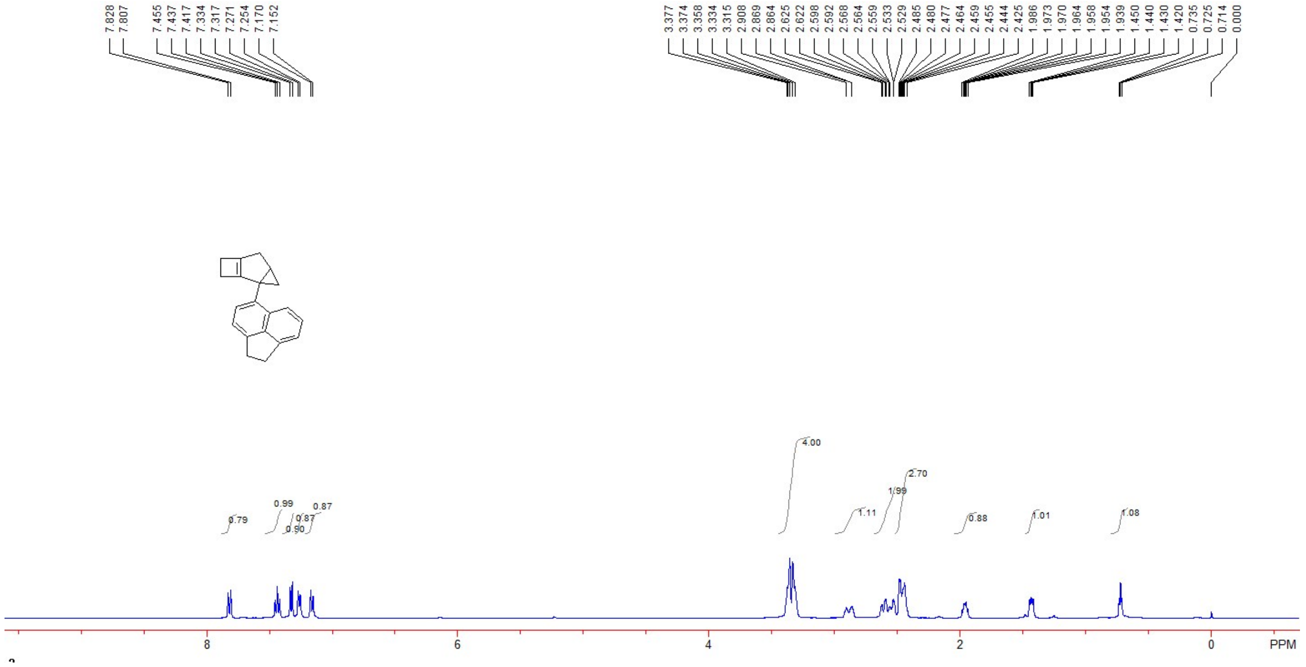

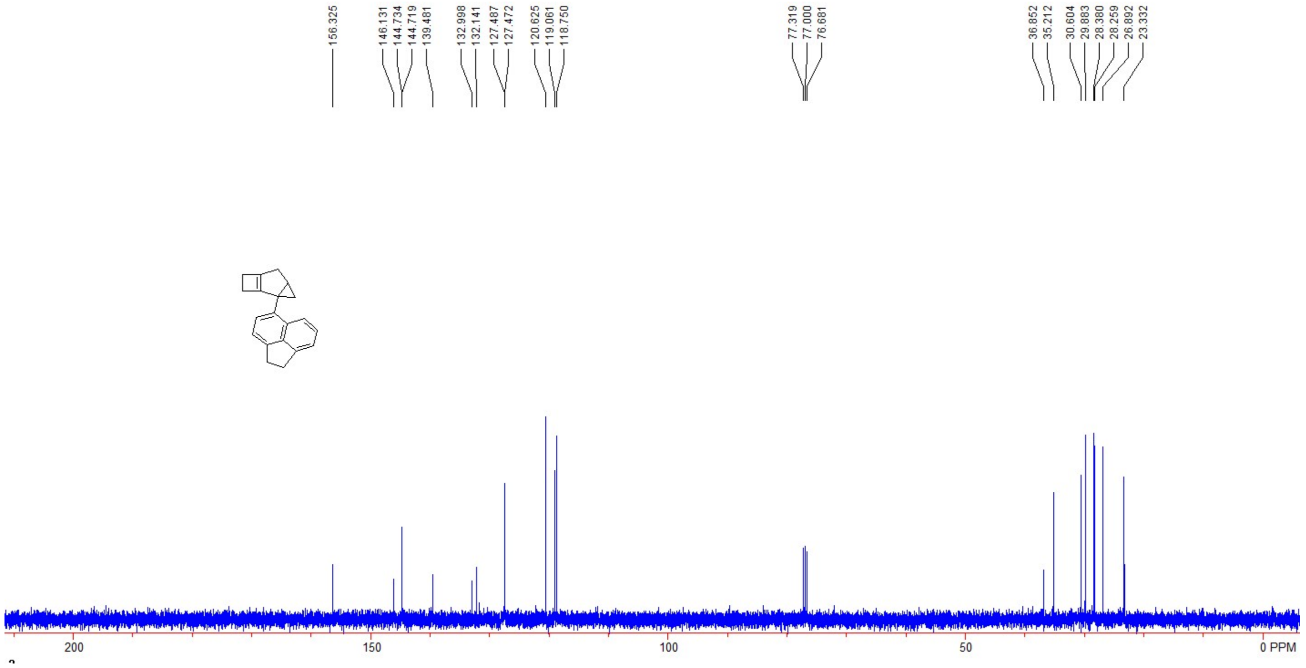

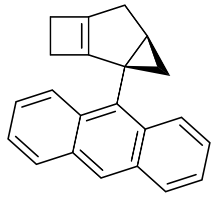

9-(tricyclo[4.2.0.0^{2,4}]oct-1(6)-en-2-yl)anthracene 4f:

30 mg, yield = 52%. Colorless oil. ^{1}H NMR (400 MHz, $CDCl_3$, TMS): δ 1.16-1.18 (m, 1H, CH_2), 1.36-1.39 (m, 1H, CH_2), 2.13-2.18 (m, 1H, CH), 2.38-2.42 (m, 1H, CH_2), 2.51-2.65 (m, 4H, CH_2), 3.07-3.12 (m, 1H, CH_2), 7.41-7.51 (m, 4H, Ar), 7.97 (d, J = 8.0 Hz, 2H, Ar), 8.27 (d, J = 8.8 Hz, 1H,

Ar), 8.34 (s, 1H, Ar), 8.70 (d, J = 8.8 Hz, 1H, Ar). ^{13}C NMR (100 MHz, $CDCl_3$, TMS): δ 26.6, 28.1, 28.4, 29.2, 34.1, 34.9, 124.8, 125.16, 125.22, 125.7, 126.0, 126.4, 128.8, 131.5, 131.6, 131.9, 132.9, 144.6, 156.9. IR (CH_2Cl_2) ν 3050, 2917, 2835, 1673, 1441, 1285, 885, 733, 700 cm^{-1}. MS (%) m/z 282 (M^+, 100.00), 267 (34.25), 253 (47.29), 239 (51.17), 215 (24.50), 202 (13.87), 126 (25.52), 120 (13.53), 113 (11.94). HRMS (EI) calcd. for $C_{22}H_{18}$: 282.1409, found: 282.1400.

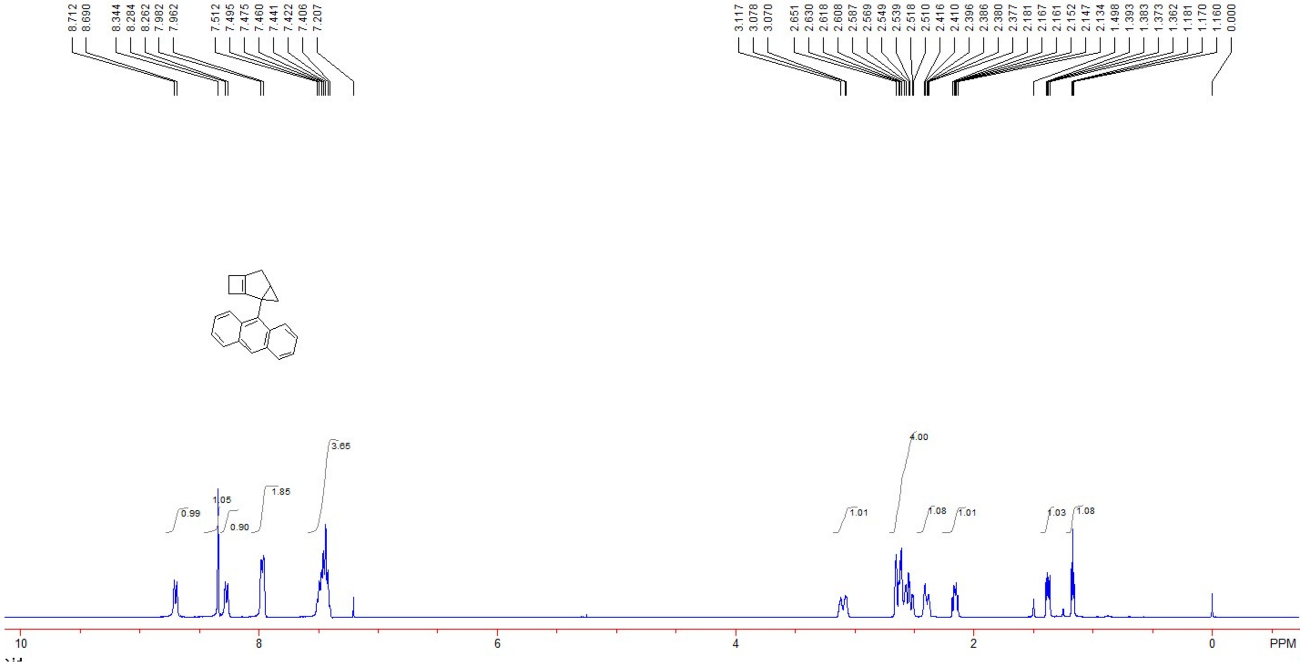

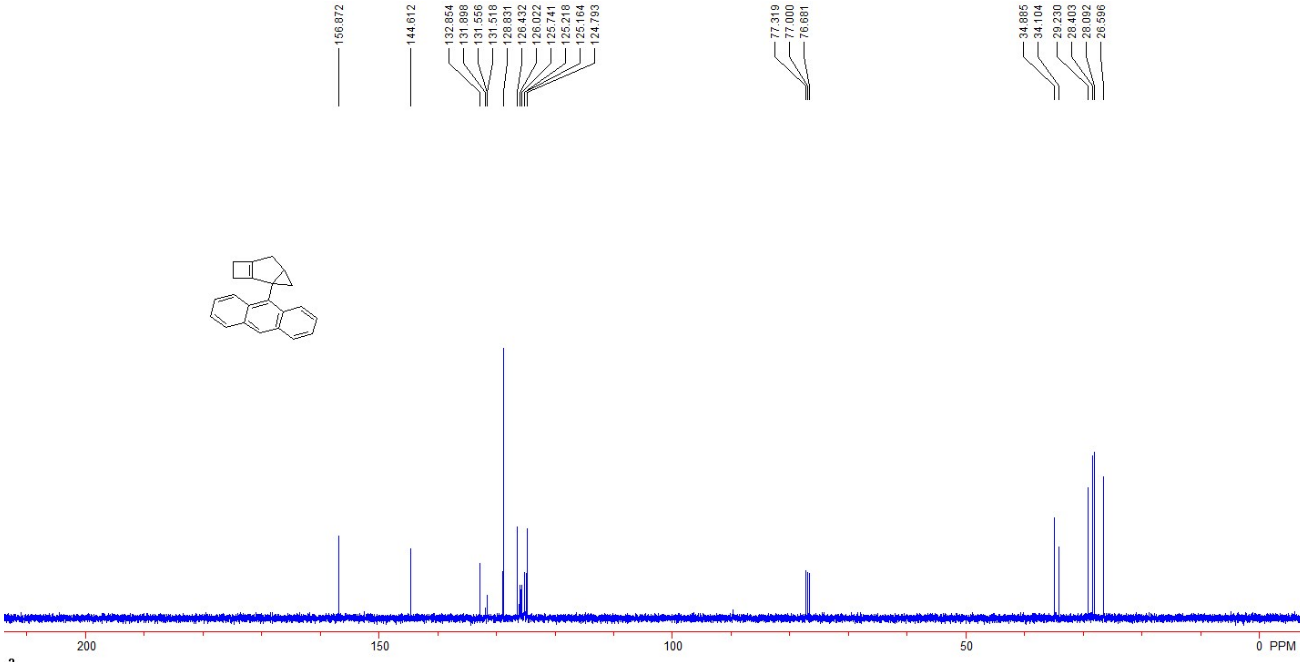

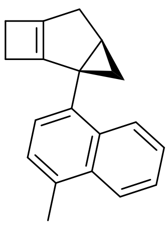

2-(4-Methylnaphthalen-1-yl)tricyclo[4.2.0.02,4]oct-1(6)-ene 4g:

30 mg, yield = 60%. Colorless oil. ^{1}H NMR (400 MHz, $CDCl_3$, TMS): δ 0.76-0.78 (m, 1H, CH_2), 1.36-1.39 (m, 1H, CH_2), 2.01-2.06 (m, 1H, CH), 2.38-2.73 (m, 5H, CH_2), 2.69 (s, 3H, CH_3), 7.22 (d, J = 7.6 Hz, 1H, Ar), 7.31 (d, J = 7.2 Hz, 1H, Ar), 7.31-7.53 (m, 2H, Ar), 7.99-8.01 (m, 1H, Ar), 8.25-8.28 (m, 1H, Ar). ^{13}C NMR (100 MHz, $CDCl_3$, TMS): δ 19.4, 24.0, 26.7, 28.2, 28.4, 35.2, 37.5, 124.5, 125.3, 125.4, 125.9, 126.0, 126.1, 132.8, 133.1, 133.8, 135.1, 144.6, 156.5. IR (CH_2Cl_2) ν 3062, 2918, 2837, 1705, 1440, 1024, 906, 832, 757, 728 cm^{-1}. MS (%) m/z 246 (M^+, 28.21), 231 (100.00), 215 (38.02), 189 (11.84), 178 (8.78), 152 (7.67), 108 (14.43), 95 (1.02). HRMS (EI) calcd. for $C_{19}H_{18}$: 246.1409, found: 246.1405.

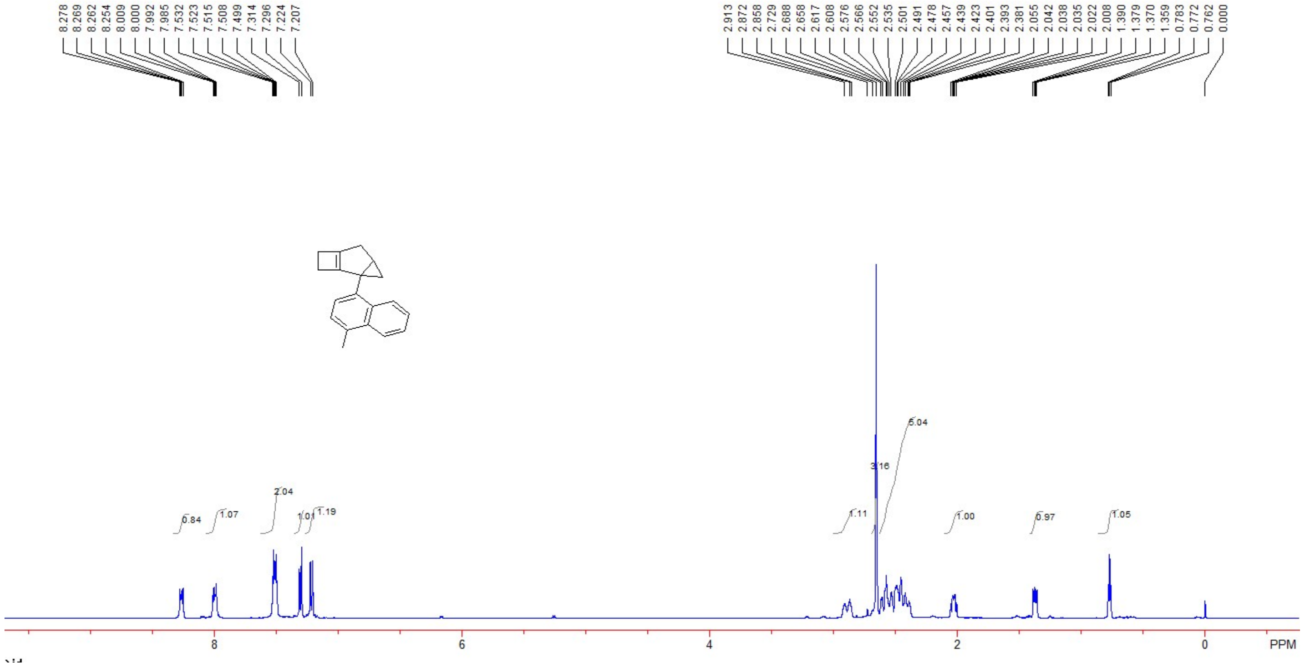

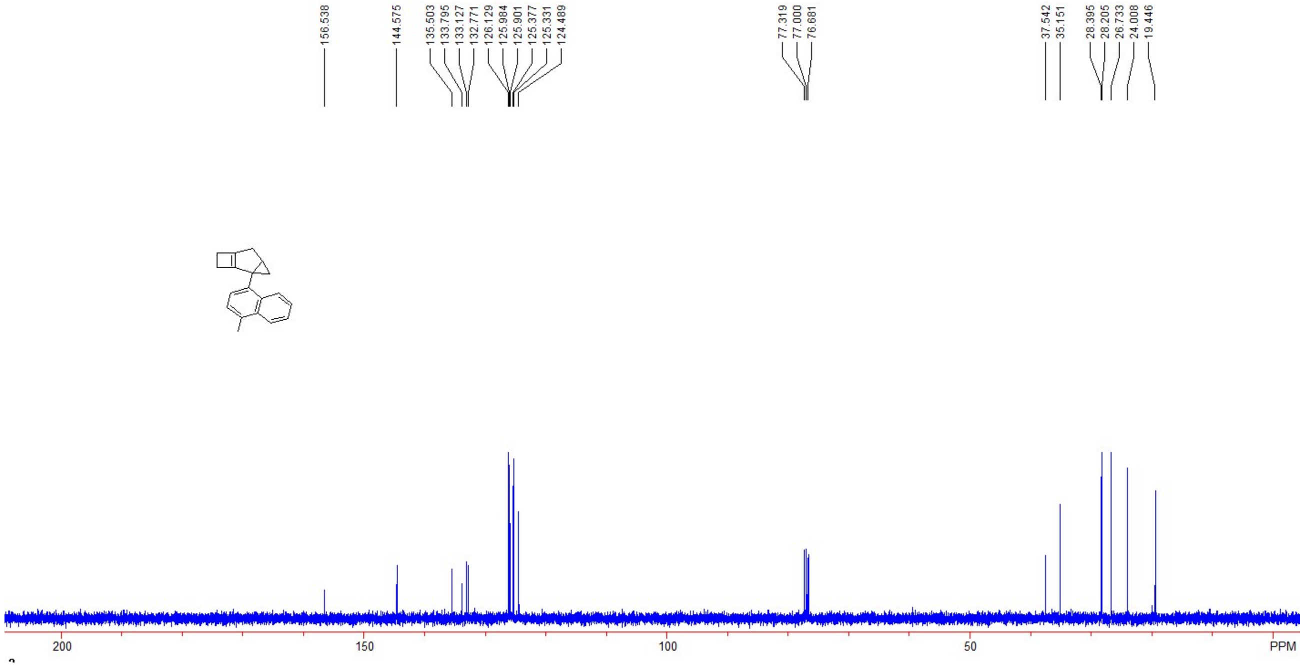

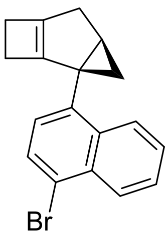

2-(4-Bromonaphthalen-1-yl)tricyclo[4.2.0.02,4]oct-1(6)-ene 4h:

36 mg, yield = 58%. Colorless oil. ^{1}H NMR (400 MHz, $CDCl_3$, TMS): δ 0.79-0.81 (m, 1H, CH_2), 1.36-1.39 (m, 1H, CH_2), 2.03-2.07 (m, 1H, CH), 2.37-2.42 (m, 1H, CH_2), 2.46-2.63 (m, 4H, CH_2), 2.86-2.91 (m, 1H, CH_2), 7.27 (dd, J_1 = 8.8 Hz, J_2 = 8.0 Hz, 1H, Ar), 7.53-7.61 (m, 2H, Ar), 7.68 (d, J = 7.6 Hz, 1H, Ar), 8.24-8.27 (m, 2H, Ar). ^{13}C NMR (100 MHz, $CDCl_3$, TMS): δ 24.0, 26.9, 28.2, 28.4, 35.1, 37.4, 121.6, 125.8, 126.5, 126.8, 126.9, 127.6, 129.4, 131.9, 135.0, 145.1, 155.9. IR (CH_2Cl_2) ν 3066, 2920, 2831, 1706, 1505, 1380, 907, 759, 730 cm^{-1}. MS (%) m/z 310 (M^+, 17.75), 231 (100.00), 215 (81.54), 202 (46.42), 189 (24.63), 178 (9.55), 163 (14.85), 152 (10.37), 108 (23.78), 95 (12.18). HRMS (EI) calcd. for $C_{18}H_{15}Br$: 310.0357, found: 310.0356.

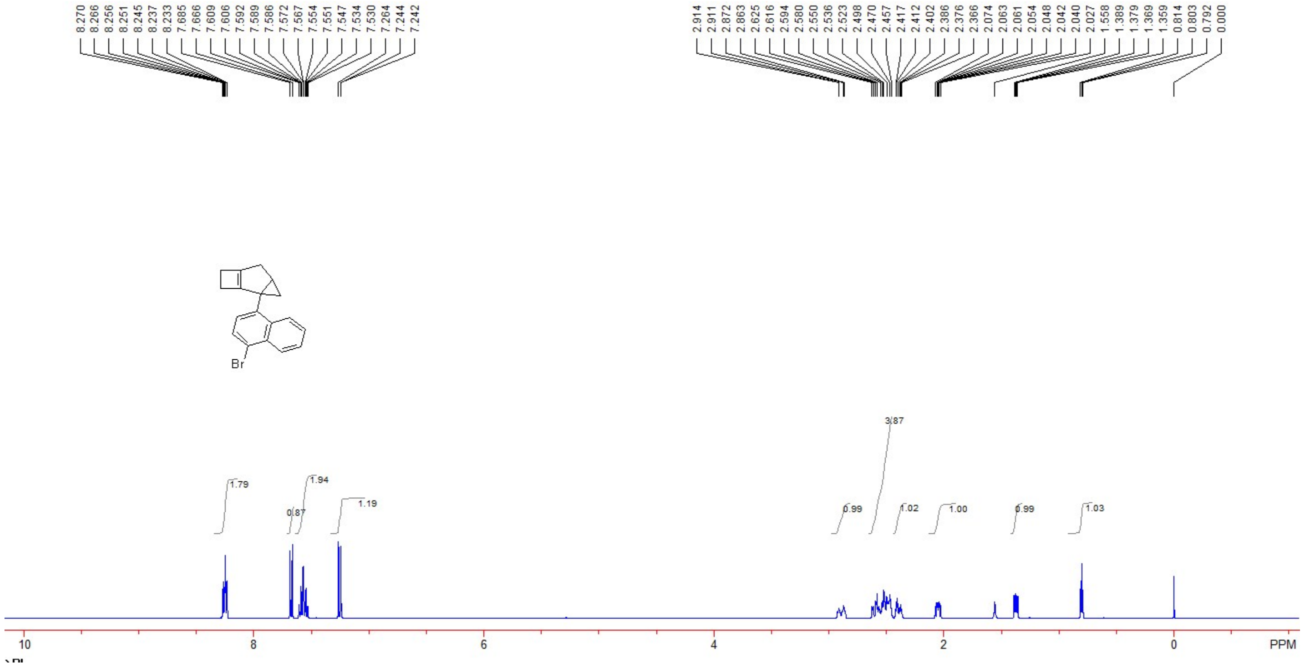

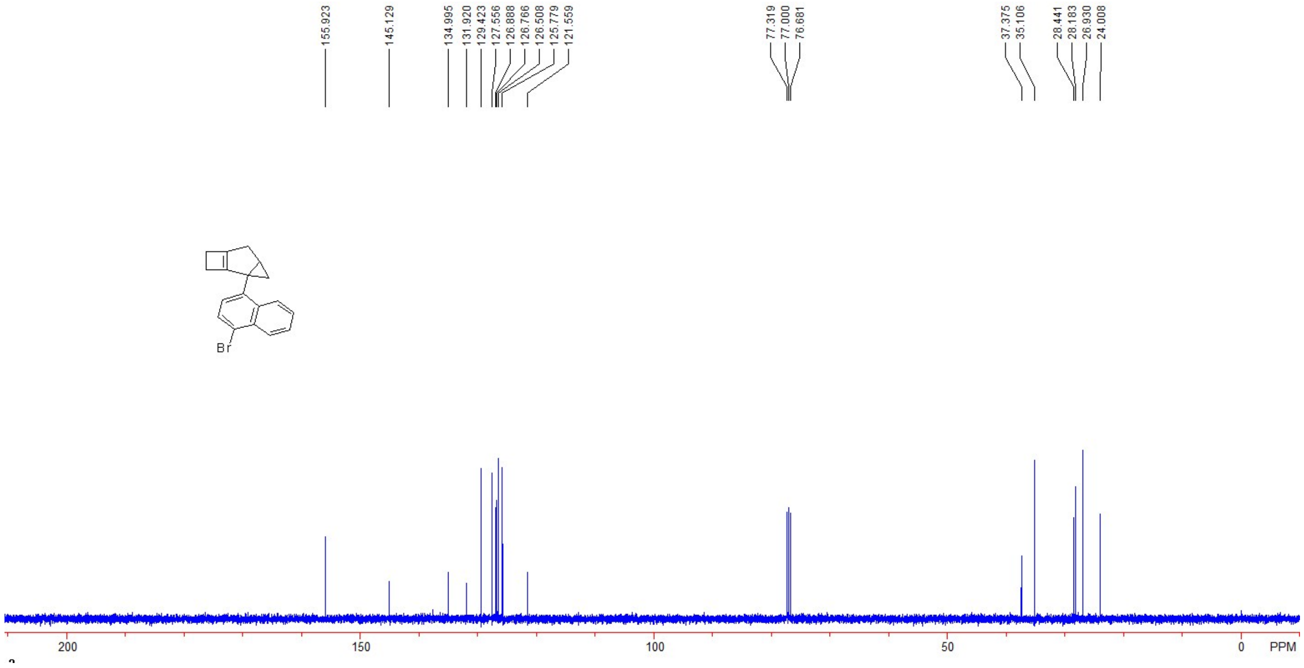

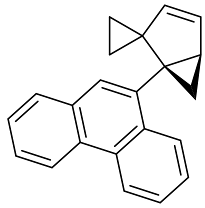

9-(Spiro[bicyclo[3.1.0]hexane-2,1'-cyclopropan]-3-en-1-yl)phenanthrene 5a:

47 mg, yield = 84%. A white solid. Mp: 87-89 ^{o}C (Mixtures of two diastereomers, dr = 14.6/1). ^{1}H NMR (400 MHz, $CDCl_3$, TMS): δ 0.12-0.17 (m, 1H, CH_2), 0.59-0.62 (m, 1H, CH_2), 0.63-0.74 (m,

2H, CH_2), 0.85-0.87 (m, 1H, CH_2), 1.53-1.55 (m, 1H, CH_2), 2.23-2.26 (m, 1H, CH), 5.29 (d, J = 5.6 Hz, 1H, =CH), 6.19 (dd, J_1 = 5.6 Hz, J_2 = 2.0 Hz, 1H, =CH), 7.18-7.59 (m, 2H, Ar), 7.61-7.64 (m, 3H, Ar), 7.80-7.82 (m, 1H, Ar), 8.16-8.18 (m, 1H, Ar), 8.62 (d, J = 8.0 Hz , 2H, Ar), 8.65-8.68 (m, 1H, Ar). ^{13}C NMR (100 MHz, $CDCl_3$, TMS): δ 10.6, 14.2, 23.8, 32.6, 35.96, 35.98, 122.5, 122.7, 126.1, 136.3, 126.4, 126.5, 127.2, 128.3, 128.5, 130.1, 130.4, 131.6, 131.8, 132.7, 135.9, 136.8. IR (CH_2Cl_2) ν 3058, 2992, 2927, 1449, 1264, 1045, 895, 763, 726 cm^{-1}. MS (%) m/z 282 (27.59), 267 (29.68), 254 (100.00), 239 (12.58), 191 (73.03), 178 (7.43), 165 (10.31), 126 (32.74), 120 (12.18). HRMS (EI) calcd. for $C_{22}H_{18}$: 282.1409, found: 282.1407.

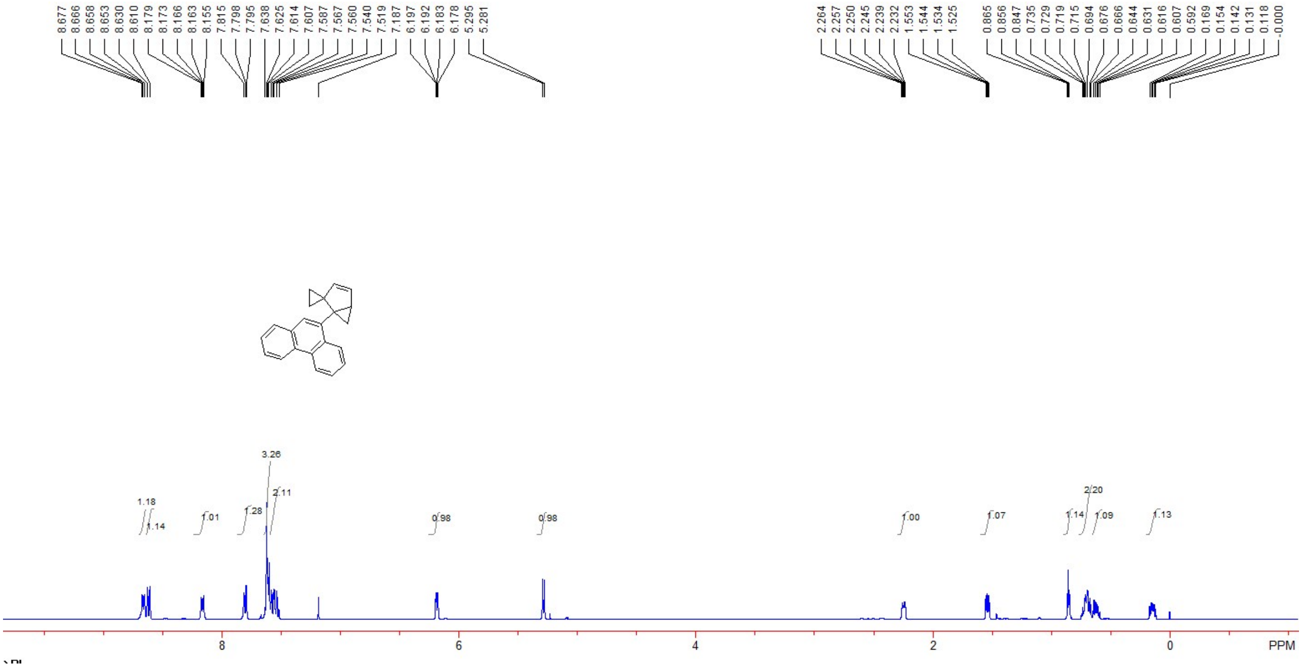

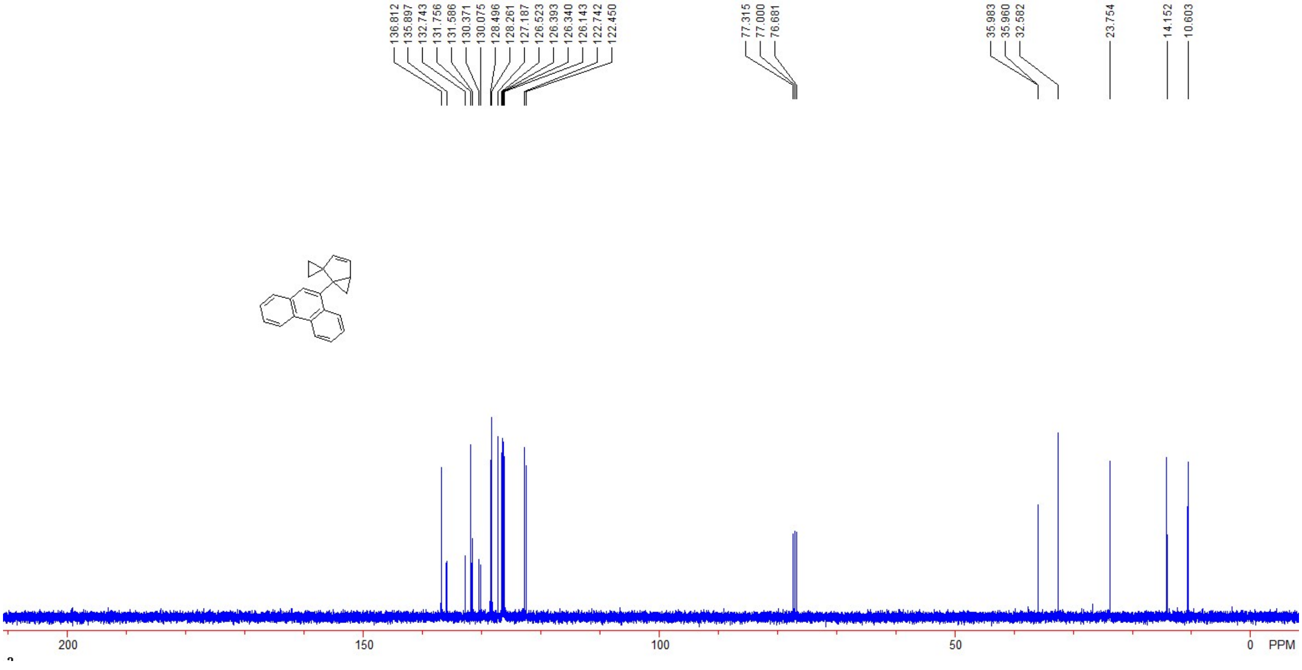

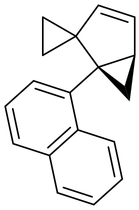

1-(Naphthalen-1-yl)spiro[bicyclo[3.1.0]hexane-2,1'-cyclopropan]-3-ene 5b:

37 mg, 80%. Colorless oil (Mixtures of two diastereomers, dr = 9.9/1). ^{1}H NMR (400 MHz, $CDCl_3$, TMS): δ 0.02-0.07 (m, 1H, CH_2), 0.55-0.73 (m, 3H, CH_2), 0.79-0.80 (m, 1H, CH_2), 1.43-1.48 (m, 1H, CH_2), 2.20-2.23 (m, 1H, CH), 5.26 (d, J = 5.6 Hz, 1H, =CH), 6.17 (dd, J_1 = 5.6 Hz, J_2 = 2.0 Hz, 1H, =CH), 7.21-7.36 (m, 2H, Ar), 7.43-7.51 (m, 2H, Ar), 7.70-7.72 (m, 1H, Ar), 7.80-7.82 (m, 1H, Ar), 8.07 (d, J = 8.0 Hz , 2H, Ar). ^{13}C NMR (100 MHz, $CDCl_3$, TMS): δ 10.5, 13.9, 23.6, 32.7, 35.7, 36.3, 125.1, 125.5, 125.7, 126.5, 127.5, 127.9, 128.4, 131.7, 133.6, 134.0, 136.7, 137.6. IR (CH_2Cl_2) ν 3057, 2989, 2915, 1587, 1508, 1420, 799, 776, 724 cm^{-1}. MS (%) m/z 232 (M^+, 53.49), 215 (58.84), 204 (100.00), 189 (13.57), 141 (60.07), 115 (12.95), 101 (35.26), 94 (3.28), 77 (11.86). HRMS (EI) calcd. for $C_{18}H_{16}$: 232.1252, found: 232.1249.

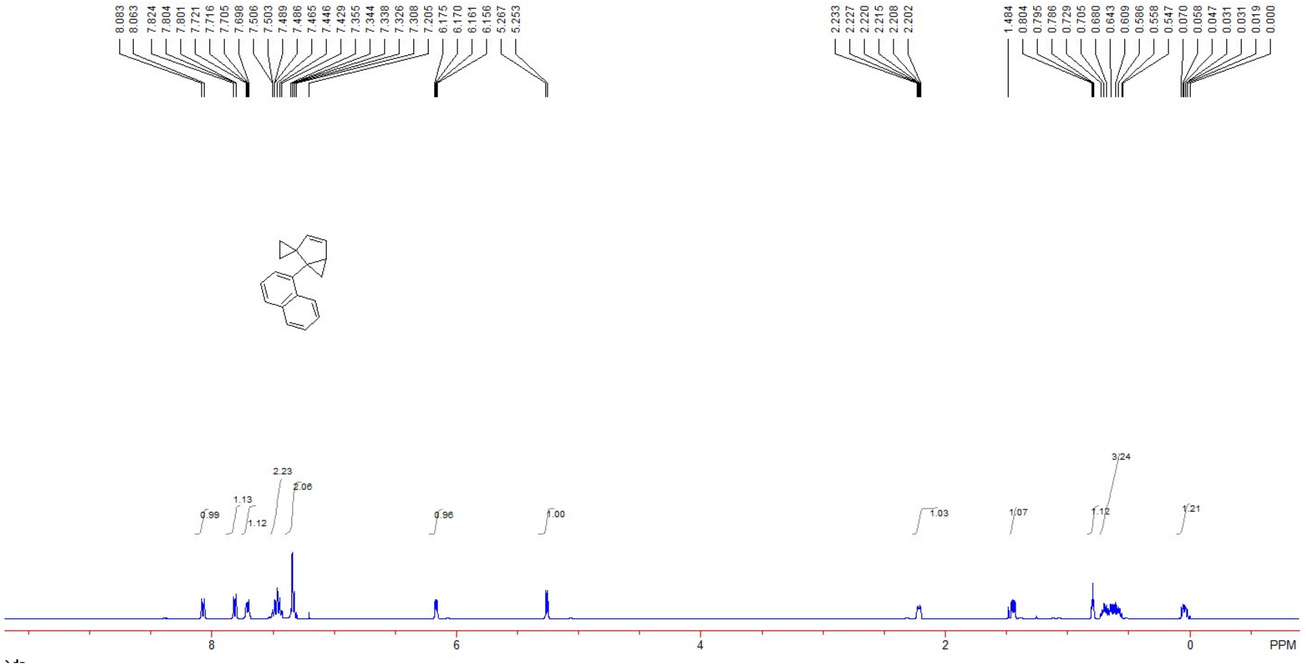

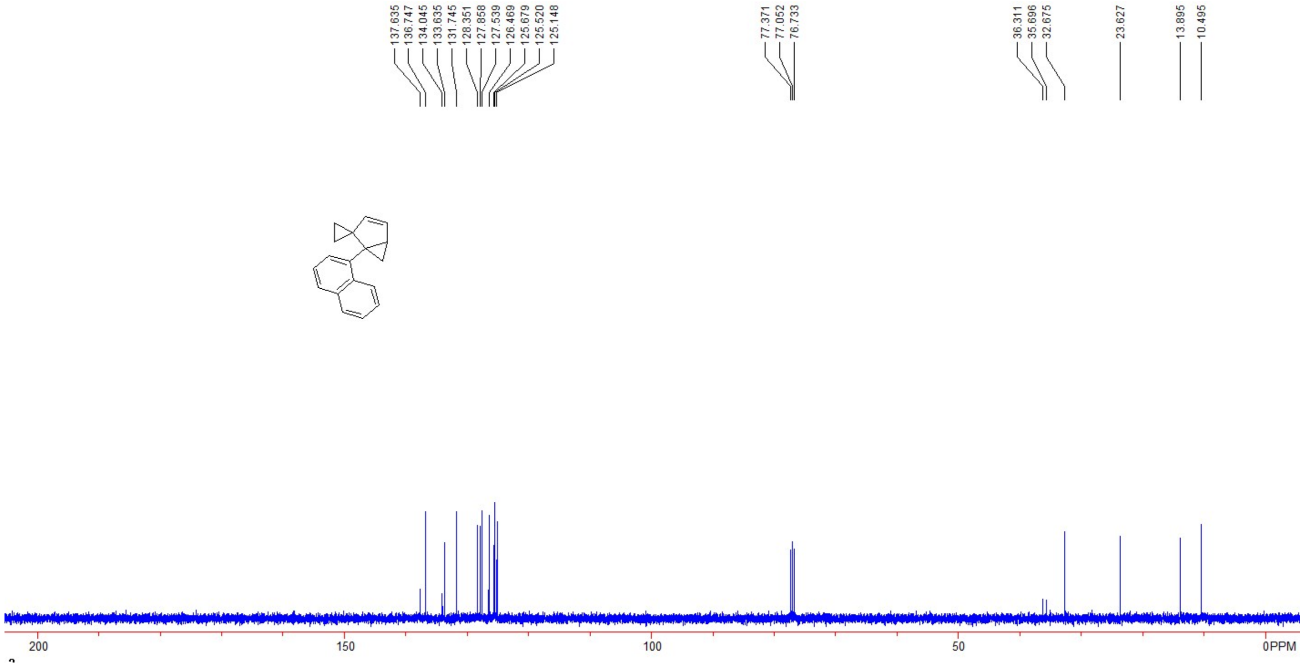

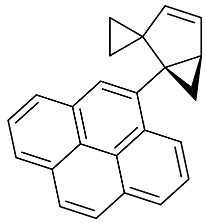

4-(spiro[bicyclo[3.1.0]hexane-2,1'-cyclopropan]-3-en-1-yl)pyrene 5c:

49 mg, yield = 80%. A white solid, mp: 81-82 °C (Mixtures of two diastereomers, dr = 7.1/1). ^{1}H NMR (400 MHz, $CDCl_3$, TMS): δ 0.15-0.20 (m, 1H, CH_2), 0.73-0.79 (m, 1H, CH_2), 0.83-0.88 (m, 2H, CH_2), 0.91-1.07 (m, 1H, CH_2), 1.72-1.75 (m, 1H, CH_2), 2.48-2.50 (m, 1H, CH), 5.46 (d, J = 5.6 Hz, 1H, =CH), 6.38 (dd, J = 5.6 Hz, 1H, =CH), 7.98 (d, J = 5.6 Hz, 1H, Ar), 8.06-8.15 (m, 4H, Ar), 8.17-8.29 (m, 3H, Ar), 8.45 (d, J = 9.2 Hz, 1H, Ar). ^{13}C NMR (100 MHz, $CDCl_3$, TMS): δ 10.5, 13.9, 23.8, 33.7, 36.2, 37.1, 124.4, 124.85, 124.88, 124.9, 125.2, 125.9, 127.1, 127.2, 127.4, 128.5, 130.5, 131.0, 131.3, 131.8, 131.9, 135.3, 136.9. IR (CH_2Cl_2) ν 3041, 1585, 1180, 971, 845, 757, 722, 683 cm^{-1}. MS (%) m/z 306 (M^+, 100.00), 289 (65.13), 273 (84.18), 239 (12.36), 215 (80.68), 145 (12.23), 138 (41.10), 117 (13.79), 91 (4.68). HRMS (EI) calcd. for $C_{24}H_{18}$: 306.1409, found: 306.1403.

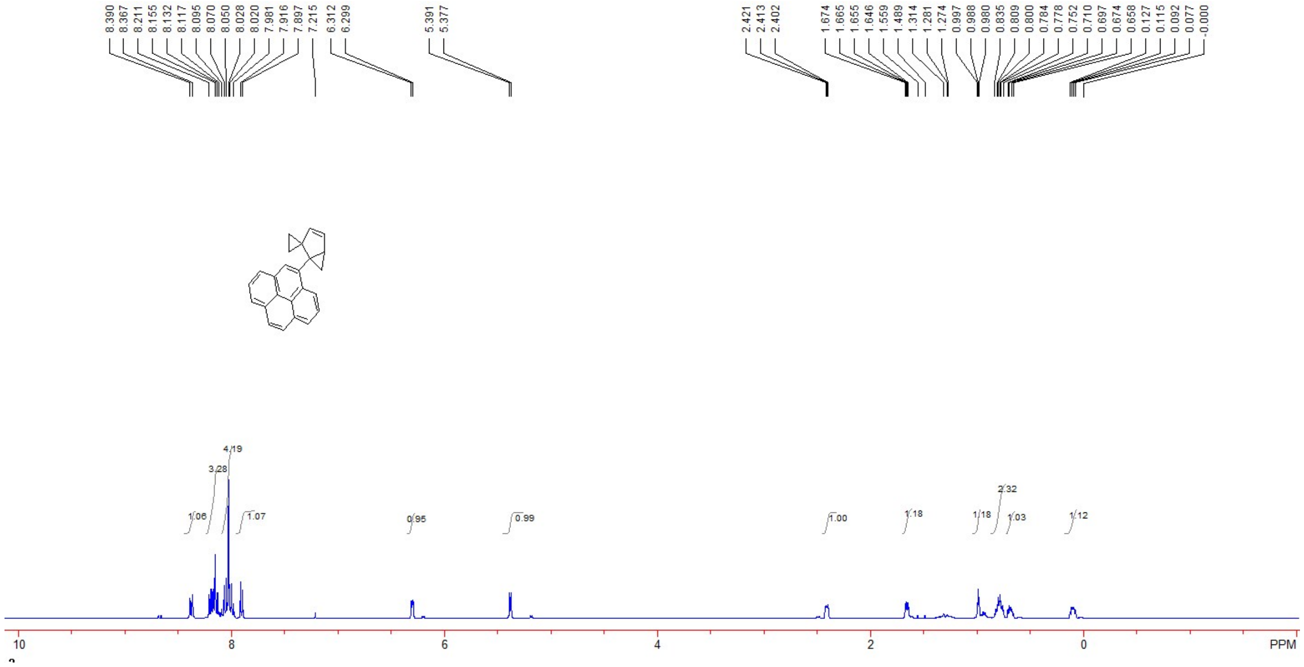

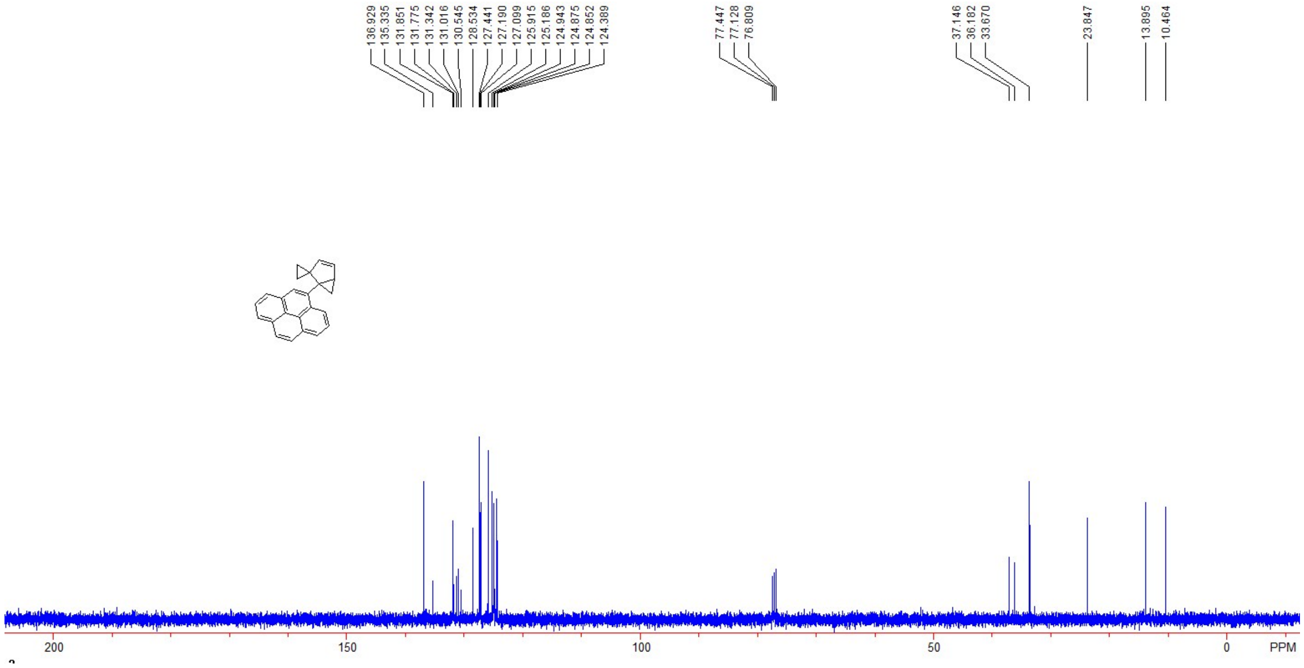

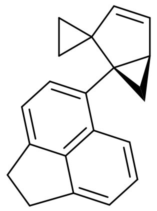

5-(Spiro[bicyclo[3.1.0]hexane-2,1'-cyclopropan]-3-en-1-yl)-1,2-dihydroacenaphthylene 5e:

36 mg, yield = 70%. A white solid, mp: 79-81 °C. ^{1}H NMR (400 MHz, $CDCl_3$, TMS): δ 0.11-0.12 (m, 1H, CH_2), 0.58-0.59 (m, 1H, CH_2), 0.60-0.75 (m, 3H, CH_2), 1.43-1.52 (m, 1H, CH_2), 2.17 (b, 1H, CH), 3.32-3.37 (m, 4H, CH_2), 5.24 (br, 1H, =CH), 6.14 (br, 1H, =CH), 7.15 (d, J = 6.4 Hz, 1H, Ar),

7.23-7.27 (m, 2H, Ar), 7.44 (dd, J_1 = 5.6 Hz, J_2 = 2.0 Hz, 1H, =CH), 7.70-7.71 (m, 1H, Ar). ^{13}C NMR (100 MHz, $CDCl_3$, TMS): δ 10.3, 13.6, 23.4, 29.9, 30.6, 32.1, 34.8, 35.9, 118.6, 119.0, 121.6, 127.4, 129.1, 131.7, 132.3, 133.2, 136.5, 139.3, 145.2, 146.1. IR (CH_2Cl_2) ν 3059, 1588, 1444, 879, 837, 772, 755, 725 cm^{-1}. MS (%) m/z 258 (M^+, 76.36), 243 (39.55), 230 (100.00), 215 (27.90), 202 (20.48), 167 (54.36), 152 (29.74), 113 (16.01), 101 (13.97). HRMS (EI) calcd. for $C_{20}H_{18}$: 258.1409, found: 258.1402.

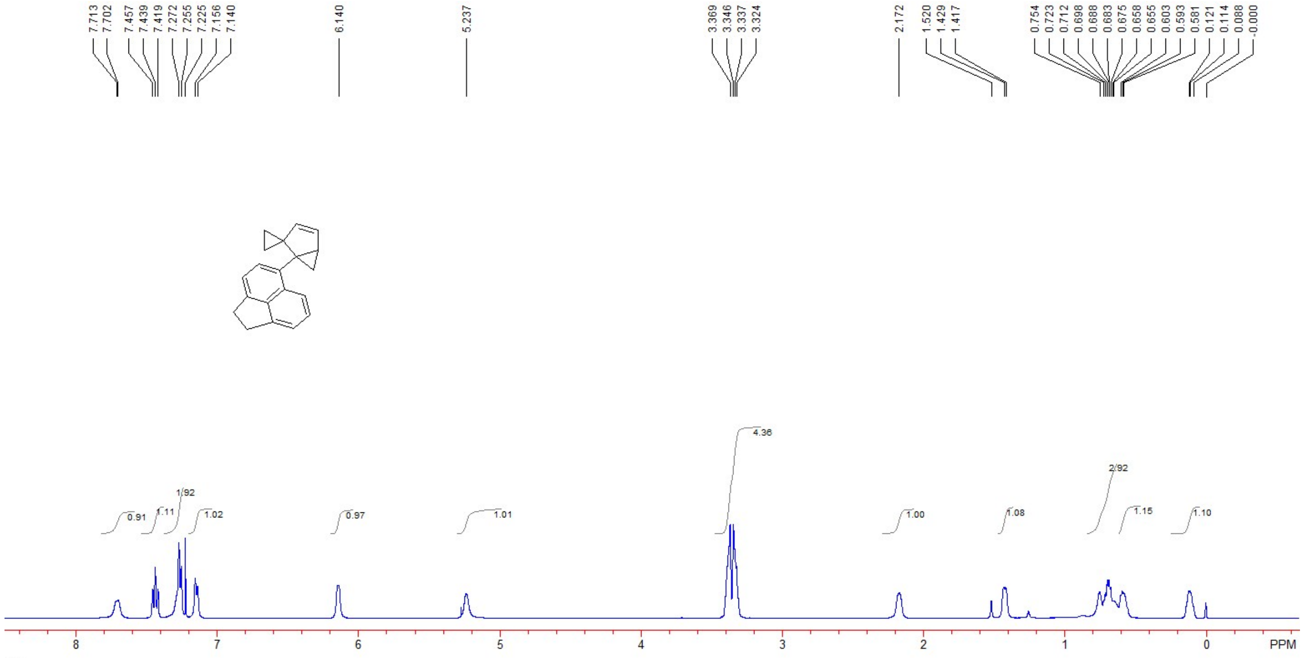

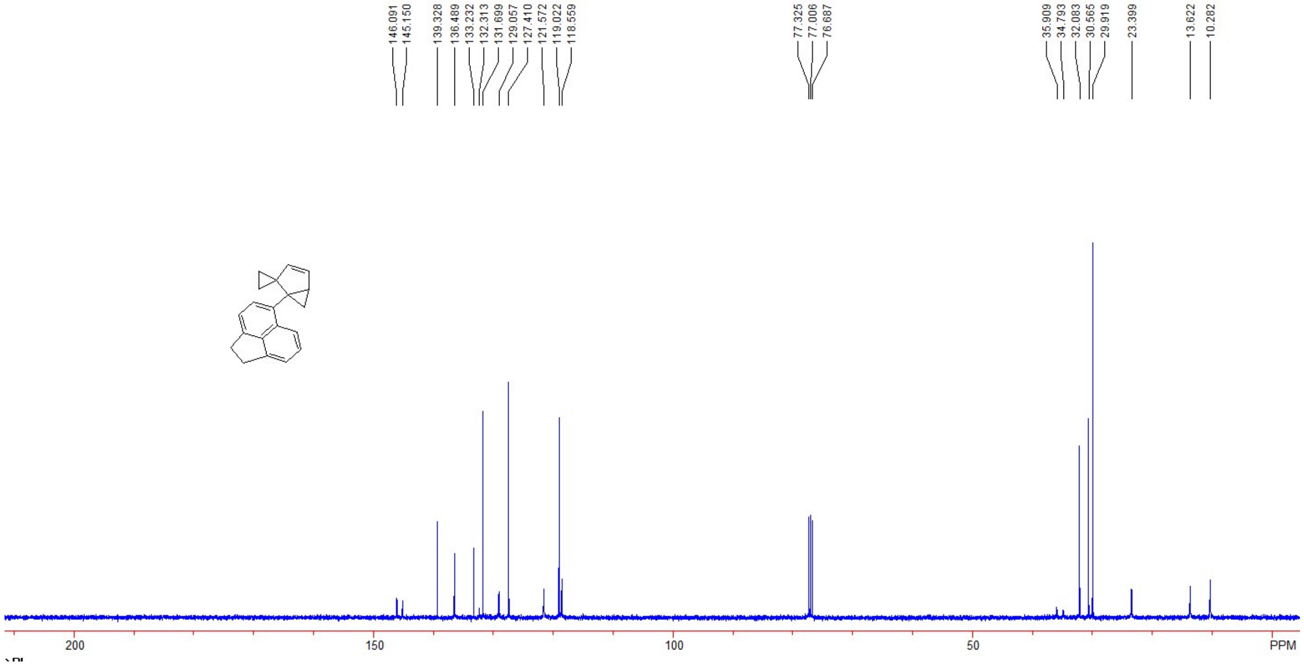

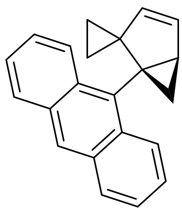

1-Phenylspiro[bicyclo[3.1.0]hexane-2,1'-cyclopropan]-3-ene 5f:

39 mg, yield = 68%. A white solid, mp: 95-97 °C. ^{1}H NMR (400 MHz, $CDCl_3$, TMS): δ 0.02-0.07 (m, 1H, CH_2), 0.60-0.64 (m, 1H, CH_2), 0.73-0.78 (m, 1H, CH_2), 1.15-1.20 (m, 1H, CH_2), 1.36-1.37 (m, 1H, CH_2), 1.55-1.59 (m, 1H, CH_2), 2.37-2.40 (m, 1H, CH), 5.34 (d, J = 5.6 Hz, 1H, =CH), 6.29 (dd, J_1 = 5.6 Hz, J_2 = 2.4 Hz, 1H, =CH), 7.43-7.56 (m, 4H, Ar), 7.98-8.02 (m, 2H, Ar), 8.37-8.41 (m, 2H, Ar), 8.70 (d, J = 8.4 Hz, 1H, Ar). ^{13}C NMR (100 MHz, $CDCl_3$, TMS): δ 8.8, 13.0, 27.0, 30.9, 34.6, 37.3, 124.7, 124.9, 125.1, 125.3, 125.9, 126.8, 128.8, 131.42, 131.45, 131.47, 132.0, 132.5, 133.3, 136.7. IR (CH_2Cl_2) ν 3050, 2999, 2916, 1623, 1442, 1322, 1016, 906, 791 cm^{-1}. MS (%) m/z 282 (M$^+$, 18.23), 267 (9.10), 254 (26.90), 239 (8.43), 191 (100.00), 178 (6.22), 126 (11.37), 114 (1.00), 91 (1.97), 77 (1.50). HRMS (EI) calcd. for $C_{22}H_{18}$: 282.1409, found: 282.1412.

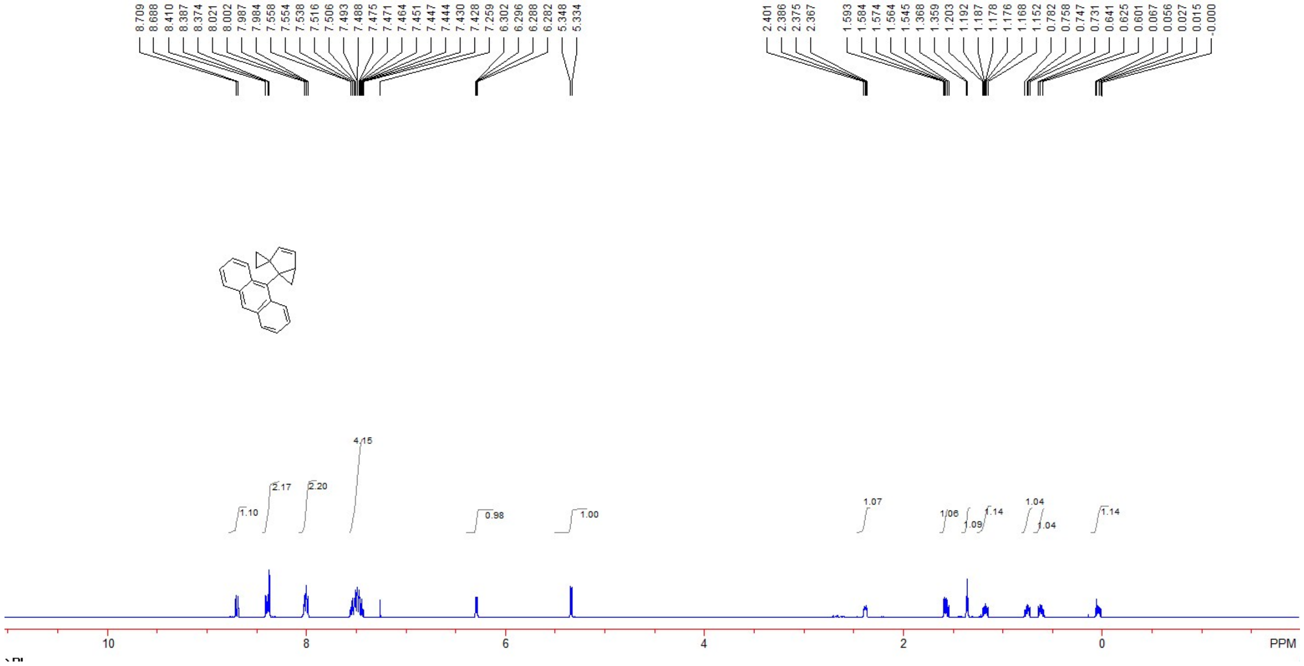

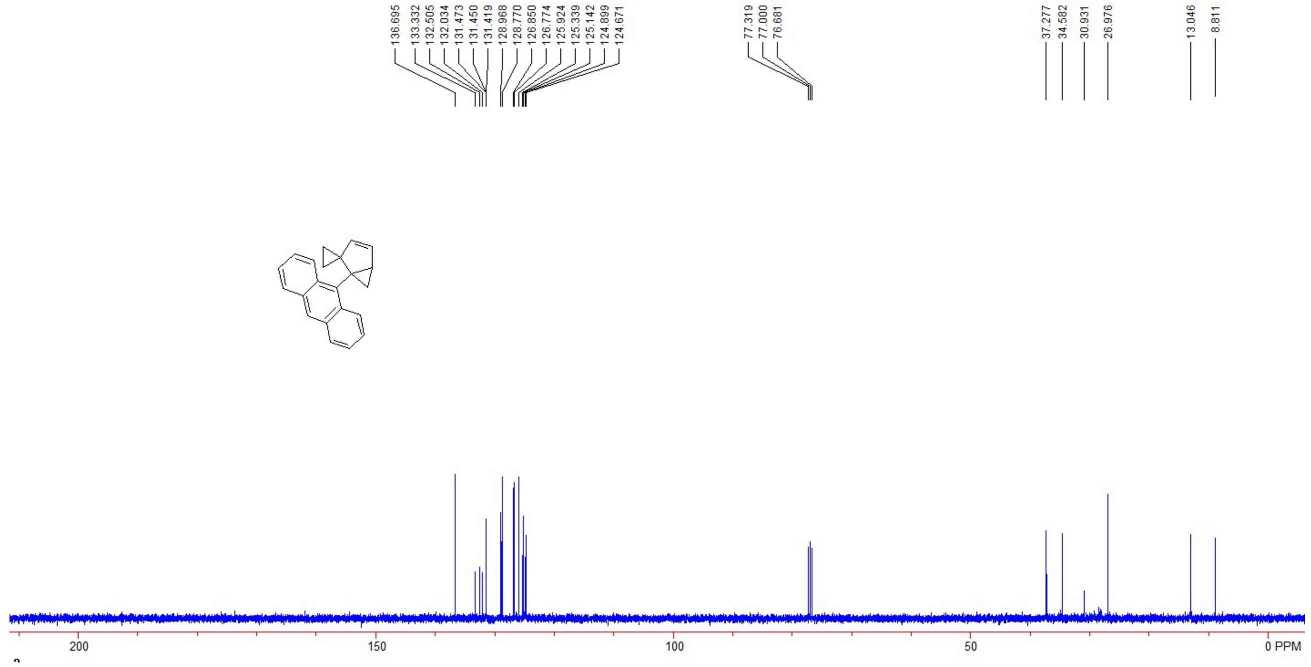

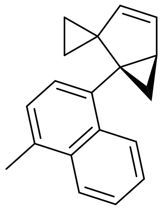

1-(4-methylnaphthalen-1-yl)spiro[bicyclo[3.1.0]hexane-2,1'-cyclopropan]-3-ene 5g:

42 mg, yield = 84%. Colorless oil (Mixtures of two diastereomers, dr = 9.9/1). ^{1}H NMR (400 MHz, $CDCl_3$, TMS): δ 0.03-0.08 (m, 1H, CH_2), 0.55-0.71 (m, 3H, CH_2), 0.72-0.78 (m, 1H, CH_2), 1.41-1.44 (m, 1H, CH_2), 2.18-2.21 (m, 1H, CH), 2.64 (s, 3H, CH_3), 5.26 (d, J = 5.6 Hz, 1H, =CH), 6.16 (dd, J_1 = 5.6 Hz, J_2 = 2.0 Hz, 1H, =CH), 7.17 (d, J = 7.6 Hz, 1H, Ar), 7.24 (d, J = 6.8 Hz, 1H, Ar), 7.49 (dd, J_1 = 6.8 Hz, J_2 = 3.6 Hz, 1H, Ar), 7.96-7.99 (m, 1H, Ar), 8.08-8.11 (m, 1H, Ar). ^{13}C NMR (100 MHz, $CDCl_3$, TMS): δ 10.4, 13.9, 19.5, 23.6, 32.7, 35.6, 36.2, 124.4, 125.3, 125.9, 127.0, 127.6, 131.7, 132.6, 133.6, 133.9, 135.7, 136.7. IR (CH_2Cl_2) ν 2989, 1275, 1261, 973, 878, 836, 752, 725 cm^{-1}. MS (%) m/z 246 (M^+, 21.86), 231 (74.66), 218 (100.00), 203 (45.02), 189 (13.77), 155 (49.96), 115 (17.63), 108 (15.66), 95 (1.09). HRMS (EI) calcd. for $C_{19}H_{18}$: 246.1409, found: 246.1404.

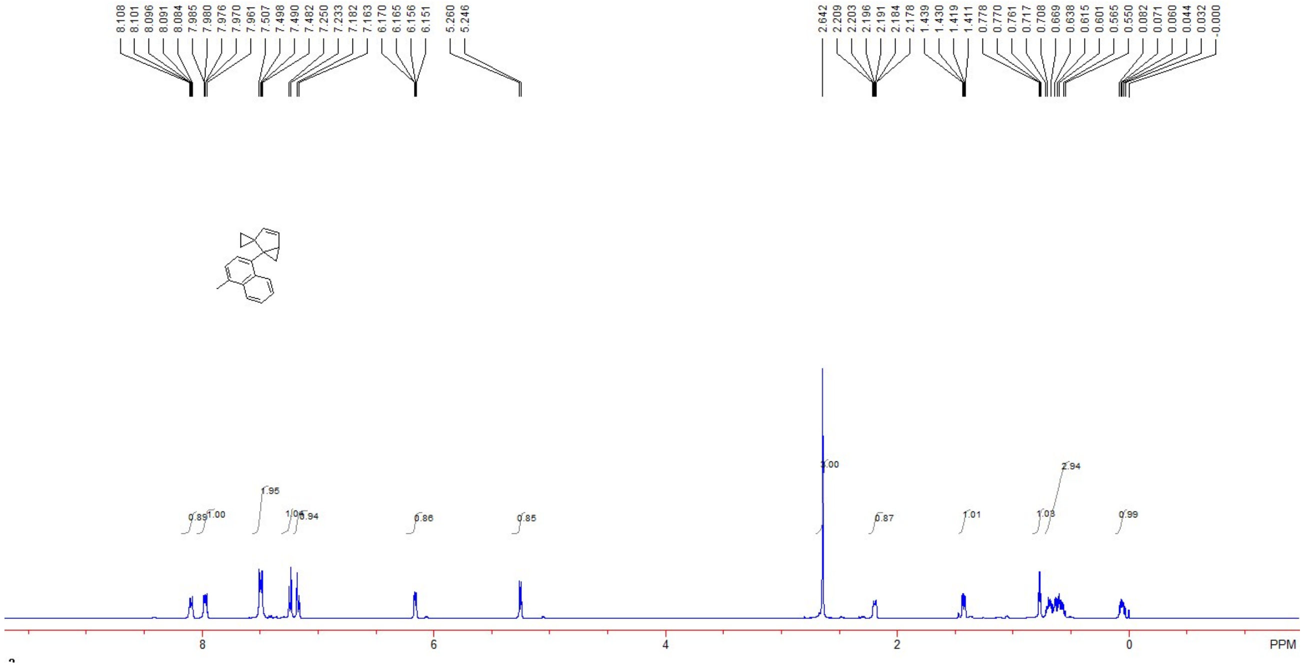

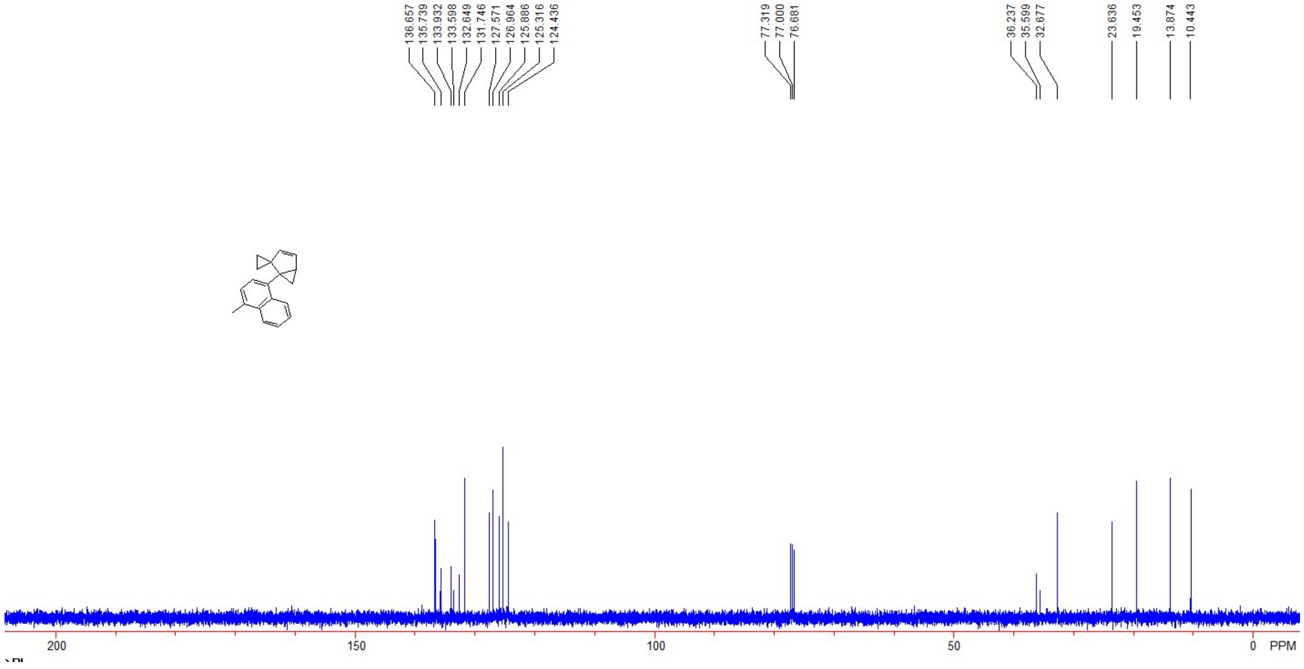

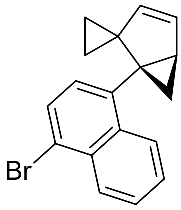

1-(4-bromonaphthalen-1-yl)spiro[bicyclo[3.1.0]hexane-2,1'-cyclopropan]-3-ene 5h:

51 mg, yield = 82%. Colorless oil (Mixtures of two diastereomers, dr = 11.6/1). ^{1}H NMR (400 MHz, $CDCl_3$, TMS): δ 0.03-0.09 (m, 1H, CH_2), 0.59-0.68 (m, 2H, CH_2), 0.71-0.77 (m, 1H, CH_2), 0.83-0.84 (m, 1H, CH_2), 1.43-1.46 (m, 1H, CH_2), 2.23-2.25 (m, 1H, CH), 5.29 (d, J = 5.6 Hz, 1H, =CH), 6.19

(dd, J_1 = 5.6 Hz, J_2 = 2.0 Hz, 1H, =CH), 7.22 (d, J = 7.6 Hz, 1H, Ar), 7.55-7.62 (m, 2H, Ar), 7.67 (d, J = 7.6 Hz, 1H, Ar), 8.11 (d, J = 8.8 Hz, 1H, Ar), 8.26-8.28 (m, 1H, Ar). ^{13}C NMR (100 MHz, $CDCl_3$, TMS): δ 10.5, 13.9, 23.5, 32.9, 35.4, 36.3, 122.2, 126.5, 126.86, 126.93, 127.5, 128.2, 129.2, 131.6, 131.8, 135.1, 136.7, 137.8. IR (CH_2Cl_2) ν 1587, 1505, 1387, 912, 836, 755, 724, 659 cm^{-1}. MS (%) m/z 310 (M$^+$, 9.18), 284 (54.07), 231 (81.95), 215 (100.00), 202 (88.07), 189 (13.52), 163 (9.08), 126 (11.22), 108 (27.80). HRMS (EI) calcd. for $C_{18}H_{15}Br$: 310.0357, found: 310.0355.

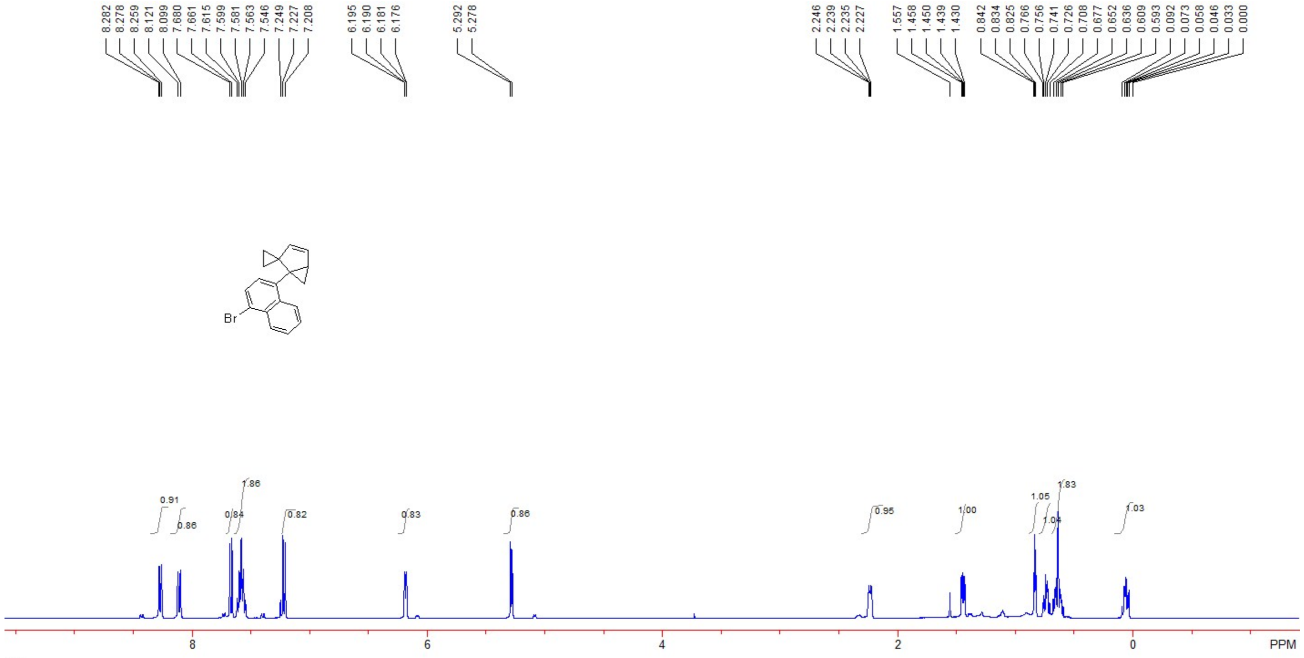

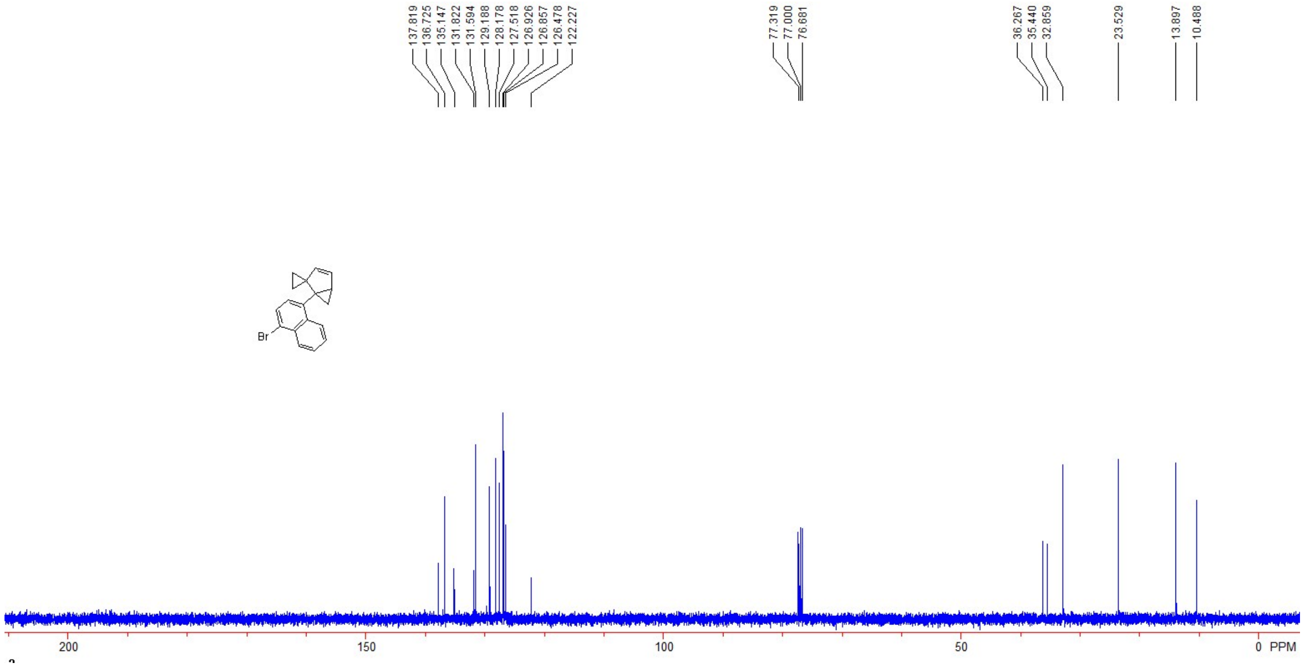

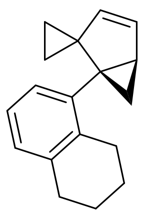

1-(5,6,7,8-tetrahydronaphthalen-1-yl)spiro[bicyclo[3.1.0]hexane-2,1'-cyclopropan]-3-ene 5j:
39 mg, yield = 83%. Colorless oil (Mixtures of two diastereomers, dr = 22.7/1). ^{1}H NMR (400 MHz, $CDCl_3$, TMS): δ 0.26-0.30 (m, 1H, CH_2), 0.57-0.70 (m, 4H, CH_2), 1.29-1.31 (m, 1H, CH_2), 1.70-1.78 (m, 2H, CH_2), 1.80-1.86 (m, 2H, CH_2), 2.07-2.80 (m, 1H, CH), 2.69-2.70 (m, 2H, CH_2), 2.71-2.77 (m, 2H, CH_2), 5.13 (d, J = 5.6 Hz, 1H, =CH), 6.02 (dd, J_1 = 5.6 Hz, J_2 = 2.0 Hz, 1H, =CH), 6.94-7.01 (m, 3H, Ar). ^{13}C NMR (100 MHz, $CDCl_3$, TMS): δ 9.8, 12.8, 23.1, 23.2, 24.1, 26.4, 29.9, 31.7, 34.9, 36.0. 124.8, 128.0, 128.3, 131.7, 136.1, 137.0, 138.4, 139.2. IR (CH_2Cl_2) ν 3058, 2928, 2857, 1580, 1452, 1047, 971, 831, 722 cm^{-1}. MS (%) m/z 236 (63.93), 208 (100.00), 193 (60.12), 179 (74.89), 165 (64.71), 152 (19.80), 128 (19.21), 115 (28.30), 89 (16.81). HRMS (EI) calcd. for $C_{18}H_{20}$: 236.1565, found: 236.1569.

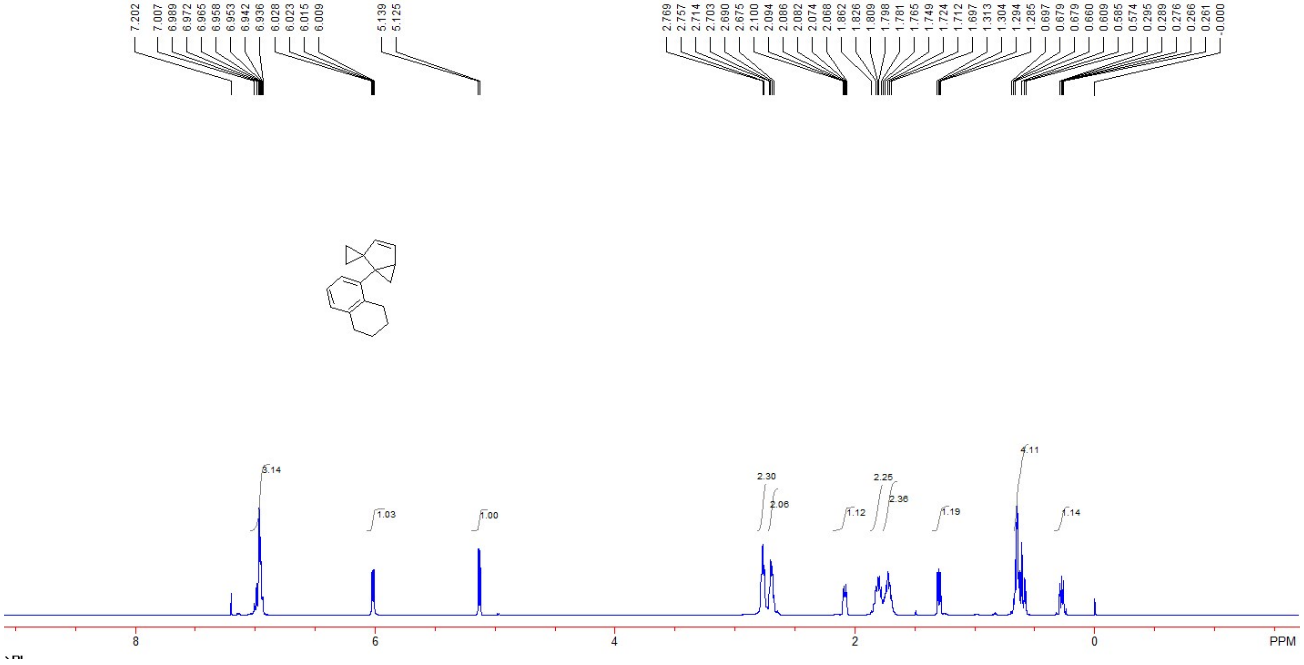

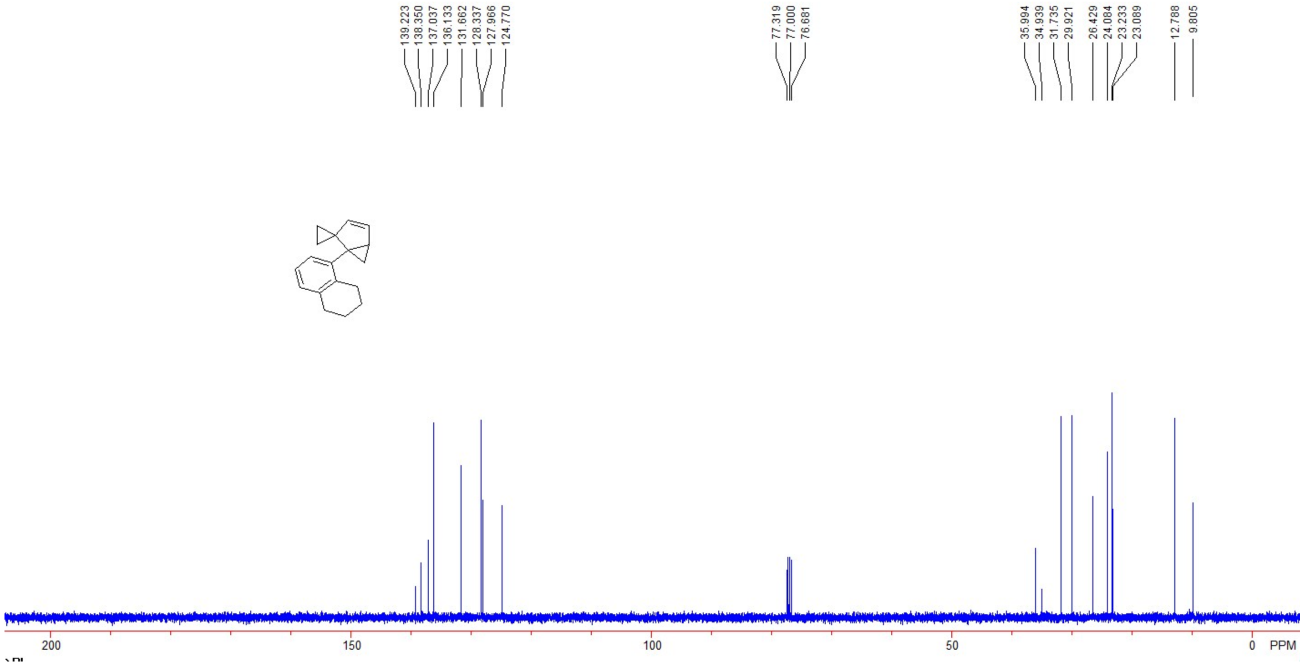

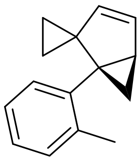

1-(o-tolyl)spiro[bicyclo[3.1.0]hexane-2,1'-cyclopropan]-3-ene 5k:

32 mg, yield = 80%. Colorless oil (Mixtures of two diastereomers, dr = 15.9/1). ^{1}H NMR (400 MHz, $CDCl_3$, TMS): δ 0.26-0.29 (m, 1H, CH_2), 0.59-0.60 (m, 1H, CH_2), 0.62-0.69 (m, 3H, CH_2), 1.32-1.34 (m, 1H, CH_2), 2.07-2.10 (m, 1H, CH), 2.29 (s, 3H, CH_3), 5.14 (d, J = 5.6 Hz, 1H, =CH), 6.03 (dd, J_1 = 5.6 Hz, J_2 = 2.0 Hz, 1H, =CH), 7.05-7.09 (m, 1H, Ar), 7.10-7.13 (m, 3H, Ar). ^{13}C NMR (100 MHz, $CDCl_3$, TMS): δ 9.7, 12.8, 19.5, 23.8, 31.8, 35.1, 36.1, 125.3, 126.8, 129.8, 130.8, 131.6, 136.2, 138.8, 140.2. IR (CH_2Cl_2) ν 3060, 2989, 1587, 1490, 971, 877, 833, 750, 724 cm^{-1}. MS (%) m/z 196 (12.52), 181 (100.00), 167 (98.03), 153 (48.96), 128 (19.41), 115 (34.53), 105 (31.70), 89 (31.73), 77 (23.03). HRMS (EI) calcd. for $C_{15}H_{16}$: 196.1252, found: 196.1255.

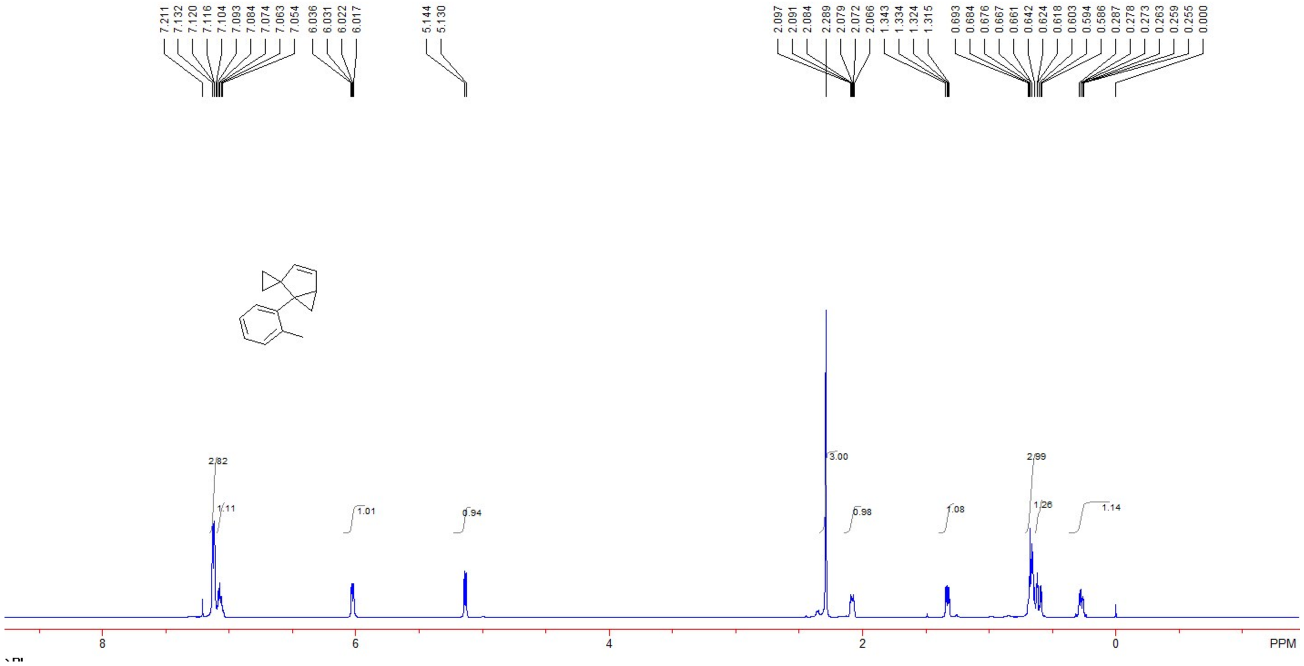

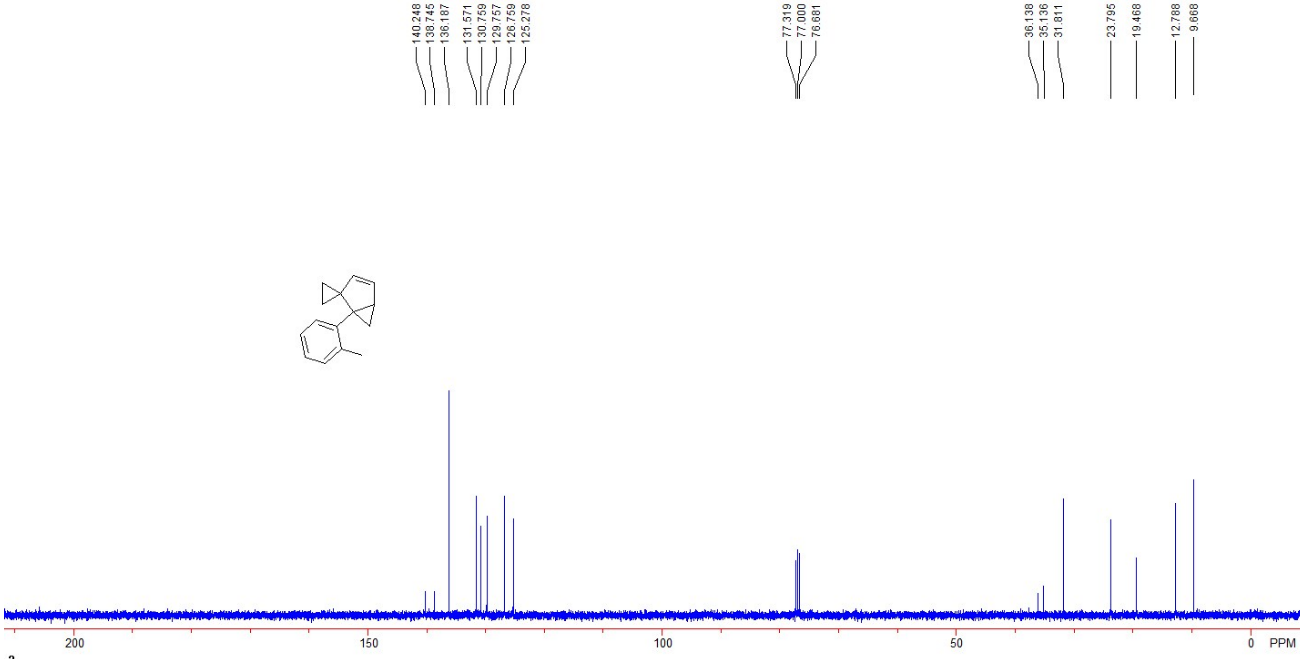

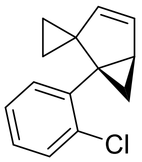

1-(2-chlorophenyl)spiro[bicyclo[3.1.0]hexane-2,1'-cyclopropan]-3-ene 5l:

34 mg, yield = 79%. Colorless oil. ^{1}H NMR (400 MHz, $CDCl_3$, TMS): δ 0.28-0.32 (m, 1H, CH_2), 0.59-0.73 (m, 4H, CH_2), 1.31 (b, 1H, CH_2), 2.21-2.25 (m, 1H, CH_2), 5.13 (br, 1H, =CH), 6.02 (d, J = 4.0 Hz, 1H, =CH), 7.14-7.16 (m, 2H, Ar), 7.21-7.25 (m, 1H, Ar), 7.32-7.34 (m, 1H, Ar). ^{13}C NMR (100 MHz, $CDCl_3$, TMS): δ 9.5, 13.0, 23.7, 32.4, 35.0, 36.1, 126.3, 128.0, 129.4, 131.4, 132.5, 136.0,

138.3. IR (CH_2Cl_2) ν 3060, 2992, 1476, 1432, 1057, 826, 750, 725, 707 cm^{-1}. MS (%) m/z 216 (M^+, 5.11), 201 (9.96), 188 (100.00), 181 (36.72), 165 (43.10), 152 (30.59), 127 (6.85), 115 (8.43), 89 (16.73). HRMS (EI) calcd. for $C_{14}H_{13}Cl$: 216.0706, found: 216.0703.

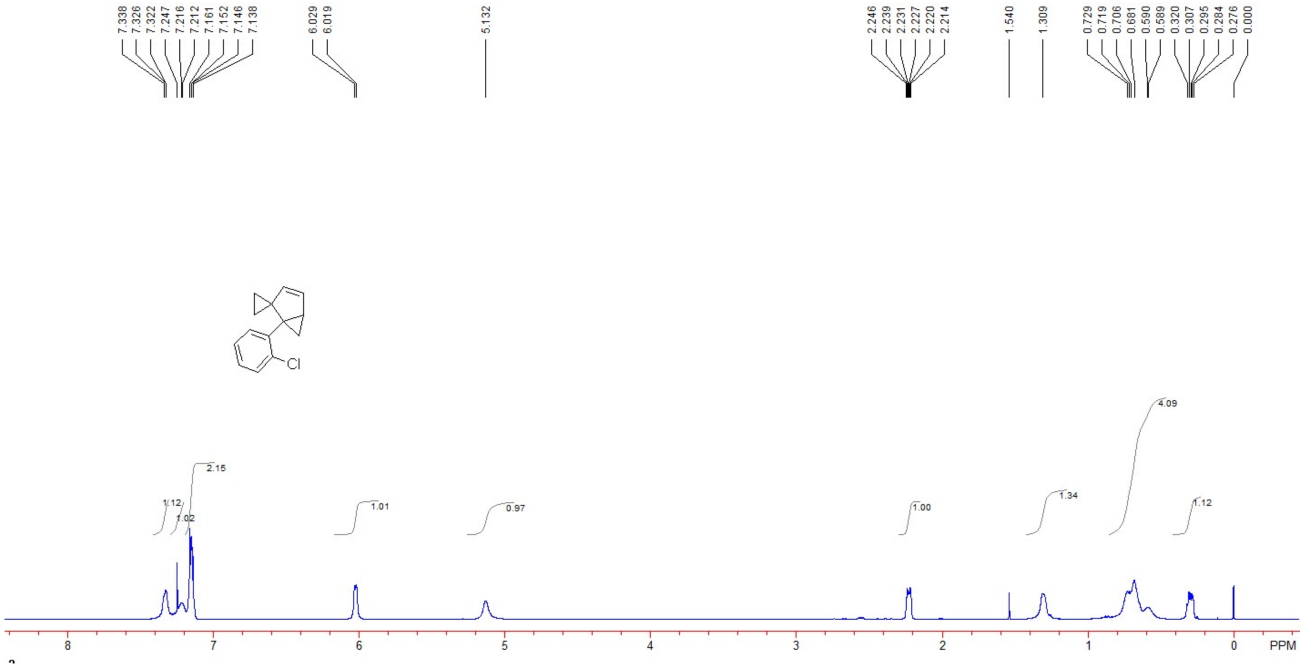

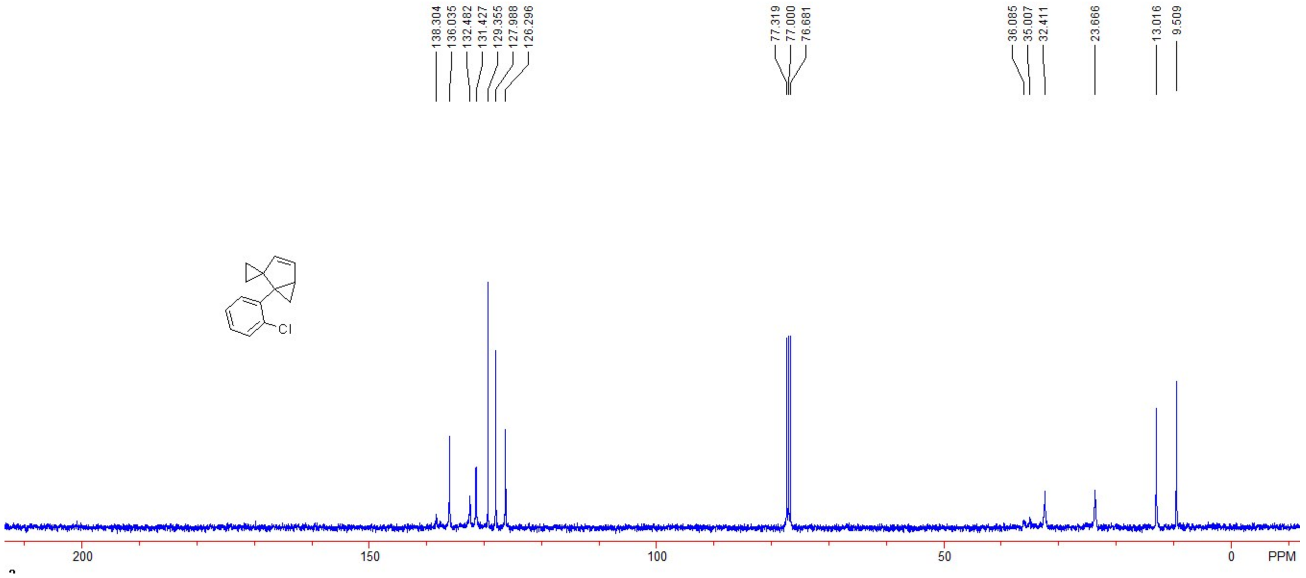

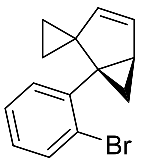

1-(2-bromophenyl)spiro[bicyclo[3.1.0]hexane-2,1'-cyclopropan]-3-ene 5m:

34 mg, yield = 65%. Colorless oil (Mixtures of two diastereomers, dr = 5.2/1). ^{1}H NMR (400 MHz, $CDCl_3$, TMS): δ 0.32-0.33 (m, 1H, CH_2), 0.57-0.68 (m, 1H, CH_2), 0.69-0.77 (m, 3H, CH_2), 1.30-1.31

(m, 1H, CH_2), 2.26 (br, 1H, CH), 5.14 (d, *J* = 5.2 Hz, 1H, =CH), 6.04 (d, *J* = 3.6 Hz, 1H, =CH), 7.07 (br, 1H, Ar), 7.20-7.24 (m, 2H, Ar), 7.54 (d, *J* = 7.6 Hz, 1H, Ar). ^{13}C NMR (100 MHz, $CDCl_3$, TMS): δ 9.5, 13.4, 24.1, 32.7, 35.0, 37.8, 125.1, 126.9, 128.1, 128.2, 131.6, 132.7, 132.9, 136.0, 139.8. IR (CH_2Cl_2) ν 3059, 2992, 1473, 1425, 1024, 750, 724, 687 cm^{-1}. MS (%) m/z 260 (M^+, 1.21), 232 (100.00), 181 (45.84), 165 (75.76), 152 (64.65), 89 (43.88), 76 (52.22), 63 (19.75), 51 (18.16). HRMS (EI) calcd. for $C_{14}H_{13}Br$: 260.0201, found: 260.0200.

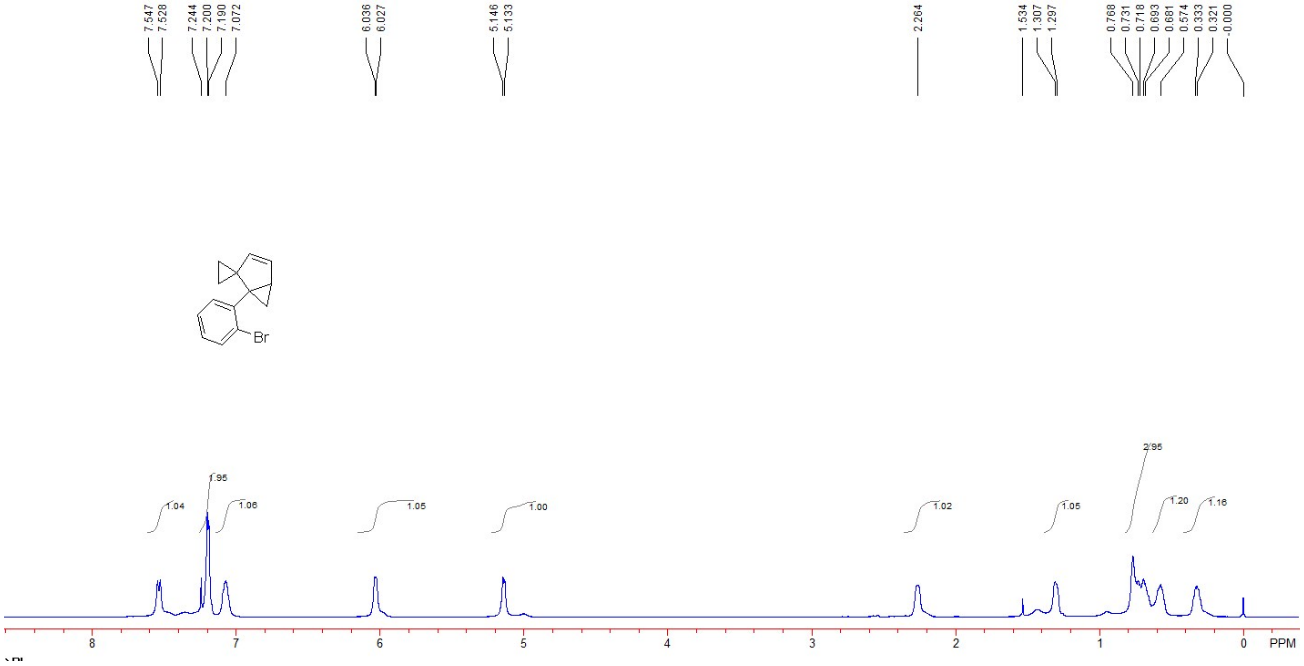

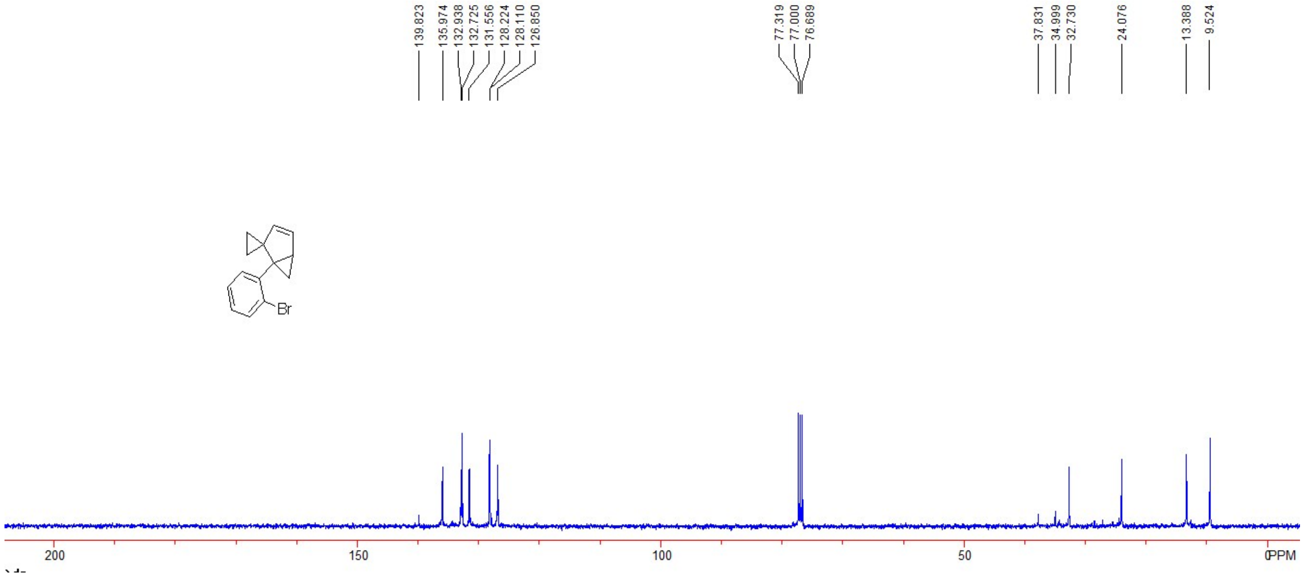

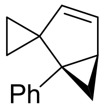

1-Phenylspiro[bicyclo[3.1.0]hexane-2,1'-cyclopropan]-3-ene 5m':

30 mg, yield = 83%. Colorless oil. ^{1}H NMR (400 MHz, $CDCl_3$, TMS): δ 0.37-0.42 (m, 1H, CH_2), 0.61-0.65 (m, 2H, CH_2), 0.69-0.74 (m, 2H, CH_2), 1.29-1.32 (m, 1H, CH_2), 2.14-2.18 (m, 1H, CH), 5.08 (d, J = 5.6 Hz, 1H, =CH), 6.01 (dd, J_1 = 5.6 Hz, J_2 = 2.0 Hz, 1H, =CH), 7.17-7.19 (m, 1H, Ar), 7.20-7.25 (m, 4H, Ar). ^{13}C NMR (100 MHz, $CDCl_3$, TMS): δ 9.8, 12.8, 23.4, 31.8, 36.4, 36.6, 126.5, 127.9, 130.6, 131.8, 136.3, 140.9. IR (CH_2Cl_2) ν 3058, 3026, 2990, 1589, 1495, 823, 724, 699 cm^{-1}. MS (%) m/z 182 (M^+, 24.57), 167 (55.15), 154 (100.00), 128 (15.21), 115 (17.72), 91 (21.20), 77 (14.31), 63 (6.38), 51 (11.55). HRMS (EI) calcd. for $C_{14}H_{14}$: 182.1096, found: 182.1099.

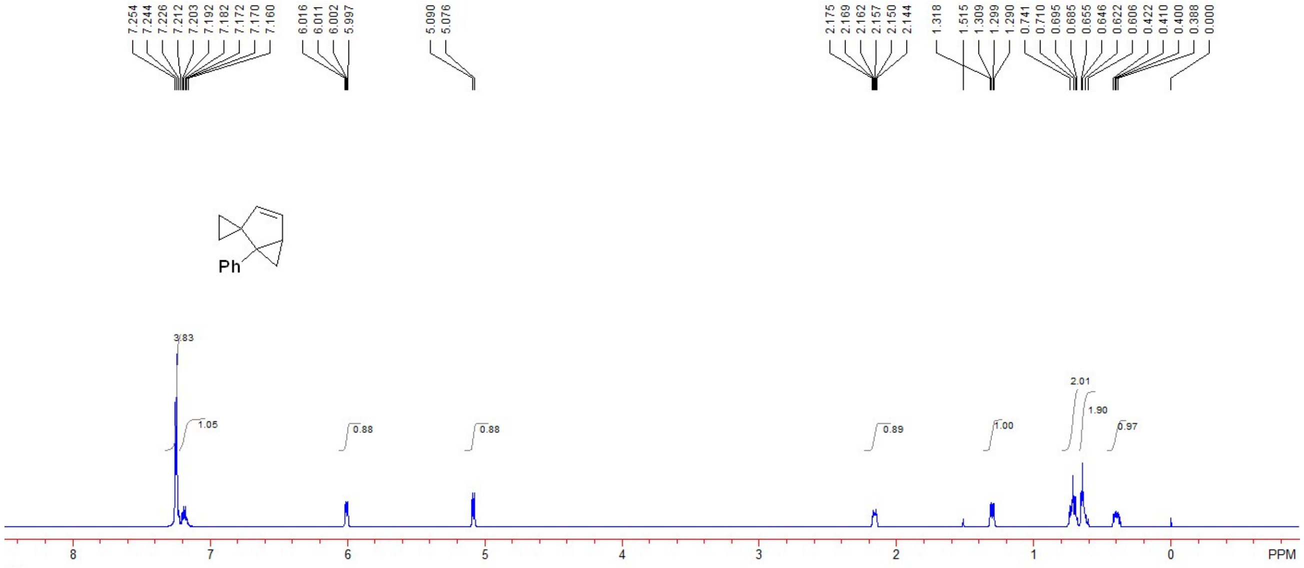

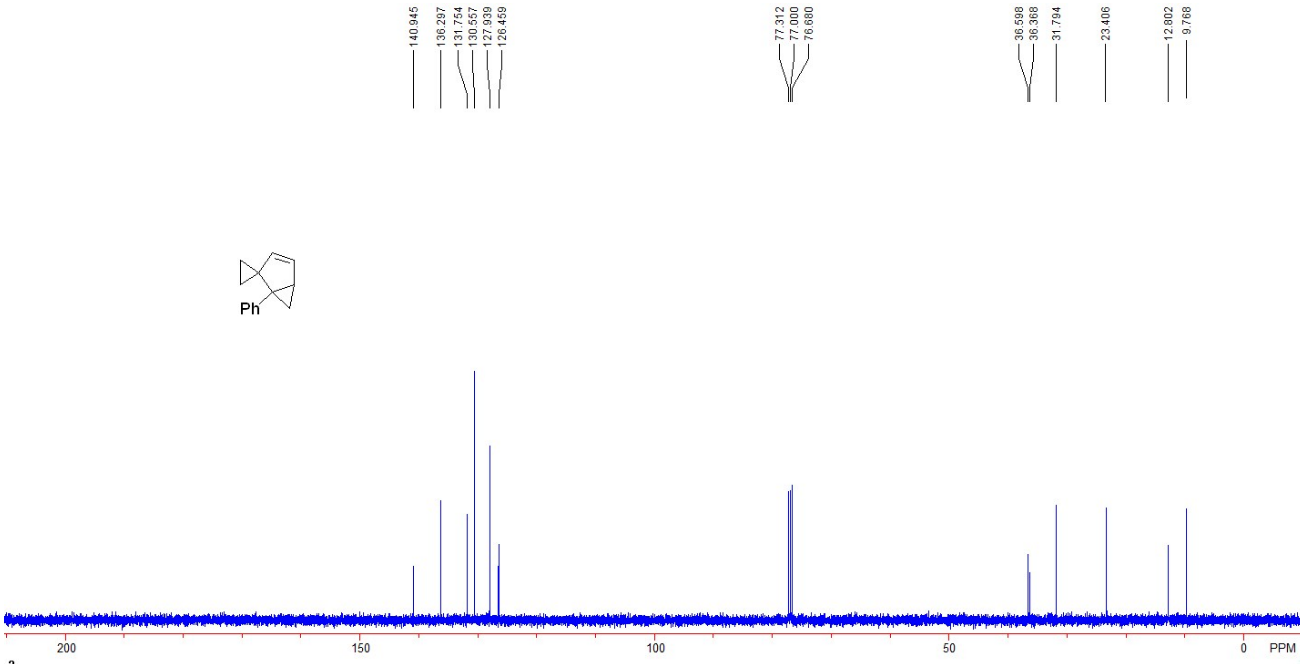

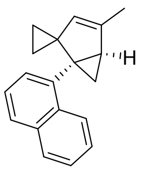

4-Methyl-1-(naphthalen-1-yl)spiro[bicyclo[3.1.0]hexane-2,1'-cyclopropan]-3-ene 5p:

38 mg, yield = 78%. Colorless oil (Mixtures of two diastereomers, dr = 8.8/1). ^{1}H NMR (400 MHz, $CDCl_3$, TMS): δ 0.01-0.05 (m, 1H, CH_2), 0.53-0.68 (m, 1H, CH_2), 0.87-0.89 (m, 1H, CH_2), 1.45-1.48 (m, 1H, CH_2), 2.02 (s, 3H, CH_3), 2.09-2.11 (m, 1H, CH), 4.90 (s, 1H, =CH), 7.35-7.39 (m, 2H, Ar), 7.48-7.57 (m, 2H, Ar), 7.74-7.77 (m, 1H, Ar), 7.85-7.87 (m, 1H, Ar), 8.15 (d, J = 8.4 Hz, 1H, Ar). ^{13}C NMR (100 MHz, $CDCl_3$, TMS): δ 9.9, 13.5, 16.6, 23.0, 35.68, 35.73, 36.4, 125.1, 125.4, 125.6, 126.4, 127.4, 127.8, 128.3, 129.5, 133.6, 134.0, 137.7, 141.9. IR (CH_2Cl_2) ν 3040, 2988, 2846, 1594, 1508, 1403, 874, 801, 775 cm^{-1}. MS (%) m/z 246 (M^+, 30.03), 231 (60.33), 218 (100.00), 203 (52.07), 165 (9.57), 141 (74.85), 115 (16.02), 108 (27.03), 100 (4.18). HRMS (EI) calcd. for $C_{19}H_{18}$: 246.1409, found: 246.1404.

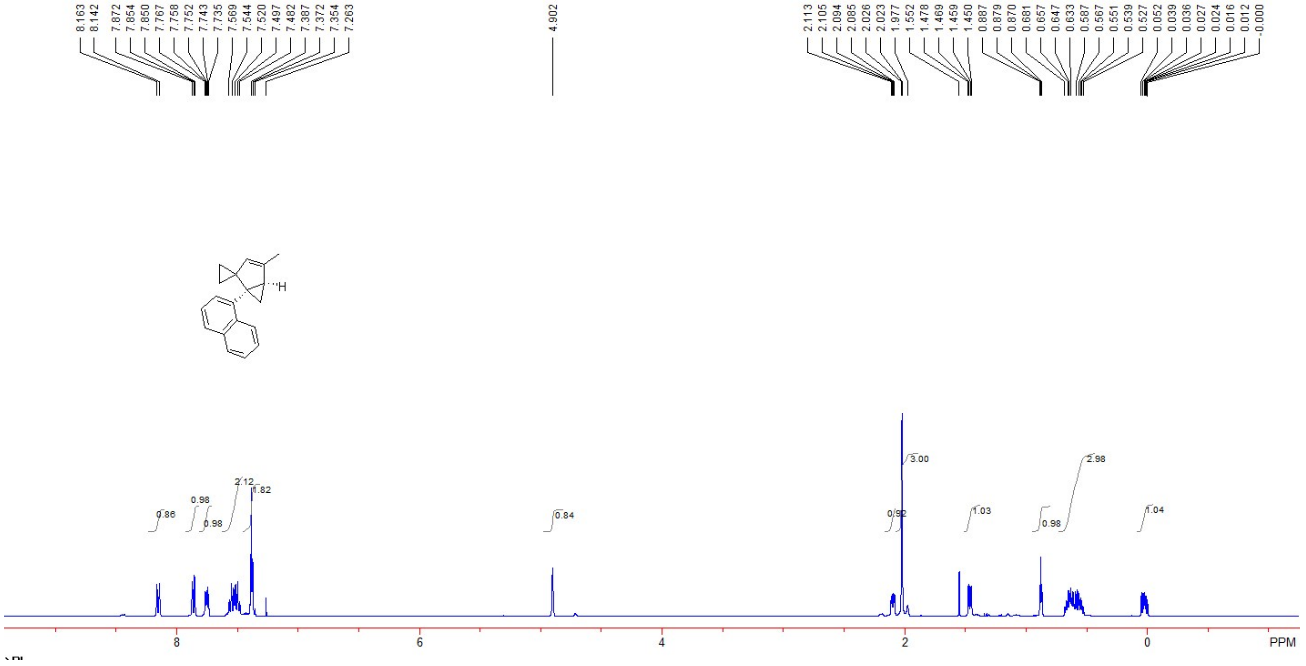

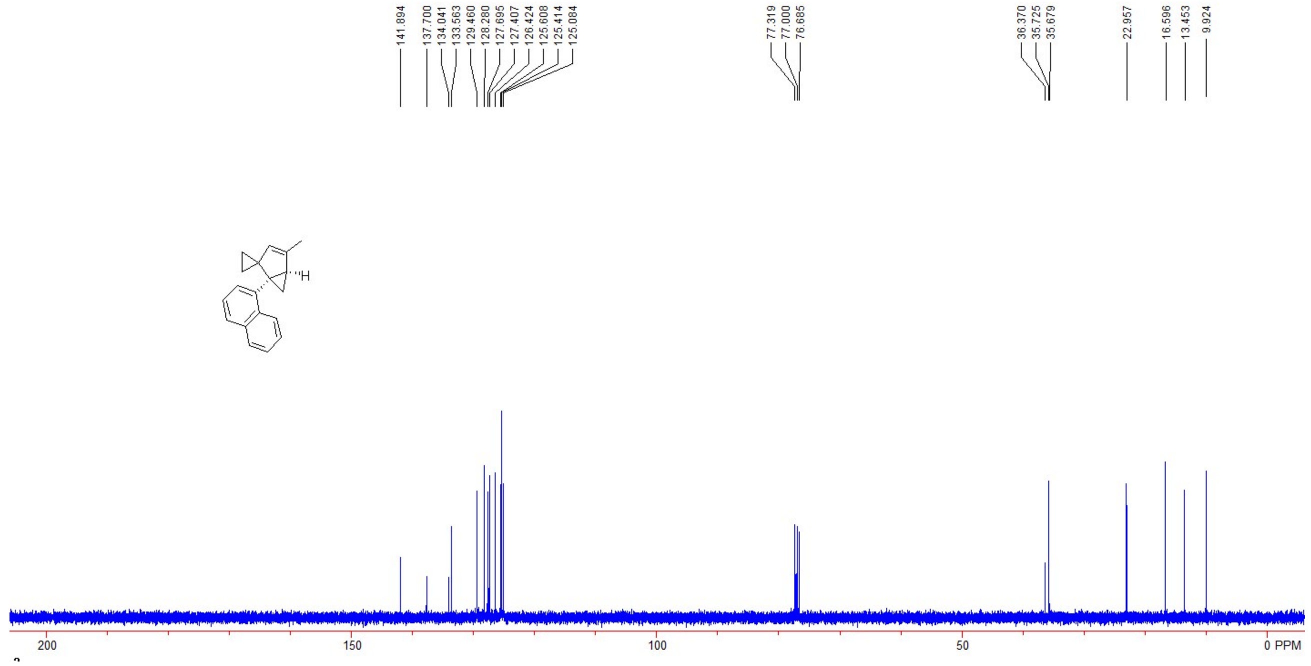

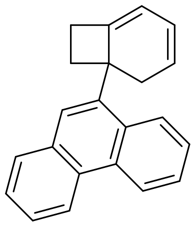

9-(Bicyclo[4.2.0]octa-3,5-dien-1-yl)phenanthrene 6a:

54 mg, yield = 95%. A white solid. Mp: 89-91 ^{o}C ^{1}H NMR (400 MHz, $CDCl_3$, TMS): δ 2.34-2.41 (m, 1H, CH_2), 2.54-2.59 (m, 1H, CH_2), 2.67-2.73 (m, 1H, CH_2), 2.76-2.86 (m, 3H, CH_2), 5.38-5.42 (m, 1H, =CH), 5.96-6.04 (m, 2H, =CH), 7.50-7.61 (m, 4H, Ar), 7.75 (s, 1H, Ar), 7.82 (d, J = 8.0 Hz , 1H, Ar), 7.89 (d, J = 8.4 Hz , 1H, Ar), 8.60 (d, J = 8.0 Hz, 1H, Ar), 8.72 (d, J = 8.0 Hz , 1H, Ar). ^{13}C NMR (100 MHz, $CDCl_3$, TMS): δ 30.2, 35.5, 36.2, 46.0, 114.9, 122.3, 123.8, 124.0, 124.7, 125.0, 125.69, 125.75, 125.8, 126.1, 126.6, 128.6, 129.0, 129.9, 131.5, 136.0, 141.7. IR (CH_2Cl_2) ν 3049, 2929, 1674, 1424, 1264, 1045, 907, 727, 703 cm^{-1}. MS (%) m/z 282 (M^+, 52.05), 267 (33.75), 254 (100.00), 239 (14.53), 226 (7.36), 202 (18.44), 191 (66.57), 165 (10.30), 126 (26.67). HRMS (EI) calcd. for $C_{22}H_{18}$: 282.1409, found: 282.1411.

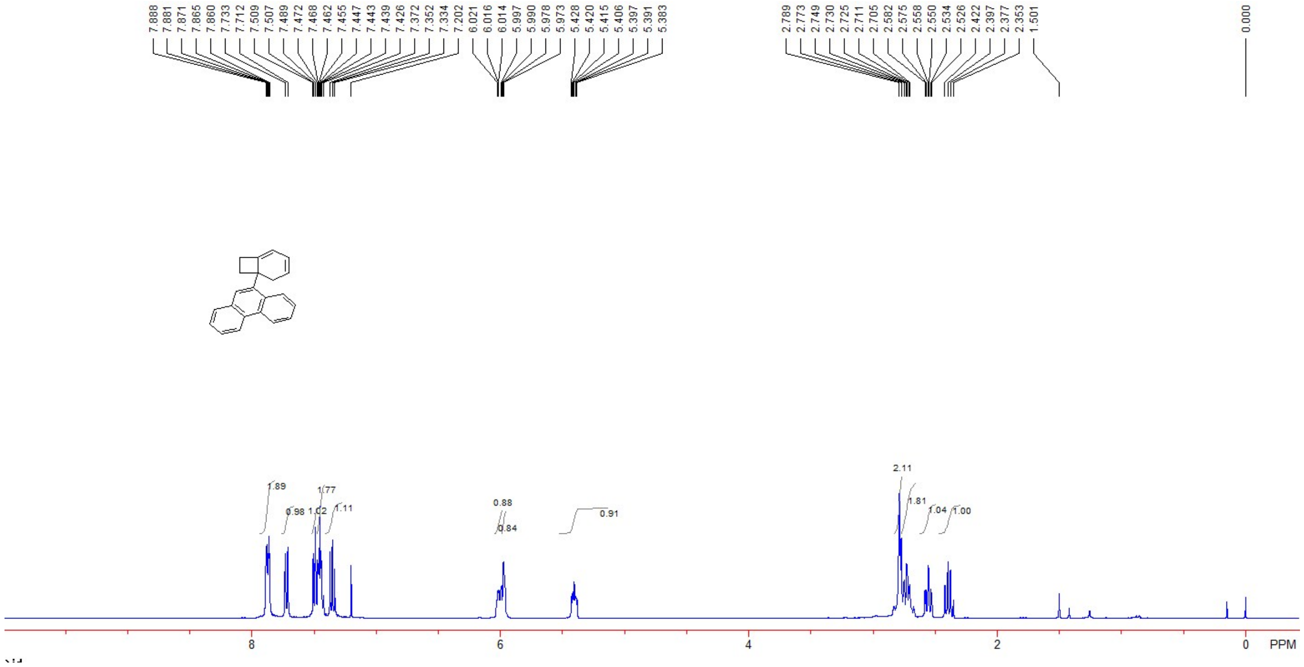

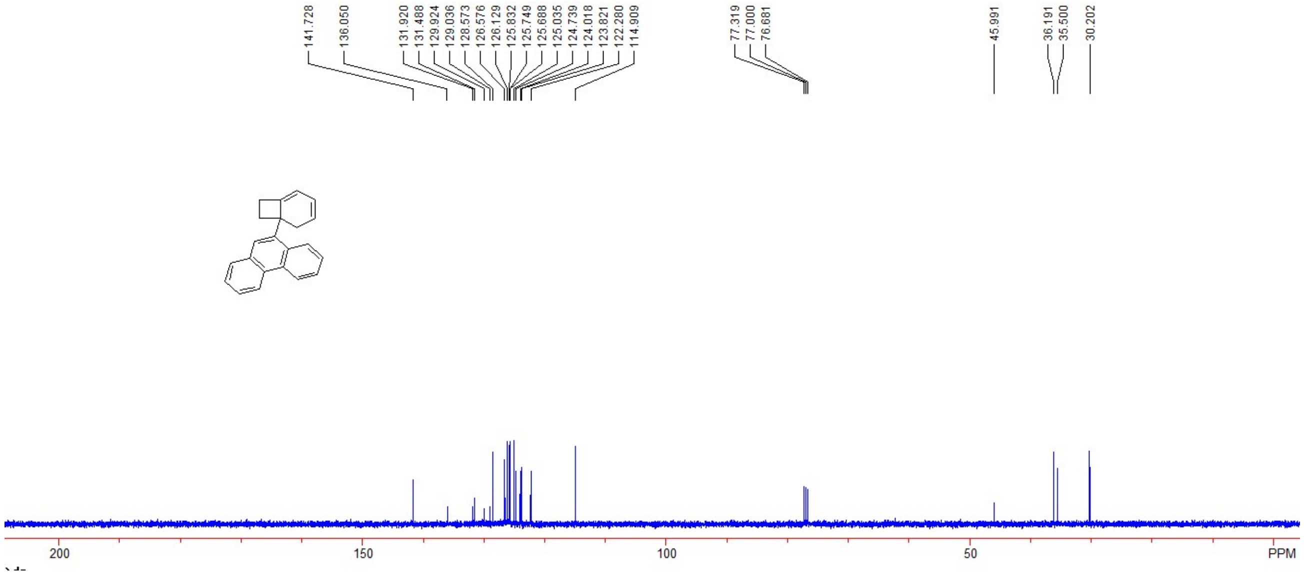

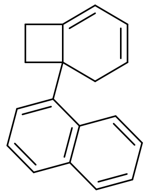

1-(Bicyclo[4.2.0]octa-3,5-dien-1-yl)naphthalene 6b:

40 mg, 87%. Colorless oil. ^{1}H NMR (400 MHz, $CDCl_3$, TMS): δ 2.34-2.41 (m, 1H, CH_2), 2.52-2.58 (m, 1H, CH_2), 2.70-2.72 (m, 2H, CH_2), 2.74-2.78 (m, 2H, CH_2), 5.38-5.42 (m, 1H, =CH), 5.96-6.02 (m, 2H, =CH), 7.33-7.37 (m, 1H, Ar), 7.42-7.45 (m, 2H, Ar), 7.46-7.51 (m, 1H, Ar), 7.71 (d, J = 8.4 Hz , 1H, Ar), 7.85-7.88 (m, 2H, Ar). ^{13}C NMR (100 MHz, $CDCl_3$, TMS): δ 30.2, 35.8, 36.7, 46.0, 114.7, 124.0, 124.5, 124.99, 125.02, 125.09, 125.13, 127.3, 129.5, 130.0, 135.0, 138.8, 142.0. IR

(CH_2Cl_2) ν 3040, 2932, 2855, 1674, 1508, 907, 777, 732, 669 cm^{-1}. MS (%) m/z 232 (M^+, 72.60), 215 (47.19), 204 (100.00), 189 (13.82), 178 (8.72), 141 (55.90), 115 (15.29), 101 (39.08), 77 (12.90). HRMS (EI) calcd. for $C_{18}H_{16}$: 232.1252, found: 232.1251.

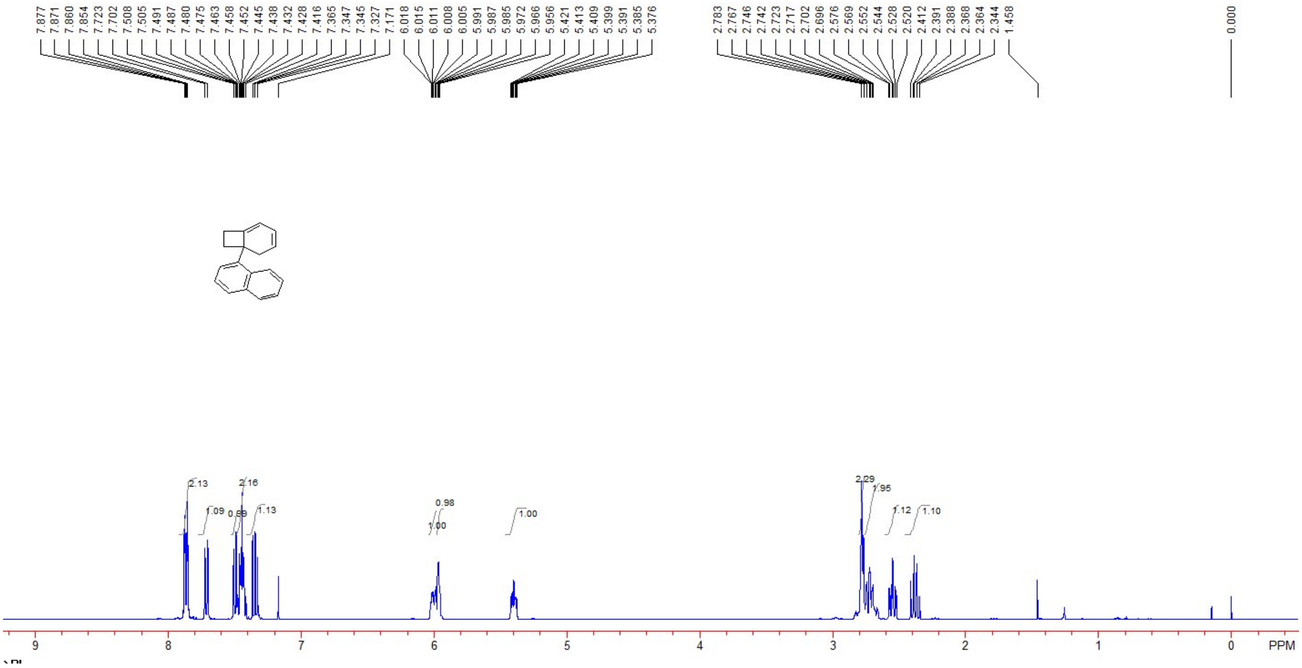

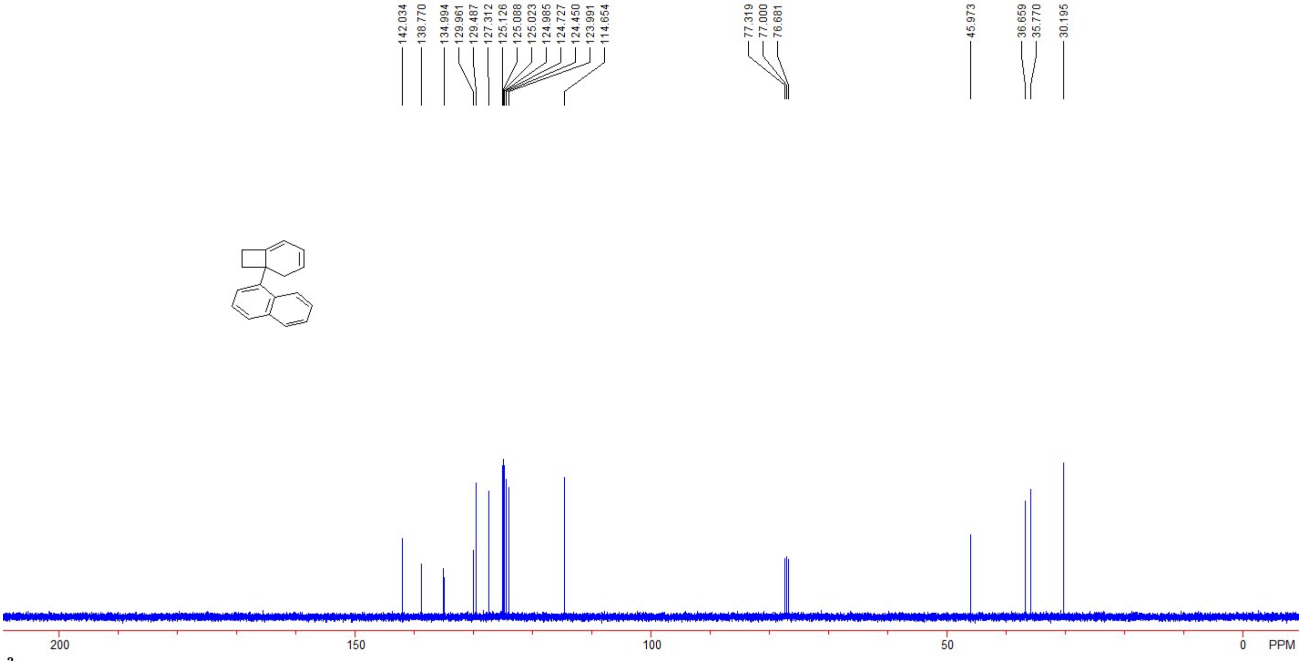

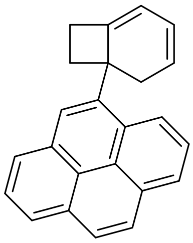

4-(Bicyclo[4.2.0]octa-3,5-dien-1-yl)pyrene 6c:

63 mg, yield = 90%. A white solid. Mp: 89-91 °C ^{1}H NMR (400 MHz, $CDCl_3$, TMS): δ 2.48-2.55 (m, 1H, CH_2), 2.64-2.70 (m, 1H, CH_2), 2.74-2.77 (m, 2H, CH_2), 2.80-2.95 (m, 2H, CH_2), 5.38-5.42 (m, 1H, =CH), 6.05-6.07 (m, 2H, =CH), 7.90-7.99 (m, 3H, Ar), 8.02-8.06 (m, 3H, Ar), 8.08-8.18 (m, 3H, Ar). ^{13}C NMR (100 MHz, $CDCl_3$, TMS): δ 30.3, 36.7, 37.7, 46.3, 114.9, 123.9, 124.2, 124.4, 124.8, 124.9, 125.0, 125.1, 125.3, 125.7, 126.5, 126.6, 127.4, 127.5, 130.4, 130.5, 131.2，138.1, 142.2. IR (CH_2Cl_2) ν 3037, 2931, 2854, 1422, 1263, 845, 752, 665 cm^{-1}. MS (%) m/z 306 (M^+, 100.00), 289 (69.03), 278 (84.36), 239 (13.08), 215 (81.69), 202 (9.83), 145 (33.84), 138 (40.15), 117 (12.13). HRMS (EI) calcd. for $C_{24}H_{18}$: 306.1409, found: 306.1414.

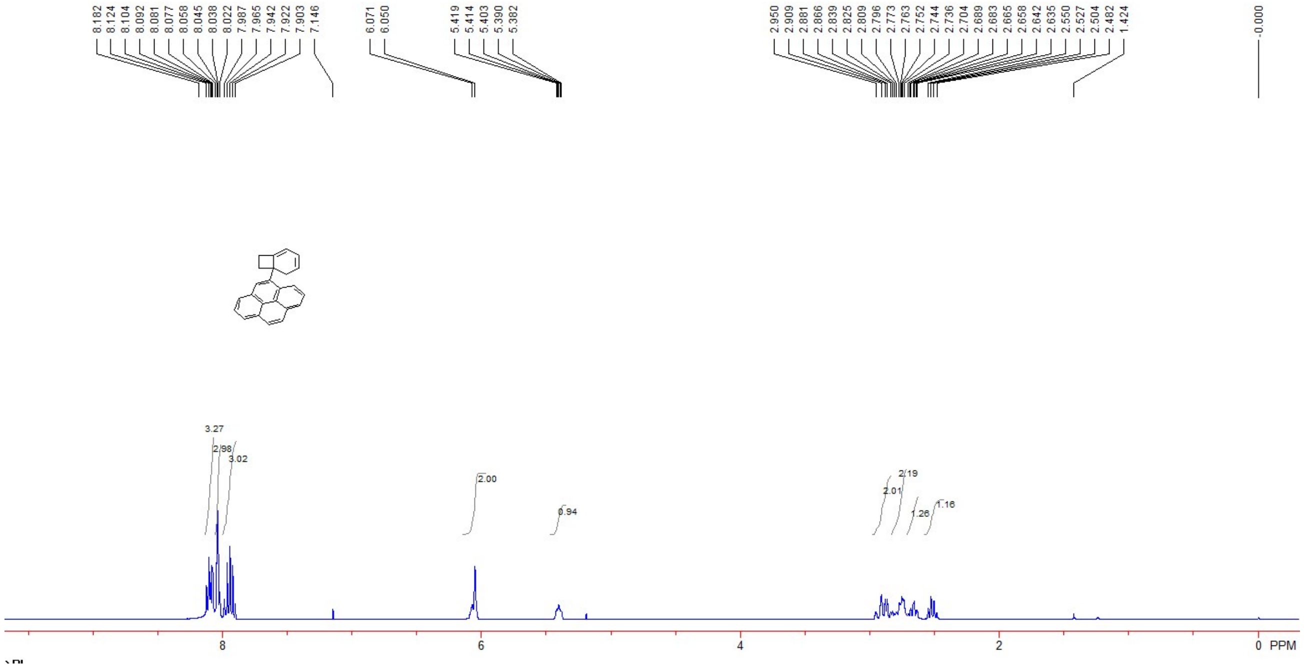

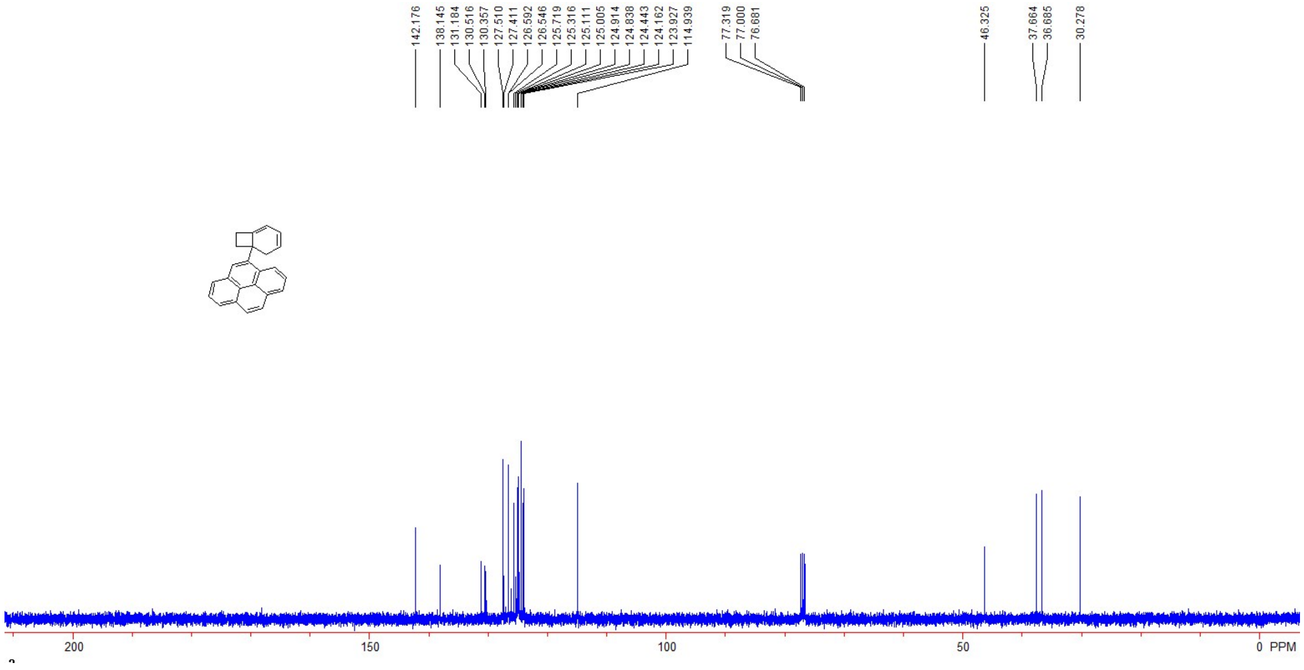

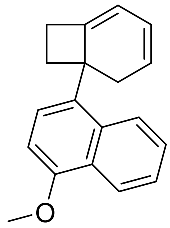

1-(Bicyclo[4.2.0]octa-3,5-dien-1-yl)-4-methoxynaphthalene 6d:

48 mg, yield = 92%. A white solid. Mp: 72-74 °C. ^{1}H NMR (400 MHz, $CDCl_3$, TMS): δ 2.29-2.36 (m, 1H, CH_2), 2.48-2.54 (m, 1H, CH_2), 2.65-2.76 (m, 4H, CH_2), 3.92 (s, 3H, CH_3), 5.39-5.42 (m, 1H, =CH), 5.94-6.00 (m, 2H, =CH), 6.64-6.67 (m, 1H, Ar), 7.39-7.49 (m, 3H, Ar), 7.79-7.82 (m, 1H, Ar), 8.32-8.35 (m, 1H, Ar). ^{13}C NMR (100 MHz, $CDCl_3$, TMS): δ 30.1, 35.5, 36.6, 45.4, 55.3, 102.6, 114.4, 123.3, 124.1, 124.3, 124.4, 124.7, 124.8, 125.6, 127.1, 130.2, 130.8, 142.2, 154.6. IR (CH_2Cl_2) ν 3035, 2934, 2838, 1586, 1460, 1234, 1086, 816, 764, 666 cm^{-1}. MS (%) m/z 262 (M^+, 90.56), 247 (25.91), 234 (100.00), 215 (47.10), 202 (33.07), 191 (36.47), 171 (66.66), 139 (20.59), 115 (23.22), 101 (24.25). HRMS (EI) calcd. for $C_{19}H_{18}O$: 262.1358, found: 262.1357.

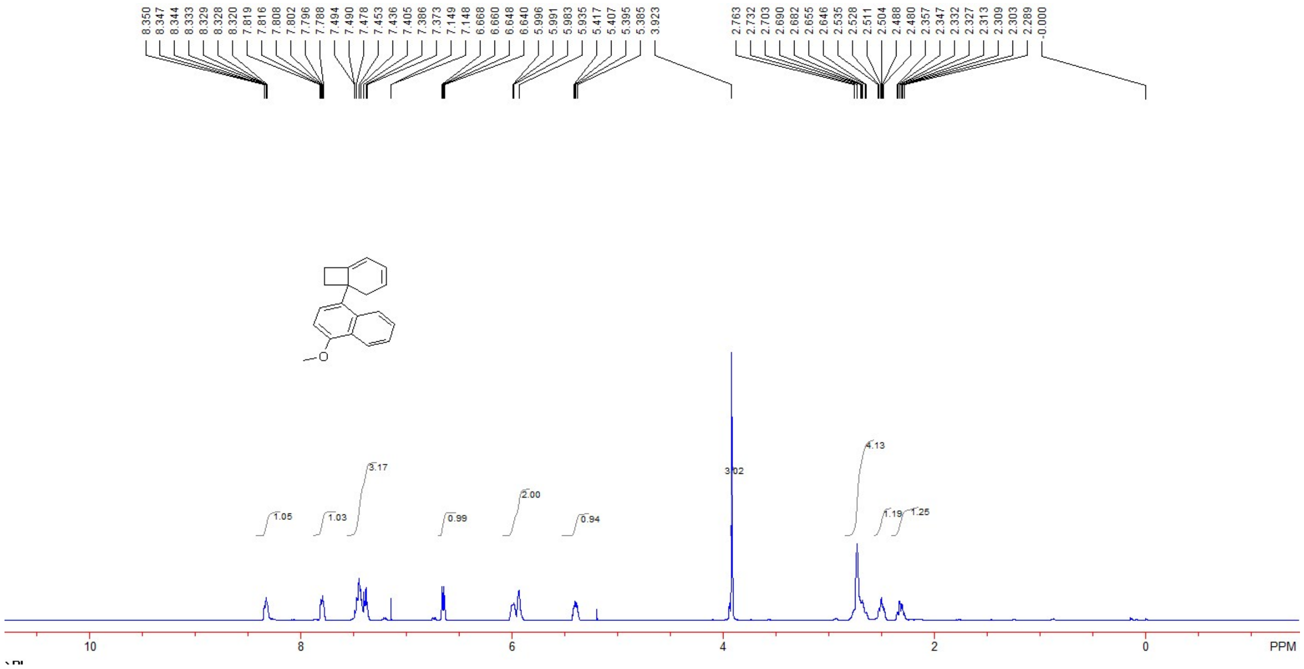

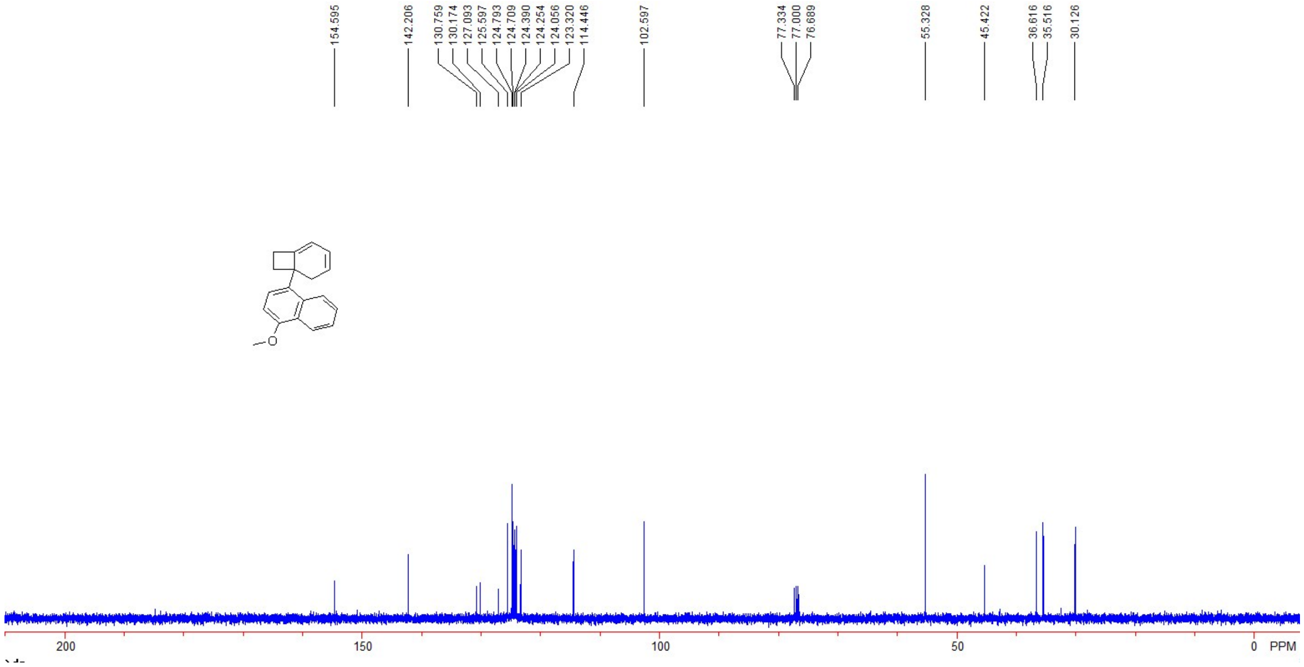

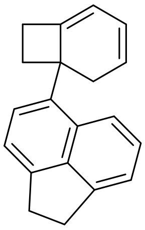

5-(Bicyclo[4.2.0]octa-3,5-dien-1-yl)-1,2-dihydroacenaphthylene 6e:

42 mg, yield = 81%. A white solid, mp = 85-87 °C. ^{1}H NMR (400 MHz, $CDCl_3$, TMS): δ 2.29-2.35 (m, 1H, CH_2), 2.50-2.55 (m, 1H, CH_2), 2.67-2.80 (m, 4H, CH_2), 3.32-3.38 (m, 4H, CH_2), 5.38-5.43 (m, 1H, =CH), 5.93-5.95 (m, 1H, =CH), 5.99-6.02 (m, 1H, =CH), 7.15 (d, J = 6.8 Hz, 1H, Ar), 7.26 (d, J = 6.8 Hz, 1H, Ar), 7.40-7.44 (m, 2H, Ar), 7.56 (d, J = 8.4 Hz, 1H, Ar). ^{13}C NMR (100 MHz, $CDCl_3$, TMS): δ 29.8, 30.2, 30.6, 35.1, 36.2, 45.6, 114.3, 118.5, 118.8, 120.4, 123.8, 124.8, 125.7, 126.9, 128.5, 134.6, 140.6, 142.4, 144.9, 147.2. IR (CH_2Cl_2) ν 3028, 2931, 2837, 1607, 1423, 1271, 837, 749, 718, 667 cm^{-1}. MS (%) m/z 258 (M^+, 99.23), 243 (44.16), 230 (100.00), 215 (25.79), 202 (20.12), 189 (13.09), 167 (56.15), 152 (30.49), 114 (15.94), 101 (14.19). HRMS (EI) calcd. for $C_{20}H_{18}$: 258.1409, found: 258.1410.

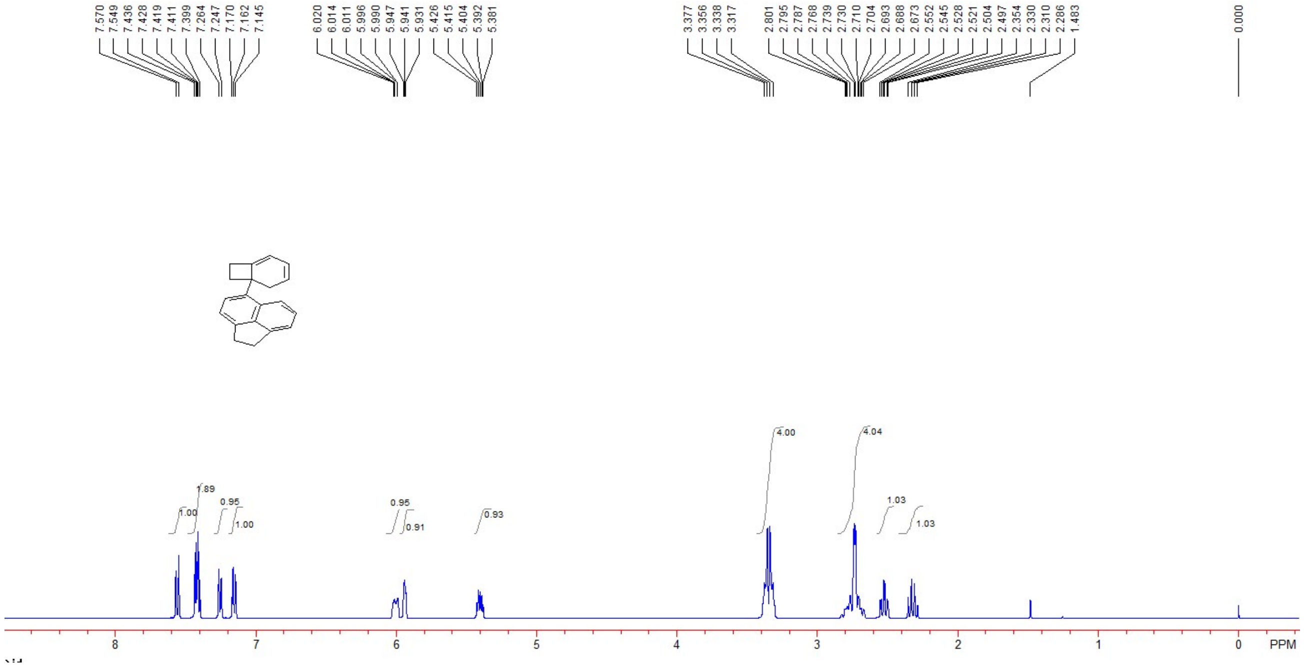

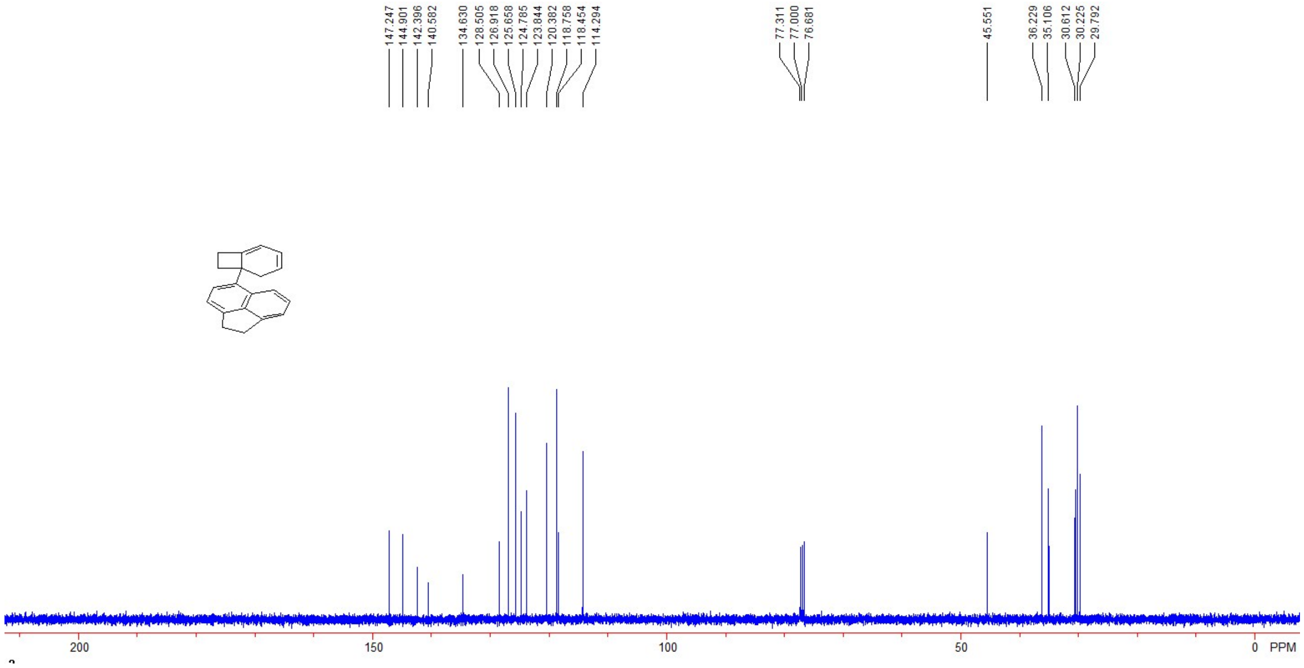

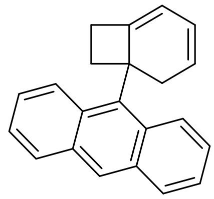

9-(Bicyclo[4.2.0]octa-3,5-dien-1-yl)anthracene 6f:

48 mg, yield = 84%. Colorless oil. ^{1}H NMR (400 MHz, $CDCl_3$, TMS): δ 2.45-2.51 (m, 1H, CH_2), 2.66-2.71 (m, 2H, CH_2), 2.75-2.79 (m, 1H, CH_2), 3.00-3.06 (m, 1H, CH_2), 3.13-3.17 (m, 1H, CH_2), 5.64-5.68 (m, 1H, =CH), 6.04 (br, 1H, =CH), 6.19 (t, J = 4.8 Hz, 1H, =CH), 7.27-7.35 (m, 2H, Ar),

7.38-7.45 (m, 2H, Ar), 7.87 (d, *J* = 8.0 Hz, 2H, Ar), 7.93 (d, *J* = 8.8 Hz, 1H, Ar), 7.97 (d, *J* = 7.6 Hz, 1H, Ar), 8.29 (s, 1H, Ar), 9.33 (d, *J* = 8.8 Hz, 1H, Ar). ^{13}C NMR (100 MHz, $CDCl_3$, TMS): δ 32.3, 40.1, 43.3, 48.7, 114.0, 124.2, 124.3, 124.6, 124.8, 125.3, 125.4, 125.8, 126.9, 127.1, 128.5, 129.9, 131.3, 131.9, 132.4, 139.4, 144.2. IR (CH_2Cl_2) ν 3043, 2929, 2851, 1673, 1023, 906, 730, 675 cm^{-1}. MS (%) m/z 282 (M^+, 41.10), 167 (13.36), 254 (31.96), 239 (9.74), 215 (7.99), 191 (100.00), 178 (6.42), 126 (19.62), 113 (8.15). HRMS (EI) calcd. for $C_{22}H_{18}$: 282.1409, found: 282.1410.

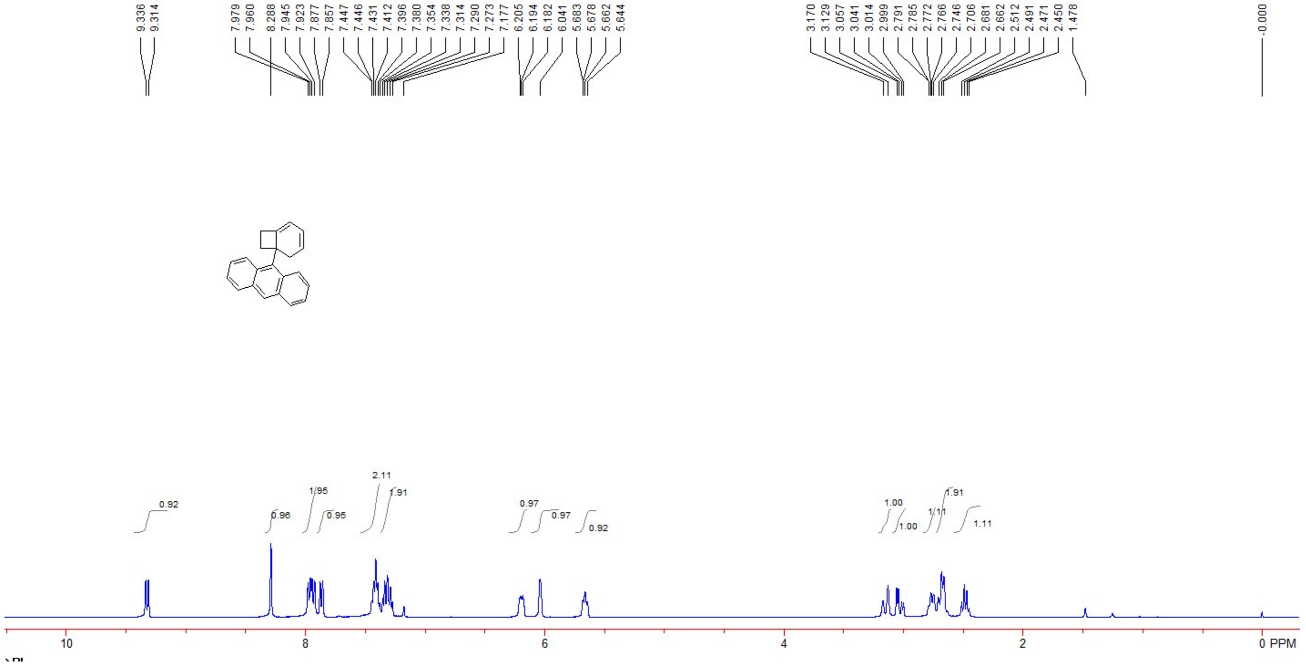

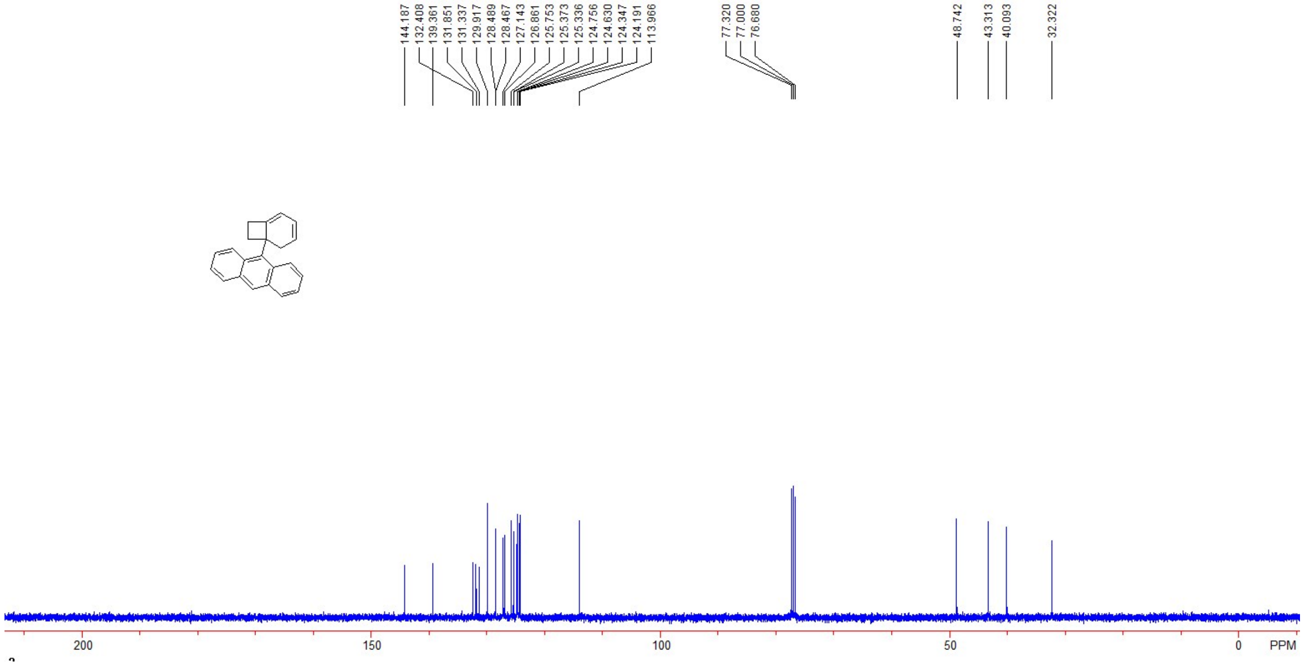

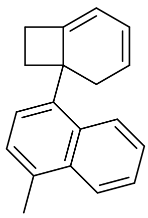

1-(Bicyclo[4.2.0]octa-3,5-dien-1-yl)-4-methylnaphthalene 6g:

45 mg, yield = 86%. Yellowish oil. ^{1}H NMR (400 MHz, $CDCl_3$, TMS): δ 2.34-2.41 (m, 1H, CH_2), 2.51-2.57 (m, 1H, CH_2), 2.66-2.69 (m, 2H, CH_2), 2.67 (s, 3H, CH_3), 2.72-2.78 (m, 2H, CH_2), 5.38-5.42 (m, 1H, =CH), 5.96-6.02 (m, 2H, =CH), 7.18-7.21 (m, 1H, Ar), 7.39-7.41 (m, 1H, Ar), 7.46-7.52 (m, 2H, Ar), 7.88-7.90 (m, 1H, Ar), 8.03-8.06 (m, 1H, Ar). ^{13}C NMR (100 MHz, $CDCl_3$, TMS): δ 19.5, 30.2, 35.8, 36.8, 45.8, 114.6, 124.0, 124.2, 124.72, 124.73, 124.9, 125.57, 125.61, 125.9, 130.0, 133.3, 134.1, 136.8, 142.1. IR (CH_2Cl_2) ν 3033, 2933, 2857, 1673, 1596, 1514, 905, 727 cm^{-1}. MS (%) m/z 246 (M^+, 41.63), 231 (85.76), 218 (100.00), 203 (43.45), 189 (13.94), 155 (48.11), 115 (19.36), 108 (21.49), 94 (7.49). HRMS (EI) calcd. for $C_{19}H_{18}$: 246.1409, found: 246.1403.

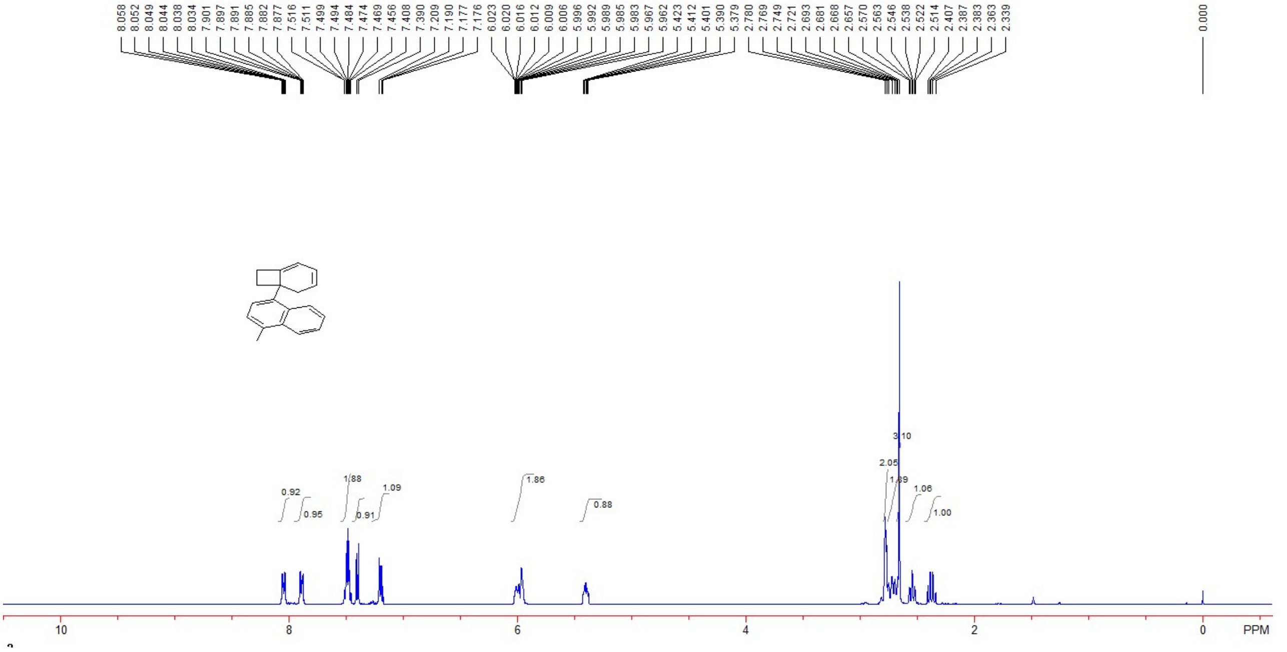

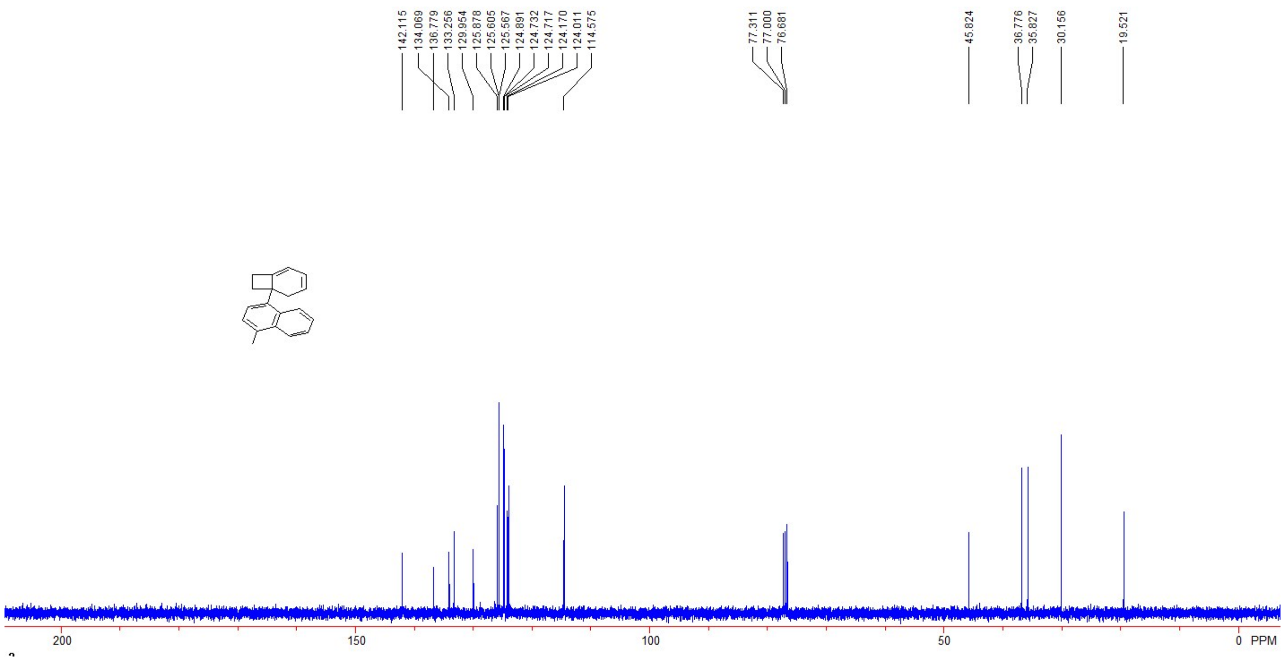

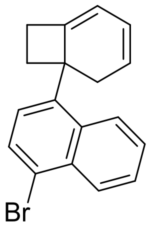

1-(Bicyclo[4.2.0]octa-3,5-dien-1-yl)-4-bromonaphthalene 6h:

56 mg, yield = 90%. Colorless oil. ^{1}H NMR (400 MHz, $CDCl_3$, TMS): δ 2.37-2.43 (m, 1H, CH_2), 2.49-2.55 (m, 1H, CH_2), 2.69-2.83 (m, 4H, CH_2), 5.37-5.42 (m, 1H, =CH), 5.98-6.03 (m, 2H, =CH), 7.34 (d, J = 7.6 Hz, 1H, Ar), 7.50-7.58 (m, 2H, Ar), 7.66 (d, J = 7.6 Hz, 1H, Ar), 7.86-7.88 (m, 1H, Ar), 8.31-8.33 (m, 1H, Ar). ^{13}C NMR (100 MHz, $CDCl_3$, TMS): δ 30.2, 35.8, 36.7, 45.8, 115.1, 121.9, 124.0, 124.8, 125.2, 125.5, 125.9, 126.5, 128.7, 129.3, 131.4, 133.2, 139.1, 141.6. IR (CH_2Cl_2) ν 3077, 2934, 2857, 1673, 1372, 906, 758, 730, 671 cm^{-1}. MS (%) m/z 310 (M^+, 22.04), 282 (63.86), 231 (97.14), 215 (100.00), 202 (85.77), 189 (15.73), 152 (22.52), 108 (32.77), 101 (31.00), 77 (9.59). HRMS (EI) calcd. for $C_{18}H_{15}Br$: 310.0357, found: 310.0351.

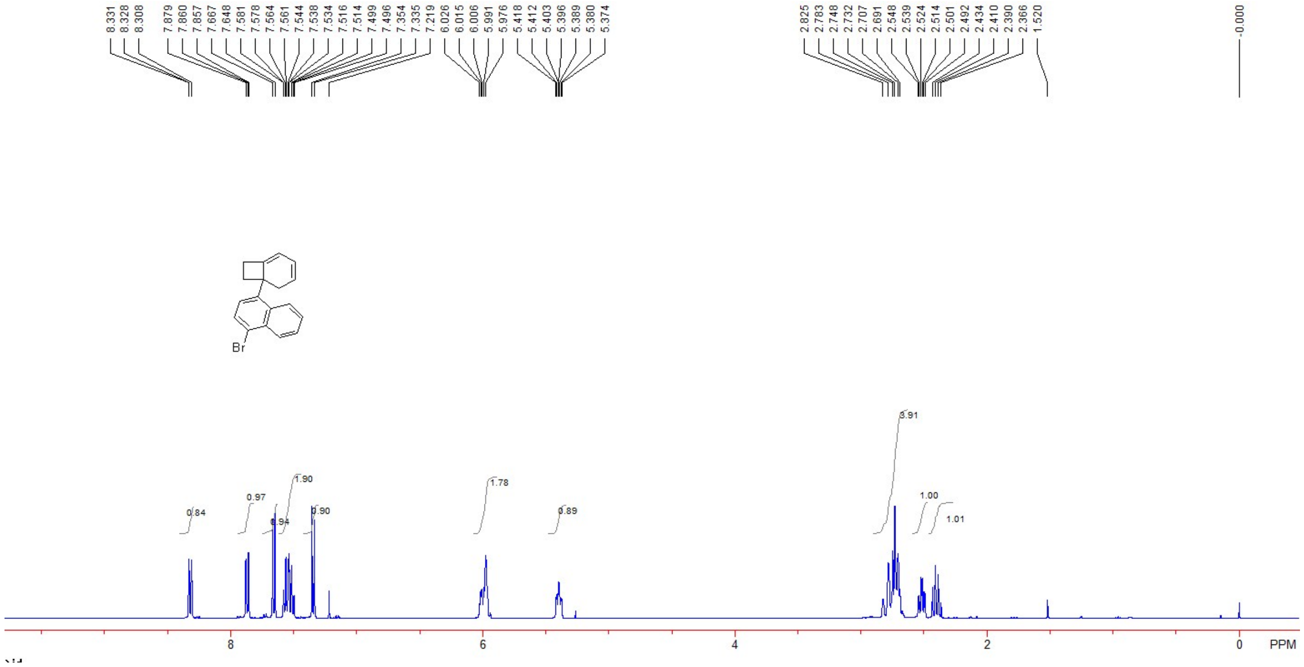

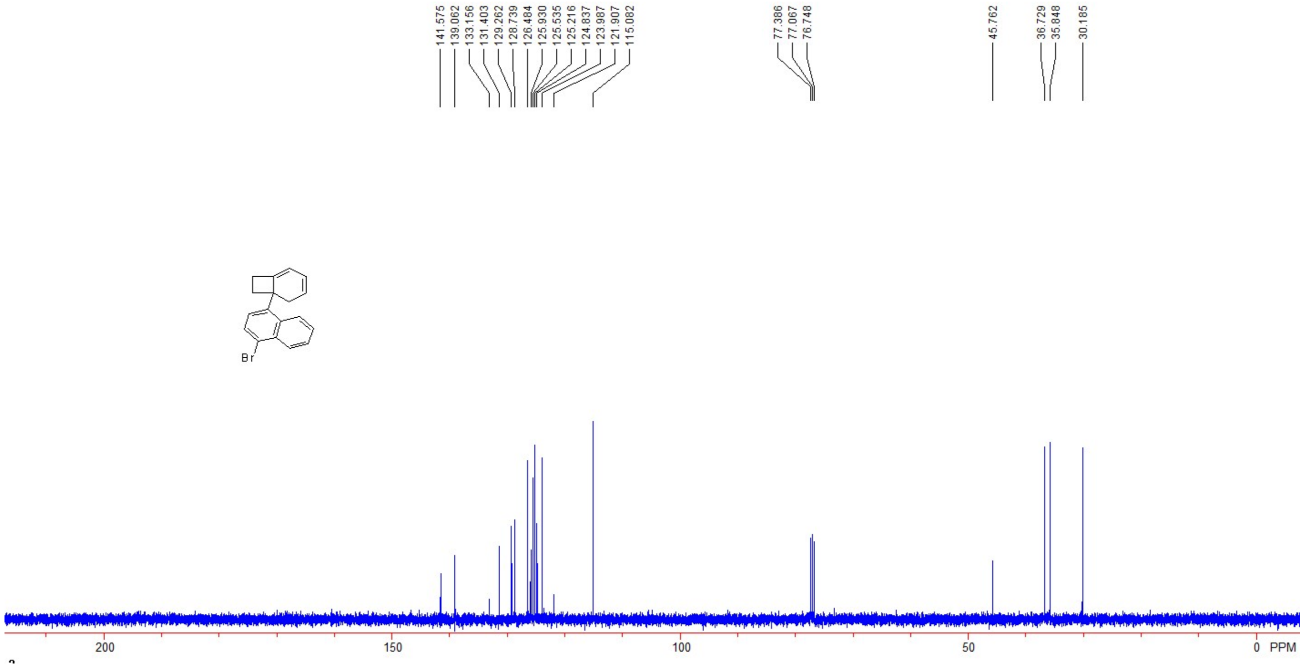

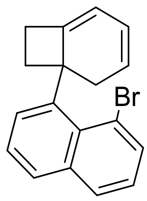

1-(Bicyclo[4.2.0]octa-3,5-dien-1-yl)-8-bromonaphthalene 6i:

40 mg, yield = 64%. Colorless oil. ^{1}H NMR (400 MHz, $CDCl_3$, TMS): δ 2.40-2.52 (m, 3H, CH_2), 2.82-2.92 (m, 2H, CH_2), 3.16-3.22 (m, 1H, CH_2), 5.43-5.47 (m, 1H, =CH), 5.94 (d, J = 4.0 Hz, 1H, =CH), 6.03-6.07 (m, 1H, =CH), 7.21 (dd, J_1 = 8.4 Hz, J_2 = 7.2 Hz, 1H, Ar), 7.35 (dd, J_1 = 8.0 Hz, J_2

= 7.2 Hz, 1H, Ar), 7.73-7.76 (m, 2H, Ar), 7.81-7.83 (m, 1H, Ar), 7.91 (dd, J_1 = 7.2 Hz, J_2 = 1.2 Hz, 1H, Ar). ^{13}C NMR (100 MHz, $CDCl_3$, TMS): δ 28.8, 39.5, 40.3, 46.6, 114.5, 117.7, 124.0, 124.7, 125.1, 125.5, 128.5, 129.3, 130.2, 130.6, 134.4, 137.6, 137.9, 143.3. IR (CH_2Cl_2) ν 3033, 2952, 2854, 1560, 1422, 1192, 816, 762, 724 cm^{-1}. MS (%) m/z 310 (M^+, 4.45), 282 (24.37), 231 (12.41), 215 (38.51), 203 (100.00), 152 (7.54), 113 (7.76), 101 (23.27). HRMS (EI) calcd. for $C_{18}H_{15}Br$: 310.0357, found: 310.0353.

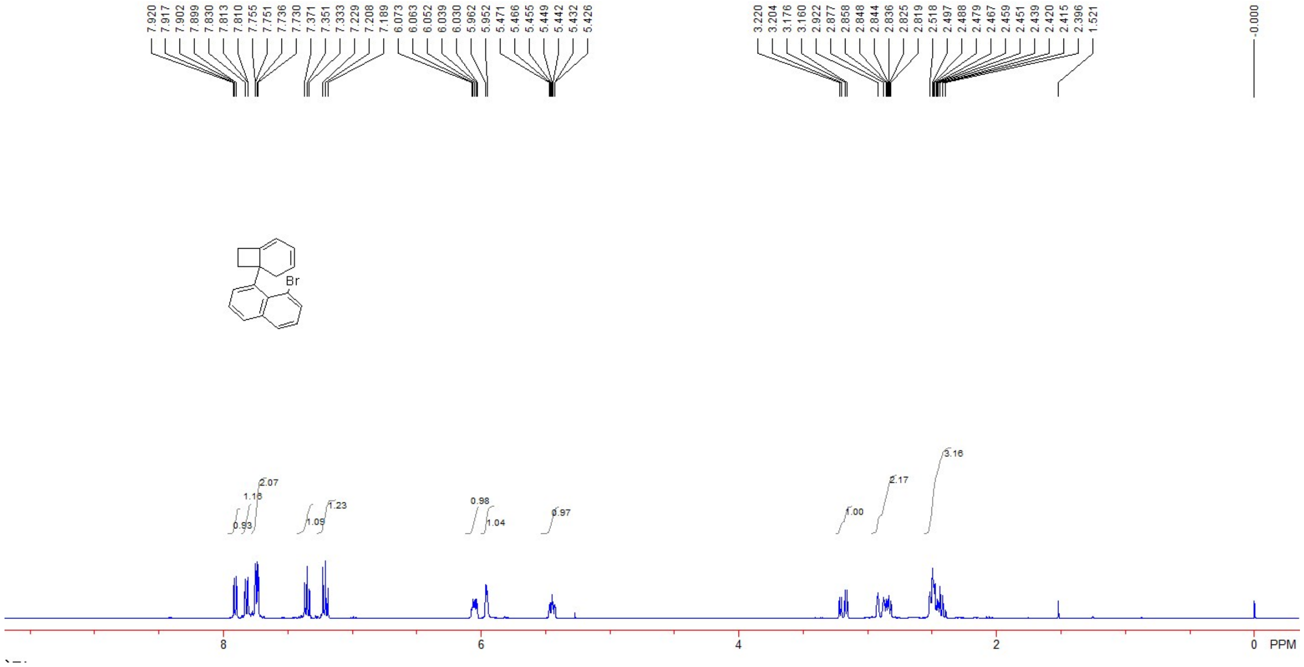

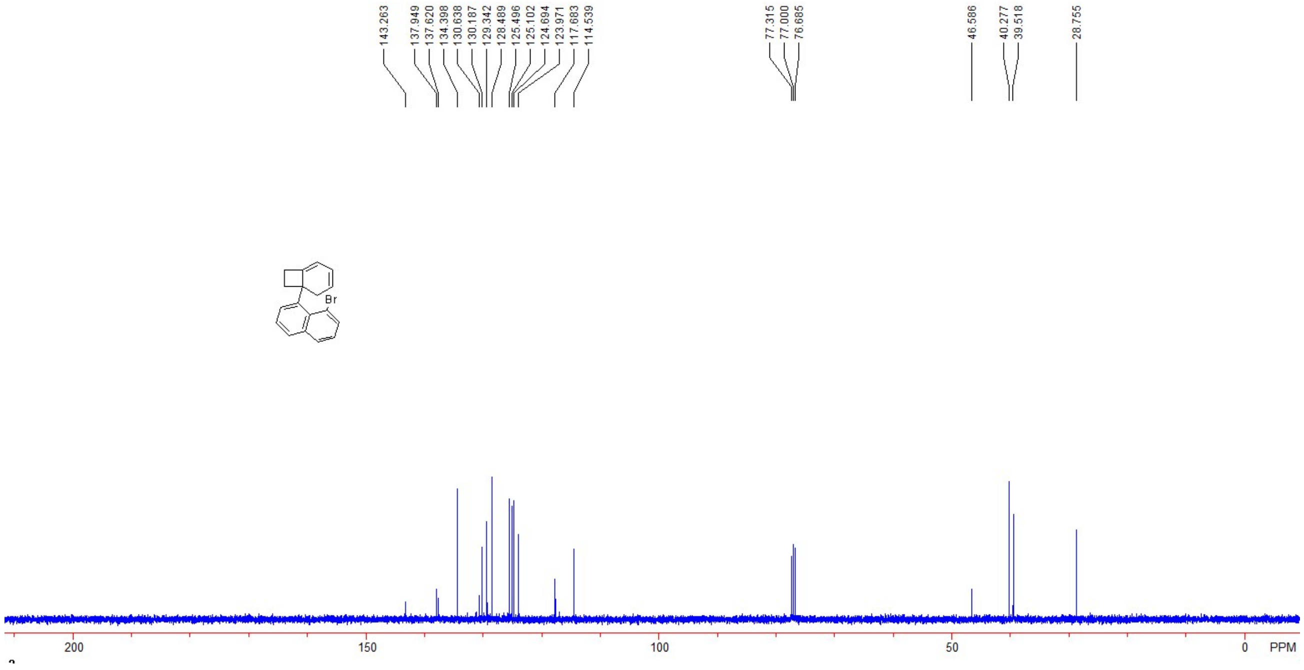

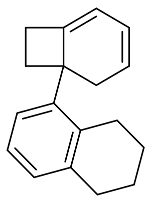

5-(Bicyclo[4.2.0]octa-3,5-dien-1-yl)-1,2,3,4-tetrahydronaphthalene 6j:

35 mg, yield = 75%. Colorless oil. ^{1}H NMR (400 MHz, $CDCl_3$, TMS): δ 1.51-1.82 (m, 4H, CH_2), 2.09-2.16 (m, 1H, CH_2), 2.30-2.36 (m, 1H, CH_2), 2.55-2.72 (m, 5H, CH_2), 2.79-2.82 (m, 2H, CH_2), 5.42 (t, *J* = 6.8 Hz, 1H =CH), 5.89 (br, 1H, =CH), 5.98-6.01 (m, 1H, =CH), 6.97-7.03 (m, 2H, Ar), 7.15 (d, *J* = 7.2 Hz, 1H, Ar). ^{13}C NMR (100 MHz, $CDCl_3$, TMS): δ 22.76, 22.80, 27.2, 29.4, 29.7, 34.9, 35.7, 45.9, 114.3, 123.5, 124.6, 124.8, 125.0, 127.7, 135.9, 138.6, 140.5, 142.4. IR (CH_2Cl_2) ν 3035, 2929, 2855, 1674, 1583, 1461, 776, 750, 664 cm^{-1}. MS (%) m/z 236 (M^+, 84.89), 208 (100.00), 193 (56.10), 179 (63.09), 165 (55.72), 145 (20.82), 128 (23.64), 115 (28.80), 103 (9.95), 89 (23.91). HRMS (EI) calcd. for $C_{18}H_{20}$: 236.1565, found: 236.1562.

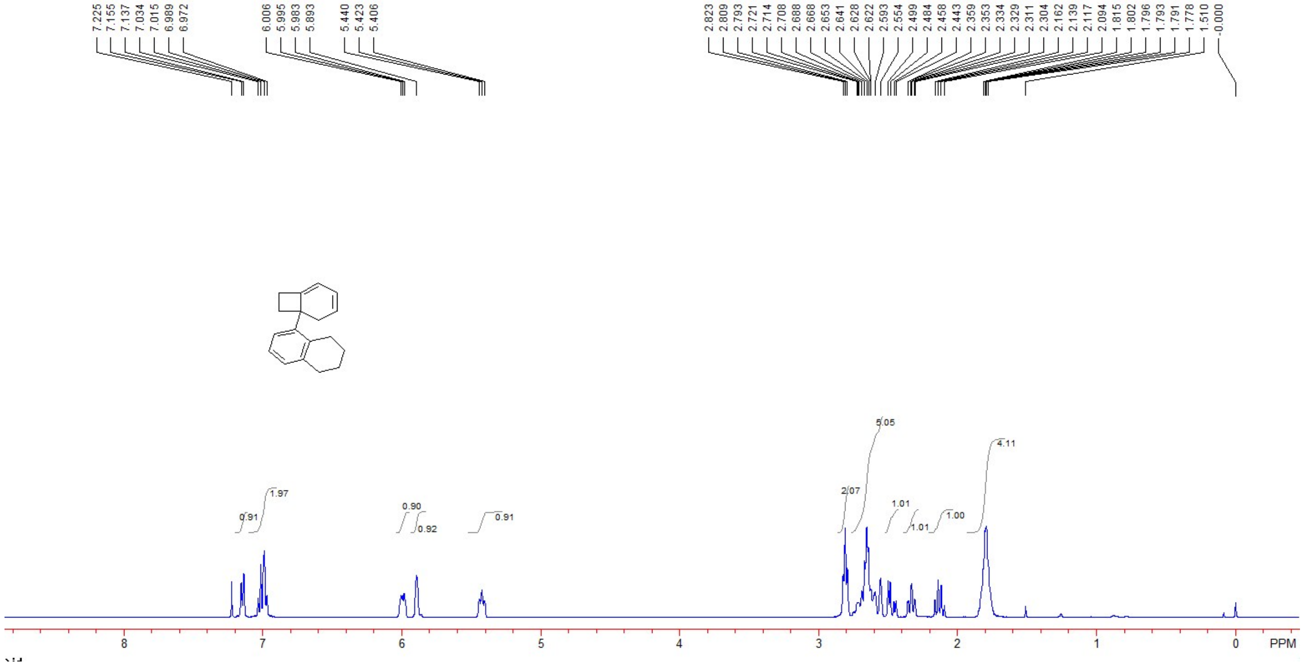

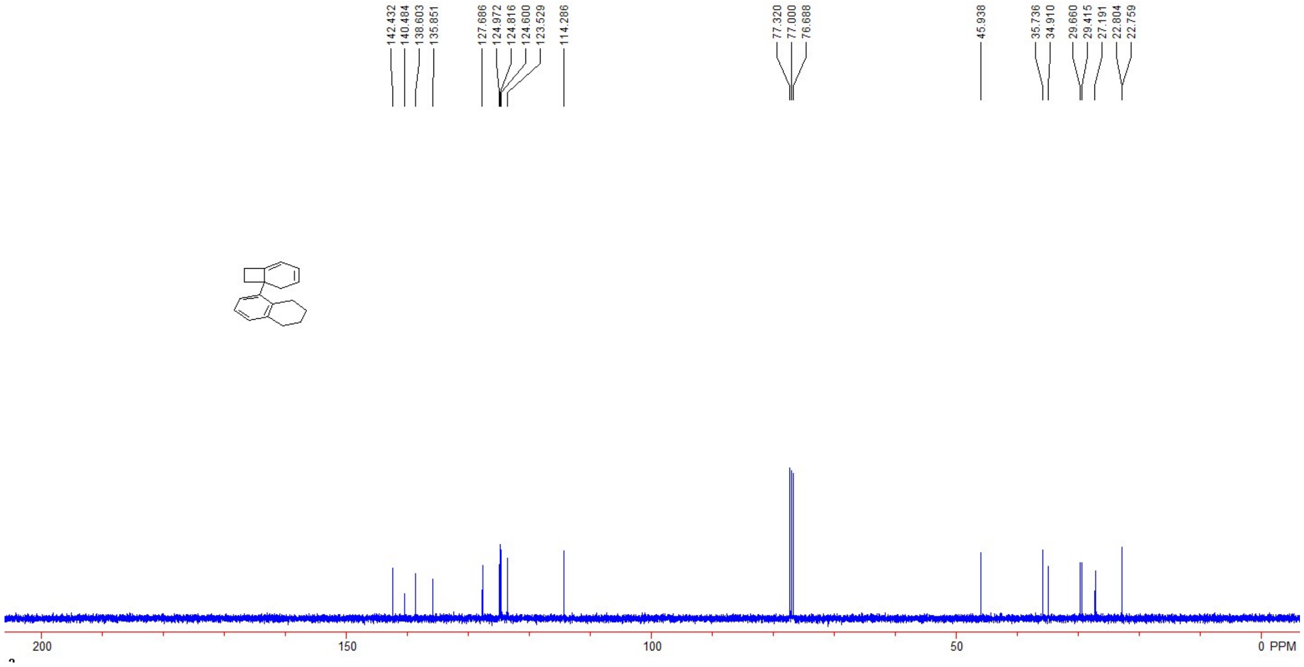

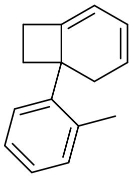

6-(o-Tolyl)bicyclo[4.2.0]octa-1,3-diene 6k:

34 mg, yield = 85%. Colorless oil. ^{1}H NMR (400 MHz, $CDCl_3$, TMS): δ 2.12-2.19 (m, 1H, CH_2), 2.30-2.39 (m, 1H, CH_2), 2.36 (s, 3H, CH_3), 2.43-2.49 (m, 1H, CH_2), 2.58-2.73 (m, 3H, CH_2), 5.40-5.45 (m, 1H, =CH), 5.91 (br, 1H, =CH), 5.99-6.03 (m, 1H, =CH), 7.08 (dd, J_1 = 7.6 Hz, J_2 = 5.6 Hz, 1H, Ar), 7.13-7.20 (m, 2H, Ar), 7.28 (dd, J_1 = 6.4 Hz, J_2 = 1.2 Hz, 1H, Ar). ^{13}C NMR (100 MHz, $CDCl_3$, TMS): δ 19.6, 29.4, 34.8, 35.7, 45.9, 114.4, 123.4, 125.0, 125.1, 126.5, 127.2, 131.6, 135.8, 141.3, 142.1. IR (CH_2Cl_2) ν 3058, 3016, 2925, 1674, 1484, 1449, 1046, 757, 730 cm^{-1}. MS (%) m/z 196 (M^+, 27.72), 181 (100.00), 168 (82.54), 153 (35.98), 128 (17.67), 115 (33.17), 105 (27.98), 89 (28.86), 73 (44.02). HRMS (EI) calcd. for $C_{15}H_{16}$: 196.1252, found: 196.1246.

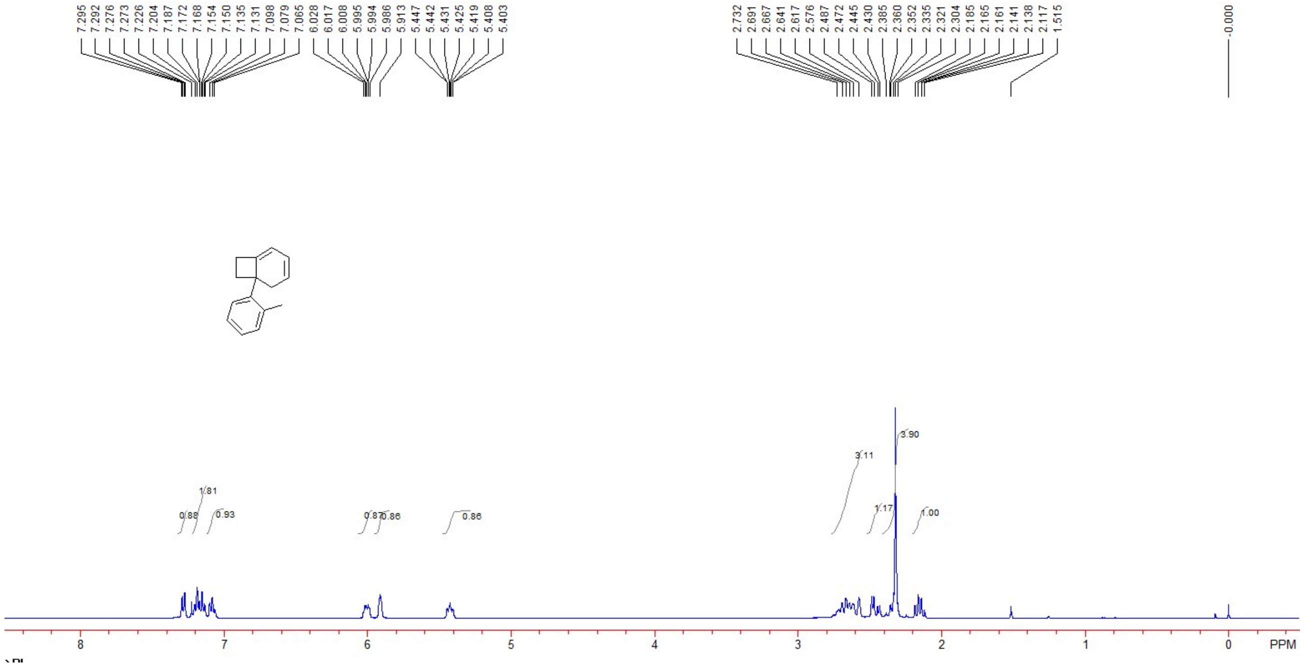

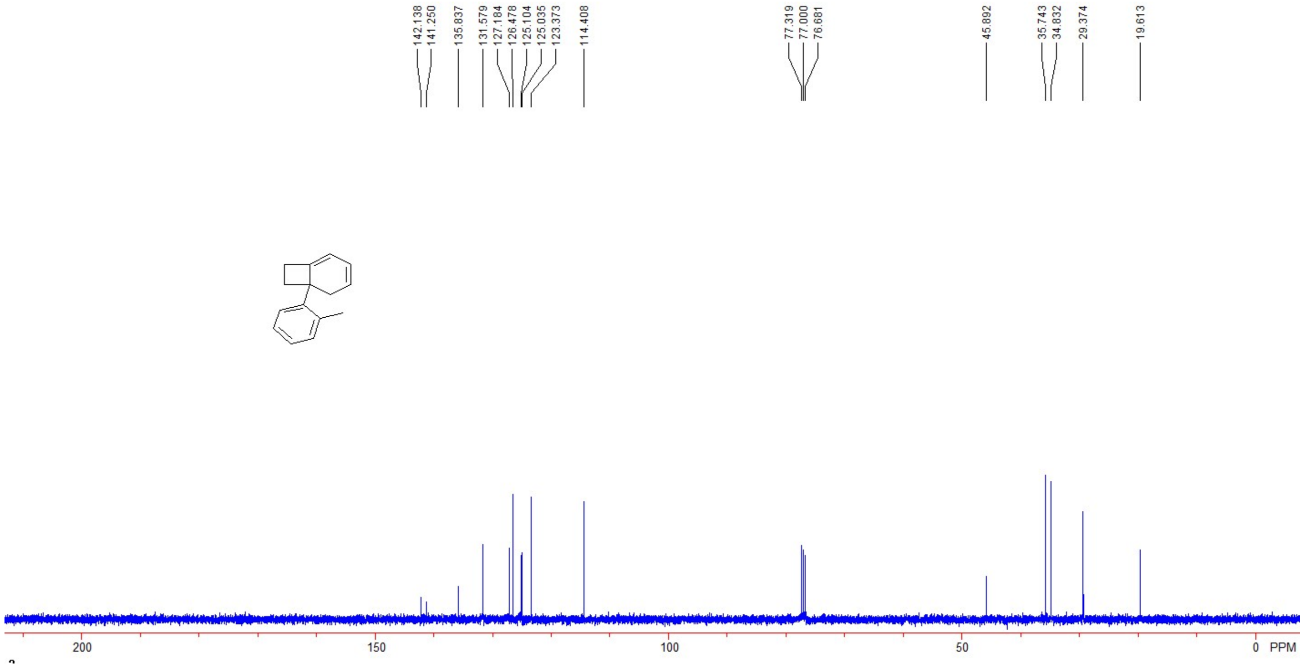

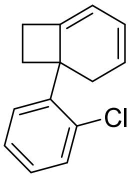

6-(2-chlorophenyl)bicyclo[4.2.0]octa-1,3-diene 6l:

38 mg, yield = 88%. Colorless oil. ^{1}H NMR (400 MHz, $CDCl_3$, TMS): δ 2.15-2.22 (m, 1H, CH_2), 2.49-2.51 (m, 2H, CH_2), 2.52-2.74 (m, 3H, CH_2), 5.44 (m, 1H, =CH), 5.92-5.99 (m, 1H, =CH), 6.00-6.02 (m, 1H, =CH), 7.14-7.20 (m, 2H, Ar), 7.32-7.38 (m, 2H, Ar). ^{13}C NMR (100 MHz, $CDCl_3$,

TMS): δ 29.3, 34.4, 34.8, 45.9, 115.0, 123.9, 124.9, 126.2, 127.8, 129.0, 130.7, 133.7, 140.3, 141.1. IR (CH_2Cl_2) ν 3036, 2951, 2817, 1467, 1423, 1030, 830, 748, 668 cm^{-1}. MS (%) m/z 216 (M^+, 2.11), 201 (10.96), 188 (100.00), 181 (33.70), 165 (50.76), 152 (34.68), 115 (7.55), 89 (15.49), 76 (12.02). HRMS (EI) calcd. for $C_{14}H_{13}Cl$: 216.0706, found: 216.0708.

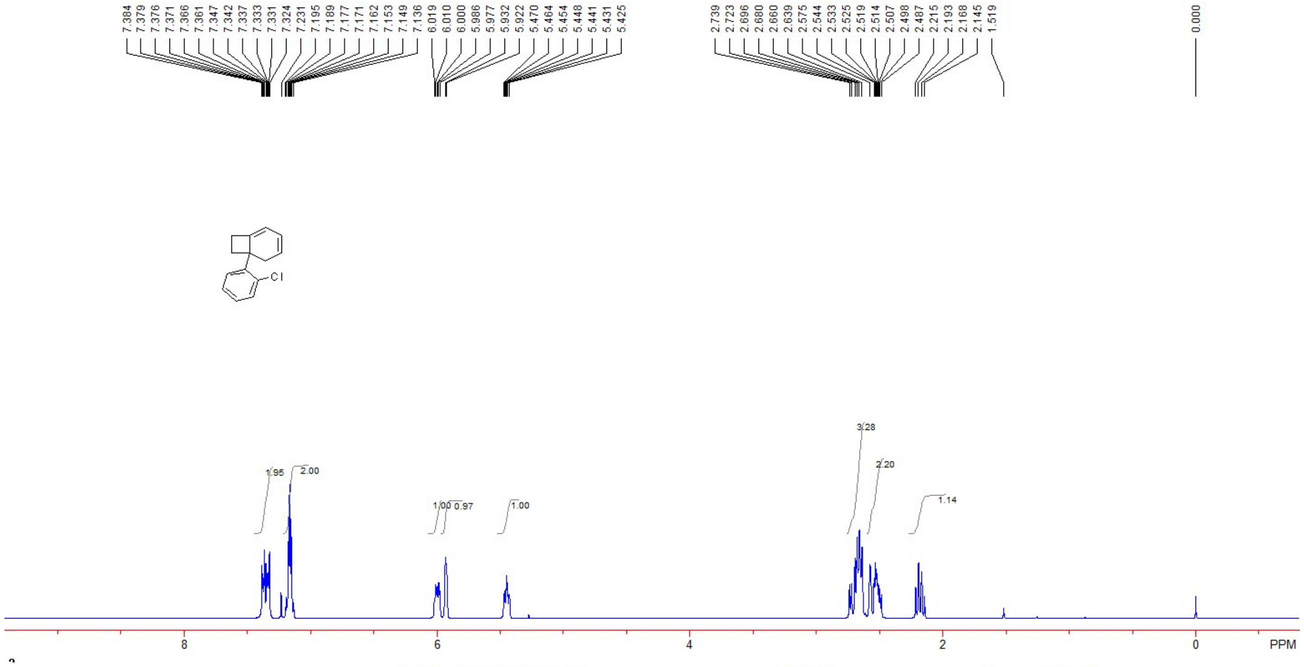

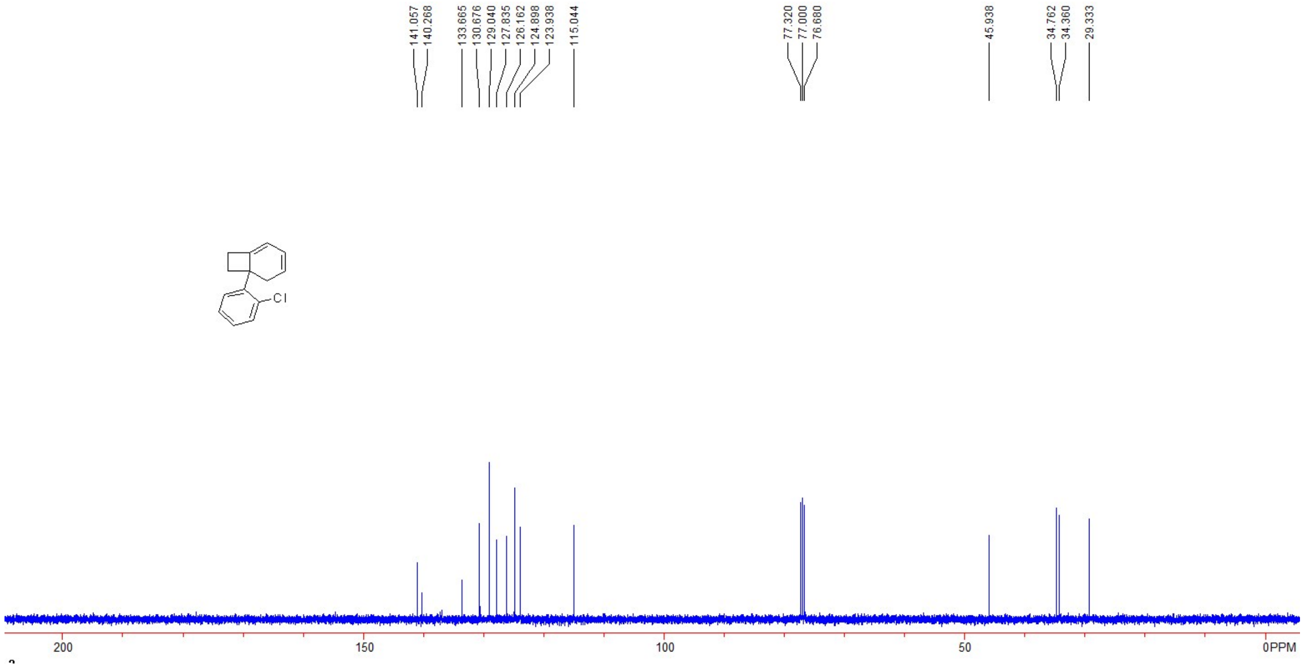

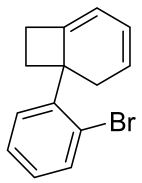

6-(2-bromophenyl)bicyclo[4.2.0]octa-1,3-diene 6m:

45 mg, yield = 87%. Colorless oil. ^{1}H NMR (400 MHz, $CDCl_3$, TMS): δ 2.15-2.21 (m, 1H, CH_2), 2.54-2.57 (m, 2H, CH_2), 2.58-2.67 (m, 2H, CH_2), 2.75-2.80 (m, 2H, CH_2), 5.42-5.45 (m, 1H, =CH), 5.93 (br, 1H, =CH), 5.99-6.02 (m, 1H, =CH), 7.06-7.09 (m, 1H, Ar), 7.10-7.19 (m, 1H, Ar), 7.20-7.23 (m, 1H, Ar), 7.35 (dd, J_1 = 7.6 Hz, J_2 = 1.2 Hz, 1H, Ar), 7.58 (d, J = 7.6 Hz, 1H, Ar). ^{13}C NMR (100 MHz, $CDCl_3$, TMS): δ 29.4, 34.7, 35.0, 47.1, 115.1, 123.4, 124.0, 124.9, 126.8, 128.1, 129.4, 134.2, 141.1, 141.5. IR (CH_2Cl_2) ν 3035, 2949, 2815, 1462, 1422, 1019, 746, 663 cm^{-1}. MS (%) m/z 262 (M^+, 1.31), 245 (7.49), 232 (100.00), 181 (48.46), 165 (91.15), 152 (71.61), 89 (31.44), 76 (27.98), 51 (15.40). HRMS (EI) calcd. for $C_{14}H_{13}Br$: 260.0201, found: 260.0195.

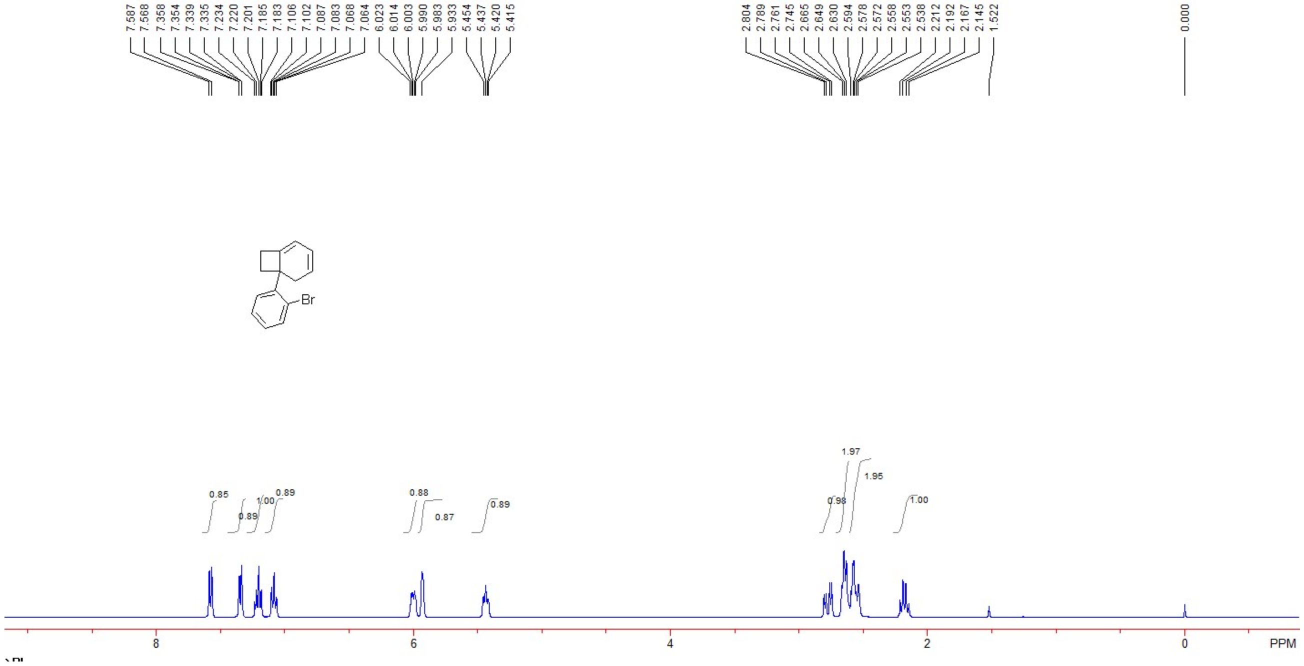

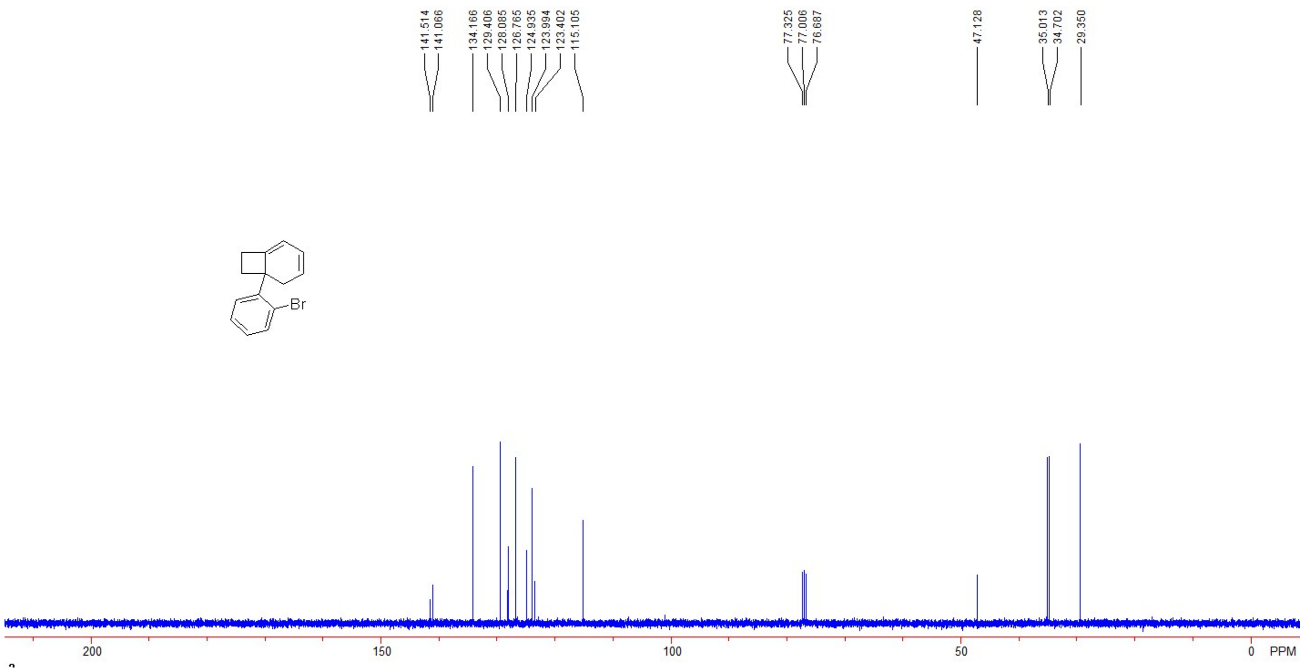

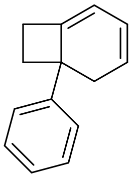

6-phenylbicyclo[4.2.0]octa-1,3-diene 6m':

24 mg, yield = 86%. This is a known compound.[6] ^{1}H NMR (400 MHz, $CDCl_3$, TMS): δ 2.11-2.18 (m, 1H, CH_2), 2.29-2.39 (m, 2H, CH_2), 2.61-2.70 (m, 2H, CH_2), 2.74-2.80 (m, 1H, CH_2), 5.44 (dd, J_1 = 7.6 Hz, J_2 = 7.6 Hz, 1H, =CH), 5.87 (br, 1H, =CH), 6.01 (dd, J_1 = 9.6 Hz, J_2 = 4.8 Hz, 1H, =CH), 7.18-7.20 (m, 1H, Ar), 7.21-7.35 (m, 4H, Ar). ^{13}C NMR (100 MHz, $CDCl_3$, TMS): δ 30.0, 35.8, 37.8, 46.3, 113.9, 122.8, 125.2, 126.1, 126.9, 128.2, 142.9, 145.2.

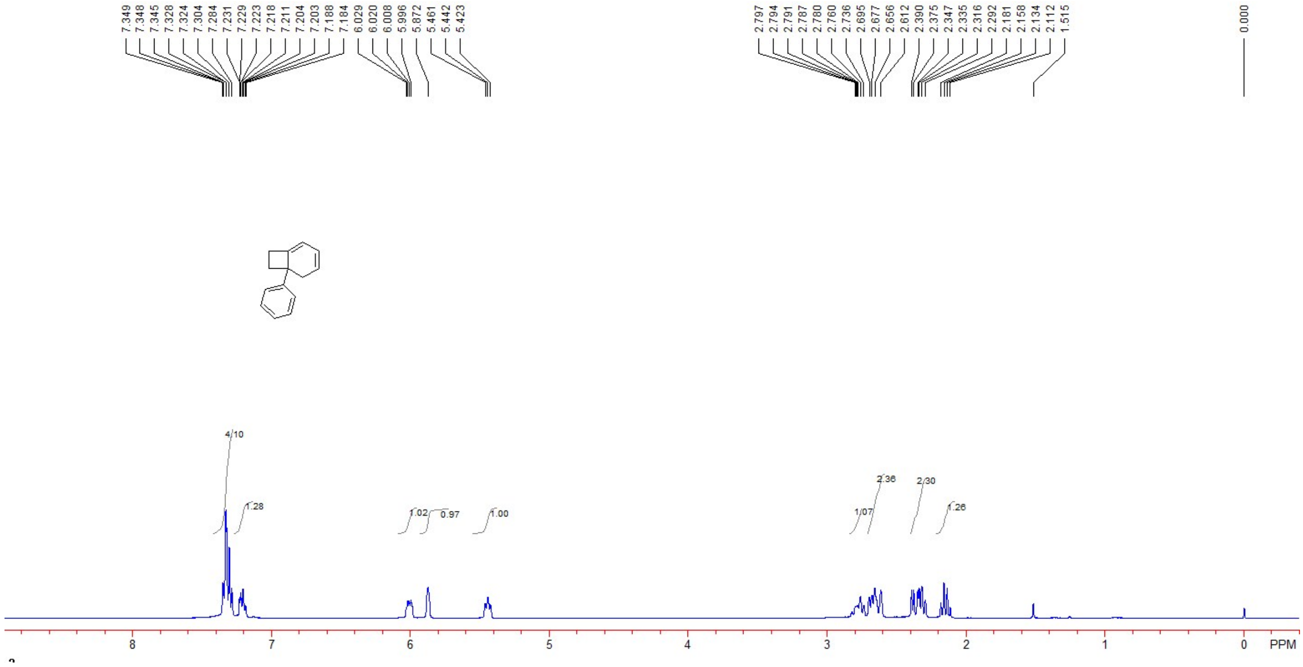

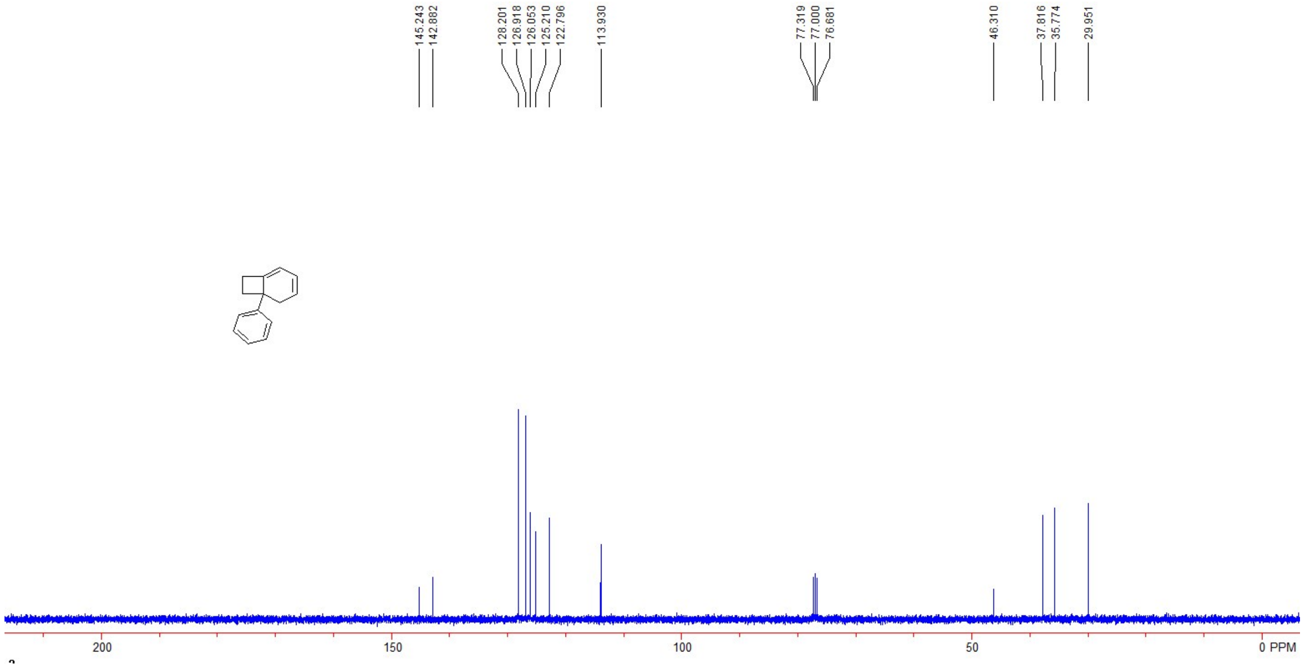

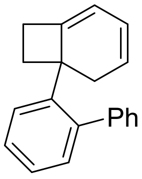

6-(2-Bromophenyl)bicyclo[4.2.0]octa-1,3-diene 6n:

43 mg, yield = 83%. Colorless oil. ^{1}H NMR (400 MHz, $CDCl_3$, TMS): δ 1.65-1.72 (m, 1H, CH_2), 2.15-2.20 (m, 1H, CH_2), 2.35-2.40 (m, 1H, CH_2), 2.49-2.58 (m, 2H, CH_2), 2.61-2.68 (m, 1H, CH_2), 5.43-5.48 (m, 1H, =CH), 5.84 (br, 1H, =CH), 5.99-6.03 (m, 1H, =CH), 7.18-7.22 (m, 1H, Ar), 7.25-

7.28 (m, 2H, Ar), 7.32-7.37 (m, 5H, Ar), 7.46-7.48 (m, 1H, Ar). ^{13}C NMR (100 MHz, $CDCl_3$, TMS): δ 30.0, 35.5, 37.9, 46.1, 114.1, 123.6, 125.1, 126.3, 126.7, 126.9, 127.8, 128.3, 129.5, 132.3, 141.0, 141.5, 143.1, 143.3. IR (CH_2Cl_2) ν 3034, 1474, 1433, 1033, 1009, 745, 700, 672 cm^{-1}. MS (%) m/z 262 (M^+, 1.31), 245 (7.49), 232 (100.00), 181 (48.46), 165 (91.15), 152 (71.61), 89 (31.44), 76 (27.98), 51 (15.40). HRMS (EI) calcd. for $C_{20}H_{18}$: 258.1409, found: 258.1407.

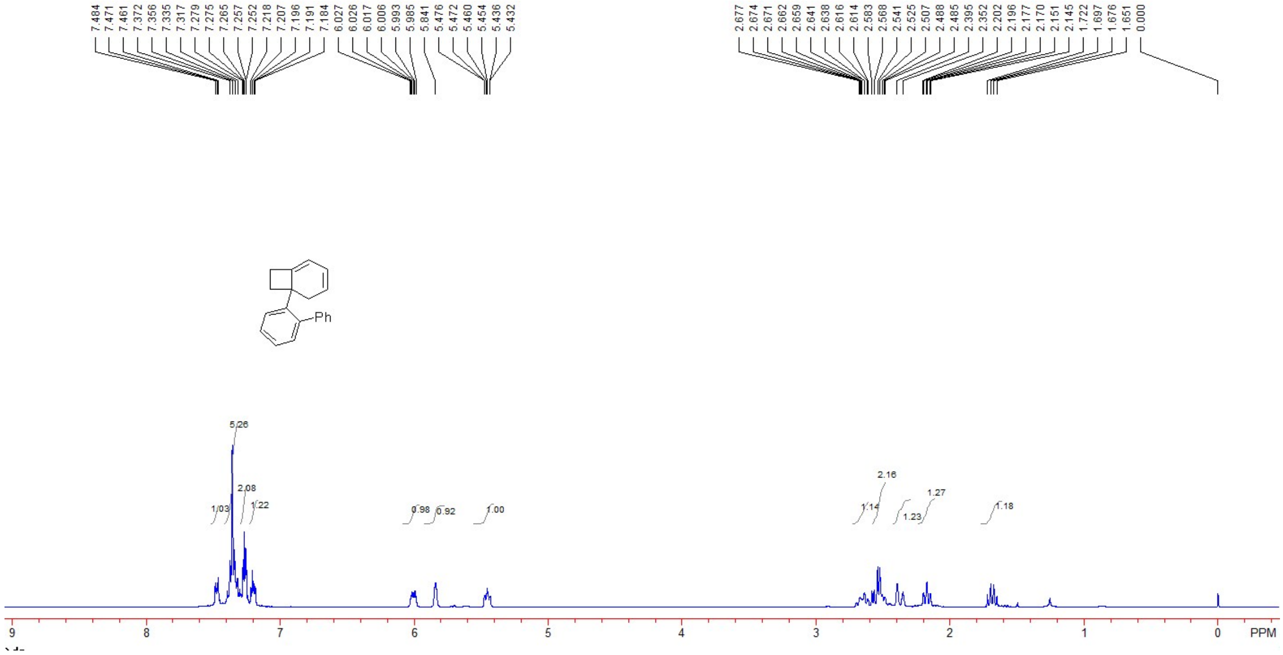

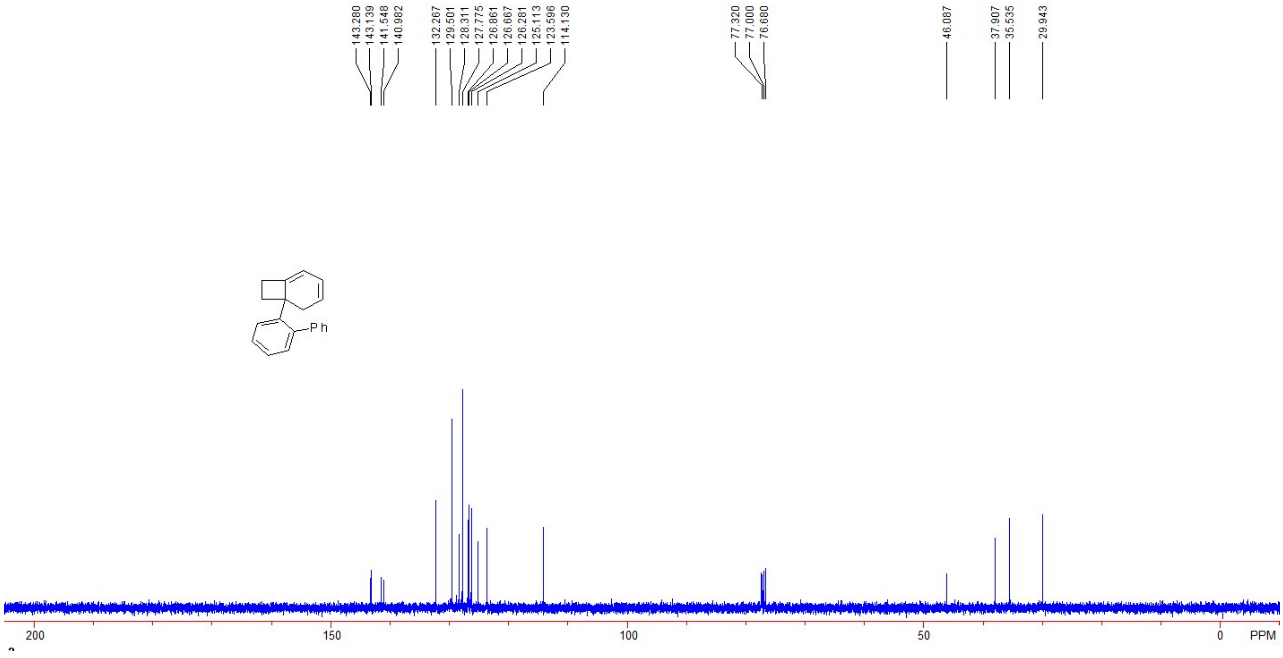

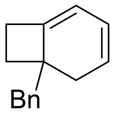

6-Benzylbicyclo[4.2.0]octa-1,3-diene 6o:

33 mg, yield = 83%. Colorless oil. ^{1}H NMR (400 MHz, $CDCl_3$, TMS): δ 1.64-1.71 (m, 1H, CH), 2.15-2.22 (m, 3H, CH_2), 2.47-2.59 (m, 2H, CH_2), 2.67 (d, J = 13.2 Hz, 1H, CH_2), 2.87 (d, J = 13.2 Hz, 1H, CH_2), 5.55-5.63 (m, 1H, =CH), 5.62-5.63 (m, 1H, =CH), 5.90-5.93 (m, 1H, =CH), 7.19-7.22 (m, 3H, Ar), 7.25-7.28 (m, 2H, Ar). ^{13}C NMR (100 MHz, $CDCl_3$, TMS): δ 29.5, 31.3, 33.0, 37.8, 43.2, 112.7, 123.2, 124.7, 125.8, 127.8, 130.0, 139.1, 145.3. IR (CH_2Cl_2) ν 3028, 2918, 2817, 1671, 1494, 1435, 742, 700, 676 cm^{-1}. MS (%) m/z 196 (M^+, 3.53), 167 (26.67), 152 (4.63), 115 (8.81), 105 (100.00), 91 (84.62), 77 (34.72), 65 (20.02), 51 (11.33). HRMS (EI) calcd. for $C_{15}H_{16}$: 196.1252, found: 196.1257.

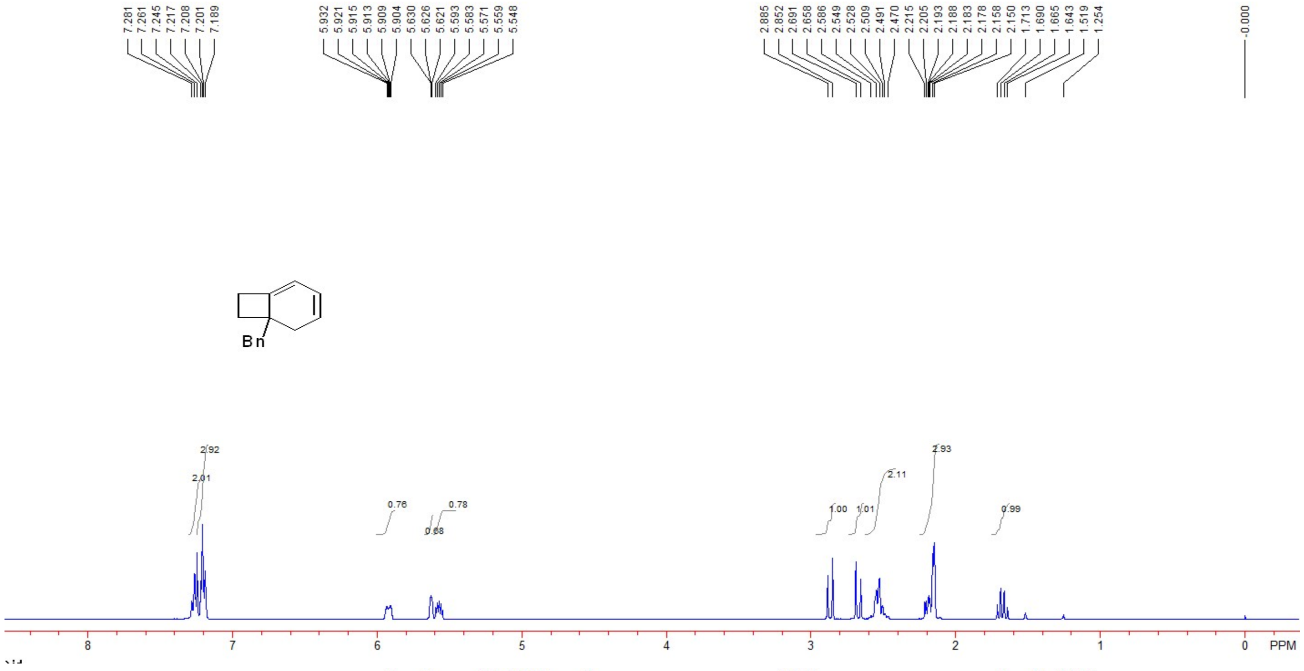

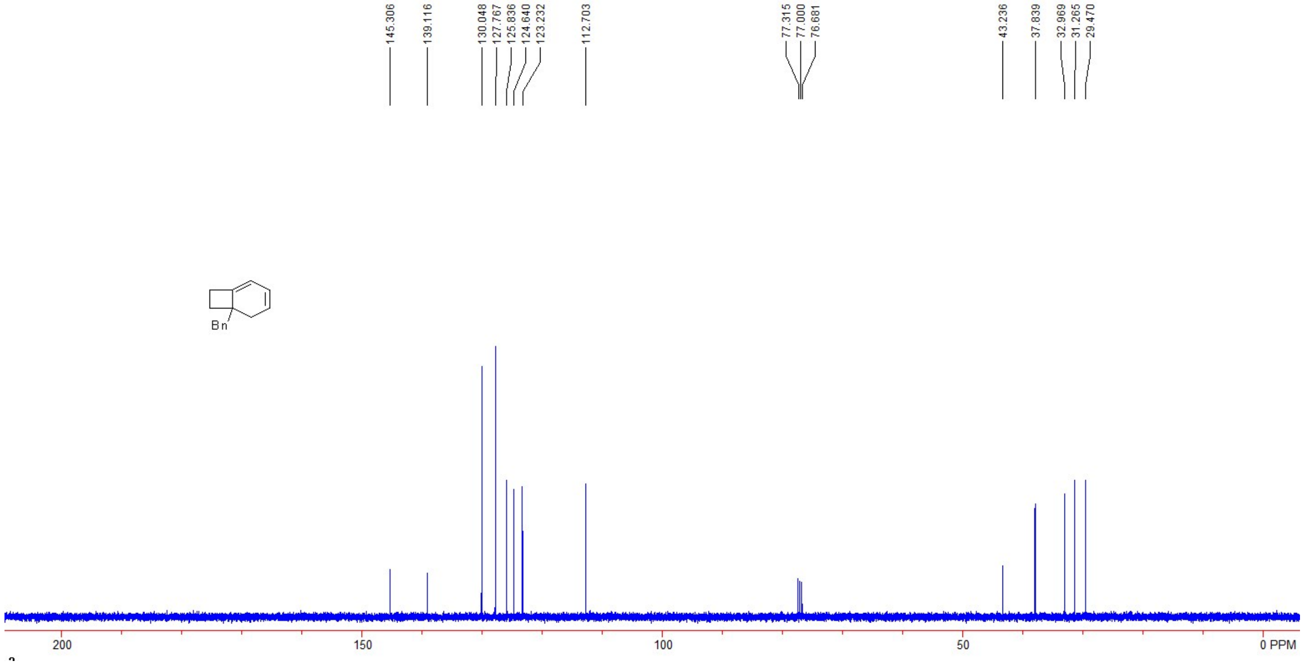

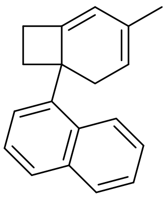

1-(4-Methylbicyclo[4.2.0]octa-3,5-dien-1-yl)naphthalene 6p:

45 mg, yield = 91%. Colorless oil. ^{1}H NMR (400 MHz, $CDCl_3$, TMS): δ 1.53 (s, 3H, CH_3), 2.33-2.40 (m, 1H, CH_2), 2.50-2.55 (m, 1H, CH_2), 2.68-2.79 (m, 4H, CH_2), 5.06-5.07 (m, 1H, =CH), 5.86 (br, 1H, =CH), 7.32-7.35 (m, 1H, Ar), 7.41-7.47 (m, 3H, Ar), 7.70 (d, J = 8.0 Hz, 2H, Ar), 7.85-7.87 (m, 2H, Ar). ^{13}C NMR (100 MHz, $CDCl_3$, TMS): δ 21.3, 29.8, 35.7, 37.5, 46.8, 118.1, 118.3, 124.2, 124.96, 124.99, 125.10, 125.15, 127.24, 129.4, 130.1, 132.3, 135.0, 139.0, 142.3. IR (CH_2Cl_2) ν 2928, 2855, 2813, 1680, 1508, 1252, 851, 775, 732 cm^{-1}. MS (%) m/z 246 (M^+, 54.75), 231 (67.33), 218 (100.00), 203 (54.25), 189 (25.62), 165 (16.43), 141 (56.29), 115 (14.89), 101 (16.02), 108 (20.68). HRMS (EI) calcd. for $C_{19}H_{18}$: 246.1409, found: 246.1411.

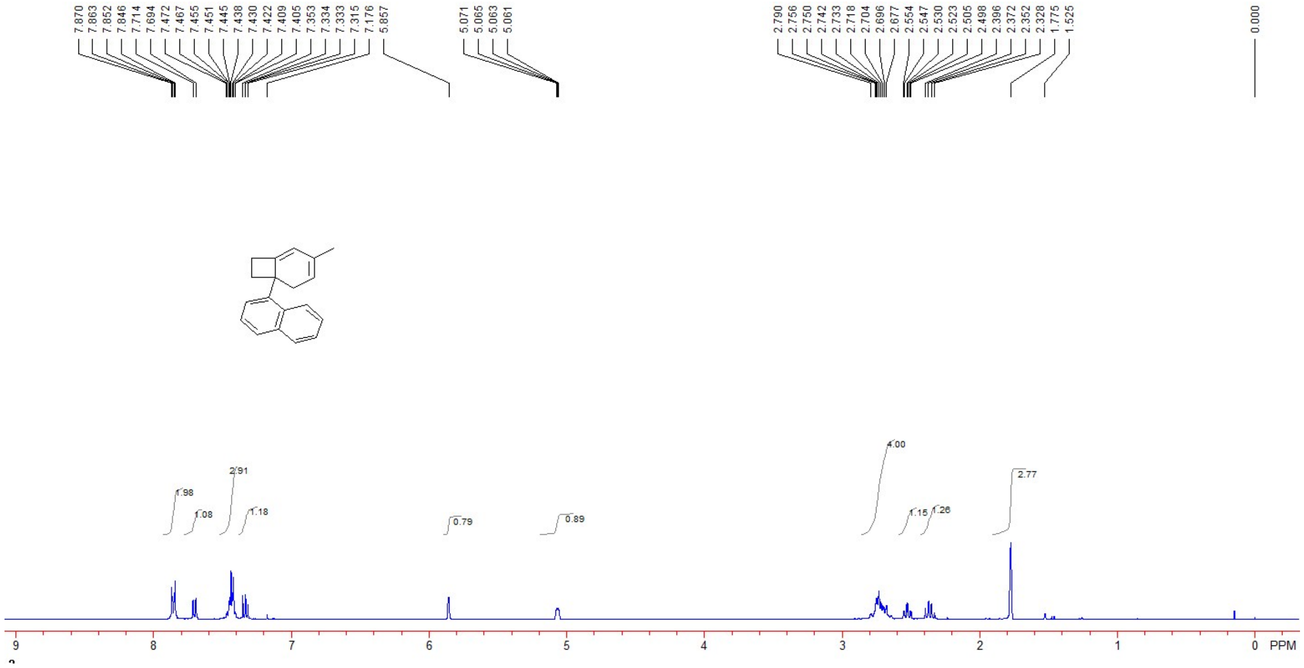

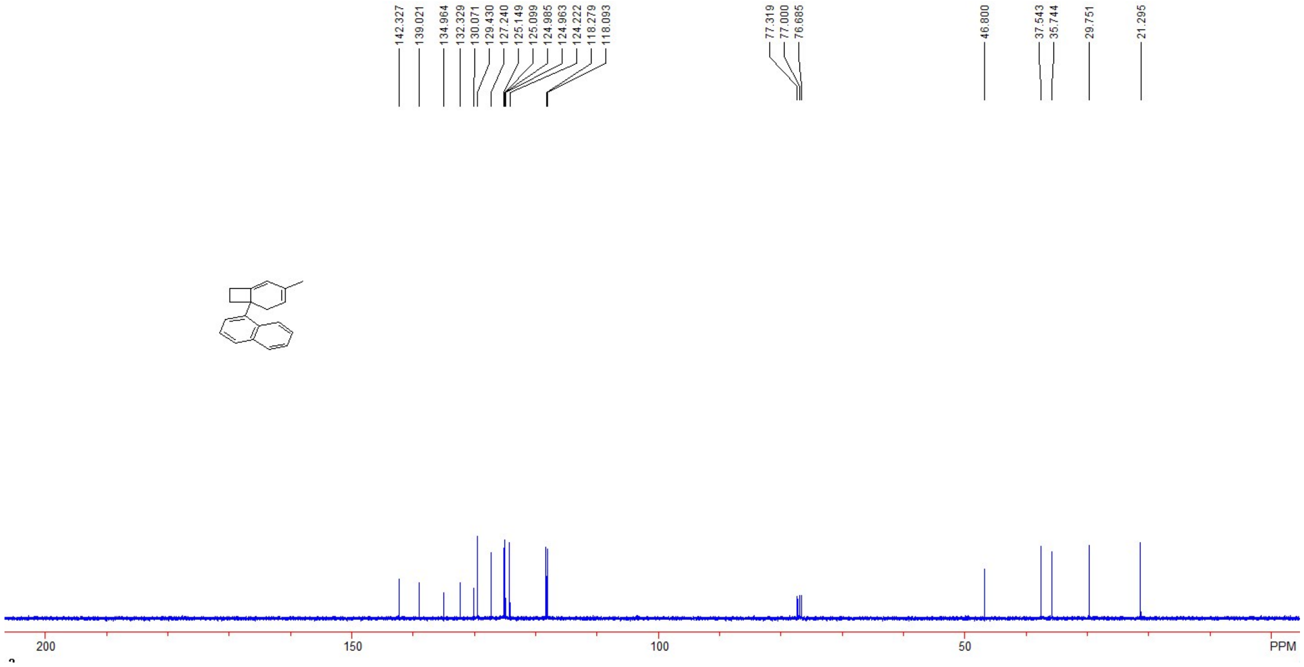

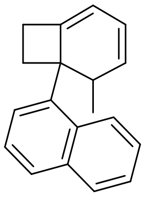

1-(2-Methylbicyclo[4.2.0]octa-3,5-dien-1-yl)naphthalene 6q:

44 mg, yield = 90%. Colorless oil (Mixtures of two diastereomers, dr = 6.4/1). ^{1}H NMR (400 MHz, $CDCl_3$, TMS): δ 1.27 (d, J = 7.2 Hz, 3H, CH_3), 2.19-2.25 (m, 1H), 2.53-2.74 (m, 3H), 2.79-2.85 (m, 1H), 5.35-5.39 (m, 1H, =CH), 5.84-5.87 (m, 1H, =CH), 5.90-5.92 (m, 1H, =CH), 7.33-7.37 (m, 1H, Ar), 7.41-7.47 (m, 2H, Ar), 7.54-7.56 (m, 1H, Ar), 7.71-7.76 (m, 2H, Ar), 7.87-7.89 (m, 1H, Ar). ^{13}C NMR (100 MHz, $CDCl_3$, TMS): δ 15.8, 27.4, 28.4, 36.1, 51.4, 113.8, 122.3, 123.7, 124.8, 124.9, 125.1, 125.2, 127.2, 128.6, 130.1, 130.3, 135.2, 140.0, 140.5. IR (CH_2Cl_2) ν 3030, 2955, 2858, 1594, 1393, 1061, 907, 842, 800, 719 cm^{-1}. MS (%) m/z 246 (M^+, 23.68), 231 (60.87), 218 (100.00), 203 (49.09), 189 (14.88), 165 (15.57), 141 (71.77), 101 (15.54), 108 (20.90). HRMS (EI) calcd. for $C_{19}H_{18}$: 246.1409, found: 246.1405.

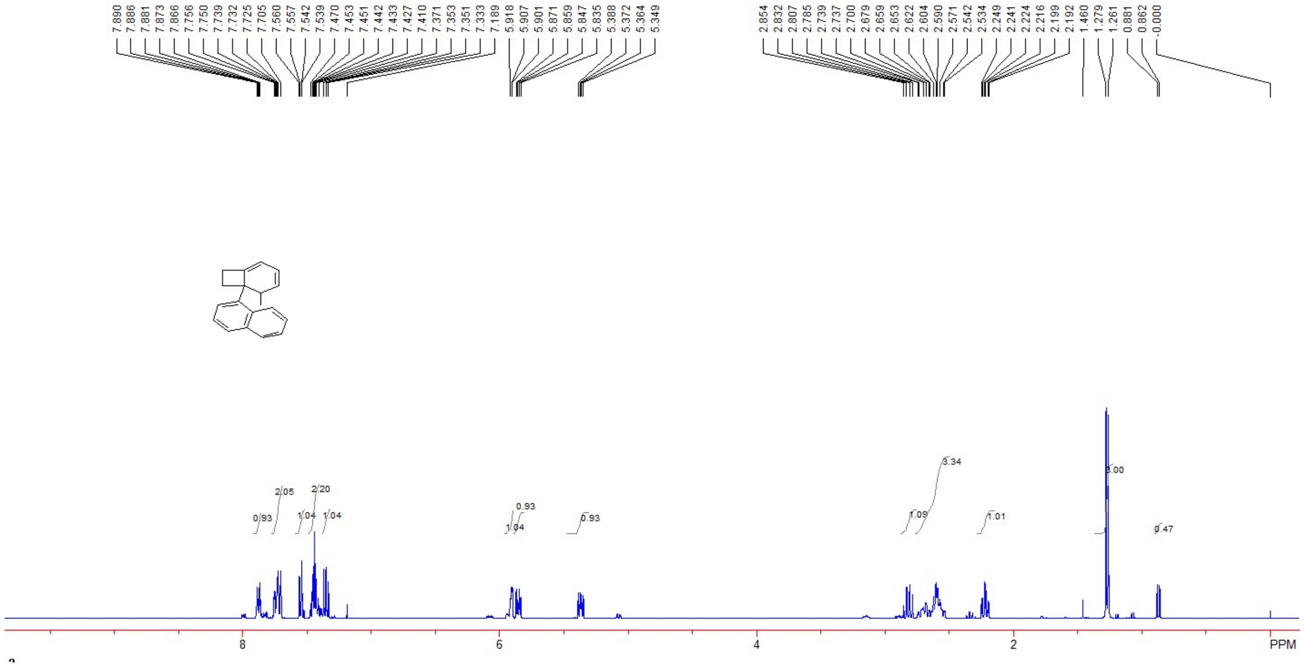
7.890 7.886 7.881 7.873 7.866 7.756 7.750 7.739 7.732 7.725 7.705 7.560 7.557 7.542 7.539 7.470 7.453 7.451 7.442 7.433 7.427 7.410 7.371 7.353 7.351 7.333 7.189 5.918 5.907 5.901 5.871 5.859 5.847 5.835 5.388 5.372 5.364 5.349
2.854 2.832 2.807 2.785 2.739 2.737 2.700 2.679 2.659 2.653 2.622 2.604 2.590 2.571 2.542 2.534 2.249 2.241 2.224 2.216 2.199 2.192 1.460 1.279 1.261 0.881 0.862 -0.000
0.93 2.05 1.04 2.20 1.04 0.93 1.04 0.93 1.09 3.34 1.01 3.00 0.47
8 6 4 2 PPM

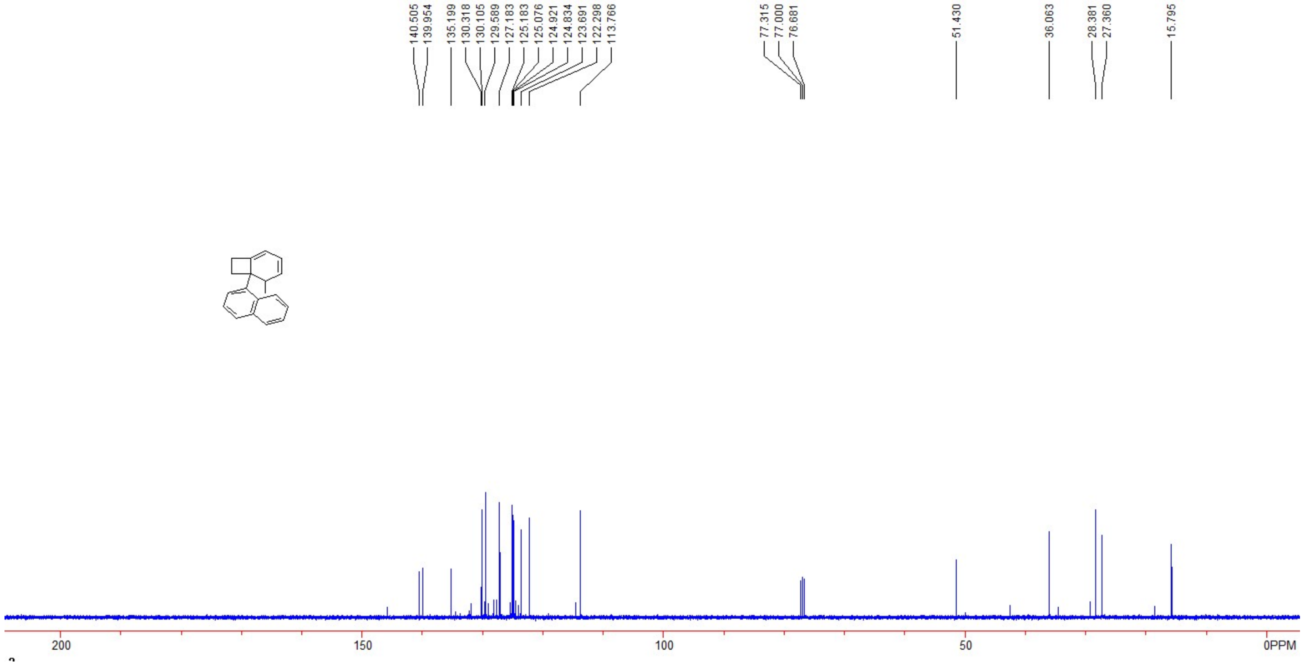
140.505 139.954 135.199 130.318 130.105 129.589 127.183 125.183 125.076 124.921 124.834 123.691 122.298 113.766 77.315 77.000 76.681 51.430 36.063 28.381 27.360 15.795
200 150 100 50 0PPM

6. Evidences for the Existence of Rotamer.

6.1 HPLC resolution of compound **6a**.

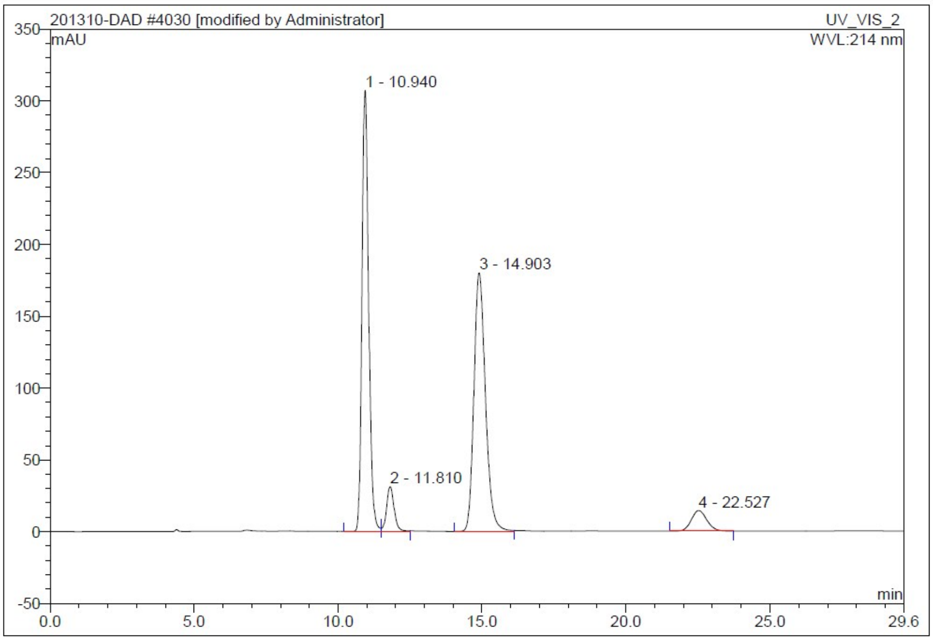

No.	Ret.Time min	Peak Name	Height mAU	Area mAU*min	Rel.Area %	Amount	Type
1	10.94	n.a.	307.251	82.781	44.77	n.a.	BM *
2	11.81	n.a.	31.293	9.552	5.17	n.a.	M *
3	14.90	n.a.	179.996	83.638	45.23	n.a.	BM *
4	22.53	n.a.	14.326	8.941	4.84	n.a.	MB*
Total:			532.865	184.911	100.00	0.000	

6.2 ^{1}H NMR of compound **6m** at rt and at 50 ^{o}C.

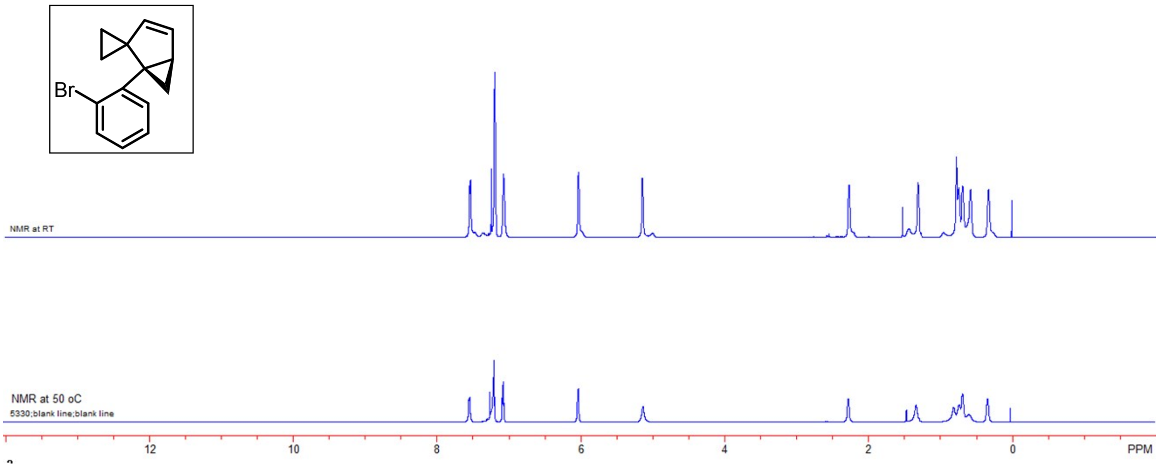

7. ^{1}H and ^{13}C NMR Spectra of the Compound 7j.

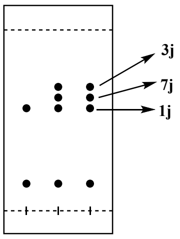

Figure SI-1: TLC Detection of compound **7j**.

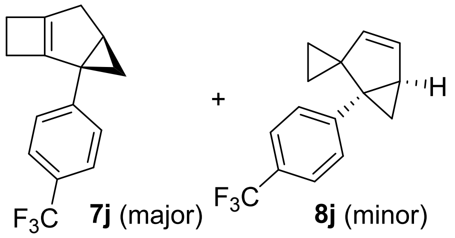

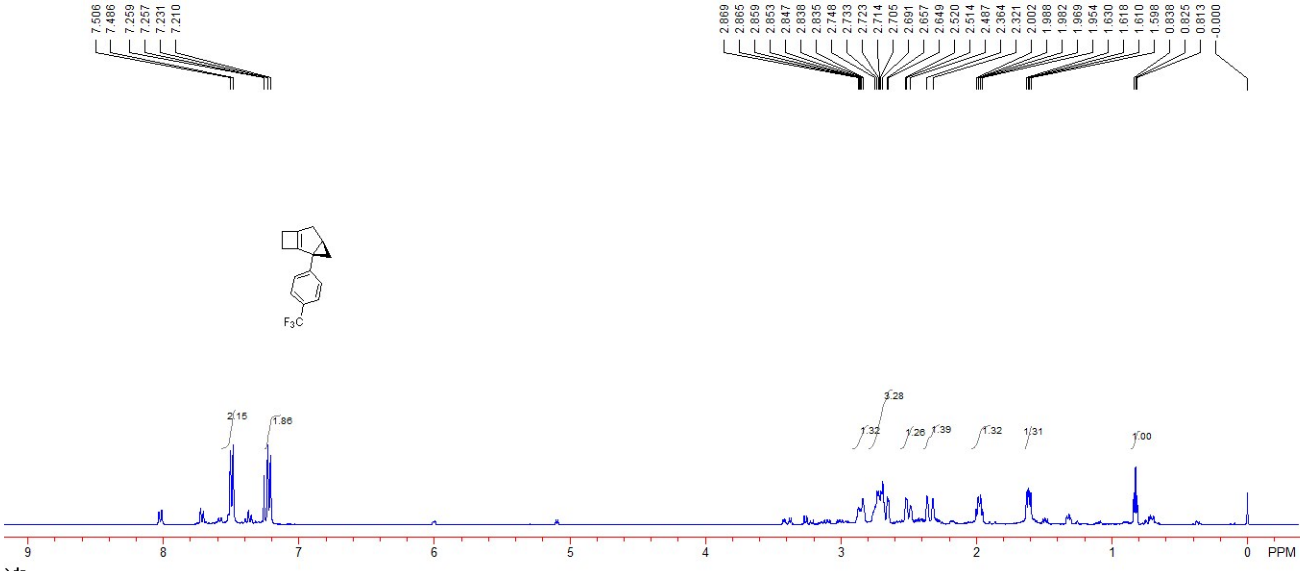

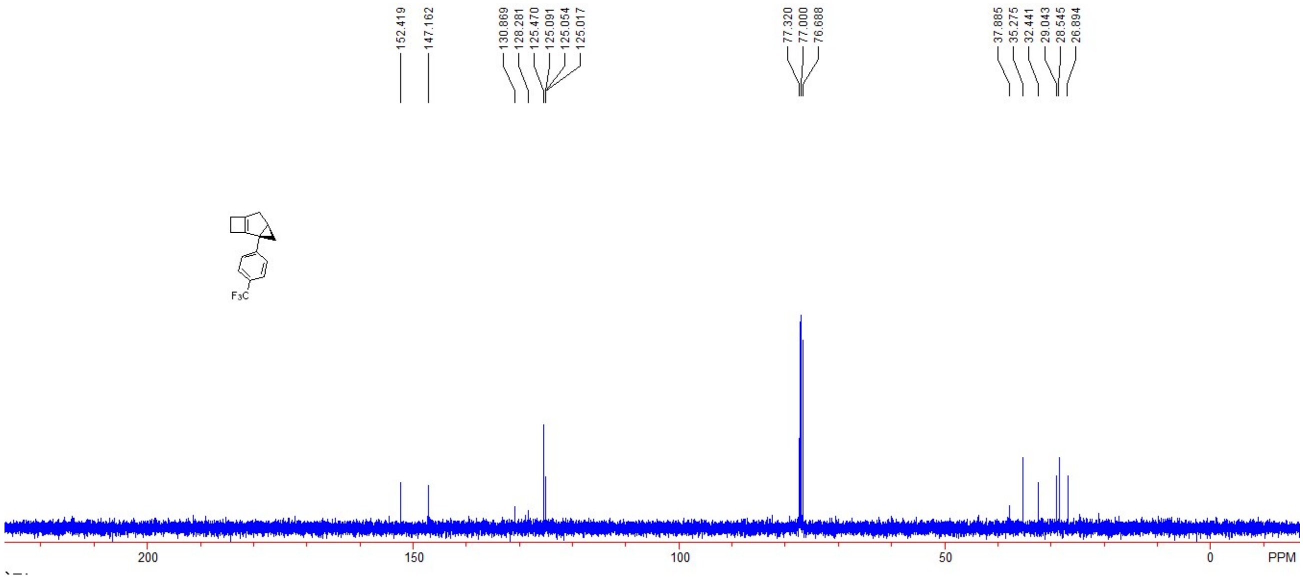

8. ^{1}H NMR Spectra of the Deuterated Compounds.

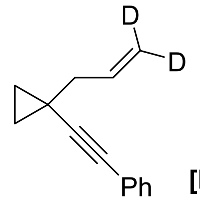

[D$_2$]-1a

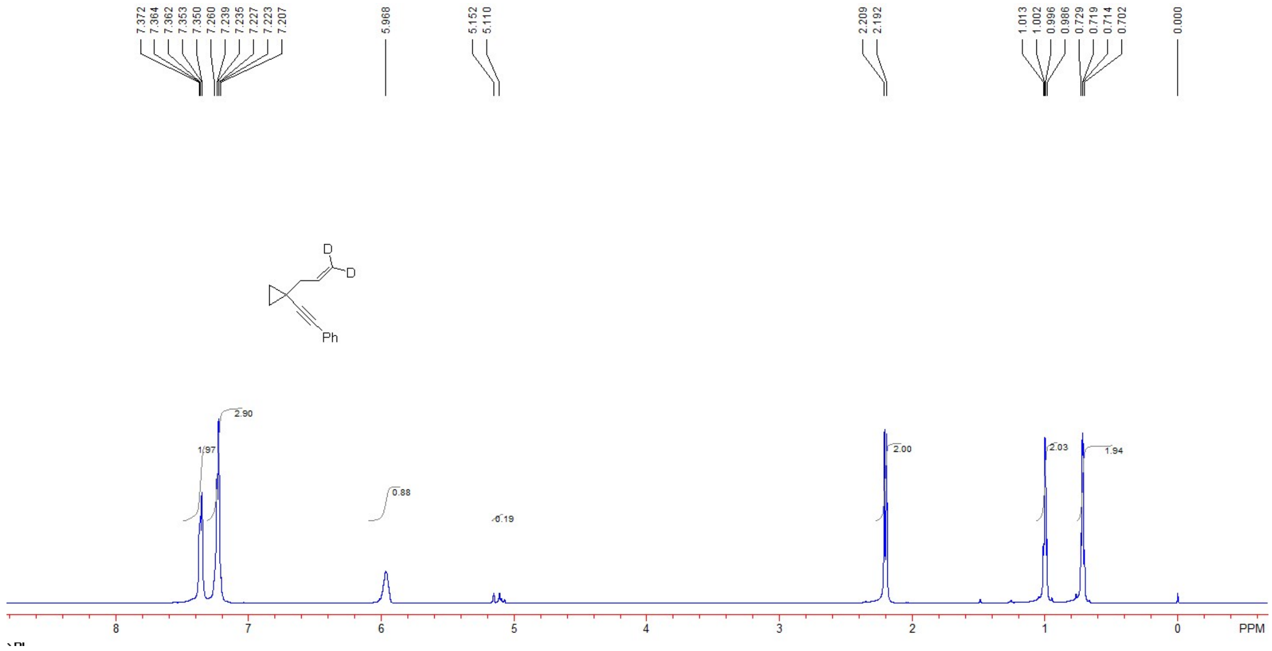

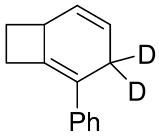

[D$_2$]-3a

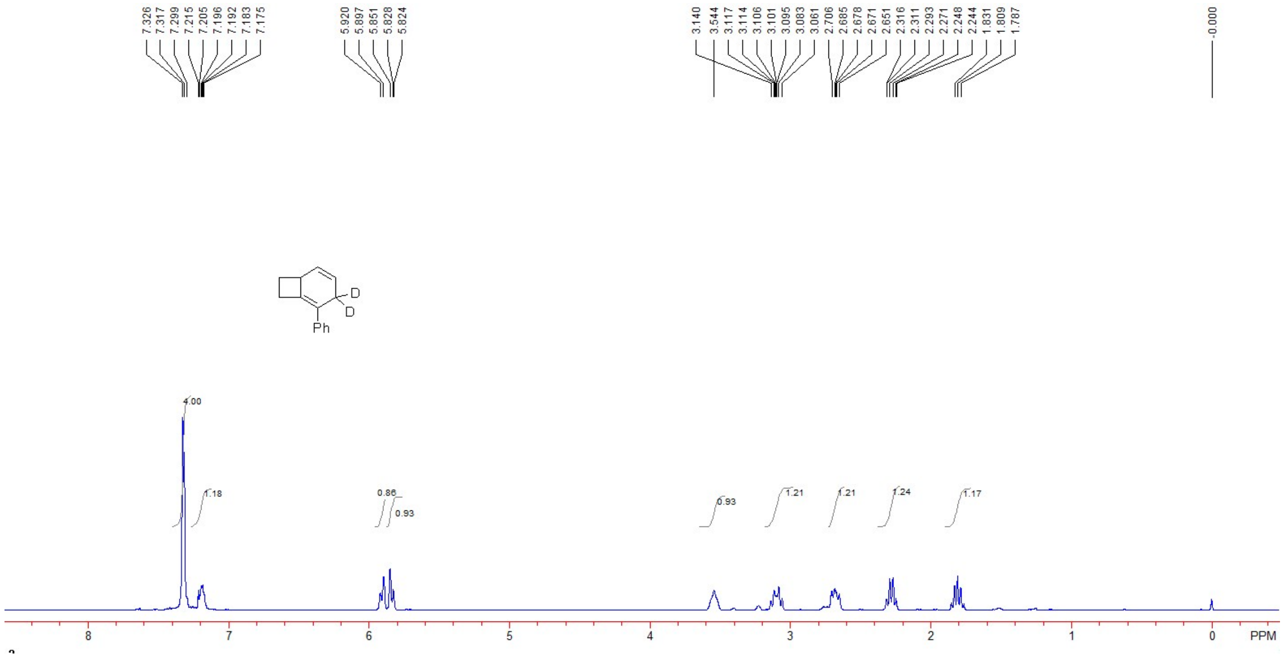

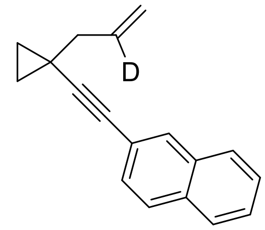

[D$_1$]-1n

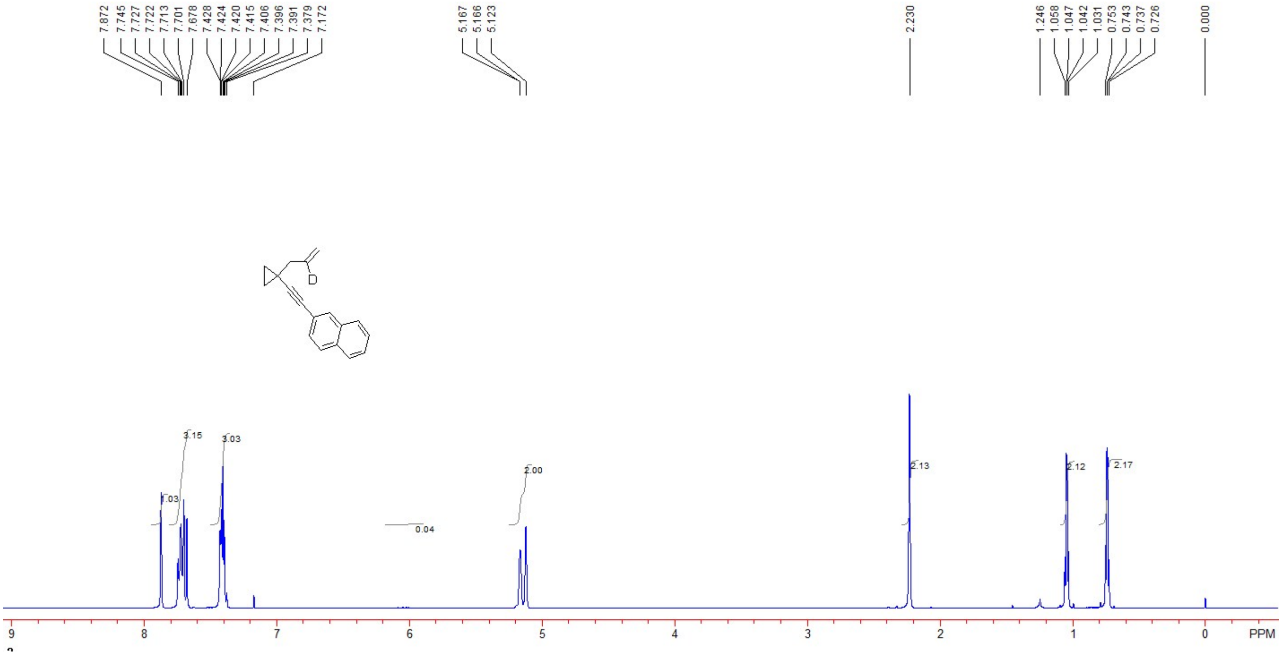

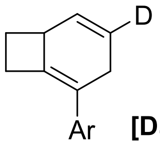

[D$_1$]-3n: Ar = 2-naphth

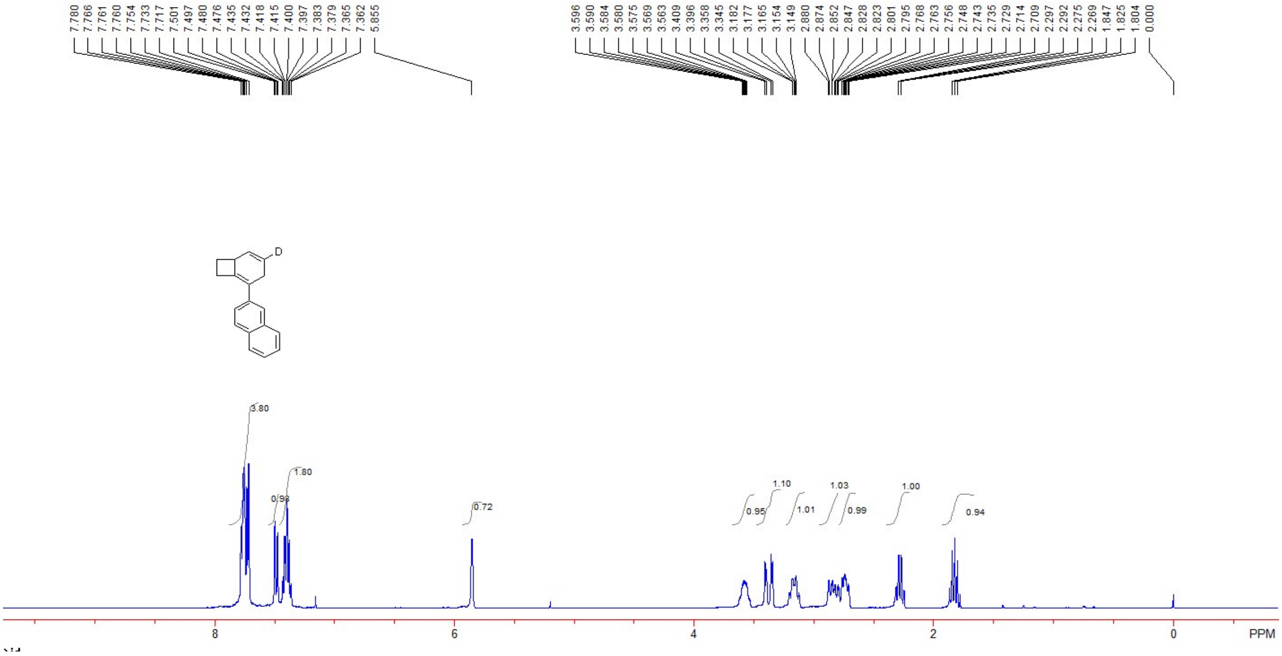

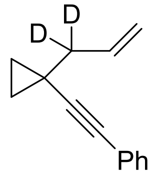
D
D
Ph

[D$_2$]-1a'

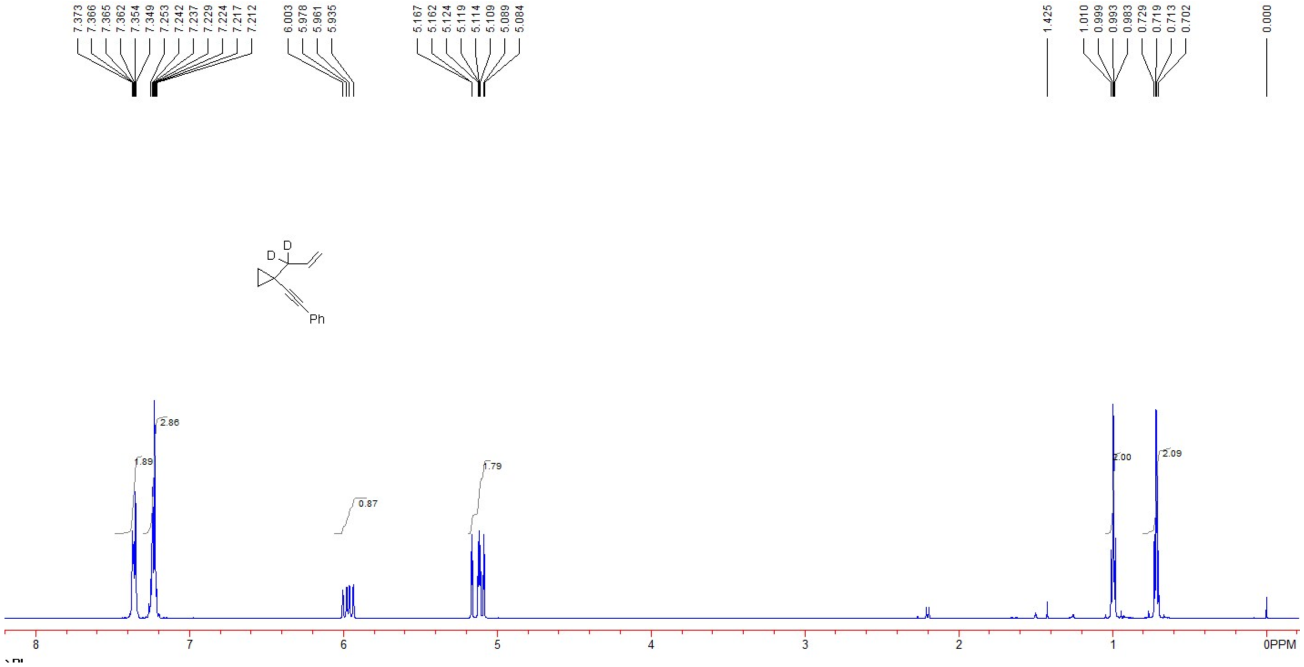
7.373
7.366
7.365
7.362
7.354
7.349
7.253
7.242
7.237
7.229
7.224
7.217
7.212
6.003
5.978
5.961
5.935
5.167
5.162
5.124
5.119
5.114
5.109
5.089
5.084
1.425
1.010
0.999
0.993
0.983
0.729
0.719
0.713
0.702
0.000
D
D
Ph
1.89
2.86
0.87
1.79
2.00
2.09
8
7
6
5
4
3
2
1
0PPM

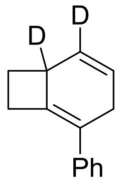
D
D
Ph

[D$_2$]-3a'

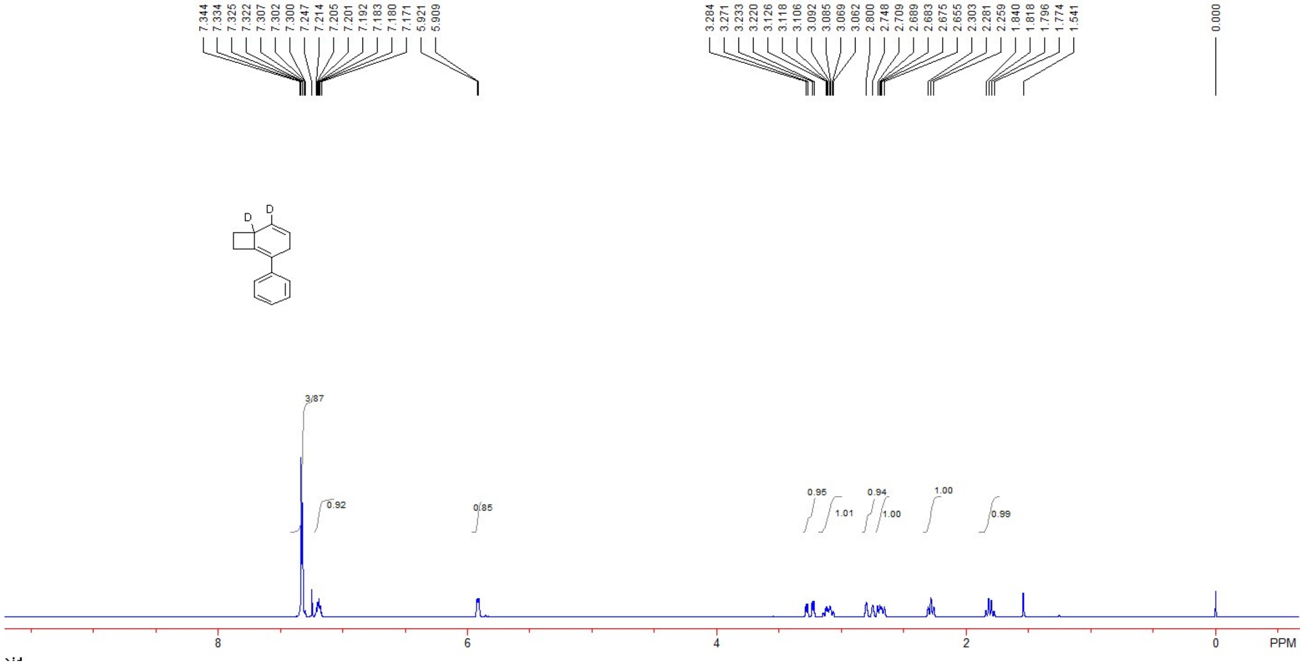
7.344
7.334
7.325
7.322
7.307
7.302
7.300
7.247
7.214
7.205
7.201
7.192
7.183
7.180
7.171
5.921
5.909
3.284
3.271
3.233
3.220
3.126
3.118
3.106
3.092
3.085
3.069
3.062
2.800
2.748
2.709
2.689
2.683
2.675
2.655
2.303
2.281
2.259
1.840
1.818
1.796
1.774
1.541
0.000
D
D
3.87
0.92
0.85
0.95
1.01
0.94
1.00
1.00
0.99
8
6
4
2
0
PPM

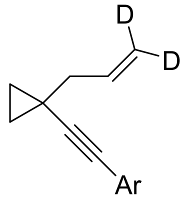

[D$_2$]-2b: Ar = 1-naphth

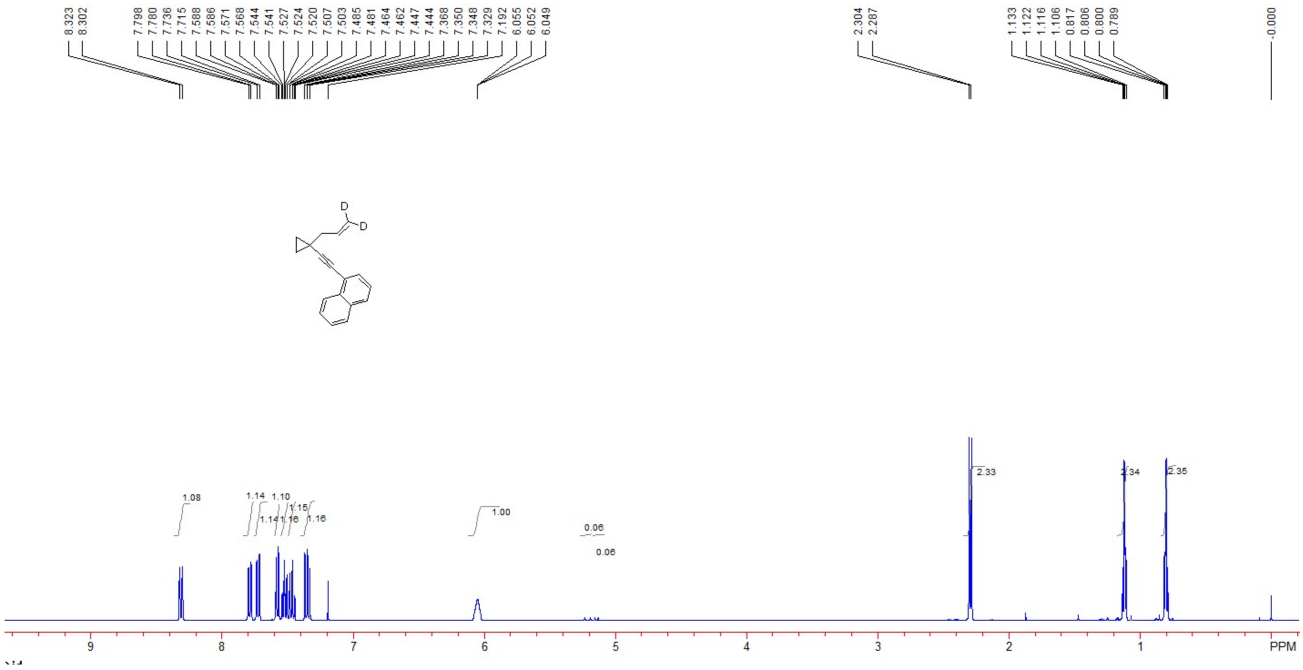

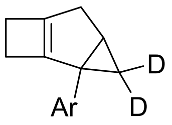

[D$_2$]-4b: Ar = 1-naphth

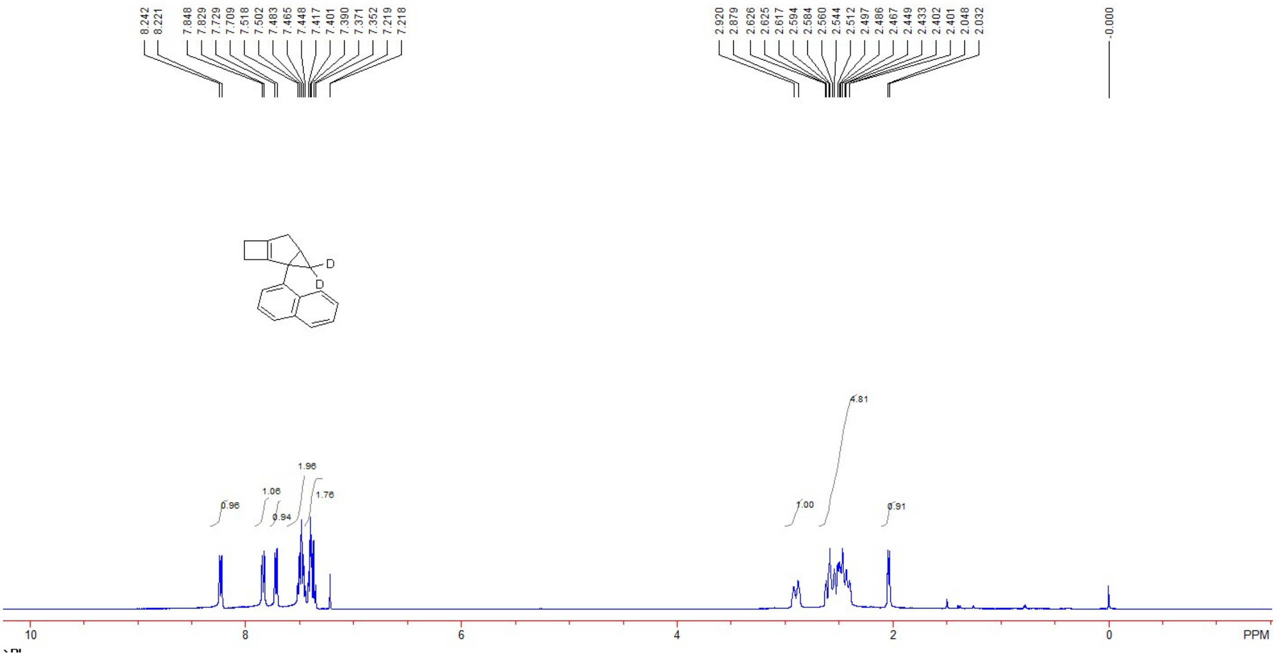

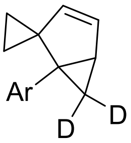

[D$_2$]-5b: Ar = 1-naphth

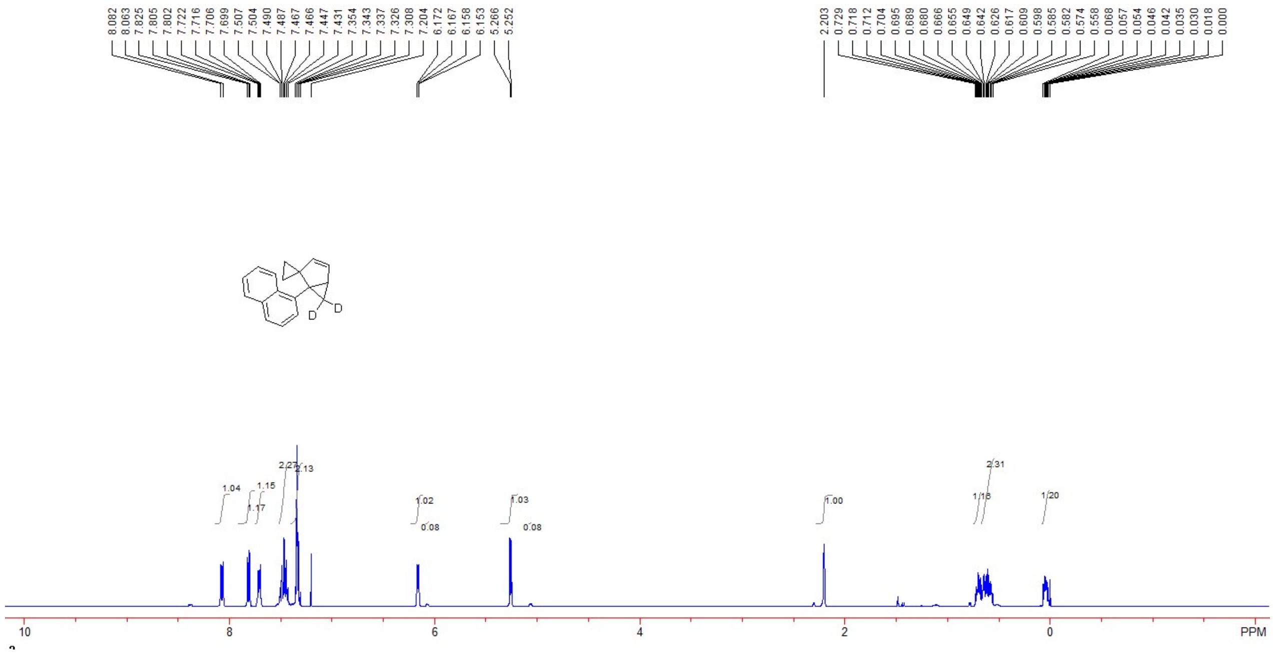

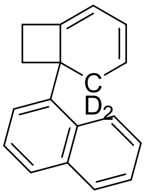

[D$_2$]-6b

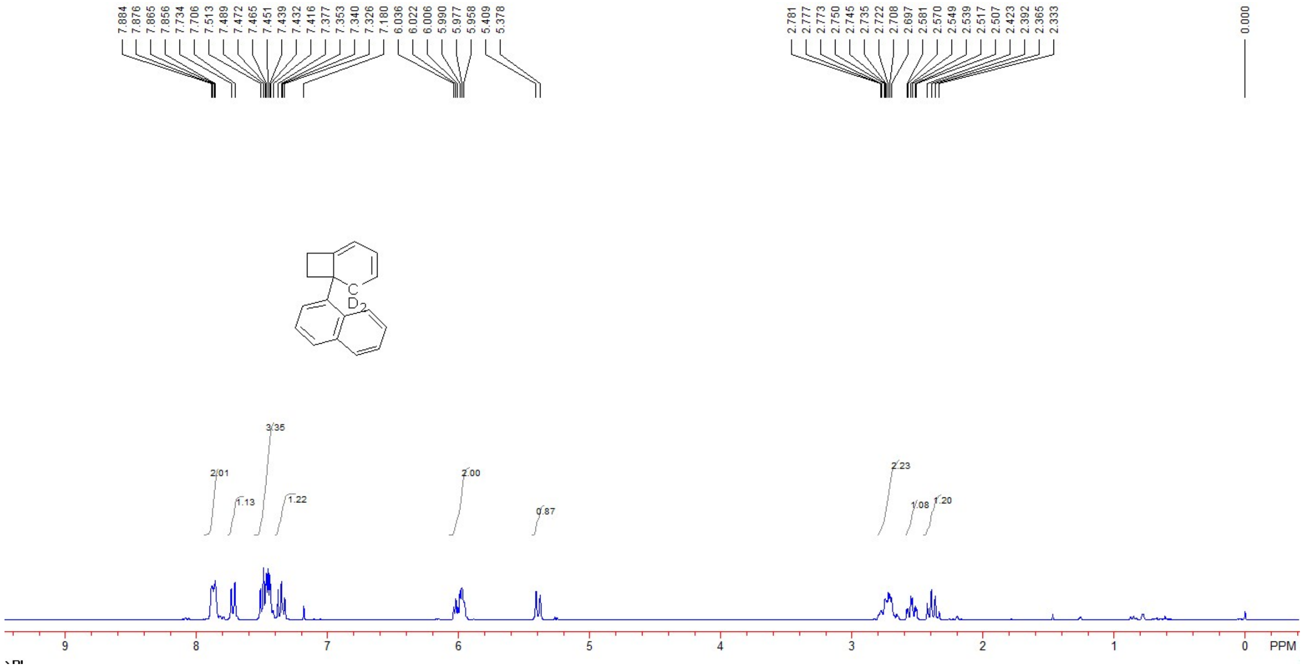

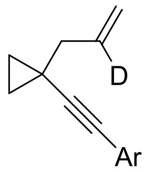

[D$_1$]-2b: Ar = 1-naphth

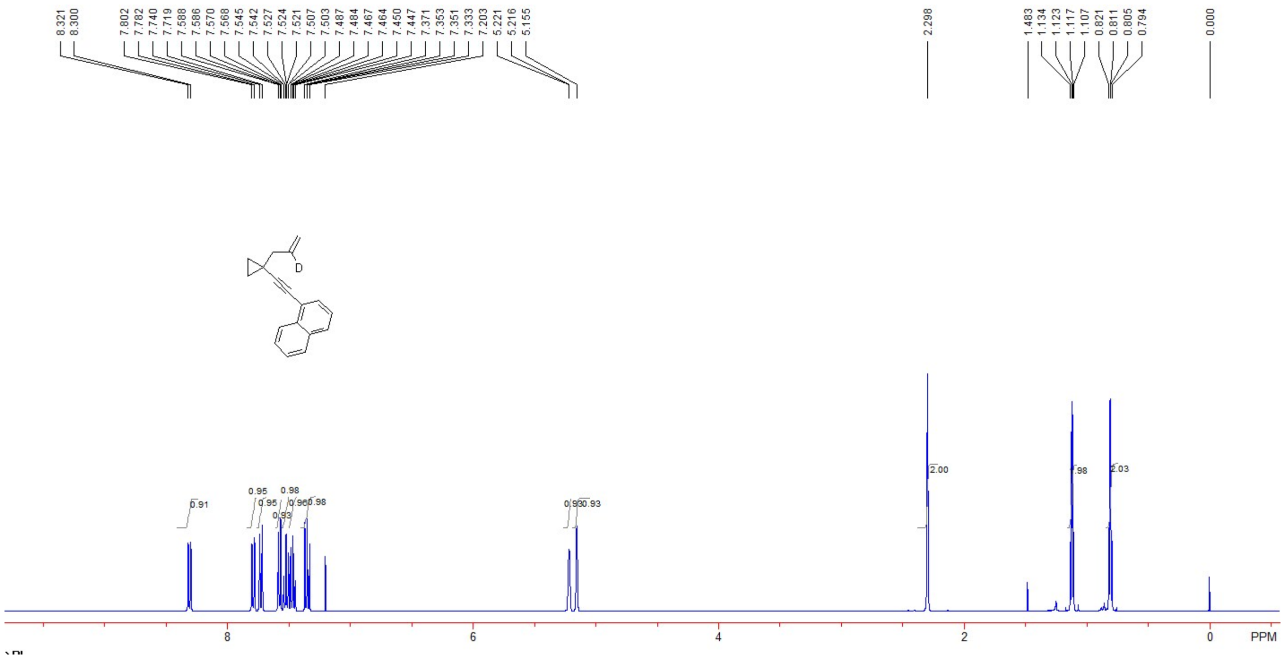

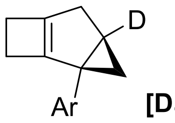

[D$_1$]-4b: Ar = 1-naphth

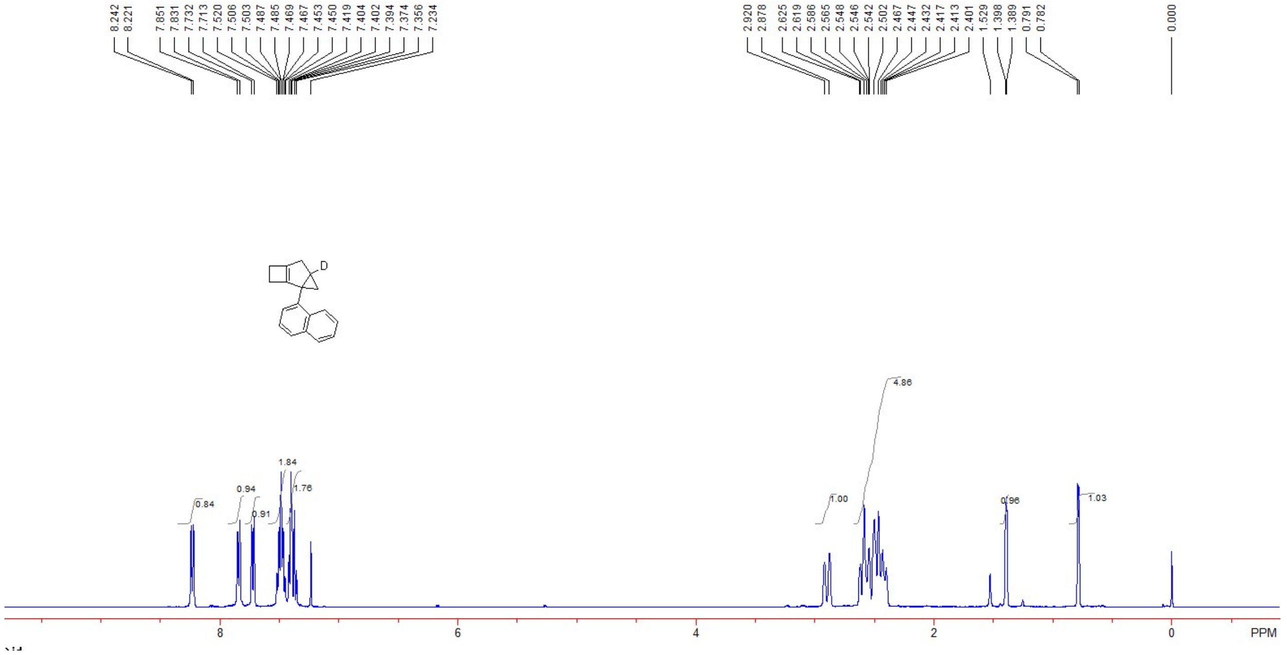

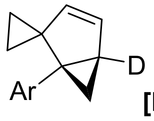

[D$_1$]-5b: Ar = 1-naphth

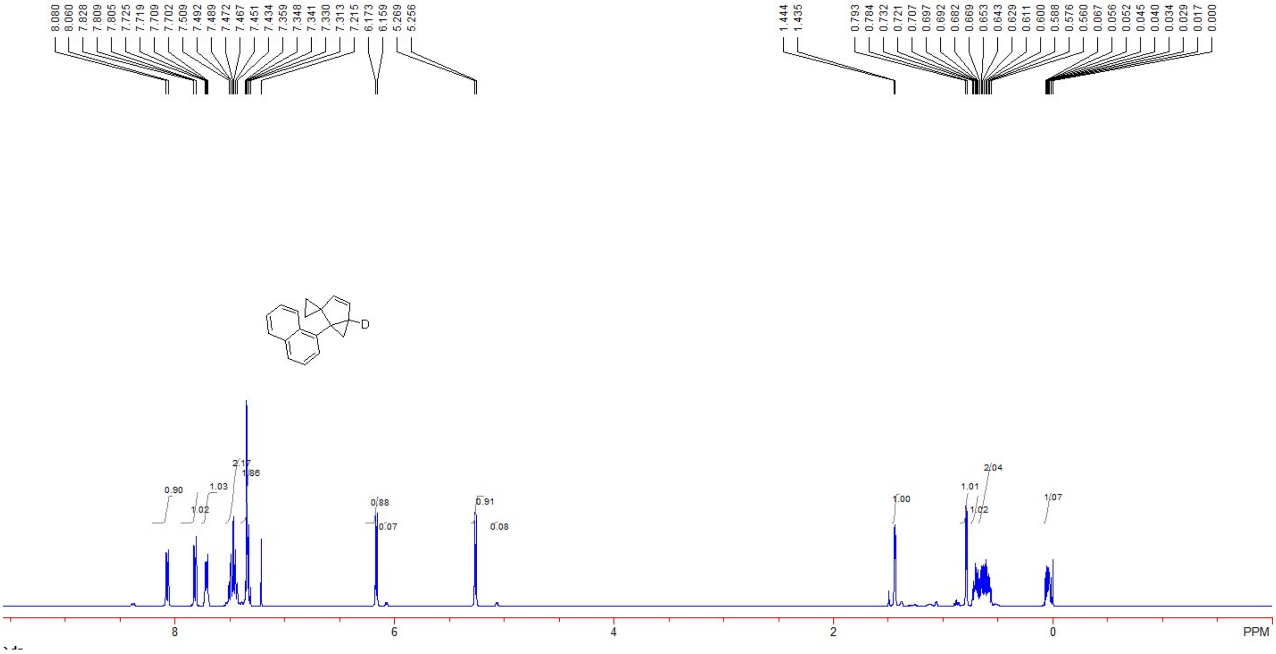

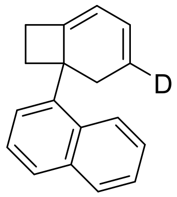

[D$_1$]-6b

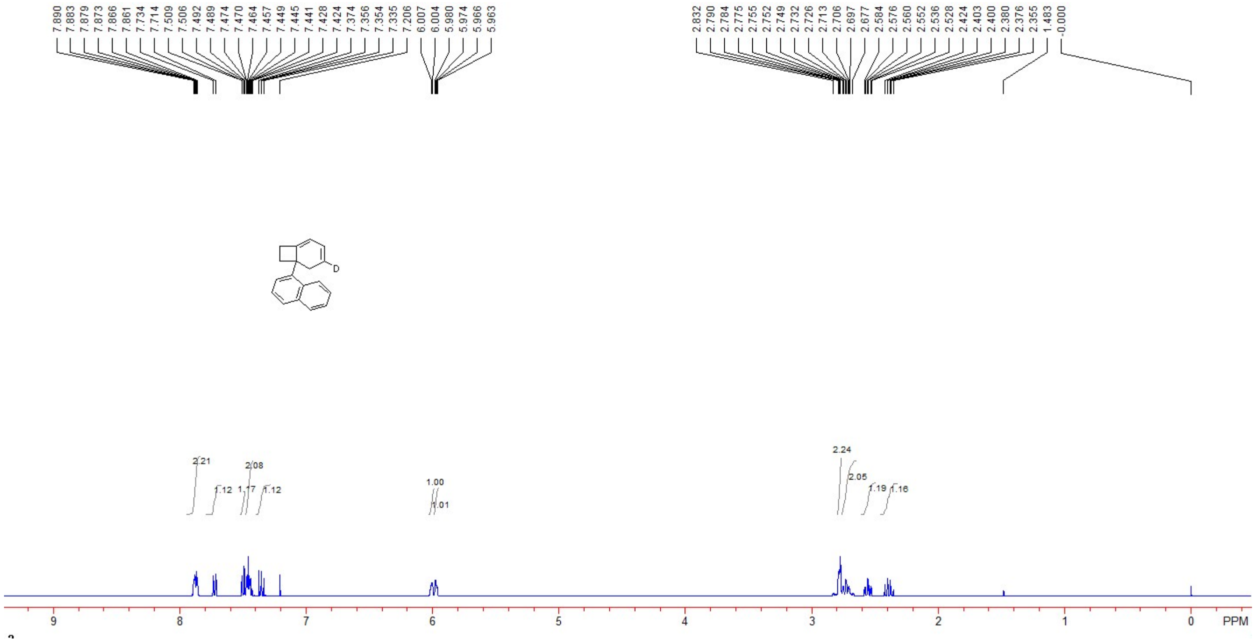

9. Crystallographic Information.

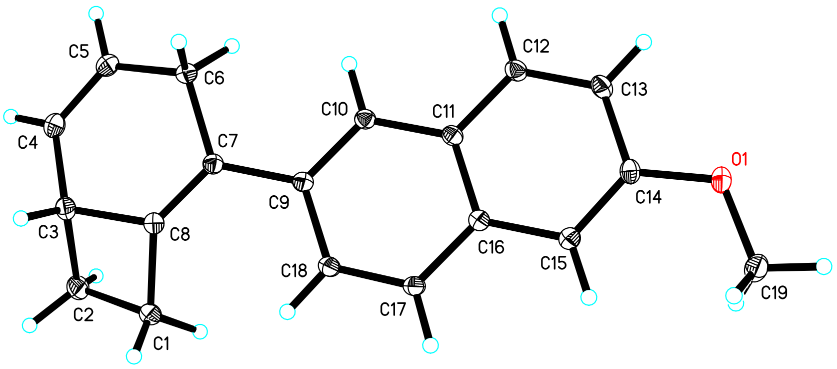

The crystal data of **3o** have been deposited in CCDC with number 1048653. Empirical formula: $C_{19}H_{18}O$, Formula weight: 262.33, Crystal system: Monoclinic, Space group: P 1 21/n 1, Unit cell dimensions: a = 9.9373(12) Å, α = 90°; b = 5.8477(7) Å, β = 98.064(2)°; c = 23.768(3) Å, γ = 90°. Volume: 1367.5(3) Å^3, Z = 4, Density (calculated): 1.274 Mg/m^3, F(000) = 560, Crystal size: 0.25 x 0.22 x 0.2 mm^3, Final R indices [I>2sigma(I)]: R1 = 0.0498, wR2 = 0.1357.

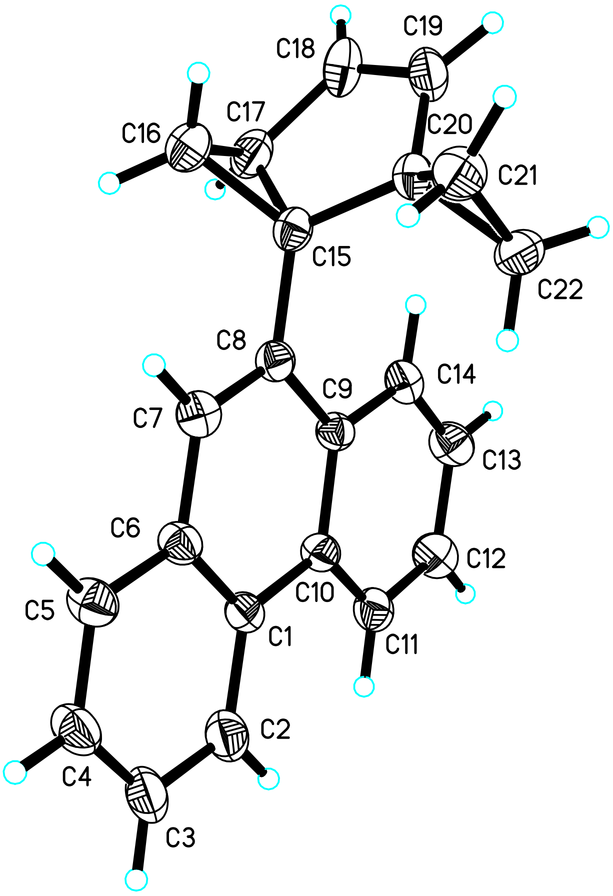

The crystal data of **5a** have been deposited in CCDC with number 1033827. Empirical formula: $C_{22}H_{18}$, Formula weight: 282.36, Crystal system: Orthorhombic, Space group: P na21, Unit cell dimensions: a = 22.283(3) Å, α = 90°; b = 10.1485(15) Å, β = 90°; c = 6.7864(11) Å, γ = 90°. Volume: 1534.7(4) $Å^3$, Z = 4, Density (calculated): 1.222 Mg/m^3, F(000) = 600, Crystal size: 0.211 x 0.167 x 0.123 mm^3, Final R indices [I>2sigma(I)]: R1 = 0.0455, wR2 = 0.1115.

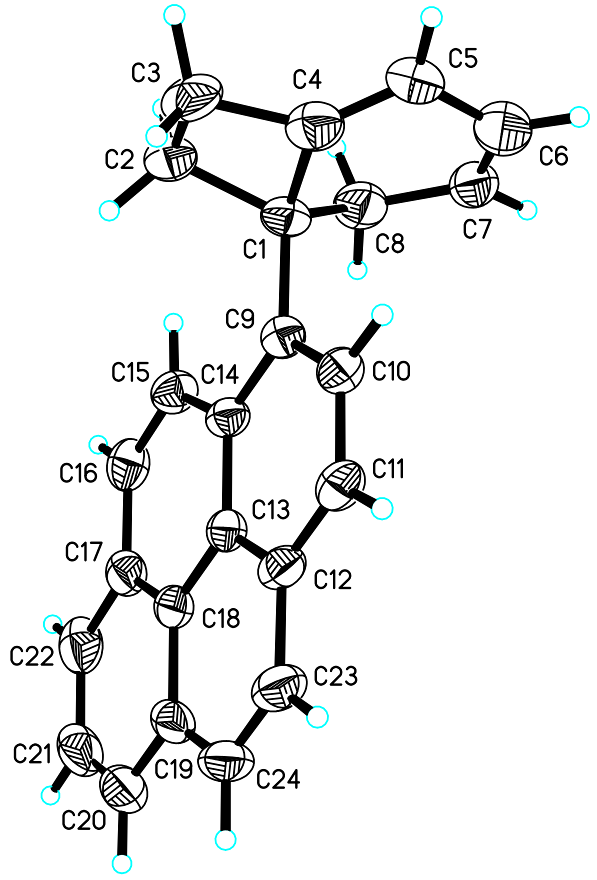

The crystal data of **6c** have been deposited in CCDC with number 1036129. Empirical formula: $C_{24}H_{18}$, Formula weight: 306.38, Crystal system: Monoclinic, Space group: P 21/n, Unit cell dimensions: a = 7.4014(12) Å, α = 90°; b = 11.2513(18) Å, β = 91.021(4)°; c = 19.726(3) Å, γ = 90°. Volume: 1642.4(5) Å^3, Z = 4, Density (calculated): 1.239 Mg/m^3, F(000) = 648, Crystal size: 0.211 x 0.134 x 0.056 mm^3, Final R indices [I>2sigma(I)]: R1 = 0.0744, wR2 = 0.1758.

10. 2D-NMR Data of Compounds 3a, 4b, 5a and 6a.

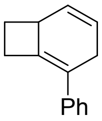

3a

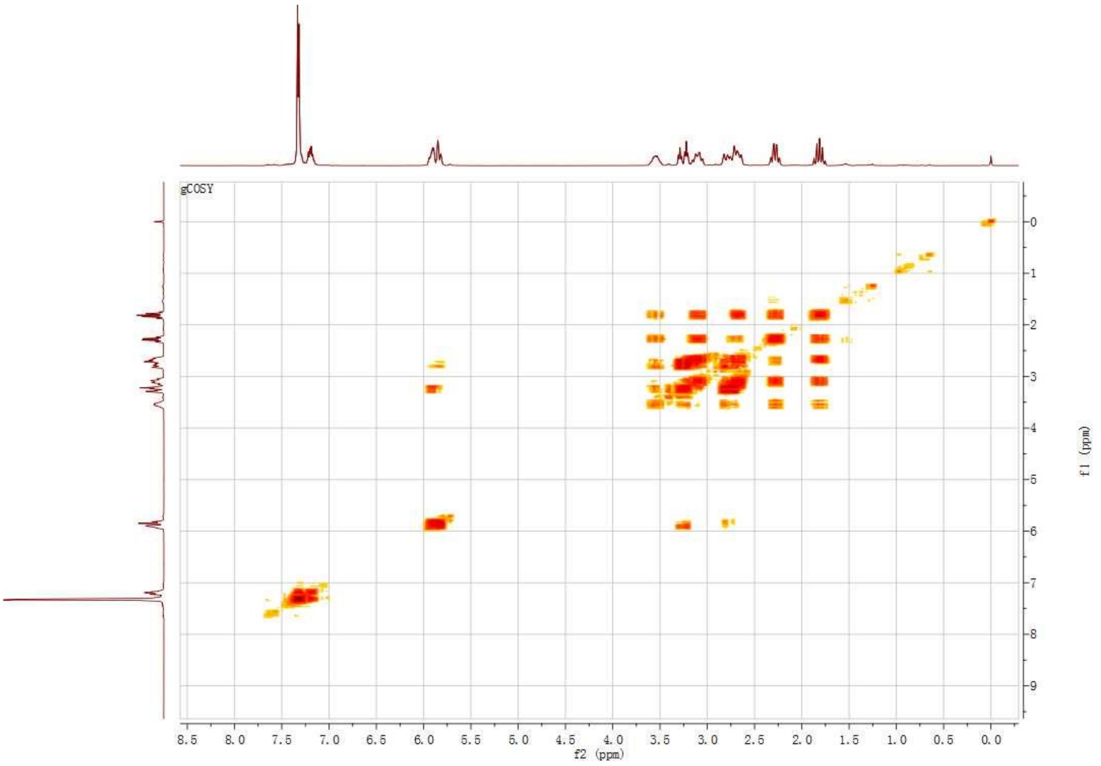

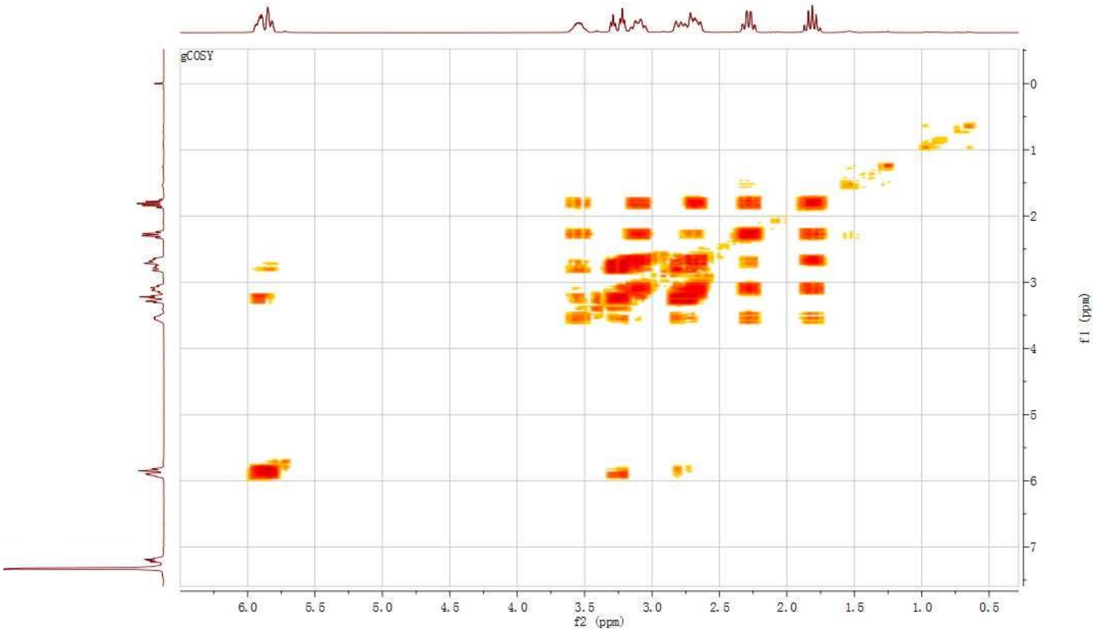

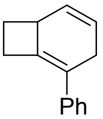

3a

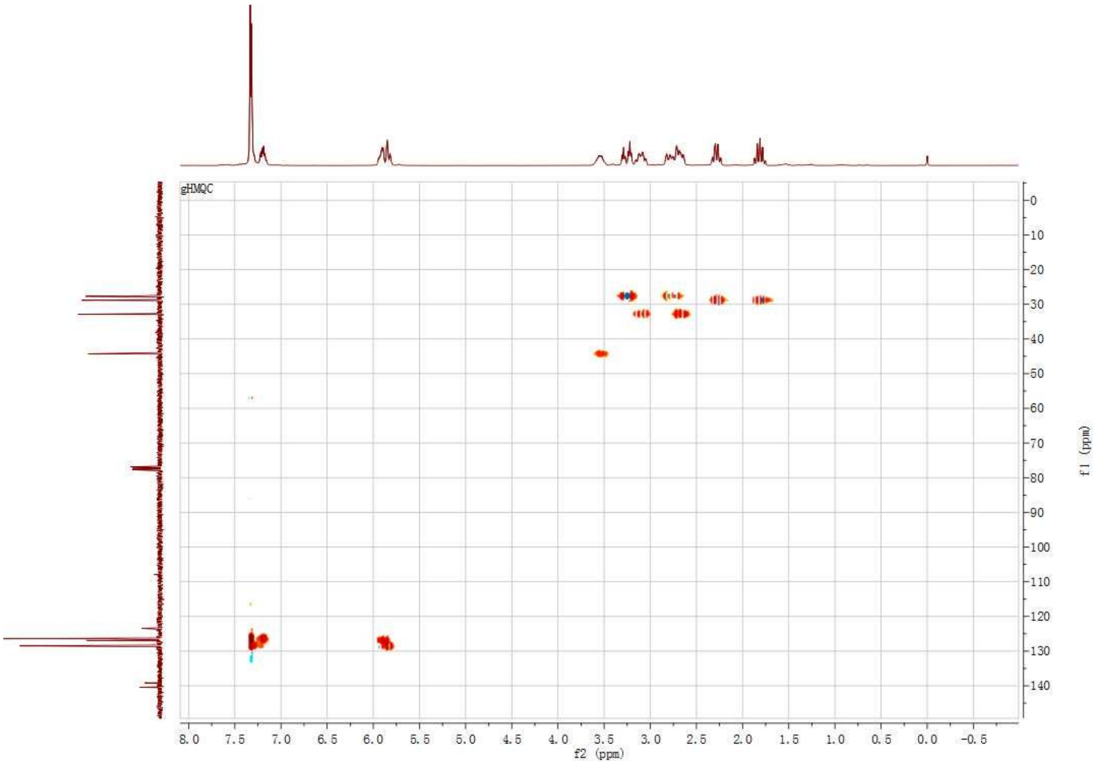

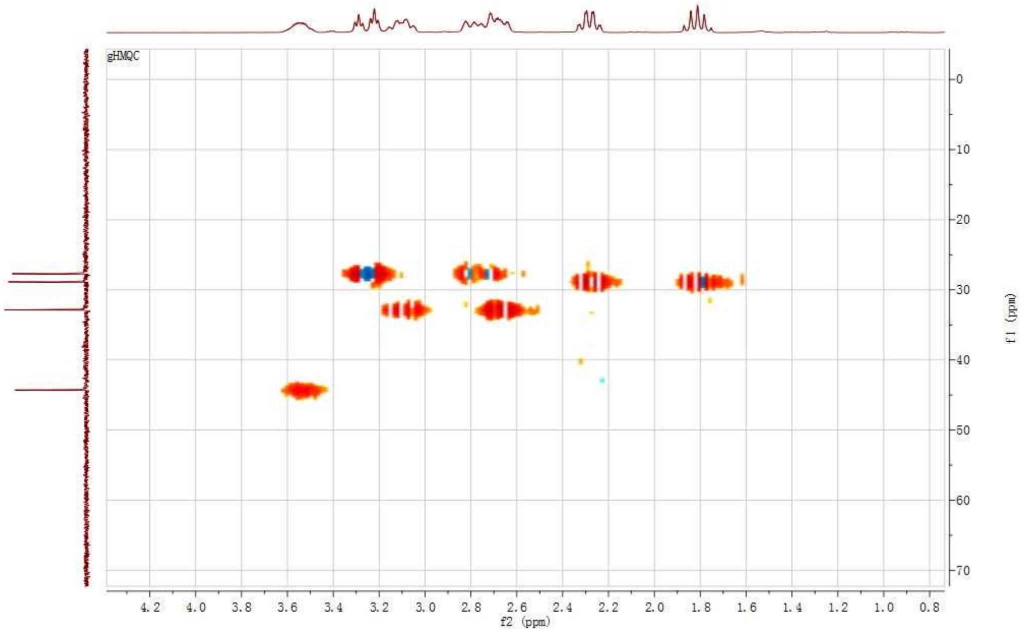

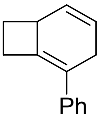

3a

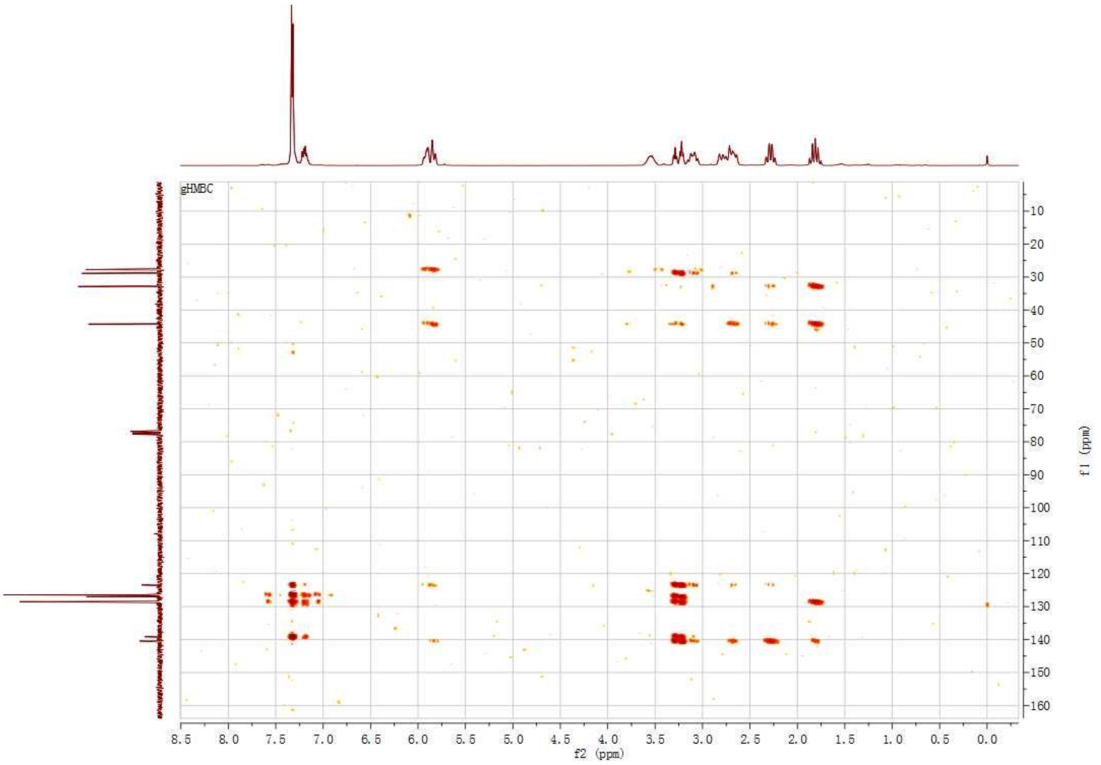

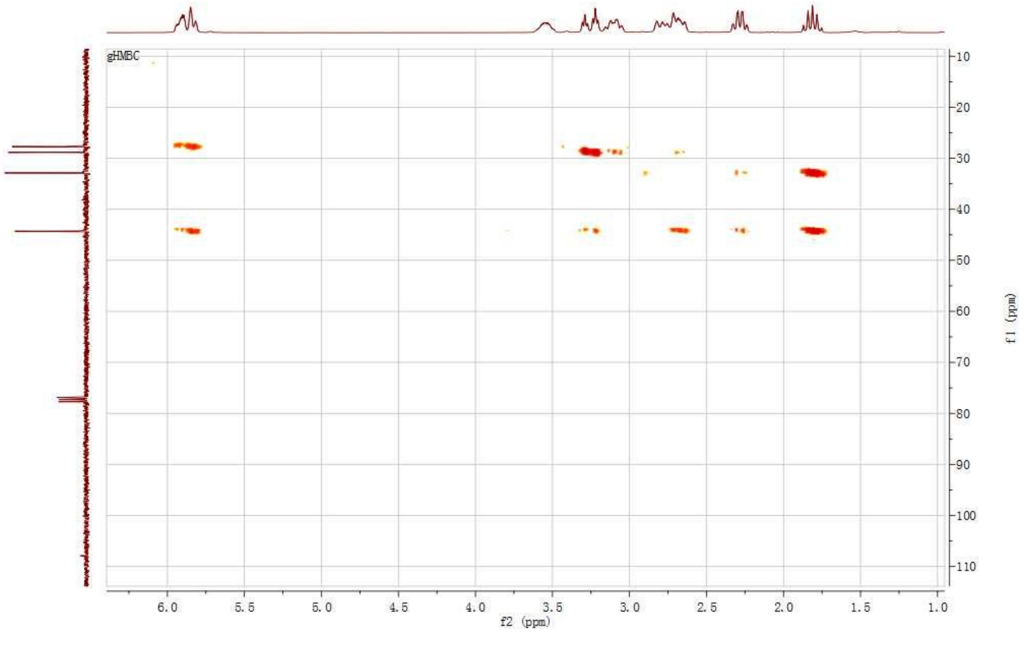

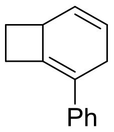

3a

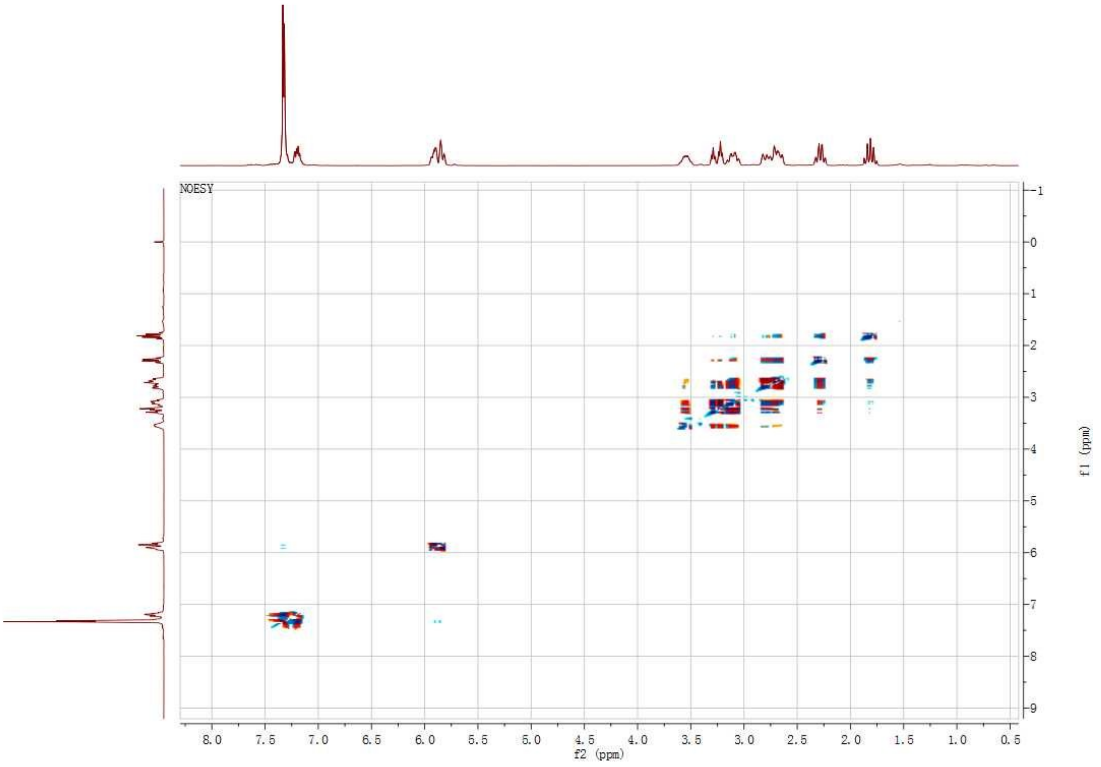

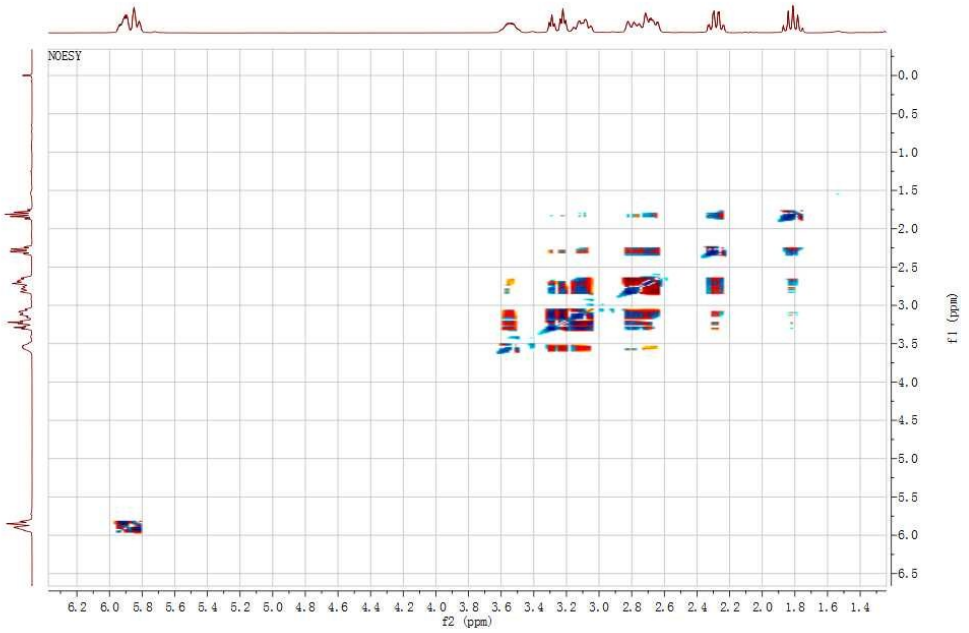

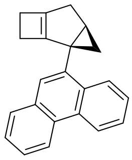

4a

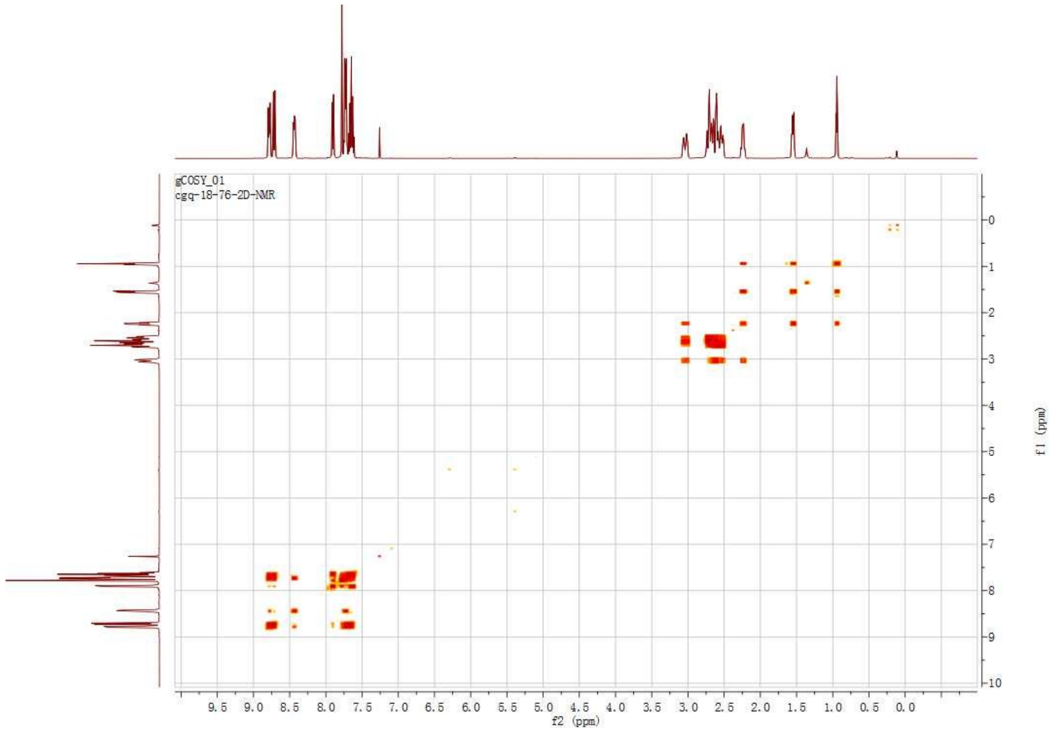
gCOSY_01
cgq-18-76-2D-NMR
f2 (ppm)
f1 (ppm)

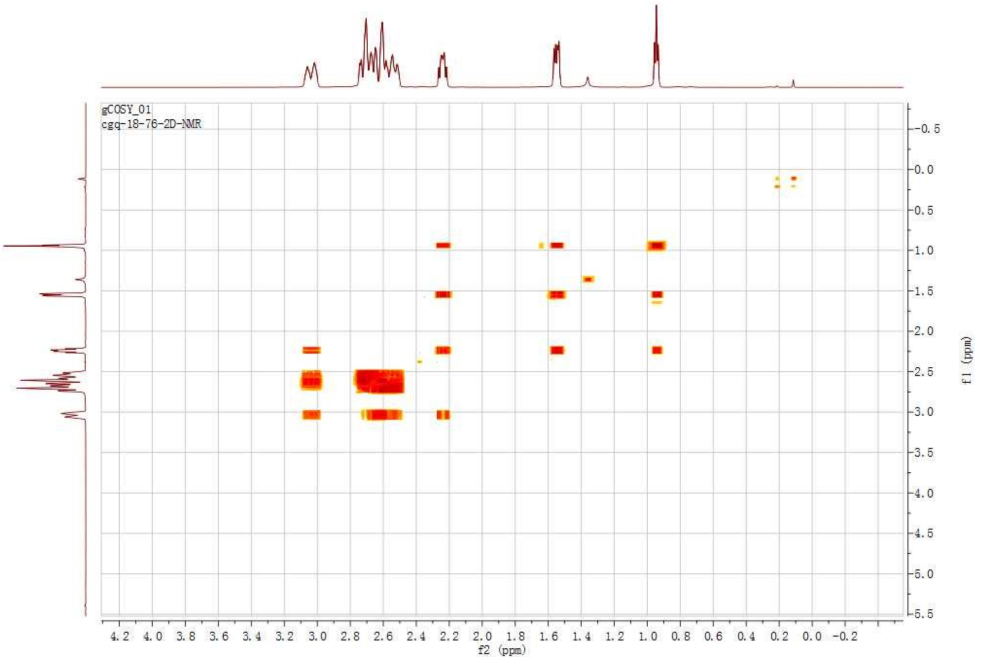
gCOSY_01
cgq-18-76-2D-NMR
f2 (ppm)
f1 (ppm)

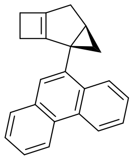

4a

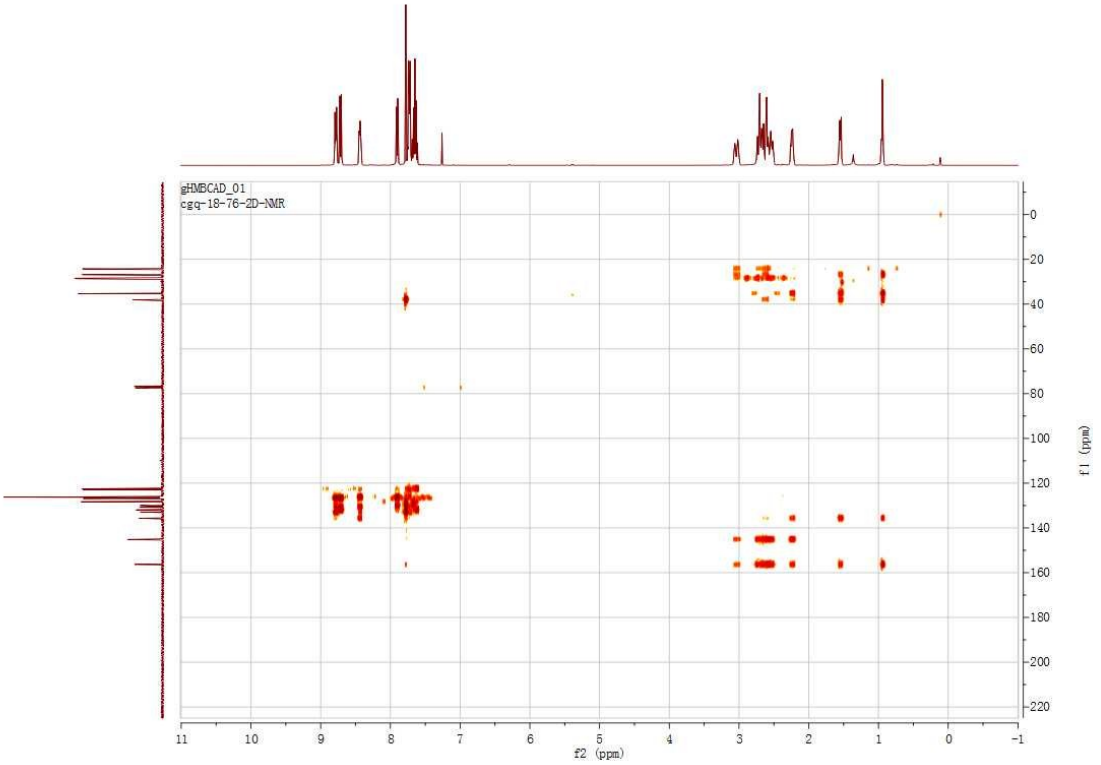
gHMBCAD_01
cgq-18-76-2D-NMR
f1 (ppm)
f2 (ppm)

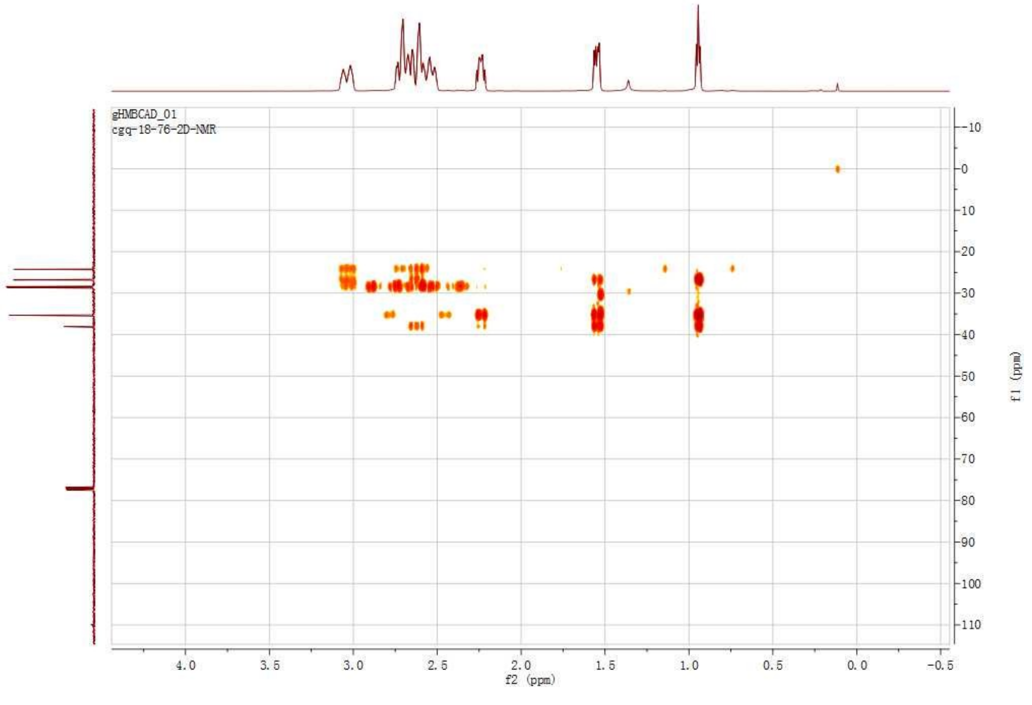
gHMBCAD_01
cgq-18-76-2D-NMR
f1 (ppm)
f2 (ppm)

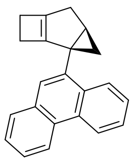

4a

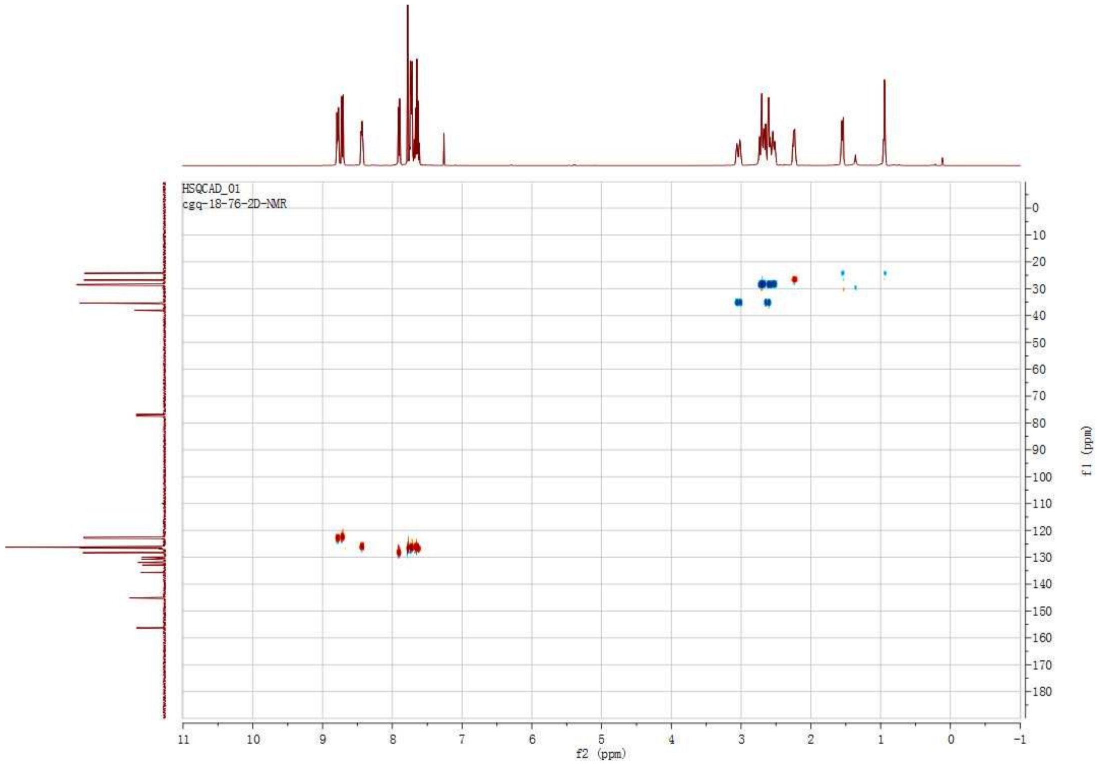
HSQCAD_01
cgq-18-76-2D-NMR
f2 (ppm)
f1 (ppm)

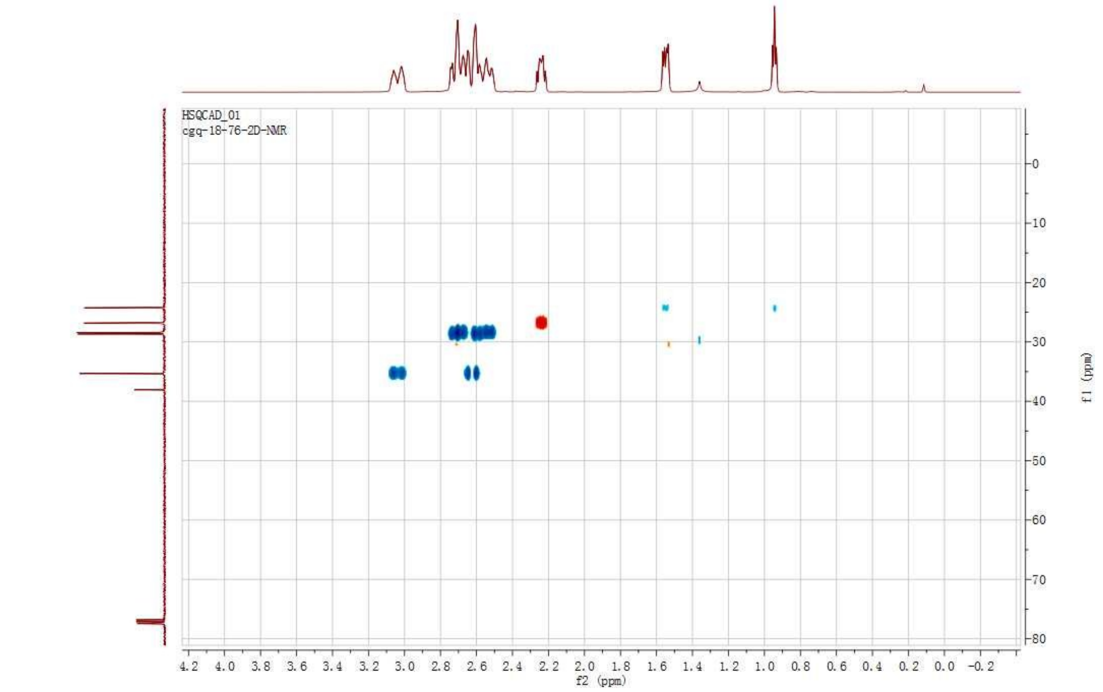
HSQCAD_01
cgq-18-76-2D-NMR
f2 (ppm)
f1 (ppm)

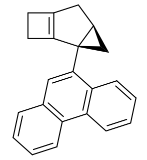

4a

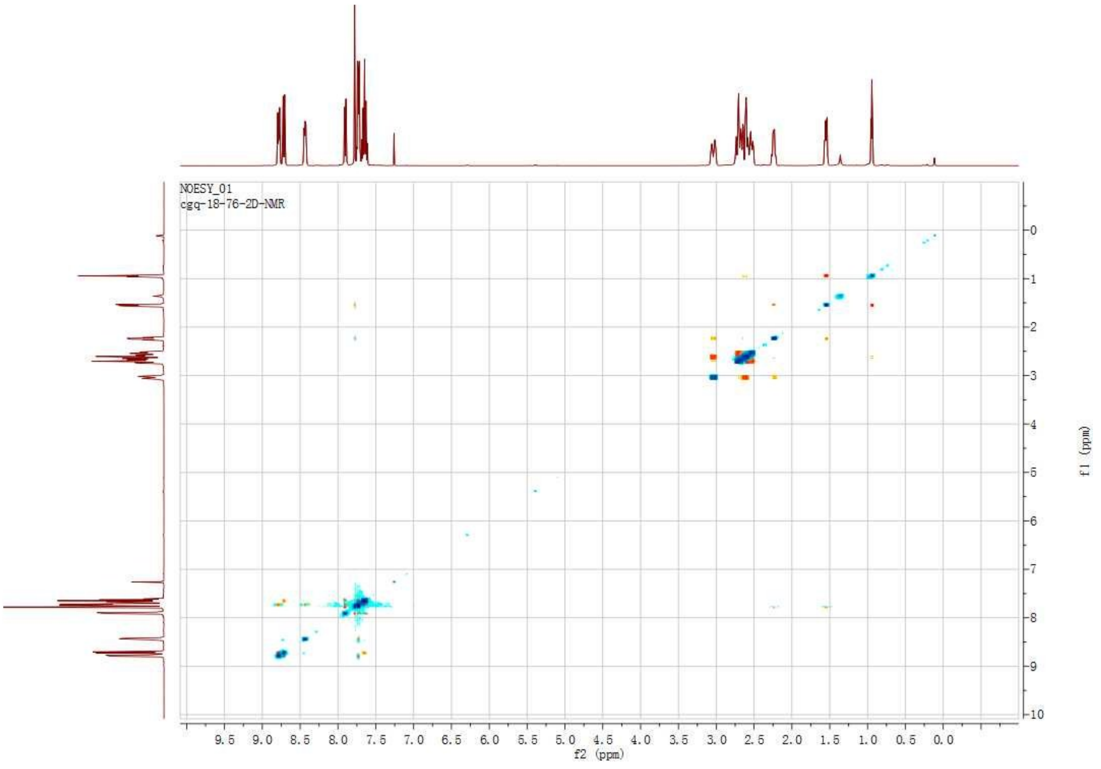
NOESY_01
cgq-18-76-2D-NMR
f2 (ppm)
f1 (ppm)

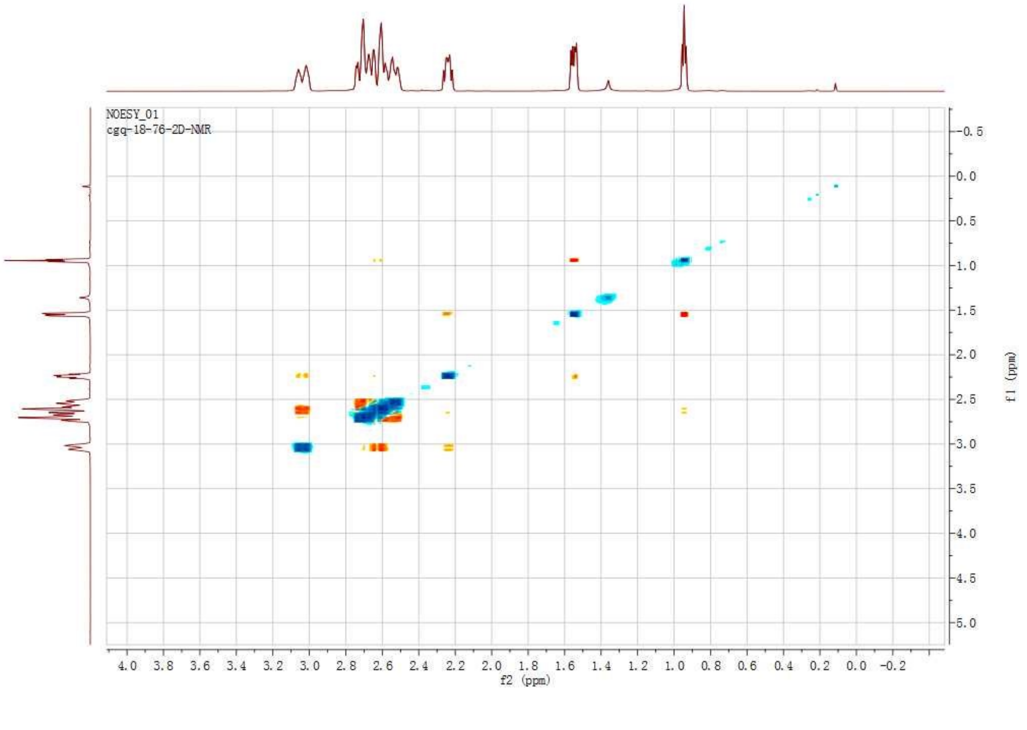
NOESY_01
cgq-18-76-2D-NMR
f2 (ppm)
f1 (ppm)

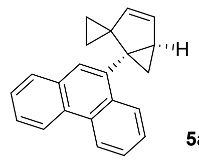
H

5a

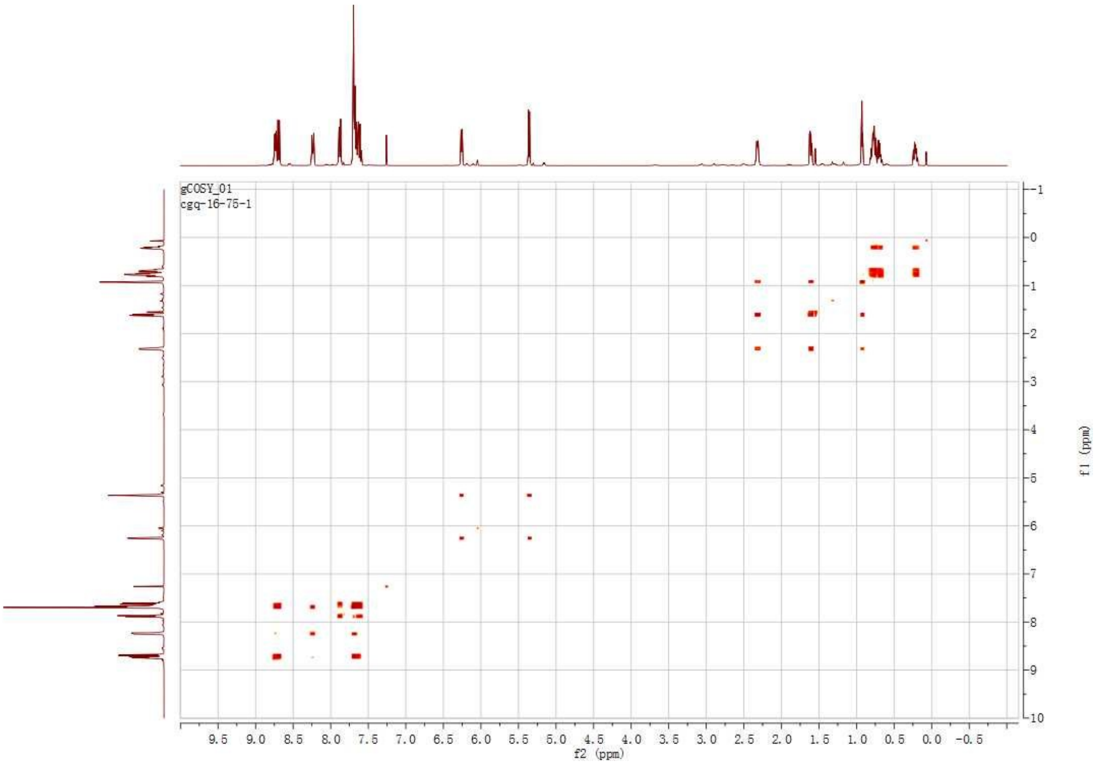
gCOSY_01
cgq-16-75-1
f2 (ppm)
f1 (ppm)

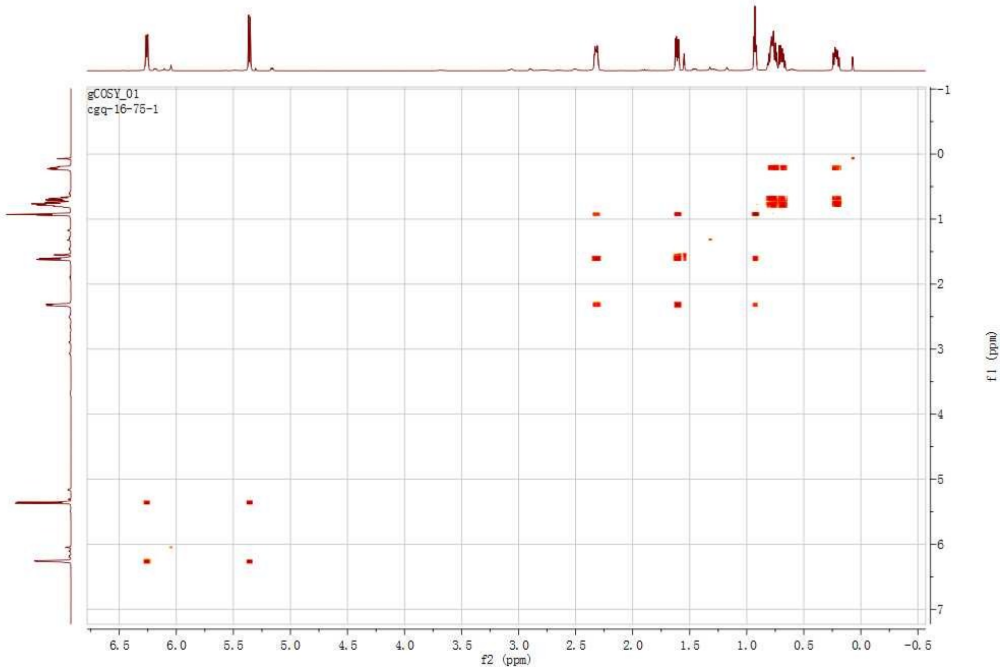
gCOSY_01
cgq-16-75-1
f2 (ppm)
f1 (ppm)

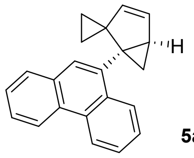

5a

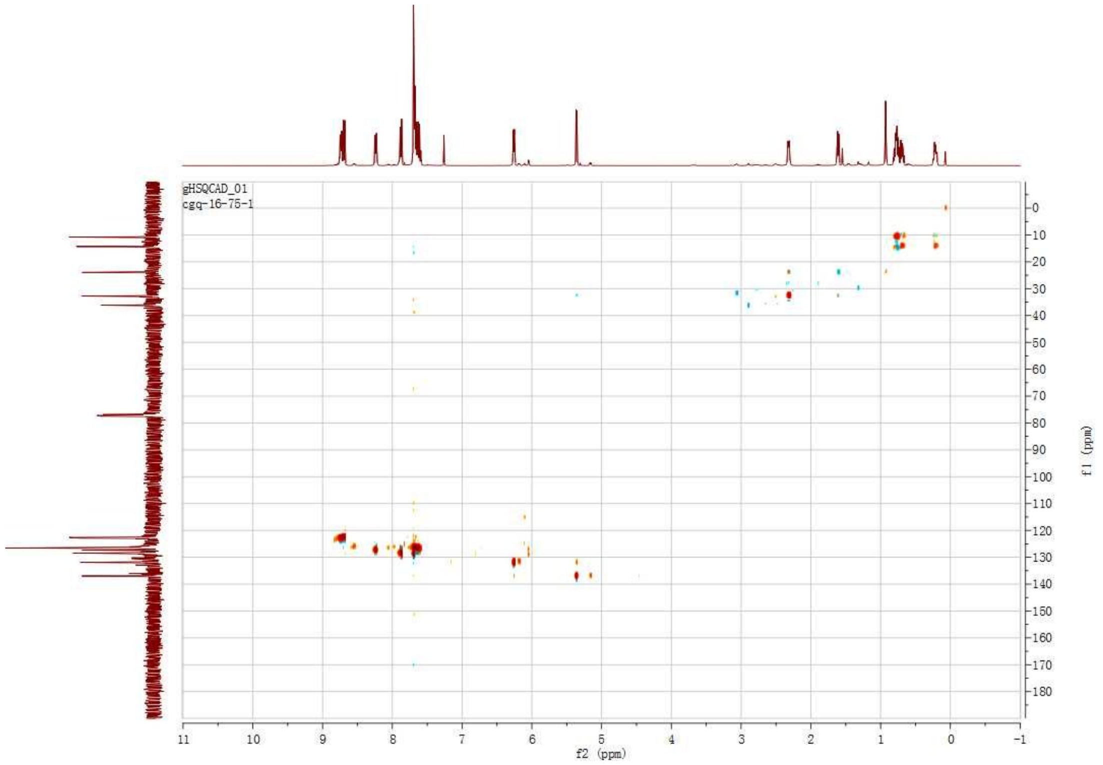
gHSQCAD_01
cgq-16-75-1
f2 (ppm)
f1 (ppm)

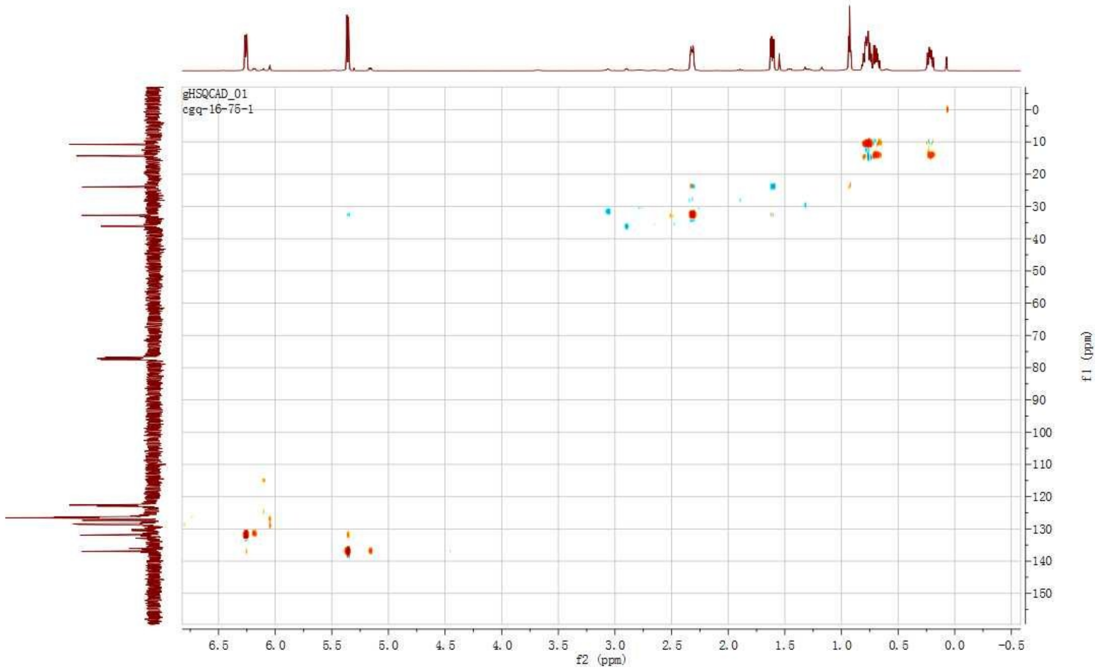
gHSQCAD_01
cgq-16-75-1
f2 (ppm)
f1 (ppm)

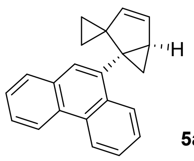

5a

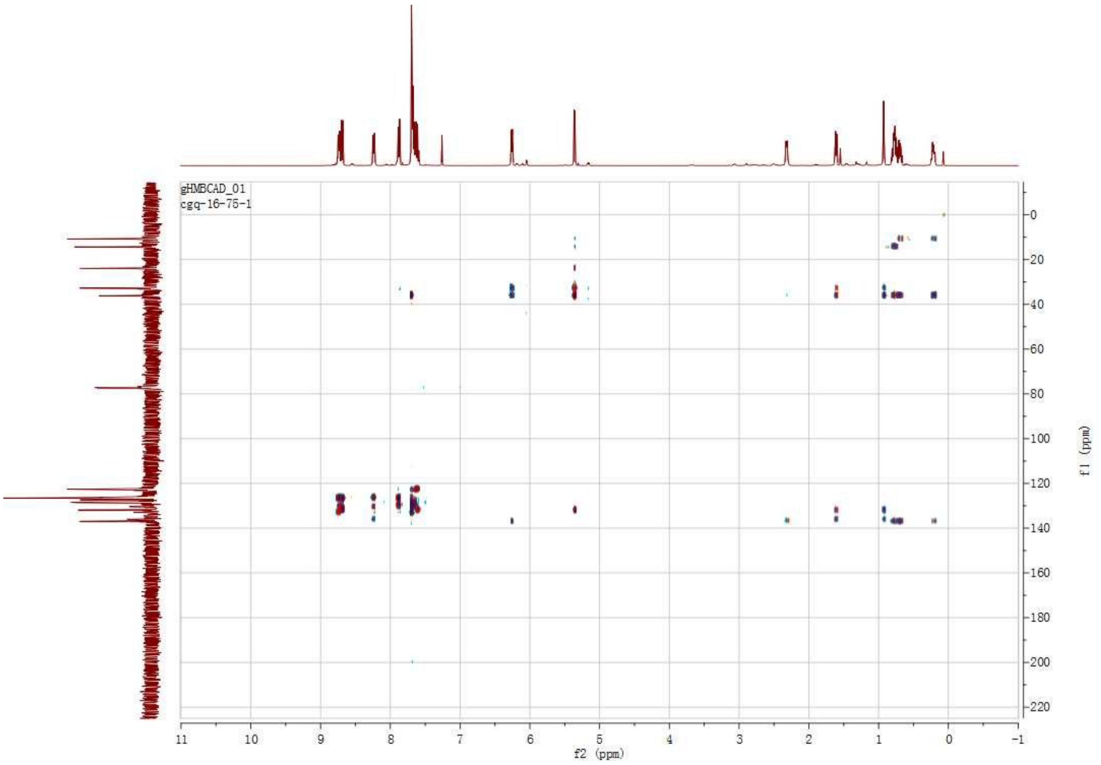

gHMBCAD_01
cgq-16-75-1
f2 (ppm)
f1 (ppm)

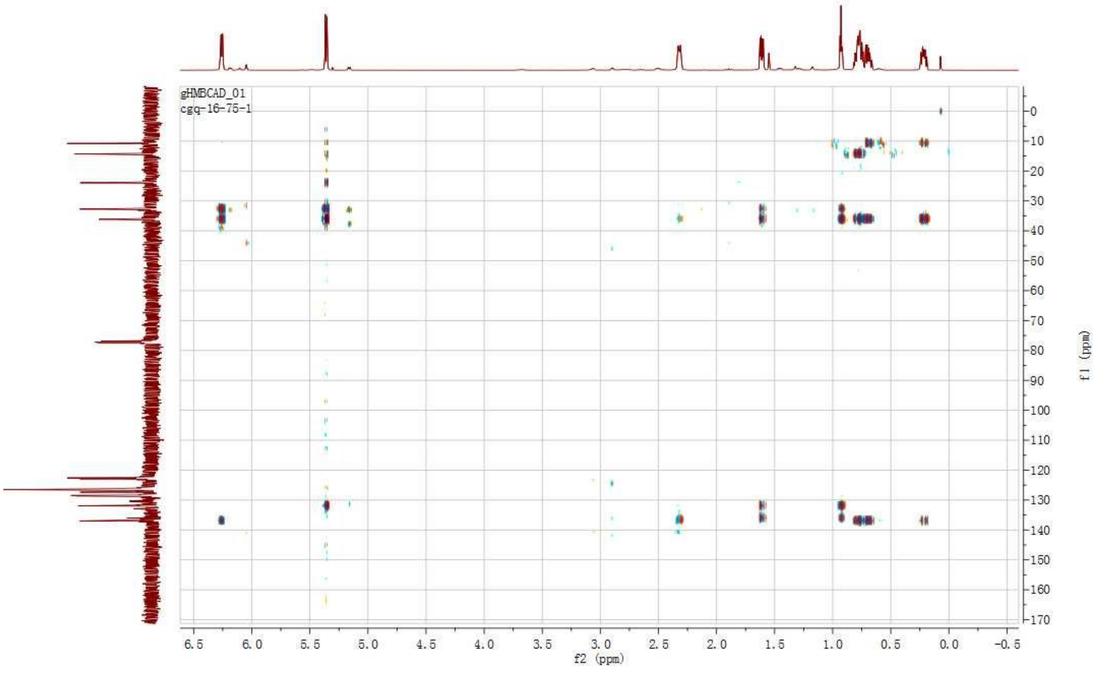

gHMBCAD_01
cgq-16-75-1
f2 (ppm)
f1 (ppm)

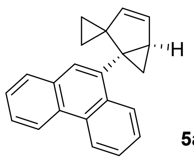

5a

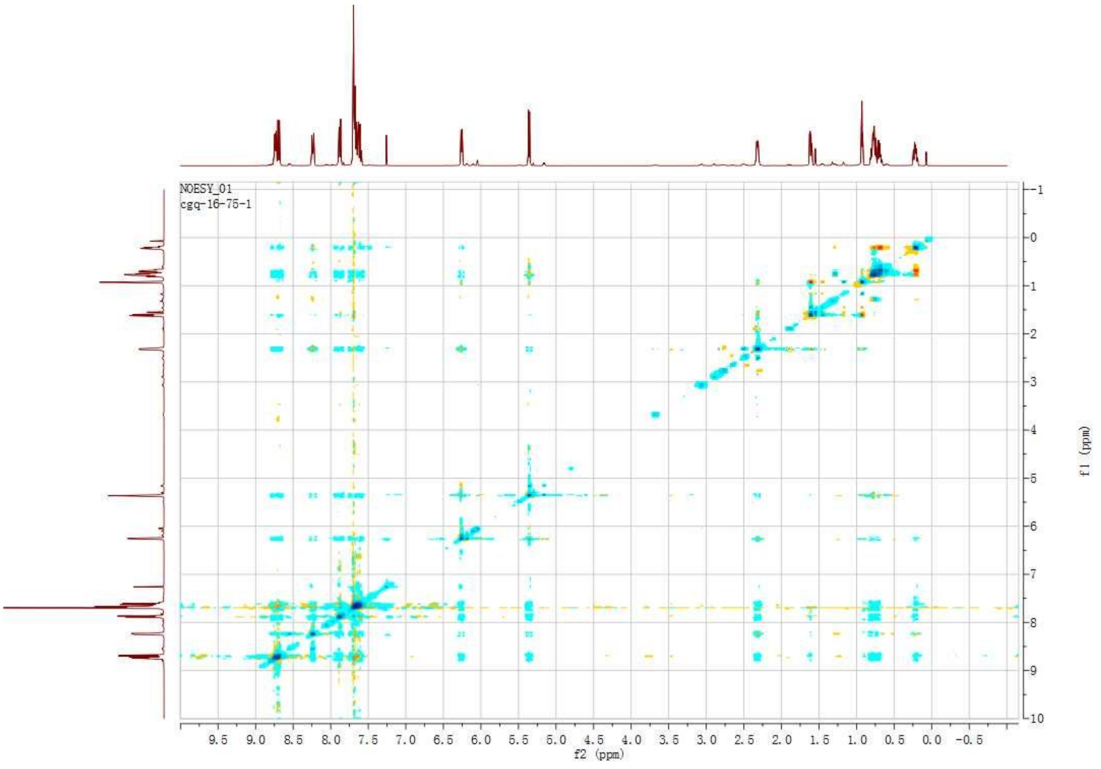
NOESY_01
cgq-16-75-1
f2 (ppm)
f1 (ppm)

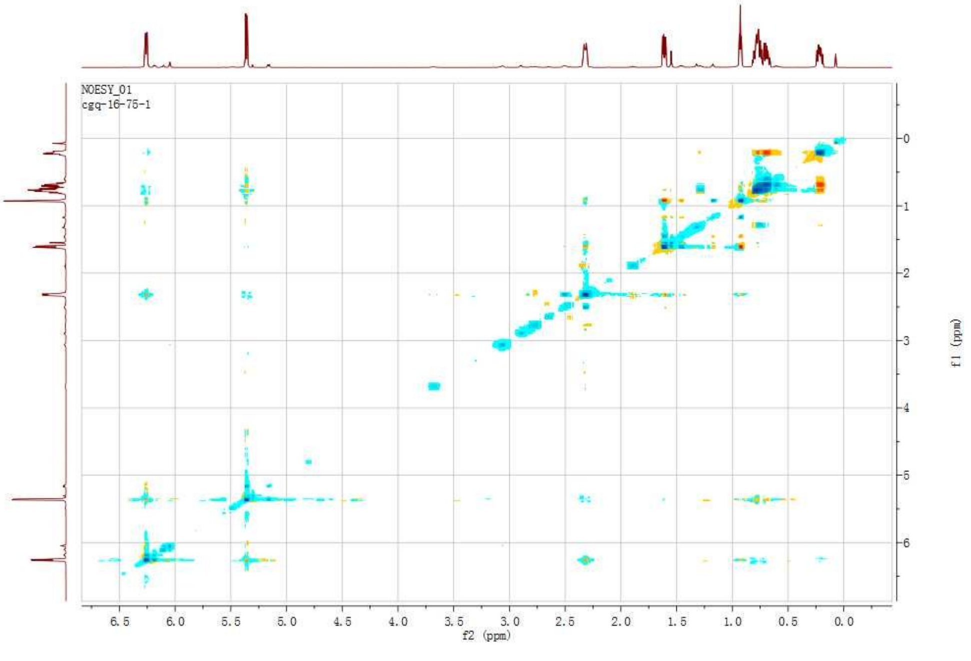
NOESY_01
cgq-16-75-1
f2 (ppm)
f1 (ppm)

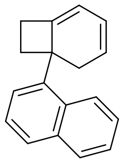

6b

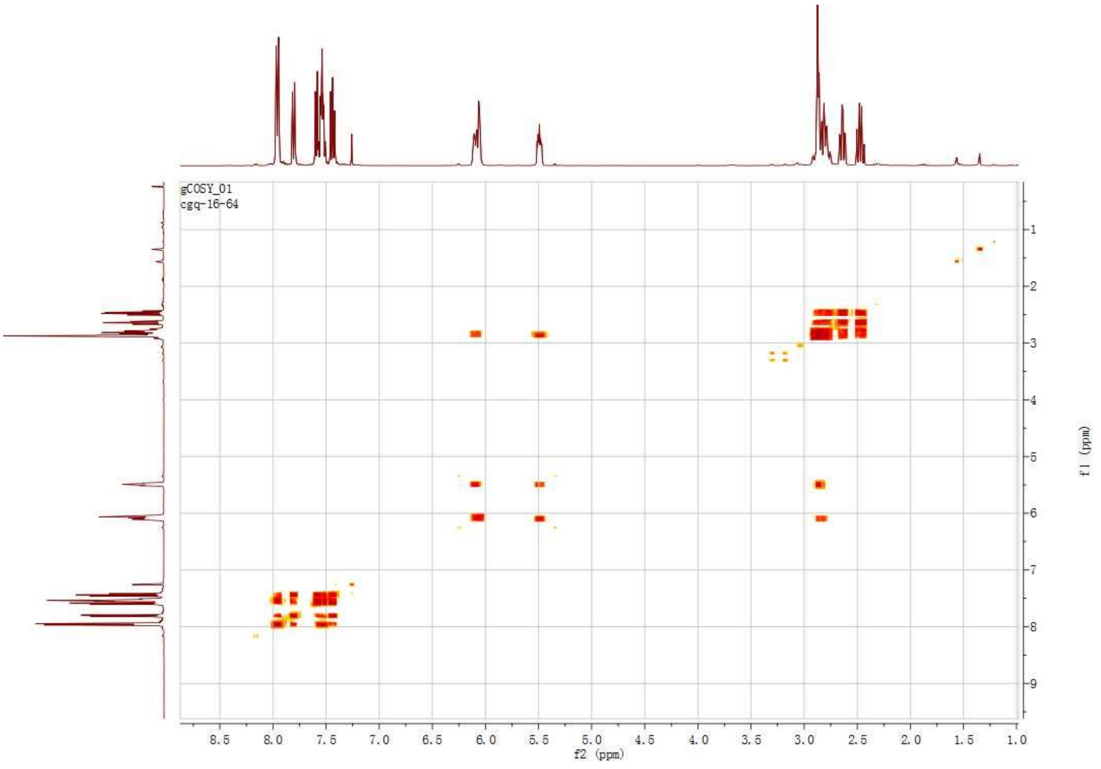

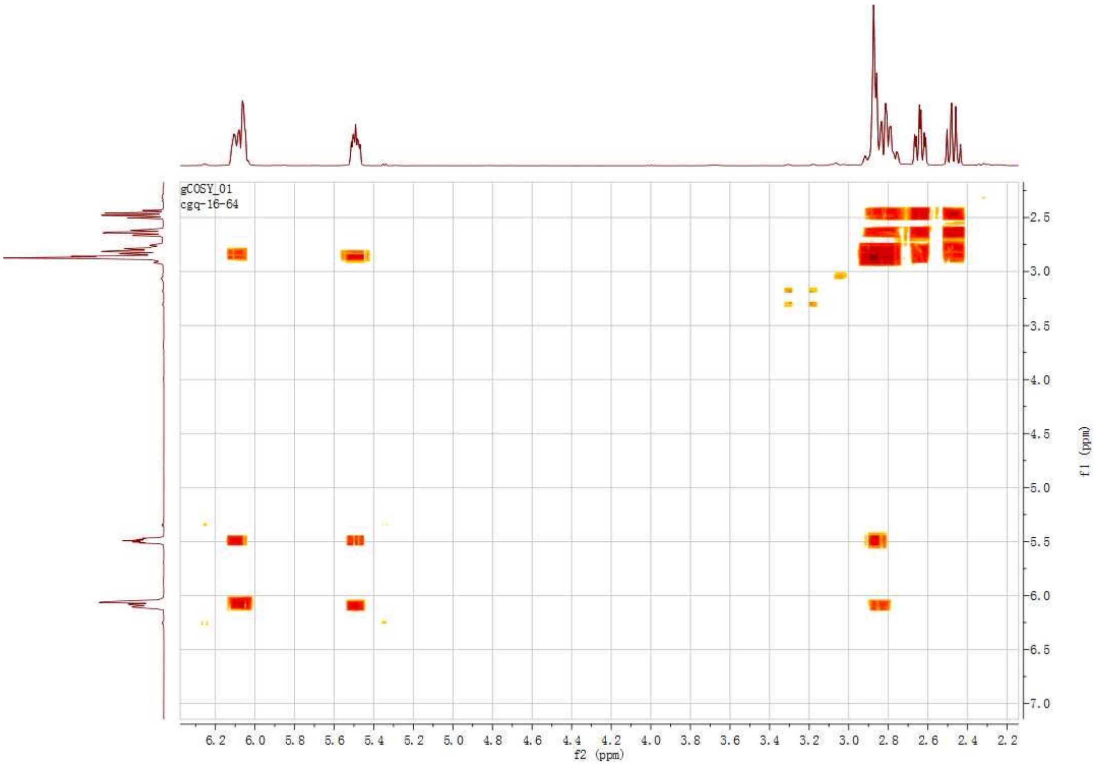

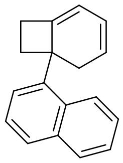

6b

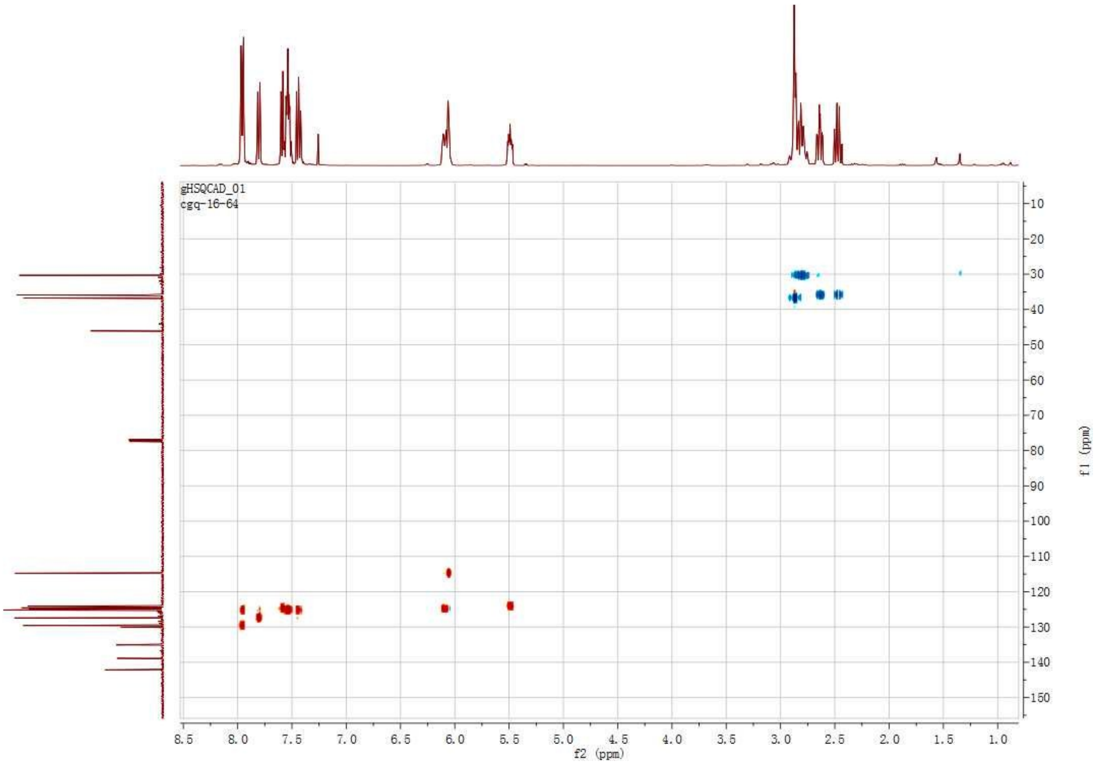
gHSQCAD_01
cgq-16-64
f2 (ppm)
f1 (ppm)

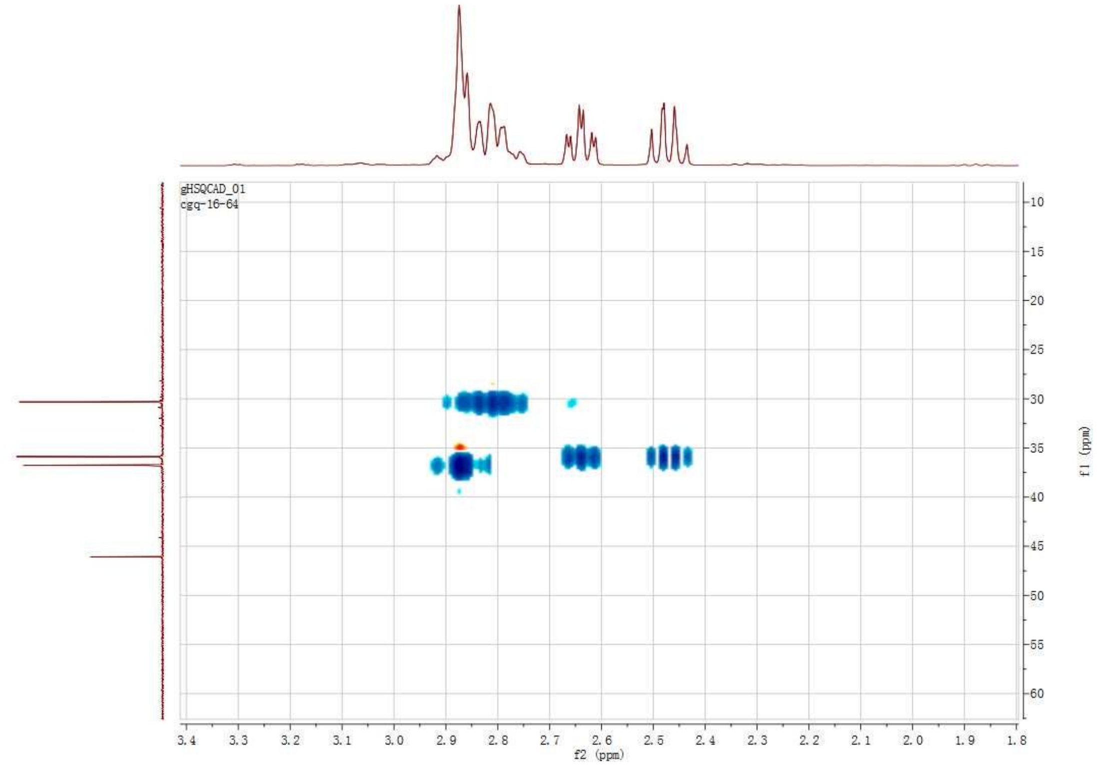
gHSQCAD_01
cgq-16-64
f2 (ppm)
f1 (ppm)

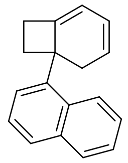

6b

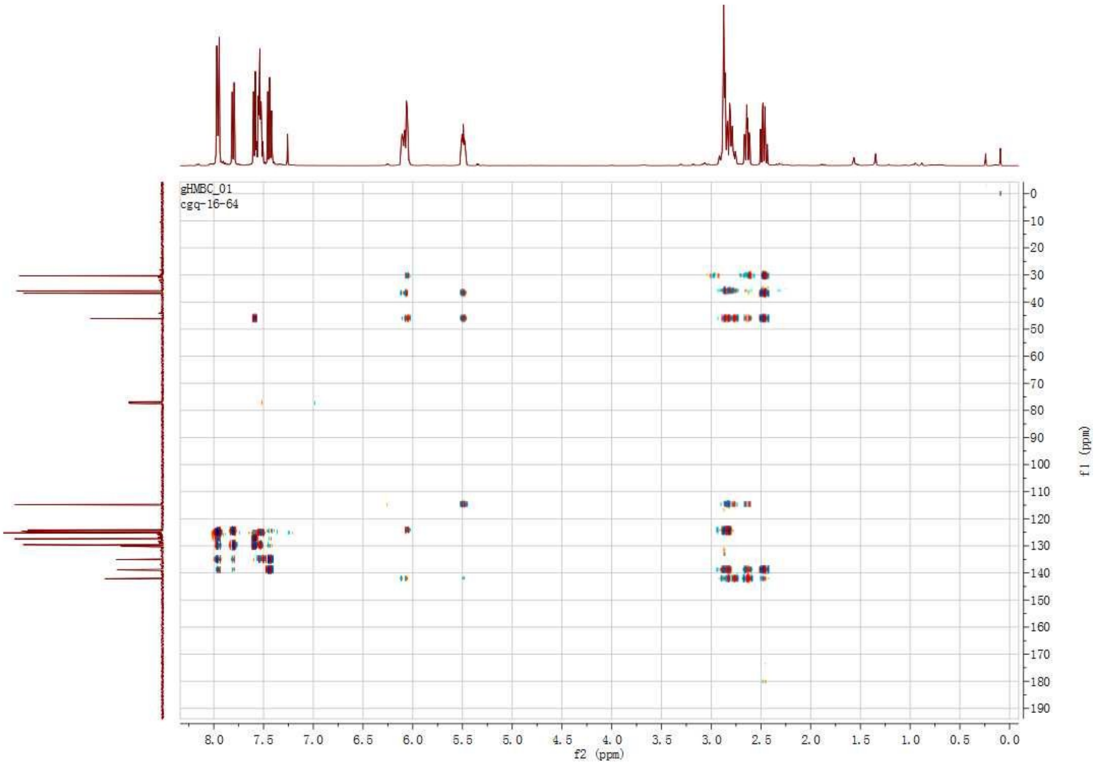

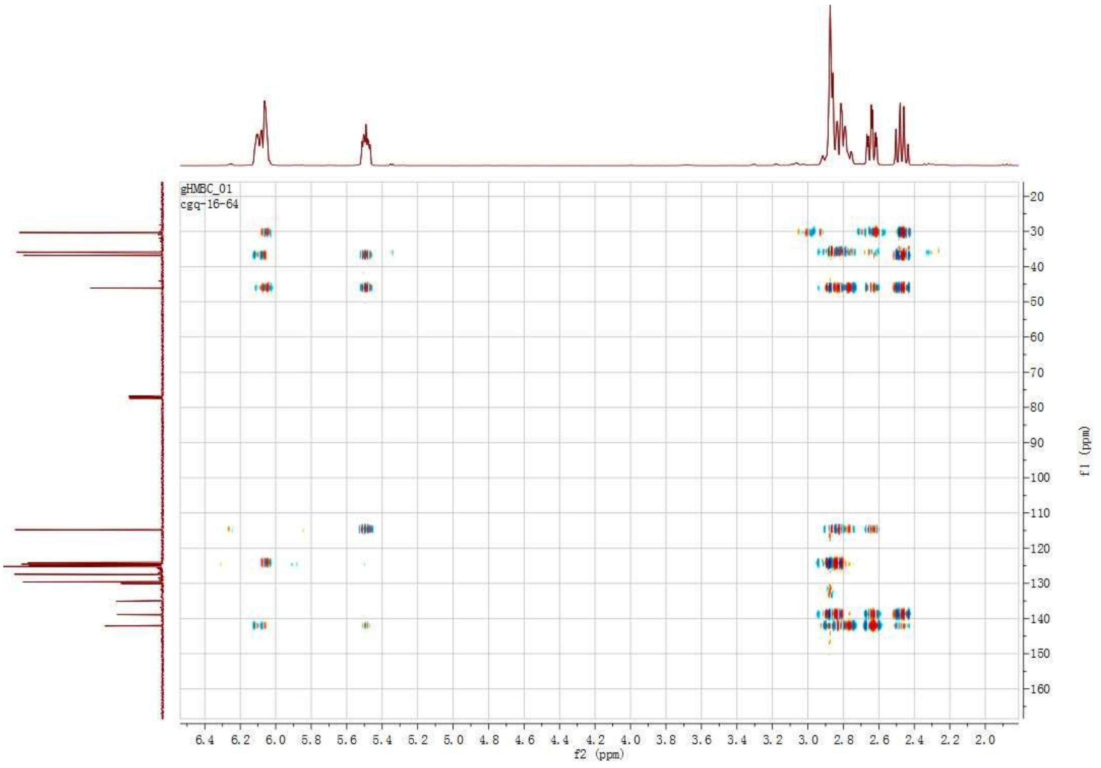

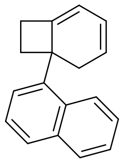

6b

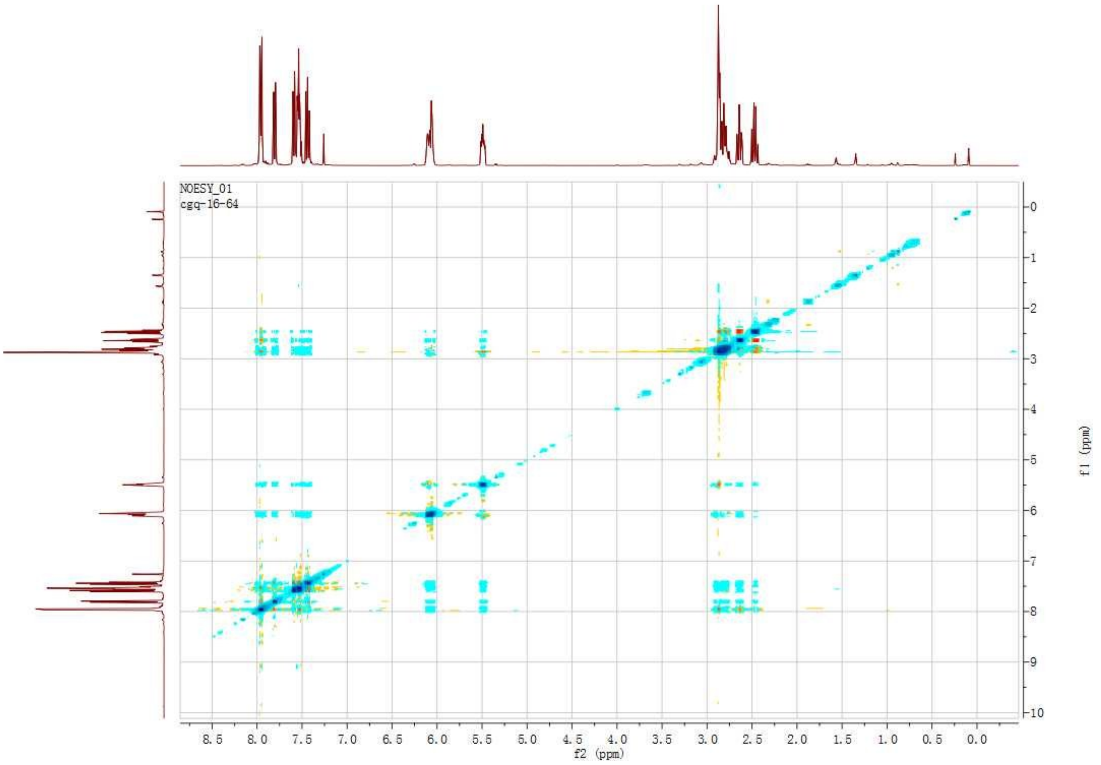

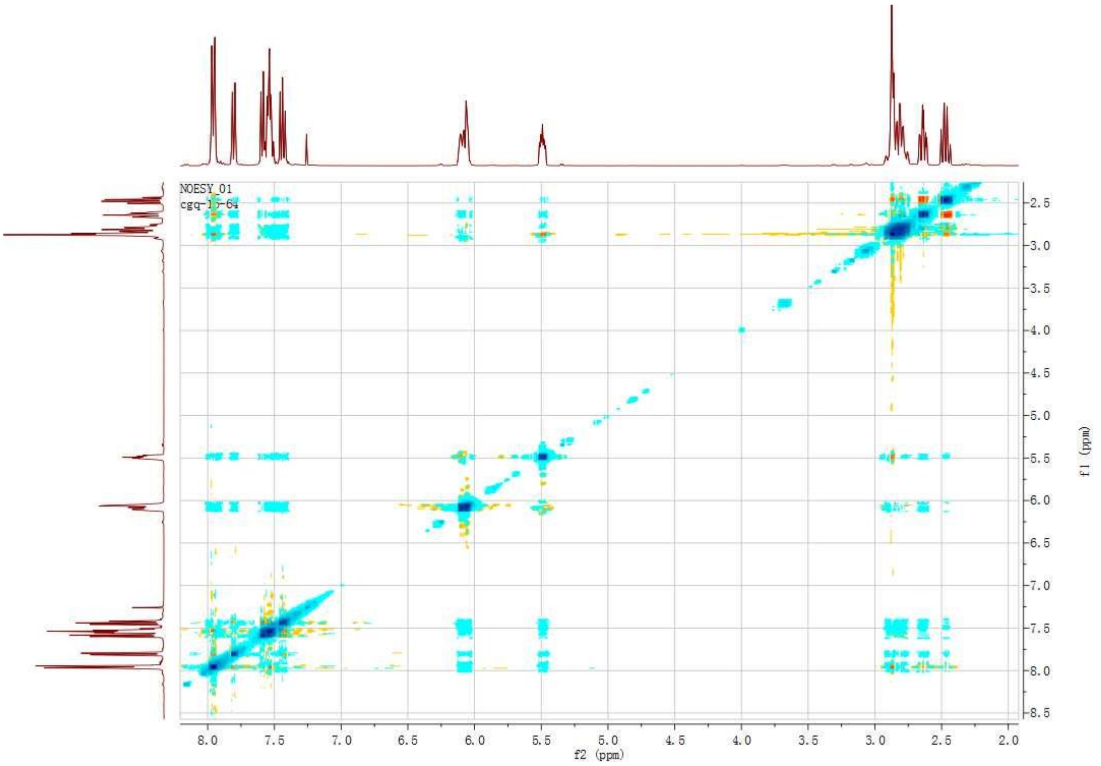

11. Three possible routes for the formation of product 3 from 4

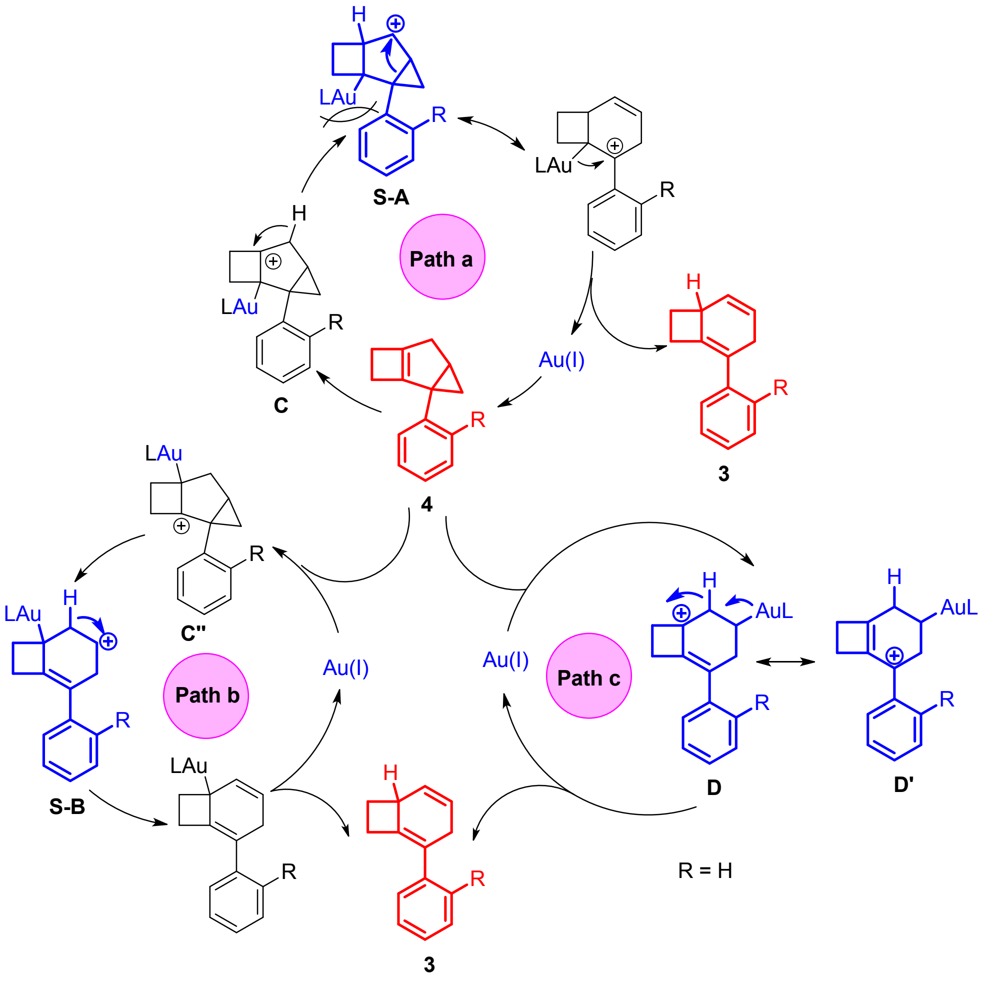

Scheme S9

The formation of product **3** was suggested to obtain through the reaction pathway Cycle II depicted in Scheme 7 in the main text of article. Actually, we initially investigated three possible routes to generate **3** as shown in Scheme S9 (Path a, Path b and Path c). The Path b was ruled out based on both the deuterium labeling experiment and theoretical calculations, since this reaction route was conflicted to the deuterium labeling experiment's results and the intermediate **S-B** could not be located theoretically. Both Path a and Path c were in consistence with the deuterium labeling experiment's results. However, the intermediate **S-A** in Path a could not be located theoretically, probably due to the steric hindrance between the ligand and the aryl group. Thus, the Path a was also ruled out. The most possible reaction route Path c is proposed as the Cycle II in Scheme 7.

12. Another mechanism for the formation of products 5b and 6b through intermediate 4b’

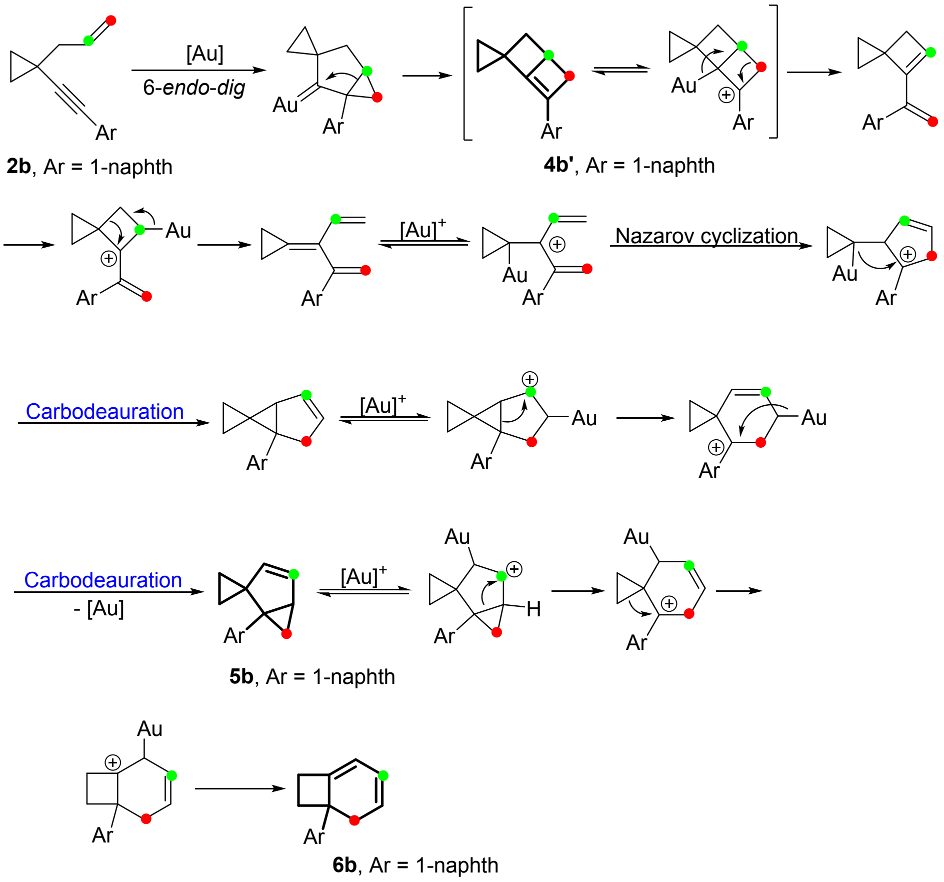

Scheme S10

Another mechanism has been initially proposed for the formation of products **5b** and **6b** via intermediate **4b’** that is a constitutional isomer of **4b**. Compounds **4b** and **4b’** have very similar ^{1}H-NMR ^{13}C-NMR and DEPT spectra (**4b’** was formed through a formal intramolecular [2+2] cycloaddition in the presence of gold catalyst. As for gold catalyzed [2+2] cycloaddition, see Obradors, C.; Echavarren, A. M. *Acc. Chem. Res.* **2014**, *47*, 902). However, this mechanism was conflicted to the deuterium labeling experiment’s results, and was excluded.

13. Computational Details

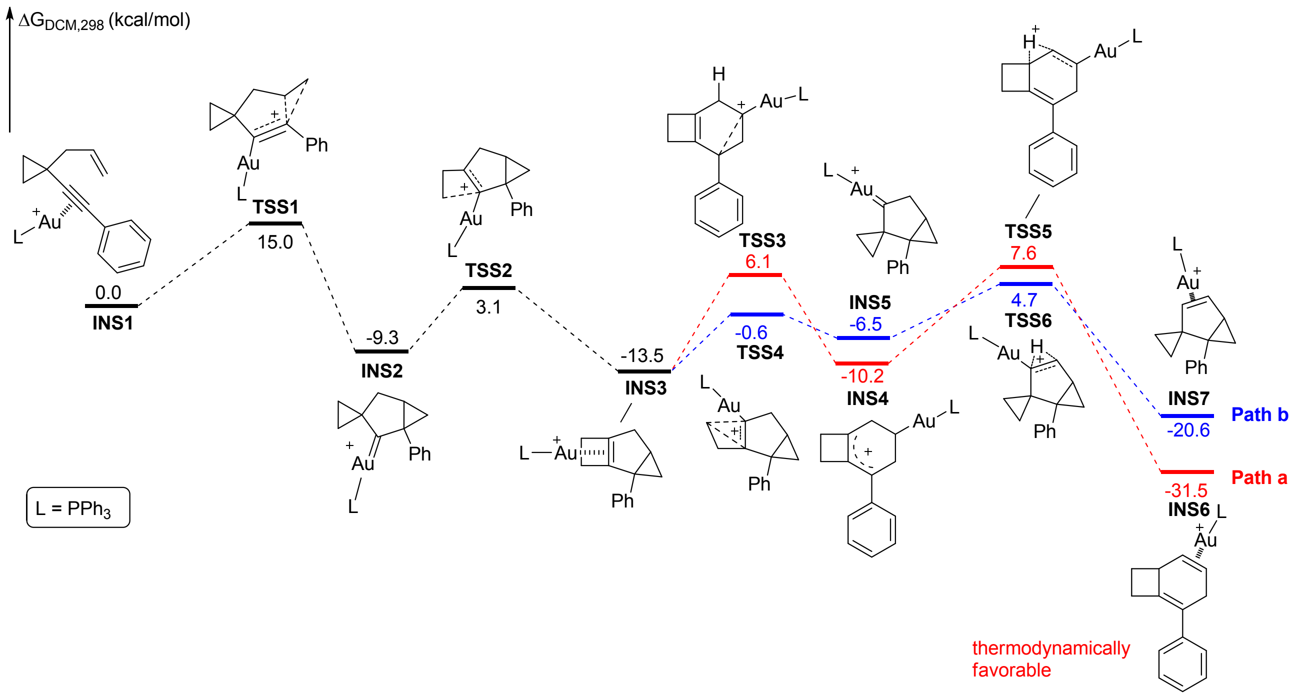

Scheme S11

Table SI-2. The total energies, enthalpies and free energies of the lowest conformers of all species shown in Scheme 9.

	E_{tot} (E_h)[a]	H_{298}[a]	G_{298}[a]	G_{DCM}[b]
**IN1**	-1795.409664	-1794.708288	-1794.823014	-1794.994258
**TS1**	-1795.392126	-1794.691972	-1794.803445	-1794.970724
**IN2**	-1795.440729	-1794.738231	-1794.847132	-1795.014332
**TS2**	-1795.414112	-1794.71318	-1794.819057	-1794.988891
**IN3**	-1795.443929	-1794.740923	-1794.847150	-1795.013708
**TS3**	-1795.416056	-1794.714296	-1794.819643	-1794.985217
**IN4**	-1795.435591	-1794.733321	-1794.846133	-1795.013995
**TS5**	-1795.403003	-1794.703656	-1794.811670	-1794.984100
**IN6**	-1795.470217	-1794.766305	-1794.875766	-1795.048970
**TS4**	-1795.423112	-1794.721647	-1794.829082	-1794.996887
**IN5**	-1795.431279	-1794.729411	-1794.839255	-1795.008039
**TS6**	-1795.405754	-1794.707289	-1794.815886	-1794.989270
**IN7**	-1795.455216	-1794.752938	-1794.861716	-1795.033181

a. Calculated at the PBE1PBE/6-31G(d)/SDD level of theory;

b. SMD/PBE1PBE/6-31+G(d,p)// PBE1PBE/6-31G(d)/SDD

Table SI-3. The total energies, enthalpies and free energies of the lowest conformers of all species shown in Scheme 10.

	E_{tot} (E_h)[a]	H_{298}[a]	G_{298}[a]	G_{DCM}[b]
**IN8**	-4365.88657	-4365.193761	-4365.31192	-4365.487226
**TS7**	-4365.869048	-4365.177625	-4365.293935	-4365.465775
**IN9**	-4365.920432	-4365.226293	-4365.337504	-4365.509414
**TS8**	-4365.892703	-4365.199944	-4365.308291	-4365.482132
**IN10**	-4365.923198	-4365.228481	-4365.336308	-4365.502751
**TS9**	-4365.890328	-4365.197297	-4365.30551	-4365.471503
**IN11**	-4365.902778	-4365.209454	-4365.324168	-4365.495742
**TS11**	-4365.873289	-4365.182705	-4365.29489	-4365.470252
**IN13**	-4365.947328	-4365.25206	-4365.365309	-4365.541846
**TS10**	-4365.900291	-4365.207296	-4365.317242	-4365.488422
**IN12**	-4365.911315	-4365.217919	-4365.33054	-4365.503239
**TS12**	-4365.88695	-4365.197103	-4365.308562	-4365.483791
**IN14**	-4365.934205	-4365.240202	-4365.351058	-4365.527037

a. Calculated at the PBE1PBE/6-31G(d)/SDD level of theory;
b. SMD/PBE1PBE/6-31+G(d,p)// PBE1PBE/6-31G(d)/SDD

Table SI-4. The total energies, enthalpies and free energies of the lowest conformers of all species shown in Scheme 11.

	E_{tot} (E_h)[a]	H_{298}[a]	G_{298}[a]	G_{DCM}[b]
**IN15**	-4405.915598	-4405.061034	-4405.197758	-4406.113513
**TS13**	-4405.897622	-4405.043300	-4405.178294	-4406.092502
**IN16**	-4405.947934	-4405.090951	-4405.224125	-4406.144192
**TS14**	-4405.920048	-4405.064981	-4405.193771	-4406.117400
**IN17**	-4405.946480	-4405.088939	-4405.219469	-4406.139850
**TS15**	-4405.913119	-4405.057331	-4405.185678	-4406.104955
**IN18**	-4405.929729	-4405.073669	-4405.208626	-4406.123733
**TS17**	-4405.89896	-4405.045557	-4405.17826	-4406.099847
**IN20**	-4405.973085	-4405.114907	-4405.249902	-4406.173843
**TS16**	-4405.929699	-4405.073969	-4405.206287	-4406.123981
**IN19**	-4405.940683	-4405.084493	-4405.219073	-4406.136624
**TS18**	-4405.914565	-4405.061783	-4405.194823	-4406.113794
**IN21**	-4405.960543	-4405.103759	-4405.236965	-4406.160204

a. Calculated at the PBE1PBE/6-31G(d)/SDD level of theory;
b. SMD/PBE1PBE/6-31+G(d,p)// PBE1PBE/6-31G(d)/SDD

14. Archive Entries

IN1

1\1\GINC-SHI_03\FOpt\RPBE1PBE\GenECP\C34H41Au1P1(1+)\YIN\23-Oct-2015\0
\\#p genecp pbe1pbe opt\\Title Card Required\\1,1\C,0.0005714896,0.001
0399139,0.000079676\C,0.0006170066,0.0007399565,1.3888709651\C,1.22036
00898,0.0000096612,2.0857078132\C,2.4319744921,0.0067775983,1.37661818
6\C,2.418768279,0.0068979458,-0.0120802696\C,1.2061769393,0.005121111,
-0.7005123255\H,-0.9425921793,-0.0042408482,-0.538022314\H,-0.93297862
04,-0.0059067642,1.943355819\H,3.3679614031,0.0062594923,1.9275053043\
H,3.3558826768,0.0053137484,-0.5609203165\H,1.2003516677,0.0043340361,
-1.7866355809\C,1.2090935389,-0.0323110894,3.5203932691\C,1.0031920735
,-0.2195554093,4.7317852116\C,0.6696948498,-0.8398470187,5.9985629595\
C,0.1132773644,-2.2498485132,5.893160184\C,1.4954616693,-2.0443589309,
6.4124360446\H,-0.003461246,-2.6798941532,4.9033306819\H,-0.684603767,
-2.4905270731,6.5904807701\H,1.6771133008,-2.1603296157,7.4777407977\H
,2.3247877317,-2.3276550289,5.7722777931\C,0.0991099639,0.0637863903,7
.0852785021\H,0.821201374,0.8717987536,7.2810418481\H,0.0210059032,-0.
5167964599,8.0119345215\C,-1.2433571318,0.6289619884,6.7287509414\H,-1
.3009197664,1.1786807926,5.7874782476\C,-2.3409179252,0.4879975543,7.4
69043117\H,-2.3284740018,-0.0543581316,8.4125280132\H,-3.2932501014,0.
9129877611,7.1651777227\Au,1.9636004901,1.840254092,4.5635877131\P,2.5
895107697,4.0849107858,4.7501763483\C,1.5854516477,4.8435362616,6.1765
152317\C,0.176248784,4.2334095326,6.1612600207\C,2.2864672383,4.414337
7611,7.4720917771\C,1.4450761072,6.3687950404,6.1480758921\H,-0.380886
5433,4.469148226,5.2504309737\H,0.2013140982,3.1446641736,6.2726113153
\H,-0.3890183357,4.6398895151,7.0089677082\H,3.2764972874,4.8669446369
,7.5795598485\H,1.6741435615,4.7314369025,8.3248851424\H,2.3962290664,
3.3246016956,7.5338341372\H,0.8510091024,6.6719735046,7.0189268411\H,2
.4007317091,6.8926192162,6.223458624\H,0.914009249,6.7223844172,5.2596
658676\C,2.2556321958,4.8573031622,3.0495386018\C,3.0149324108,3.98634
32618,2.0374463672\C,0.7540009007,4.7826525184,2.7537827084\C,2.739563
1966,6.301963997,2.8902264483\H,4.0883827838,3.9466038933,2.2543936216
\H,2.6283612815,2.9617096546,1.9996501229\H,2.8942952427,4.424362569,1
.0387652014\H,0.1755317877,5.4748886671,3.3730683017\H,0.5889998707,5.
0657261718,1.7071709781\H,0.3551075637,3.7705878772,2.8908358918\H,2.4
383680935,6.6521651615,1.8952900069\H,2.3030130832,6.9883479791,3.6202
538945\H,3.8288654175,6.3760554962,2.9426486831\C,4.3685035683,4.38720
20648,5.0931326474\C,5.3440885017,3.3670798782,5.1365792383\C,4.785772
5122,5.7194912463,5.2619785029\C,6.6882762013,3.7278688298,5.310521135
1\C,5.0923542958,1.9007623111,5.0494422511\C,6.1193787784,6.0538621482
,5.4550330571\H,4.0589422223,6.5214781039,5.2285078133\C,7.0807583749,
5.0497479391,5.4688021497\H,7.4320544224,2.9366520796,5.341127922\C,4.
6107306312,1.1946789742,6.1606455788\C,5.5107210833,1.1819849657,3.923
8115227\H,6.4018963639,7.0941807944,5.5857970029\H,8.130066646,5.29145

668,5.6103315668\C,4.552909993,-0.1973228104,6.1445053628\H,4.32989765
23,1.7405626518,7.0578774629\C,5.4429646434,-0.2100438753,3.9055333229
\H,5.9154007451,1.7188230166,3.0693796357\C,4.9676929973,-0.9028220665
,5.0166774747\H,4.2051610132,-0.7300506955,7.0249883846\H,5.7917097936
,-0.7555043094,3.0324491743\H,4.944616169,-1.9891078569,5.0115404877\\
Version=EM64L-G09RevA.01\State=1-A\HF=-1795.4096634\RMSD=4.756e-09\RMS
F=2.557e-06\Dipole=0.0274845,0.4029217,-0.3283401\Quadrupole=-4.617804
9,6.0655122,-1.4477073,9.2296413,-0.1752284,2.772039\PG=C01 [X(C34H41A
u1P1)]\\@

TS1

1\1\GINC-SHI_02\FTS\RPBE1PBE\GenECP\C34H41Au1P1(1+)\YIN\13-Jul-2015\0\
\#p opt=(calcfc,ts,noeigen) genecp pbe1pbe\\Title Card Required\\1,1\C
,2.0974561632,-3.1275037314,-1.3852536549\C,2.950278396,-2.0191255015,
-0.8180002263\C,2.5741618598,-3.233165749,0.0210731168\H,1.0485208571,
-2.9109258899,-1.5629810084\H,2.5650321163,-3.7586701758,-2.136807551\
H,3.3693060348,-3.9387489485,0.247100827\H,1.8590703176,-3.0749010657,
0.8223862311\C,2.3609878281,-0.7186208667,-0.4355397271\C,3.028547909,
0.3270784561,-0.0672768044\C,3.153579802,1.7406858089,0.1562564638\C,3
.313236656,2.2597792828,1.4525910902\C,3.1105088417,2.62142811,-0.9391
927343\C,3.409189245,3.63035478,1.6482600455\C,3.2088461192,3.99187503
02,-0.7363363696\H,2.996971478,2.2159446124,-1.9403648839\C,3.35765427
42,4.4963797826,0.5551387883\H,3.1747087844,4.66840314,-1.5851505143\H
,3.4394451373,5.5682770029,0.7109923519\C,4.3629479927,-1.8762780681,-
1.388863407\H,4.305506543,-1.7069080569,-2.4700431509\H,4.9089639322,-
2.8199122924,-1.2480074666\C,5.1057654565,-0.7541415866,-0.7476800824\
C,5.0206585733,-0.4916919856,0.578755376\H,5.6833662456,-0.0856718411,
-1.3831367026\H,5.5509668146,0.3482666846,1.0169653339\H,4.5452204771,
-1.1883004232,1.2644314002\Au,0.307820317,-0.277030939,-0.418062749\H,
3.3565765922,1.5765286449,2.2956214101\H,3.5285461935,4.0277601101,2.6
517977269\P,-1.9550026171,0.2343791669,-0.783414308\C,-3.0829889636,0.
1319943416,0.6650095242\C,-4.420492834,0.530554763,0.4925967607\C,-2.6
860843254,-0.3521649378,1.9297184719\C,-5.3501406643,0.454762956,1.520
7950824\H,-4.7517492671,0.9019464915,-0.4689213573\C,-3.643199645,-0.4
348751016,2.9518382234\C,-4.9595440928,-0.0384303036,2.7606351026\H,-6
.3734320246,0.775216691,1.3487843395\H,-3.3280100704,-0.8120230652,3.9
206539241\H,-5.6730414841,-0.1109704037,3.5762866493\C,-2.6147259694,-
1.0411210061,-2.0211273237\C,-1.81005947,-0.9300354158,-3.3210324895\C
,-2.3509744186,-2.4083309043,-1.3731926001\C,-4.1109531053,-0.95071229
11,-2.3358581316\H,-2.05344702,-0.0216692969,-3.8813879198\H,-0.728620
8335,-0.9479894744,-3.1398980362\H,-2.0571094939,-1.7836595799,-3.9642
620126\H,-2.8942446719,-2.5199932193,-0.4283906505\H,-2.6981223237,-3.
1966723916,-2.0526927599\H,-1.285557905,-2.5741116368,-1.1777901056\H,
-4.3483634609,-1.7169117,-3.084334496\H,-4.7268271971,-1.1569779276,-1

.4565342602\H,-4.4074000111,0.0138745075,-2.7558645989\C,-2.005276937,
2.0339462845,-1.406808299\C,-1.8846732909,2.9206440189,-0.1604128094\C
,-0.7731455873,2.2761276603,-2.2919390991\C,-3.2482000044,2.4323055581
,-2.2086198921\H,-2.7652511238,2.8528649013,0.4850338515\H,-0.99842493
26,2.6644378189,0.4332763873\H,-1.7781972872,3.9650691048,-0.478791184
7\H,-0.7579836552,1.6405037213,-3.1817825498\H,-0.7879471417,3.3199028
169,-2.6304116465\H,0.1590560928,2.1147205452,-1.740058377\H,-3.146567
4709,3.4859618255,-2.4970698331\H,-3.3493019518,1.8553957996,-3.132538
587\H,-4.1763523669,2.3520764384,-1.6384308922\C,-1.312232493,-0.78297
56933,2.3224353585\C,-0.3826659418,0.1551639423,2.7885369455\C,-1.0030
129361,-2.1444560165,2.419248961\C,0.8250965185,-0.2616027046,3.343569
2184\H,-0.6283088199,1.2135533211,2.7521416089\C,0.2081892977,-2.55940
97748,2.9692511973\H,-1.7301206981,-2.8819111647,2.0890302946\C,1.1233
873782,-1.6189694802,3.4375599691\H,1.5198678238,0.4783953956,3.731532
9371\H,0.4219048673,-3.6213485061,3.059097801\H,2.0541595721,-1.944275
398,3.894353483\\Version=EM64L-G09RevA.01\State=1-A\HF=-1795.3921256\R
MSD=4.540e-09\RMSF=1.104e-06\Dipole=1.1095237,0.5432801,-0.2446746\Qua
drupole=21.6636025,-6.6566853,-15.0069172,0.5301328,1.414571,-0.091380
9\PG=C01 [X(C34H41Au1P1)]\\@

IN2

1\1\GINC-SHI_02\FOpt\RPBE1PBE\GenECP\C34H41Au1P1(1+)\YIN\15-Jul-2015\0
\\#p opt genecp pbe1pbe\\Title Card Required\\1,1\C,-0.1211430953,0.18
63330348,-0.0339520303\C,0.2199108748,0.1160324905,1.4690479802\C,1.26
99592943,-0.0552281379,0.3404705102\H,-0.7147085136,-0.633273899,-0.42
70368217\H,-0.4122169438,1.1804117235,-0.3626824484\H,1.9803231992,0.7
647628179,0.2822553083\H,1.6890020435,-1.0499523815,0.2259223906\C,-0.
160308074,-1.0580104706,2.2121369109\C,-0.3186579549,-0.7028242691,3.6
066327343\C,-1.2111793538,-1.4722993588,4.523477195\C,-0.715207493,-2.
4944556424,5.3374942504\C,-2.5776675268,-1.1780695867,4.5587073554\C,-
1.5694757269,-3.2108582336,6.1715306316\C,-3.4320569675,-1.8904584837,
5.3959562986\H,-2.9704041471,-0.3789012411,3.9344214834\C,-2.929269841
1,-2.908518093,6.2031873696\H,-4.4899342919,-1.6449766819,5.4230719502
\H,-3.5939184014,-3.4586165355,6.8632014392\C,0.1221405317,1.372949716
6,2.335371081\H,-0.7491740592,1.9716796895,2.0470126373\H,1.0075737053
,2.0139068716,2.2534315273\C,-0.0413340146,0.8221363419,3.7332414485\C
,1.004014606,-0.1106917426,4.1700301655\H,-0.6002937347,1.3751430239,4
.4820462607\H,1.1162307395,-0.3009883893,5.2337931284\H,1.9077880676,-
0.2290267526,3.5779671088\Au,-0.5076794531,-2.9231973662,1.5004168755\
H,0.3473441012,-2.7257994445,5.3195308817\H,-1.1712903201,-3.997393011
7,6.8065650175\P,-1.2640740126,-5.0024391984,0.6300151252\C,0.00909753
73,-6.3108659204,0.4043870557\C,-0.4136794783,-7.579540387,-0.02868537
27\C,1.3936106745,-6.0869671209,0.5703012309\C,0.4847778074,-8.5999671
863,-0.3105198739\H,-1.4698390232,-7.7771400701,-0.1633931702\C,2.2870
577075,-7.122162891,0.2561719639\C,1.8487230843,-8.3648788784,-0.17933

30047\H,0.1178401803,-9.5679790801,-0.6385676931\H,3.3503929959,-6.940
6238819,0.3854824579\H,2.5684589841,-9.1465615054,-0.4041192042\C,-1.9
197979346,-4.6487591552,-1.1130072058\C,-3.0921228345,-3.6670874226,-1
.0048821018\C,-0.7597265133,-3.9686555067,-1.8559456463\C,-2.347901596
2,-5.8745219872,-1.9249073215\H,-3.9875833696,-4.1350729567,-0.5843334
372\H,-2.8394114257,-2.7914989978,-0.394728863\H,-3.3517933213,-3.3128
423939,-2.010190017\H,0.1177959892,-4.6207865151,-1.9268613511\H,-1.08
26146337,-3.7323781366,-2.8775923774\H,-0.4543531045,-3.0339066348,-1.
371472829\H,-2.7319890634,-5.5266670031,-2.8920430193\H,-1.5064083893,
-6.5411312036,-2.1319891577\H,-3.1452005053,-6.4508958539,-1.448864740
8\C,-2.5759531231,-5.6752273136,1.8330557952\C,-1.8082259792,-6.345324
5588,2.9800609609\C,-3.3543530771,-4.4822213913,2.4094480097\C,-3.5855
819975,-6.6620229291,1.2393402452\H,-1.2731312059,-7.2433812919,2.6575
431724\H,-1.0896451183,-5.6566109894,3.4406566432\H,-2.5237237139,-6.6
3798833,3.7583133283\H,-3.903670298,-3.9254557705,1.6449076762\H,-4.08
80652724,-4.8620271503,3.1315937073\H,-2.7013760525,-3.7816015942,2.94
07354697\H,-4.2752952962,-6.9668492772,2.0362524953\H,-4.1907954426,-6
.2119866025,0.4470997542\H,-3.1262698281,-7.5757627743,0.8549959901\C,
2.0298106323,-4.8474161663,1.0984564606\C,1.9622165464,-4.5336836686,2
.4633624639\C,2.8619211033,-4.0808038772,0.2734093346\C,2.7200895776,-
3.4879048589,2.9890167319\H,1.3553598607,-5.1495715639,3.1221455971\C,
3.611853624,-3.0305833779,0.7985147718\H,2.9402505868,-4.3302766066,-0
.7817757491\C,3.5483645049,-2.735792147,2.1591395341\H,2.6838143667,-3
.2823411657,4.0557099843\H,4.2697131724,-2.4612866479,0.1469814476\H,4
.1619609291,-1.9403549669,2.5738414419\\Version=EM64L-G09RevA.01\State
=1-A\HF=-1795.4407311\RMSD=9.478e-09\RMSF=2.618e-06\Dipole=0.1231742,0
.8609439,-0.1654766\Quadrupole=-7.7245501,15.8057793,-8.0812291,6.7538
079,-2.8313835,9.4342118\PG=C01 [X(C34H41Au1P1)]\\@

TS2

1\1\GINC-SHI_03\FTS\RPBE1PBE\GenECP\C34H41Au1P1(1+)\YIN\18-Jul-2015\0\
\#p opt=(calcfc,ts,noeigen) genecp pbe1pbe\\Title Card Required\\1,1\C
,-2.4430787195,-0.724928238,-2.4950075297\C,-1.9923516075,-2.162072863
4,-2.8278789747\H,-3.4627719704,-0.5068927484,-2.8073332518\H,-1.75425
93365,0.0773201032,-2.750122568\H,-0.9089686931,-2.2415208389,-2.89832
6585\H,-2.5103285146,-2.7052456602,-3.6168898214\C,-2.5677747281,-2.24
72748038,-1.4781687709\C,-3.8138419441,-2.9339245573,-1.0172730206\C,-
4.3526562179,-2.0193738446,0.0642525646\H,-3.5168287658,-3.9231726539,
-0.6355097922\H,-4.5123161443,-3.1259455275,-1.8423440368\C,-3.4116165
468,-0.8169930079,0.1909039189\C,-4.810626571,-0.6646128529,-0.3652804
105\H,-4.9626523235,-0.4618843862,-1.4230440532\H,-5.5186365045,-0.158
004293,0.2841319534\C,-2.2916104609,-1.0317774729,-0.8180108763\Au,-0.
3132176201,-0.5098459489,-0.2737747762\H,-4.8055332999,-2.464516275,0.
9441402471\C,-3.1067204148,-0.1778860184,1.5097966139\C,-3.1152857916,

-0.9100106893,2.6985469105\C,-2.8275565642,1.1929757911,1.5679281286\C
,-2.8548010137,-0.288919489,3.9195264514\H,-3.3293560721,-1.9755119095
,2.6735032679\C,-2.5601830795,1.8141466085,2.7836915318\H,-2.836750088
2,1.7745522267,0.6494380756\C,-2.5748372234,1.0732393641,3.9652450507\
H,-2.8756636128,-0.8726365359,4.8356919571\H,-2.3588838413,2.881805004
6,2.8137728122\H,-2.3805819078,1.5598722864,4.9168189889\P,1.895816467
3,-0.3074196644,0.4925994936\C,2.8875516488,-1.6992357357,-0.328534998
9\C,2.3024764078,-3.0491142514,0.1004972972\C,2.6838641799,-1.52358093
73,-1.8401918048\C,4.3926266444,-1.6727941499,-0.045943002\H,2.5125095
797,-3.2798141748,1.1492491262\H,1.2176406552,-3.0911286602,-0.0540432
456\H,2.7596110789,-3.8417726608,-0.5048358172\H,3.0635421262,-0.55919
26939,-2.1945443797\H,3.2358423499,-2.3125173544,-2.366268608\H,1.6266
771013,-1.6012346808,-2.119492953\H,4.8529154725,-2.5269819278,-0.5581
084303\H,4.8651738305,-0.7668517328,-0.4353934079\H,4.6327222741,-1.76
47617158,1.0161407091\C,1.8322480207,-0.4318514081,2.3930251723\C,1.46
81919448,0.9670934721,2.9070872977\C,0.690381162,-1.3904783334,2.76596
79639\C,3.1031058119,-0.932043463,3.088970489\H,2.2689863862,1.6948084
657,2.746848079\H,0.552267167,1.34333362,2.4354671721\H,1.2766549093,0
.9060595418,3.9854590864\H,0.8417315336,-2.401276727,2.3760037802\H,0.
6416528256,-1.4614600258,3.8598868792\H,-0.2805286881,-1.0261486993,2.
4160278574\H,2.9113524413,-0.9492945897,4.1690235855\H,3.3653946789,-1
.9526074758,2.7952065681\H,3.9735682693,-0.2900705675,2.9380324988\C,2
.8126580488,1.2301450992,0.0624135353\C,4.0614675542,1.4351207844,0.67
47234365\C,2.3751412256,2.1736374821,-0.8923575361\C,4.8677092299,2.52
2642308,0.3679034911\H,4.4237225208,0.7213004948,1.403190828\C,3.21310
67739,3.255634211,-1.2041878845\C,4.4424521412,3.4385637469,-0.5872447
907\H,5.8236352052,2.6452856088,0.868358812\H,2.8678080401,3.975622386
,-1.9408392834\H,5.0596264171,4.293334481,-0.8480643201\C,1.0763082436
,2.1704868361,-1.6249849229\C,-0.1055644883,2.5897604622,-1.0004141518
\C,1.0630299322,1.9404081882,-3.005775956\C,-1.2653419764,2.7930742181
,-1.7460363412\H,-0.099916925,2.8023238371,0.065616296\C,-0.0996336822
,2.1306630986,-3.7492000054\H,1.9810999282,1.6396495675,-3.5041093969\
C,-1.2650333195,2.5692559546,-3.1223465866\H,-2.1640929058,3.158030333
2,-1.2558602441\H,-0.0857459331,1.9665034184,-4.8233215713\H,-2.162041
7318,2.7578502441,-3.7062788217\\Version=EM64L-G09RevA.01\State=1-A\HF
=-1795.4141123\RMSD=9.905e-09\RMSF=3.769e-06\Dipole=-0.5439996,-1.0565
49,-0.9173233\Quadrupole=12.4608699,-9.0136281,-3.4472418,13.1571255,9
.751049,2.0460998\PG=C01 [X(C34H41Au1P1)]\\@

IN3

1\1\GINC-SHI_03\FOpt\RPBE1PBE\GenECP\C34H41Au1P1(1+)\YIN\06-Jul-2015\0
\\#p opt genecp pbe1pbe\\Title Card Required\\1,1\C,-0.0041407497,0.00
96827273,-0.0060723133\C,-0.0246565052,0.0207701259,1.554424828\H,1.01
23279777,0.0085245687,-0.4143869851\H,-0.5956846772,0.7633085858,-0.52

9746883\H,-0.6051486019,0.7959645148,2.0608075769\H,0.9845004577,0.004
9249389,1.9829682079\C,-0.6100674843,-1.3908385642,1.4745013318\C,-0.4
140431886,-2.7582843967,2.0912194724\C,-0.2226002564,-3.6297860674,0.8
450859832\H,-1.2442530316,-3.0847283463,2.7263160754\H,0.4847139036,-2
.7574616814,2.7223619516\C,-0.3451071554,-2.7884805385,-0.4415692297\C
,0.9884725293,-3.3378055117,0.0130564968\H,1.7381237242,-2.6520152427,
0.4056079174\H,1.3933361805,-4.1515281468,-0.5822130713\C,-0.585316133
4,-1.4104062561,0.0855122268\Au,-2.7477286313,-1.1314375116,0.72718938
54\H,-0.6011805794,-4.6476715382,0.8493797217\C,-0.9654459976,-3.28449
20256,-1.7060933567\C,-2.2151026196,-3.9101524127,-1.7014357268\C,-0.3
110780021,-3.0959778641,-2.9269767152\C,-2.8091385878,-4.3217984051,-2
.8926143852\H,-2.7180357747,-4.0897923246,-0.7531202193\C,-0.903079968
9,-3.5028729267,-4.1199181566\H,0.671193659,-2.6293599201,-2.938516425
7\C,-2.156633569,-4.1124416223,-4.1057934389\H,-3.7761651347,-4.817453
6814,-2.8737635798\H,-0.3820364172,-3.3520333846,-5.0612348381\H,-2.61
59622646,-4.4365288816,-5.0351758689\P,-4.9161127216,-1.0080746634,1.6
683993923\C,-4.7578680515,0.0245967397,3.2503820086\C,-3.8347442758,-0
.7005582591,4.2354934539\C,-4.0979243479,1.3417563009,2.8173379061\C,-
6.0859128293,0.3564573278,3.9372002079\H,-4.2959698763,-1.6000482344,4
.6549425309\H,-2.8787081388,-0.9781187471,3.7762315864\H,-3.6172125024
,-0.0276656228,5.0739652006\H,-4.7015269944,1.8800049924,2.0783765674\
H,-3.9912917489,1.9896219665,3.6961028029\H,-3.0995020962,1.1803219668
,2.3947588542\H,-5.866284816,0.9306797414,4.8457209526\H,-6.7284858035
,0.9782854374,3.3084146501\H,-6.6446979789,-0.5314158981,4.2433702524\
C,-5.5282014141,-2.787480992,1.9519270837\C,-6.0643138083,-3.287324816
3,0.6040494931\C,-4.3225204005,-3.6549539656,2.3439925384\C,-6.6025345
18,-2.9598606404,3.0317961209\H,-6.9648602121,-2.7534553793,0.28796041
87\H,-5.3137358196,-3.1965388811,-0.190575184\H,-6.3169283139,-4.35064
36967,0.6974027672\H,-3.8642421901,-3.3396752711,3.2858065237\H,-4.661
6802005,-4.6903989048,2.4717845618\H,-3.5498258217,-3.6478932587,1.567
1567335\H,-6.868014483,-4.0231542109,3.08193122\H,-6.2468439122,-2.671
6246896,4.0252387788\H,-7.52582616,-2.4164803257,2.8182995157\C,-6.165
8667477,-0.1312020154,0.6491448334\C,-7.4990563738,-0.1226069656,1.093
7834907\C,-5.8365170418,0.6103411245,-0.5075024535\C,-8.4889586361,0.5
966511972,0.437631613\H,-7.7746483038,-0.6769728111,1.9826365031\C,-6.
8466427327,1.3500104728,-1.1414349407\C,-8.1567563378,1.348646078,-0.6
837051309\H,-9.5094066483,0.5742192509,0.8081867443\H,-6.5876624487,1.
9145437028,-2.0326868946\H,-8.9146222859,1.9244234382,-1.2067528961\C,
-4.504071265,0.6612443027,-1.1657646796\C,-3.9575460389,-0.4789466167,
-1.7802307595\C,-3.8567676256,1.8906241617,-1.3363921903\C,-2.80988008
92,-0.379423663,-2.5708863727\H,-4.489156778,-1.4255717835,-1.72261721
43\C,-2.7049845486,1.9854415732,-2.1147232461\H,-4.2757492373,2.781422
3454,-0.8752039793\C,-2.1853585526,0.8541636041,-2.7409011684\H,-2.427
6002085,-1.2640089853,-3.0708361353\H,-2.2264040626,2.9517070072,-2.24

92318335\H,-1.3051519313,0.9350925821,-3.3725158875\\Version=EM64L-G09
RevA.01\State=1-A\HF=-1795.4439289\RMSD=8.412e-09\RMSF=3.071e-06\Dipol
e=-0.2348046,0.2514453,0.722679\Quadrupole=13.4373895,-10.1252949,-3.3
120946,-4.6444734,-7.998228,-0.614177\PG=C01 [X(C34H41Au1P1)]\\@

TS3

1\1\GINC-SHI_02\FTS\RPBE1PBE\GenECP\C34H41Au1P1(1+)\YIN\17-Aug-2015\0\
\#p opt=(calcfc,ts,noeigen) genecp pbe1pbe\\Title Card Required\\1,1\C
,4.2257069027,-1.7441887666,-1.5074974085\C,3.8609096293,-1.4106999031
,-2.9984735986\H,5.2900138168,-1.651770656,-1.2660506798\H,3.86789294,
-2.7198886603,-1.1604591038\H,3.302801311,-2.1906096462,-3.5286052374\
H,4.7212816904,-1.1173525373,-3.6098599434\C,3.0455872553,-0.275976551
2,-2.4221843882\C,2.1976034165,0.92935002,-2.6701715558\C,2.0590109507
,1.54837692,-1.2824685252\H,1.2263024059,0.6997084205,-3.1271164506\H,
2.7025540318,1.6505006712,-3.3319402809\C,2.9627047748,0.4431054081,-0
.1189795353\C,3.3451125557,1.8145417087,-0.5647897975\H,4.2438384534,1
.8745736135,-1.180142375\H,3.2935896738,2.5999380536,0.1868671754\C,3.
3651859808,-0.5435781275,-1.1524086768\Au,0.4725237624,0.1228228547,-0
.2585468532\H,1.2667745326,2.2746376557,-1.0794834486\C,2.8133225163,0
.1086722265,1.3290221011\C,3.030039796,-1.2121911928,1.7492924811\C,2.
5587859973,1.0857422061,2.3037975123\C,3.0042737184,-1.5419752095,3.10
08965161\H,3.2421825179,-1.9814140102,1.0133915008\C,2.5172154859,0.74
93952627,3.651258815\H,2.3803195628,2.1155181543,2.0099481727\C,2.7435
066635,-0.5650681771,4.057426838\H,3.195850098,-2.5670591597,3.4053927
266\H,2.3208781401,1.520947593,4.3904741389\H,2.7265781075,-0.82158505
56,5.1125353319\P,-1.4998944625,-1.0630223363,-0.0121036087\C,-1.41932
24025,-2.0630305295,1.5957022813\C,-0.2901200003,-3.0913895329,1.48078
56386\C,-1.0751934624,-1.0508639818,2.6976167968\C,-2.7164619461,-2.77
50432924,1.9948170225\H,-0.5357230658,-3.9005766892,0.7860540833\H,0.6
542673132,-2.6305889591,1.1724155468\H,-0.129171156,-3.5453283679,2.46
62393986\H,-1.8523408213,-0.2845152194,2.7981832038\H,-1.012384628,-1.
5819460366,3.6554315087\H,-0.1117296528,-0.558732605,2.5251429464\H,-2
.5177026951,-3.3487644015,2.9084312799\H,-3.5180510281,-2.0686519211,2
.226303812\H,-3.0728725037,-3.4837519601,1.2421112781\C,-1.6753551169,
-2.1105495758,-1.5933906344\C,-2.155726428,-1.1495737025,-2.6893067367
\C,-0.2890199412,-2.6414864337,-1.9894023428\C,-2.6339006633,-3.301394
0166,-1.496073411\H,-3.1845816889,-0.8159270731,-2.5271745216\H,-1.514
0735397,-0.2622515707,-2.7610008612\H,-2.1162836901,-1.6684312663,-3.6
549041701\H,0.145486652,-3.3016826201,-1.2341349907\H,-0.3922857576,-3
.2210637663,-2.9153104203\H,0.4205244308,-1.8281399059,-2.1781578006\H
,-2.3121287281,-4.0386176034,-0.7547714064\H,-3.6640951262,-3.00809068
2,-1.2824453771\H,-2.6472999797,-3.8086670185,-2.4685816644\C,-3.01171
23527,-0.0201096297,0.082931918\C,-4.2478554302,-0.6905107826,0.112802
632\C,-3.0175990059,1.3922622615,0.0350298885\C,-5.4589445889,-0.01255

79742,0.092951405\H,-4.271443152,-1.7718616123,0.1471323476\C,-4.25323
32084,2.0574172066,0.004734634\C,-5.4616213879,1.375904937,0.033339231
6\H,-6.3907138503,-0.5696135553,0.1190742049\H,-4.2494138569,3.1432725
498,-0.0272778536\H,-6.3968830125,1.9275734331,0.0132152314\C,-1.83427
81688,2.3002553014,0.0242124254\C,-1.5902394121,3.0934376133,-1.105168
9019\C,-1.0819852358,2.5342830048,1.183586466\C,-0.6197980699,4.092599
7449,-1.0793985225\H,-2.1848669585,2.9375983094,-2.0018015819\C,-0.121
4485552,3.5443812211,1.2131874588\H,-1.2846047998,1.9574097783,2.08139
95593\C,0.1120544924,4.3277526391,0.0843305898\H,-0.4551117151,4.70645
00305,-1.9607405187\H,0.4212271698,3.7396030604,2.1342786449\H,0.84377
64992,5.1303064151,0.1163694394\\Version=EM64L-G09RevA.01\State=1-A\HF
=-1795.4160561\RMSD=5.442e-09\RMSF=1.378e-06\Dipole=-0.1963372,-0.1863
621,-0.1926354\Quadrupole=9.3402799,-1.6981079,-7.642172,3.5497286,-1.
0388844,-2.2832609\PG=C01 [X(C34H41Au1P1)]\\@

IN4

1\1\GINC-SHI_02\FOpt\RPBE1PBE\GenECP\C34H41Au1P1(1+)\YIN\07-Jul-2015\0
\\#p opt genecp pbe1pbe\\Title Card Required\\1,1\C,-1.148893854,-0.35
95984171,-2.5853880193\C,-1.5140592492,0.7062177284,-1.5022731201\H,-0
.1835676689,-0.2076360431,-3.078967918\H,-1.9233872713,-0.5035477839,-
3.3457757607\H,-2.4839344851,1.2052891971,-1.6199032169\H,-0.755039784
6,1.4784429588,-1.328199879\C,-1.5071052922,-0.4119409425,-0.503971545
\C,-1.7141160622,-0.6452004364,0.9254581109\C,-2.00284441,-2.142148511
7,1.1251043061\H,-2.4853184111,0.0109056588,1.3445669265\H,-0.76462916
46,-0.3404914975,1.4213219371\C,-0.8349784368,-2.6798556396,-1.0956287
417\C,-0.9417665224,-2.9688145869,0.3770029506\H,0.0736582182,-2.76220
47008,0.782076041\H,-1.118643124,-4.0346039948,0.5402448503\C,-1.15803
83433,-1.362718194,-1.4431240339\Au,-3.9670946896,-2.6731532376,0.6102
710811\H,-1.8944693963,-2.3730365643,2.1912219732\C,-0.3449140587,-3.6
557331431,-2.035613005\C,-0.4150651013,-3.4162732433,-3.4278665322\C,0
.2282266097,-4.8688726712,-1.5913985392\C,0.0577289901,-4.3521450975,-
4.3313961931\H,-0.8658172808,-2.5023087067,-3.7963098722\C,0.718758511
3,-5.7920542643,-2.4987878703\H,0.3112305616,-5.0742446072,-0.52983468
85\C,0.6305185984,-5.5384037575,-3.8690158818\H,-0.0138256613,-4.16144
80362,-5.3976734874\H,1.1704701231,-6.7130903773,-2.1438572267\H,1.009
8448141,-6.2679224684,-4.5789481546\P,-6.1279626377,-3.3853492783,-0.0
482431346\C,-6.1333594692,-5.282260651,-0.0320262823\C,-5.1046827574,-
5.7826303008,-1.0525768952\C,-5.6662612871,-5.6718759858,1.3784460269\
C,-7.4795834009,-5.963354985,-0.2896667704\H,-5.4433909423,-5.64299344
45,-2.0844011922\H,-4.1357753381,-5.283812907,-0.9310960701\H,-4.95200
98645,-6.859102444,-0.9037819556\H,-6.3629368546,-5.3161454065,2.14584
32681\H,-5.6237022128,-6.7661345919,1.4511509516\H,-4.6698462007,-5.27
43370856,1.603808848\H,-7.3208758881,-7.049192051,-0.2888613822\H,-8.2
060479086,-5.742219483,0.4968539077\H,-7.9170848759,-5.7008946142,-1.2

566303651\C,-6.4819498545,-2.6301548183,-1.7625646675\C,-6.8621970953,
-1.1662086367,-1.5008669607\C,-5.1793845082,-2.6463286766,-2.578400154
9\C,-7.5729768229,-3.3019363212,-2.6011189197\H,-7.827572287,-1.073346
6365,-0.9948206193\H,-6.1050165387,-0.6572834565,-0.8910372453\H,-6.93
11937648,-0.6382044819,-2.460399269\H,-4.8274405313,-3.6599195894,-2.7
894066315\H,-5.3655742529,-2.1522750759,-3.5412558235\H,-4.374180975,-
2.1098327801,-2.0637724877\H,-7.3304952861,-4.3401337757,-2.8478903892
\H,-8.5568667705,-3.2725454157,-2.1277535644\H,-7.6646027867,-2.758702
4876,-3.5504879455\C,-7.5382311358,-2.8756516214,1.0242505364\C,-8.845
7415126,-3.2056207951,0.6251771542\C,-7.3728858699,-2.1272525033,2.210
2242541\C,-9.9618118775,-2.8162116762,1.3537995147\H,-9.0022706424,-3.
7830836893,-0.2770004862\C,-8.5121816065,-1.7394910488,2.9310324027\C,
-9.7940004674,-2.0740452343,2.5172117923\H,-10.954597121,-3.0923889037
,1.0106337135\H,-8.3717503286,-1.1651094898,3.8424848058\H,-10.6538511
582,-1.7588010987,3.1012374835\C,-6.0762484117,-1.6895499817,2.8062331
36\C,-5.6620320061,-0.3589252169,2.67225439\C,-5.3537844492,-2.5376367
619,3.6533282452\C,-4.5467326931,0.1096989426,3.3631561135\H,-6.236016
9394,0.3156137131,2.0416455306\C,-4.2387301012,-2.0687686638,4.3440605
748\H,-5.6850544252,-3.5628973352,3.7923992892\C,-3.833226241,-0.74354
75827,4.2028294397\H,-4.2537688934,1.1524840626,3.2683692417\H,-3.6996
805405,-2.7362561817,5.0109917633\H,-2.978471861,-0.3717975502,4.76147
31967\\Version=EM64L-G09RevA.01\State=1-A\HF=-1795.4355912\RMSD=9.827e
-09\RMSF=2.953e-06\Dipole=2.4007959,0.0806553,-2.1561057\Quadrupole=19
.2393399,-11.2374292,-8.0019107,-1.5437428,-20.1174915,6.7744738\PG=C0
1 [X(C34H41Au1P1)]\\@

TS5

1\1\GINC-SHI_02\FTS\RPBE1PBE\GenECP\C34H41Au1P1(1+)\YIN\20-Aug-2015\0\
\#p opt=(calcfc,ts,noeigen) genecp pbe1pbe\\Title Card Required\\1,1\C
,4.5631023786,-1.1028034089,-2.2259309612\C,3.9595851438,-0.4070151063
,-3.49479721\H,5.6440071328,-0.9911634504,-2.0949280496\H,4.3099029068
,-2.1671189362,-2.1638525684\H,3.4776260288,-1.0591442799,-4.228855687
1\H,4.6602686551,0.2524217668,-4.0160519397\C,3.0489119437,0.355900648
7,-2.549924065\C,1.5442197283,1.635690249,-1.1091283293\H,1.7718467058
,-0.0149307228,-2.6415945392\H,1.4988176767,1.6754823212,-3.3548145765
\C,1.9601196204,1.2469178829,-2.4650868583\C,3.5761734156,0.3783136093
,-0.1504696731\C,2.6902624041,1.6195332694,-0.0809108893\H,3.351100306
2,2.4966805388,-0.2071211269\H,2.2594007341,1.7123613248,0.9192834353\
C,3.6976875392,-0.1805317018,-1.3831734068\Au,-0.0853700153,0.38088435
4,-0.5583873896\H,1.0557022519,2.6154429765,-1.1342454814\C,4.35086800
06,-0.0432953909,1.0129982761\C,4.9469269523,-1.3176650708,1.056893657
5\C,4.5397914177,0.8193292818,2.1066456115\C,5.7069746742,-1.709570502
6,2.1488196078\H,4.7887838669,-2.0130472793,0.2384047078\C,5.312623615
1,0.4293076432,3.1930602331\H,4.1001835979,1.8118260968,2.1002581774\C

,5.8967956229,-0.8350102484,3.2187457039\H,6.1521511755,-2.7000126706,
2.1696293991\H,5.4595638531,1.1139262163,4.0232162504\H,6.4955024721,-
1.1408782704,4.0718317532\P,-1.8058222468,-1.1173023513,0.0229106061\C
,-1.1708893089,-2.2279239617,1.4201105355\C,0.0160937784,-3.0438696749
,0.895225256\C,-0.6714006934,-1.2734641238,2.5141900162\C,-2.204484900
7,-3.1668983046,2.0497106739\H,-0.2941719021,-3.8176152442,0.186048554
9\H,0.7674303753,-2.4055103487,0.4147382331\H,0.4977946335,-3.55125004
37,1.7403562018\H,-1.4828545461,-0.6537382506,2.911286321\H,-0.2707894
992,-1.8666932138,3.3457402257\H,0.1276939901,-0.6166445131,2.15141983
62\H,-1.6991057232,-3.7669197425,2.8168290182\H,-3.0061357481,-2.61473
40132,2.5478252277\H,-2.6484532049,-3.8649233988,1.3351168068\C,-2.306
3528974,-2.0676564633,-1.5506718787\C,-3.1915041477,-1.1117645938,-2.3
616441061\C,-1.0372751405,-2.3536319622,-2.3683474135\C,-3.0411642388,
-3.3939334183,-1.332876913\H,-4.1596786866,-0.9372455312,-1.8839009162
\H,-2.70322145,-0.1411136059,-2.5134775667\H,-3.375410175,-1.551083411
4,-3.3499615156\H,-0.3218629294,-2.9857747589,-1.8350791362\H,-1.32393
21137,-2.8766520883,-3.2894716444\H,-0.5298320204,-1.4243131089,-2.650
2131707\H,-2.4224450062,-4.1321711966,-0.8142017253\H,-3.9826556935,-3
.2798304013,-0.7906271571\H,-3.2912420156,-3.8156521104,-2.3146000675\
C,-3.336246196,-0.3122743163,0.6510891562\C,-4.4543343496,-1.119898601
,0.9223761028\C,-3.4651257377,1.0858595721,0.806705043\C,-5.6699911048
,-0.5849605437,1.3269926665\H,-4.379187211,-2.1942259639,0.8111520744\
C,-4.7040611211,1.6077924605,1.2069954783\C,-5.7973481079,0.7925030165
,1.4657536579\H,-6.5106929588,-1.2426648756,1.5271642975\H,-4.79424403
03,2.6840900121,1.3245542405\H,-6.740661909,1.2316683654,1.7771238926\
C,-2.393153054,2.1037276067,0.5993750214\C,-2.446537477,2.9591277303,-
0.5085784959\C,-1.4338066056,2.3489597074,1.5917115366\C,-1.5589251078
,4.0266738118,-0.6258619642\H,-3.2041962858,2.7955207701,-1.2707809715
\C,-0.5525151955,3.422086957,1.4778362222\H,-1.4062064541,1.7186992738
,2.4760909887\C,-0.613622771,4.2653089322,0.3700253155\H,-1.6250973597
,4.6887631963,-1.4850605482\H,0.1651043821,3.6140873795,2.2711912472\H
,0.0569663608,5.1169502036,0.2936456746\\Version=EM64L-G09RevA.01\Stat
e=1-A\HF=-1795.4030027\RMSD=4.649e-09\RMSF=1.570e-06\Dipole=1.1604684,
-0.5761388,-0.9640342\Quadrupole=20.5000117,-12.1209607,-8.379051,0.76
49869,-4.4577269,-0.4899213\PG=C01 [X(C34H41Au1P1)]\\@

IN6

1\1\GINC-SHI_02\FOpt\RPBE1PBE\GenECP\C34H41Au1P1(1+)\YIN\14-Jul-2015\0
\\#p genecp pbe1pbe opt\\Title Card Required\\1,1\C,0.0801028939,-0.23
20276978,-0.6644096494\C,-0.3343665515,-0.054784849,0.8258096107\H,1.1
270909865,-0.4530190461,-0.8893031582\H,-0.2427143031,0.6254339121,-1.
2679097774\H,-0.46538059,0.9639484714,1.2012553327\H,0.3327366655,-0.6
127803185,1.4894545529\C,-1.6277680067,-0.8637285274,0.5039248167\C,-2
.2361378716,-3.2323972912,0.9339968653\H,-2.2888533104,-1.7331904043,2

.4661741612\C,-2.1604450833,-1.9370174031,1.4018337635\C,-0.8482596367
,-2.6602026242,-1.1161403224\C,-1.7507930135,-3.6672114726,-0.43190033
13\H,-1.2085581009,-4.6097293299,-0.285437972\H,-2.5991178764,-3.94006
41533,-1.0768124529\C,-0.8870453865,-1.3895815724,-0.6946662819\Au,-4.
3551391064,-2.3058729347,0.867378997\H,-2.4435392878,-4.0306967901,1.6
48255684\C,0.0781373682,-3.1197204815,-2.1706946123\C,0.381429783,-2.2
690527965,-3.2451749449\C,0.6860033666,-4.3830346866,-2.1386195987\C,1
.2699255293,-2.6590030146,-4.2403226837\H,-0.1080734423,-1.3008549521,
-3.3071498029\C,1.5743831392,-4.7737452648,-3.1348202888\H,0.491585405
3,-5.0679276882,-1.3175794322\C,1.8710032713,-3.9142710672,-4.18931297
45\H,1.4849696461,-1.9844293656,-5.0644270574\H,2.0404033779,-5.753823
9345,-3.0837686844\H,2.5616824089,-4.2229720675,-4.96866118\P,-6.58270
71727,-2.3015260277,0.1570400171\C,-7.2952479222,-4.0349750658,0.42991
66955\C,-6.540936174,-5.0280489392,-0.4602694615\C,-7.0320147505,-4.37
02532386,1.9042711255\C,-8.8013200128,-4.1560378462,0.1761020825\H,-6.
7830633636,-4.9005241052,-1.5196121499\H,-5.4540528817,-4.9514024392,-
0.337747278\H,-6.8357278307,-6.0464378704,-0.1797128284\H,-7.552154807
8,-3.6783057186,2.575602581\H,-7.4117530958,-5.3783315183,2.1108218573
\H,-5.96320664,-4.3565138168,2.1476150135\H,-9.0887167233,-5.203526457
2,0.3287129698\H,-9.383748124,-3.5558017482,0.8802983342\H,-9.09099836
69,-3.8873341243,-0.8431360524\C,-6.5640293392,-1.7211458622,-1.653098
6114\C,-6.3643761822,-0.2001820097,-1.6145735842\C,-5.3529690086,-2.35
02705204,-2.3593054302\C,-7.8215691605,-2.056422648,-2.461119044\H,-7.
2302796351,0.323298016,-1.1997181513\H,-5.4789983895,0.0801240491,-1.0
302049281\H,-6.2103207809,0.1586912964,-2.6394002847\H,-5.4011717785,-
3.4418842364,-2.3962913339\H,-5.3271877622,-1.9870296388,-3.3939843423
\H,-4.4079912451,-2.0632241373,-1.8839292406\H,-7.9806370231,-3.134361
1122,-2.5567134773\H,-8.7272433448,-1.5985294253,-2.0561291826\H,-7.69
31849733,-1.6586450818,-3.4751422545\C,-7.6321722809,-1.1254564624,1.0
940588174\C,-8.960833353,-0.9444405335,0.6707729263\C,-7.1519809329,-0
.3438782991,2.1678776585\C,-9.8022098067,-0.0180272361,1.2715794438\H,
-9.3520145075,-1.5358967383,-0.1480194768\C,-8.0159640256,0.5930640577
,2.7538128722\C,-9.3233818606,0.7615369824,2.3185352464\H,-10.82305768
01,0.0938579202,0.9188003301\H,-7.6427753564,1.1903725936,3.5808985878
\H,-9.9645141425,1.4947338623,2.7989640295\C,-5.7958401501,-0.41265074
11,2.7904693999\C,-4.8914040662,0.6444792884,2.6106279416\C,-5.4904818
881,-1.4033855981,3.7361964371\C,-3.7207510069,0.7146781561,3.36334495
61\H,-5.1306190154,1.4330171758,1.9013154669\C,-4.3214161474,-1.329270
6158,4.4913436217\H,-6.1988657292,-2.2071550644,3.9156205033\C,-3.4379
537541,-0.2654494056,4.3133193921\H,-3.0429276951,1.5528717549,3.22653
98731\H,-4.1175789446,-2.0885242131,5.2415160468\H,-2.5428190167,-0.19
11580977,4.9248176883\H,-2.4176010802,-0.150061291,0.2248687407\\Versi
on=EM64L-G09RevA.01\State=1-A\HF=-1795.4702169\RMSD=9.930e-09\RMSF=2.3
90e-06\Dipole=-1.3075906,0.1108057,0.4113822\Quadrupole=17.8452127,-9.

4973542,-8.3478586,-4.203374,-4.8169983,5.0180888\PG=C01 [X(C34H41Au1P

TS4

1\1\GINC-SHI_02\FTS\RPBE1PBE\GenECP\C34H41Au1P1(1+)\YIN\06-Jul-2015\0\
\#p opt=(calcfc,ts,noeigen) genecp pbe1pbe\\Title Card Required\\1,1\C
,2.7802338488,0.0492661916,-1.582896486\C,2.5492578805,-1.3960817743,-
1.1080340142\H,1.8914385522,0.4786834247,-2.0381540514\H,3.7181172907,
0.2843662038,-2.0795913336\H,3.4340519565,-2.0196364458,-0.9925712189\
H,1.6981073901,-1.8964256933,-1.5557807344\C,1.7781271459,-0.824240229
1,0.4042910192\C,2.3157579733,-1.4797153505,1.6747872317\C,3.707103004
8,-0.9073682538,1.8923729803\H,2.3225441155,-2.5701772633,1.5573593722
\H,1.6531764052,-1.2677258787,2.5217514665\C,4.0575966718,-0.022674832
,0.6947585661\C,3.7865326756,0.5653575574,2.0741592674\H,2.8673502699,
1.1268462447,2.2245033991\H,4.6638270969,0.968819947,2.5708843752\C,2.
8212181015,-0.0137186234,-0.12419388\Au,-0.2690301131,-0.6340700111,0.
1129260959\H,4.4879180272,-1.5222264044,2.3299853945\C,5.4021479559,0.
0703398504,0.056586172\C,6.1815809594,-1.0730711305,-0.1405661479\C,5.
8878915089,1.3069719721,-0.3829472842\C,7.4242259998,-0.9834609223,-0.
7628735303\H,5.8209600709,-2.0374452447,0.2107686843\C,7.1266415499,1.
396506822,-1.0098832834\H,5.293855012,2.2037102125,-0.220233011\C,7.89
68142197,0.2507068915,-1.2004340742\H,8.0245591258,-1.8779010143,-0.90
21660876\H,7.4959612879,2.3624622655,-1.3422808563\H,8.8670500613,0.32
17902838,-1.683358377\P,-2.6127875966,-0.7065181631,-0.1277616023\C,-3
.2812824008,-1.610706512,1.3992358888\C,-2.7218215485,-3.0380335602,1.
4048658392\C,-2.7188219496,-0.8455018211,2.6062699984\C,-4.8055405854,
-1.6589826009,1.5410795113\H,-3.1789533365,-3.6654425006,0.6331961326\
H,-1.6338515996,-3.0537698912,1.2686861388\H,-2.9446997084,-3.50177181
,2.373776936\H,-3.074496025,0.1906999819,2.6301513645\H,-3.0603611991,
-1.3341398864,3.5273095669\H,-1.6225487328,-0.8380095283,2.6129085276\
H,-5.0455787097,-2.2431617306,2.4382712669\H,-5.235487789,-0.663488530
7,1.6812747674\H,-5.304894397,-2.1446892947,0.6985252072\C,-2.98607733
48,-1.586766005,-1.775162951\C,-2.8112159793,-0.5314033139,-2.87539507
31\C,-1.9339119767,-2.6852790279,-1.9891015124\C,-4.3732801378,-2.2249
036189,-1.8891711746\H,-3.5897801639,0.235839382,-2.8403312826\H,-1.83
53268077,-0.0342250864,-2.8090698427\H,-2.8663815408,-1.0264978899,-3.
8527286133\H,-1.9556691571,-3.4523286434,-1.2105610733\H,-2.1312863222
,-3.1789708604,-2.9489494574\H,-0.921147259,-2.268657179,-2.0265752945
\H,-4.4630990289,-2.6818884232,-2.8825790825\H,-4.5262213888,-3.020669
8043,-1.1542585087\H,-5.1866629184,-1.5009135167,-1.8012347797\C,-3.48
53268784,0.9105188358,-0.1988997784\C,-4.8766567052,0.9089182359,-0.40
28867107\C,-2.8218945821,2.153614769,-0.117863056\C,-5.6043920885,2.08
46284308,-0.5279293632\H,-5.4101989124,-0.030961274,-0.4677940109\C,-3
.5730099798,3.3304205325,-0.2531793008\C,-4.9461108779,3.3069960968,-0
.4554555239\H,-6.6782569248,2.0415825928,-0.6838926327\H,-3.0534707794

,4.2823594372,-0.188389584\H,-5.4967626699,4.238015255,-0.5534099698\C
,-1.3622025325,2.3650574703,0.1102423315\C,-0.5384638506,2.7470267331,
-0.9560130201\C,-0.8351870018,2.3789105032,1.4073806421\C,0.7810077676
,3.1328475794,-0.7311752418\H,-0.9492575531,2.7709284269,-1.9623987541
\C,0.482488659,2.7718984377,1.6324485182\H,-1.4746190837,2.1191948091,
2.2465946678\C,1.2929147457,3.152967427,0.564747675\H,1.3986341182,3.4
511716387,-1.5671499523\H,0.8632907545,2.8114350415,2.6499599258\H,2.3
104829238,3.4900370609,0.7447555874\\Version=EM64L-G09RevA.01\State=1-
A\HF=-1795.4231121\RMSD=3.312e-09\RMSF=1.392e-06\Dipole=0.6140127,-0.3
882497,-0.3978959\Quadrupole=37.4245491,-14.6412609,-22.7832882,-0.488
2669,-1.2764354,-1.117762\PG=C01 [X(C34H41Au1P1)]\\@

IN5

1\1\GINC-SHI_02\FOpt\RPBE1PBE\GenECP\C34H41Au1P1(1+)\YIN\07-Jul-2015\0
\\#p opt genecp pbe1pbe\\Title Card Required\\1,1\C,0.2801928019,0.634
7518187,-0.0667351959\C,0.467800033,0.9389914536,1.3848248503\C,1.8998
183197,0.600730102,1.7443409924\H,-0.2665758585,0.326657653,1.93527293
94\H,0.1769002397,1.978119469,1.5981099435\C,2.5388142229,-0.054324847
3,0.5340973777\C,2.966621525,1.3207883719,0.9734554723\H,2.6801058485,
2.174255645,0.3604951782\H,3.9407213182,1.4054940447,1.4464774836\Au,-
1.4308968492,1.0615999721,-1.0574806784\H,2.1290884566,0.2498450072,2.
7457459714\C,3.4775957543,-1.2117917671,0.6003239901\C,3.069595518,-2.
4311637152,1.1543236311\C,4.7695014788,-1.1080646452,0.0764645306\C,3.
9341517476,-3.5208720297,1.1832224591\H,2.0710949078,-2.5188073883,1.5
77923949\C,5.635973784,-2.1981852337,0.1035372766\H,5.1010061658,-0.16
0255092,-0.3418935672\C,5.2192191733,-3.4062583483,0.6556726676\H,3.60
84133694,-4.458527166,1.6247361963\H,6.6391756019,-2.1015655649,-0.301
8856083\H,5.8960116101,-4.2554074064,0.681967374\P,-3.5685695104,1.324
3446107,-2.0642941557\C,-4.8393005329,1.4972352668,-0.6701933334\C,-4.
8518542323,0.2022631844,0.1505614927\C,-4.3317324832,2.6458131792,0.21
30244554\C,-6.2651771795,1.8366551929,-1.1138811272\H,-5.319398081,-0.
6267109272,-0.3897139841\H,-3.8437490549,-0.1031033883,0.455391717\H,-
5.4400823453,0.3677117169,1.0614619748\H,-4.297916838,3.5931915564,-0.
3360966237\H,-5.0218699795,2.7768763284,1.0556756867\H,-3.3351415129,2
.4417260518,0.6217659643\H,-6.9020495537,1.8715522852,-0.2212539622\H,
-6.3233784639,2.8192703792,-1.5900646217\H,-6.6954458418,1.091633493,-
1.7879294732\C,-3.8518139691,-0.1801763337,-3.1967163682\C,-3.02767831
26,0.0868842071,-4.4635828911\C,-3.2813593324,-1.4275233799,-2.5042529
13\C,-5.3057264163,-0.4713023218,-3.5779317557\H,-3.4346991592,0.91048
61953,-5.0571857228\H,-1.9817493937,0.3188137601,-4.2260591674\H,-3.03
42696358,-0.8156794987,-5.0869631566\H,-3.7967203195,-1.6714091404,-1.
5715158655\H,-3.3959616733,-2.2844897648,-3.1795954864\H,-2.2146367727
,-1.3145058799,-2.282271788\H,-5.3180505444,-1.3444971376,-4.242131015
8\H,-5.9217587339,-0.7209856444,-2.7090833033\H,-5.780019844,0.3461023

837,-4.1258383198\C,-3.7455961227,2.8159788273,-3.1236271746\C,-4.9464
169381,2.9915457883,-3.8331277958\C,-2.7100403987,3.7575674121,-3.3129
55868\C,-5.134295975,4.0506288102,-4.7111120597\H,-5.7568708919,2.2855
387414,-3.7025648378\C,-2.9153708343,4.8147058521,-4.2114951364\C,-4.1
077309592,4.9676577452,-4.9059033039\H,-6.0768309135,4.1529920983,-5.2
406742217\H,-2.1142579279,5.5353356552,-4.3501816274\H,-4.2338694836,5
.8008132288,-5.5910934378\C,-1.3836774905,3.7606247833,-2.6294404721\C
,-0.2257573462,3.4424608978,-3.3516798766\C,-1.2469765049,4.2570423489
,-1.3257944183\C,1.035464977,3.6161397277,-2.7850330349\H,-0.319241004
2,3.0874631666,-4.3751049154\C,0.0153875716,4.4345214797,-0.7614445739
\H,-2.1329279572,4.5489171971,-0.7690649549\C,1.1594205266,4.118905463
,-1.4912913873\H,1.9236524346,3.3894767186,-3.3690695192\H,0.102636332
3,4.8515197038,0.2384690973\H,2.1439739407,4.2911407788,-1.0647061059\
C,1.4543796702,-0.0140904143,-0.5295696914\C,1.2079669115,-1.230949827
4,-1.503274658\C,1.8075593228,-0.0262643421,-2.0405832317\H,1.84323431
64,-2.0733150741,-1.2425157306\H,0.1679950545,-1.4557348009,-1.7129644
495\H,1.2188955027,0.6380999051,-2.6649455274\H,2.8829815287,-0.012577
5774,-2.1991235123\\Version=EM64L-G09RevA.01\State=1-A\HF=-1795.431278
7\RMSD=7.133e-09\RMSF=3.722e-06\Dipole=-0.2047669,-0.2408614,-0.231316
\Quadrupole=21.4073079,-8.5385536,-12.8687543,-13.1100886,13.5681629,-
8.8051275\PG=C01 [X(C34H41Au1P1)]\\@

TS6

1\1\GINC-SHI_03\FTS\RPBE1PBE\GenECP\C34H41Au1P1(1+)\YIN\09-Jul-2015\0\
\#p opt=(z-matrix,calcfc,ts,noeigen) genecp pbe1pbe\\Title Card Requir
ed\\1,1\C,1.7866293371,0.2388824243,0.505370407\H,2.0395920809,-0.1527
022353,1.6612872101\C,2.3134366678,1.1980661485,1.3958466293\C,3.71608
0458,1.5149821578,1.1573579591\H,1.7100177013,1.6539871486,2.178275071
3\C,4.1570254778,0.6039493711,-0.0077973352\C,3.9766280159,2.059460836
,-0.2421627045\H,3.1227643579,2.4038804592,-0.8203046107\H,4.870304209
9,2.6713954662,-0.3221088938\Au,-0.2113492364,-0.2342077209,0.26751322
16\H,4.3787210529,1.80815489,1.9648267716\C,5.5175102824,-0.0051727043
,-0.0508710866\C,5.9063873703,-0.9495119986,0.9061427074\C,6.411505828
,0.3346385666,-1.0696311627\C,7.1661769655,-1.5350442221,0.8490132806\
H,5.2190819749,-1.219321718,1.7051291816\C,7.6724453071,-0.2535631254,
-1.1297595876\H,6.1170348853,1.0653360247,-1.8192201681\C,8.0517683799
,-1.1875381486,-0.1702395631\H,7.46041968,-2.2602865436,1.6022932466\H
,8.3588233719,0.020154298,-1.9258678544\H,9.0365272675,-1.6437011937,-
0.2136872895\P,-2.434507249,-0.9683400648,0.0314023365\C,-2.9698004382
,-1.6906695324,1.6991417428\C,-2.0940472506,-2.9081603738,2.0161144462
\C,-2.6902199,-0.5892441317,2.7323178468\C,-4.4474188176,-2.0785073748
,1.8050368361\H,-2.3270750698,-3.7653881349,1.3769417187\H,-1.02551448
89,-2.681455934,1.9210686021\H,-2.2792955908,-3.2163020619,3.052471302
8\H,-3.2651846827,0.3199463165,2.5243841792\H,-2.9871243937,-0.9505148

563,3.724834344\H,-1.6267117835,-0.32638411,2.7721550234\H,-4.61628100
91,-2.5130547452,2.7981472029\H,-5.1062549775,-1.2104932975,1.71554517
75\H,-4.7501386313,-2.8277405929,1.0690608241\C,-2.4836126842,-2.20814
8428,-1.4106247563\C,-2.4869062535,-1.3678538656,-2.6947298888\C,-1.18
92992267,-3.0367169505,-1.3796496357\C,-3.6681400606,-3.1794393155,-1.
4258877416\H,-3.42079774,-0.8139346389,-2.8268026265\H,-1.6564891844,-
0.6507780312,-2.7114688571\H,-2.3642819683,-2.0359601324,-3.5559502012
\H,-1.0912259389,-3.6380193296,-0.4714198204\H,-1.1953183454,-3.725902
3113,-2.2333173062\H,-0.2995782755,-2.4031860356,-1.4666912957\H,-3.57
44298903,-3.8206068383,-2.3113159495\H,-3.6784938763,-3.8391009544,-0.
5531774968\H,-4.6370544725,-2.6803110183,-1.5024864291\C,-3.653403582,
0.3549873344,-0.3438573941\C,-4.9860786956,-0.0153297,-0.5938070075\C,
-3.3231659928,1.7271002675,-0.3652864625\C,-5.9757014245,0.9241784068,
-0.8497648542\H,-5.2632604531,-1.0620967788,-0.5829355091\C,-4.3388330
821,2.662252178,-0.61231557\C,-5.6504150956,2.2759045381,-0.8523627073
\H,-6.9938614387,0.5986284563,-1.0412676728\H,-4.0764758916,3.71645552
29,-0.6243472905\H,-6.4110611068,3.0268248607,-1.0450259999\C,-1.96466
43306,2.3163550058,-0.1711549148\C,-1.1027403211,2.475775926,-1.265167
6576\C,-1.6137776868,2.8997893311,1.0524112704\C,0.0768160204,3.207165
9871,-1.1391032631\H,-1.38372917,2.0596501451,-2.2292560184\C,-0.43030
91323,3.6246100863,1.1794181604\H,-2.2895899453,2.8109096717,1.8990255
338\C,0.4142429249,3.7867618038,0.0823411689\H,0.7156006598,3.34763330
56,-2.0072820638\H,-0.1880910757,4.0949123715,2.129173925\H,1.31686346
32,4.3854409244,0.1730387961\C,2.9227879373,-0.2247786579,-0.350768903
1\C,3.0002350776,-1.6778348598,-0.765605126\C,2.6617399693,-0.64784594
66,-1.7823314855\H,3.9919824739,-2.1197186739,-0.7946819308\H,2.211220
5244,-2.3336146053,-0.4072297457\H,1.6407293165,-0.5795628387,-2.14752
67265\H,3.4278908206,-0.3712704993,-2.5021125596\\Version=EM64L-G09Rev
A.01\State=1-A\HF=-1795.4057537\RMSD=8.082e-09\RMSF=1.784e-06\Dipole=0
.2440421,0.1450148,0.3794637\Quadrupole=35.5352137,-14.3814916,-21.153
7221,4.8133485,2.0842521,0.1904346\PG=C01 [X(C34H41Au1P1)]\\@

IN7

1\1\GINC-SHI_02\FOpt\RPBE1PBE\GenECP\C34H41Au1P1(1+)\YIN\14-Jul-2015\0
\\#p opt genecp pbe1pbe\\Title Card Required\\1,1\C,-0.2580375517,-1.1
788517596,0.198568245\C,-0.227691131,0.0121480179,0.8988822061\C,1.102
0367863,0.670477733,0.7476738707\H,-0.9211881128,0.2816642844,1.693825
2313\C,1.965820742,-0.2781343653,-0.0876943243\C,1.5509704255,1.048357
6796,-0.6439189361\H,0.800118527,1.0860334842,-1.4325308223\H,2.322373
5007,1.8106784186,-0.7137663659\Au,-1.6869837646,0.3615586395,-0.84687
37809\H,1.5085254637,1.2212514853,1.5905468739\C,3.4076900563,-0.52824
04504,0.2089384286\C,3.797982433,-1.0837362226,1.4326546102\C,4.387705
971,-0.2389267149,-0.7443009179\C,5.1393836618,-1.3387026036,1.6979316
333\H,3.0430510146,-1.3099561209,2.1824254342\C,5.7309616052,-0.496851

5031,-0.4820235856\H,4.0940676018,0.1973415479,-1.6965452774\C,6.10876
83274,-1.0463343659,0.7398831284\H,5.4303835763,-1.7626276319,2.654950
8159\H,6.4825536751,-0.2652870036,-1.2314763072\H,7.1564660578,-1.2436
370648,0.9479188016\P,-3.2461442387,0.7835009474,-2.544818699\C,-4.443
6344018,-0.6847816499,-2.5892655255\C,-3.6742881268,-1.9452246586,-2.9
995004647\C,-4.9533663703,-0.8518546572,-1.150333253\C,-5.6573399286,-
0.5018665685,-3.506232352\H,-3.3852279144,-1.931600117,-4.0548724499\H
,-2.7737576057,-2.0935934961,-2.3919013576\H,-4.3227710556,-2.81739899
75,-2.8521389093\H,-5.4792484729,0.0412575653,-0.7955891607\H,-5.66322
00026,-1.6877943864,-1.1229102282\H,-4.141637679,-1.077283845,-0.44911
81385\H,-6.2537215939,-1.4216767165,-3.467587815\H,-6.3019260513,0.315
6200562,-3.1725659623\H,-5.3887018062,-0.3354730924,-4.5527522294\C,-2
.2967230147,1.0788883349,-4.162804213\C,-1.7608636853,2.5146497492,-4.
0892994981\C,-1.1032392171,0.1127063359,-4.2111188366\C,-3.1091635566,
0.893501758,-5.4486383795\H,-2.5611813643,3.2595771924,-4.1248933506\H
,-1.1740330548,2.6831616003,-3.1778062374\H,-1.0972731969,2.6886866199
,-4.944931644\H,-1.4089064979,-0.9370046223,-4.2330735039\H,-0.5327166
41,0.3095435318,-5.1273033247\H,-0.4275879986,0.2518719052,-3.36060557
27\H,-2.4547711777,1.1109396355,-6.3017267935\H,-3.4644529508,-0.13383
38736,-5.570212371\H,-3.9610483556,1.5730674949,-5.5243527862\C,-4.285
9165236,2.2582675895,-2.2137695484\C,-5.2022546421,2.6622684749,-3.200
2805939\C,-4.2528036518,2.966569407,-0.9927117253\C,-6.0779061477,3.72
06926411,-2.9996914587\H,-5.2456166745,2.1339538308,-4.1449617215\C,-5
.1606506816,4.0189024184,-0.8020166339\C,-6.0644716411,4.3974686939,-1
.7852323668\H,-6.769824484,4.0066032059,-3.7862326749\H,-5.1329301957,
4.5580444284,0.1407130321\H,-6.7478839733,5.2220431868,-1.6050545018\C
,-3.3100030661,2.7452810824,0.1434416411\C,-2.0070012923,3.2688097106,
0.0980044609\C,-3.7802084957,2.2185084605,1.3538861708\C,-1.2095184063
,3.287037195,1.2418246337\H,-1.6473189736,3.7227318398,-0.8221277258\C
,-2.9764558948,2.2263167123,2.4922555177\H,-4.7949065475,1.8325389625,
1.4083375565\C,-1.6951028891,2.7734867289,2.4425594081\H,-0.2170053021
,3.726233409,1.1958327354\H,-3.3653336648,1.8319962155,3.42709143\H,-1
.0841424383,2.8133788669,3.3402503246\C,1.0526314791,-1.4558614381,-0.
4300730588\C,1.5502960322,-2.8908260744,-0.422012555\C,1.1959296059,-2
.2430038896,-1.71339242\H,2.6008076859,-3.0327361088,-0.1870790472\H,0
.8856231784,-3.6499252216,-0.0184940609\H,0.2887182094,-2.5547688774,-
2.2243467779\H,2.0079940901,-1.9402700834,-2.3695953164\H,-1.019742830
9,-1.9484889199,0.3086505259\\Version=EM64L-G09RevA.01\State=1-A\HF=-1
795.4552156\RMSD=5.141e-09\RMSF=4.056e-06\Dipole=-0.9622547,0.0432193,
-0.4954506\Quadrupole=16.8665594,-13.9324138,-2.9341456,-12.4347964,13
.4389325,-2.1361905\PG=C01 [X(C34H41Au1P1)]\\@

IN8

1\1\GINC-SHI_02\FOpt\RPBE1PBE\GenECP\C34H40Au1Br1P1(1+)\YIN\22-Jul-201

5\0\\#p opt genecp pbe1pbe\\Title Card Required\\1,1\C,0.1757398174,0.
5053109068,-0.0270264843\C,0.1515897696,0.5140057895,1.3618016558\C,1.
2252110505,-0.0161954711,2.1010982982\C,2.3259940273,-0.5448514958,1.4
065608365\C,2.347308721,-0.5585817359,0.0198437096\C,1.2725775521,-0.0
323877553,-0.6948686705\H,-0.664343537,0.9116637522,-0.5798733862\H,3.
1563495024,-0.9434949189,1.9809646912\H,3.1994256416,-0.9795458643,-0.
5044604024\H,1.2819732776,-0.0402147126,-1.780740361\C,1.2048760964,-0
.0521484422,3.5327637076\C,1.0568648773,-0.2636351089,4.7461642734\C,0
.7346833999,-0.8640414579,6.0239710637\C,0.1994347296,-2.2846754574,5.
9500564551\C,1.5780726761,-2.046136164,6.4642832746\H,0.0875794897,-2.
7376324266,4.9700198269\H,-0.5960440857,-2.5183580267,6.6523129288\H,1
.7626139533,-2.1343719265,7.5316667774\H,2.4116456079,-2.3318544988,5.
8306774381\C,0.1452555078,0.0570842582,7.0854703935\H,0.8471139698,0.8
88633608,7.2540269468\H,0.0850206338,-0.4962641463,8.0300261239\C,-1.2
122014119,0.5764255297,6.7143544851\H,-1.2933413938,1.0621973803,5.740
1822306\C,-2.2952043651,0.4626200954,7.4803426855\H,-2.2590447728,-0.0
208007183,8.4548298591\H,-3.2596285489,0.8511786928,7.1661378716\Au,1.
9523182478,1.8346491314,4.5535337222\P,2.5530959439,4.0856040741,4.748
277115\C,1.5380057835,4.8291863817,6.1725678477\C,0.1336527457,4.20931
14587,6.145471999\C,2.2360945538,4.3958193622,7.4683190193\C,1.3882418
775,6.3539150353,6.1510437846\H,-0.4199155689,4.4460042587,5.232949062
\H,0.1663964181,3.1202313286,6.2497997059\H,-0.4393321432,4.6061540254
,6.9925619747\H,3.2228609017,4.8538746645,7.5836791196\H,1.6183161295,
4.7025931598,8.320924211\H,2.3528437118,3.306205797,7.5220761095\H,0.7
839082636,6.6484504128,7.0177617678\H,2.3392887332,6.8843377523,6.2375
746213\H,0.8640569441,6.7087222087,5.2591500309\C,2.2226553866,4.86402
5667,3.050316764\C,2.9803001236,3.9910446051,2.038655582\C,0.721184390
1,4.8003703204,2.7508716502\C,2.7151412303,6.3063916843,2.8949263708\H
,4.0555734776,3.9626098217,2.2472078808\H,2.6031074404,2.9622434114,2.
014878184\H,2.8479425685,4.4193768599,1.0373959493\H,0.1453973093,5.49
4704981,3.3706400136\H,0.5615441138,5.0921146458,1.7055282534\H,0.3094
820922,3.7925621632,2.8779465759\H,2.4373991694,6.6511137191,1.8912491
989\H,2.2617878039,6.9977836242,3.6100317748\H,3.8030063595,6.37954676
3,2.9728940744\C,4.3287744271,4.3995628739,5.0975905631\C,5.3148122269
,3.3895959792,5.1270630251\C,4.7317653358,5.7330999464,5.288648472\C,6
.65487765,3.7613695845,5.3092769322\C,5.0785911159,1.9210754739,5.0234
991636\C,6.0613159412,6.0787208401,5.4890030302\H,3.9953957426,6.52641
5187,5.2696247262\C,7.0333200528,5.0847355082,5.488413247\H,7.40689239
52,2.9776064979,5.3280177444\C,4.6501030348,1.1960481018,6.144329716\C
,5.4614251591,1.2219912179,3.8736760699\H,6.3323949075,7.1197851073,5.
6369145204\H,8.0798562907,5.3353457854,5.6349207707\C,4.6118697382,-0.
1959901963,6.1145751347\H,4.3932597891,1.7279262175,7.0571231195\C,5.4
146472112,-0.1707361873,3.8428810353\H,5.8236275514,1.7735493548,3.009
8180872\C,4.9948382718,-0.882990067,4.9638559602\H,4.3072098815,-0.743

5435958,7.0018964853\H,5.7399252063,-0.6994579324,2.9506136944\H,4.991
9042375,-1.969634804,4.9495716661\Br,-1.3438839782,1.2410444341,2.2418
306271\\Version=EM64L-G09RevA.01\State=1-A\HF=-4365.8865695\RMSD=9.081
e-09\RMSF=1.983e-06\Dipole=1.0001197,0.3888435,-0.0731834\Quadrupole=-
8.6514478,7.7670553,0.8843924,5.9942917,-1.5640945,4.390811\PG=C01 [X(
C34H40Au1Br1P1)]\\@

TS7

1\1\GINC-SHI_02\FTS\RPBE1PBE\GenECP\C34H40Au1Br1P1(1+)\YIN\22-Jul-2015
\0\\#p genecp pbe1pbe opt=(calcfc,ts,noeigen)\\Title Card Required\\1,
1\C,-1.3273491118,2.082979268,-3.189407149\C,-2.2568162169,1.821396730
2,-2.03152374\C,-1.6455193563,3.1978718359,-2.2540287357\H,-0.33716088
69,1.6392819713,-3.1481743081\H,-1.7778709093,2.0960857211,-4.17863312
89\H,-2.3142764759,3.9851757322,-2.5916567027\H,-0.8810466577,3.514842
2312,-1.5521774382\C,-1.8136263323,1.0628544076,-0.8409695499\C,-2.574
0414999,0.7347296895,0.150908209\C,-2.8664289884,-0.0062835926,1.34195
40382\C,-2.6113764038,0.5669578456,2.6016230293\C,-3.4099017315,-1.306
0439511,1.2980945855\C,-2.8683719667,-0.132238395,3.7702084186\C,-3.67
88427041,-2.0049378655,2.4689159986\C,-3.405849083,-1.4175166072,3.700
8923083\H,-4.1004538129,-3.0027287092,2.4134484014\H,-3.6205826975,-1.
9696607648,4.6112061223\C,-3.7270628048,1.5637323573,-2.3611896825\H,-
3.8098833632,0.6661994037,-2.984286873\H,-4.1187161317,2.3995146013,-2
.9583762555\C,-4.5477066981,1.3868991002,-1.1296506445\C,-4.3389460598
,2.1124662105,-0.0038243052\H,-5.2950231099,0.5965190379,-1.1193165002
\H,-4.9338519519,1.9424346337,0.8884928506\H,-3.6819316928,2.978416960
4,-0.0002787684\Au,0.0991448966,0.2545080131,-0.5490836773\H,-2.193439
4281,1.5682503191,2.631982631\H,-2.6589497927,0.3216896289,4.733792836
1\P,2.1246392339,-0.9267172888,-0.4620205121\C,3.4401800201,-0.2379753
679,0.6229050906\C,4.6194030834,-0.9879861301,0.7812750899\C,3.3054199
904,0.9601862669,1.3578075536\C,5.6379474022,-0.591202046,1.636954807\
H,4.7493219449,-1.907676162,0.225698941\C,4.3419245584,1.3389098114,2.
2244609517\C,5.4955481399,0.5812624116,2.3700024477\H,6.5333247245,-1.
1986667031,1.7299056003\H,4.2301334591,2.2632298264,2.784289353\H,6.27
85328723,0.9083277464,3.0479542453\C,2.8379135095,-0.955462443,-2.2181
27675\C,1.8474771946,-1.6765931247,-3.1395659729\C,2.9450709444,0.5156
310287,-2.6433845738\C,4.224757634,-1.5893370233,-2.3620762753\H,1.829
4121889,-2.7565111464,-2.962233057\H,0.8270595455,-1.289252083,-3.0328
52756\H,2.1577280176,-1.5242811301,-4.1807246436\H,3.6392617447,1.0718
792654,-2.0037466038\H,3.3299386843,0.5626237688,-3.6696350789\H,1.972
5454104,1.0207837414,-2.6226543414\H,4.4978759246,-1.5749166745,-3.424
478943\H,4.9911654563,-1.0251122445,-1.8238188054\H,4.2578225684,-2.63
16864518,-2.0346389777\C,1.6964434976,-2.6467678723,0.2385730061\C,1.5
460364623,-2.458310942,1.7543472723\C,0.3370896214,-3.0849151805,-0.32
79147023\C,2.7108959024,-3.7582427349,-0.0461146634\H,2.497379388,-2.2

250076651,2.2413225425\H,0.8290740998,-1.6629723492,1.9939866993\H,1.1
639211175,-3.3908442268,2.1878146534\H,0.3471498767,-3.2043727172,-1.4
143147892\H,0.0776825267,-4.0575281776,0.1088401469\H,-0.4616438566,-2
.3804270751,-0.0730772559\H,2.3469017149,-4.6818238743,0.4210054472\H,
2.8172075034,-3.9613202275,-1.1158610476\H,3.6981326892,-3.5610682184,
0.3777838535\C,2.1583400462,1.9134941181,1.3104931195\C,1.3020483776,2
.0210807127,2.4128721125\C,2.0421866991,2.8493466338,0.2749223588\C,0.
3527707692,3.0386317198,2.4781147116\H,1.4049301017,1.3181835961,3.235
7821814\C,1.0967032888,3.8703480471,0.3446408905\H,2.7288745707,2.8048
23274,-0.5658234752\C,0.2491941664,3.9685986031,1.4463641732\H,-0.2814
58293,3.1268222693,3.3567253289\H,1.0492535623,4.6117554991,-0.4488470
642\H,-0.4683228405,4.7819096975,1.512510271\Br,-3.7913032732,-2.13024
35711,-0.3481346054\\Version=EM64L-G09RevA.01\State=1-A\HF=-4365.86904
84\RMSD=6.543e-09\RMSF=3.075e-06\Dipole=-0.4062893,0.3051128,0.5274955
\Quadrupole=16.0374075,-11.0485672,-4.9888403,-10.9044549,-2.0778327,-
3.130065\PG=C01 [X(C34H40Au1Br1P1)]\\@

IN9

1\1\GINC-SHI_02\FOpt\RPBE1PBE\GenECP\C34H40Au1Br1P1(1+)\YIN\21-Jul-201
5\0\\#p genecp pbe1pbe opt\\Title Card Required\\1,1\C,-0.0280413314,-
0.0496517638,-0.0028284626\C,-0.0050884103,-0.0342805131,1.538033235\C
,1.2743416524,-0.0645408342,0.6566653592\H,-0.4020437457,-0.9590333537
,-0.4625130366\H,-0.3885030462,0.8799390684,-0.4350932582\H,1.85145675
78,0.8546961031,0.7048138876\H,1.8508903345,-0.9838385036,0.6827976945
\C,-0.3458584441,-1.2356991584,2.2526571135\C,-0.8132668975,-0.8732940
868,3.5800854026\C,-1.7716023313,-1.7305715774,4.3299560133\C,-1.41258
35877,-2.3969612483,5.5056842286\C,-3.0881416143,-1.8892414809,3.87469
98381\C,-2.3270590402,-3.173011655,6.2102489594\C,-4.0170641027,-2.653
4953571,4.5715378701\C,-3.6331362972,-3.2949615878,5.7456608547\H,-5.0
316972292,-2.7407903709,4.1978042762\H,-4.3598854513,-3.8865241741,6.2
944966264\C,-0.4646946928,1.2096102112,2.2971515309\H,-1.3548492276,1.
6367332459,1.8223869332\H,0.3027588559,1.9912076178,2.3389203999\C,-0.
800151594,0.6775112219,3.6695754305\C,0.2649909855,-0.0735615591,4.349
1700633\H,-1.5719471479,1.1523713787,4.2676161526\H,0.1977200083,-0.19
10363831,5.4268472266\H,1.2753939644,-0.0708170579,3.9491798324\Au,-0.
2400681776,-3.1425890626,1.5726806518\H,-0.3933167083,-2.3047485087,5.
8711919419\H,-2.0213162527,-3.6742228689,7.1236294538\P,-0.4167945142,
-5.3165898097,0.6195783223\C,1.0796478568,-6.382801421,0.717948707\C,0
.9866196406,-7.6975318744,0.2294964407\C,2.3308116812,-5.9311337864,1.
1919651068\C,2.0836995045,-8.5473897193,0.1852320128\H,0.0374211037,-8
.0684567709,-0.1367202462\C,3.4328934561,-6.7962781482,1.1166256363\C,
3.3212267675,-8.0879831908,0.6212766918\H,1.9698238904,-9.5580802496,-
0.1953656207\H,4.3919828195,-6.4403232353,1.4824860595\H,4.1938980117,
-8.733605863,0.5866341418\C,-0.7082727533,-5.0515217666,-1.2352433943\

C,-2.0177368975,-4.2753781444,-1.4196460585\C,0.4669210389,-4.18266622
71,-1.7089448799\C,-0.7370468823,-6.3171594256,-2.0962481621\H,-2.8975
450634,-4.8884023506,-1.2004249104\H,-2.058556408,-3.3773387593,-0.791
5805478\H,-2.0963649996,-3.9578367333,-2.4668447816\H,1.4290455005,-4.
6888308175,-1.5722655317\H,0.348072741,-3.976350112,-2.7799752995\H,0.
5069861477,-3.223144863,-1.1803905095\H,-0.933636081,-6.0190550546,-3.
133672776\H,0.222484679,-6.8411641704,-2.0870267924\H,-1.5246190854,-7
.0174915987,-1.807397217\C,-1.8306083709,-6.2323978072,1.5047767788\C,
-1.2376214468,-6.8091447218,2.7968379734\C,-2.897659542,-5.1948874884,
1.8842767631\C,-2.5111407699,-7.347498062,0.7052170759\H,-0.511770083,
-7.6045578718,2.6048287597\H,-0.7501652421,-6.0333457021,3.3998994263\
H,-2.0511162708,-7.2298417439,3.4006081801\H,-3.3199038972,-4.68194657
36,1.0156516276\H,-3.7193959436,-5.709660768,2.3983643794\H,-2.5022025
339,-4.4357445957,2.5668723792\H,-3.2890369103,-7.7952281446,1.3361948
2\H,-3.0060677863,-6.9707780245,-0.1944580261\H,-1.8333528225,-8.15569
78872,0.4208533081\C,2.6104808221,-4.6089157735,1.8186835042\C,2.17654
21656,-4.326141462,3.1213207695\C,3.468891708,-3.7026299824,1.18455901
73\C,2.6034831584,-3.1713523776,3.7755100315\H,1.5442896368,-5.0424199
464,3.6397404707\C,3.8882899233,-2.5448886643,1.8363941885\H,3.8301544
083,-3.9238852257,0.1833272348\C,3.4626877518,-2.280153594,3.136667716
4\H,2.2848733577,-2.9856477044,4.7978283662\H,4.5729726897,-1.86436415
26,1.336927943\H,3.8204627962,-1.3960698876,3.6579125536\Br,-3.6456020
489,-1.0533707251,2.2691627618\\Version=EM64L-G09RevA.01\State=1-A\HF=
-4365.9204322\RMSD=7.161e-09\RMSF=6.469e-06\Dipole=0.7503152,0.1876021
,0.079072\Quadrupole=-8.8009305,13.9720388,-5.1711083,3.257642,-5.7346
065,9.6128472\PG=C01 [X(C34H40Au1Br1P1)]\\@

TS8

1\1\GINC-SHI_03\FTS\RPBE1PBE\GenECP\C34H40Au1Br1P1(1+)\YIN\22-Jul-2015
\0\\#p genecp pbe1pbe opt=(calcfc,ts,noeigen) geom=check\\Title Card R
equired\\1,1\C,-0.0602831171,0.0588526021,-0.1014790076\C,0.0067879374
,0.0749320585,1.4399268635\H,0.9217500389,0.0195407891,-0.5698531666\H
,-0.7039654879,0.7943451216,-0.5772990978\H,-0.8797378579,0.5256964541
,1.8832164049\H,0.9260570032,0.4062356502,1.921776516\C,-0.0839496924,
-1.3572481091,1.1100120816\C,0.9233533515,-2.4441796844,1.2955208782\C
,0.7743207883,-3.2899710923,0.0496680313\H,0.6432950341,-2.9921304594,
2.2091093023\H,1.9333193521,-2.0510642517,1.4692435919\C,-0.3417161384
,-2.6871258374,-0.810745442\C,1.0996269399,-2.6608805896,-1.2645165062
\H,1.644888203,-1.7209842238,-1.2942424253\H,1.3571987817,-3.339236584
2,-2.0730480595\C,-0.8429613225,-1.4506219841,-0.074246054\Au,-2.91264
65106,-0.9478492916,-0.0962577682\H,0.8586188528,-4.3676264418,0.14112
03799\C,-1.2997965068,-3.5670638366,-1.5439644981\C,-2.1208424258,-4.4
997918613,-0.8980510135\C,-1.3820277726,-3.5027929329,-2.9398046569\C,
-2.9777601172,-5.3377953291,-1.6058753848\C,-2.2415475585,-4.322207350

5,-3.6627290666\H,-0.7387331612,-2.7968753637,-3.4594035544\C,-3.03952
5096,-5.2454942113,-2.9924375698\H,-3.5845883614,-6.0607753274,-1.0708
984342\H,-2.2765023473,-4.2521461764,-4.7459347244\H,-3.7026538729,-5.
9051796945,-3.5443417241\P,-5.1815839498,-0.4130306227,0.1966858264\C,
-5.3169792116,0.0983375,2.0234267725\C,-4.9327430257,-1.1031171683,2.8
949106713\C,-4.2794821318,1.2143757956,2.2166115827\C,-6.674776684,0.6
418034803,2.4808152048\H,-5.7085546002,-1.8750652399,2.8986050205\H,-3
.9885036885,-1.5605104862,2.5756422538\H,-4.8092195399,-0.7623259945,3
.9305097388\H,-4.465455607,2.0666888251,1.5535454765\H,-4.3350430695,1
.57550189,3.2510743854\H,-3.2602657164,0.8532977766,2.0373375153\H,-6.
6185290852,0.8260146213,3.5609392999\H,-6.917326697,1.5944593788,2.002
9732134\H,-7.4993843988,-0.0560116682,2.3159499517\C,-6.2517595188,-1.
9180219475,-0.2655407164\C,-6.3735899996,-1.9138306786,-1.794874903\C,
-5.4965467584,-3.1863477492,0.1508914532\C,-7.6497430352,-1.9647847086
,0.3587391953\H,-6.9245896505,-1.0461314678,-2.1700269787\H,-5.3898746
316,-1.9423664906,-2.2778252906\H,-6.914226592,-2.8164102241,-2.105405
456\H,-5.365651847,-3.2710888287,1.2326518305\H,-6.0722212923,-4.05961
89386,-0.1813994354\H,-4.5086237322,-3.2391783398,-0.3157589322\H,-8.1
421209459,-2.8841543965,0.0180804777\H,-7.6186949472,-2.0059948343,1.4
511871134\H,-8.290520817,-1.1350342494,0.0512806866\C,-5.8361890016,1.
0244461249,-0.7539782813\C,-7.1846898413,1.3642907444,-0.5448063297\C,
-5.0693209116,1.841663866,-1.6161360814\C,-7.7699415083,2.4788191538,-
1.1302658341\H,-7.7968621252,0.7513297789,0.1033533817\C,-5.6733210942
,2.9784927636,-2.1772605948\C,-7.0014308072,3.303694114,-1.9426689777\
H,-8.8165863052,2.7004725288,-0.9439485206\H,-5.0784093747,3.598143520
9,-2.8422949208\H,-7.4346851121,4.1860000367,-2.4045795397\C,-3.669769
6417,1.6040436041,-2.0631015263\C,-3.3464432979,0.510715056,-2.8768945
946\C,-2.6948247453,2.5860851149,-1.841718253\C,-2.0852896583,0.407501
9662,-3.4600368039\H,-4.1058936995,-0.2340869793,-3.0966621901\C,-1.43
43043686,2.4839997504,-2.4260854148\H,-2.9403666992,3.4498632754,-1.22
8690303\C,-1.1276951898,1.3959566341,-3.2423162112\H,-1.8646235907,-0.
4344173763,-4.1099695357\H,-0.6990535484,3.2671192301,-2.2605852947\H,
-0.1545914699,1.3294236575,-3.7212770293\Br,-2.0950351865,-4.674042074
1,0.9912390648\\Version=EM64L-G09RevA.01\State=1-A\HF=-4365.8927028\RM
SD=9.096e-09\RMSF=2.384e-06\Dipole=0.775566,1.0382503,0.2635812\Quadru
pole=18.4795974,-6.0254547,-12.4541427,-4.8485069,1.2239754,3.5366962\
PG=C01 [X(C34H40Au1Br1P1)]\\@

IN10

1\1\GINC-SHI_03\FOpt\RPBE1PBE\GenECP\C34H40Au1Br1P1(1+)\YIN\20-Jul-201
5\0\\#p opt genecp pbe1pbe\\Title Card Required\\1,1\C,-0.1787967279,-
0.158654779,0.1891775568\C,-0.2440579736,-0.3524609706,1.7360509992\H,
0.8471863823,-0.0822264073,-0.1869856943\H,-0.7768242411,0.6431342935,
-0.2491615354\H,-0.8309946333,0.3510926248,2.3317265274\H,0.7566837277

,-0.4320855466,2.1783675378\C,-0.820513337,-1.7433120867,1.4589520072\
C,-0.5968555923,-3.1712888089,1.9122269246\C,-0.2117250502,-3.85522564
78,0.5988651705\H,-1.4713922003,-3.6261066441,2.3879342701\H,0.2210510
805,-3.2144296899,2.6430782563\C,-0.334488083,-2.87366278,-0.580431681
9\C,1.0186372589,-3.3408912348,-0.0775352259\H,1.658075517,-2.63615260
88,0.4521766592\H,1.553776652,-4.03016257,-0.7248048007\C,-0.730770326
6,-1.5902564481,0.0774047553\Au,-2.9128284572,-1.3641207233,0.67392637
45\H,-0.478466652,-4.896906916,0.4513503227\C,-0.7422609357,-3.2524168
131,-1.9645025476\C,-1.943568404,-3.8904488865,-2.2881417241\C,0.11060
79548,-2.9364007779,-3.0299781911\C,-2.2857988388,-4.2118714888,-3.598
5692436\C,-0.216381292,-3.233948195,-4.3476191577\H,1.0555729871,-2.44
91676925,-2.8030172278\C,-1.4180791171,-3.8771963316,-4.6328008934\H,-
3.2224072859,-4.719943405,-3.8029357433\H,0.469332265,-2.9742969243,-5
.1484989252\H,-1.6820236999,-4.1275121979,-5.655929018\P,-5.0041601237
,-1.011027697,1.7508416905\C,-4.7028108651,0.2041604851,3.1745985239\C
,-3.745359642,-0.4339355942,4.1857565032\C,-4.0288152729,1.4305318047,
2.5442443971\C,-5.9778813409,0.6726930057,3.881631424\H,-4.2095910485,
-1.2560459264,4.7389804815\H,-2.8343827286,-0.8089246326,3.7050541922\
H,-3.448805909,0.324300743,4.9209174222\H,-4.6784390656,1.9204161751,1
.8113991984\H,-3.8101851614,2.1593512072,3.334553284\H,-3.0858539956,1
.1741453748,2.0482534148\H,-5.6934373877,1.3846408664,4.6662726171\H,-
6.6557403163,1.1939927712,3.1996911969\H,-6.5243539284,-0.1400561174,4
.3651954547\C,-5.700149726,-2.6908804217,2.312766056\C,-6.3891863121,-
3.3168000563,1.0927776272\C,-4.5116385893,-3.5813622908,2.7077465308\C
,-6.6703068942,-2.662635433,3.5002282741\H,-7.3118189871,-2.7979367186
,0.8193442895\H,-5.7329938222,-3.3279250934,0.2145209461\H,-6.64489118
06,-4.3572828087,1.3281419019\H,-3.9347973266,-3.1704650174,3.54203597
69\H,-4.8962088839,-4.5596876021,3.0219710295\H,-3.8309843786,-3.74304
87979,1.8659733147\H,-7.0034355324,-3.6907553921,3.688357116\H,-6.1948
900944,-2.3067592003,4.4186640029\H,-7.5697664267,-2.0689403029,3.3232
925496\C,-6.2683205071,-0.1848234042,0.7048590322\C,-7.5985163982,-0.1
495508176,1.1547299759\C,-5.9438104665,0.4999458781,-0.4868868613\C,-8
.5921633563,0.5292199666,0.4618536402\H,-7.8696032649,-0.6527867323,2.
0747827334\C,-6.9569905463,1.1971129664,-1.1620490434\C,-8.2670906875,
1.2132189925,-0.7040758282\H,-9.6107287826,0.5282364916,0.8382939209\H
,-6.6993917099,1.7188399011,-2.0794981879\H,-9.0288157547,1.7544928892
,-1.2574720069\C,-4.6008807605,0.5554607439,-1.1264629955\C,-4.0461010
489,-0.5742470267,-1.7552991475\C,-3.9490793409,1.7862698759,-1.259236
8719\C,-2.8804967202,-0.4559028724,-2.5203367348\H,-4.5878108241,-1.51
74951339,-1.7365582133\C,-2.7793282169,1.8963286131,-2.008356517\H,-4.
3783829563,2.6682684997,-0.7910785369\C,-2.2468485488,0.7780442148,-2.
6457615902\H,-2.493073856,-1.3240794273,-3.0436041696\H,-2.2972117155,
2.8646266002,-2.112053199\H,-1.3519500805,0.8693927698,-3.2547249939\B
r,-3.1932436819,-4.3514081567,-0.9340873396\\Version=EM64L-G09RevA.01\

State=1-A\HF=-4365.9231981\RMSD=2.796e-09\RMSF=5.413e-06\Dipole=0.1099
365,0.8066502,0.4588814\Quadrupole=12.6405911,-13.3567731,0.716182,-7.
6200438,-12.2312843,2.1320057\PG=C01 [X(C34H40Au1Br1P1)]\\@

TS9

1\1\GINC-SHI_02\FTS\RPBE1PBE\GenECP\C34H40Au1Br1P1(1+)\YIN\18-Aug-2015
\0\\#p opt=(calcfc,ts,noeigen) genecp pbe1pbe\\Title Card Required\\1,
1\C,-2.4659755218,-2.7087891819,2.72442306\C,-1.9397748602,-1.96059658
4,3.9997373899\H,-3.4954102574,-3.0762453171,2.8010420797\H,-1.8195192
889,-3.5261204259,2.3847763859\H,-1.0028579831,-2.3510949977,4.4133869
942\H,-2.6756982614,-1.8855628426,4.8076144805\C,-1.8217116255,-0.7547
309761,3.0997260787\C,-1.4420176786,0.681733225,2.9742090313\C,-1.8480
113985,1.0467260814,1.5527439414\H,-0.3803676622,0.876046048,3.1703867
593\H,-2.0195585047,1.3122205924,3.6701734121\C,-2.5406346115,-0.56919
06959,0.7928953655\C,-3.2572458059,0.6824239467,1.1935830193\H,-3.9470
289476,0.5605839806,2.0311669873\H,-3.6845616544,1.267527564,0.3840401
853\C,-2.2904840065,-1.377208094,2.0118096309\Au,-0.1589930243,0.02018
26599,0.3280463203\H,-1.4639929947,1.9592203362,1.0884648114\C,-2.7082
632698,-1.175357146,-0.5605949396\C,-2.9113848139,-2.5659717316,-0.611
4259325\C,-2.7886791741,-0.4879204968,-1.7859066297\C,-3.2124596783,-3
.227702567,-1.7948922661\H,-2.8564866451,-3.1361317324,0.3083632815\C,
-3.0863719323,-1.1423907308,-2.9761821803\C,-3.3074151351,-2.515019238
,-2.9847934983\H,-3.3791664178,-4.300529179,-1.7806903106\H,-3.1397192
383,-0.5691547783,-3.8956773702\H,-3.5485144854,-3.0164383493,-3.91700
27818\P,1.9098262142,-0.7544905715,-0.4269023004\C,1.7541064809,-1.255
6369814,-2.2474810853\C,0.7216267686,-2.3823879286,-2.3605947424\C,1.2
352600738,-0.0110510001,-2.9798023153\C,3.0594617756,-1.690471695,-2.9
224806686\H,1.091713896,-3.326043042,-1.9465655103\H,-0.2257995461,-2.
1317019707,-1.8710139213\H,0.5077711985,-2.5564337036,-3.4222983882\H,
1.9478894415,0.8191709839,-2.9176895303\H,1.1083042723,-0.2556207766,-
4.0418000992\H,0.26777344,0.3266303923,-2.5939080148\H,2.8238148439,-1
.990722085,-3.9509830339\H,3.7814406146,-0.8719246302,-2.9838380167\H,
3.5342345543,-2.5472862296,-2.4377924704\C,2.3801409138,-2.1845923733,
0.7438642672\C,2.9418745829,-1.5359199292,2.0162137442\C,1.1001462404,
-2.9455364477,1.1187049449\C,3.3891781597,-3.1956850419,0.1899116344\H
,3.9189247717,-1.0735353719,1.8511464721\H,2.2635095476,-0.7715373855,
2.4160779648\H,3.0589727552,-2.3106631085,2.783957031\H,0.5845767453,-
3.3656423926,0.2505345657\H,1.3710363792,-3.7777682376,1.7806960653\H,
0.3955284984,-2.3009084079,1.6549743407\H,2.9999801917,-3.7407119357,-
0.6751650625\H,4.3496730056,-2.7490431125,-0.0770331468\H,3.593406846,
-3.9374399563,0.9719449757\C,3.2893130369,0.4582047364,-0.3323516972\C
,4.5798255871,-0.0100645271,-0.6395511339\C,3.1481613117,1.7956975027,
0.0973579077\C,5.7045650299,0.7960667798,-0.5314479345\H,4.7176964674,
-1.0321534422,-0.9684047068\C,4.2990427149,2.5915320443,0.2082015337\C

,5.5632427581,2.1097137255,-0.0997800337\H,6.6826567403,0.3950002714,-
0.779927145\H,4.1806697401,3.6204688193,0.5359122203\H,6.4297081945,2.
7576187693,-0.0052022559\C,1.8812809805,2.4994202224,0.4522550523\C,1.
647755753,2.8624846726,1.7859223435\C,1.0273092172,2.9983354789,-0.539
9817325\C,0.5875489626,3.7011117617,2.1200479719\H,2.3243045451,2.5060
947488,2.559030725\C,-0.0255864324,3.8479749217,-0.2067851388\H,1.2165
297599,2.7550023899,-1.5814552789\C,-0.2480333558,4.2037595363,1.12266
5022\H,0.4318186277,3.9867690466,3.1569008117\H,-0.6582363416,4.253529
0303,-0.9917744048\H,-1.0546628515,4.8854314947,1.3780548488\Br,-2.416
125234,1.3660065858,-1.9359099802\\Version=EM64L-G09RevA.01\State=1-A\
HF=-4365.8903277\RMSD=4.722e-09\RMSF=5.196e-06\Dipole=0.4938023,-0.860
6551,0.5041696\Quadrupole=8.2003565,-2.598631,-5.6017256,4.338145,-3.5
283402,6.254315\PG=C01 [X(C34H40Au1Br1P1)]\\@

IN11

1\1\GINC-SHI_02\FOpt\RPBE1PBE\GenECP\C34H40Au1Br1P1(1+)\YIN\18-Jul-201
5\0\\#p opt genecp pbe1pbe\\Title Card Required\\1,1\C,-0.2799029662,-
0.0396633283,-1.3053856416\C,-0.1338343562,0.7664774091,0.0259001929\H
,0.6516230771,-0.2219718875,-1.8499300237\H,-1.0191570885,0.3809831788
,-1.9937432072\H,-0.7551902192,1.6654488564,0.1232940173\H,0.893600886
7,1.0351651218,0.3000741489\C,-0.6663175071,-0.4415088856,0.7347376314
\C,-0.9820974662,-0.913184863,2.0832378062\C,-1.8838955014,-2.14964548
31,1.9486040136\H,-1.4037313725,-0.1183829886,2.7097707924\H,-0.001438
446,-1.1643411063,2.5490140696\C,-1.0179971132,-2.5593538026,-0.418881
7401\C,-1.2465322652,-3.1309208599,0.9477499572\H,-0.2430925397,-3.447
6645376,1.3044014002\H,-1.8296829279,-4.0525565125,0.8758081071\C,-0.7
6513735,-1.1830391533,-0.4278484308\Au,-3.8778240944,-1.6839617383,1.4
969635613\H,-1.9149088008,-2.6629683154,2.9170786545\C,-1.035676912,-3
.3299089723,-1.6468779802\C,-1.4849042949,-2.6541718638,-2.8078981416\
C,-0.7001437499,-4.700373001,-1.7897855002\C,-1.6398877427,-3.29645713
3,-4.0220925171\H,-1.7651596044,-1.6108662466,-2.7200326189\C,-0.82767
00996,-5.336405852,-3.0193802115\C,-1.3064265387,-4.6455040718,-4.1276
972861\H,-2.013919916,-2.7512709943,-4.8826352181\H,-0.5429872149,-6.3
793081085,-3.1049308334\H,-1.4095133858,-5.1656587496,-5.075462034\P,-
6.1224649266,-1.2252756038,0.885489432\C,-6.7292717119,-2.6932964134,-
0.152931841\C,-5.8628149856,-2.7884828572,-1.4140856104\C,-6.47940748,
-3.9311768881,0.7211357335\C,-8.207995999,-2.683179818,-0.5498867672\H
,-6.0823808279,-1.987254625,-2.1278176583\H,-4.7929283662,-2.762354181
2,-1.1743846424\H,-6.0733270221,-3.7400155979,-1.9188705419\H,-7.06750
15739,-3.8971081493,1.6448990847\H,-6.7837515953,-4.8281002857,0.16671
70832\H,-5.4218008453,-4.0348592441,0.9897746128\H,-8.403581959,-3.568
1077196,-1.1688428007\H,-8.8647565978,-2.7475820626,0.3218178423\H,-8.
4908729049,-1.8067957851,-1.1392715279\C,-6.1600289583,0.4365332299,-0
.0467877097\C,-6.1136951344,1.5284985122,1.0307184294\C,-4.8771244974,

0.5409827836,-0.8851617433\C,-7.3537233985,0.6789411056,-0.9748513378\
H,-7.0331025024,1.5665512981,1.6219983452\H,-5.2701195295,1.381637446,
1.7166021212\H,-5.9833593936,2.5038763747,0.545077532\H,-4.8257777539,
-0.2188827304,-1.6706181278\H,-4.8530118665,1.5254051691,-1.3705585563
\H,-3.9851504748,0.4401111702,-0.257322393\H,-7.395446752,-0.044941049
3,-1.7942768431\H,-8.3138806807,0.6747997773,-0.4535448431\H,-7.245174
6426,1.6728361329,-1.4277848895\C,-7.3397460736,-1.071896818,2.2607290
108\C,-8.6680012863,-0.7273998007,1.9554339981\C,-6.999814042,-1.28944
6587,3.6135644078\C,-9.6422427203,-0.602050687,2.9367852154\H,-8.95394
03641,-0.5572474452,0.9252283873\C,-7.9990752983,-1.1675645411,4.59022
73231\C,-9.3057037054,-0.8286506328,4.2666290468\H,-10.6572286115,-0.3
320098745,2.6600909847\H,-7.7269840531,-1.3402683306,5.6277588707\H,-1
0.0539180066,-0.7395533759,5.0489710665\C,-5.6444308187,-1.6341359058,
4.137018579\C,-4.7651043865,-0.6204469556,4.5377402195\C,-5.3041199988
,-2.965357944,4.4019280146\C,-3.5707766435,-0.9328394387,5.183202501\H
,-5.0372058655,0.4181359197,4.3674532237\C,-4.1079323272,-3.2773432191
,5.0437986902\H,-5.9927362377,-3.7583162596,4.1220623266\C,-3.24009609
46,-2.2619897222,5.4397169367\H,-2.9105964815,-0.1334187723,5.51000745
04\H,-3.8654244888,-4.3154308533,5.2545896719\H,-2.3201178118,-2.50434
84827,5.9649271077\Br,0.0407103136,-5.740762459,-0.4091093469\\Version
=EM64L-G09RevA.01\State=1-A\HF=-4365.9027782\RMSD=9.513e-09\RMSF=2.931
e-06\Dipole=1.3668714,0.7601838,-2.1219022\Quadrupole=11.8735477,-14.4
481223,2.5745746,-3.984334,-14.2284781,7.6164572\PG=C01 [X(C34H40Au1Br

TS11

1\1\GINC-SHI_02\FTS\RPBE1PBE\GenECP\C34H40Au1Br1P1(1+)\YIN\21-Aug-2015
\0\\#p opt=(calcfc,ts,noeigen) genecp pbe1pbe\\Title Card Required\\1,
1\C,3.6108927653,-2.8064671998,-1.8659974995\C,3.0576045191,-2.5512903
643,-3.3123627795\H,4.6990605315,-2.8742238431,-1.7792055166\H,3.16509
54545,-3.6863023089,-1.3900148504\H,2.4243653795,-3.3311399131,-3.7450
06247\H,3.8307804746,-2.2834805118,-4.0393047308\C,2.3600406242,-1.325
4597474,-2.750868426\C,1.1540752501,0.6177543776,-1.8822367758\H,1.031
1500145,-1.497643001,-2.6487879227\H,0.9157950503,-0.2077942185,-3.961
6911591\C,1.396268051,-0.3238136082,-2.9898944291\C,3.0392713581,-0.47
7121322,-0.5503115475\C,2.392209625,0.835043892,-0.9965329002\H,3.1569
480178,1.4199423866,-1.5308628039\H,2.1142367783,1.4318091356,-0.12488
26053\C,2.9976184442,-1.4742086295,-1.4673725862\Au,-0.5571528554,-0.0
448372622,-0.8089679481\H,0.7949936477,1.5738853912,-2.2781291929\C,3.
6945866715,-0.6585020401,0.7538798265\C,3.4649512803,-1.8728596417,1.4
283860113\C,4.5245226863,0.279765898,1.393417145\C,4.0076702381,-2.135
9943388,2.6771957534\H,2.8155798035,-2.6052048257,0.9575010272\C,5.077
8853871,0.0209616808,2.6435935623\C,4.8152927349,-1.181706946,3.289735
5636\H,3.799733343,-3.0800647003,3.1714670903\H,5.7245226339,0.7624940
858,3.1003272292\H,5.249392704,-1.3702343536,4.2670963408\P,-2.4130275

409,-0.8868243252,0.3706013588\C,-1.7737343689,-1.7399665115,1.9368465
167\C,-0.9008511294,-2.9294724298,1.522180113\C,-0.8946623306,-0.69711
30958,2.641671024\C,-2.8423043894,-2.2094813483,2.9284638214\H,-1.4904
845416,-3.7501907926,1.1013198598\H,-0.135855211,-2.6354588336,0.79335
73685\H,-0.3886489378,-3.3188327178,2.4109520588\H,-1.4730747166,0.185
0947367,2.9378399327\H,-0.4779239039,-1.1405995342,3.5547379421\H,-0.0
592029176,-0.3723835163,2.0105340752\H,-2.3390911741,-2.7159039256,3.7
618078433\H,-3.4021449065,-1.3711372909,3.3513954482\H,-3.5488277684,-
2.9231377258,2.4961638915\C,-3.3719598425,-2.0378887947,-0.8048901949\
C,-4.1765257229,-1.1183252649,-1.7348346716\C,-2.3615302409,-2.8156601
411,-1.6629141049\C,-4.311592835,-3.0493989502,-0.1422236737\H,-4.9843
630872,-0.6013033197,-1.2091211806\H,-3.5375540945,-0.363262814,-2.209
5887736\H,-4.6241791481,-1.7250891199,-2.5317311501\H,-1.7154284292,-3
.4696416713,-1.0706474452\H,-2.9152434592,-3.4477490334,-2.3687214258\
H,-1.7255513871,-2.1378368578,-2.2431109782\H,-3.7776340932,-3.7615451
168,0.4940412417\H,-5.1042557772,-2.5782151277,0.4433176217\H,-4.80573
90167,-3.6295849025,-0.9316683341\C,-3.6177305231,0.3891610313,0.92352
3102\C,-4.7979287048,-0.0350618774,1.5596905638\C,-3.4515548826,1.7691
271807,0.6710419149\C,-5.7948961572,0.8555235268,1.9345168467\H,-4.950
1550771,-1.0870779224,1.7659667897\C,-4.4740931817,2.6516832812,1.0497
520455\C,-5.6334966697,2.2113659302,1.672982747\H,-6.6923084673,0.4879
952506,2.4234040944\H,-4.3371511579,3.7111111091,0.8519280913\H,-6.403
1991207,2.9240732294,1.9545027419\C,-2.2694342529,2.4177803796,0.03145
32502\C,-2.3808239414,2.9406817028,-1.2630248361\C,-1.1050884653,2.681
9669728,0.7660640022\C,-1.3511364275,3.7006538375,-1.813679674\H,-3.29
1365037,2.7657558061,-1.8306543421\C,-0.0807622976,3.4521936731,0.2186
390765\H,-1.0219574743,2.3167297008,1.7860419689\C,-0.2009613486,3.963
7671037,-1.0720948706\H,-1.4606151102,4.1098819519,-2.8144086614\H,0.8
019001295,3.6731773492,0.8131416597\H,0.5886570617,4.582594484,-1.4895
127595\Br,5.0268064632,1.9059616523,0.5730934354\\Version=EM64L-G09Rev
A.01\State=1-A\HF=-4365.8732886\RMSD=4.002e-09\RMSF=3.936e-06\Dipole=-
0.1915575,-1.8412747,-0.7763352\Quadrupole=13.3976792,-9.3488527,-4.04
88265,-7.3182566,-5.5094047,0.8748628\PG=C01 [X(C34H40Au1Br1P1)]\\@

IN13

1\1\GINC-SHI_02\FOpt\RPBE1PBE\GenECP\C34H40Au1Br1P1(1+)\YIN\15-Jul-201
5\0\\#p opt genecp pbe1pbe\\Title Card Required\\1,1\C,-0.0270086795,-
0.0070627527,-0.4694301033\C,-0.2342796739,0.251910642,1.0515671847\H,
0.9964460711,-0.0442944332,-0.8513939985\H,-0.6123613535,0.694375497,-
1.0758684033\H,-0.5282975646,1.2585048058,1.3601734243\H,0.636199453,-
0.0746183536,1.6279632898\C,-1.3498627105,-0.8383911251,1.0399674583\C
,-1.3243877839,-3.2269301195,1.729249808\H,-1.2970455521,-1.6195047284
,3.1446565524\C,-1.4147923989,-1.9005821049,2.0962870502\C,-0.51061919
9,-2.5970390364,-0.568077491\C,-1.1405374833,-3.676178514,0.2940539825

\H,-0.5020470815,-4.5663225134,0.2974237967\H,-2.0968733642,-4.0034250
459,-0.1400299991\C,-0.7259088477,-1.3223894959,-0.24335655\Au,-3.5280
757206,-2.7720160619,2.2615647965\H,-1.1439891438,-3.9725782808,2.5046
874327\C,0.3488624135,-3.0026762402,-1.7040458298\C,-0.0625160107,-2.7
490700827,-3.018537552\C,1.5849569825,-3.6393644113,-1.5288529972\C,0.
714477422,-3.1249487953,-4.1088906513\H,-1.0161277115,-2.2514221507,-3
.1724881319\C,2.3713488507,-4.025140651,-2.6078755165\C,1.9299366299,-
3.7701243315,-3.9027282846\H,0.3695081635,-2.9160912069,-5.117137107\H
,3.3255695146,-4.5100061745,-2.431681481\H,2.5439842361,-4.0716877403,
-4.7461495752\P,-5.8252996435,-3.2035753662,2.313013257\C,-6.051921395
1,-5.0837021044,2.3263297868\C,-5.4746215988,-5.6600961779,1.029374201
7\C,-5.2332982406,-5.5993384159,3.5179756709\C,-7.498089982,-5.5585558
601,2.5045540828\H,-6.0822449454,-5.4052449079,0.1559984204\H,-4.44618
59995,-5.3239116781,0.8506959202\H,-5.4580420374,-6.7539292296,1.10632
23121\H,-5.60892084,-5.2058233374,4.4689463173\H,-5.3173756256,-6.6923
03303,3.5566065296\H,-4.1700117615,-5.3473225887,3.429310742\H,-7.5024
978368,-6.6546982386,2.4630208283\H,-7.9045620042,-5.271372425,3.47806
65031\H,-8.1718682101,-5.2038919804,1.7202625043\C,-6.5843142689,-2.32
13541445,0.8084810915\C,-6.7072703923,-0.8416421729,1.1962137219\C,-5.
6040369853,-2.4337454899,-0.3694551555\C,-7.9459916443,-2.8546378927,0
.3530117146\H,-7.4654739614,-0.6748455805,1.9664617505\H,-5.7546343229
,-0.4344280937,1.5579246569\H,-6.9956142057,-0.2682902422,0.3068752408
\H,-5.4270393771,-3.4669212069,-0.6809902996\H,-6.0288133119,-1.898713
285,-1.2277602381\H,-4.6357358126,-1.9761412185,-0.1373150001\H,-7.892
1680378,-3.8912088157,0.007865421\H,-8.7182299588,-2.7771787507,1.1219
930729\H,-8.2838957985,-2.2489338109,-0.4966481665\C,-6.6629601824,-2.
5493491804,3.8080776073\C,-8.0631647596,-2.6604184489,3.8729599089\C,-
5.9883375316,-1.8860738359,4.8573041173\C,-8.7939227109,-2.1350891872,
4.9297652486\H,-8.6006723518,-3.1649290681,3.0795833537\C,-6.747309460
8,-1.3546110223,5.9106452088\C,-8.1297670368,-1.4723778043,5.955761632
\H,-9.8744755215,-2.2411651792,4.9458526221\H,-6.2237612685,-0.8474344
797,6.7161413217\H,-8.6829202924,-1.051137205,6.7900392669\C,-4.514422
4791,-1.6858944282,4.9891231952\C,-3.97591522,-0.3990829103,4.84847347
45\C,-3.6817747235,-2.714959888,5.4537086819\C,-2.6437327431,-0.145763
2151,5.1695032236\H,-4.6186595237,0.4124211438,4.5165095048\C,-2.35010
89502,-2.4597998521,5.7774302484\H,-4.0932742606,-3.7082875242,5.60828
48356\C,-1.8295140442,-1.1732022405,5.6436206573\H,-2.2498415976,0.862
4065086,5.0740282722\H,-1.7282810134,-3.2623460925,6.1648916641\H,-0.8
003060451,-0.9688706178,5.9258806956\H,-2.3241996437,-0.3532123386,0.8
837203663\Br,2.2639721916,-3.9427042729,0.2161162775\\Version=EM64L-G0
9RevA.01\State=1-A\HF=-4365.9473278\RMSD=7.621e-09\RMSF=2.287e-06\Dipo
le=-2.3949608,0.3016699,0.5226874\Quadrupole=14.9970858,-17.6367705,2.
6396848,3.0998385,-17.3279641,4.4419754\PG=C01 [X(C34H40Au1Br1P1)]\\@

TS10

1\1\GINC-SHI_03\FTS\RPBE1PBE\GenECP\C34H40Au1Br1P1(1+)\YIN\21-Jul-2015
\0\\#p opt=(calcfc,ts,noeigen) geom=check genecp pbe1pbe\\Title Card R
equired\\1,1\C,0.0207641153,0.0001854968,0.0327614005\C,-0.0092947329,
-0.0380072985,1.5680590105\H,1.032845233,0.0235639994,-0.3634184235\H,
-0.6902728777,0.6431294922,-0.4797942231\H,-0.9187441345,0.3020966924,
2.0587290918\H,0.9122653498,0.26021801,2.0542560698\C,0.2647082352,-1.
8228616459,1.5707772198\C,-0.7040274917,-2.6162133221,2.4469693213\C,-
2.0280428093,-2.6431539145,1.70356348\H,-0.7911121004,-2.1359242758,3.
4292088668\H,-0.3199063114,-3.6261104241,2.6291144828\C,-1.9146843959,
-1.7062111512,0.5011912833\C,-1.9991708202,-3.2237161537,0.3387175466\
H,-1.1114364107,-3.7556185046,0.004172536\H,-2.9372951006,-3.57822494,
-0.0781181854\C,-0.4899752918,-1.2899073448,0.4916148998\Au,2.30754479
02,-2.1893038043,1.665829278\H,-2.9597552857,-2.6661736808,2.258882448
7\C,-2.9853088265,-0.8354145311,-0.0656032871\C,-3.7755397562,0.049395
356,0.6798105236\C,-3.2116500557,-0.8848941287,-1.4484082047\C,-4.7521
299873,0.8384808023,0.0774125359\C,-4.1738840623,-0.0961439333,-2.0653
803642\H,-2.6151518706,-1.5744531733,-2.0410834766\C,-4.949344164,0.76
71799839,-1.2965439686\H,-5.3522157231,1.5041227583,0.6883609247\H,-4.
3242089653,-0.1618790813,-3.1386052791\H,-5.71272235,1.3847400209,-1.7
601813008\P,4.5951371737,-2.5690377632,2.0869222098\C,4.6914787639,-3.
8476028467,3.4830877852\C,4.0389201318,-3.2527081201,4.7364200885\C,3.
8584555393,-5.0440665946,3.0012216056\C,6.0947001488,-4.3550712133,3.8
266462956\H,4.6582472352,-2.4768153114,5.197177878\H,3.0494499178,-2.8
299074493,4.5252933156\H,3.9105644463,-4.0485422197,5.4805556019\H,4.2
783050035,-5.4897166917,2.0924090991\H,3.8611296514,-5.8166153576,3.78
02464646\H,2.8169369231,-4.7650327919,2.8035722069\H,6.0097005434,-5.0
539334379,4.6680179914\H,6.5454150694,-4.9026042102,2.9944836327\H,6.7
769265904,-3.5587983031,4.1357982908\C,5.3742118676,-0.8858017969,2.52
30569364\C,5.6151925817,-0.1695079725,1.1869077366\C,4.3558478121,-0.0
666493103,3.3302326263\C,6.6802916755,-0.93812849,3.32119252\H,6.40267
32327,-0.6459466837,0.5960722615\H,4.7038788441,-0.1315487935,0.576794
191\H,5.9225297573,0.8638460917,1.3897621333\H,4.1021705983,-0.5278276
304,4.2882178977\H,4.7874363844,0.9199297323,3.540202023\H,3.426482672
9,0.0835124715,2.7699440334\H,7.0259142908,0.0905006812,3.483443878\H,
6.5480929591,-1.3913030271,4.3081143937\H,7.4841947186,-1.4599629081,2
.7970344644\C,5.5872385091,-3.2423715884,0.691884115\C,6.9718049262,-3
.4080866424,0.8737336475\C,5.038700232,-3.543003491,-0.5736057862\C,7.
8020991924,-3.8493800731,-0.1478017324\H,7.4189896095,-3.1865338895,1.
8344601327\C,5.8942767763,-3.9815774163,-1.5949787681\C,7.2589696451,-
4.13615244,-1.3950506138\H,8.8666541644,-3.9649360885,0.0331475123\H,5
.4614796798,-4.2123974977,-2.5643801515\H,7.891765606,-4.4809175171,-2
.207639172\C,3.6005061604,-3.4525304447,-0.9606167292\C,3.1698744623,-
2.4166624806,-1.7991164811\C,2.7103370952,-4.4911511015,-0.6614866702\

C,1.8805826106,-2.4191189109,-2.3264855675\H,3.8643333822,-1.622293453
6,-2.0613951224\C,1.4239780482,-4.4977872286,-1.1964600411\H,3.0428765
282,-5.3187586219,-0.0412990061\C,1.0070812123,-3.4642165641,-2.032969
7683\H,1.5710303362,-1.6202515957,-2.9954351478\H,0.7597396759,-5.3315
567851,-0.983102484\H,0.0147155101,-3.4866689065,-2.4759546419\Br,-3.5
822601036,0.2261301676,2.5591074516\\Version=EM64L-G09RevA.01\State=1-
A\HF=-4365.9002914\RMSD=5.950e-09\RMSF=4.631e-06\Dipole=0.3883922,0.21
375,-0.6033856\Quadrupole=32.0090178,-19.3933408,-12.615677,-16.216078
8,15.2663936,-3.4087824\PG=C01 [X(C34H40Au1Br1P1)]\\@

IN12

1\1\GINC-SHI_02\FOpt\RPBE1PBE\GenECP\C34H40Au1Br1P1(1+)\YIN\20-Jul-201
5\0\\#p opt genecp pbe1pbe\\Title Card Required\\1,1\C,0.0429035596,0.
0975456818,-0.038543622\C,0.0610457601,0.2339057204,1.4476269463\C,1.5
053457073,0.1894476344,1.8979835646\H,-0.5185234719,-0.6188531141,1.84
10186403\H,-0.4963729773,1.1269851692,1.7644415511\C,2.3545398595,-0.1
383969132,0.685809914\C,2.4403440111,1.2182036237,1.3365914579\H,2.027
0764339,2.0707333054,0.799619007\H,3.3287101328,1.4380625331,1.9219500
572\Au,-1.6573045439,0.2177117655,-1.1246392307\H,1.7428276881,-0.2512
41521,2.8609213128\C,3.5040547479,-1.0837472997,0.7131785835\C,3.34535
96323,-2.4578964925,0.9424165995\C,4.8039198688,-0.61928715,0.48202956
55\C,4.4280934228,-3.330438297,0.948603038\C,5.8980066701,-1.477683388
3,0.4839960878\H,4.9491890671,0.4437783491,0.3066007014\C,5.7084573474
,-2.8360586527,0.7184596351\H,4.2669503082,-4.3862457147,1.1385709711\
H,6.89490918,-1.0860882642,0.3059327069\H,6.554172393,-3.517153289,0.7
278270348\P,-3.719631464,0.0434954521,-2.2953435819\C,-5.0193181453,-0
.546929726,-1.0488419783\C,-4.6193509489,-1.9385877611,-0.5440524053\C
,-4.9574486375,0.448474506,0.1185292236\C,-6.4621538736,-0.5829572433,
-1.5620339813\H,-4.7496882641,-2.7113368079,-1.3082125606\H,-3.5810401
383,-1.9680315999,-0.1926201593\H,-5.2652647232,-2.2065956302,0.301077
8067\H,-5.2257374936,1.4617900079,-0.200122219\H,-5.6795622077,0.14101
30166,0.8850294901\H,-3.9652778876,0.4784138662,0.5841004561\H,-7.1003
452625,-0.9698041367,-0.7578438882\H,-6.8314512721,0.4148089764,-1.814
4061283\H,-6.5982423074,-1.2397998905,-2.4252635661\C,-3.4551995668,-1
.1407581635,-3.7611093693\C,-2.7681941167,-0.3189611627,-4.8598930696\
C,-2.4930633485,-2.2524244565,-3.316173654\C,-4.718608902,-1.798997115
4,-4.3234889138\H,-3.432814396,0.4354415014,-5.2903591474\H,-1.8691869
94,0.1877129992,-4.4872226161\H,-2.4571845013,-0.9960204582,-5.6649303
022\H,-2.8866079093,-2.8534761507,-2.4920895753\H,-2.317909223,-2.9254
790132,-4.164680658\H,-1.5254888999,-1.844273482,-3.0053762116\H,-4.42
84073742,-2.4322328761,-5.1710768986\H,-5.2109919054,-2.446974157,-3.5
923926838\H,-5.4468098764,-1.0784995844,-4.7033418528\C,-4.3487706013,
1.6185211845,-3.0030257726\C,-5.5196471525,1.5824132889,-3.7803854475\
C,-3.6760089448,2.8516658763,-2.8596424934\C,-6.0231674236,2.715747554

1,-4.4046794141\H,-6.0527302745,0.6486184331,-3.9087823676\C,-4.196225
7667,3.9836469943,-3.503704065\C,-5.3541284364,3.9267254484,-4.2673605
788\H,-6.9308744219,2.6481820191,-4.9969631892\H,-3.6730635547,4.92858
41518,-3.3866559389\H,-5.7308357246,4.8237402659,-4.7500599306\C,-2.43
418796,3.0917668197,-2.0678769202\C,-1.2188095115,3.3117275009,-2.7294
969885\C,-2.4890517972,3.2834859916,-0.6807290402\C,-0.0874501351,3.71
49139398,-2.0225285193\H,-1.173586563,3.1953928804,-3.8096258387\C,-1.
358396525,3.6934880691,0.0243632486\H,-3.4337905801,3.152941932,-0.160
2836428\C,-0.1564911066,3.9142279087,-0.6450038915\H,0.8401357387,3.90
64167204,-2.5557973205\H,-1.4280192147,3.8706810607,1.0945086028\H,0.7
150327334,4.2665848808,-0.0996976436\C,1.3685125153,-0.1703652361,-0.4
702853539\C,1.5688671903,-1.0894974266,-1.7125348695\C,1.7924341277,0.
3300654642,-1.8951914184\H,2.4234085677,-1.7492959913,-1.590607021\H,0
.6645396228,-1.5485457631,-2.0964622528\H,1.057388642,0.9353314367,-2.
4154480443\H,2.8123916228,0.7057902444,-1.893143347\Br,1.6260019608,-3
.1879232607,1.2683217357\\Version=EM64L-G09RevA.01\State=1-A\HF=-4365.
9113147\RMSD=2.784e-09\RMSF=2.993e-06\Dipole=-0.1077159,0.6401364,-0.8
084731\Quadrupole=30.7922108,-14.7737845,-16.0184263,-7.2272189,19.098
7594,-3.4446493\PG=C01 [X(C34H40Au1Br1P1)]\\@

TS12

1\1\GINC-SHI_03\FTS\RPBE1PBE\GenECP\C34H40Au1Br1P1(1+)\YIN\22-Jul-2015
\0\\#p opt=(z-matrix,calcfc,ts,noeigen) genecp pbe1pbe\\Title Card Req
uired\\1,1\C,-1.3588221128,0.4936774713,-0.1573124798\H,-1.746219829,-
0.0239831917,-1.2118215256\C,-1.8703067047,1.3992604312,-1.1051713536\
C,-3.2297870574,1.8430012699,-0.8315763196\H,-1.3019618554,1.699400501
7,-1.9827948309\C,-3.667939009,1.0657341551,0.4258578179\C,-3.42980925
7,2.5297667461,0.511996755\H,-2.5511685697,2.8853317716,1.0439893714\H
,-4.2919949944,3.1896260858,0.5462660383\Au,0.6076780537,-0.1246043248
,-0.0111697925\H,-3.9135470545,2.0785358832,-1.640264922\C,-5.03878702
66,0.4990007573,0.5339344953\C,-5.4850514754,-0.5427103482,-0.29062571
6\C,-5.9243395074,0.9737355787,1.5075114065\C,-6.7571148185,-1.0869913
581,-0.160191306\C,-7.1998161332,0.4393976839,1.6531558052\H,-5.595362
9745,1.777548087,2.1612396369\C,-7.6157956867,-0.5920203194,0.81700913
83\H,-7.0716772325,-1.8874995715,-0.8212406191\H,-7.8658255898,0.82750
74727,2.4176555728\H,-8.6103933407,-1.0158967063,0.9181758666\P,2.7760
080526,-1.0269060683,0.1386645433\C,3.0149655011,-2.1208607853,-1.3915
393959\C,1.9906891459,-3.2598767231,-1.3397614509\C,2.7011415374,-1.21
61813719,-2.5928439564\C,4.4183309337,-2.7018102708,-1.5894000312\H,2.
2336118104,-3.9995492564,-0.5706932479\H,0.9738811371,-2.890291564,-1.
1614851286\H,1.9926341539,-3.7816107649,-2.3047085422\H,3.3864869394,-
0.363226019,-2.6519251012\H,2.8157692197,-1.799293344,-3.5151944251\H,
1.6740898705,-0.8342264918,-2.5599648937\H,4.3977475785,-3.3463936306,
-2.4769695023\H,5.1614783735,-1.9217089248,-1.7745723991\H,4.754330871

8,-3.3179567502,-0.751257534\C,2.918875298,-1.9519901208,1.7938641036\
C,3.1658276195,-0.8805533927,2.8642674802\C,1.570375884,-2.6264240947,
2.0897078274\C,4.0157040323,-3.0183440995,1.8706730256\H,4.145677571,-
0.4057205153,2.7595677898\H,2.3977149566,-0.0977527043,2.8372753264\H,
3.1218476479,-1.3523669306,3.8535356784\H,1.3032281176,-3.384914429,1.
3488128605\H,1.634342473,-3.1249170557,3.0650689359\H,0.7548644579,-1.
896393419,2.138566987\H,3.9968965414,-3.4594396401,2.8750439391\H,3.85
45605247,-3.8335149147,1.1592372672\H,5.0203283593,-2.6142830804,1.723
6665479\C,4.1374156152,0.2070537358,0.0826101092\C,5.4599924458,-0.253
5385942,0.2023197881\C,3.9236038543,1.5880866113,-0.1194604034\C,6.549
2137654,0.602611168,0.1113226937\H,5.6489555446,-1.3077866052,0.361904
2949\C,5.037011734,2.4340093798,-0.229094319\C,6.3360772344,1.95716651
26,-0.1176684571\H,7.5563421801,0.2091686808,0.2124310571\H,4.86380473
6,3.4945946751,-0.3888419481\H,7.174779529,2.6421505648,-0.2009714241\
C,2.6025448441,2.2795262987,-0.195561596\C,1.9238697748,2.6406847023,0
.9769226821\C,2.1228988883,2.7533083106,-1.4226979647\C,0.798352808,3.
4604238199,0.9225155747\H,2.310351295,2.3117738388,1.9381310372\C,0.99
37775089,3.5682443699,-1.4767368439\H,2.6583572043,2.5054687216,-2.335
6388683\C,0.3327600413,3.9302444527,-0.3039483727\H,0.3050727679,3.755
5935278,1.8449997279\H,0.6528485042,3.950409116,-2.4357637458\H,-0.524
6983151,4.5969546538,-0.344617706\C,-2.4548995073,0.2301456929,0.82755
27926\C,-2.5736361136,-1.1215207266,1.4867264917\C,-2.1109557983,0.039
5854832,2.2925057817\H,-3.5800782642,-1.4931062963,1.6558633043\H,-1.8
500978794,-1.8760428445,1.1910867434\H,-1.0629049913,0.1047987882,2.57
20962652\H,-2.804666792,0.4782604309,3.0054045651\Br,-4.3635810476,-1.
2507621821,-1.6469980029\\Version=EM64L-G09RevA.01\State=1-A\HF=-4365.
8869495\RMSD=7.606e-09\RMSF=4.654e-06\Dipole=0.0671803,0.5620538,0.307
7794\Quadrupole=40.9004861,-16.0953509,-24.8051352,-9.6570264,-6.09017
88,-1.3442141\PG=C01 [X(C34H40Au1Br1P1)]\\@

IN14

1\1\GINC-SHI_02\FOpt\RPBE1PBE\GenECP\C34H40Au1Br1P1(1+)\YIN\16-Jul-201
5\0\\#p opt genecp pbe1pbe\\Title Card Required\\1,1\C,-0.0225334521,-
0.0051689971,0.0488470403\C,0.0156447381,0.0252820658,1.4299820802\C,1
.4277107218,0.0213812697,1.9114809307\H,-0.8154589572,-0.2503762112,2.
0766959671\C,2.3071865851,-0.0960960395,0.6672880927\C,2.3394059557,1.
1503815707,1.4994035658\H,1.8924365301,2.0609389287,1.1023094866\H,3.2
186273554,1.3093937854,2.1182502173\Au,-0.7910739393,2.0783957356,0.80
21763821\H,1.6592960168,-0.5240814999,2.8207537182\C,3.5097378725,-0.9
744867951,0.6115890139\C,3.4348737453,-2.3705094365,0.7140272811\C,4.7
768320721,-0.4115892785,0.4210915516\C,4.5704400248,-3.1702453179,0.63
93119756\C,5.9212813413,-1.1968828056,0.3396460997\H,4.8541859571,0.66
97847457,0.3383761436\C,5.8162862469,-2.5798885061,0.4510246246\H,4.47
53169707,-4.2470051118,0.7304537889\H,6.8905222657,-0.7305480757,0.191

1056464\H,6.7015134629,-3.2063258975,0.3938286617\P,-1.7020712203,4.16
89205134,0.2463621188\C,-2.9909340189,3.8626767887,-1.1100148502\C,-2.
2793433625,3.3339728994,-2.359691599\C,-3.9214251562,2.7743602978,-0.5
545673127\C,-3.8485940125,5.076112928,-1.4824518121\H,-1.7024574704,4.
1128126693,-2.8681555312\H,-1.6096895735,2.4969204029,-2.1282163069\H,
-3.0335124731,2.9723518956,-3.0691908063\H,-4.4104971722,3.090821388,0
.3732921711\H,-4.7072198395,2.5710120424,-1.2924395418\H,-3.3895460008
,1.8356427748,-0.361869367\H,-4.5113484943,4.7837754899,-2.3062249776\
H,-4.4853608461,5.3964977118,-0.6537617383\H,-3.2617217102,5.930973087
9,-1.8278372998\C,-0.2813923449,5.3292153309,-0.2487302621\C,0.3948882
911,5.7551240993,1.0609420027\C,0.7318165311,4.5344995736,-1.085396007
8\C,-0.6892993325,6.5734214314,-1.0441026131\H,-0.2584266636,6.3723905
177,1.6848213543\H,0.7190727181,4.8886882381,1.6501401449\H,1.28907952
15,6.3435300956,0.8221497582\H,0.3189428145,4.1941432081,-2.0385364066
\H,1.5855326907,5.1859588627,-1.309653666\H,1.1102396838,3.6585610457,
-0.5477093609\H,0.2155235414,7.1575786331,-1.2529297114\H,-1.138526873
3,6.3244151117,-2.0098283453\H,-1.3675417913,7.2317664283,-0.496085737
9\C,-2.6188911584,4.9479791887,1.6318442196\C,-3.1782469535,6.22124361
86,1.4266055538\C,-2.8481630847,4.3019229516,2.8668703667\C,-3.9587774
32,6.8449215843,2.3907361736\H,-3.014652519,6.7365802773,0.4883878662\
C,-3.6608664694,4.9380644146,3.8169959849\C,-4.2132154859,6.1913534871
,3.591157141\H,-4.3707738293,7.8309406836,2.197946692\H,-3.8359978949,
4.4345653761,4.763536868\H,-4.8300104354,6.6575848218,4.3538187822\C,-
2.2690440542,3.0014577867,3.3130579384\C,-0.9291438108,2.9249129566,3.
7287935185\C,-3.1039005009,1.8977298247,3.5326384906\C,-0.4487675967,1
.7836588019,4.3700000115\H,-0.2851378142,3.7924325473,3.6089515093\C,-
2.6180723235,0.7535877562,4.1626890931\H,-4.1480074052,1.951991433,3.2
351052346\C,-1.2938809625,0.6989793128,4.5952755485\H,0.5811743249,1.7
556744709,4.714366511\H,-3.2850161687,-0.085331679,4.3415882821\H,-0.9
26772196,-0.179920998,5.1181098809\C,1.3428310501,-0.0690334548,-0.518
7045646\C,1.5666668566,-0.8965864031,-1.7679378007\C,1.7066820128,0.58
21594131,-1.8373812498\H,2.4680518749,-1.5016315731,-1.7898218701\H,0.
6966743489,-1.3656455203,-2.2187255221\H,0.9401184346,1.1597608571,-2.
3474600936\H,2.7092262838,0.9974095592,-1.9037395602\H,-0.9058246654,-
0.2272545062,-0.5470112097\Br,1.7679425488,-3.2327902658,0.9662654816\
\Version=EM64L-G09RevA.01\State=1-A\HF=-4365.9342053\RMSD=7.184e-09\RM
SF=3.854e-06\Dipole=-0.6759993,1.9216635,-0.3137583\Quadrupole=9.59391
86,5.8134667,-15.4073853,-24.3683526,-5.1249904,-2.5179402\PG=C01 [X(C
34H40Au1Br1P1)]\\@

IN15

1\1\GINC-SHI_02\FOpt\RPBE1PBE\GenECP\C41H49Au1Br1N2(1+)\YIN\26-Jan-201
6\0\\#p genecp pbe1pbe opt\\Title Card Required\\1,1\C,-0.7015080595,-
0.2638775194,0.2374619266\C,-0.4842651122,-0.0328496722,1.5898893523\C

,0.8091234559,-0.1113970855,2.1318407367\C,1.8811243518,-0.41833148,1.
2789544628\C,1.664569716,-0.6477679391,-0.0722805259\C,0.3738707485,-0
.5703645816,-0.5913838415\H,-1.7091001554,-0.2074070918,-0.1600140303\
H,2.8817395121,-0.4785157481,1.6959229744\H,2.5018374604,-0.8903297805
,-0.7190095492\H,0.197534364,-0.7516817014,-1.6473897679\C,1.058233209
3,0.0913545579,3.5323852232\C,1.3933363713,-0.0478896217,4.7197187143\
C,1.7750133355,-0.4764304478,6.0428072267\C,3.0058735924,-1.3660729587
,6.1137457732\C,3.0893803139,0.0383182635,6.6033560029\H,3.5056410292,
-1.6207544035,5.1845758882\H,2.9488438833,-2.1762734944,6.8356580415\H
,3.1081260103,0.2168591594,7.6748333033\H,3.6426605003,0.7639541624,6.
0137728107\C,0.6210352054,-0.7528525789,7.0052922742\H,0.0359661122,0.
1715985924,7.1200737529\H,1.0508987044,-0.9804197542,7.9880667407\C,-0
.2643388237,-1.878224146,6.5618469765\H,-0.7699233212,-1.7460568821,5.
6043191233\C,-0.4579631661,-2.9967336259,7.2570125658\H,0.021709805,-3
.1632767594,8.219836963\H,-1.1113037201,-3.7859884674,6.8965455311\Au,
0.8002315155,2.1258361135,4.3914046555\Br,-1.9471262141,0.3854701354,2
.6970800503\C,0.9283940646,6.2297667927,5.305513498\C,-0.3272552352,6.
1713028307,4.7940699504\H,1.5020136749,7.0539182042,5.7003816728\H,-1.
0756216106,6.9347319124,4.6478474029\N,1.437426752,4.949544452,5.25095
15855\N,-0.5528427097,4.857162315,4.4412056342\C,0.5302329855,4.097910
9221,4.7194542679\C,2.7466029479,4.5699552121,5.711884927\C,2.91634201
99,4.3021910092,7.0800996101\C,3.7931981475,4.5025180199,4.7776471388\
C,4.1966442529,3.938592114,7.5035503529\C,5.0504349223,4.1279366001,5.
2580190631\C,5.2517849909,3.8478316787,6.6039639201\H,4.3701210421,3.7
296393479,8.5556347626\H,5.8872463061,4.0675717045,4.5675659386\H,6.24
06519286,3.5675279018,6.9560973258\C,-1.7742600089,4.384765176,3.84292
72606\C,-2.7769680874,3.8730413104,4.6818313053\C,-1.9158801824,4.4942
269978,2.4501940495\C,-3.9627479491,3.4553902228,4.0729934949\C,-3.124
4917935,4.065871153,1.8982344629\C,-4.1376728699,3.5530903213,2.698217
3667\H,-4.764281335,3.0559584154,4.6884435115\H,-3.2739836529,4.137203
7546,0.8245189191\H,-5.0717091293,3.2294283457,2.2475827457\C,3.614257
5958,4.8386281572,3.3087720944\C,3.9462582671,3.6421033033,2.412520221
7\C,4.446749755,6.0657367305,2.9210344659\H,2.5613651134,5.0910545796,
3.1378603021\H,3.3255100797,2.7740628417,2.663927619\H,3.7693547063,3.
8957712887,1.3612978696\H,4.9972220752,3.3469079216,2.5099189915\H,4.1
943127071,6.9340866954,3.5388494822\H,5.519532481,5.8752724568,3.03725
19322\H,4.2682155166,6.3297948256,1.8729477916\C,1.7878937647,4.411600
6311,8.0893484061\C,2.0479641516,5.5516569882,9.0801558593\C,1.5545815
233,3.0882738706,8.8232969698\H,0.8631425237,4.6483295182,7.5505518752
\H,2.1807492363,6.5102108303,8.5668397259\H,1.2076978575,5.6520915202,
9.7757681528\H,2.9507810303,5.3655225715,9.6725653267\H,1.3401386934,2
.2771695232,8.118836036\H,2.4275771132,2.7980435379,9.4189709359\H,0.7
051496691,3.180557686,9.5090876834\C,-0.8285608162,5.0522047175,1.5500
223738\C,-1.2944317076,6.3278862164,0.8407208794\C,-0.3483271939,4.003

7354814,0.5416292939\H,0.0327024883,5.3202735021,2.1730895536\H,-1.610
6494851,7.0944532032,1.5564406012\H,-0.4832850428,6.7434754668,0.23295
35259\H,-2.1401386164,6.1283255273,0.1733317796\H,0.022592621,3.104431
2645,1.0457264997\H,-1.1549966972,3.699745984,-0.1348991753\H,0.462193
1179,4.4124987368,-0.0722623343\C,-2.6228605345,3.7708262656,6.1877941
381\C,-2.7076677864,2.3145140322,6.6552757333\C,-3.6512364083,4.645161
0878,6.9125163369\H,-1.6272939235,4.141423572,6.4589032384\H,-1.960112
2434,1.6924432923,6.1496316946\H,-2.5373305824,2.2509579736,7.73599593
11\H,-3.6943533463,1.8839951493,6.4506182807\H,-3.5747677361,5.6938659
659,6.6059045295\H,-4.674510486,4.3121094674,6.7069881853\H,-3.4978281
338,4.5948683806,7.9961098267\\Version=EM64L-G09RevA.01\State=1-A\HF=-
4405.9155984\RMSD=5.434e-09\RMSF=2.237e-06\Dipole=0.9040871,0.5965251,
-0.043093\Quadrupole=-2.9374274,-2.5263442,5.4637716,-4.6209771,7.3139
379,5.541583\PG=C01 [X(C41H49Au1Br1N2)]\\@

TS13

1\1\GINC-SHI_03\FTS\RPBE1PBE\GenECP\C41H49Au1Br1N2(1+)\YIN\28-Jan-2016
\0\\#p genecp pbe1pbe opt=(calcfc,ts,noeigen)\\Title Card Required\\1,
1\C,0.6904626439,-3.5183664402,-1.7504625764\C,-0.7414044295,-3.047127
5813,-1.8026937264\C,0.1450160025,-3.0120662379,-3.0410269227\H,1.4063
412158,-2.9056424324,-1.2099737758\H,0.8387602324,-4.5901343224,-1.643
9756973\H,-0.0809864745,-3.7262015127,-3.8283345947\H,0.4709894099,-2.
0378288558,-3.3933949514\C,-1.1426470855,-1.7480903031,-1.2201421781\C
,-2.355681687,-1.3085594904,-1.1457750018\C,-3.3689600578,-0.417494238
8,-0.6681737573\C,-3.7236946845,0.7131533104,-1.4278434318\C,-4.037629
0987,-0.6387466552,0.5530953653\C,-4.6958849855,1.5947213034,-0.983326
8621\C,-5.0234833204,0.2360203226,0.9931301514\C,-5.3493571452,1.34969
82364,0.2243891096\H,-5.5304947901,0.0442979352,1.9325804137\H,-6.1232
335277,2.0271902078,0.5737094278\C,-1.8321195587,-4.1082224914,-1.6559
889865\H,-1.7314680275,-4.6004155948,-0.6822336455\H,-1.6891156626,-4.
8866440574,-2.4191058383\C,-3.197652615,-3.5216671447,-1.7738740505\C,
-3.4959475589,-2.5406959166,-2.6594114284\H,-3.961624478,-3.8532647687
,-1.0740719713\H,-4.4924817243,-2.1119298192,-2.7046789858\H,-2.799070
5086,-2.2511150442,-3.4418660393\Au,0.1759070498,-0.3509098064,-0.4177
520658\H,-3.2089507542,0.8846735604,-2.3683559144\H,-4.9500976221,2.46
6797422,-1.5773293384\Br,-3.6122702579,-2.1256626102,1.6208477743\C,3.
4322190411,1.732604621,1.2938766981\C,2.6119768507,2.7869795506,1.0595
79272\H,4.4177628158,1.6761857728,1.7295002258\H,2.7326924462,3.842382
8053,1.2487230079\C,1.5520959326,0.9326371766,0.3472766947\N,1.4674848
435,2.2770265561,0.4793709647\N,2.7664203271,0.6080819082,0.8496741577
\C,3.2967772611,-0.7280057936,0.896437221\C,4.0850463954,-1.1685093013
,-0.1794957383\C,3.0077179222,-1.5253199586,2.0154489449\C,4.575033968
6,-2.4749646373,-0.1175429996\C,3.5265289754,-2.8221312574,2.024459785
5\C,4.2978384651,-3.2945595798,0.9700043205\H,5.1876830294,-2.85299157

89,-0.9313920511\H,3.3259789717,-3.4690803883,2.874032204\H,4.69371414
17,-4.3059239534,0.9998920381\C,0.3400106072,3.073449435,0.0762132378\
C,-0.6670567202,3.3282339626,1.0203660191\C,0.30762847,3.5710549722,-1
.236475851\C,-1.7494080619,4.1060575142,0.6034779215\C,-0.7984223344,4
.3435201079,-1.5979386429\C,-1.8163595738,4.608200277,-0.6900829451\H,
-2.5476538446,4.3257920783,1.3070603571\H,-0.8579194391,4.7495841497,-
2.6041234424\H,-2.662659407,5.220328073,-0.9902083391\C,-0.6174653032,
2.7956527847,2.4403232629\C,-1.7438877511,1.7882784378,2.6900105188\C,
-0.6517212015,3.9317319825,3.4674186719\H,0.3308530676,2.2627998528,2.
5748445298\H,-1.6904363522,0.9509755019,1.985224397\H,-1.6756026751,1.
3850341623,3.7067761545\H,-2.7286857898,2.2572382062,2.5816468799\H,0.
1650344312,4.6437795875,3.3075957715\H,-1.5935310549,4.4898700487,3.42
02614492\H,-0.557984612,3.5284854925,4.4816463872\C,1.4064133688,3.298
0654836,-2.2466704977\C,2.0282484489,4.5971187091,-2.7684353332\C,0.89
08202356,2.4295189625,-3.3984301972\H,2.2012316241,2.7349270803,-1.744
4129527\H,2.413949783,5.2149423567,-1.9505344604\H,2.8588279887,4.3737
047274,-3.4468511415\H,1.3007592501,5.1978978149,-3.325514639\H,0.4881
407989,1.4802641692,-3.0260562644\H,0.0972190026,2.9395424281,-3.95677
99348\H,1.7018982161,2.2070049809,-4.1011689565\C,4.407942314,-0.29215
08105,-1.375788465\C,3.7442968079,-0.8294780259,-2.6475427087\C,5.9197
731747,-0.1342476318,-1.5651648566\H,3.9960694978,0.7066839684,-1.1912
795513\H,2.6556689586,-0.8817638787,-2.5309158325\H,3.9665930779,-0.17
67118047,-3.4993773969\H,4.1065756507,-1.8343930786,-2.8929707875\H,6.
3986183264,0.2621359319,-0.6634867273\H,6.3984218844,-1.0904665054,-1.
803504204\H,6.1280691997,0.5533391809,-2.3920811656\C,2.1782169204,-1.
0329661731,3.1863395747\C,2.9963627285,-1.0290072072,4.4819229098\C,0.
8958658755,-1.8557693458,3.3445056658\H,1.8787015458,0.0019402397,2.98
44303462\H,3.9016045537,-0.4200795303,4.3845267769\H,2.4002084348,-0.6
231803298,5.3066037836\H,3.305228193,-2.0417961731,4.7638999659\H,0.29
54051051,-1.8340039097,2.4279740796\H,1.1195635509,-2.9029764291,3.578
4047101\H,0.2865281409,-1.4559457433,4.1629586746\\Version=EM64L-G09Re
vA.01\State=1-A\HF=-4405.8976222\RMSD=3.789e-09\RMSF=2.644e-06\Dipole=
-0.6921651,0.1382201,-0.7303884\Quadrupole=14.0526841,1.9460516,-15.99
87357,-2.6427444,6.4897737,5.0709238\PG=C01 [X(C41H49Au1Br1N2)]\\@

IN16

1\1\GINC-A718\FOpt\RPBE1PBE\GenECP\C41H49Au1Br1N2(1+)\ROOT\27-Jan-2016
\0\\#p genecp pbe1pbe opt\\au_ph_br_int2_up_jp\\1,1\C,-0.3045569088,-0
.0667155524,-0.0358147506\C,-0.0733331664,-0.1063110655,1.4841620684\C
,1.0743665755,-0.1726435103,0.4389054844\H,-0.7827881963,-0.9393271716
,-0.4712074373\H,-0.6615804533,0.8955325036,-0.3931979786\H,1.70594106
92,0.7112986653,0.434992318\H,1.5958239536,-1.1206480502,0.3522565187\
C,-0.370909294,-1.3143534875,2.2135519448\C,-0.5630546838,-0.974247557
6,3.612903373\C,-1.428976392,-1.7817615179,4.5143590167\C,-0.919445281

1,-2.4863714484,5.6088406177\C,-2.8145601776,-1.8281597801,4.305109353
6\C,-1.7548440198,-3.181418845,6.4769778781\C,-3.6634177412,-2.5161856
126,5.1637196252\C,-3.1288591546,-3.1889435528,6.2589064539\H,-4.73236
95541,-2.5169778691,4.9791529679\H,-3.7920140931,-3.7179389869,6.93698
21377\C,-0.3245559857,1.1305882157,2.3427051934\H,-1.2684796987,1.6067
988513,2.0548199787\H,0.471455248,1.8790874287,2.2560977724\C,-0.42311
01102,0.5675001106,3.7396455813\C,0.6968092141,-0.2754087204,4.1823024
992\H,-1.0314657805,1.0653178908,4.488620675\H,0.8270760761,-0.4320570
528,5.2489266388\H,1.6117825564,-0.3206724938,3.597514735\Au,-0.429108
9898,-3.1776475062,1.4442857096\H,0.1537080252,-2.4907006507,5.7785136
71\H,-1.3313047098,-3.7120117616,7.3243129059\Br,-3.5799264808,-0.9192
696566,2.8302014463\C,-0.6111551789,-6.741169175,-0.8674707822\C,-0.00
17077989,-7.2367912013,0.2387023784\H,-0.902629832,-7.2119716256,-1.79
35066095\H,0.3524256345,-8.2280012489,0.4761387497\C,-0.3991171944,-5.
0503147052,0.6055826069\N,0.117124455,-6.1881326841,1.126919969\N,-0.8
422133676,-5.4031749977,-0.6251033403\C,0.696938293,-6.3004115213,2.43
84566774\C,-0.1248352795,-6.7365147315,3.4900183344\C,2.0564594303,-5.
9898865076,2.6016554707\C,0.4598530827,-6.8393188455,4.7540321861\C,2.
5867014542,-6.1089054694,3.8881828996\C,1.7986189059,-6.5263936472,4.9
535540464\H,-0.1432272645,-7.1745404755,5.5935250283\H,3.635770434,-5.
8800622102,4.0549085383\H,2.2343513732,-6.619216177,5.9445950099\C,-1.
4800449726,-4.5097492787,-1.5553577129\C,-0.6833397966,-3.8699158698,-
2.5200018927\C,-2.8714792783,-4.3408074399,-1.4713294464\C,-1.33002550
54,-3.013758958,-3.4138682749\C,-3.4634495651,-3.4698067648,-2.3893322
505\C,-2.7035265402,-2.8109849361,-3.3473040313\H,-0.7507866667,-2.505
652164,-4.1796906501\H,-4.5382245766,-3.313992961,-2.3576002203\H,-3.1
864217076,-2.1435818429,-4.0557355033\C,2.9475046028,-5.556496707,1.45
2528588\C,4.0938599311,-6.5492957933,1.2338406078\C,3.4815798556,-4.13
67409303,1.666286702\H,2.3456378736,-5.5438980651,0.536625119\H,3.7184
534621,-7.5629327994,1.0578575766\H,4.6938086832,-6.2530406057,0.36627
86111\H,4.7622669226,-6.5875345645,2.1011944466\H,2.6589338185,-3.4214
487065,1.7867419965\H,4.114103183,-4.0775992753,2.5594644952\H,4.08836
03044,-3.8249336291,0.8083754689\C,-1.5866128985,-7.095377793,3.297882
5336\C,-2.4979225326,-6.1655930532,4.1031143736\C,-1.8485907225,-8.564
5544849,3.6450604477\H,-1.8386557006,-6.9596964818,2.2399545538\H,-2.3
359553234,-5.1158258161,3.8365797453\H,-3.5495174048,-6.4106808107,3.9
150113384\H,-2.3209313884,-6.2670082757,5.1797518603\H,-1.2184345808,-
9.2375613415,3.0535640119\H,-1.6499687674,-8.7679278671,4.7032552843\H
,-2.8959003926,-8.8199804484,3.4499442793\C,0.8089172571,-4.1174234931
,-2.6423928823\C,1.6125833746,-2.8166521352,-2.6998983808\C,1.11018474
23,-4.9966033831,-3.861955703\H,1.1390221937,-4.6619855855,-1.75007708
84\H,1.3948429567,-2.1755539636,-1.8392242633\H,2.6857103609,-3.037320
7129,-2.701352214\H,1.3977727379,-2.2433545303,-3.6084907129\H,0.58085
17317,-5.9541907291,-3.8109555318\H,0.8058093999,-4.4990758625,-4.7898

302354\H,2.1836594648,-5.2053993328,-3.9289811193\C,-3.7331988722,-5.0
851952211,-0.4687362526\C,-4.6488746792,-6.0866857575,-1.1820377888\C,
-4.5425202637,-4.1291208215,0.4110305667\H,-3.0742616496,-5.657223581,
0.1943102044\H,-4.075379451,-6.8010604457,-1.7827703429\H,-5.237658875
5,-6.6518406879,-0.4511404101\H,-5.3500313681,-5.5765507326,-1.8520354
206\H,-3.8943039798,-3.4243423219,0.9425718721\H,-5.2603443716,-3.5480
637986,-0.1790856574\H,-5.1134439555,-4.6960189531,1.1550604682\\Versi
on=ES64L-G09RevD.01\State=1-A\HF=-4405.9479342\RMSD=3.599e-09\RMSF=1.7
58e-06\Dipole=0.9500041,0.317187,0.0662478\Quadrupole=-12.7032081,4.90
73496,7.7958586,3.1911455,1.0876646,0.4248987\PG=C01 [X(C41H49Au1Br1N2
)]\\@

TS14

1\1\GINC-A765\FTS\RPBE1PBE\GenECP\C41H49Au1Br1N2(1+)\ROOT\01-Feb-2016\
0\\#p opt=(calcfc,ts,noeigen) genecp pbe1pbe\\Title Card Required\\1,1
\C,0.0979834626,-2.7791669977,-2.4783111331\C,0.2691058693,-3.81000407
45,-1.3420911257\H,-0.3051789134,-3.2050350407,-3.3953652599\H,0.94684
62864,-2.1277394308,-2.6653427458\H,1.0274452181,-3.5009104803,-0.6231
15964\H,0.3380018832,-4.8659758067,-1.6008912088\C,-1.0839125456,-3.26
96091864,-1.1427821941\C,-2.4120463551,-3.9385142552,-1.2807568236\C,-
3.2989430096,-2.8660765014,-1.8760462487\H,-2.7263790989,-4.2291510391
,-0.2657736459\H,-2.3561493713,-4.8673091899,-1.8631378778\C,-2.459412
9722,-1.5942549955,-2.0573614335\C,-2.9610439954,-2.3800046998,-3.2458
975156\H,-2.2652923545,-2.9443533293,-3.8618816047\H,-3.7699725644,-1.
9252589811,-3.8102888377\C,-1.059657919,-1.919985749,-1.5554319688\Au,
0.0714197285,-0.5131729285,-0.4635734658\H,-4.3257027038,-2.7883334159
,-1.5342630824\C,-3.0372453332,-0.241471739,-1.8053369137\C,-3.4602012
515,0.1738478265,-0.5368949928\C,-3.187173866,0.6677946854,-2.85764391
14\C,-4.009701884,1.4318975402,-0.319119179\C,-3.7357900048,1.93001128
89,-2.660530262\H,-2.8680850027,0.3647520695,-3.8517077394\C,-4.148115
137,2.3115872068,-1.3878933074\H,-4.3297834345,1.7152675985,0.67764003
76\H,-3.8460610557,2.6098664697,-3.5001546248\H,-4.584895364,3.2913708
33,-1.2200168515\Br,-3.3221335639,-0.9931415583,0.9543342552\C,3.17390
52361,1.2750276388,1.742654809\C,2.3534636418,2.3526406135,1.709065382
9\H,4.1509491288,1.1328000319,2.177883478\H,2.4669647749,3.3493895574,
2.1056553136\C,1.3107215699,0.6839237831,0.6137585344\N,2.5198040219,0
.2654822384,1.0659506788\N,1.2180597248,1.9720703382,1.0190500973\C,3.
0521523034,-1.0513808379,0.8465084037\C,2.7340326959,-2.0659979867,1.7
639963\C,3.8709020669,-1.2603779143,-0.2763004865\C,3.2447386638,-3.34
15651601,1.5075058768\C,4.3505013884,-2.555642643,-0.4850743807\C,4.03
92661882,-3.586928069,0.3937096442\H,3.0252702622,-4.1507503182,2.1987
679151\H,4.9866919788,-2.7565974021,-1.3426188126\H,4.4322823466,-4.58
46696785,0.2179507513\C,0.1155003858,2.8654578469,0.7813421542\C,-0.78
36984301,3.1044014598,1.8346421793\C,0.0299974727,3.5108555973,-0.4627

615817\C,-1.8012498497,4.0327202607,1.6051733728\C,-1.0159226181,4.419
5735198,-0.6384060352\C,-1.9180505022,4.683245608,0.3835297827\H,-2.50
74749286,4.2549822755,2.4001812872\H,-1.1135890423,4.9398166666,-1.587
2553112\H,-2.7127206767,5.4085034815,0.230459898\C,4.2315766185,-0.149
4440588,-1.2459640257\C,3.5239570244,-0.3408530521,-2.591339843\C,5.74
66770656,-0.0263319132,-1.432516757\H,3.8786812975,0.7994960137,-0.826
2063243\H,2.434697355,-0.3401359057,-2.4619504451\H,3.7826651216,0.471
459034,-3.2798957211\H,3.8181732928,-1.285375703,-3.0644702477\H,6.259
3177464,0.1117556394,-0.4748159317\H,6.1716045605,-0.9139645231,-1.914
0374292\H,5.9786097602,0.8338713871,-2.0696428579\C,1.889873464,-1.823
5148871,3.0013861174\C,2.6953420419,-2.0889900869,4.2777321879\C,0.604
6945511,-2.6565610381,2.9758633489\H,1.5923428408,-0.7690906422,3.0125
573295\H,3.6030929141,-1.4773417135,4.3152504863\H,2.0919542529,-1.857
0050084,5.1620630402\H,2.99834791,-3.1395146299,4.3495072035\H,-0.0013
644955,-2.4182552356,2.0939614413\H,0.8230703126,-3.730917376,2.964960
1996\H,0.0021628229,-2.4506414586,3.8675125194\C,1.0244654672,3.282659
9241,-1.5860420641\C,1.7624660548,4.5758443031,-1.9468014684\C,0.34357
33151,2.6719165436,-2.8143668484\H,1.7755711322,2.5629402899,-1.241691
929\H,2.275615122,5.0014017249,-1.0777904554\H,2.5108304525,4.38165525
01,-2.7232444366\H,1.0752272367,5.3365914602,-2.3333874652\H,-0.155301
9622,1.7294996355,-2.5604071101\H,-0.4114923076,3.3481751989,-3.231278
9474\H,1.0834132011,2.474412559,-3.5987761678\C,-0.6608568895,2.436324
9664,3.1924973723\C,-1.9704511626,1.7779488872,3.634263952\C,-0.177058
5131,3.4386432031,4.2474431959\H,0.0898438888,1.6407343176,3.117581006
5\H,-2.325898776,1.0541088378,2.8941505657\H,-1.8217487574,1.251322585
6,4.5836372788\H,-2.7621961209,2.51815962,3.7961314752\H,0.7829711972,
3.8898329696,3.9734348555\H,-0.899584959,4.2528151656,4.3748027503\H,-
0.0547676274,2.9446916887,5.2177125231\\Version=ES64L-G09RevD.01\State
=1-A\HF=-4405.9200479\RMSD=3.460e-09\RMSF=2.690e-06\Dipole=0.746898,-1
.1716117,-0.8021019\Quadrupole=-3.5790915,12.9345228,-9.3554313,-4.434
3006,7.9425091,9.3628909\PG=C01 [X(C41H49Au1Br1N2)]\\@

IN17

1\1\GINC-SHI_03\FOpt\RPBE1PBE\GenECP\C41H49Au1Br1N2(1+)\YIN\26-Jan-201
6\0\\#p opt genecp pbe1pbe\\Title Card Required\\1,1\C,-0.3501938314,0
.2683832733,-0.6418282848\C,0.0741422256,0.5122542044,0.8420986401\H,0
.4853768144,0.3511361173,-1.3453474121\H,-1.1968818307,0.8392119605,-1
.0299648185\H,-0.4459839484,1.2810115586,1.4182080106\H,1.1548035582,0
.6772687118,0.9300283471\C,-0.2791702858,-0.9587519181,1.0979836759\C,
0.2574306667,-2.1978669855,1.7889797958\C,0.3423967909,-3.1845893272,0
.620147834\H,-0.3803081509,-2.5535109727,2.605853985\H,1.2479408839,-2
.0010863069,2.21853228\C,-0.2430901465,-2.5615713412,-0.6648590033\C,1
.2499426855,-2.7915630262,-0.4985547902\H,1.9090305069,-1.9340868435,-
0.3695528154\H,1.6684640901,-3.5900149646,-1.1048210824\C,-0.602762766

4,-1.1857973993,-0.2275121137\Au,-2.5233347094,-1.0195107164,1.0472420
594\H,0.1868038585,-4.2413347793,0.8111159894\C,-0.9501856111,-3.30305
09986,-1.750659984\C,-2.0912688708,-4.0876003452,-1.5611581875\C,-0.44
80640635,-3.220367942,-3.0560640194\C,-2.6974917107,-4.7759968646,-2.6
08248579\C,-1.0481025388,-3.8848070832,-4.1183690239\H,0.4447023712,-2
.6237474561,-3.2261032556\C,-2.1741844576,-4.6710668536,-3.8919651917\
H,-3.5703219563,-5.3900442176,-2.4135463707\H,-0.6306096667,-3.7986856
248,-5.1169522829\H,-2.645341693,-5.2098059638,-4.7086820903\Br,-2.868
0357663,-4.2739420516,0.1599694176\C,-6.1728627,-0.6458854412,3.155833
9986\C,-6.5389256746,-0.0753953219,1.9817009965\H,-6.7097065217,-0.777
5314337,4.0820129762\H,-7.4631640662,0.3846397784,1.6689921294\C,-4.40
98322533,-0.7618658183,1.7672917041\N,-5.4463144494,-0.1565657265,1.14
32990176\N,-4.8673647734,-1.067951338,3.0045072573\C,-5.4062475402,0.3
99447382,-0.1846102123\C,-4.9951671522,1.7344234801,-0.329274516\C,-5.
8137938239,-0.4034418753,-1.2639112027\C,-4.9808453693,2.2599256099,-1
.6234397556\C,-5.781286484,0.1769834782,-2.5333591404\C,-5.3666641119,
1.4912438353,-2.7133803226\H,-4.6728810852,3.290514415,-1.7768417961\H
,-6.0885274566,-0.4072812727,-3.3950899686\H,-5.3531906579,1.921367895
7,-3.7109467499\C,-4.0835262494,-1.6472090464,4.0660348987\C,-4.141341
5558,-3.0373930262,4.2718117572\C,-3.3287138618,-0.7818380965,4.875025
6927\C,-3.3865292649,-3.5543210472,5.3265806752\C,-2.5923054595,-1.357
2463823,5.9137218745\C,-2.6180833897,-2.7267350151,6.1370807199\H,-3.4
017059975,-4.6221950093,5.5197523271\H,-1.9996258518,-0.7186237581,6.5
627447932\H,-2.0426772592,-3.1536291369,6.9537628716\C,-6.323150645,-1
.8200981031,-1.0721329218\C,-5.8421838336,-2.7780989075,-2.1628153939\
C,-7.8554007969,-1.8314312576,-0.9914423832\H,-5.9309564188,-2.1946926
312,-0.1185914654\H,-4.7553086975,-2.7411042183,-2.284606765\H,-6.1214
503843,-3.8042934059,-1.9001109267\H,-6.3028079109,-2.5579470104,-3.13
24007585\H,-8.2292826821,-1.2065416807,-0.1733991372\H,-8.2947045997,-
1.4571700548,-1.9231975542\H,-8.2223856651,-2.8512662003,-0.830993022\
C,-4.6128544045,2.6161939486,0.8456983065\C,-5.6453939926,3.7311750508
,1.0466050462\C,-3.2045860358,3.1951195449,0.6887982516\H,-4.610674269
4,2.0020897216,1.7533816705\H,-6.6506615482,3.3253284762,1.2031658398\
H,-5.3852487033,4.3410717513,1.9187421829\H,-5.6872501004,4.3943753405
,0.1753894957\H,-2.4623810552,2.3992098662,0.5618236999\H,-3.134318062
,3.8624122806,-0.1773118589\H,-2.9334177289,3.7789449183,1.5756265485\
C,-3.317373884,0.7247459276,4.6924400726\C,-1.8980313047,1.264558574,4
.4979282963\C,-4.0127098706,1.4191308018,5.8689450448\H,-3.8827643059,
0.9682950343,3.785757489\H,-1.4022655589,0.7763819098,3.6514832571\H,-
1.9265061711,2.3430753001,4.3056943074\H,-1.2782407215,1.1066322062,5.
3874661349\H,-5.0430979671,1.0684139824,5.9917814088\H,-3.4832924366,1
.2307202153,6.8096662398\H,-4.0385443489,2.503072562,5.711613492\C,-5.
0154277697,-3.9494020997,3.4293451258\C,-6.4017187057,-4.1147093163,4.
0672906826\C,-4.3904174737,-5.3270953759,3.1968903612\H,-5.1493688812,

-3.4752835957,2.4482064185\H,-6.9253371018,-3.1598244978,4.1757008805\
H,-7.027648206,-4.772251312,3.4539234281\H,-6.3147296262,-4.5635597331
,5.0632269179\H,-3.3507829551,-5.2578554653,2.8596258094\H,-4.40605003
78,-5.9380279534,4.1063080671\H,-4.9609826376,-5.8717972579,2.43742416
04\\Version=EM64L-G09RevA.01\State=1-A\HF=-4405.9464796\RMSD=6.140e-09
\RMSF=3.041e-06\Dipole=0.2796885,0.6846534,-0.0269011\Quadrupole=1.203
2418,-9.5821594,8.3789176,-2.4026823,-5.4130333,2.8324541\PG=C01 [X(C4
1H49Au1Br1N2)]\\@

TS15

1,1\C,0.4667355314,-4.1217556151,2.002685532\C,-1.0583566807,-4.471813
8531,1.8841966922\H,1.1361047318,-4.9877360457,1.9491228752\H,0.728667
387,-3.5214680477,2.8807560078\H,-1.6899816261,-4.094618464,2.69618613
39\H,-1.2563160593,-5.5412664023,1.7518319964\C,-1.0294966418,-3.64953
6278,0.6181607796\C,-1.7780111005,-3.171462922,-0.5827192622\C,-0.6946
189782,-2.5700092813,-1.4646806801\H,-2.5827232125,-2.4625651684,-0.35
33857961\H,-2.2305273207,-4.0134197886,-1.1318659929\C,0.9161149958,-2
.6999434576,-0.4720943348\C,0.4852639425,-3.4452139558,-1.7097454599\H
,0.3357825939,-4.5158539335,-1.5601613665\H,1.0414341543,-3.21986278,-
2.6157844436\C,0.2752817762,-3.3616177636,0.6996232016\Au,-0.185131549
9,-0.6061123086,-0.2324092041\H,-0.9558913507,-1.8725758751,-2.2599549
672\C,2.2674262332,-2.0562003496,-0.4133447258\C,3.0490651877,-2.31545
67746,0.7245604754\C,2.8759437801,-1.3066574188,-1.4361086819\C,4.3689
067587,-1.8952634675,0.8229683377\H,2.6154413355,-2.8830001875,1.53980
71188\C,4.1959296118,-0.881135332,-1.3457542022\C,4.9510561237,-1.1822
872324,-0.2181012138\H,4.9413263151,-2.1347127303,1.7140227852\H,4.625
1843643,-0.3094120734,-2.1616282709\H,5.9842044072,-0.8541616713,-0.15
86623295\Br,1.929220136,-0.7530396607,-2.9865713522\C,-0.808717208,3.3
125501831,1.2670886989\C,-2.0616917093,2.997483836,0.8579234466\H,-0.4
117004264,4.2008055001,1.7330025184\H,-2.9855841962,3.5540145651,0.887
1426455\C,-0.7343849498,1.2331363921,0.4131209675\N,-1.9965226627,1.72
05710574,0.3399326507\N,-0.0086869114,2.222879721,0.9842460077\C,-3.12
3902467,0.9923536313,-0.1759123129\C,-3.8943879946,0.2380042299,0.7247
498292\C,-3.4072158187,1.0780460167,-1.548744668\C,-4.9679874798,-0.48
25775014,0.1956748928\C,-4.4940459282,0.3379310334,-2.0210051378\C,-5.
2618402986,-0.4392782948,-1.1621982798\H,-5.5888602894,-1.0777807197,0
.8599012732\H,-4.74935597,0.3805430156,-3.076296505\H,-6.1053971111,-1
.002719852,-1.5512973723\C,1.3930878325,2.1668496622,1.3061777177\C,2.
307614792,2.7565065427,0.4170864752\C,1.7725274657,1.5742649236,2.5213
322625\C,3.6566872843,2.713120658,0.7739694631\C,3.1363199009,1.553863
1851,2.8211169931\C,4.0685882807,2.1133049394,1.9574735261\H,4.3955987
518,3.1637788339,0.1179176674\H,3.4697867121,1.1058282375,3.7531516968
\H,5.1239136103,2.0953414027,2.2153128333\C,-3.6157938204,0.1985111401
,2.216659608\C,-3.2190414637,-1.2069312164,2.6776093123\C,-4.812049165

2,0.7250356877,3.016421161\H,-2.7669869511,0.8593518094,2.4261264344\H
,-2.3183475887,-1.5532996139,2.1583246948\H,-3.0156233235,-1.208624337
1,3.754594501\H,-4.0211280665,-1.93046,2.4904877448\H,-5.0902934746,1.
7374185376,2.7047596395\H,-5.6922134794,0.0847448368,2.891565815\H,-4.
5715968929,0.7517842086,4.0848019236\C,-2.613476116,1.9535216941,-2.50
09719639\C,-3.4886439369,3.0811010831,-3.0598636251\C,-1.9808400494,1.
1386873844,-3.632345866\H,-1.7952770486,2.4193138661,-1.9397342269\H,-
3.9222830357,3.6889176757,-2.2585433979\H,-2.8953940377,3.73948897,-3.
7037984887\H,-4.3147630199,2.6846455379,-3.6607032874\H,-1.3041973007,
0.3727395333,-3.2370665215\H,-2.7400474865,0.6411594442,-4.2462101579\
H,-1.4006445689,1.7939369667,-4.291388583\C,1.8733893173,3.4758273538,
-0.8473425898\C,2.6986057506,3.0734367827,-2.0711198425\C,1.9275519349
,4.9949891475,-0.640178213\H,0.8318128857,3.2027536733,-1.0556854225\H
,2.6657493463,1.9928840489,-2.2399359482\H,2.3030740159,3.5688326755,-
2.9647250348\H,3.7472737541,3.375534238,-1.9732310051\H,1.3048231601,5
.3166299106,0.201740624\H,2.9529815899,5.3239145892,-0.4368347166\H,1.
5787235411,5.5167134568,-1.5381900486\C,0.7729241901,1.0070139604,3.51
24954331\C,0.8174766394,1.7732383543,4.8387998827\C,0.9886443851,-0.49
23042304,3.7334714621\H,-0.2336723453,1.1302742299,3.0971019583\H,0.63
91209601,2.8435489788,4.6902090926\H,0.0521079513,1.392534057,5.524136
2325\H,1.7891129151,1.6632864269,5.3330355716\H,0.9026579395,-1.041377
5813,2.7893721152\H,1.9784328267,-0.6969246197,4.1574426624\H,0.239697
0134,-0.8849210207,4.4309011284\\Version=ES64L-G09RevD.01\State=1-A\HF
=-4405.9131186\RMSD=6.499e-09\RMSF=1.847e-06\Dipole=-0.4580596,-0.0869
159,0.7379112\Quadrupole=10.7835977,1.3295794,-12.1131771,1.9403064,5.
5695061,-1.2486998\PG=C01 [X(C41H49Au1Br1N2)]\\@

IN18

1\1\GINC-A748\FOpt\RPBE1PBE\GenECP\C41H49Au1Br1N2(1+)\SIOC001\26-Jan-2
016\0\\#p genecp pbe1pbe opt\\au_ph_br_int5_down_ipr\\1,1\C,-0.1254636
608,-0.0101577991,-0.0302130578\C,-0.3351441832,0.1619507888,1.5086307
368\H,0.9179568821,-0.02983465,-0.3599534133\H,-0.6794435257,0.7135058
45,-0.6350748424\H,-1.0209483377,0.9638706882,1.8118598009\H,0.5782641
309,0.2461541839,2.1096183612\C,-0.9634665267,-1.196853349,1.505497426
9\C,-1.5297595707,-2.1788115512,2.4312081502\C,-2.4127926401,-3.124000
6513,1.596549909\H,-2.0522846839,-1.6985484029,3.2663814229\H,-0.66067
0031,-2.7064591674,2.8853120258\C,-1.0103016088,-2.5979791481,-0.48762
47412\C,-1.5917086575,-3.6499673268,0.4053747532\H,-0.7166133245,-4.22
67623131,0.7739901111\H,-2.1706921249,-4.3631268641,-0.1884812317\C,-0
.7808822884,-1.3715305387,0.1447186637\Au,-4.2317115737,-2.2369114453,
1.0722592057\H,-2.649627558,-4.0016599963,2.2117258605\C,-0.6847067434
,-2.7992609145,-1.8877094648\C,-0.7828947868,-1.6628789437,-2.72741397
52\C,-0.321787605,-4.0232545091,-2.505551939\C,-0.5768002819,-1.731539
4329,-4.0925174919\H,-1.081978772,-0.7200638527,-2.2848089863\C,-0.083

9342991,-4.0828140941,-3.8743544142\C,-0.2209836257,-2.9493791367,-4.6
6829909\H,-0.6885861587,-0.8426092602,-4.7048888993\H,0.2151204286,-5.
0271919639,-4.3156755192\H,-0.0419540176,-3.0228440844,-5.7369410701\B
r,-0.0225005419,-5.6261170269,-1.5664906579\C,-8.2325993564,-1.1031546
882,0.138340603\C,-7.774775019,0.0822235504,0.6101191897\H,-9.20440946
61,-1.4000169285,-0.2247851072\H,-8.2630890743,1.0350343816,0.74464161
48\C,-6.0585156219,-1.3874940145,0.6816679117\N,-6.4454127385,-0.11185
1028,0.9352289298\N,-7.170750521,-1.9857264389,0.1899701608\C,-5.57975
25429,0.9036556851,1.4663700847\C,-4.8862085316,1.7273042153,0.5650630
172\C,-5.4662487025,1.0308017366,2.8599061472\C,-4.0311757048,2.691651
3438,1.1035136866\C,-4.5977446594,2.0120798624,3.3446355892\C,-3.88421
93369,2.8319249701,2.4789046565\H,-3.4827480255,3.3510039929,0.4357750
187\H,-4.4874180614,2.1408691238,4.4180008552\H,-3.2237841693,3.597016
7111,2.8785554546\C,-7.2380733542,-3.359927395,-0.2263938633\C,-7.5969
708807,-4.3305615145,0.7219057528\C,-6.9460583849,-3.6677914827,-1.564
7281124\C,-7.6449024921,-5.6580025028,0.2904607326\C,-7.0100309245,-5.
0108985341,-1.9410666923\C,-7.3533399797,-5.9969613188,-1.0245059887\H
,-7.9157054359,-6.4382394306,0.9965118387\H,-6.7889624062,-5.288482214
9,-2.9682098802\H,-7.3993670514,-7.0360368916,-1.3386402522\C,-5.02727
16736,1.5907254051,-0.9398624001\C,-3.7366448267,1.045169168,-1.559325
9333\C,-5.4441718723,2.9089826229,-1.5976000815\H,-5.8170286808,0.8595
537555,-1.1444530368\H,-3.473498326,0.0785983904,-1.1133551068\H,-3.85
94055751,0.9077984037,-2.6401602539\H,-2.9039216616,1.742735733,-1.402
3519869\H,-6.374195652,3.2944695948,-1.1669777613\H,-4.6772344518,3.68
26566161,-1.4799358266\H,-5.6021860983,2.7630166837,-2.6718755134\C,-6
.2437509687,0.1614907901,3.8302373675\C,-7.160828675,1.0055650285,4.72
12927856\C,-5.3045160215,-0.7109450301,4.668196195\H,-6.8823630323,-0.
5130551027,3.2490309846\H,-7.8536805713,1.6102139894,4.1262655329\H,-7
.7522689925,0.3573979517,5.3771563728\H,-6.5878395781,1.6872350545,5.3
5992683\H,-4.6896180642,-1.3515216493,4.0251659364\H,-4.6368433672,-0.
0997649747,5.2869166258\H,-5.8821915051,-1.3556094368,5.3401337961\C,-
6.5559427906,-2.6124211499,-2.5828773757\C,-7.4900274048,-2.6204377932
,-3.7963211269\C,-5.0932580704,-2.7803183841,-3.0071047073\H,-6.645713
2889,-1.6284122791,-2.1085702148\H,-8.5351460189,-2.4847402541,-3.4985
907852\H,-7.2247569523,-1.8108078445,-4.4854015246\H,-7.4224723155,-3.
5617527891,-4.3530344341\H,-4.4266331082,-2.7283485574,-2.1385013314\H
,-4.9328687174,-3.7455996428,-3.5014792225\H,-4.8065485214,-1.99103620
82,-3.7122572278\C,-7.9124969239,-3.9920868731,2.1669204722\C,-6.85094
19255,-4.5718306355,3.1073842878\C,-9.3175350583,-4.4567060977,2.56151
79606\H,-7.8857659336,-2.9019609829,2.2776231909\H,-5.8519838685,-4.20
35920062,2.846194536\H,-7.063606468,-4.2872367697,4.1443634811\H,-6.83
22776238,-5.6667095788,3.0588285913\H,-10.0819829656,-4.0315300992,1.9
022742182\H,-9.4087200634,-5.5477152751,2.5177551622\H,-9.5437400374,-
4.1487752697,3.5882667906\\Version=ES64L-G09RevD.01\State=1-A\HF=-4405

.929729\RMSD=4.359e-09\RMSF=1.055e-06\Dipole=1.7182416,1.2908227,-1.10
71167\Quadrupole=11.3802489,-9.8305376,-1.5497113,2.5893481,-13.002232
8,8.9235713\PG=C01 [X(C41H49Au1Br1N2)]\\@

TS17

1\1\GINC-A747\FTS\RPBE1PBE\GenECP\C41H49Au1Br1N2(1+)\SIOC001\30-Jan-20
16\0\\#p opt=(calcfc,ts,noeigen) genecp pbe1pbe\\au_ph_br_int5_down_ip
r\\1,1\C,1.3298586241,-3.3960483895,-0.8485560744\C,0.4352862144,-3.08
98715187,-1.9894088561\H,1.7530768168,-4.410617575,-0.8215236829\H,0.7
975225685,-3.2530706115,0.1105955766\H,-0.5007090654,-3.5243848997,-2.
3345985772\H,1.744914202,-2.937985969,-3.259827216\C,1.3499875156,-2.1
217833503,-2.5598229171\C,1.1873813549,-0.8140387636,-3.292825701\C,1.
4677096528,0.2917930824,-2.2508134608\H,0.1931872097,-0.7369840317,-3.
7442832947\H,1.9266245399,-0.7682984678,-4.1091222318\C,2.8812523747,-
1.2165505401,-0.7919222849\C,2.8571427341,0.0575802149,-1.6228003305\H
,3.6402439966,-0.0033884332,-2.3993144025\H,3.1352941469,0.9102278113,
-0.9976891362\C,2.1769673216,-2.2271792446,-1.3126950713\Au,-0.0928589
778,0.3688472656,-0.8580232169\H,1.5085475637,1.2443941686,-2.79643872
05\C,3.5294960318,-1.2552007044,0.5305023835\C,3.3944944293,-0.1456995
787,1.3827664645\C,4.2920936904,-2.3348109074,1.0115888022\C,3.9684146
065,-0.1094091136,2.6453093269\H,2.7972773773,0.6960698264,1.045119798
3\C,4.8822597997,-2.3031789772,2.2722820878\C,4.7186807705,-1.19297517
7,3.0925606075\H,3.8340171904,0.7654416653,3.2745134378\H,5.4790428073
,-3.1474493041,2.6001862183\H,5.1864502893,-1.1772439159,4.0724659596\
Br,4.6403556986,-3.8802681246,-0.0309303433\C,-3.3341968037,1.39149793
66,1.7183079685\C,-3.4278027379,0.0433525743,1.8120232638\H,-3.9242436
375,2.1805100829,2.1584228962\H,-4.117336914,-0.5895192744,2.348889416
8\C,-1.6789384735,0.5128948752,0.4539536513\N,-2.4077191427,-0.4755789
05,1.0350419917\N,-2.263125636,1.6565545218,0.8877572142\C,-2.18802005
79,-1.8817583405,0.8539473297\C,-1.4362541114,-2.5751598434,1.81793671
01\C,-2.7845218689,-2.5154672706,-0.2500147973\C,-1.2798210013,-3.9547
850597,1.6404844883\C,-2.5864986255,-3.8949845819,-0.3835116047\C,-1.8
467351187,-4.6091854245,0.5521319299\H,-0.7236224716,-4.526778633,2.37
81808169\H,-3.0547961313,-4.4218697566,-1.2117218767\H,-1.7287055589,-
5.6842868202,0.4449254197\C,-1.8415239766,2.9826268131,0.5209825452\C,
-2.3561687855,3.5472873049,-0.6572287469\C,-0.9546406347,3.6620864399,
1.3718292226\C,-1.9383167972,4.8397996667,-0.9812910169\C,-0.577823688
9,4.9546999053,1.0026230868\C,-1.0596457934,5.5375237115,-0.1627241732
\H,-2.3130360734,5.3082225945,-1.8871533851\H,0.1028262034,5.513752771
1,1.6386130517\H,-0.7524744222,6.5442793364,-0.4316250226\C,-0.8531727
396,-1.8960696266,3.043011525\C,0.6329281512,-2.2089415858,3.230150345
7\C,-1.6518166038,-2.2775143282,4.295826772\H,-0.9435297038,-0.8128273
17,2.9049130959\H,1.2206491654,-1.9216505583,2.3520954428\H,1.02658043
61,-1.6528126467,4.087595197\H,0.805465347,-3.272814125,3.4290288381\H

,-2.7106185155,-2.0141966453,4.1994037761\H,-1.5931740724,-3.354912500
3,4.4886010705\H,-1.2522422303,-1.7579877253,5.1734170556\C,-3.6657416
461,-1.7790673683,-1.2417052281\C,-5.1092468254,-2.2903055783,-1.17066
00243\C,-3.1184933519,-1.8657396543,-2.6684127261\H,-3.6768013269,-0.7
19320163,-0.9670901515\H,-5.5154566905,-2.2047175655,-0.1573228143\H,-
5.74983522,-1.7099544051,-1.8434254838\H,-5.1791837656,-3.3420487804,-
1.4703810423\H,-2.1124561322,-1.4338828761,-2.7261888905\H,-3.08306631
57,-2.9020775063,-3.027343731\H,-3.7623877507,-1.3071523672,-3.3563347
145\C,-0.4250054567,3.0560781756,2.6578042345\C,-1.012104884,3.7669612
61,3.8822484001\C,1.105432293,3.0677469245,2.7003811462\H,-0.741944369
8,2.0075506268,2.6985561943\H,-2.1068182426,3.7260182237,3.8865288637\
H,-0.6514981658,3.3017835794,4.8065714442\H,-0.7200566443,4.8229717597
,3.9032982213\H,1.5265420657,2.5736201613,1.8181714772\H,1.502958145,4
.0880435656,2.734649154\H,1.4619147455,2.5460447681,3.5960572142\C,-3.
3355751835,2.8224060295,-1.5612042384\C,-2.7385593698,2.5851229663,-2.
9517924763\C,-4.6681284851,3.5734009799,-1.6518617317\H,-3.5427004636,
1.8389685824,-1.1234944105\H,-1.8058729458,2.0128103759,-2.8871803403\
H,-3.4447712066,2.0280523601,-3.5785891768\H,-2.5198718795,3.530948325
3,-3.4603170661\H,-5.1129213691,3.7240924956,-0.6623257216\H,-4.542115
0173,4.5589934747,-2.1135948532\H,-5.3812860002,3.0104986268,-2.264190
1725\\Version=ES64L-G09RevD.01\State=1-A\HF=-4405.8583217\RMSD=9.848e-
09\RMSF=1.942e-06\Dipole=-1.2796749,-1.4710481,-0.0498568\Quadrupole=-
7.2964916,10.4275459,-3.1310542,-4.8404326,4.7629158,10.1185399\PG=C01
[X(C41H49Au1Br1N2)]\\@

IN20

1\1\GINC-A733\FOpt\RPBE1PBE\GenECP\C41H49Au1Br1N2(1+)\ROOT\26-Jan-2016
\0\\#p opt genecp pbe1pbe\\Title Card Required\\1,1\C,0.1986904152,-0.
11546036,0.27716962\C,0.1362420612,-0.225338021,1.8292041677\H,1.18135
70182,-0.1295272477,-0.2011731329\H,-0.3659036463,0.7529339096,-0.0826
479277\H,-0.0349694259,0.6921495093,2.3981409468\H,1.0129346145,-0.748
9005382,2.2212848551\C,-1.0766644912,-1.1922536124,1.66581918\C,-1.204
6272031,-3.6786815229,1.7706220235\H,-0.9401074609,-2.4589108336,3.510
4390833\C,-1.1643022916,-2.4725761241,2.4423730919\C,-0.5149452668,-2.
5642809783,-0.3803230925\C,-1.1573101731,-3.7819037953,0.2589600209\H,
-0.5832111243,-4.6790143763,0.0021225296\H,-2.1624722534,-3.9503530079
,-0.1557079685\C,-0.5976353125,-1.3941675217,0.2519867922\Au,-3.308079
1218,-3.2314483452,2.5063906292\H,-1.0148755343,-4.5949371809,2.331074
3745\C,0.2215815471,-2.7343144425,-1.6542539026\C,-0.2827133031,-2.176
5220384,-2.8355559051\C,1.4305485317,-3.4372721257,-1.7394976862\C,0.3
801596315,-2.3234842544,-4.0492313291\H,-1.2164017606,-1.6229232612,-2
.7853795618\C,2.1035595399,-3.5964253426,-2.9451962997\C,1.5716801283,
-3.0400171435,-4.1045637916\H,-0.0337488837,-1.879616517,-4.9497613553
\H,3.0412395501,-4.1412809776,-2.9700153734\H,2.0974200597,-3.16345118

89,-5.0467715002\H,-2.0083516508,-0.6118882871,1.7223505435\Br,2.22885
09669,-4.150566511,-0.1741369544\C,-7.4958534828,-3.7080217854,2.53937
51424\C,-7.2994209311,-3.454947496,3.8588747716\H,-8.3937056317,-3.915
1481954,1.977825283\H,-7.990747024,-3.3924470833,4.6849973294\C,-5.307
3575492,-2.9648845789,5.2861852004\C,-5.1740704888,-1.617616062,5.6593
923241\C,-4.8574706453,-4.0337526131,6.0778809441\C,-4.5418798589,-1.3
555648352,6.8765930466\C,-4.232628727,-3.7107314293,7.2843359876\C,-4.
0735630479,-2.3883012859,7.679512409\H,-4.4194264303,-0.3263194936,7.2
020673185\H,-3.869691616,-4.508959984,7.9257643897\H,-3.5875663689,-2.
1609804493,8.6241200757\C,-6.0059521834,-3.8860083531,0.5383558162\C,-
5.7283019616,-5.1949182094,0.1109073181\C,-6.0519824631,-2.7838198443,
-0.3305638513\C,-5.4676131911,-5.3768638437,-1.2490935814\C,-5.7888595
442,-3.0273944699,-1.6803995978\C,-5.4957940079,-4.3070284031,-2.13543
07415\H,-5.2452681855,-6.3735585037,-1.6198442144\H,-5.8175354187,-2.2
012013775,-2.3854104394\H,-5.2952471634,-4.4736078674,-3.1900628564\C,
-5.698278761,-6.3827333654,1.0549029839\C,-4.2808595329,-6.9503133127,
1.1812884165\C,-6.6906247284,-7.4683665398,0.6265571458\H,-6.002554515
6,-6.0400317809,2.0508281561\H,-3.580152467,-6.186658726,1.5400383657\
H,-4.2654642389,-7.7866626246,1.8890289123\H,-3.9120683039,-7.32106642
21,0.218189953\H,-7.7086166998,-7.0731167233,0.5451438246\H,-6.4221435
485,-7.9016121079,-0.3432237866\H,-6.6985833874,-8.2825626882,1.359244
865\C,-6.3787010478,-1.3776911528,0.1364569205\C,-7.6905633696,-0.8818
307102,-0.4813365993\C,-5.2295234342,-0.4089861768,-0.1576449323\H,-6.
5157476634,-1.3979977117,1.2239089058\H,-8.5239876434,-1.553885294,-0.
2501448908\H,-7.9396990381,0.11356383,-0.0977351854\H,-7.616088441,-0.
8084469739,-1.5720288063\H,-4.296333667,-0.7482928452,0.3065817258\H,-
5.0517458332,-0.3111735733,-1.2343983441\H,-5.4646589989,0.5882547277,
0.2306880112\C,-5.6830380247,-0.4706548106,4.8059395772\C,-4.527899338
1,0.4036885468,4.3078947528\C,-6.7236511135,0.3668613815,5.556417185\H
,-6.1786588879,-0.8910697009,3.9229504029\H,-3.8040820186,-0.186162006
1,3.7330432742\H,-4.906149914,1.2052669645,3.663478968\H,-3.9928438847
,0.8713061304,5.1420521337\H,-7.5626522036,-0.2477283909,5.8994817388\
H,-6.2885731006,0.8563516905,6.4346451699\H,-7.1207777874,1.151852555,
4.903742798\C,-5.0236033978,-5.4873408346,5.675341265\C,-5.8435239745,
-6.2647849109,6.7101915617\C,-3.6646630087,-6.1529732451,5.4359366722\
H,-5.575812651,-5.5232285611,4.7289284029\H,-6.8247484427,-5.806335255
1,6.8728658369\H,-6.0004763842,-7.2949771014,6.3725387568\H,-5.3318339
554,-6.3092606718,7.6778477679\H,-3.0964138715,-5.6250437149,4.6609287
306\H,-3.0587848109,-6.1633858779,6.3489254208\H,-3.799981681,-7.19150
12064,5.1138269281\N,-6.255536402,-3.6635814553,1.9386648941\N,-5.9442
331823,-3.2651078846,4.0295710207\C,-5.2964188768,-3.3912448063,2.8510
459341\\Version=ES64L-G09RevD.01\State=1-A\HF=-4405.9730849\RMSD=3.920
e-09\RMSF=2.618e-06\Dipole=-1.903119,0.3214317,0.5479719\Quadrupole=-0
.5935226,-14.1439583,14.737481,4.2327479,-13.8512935,-0.1363563\PG=C01

[X(C41H49Au1Br1N2)]\\@

TS16

1\1\GINC-SHI_03\FTS\RPBE1PBE\GenECP\C41H49Au1Br1N2(1+)\YIN\29-Jan-2016
\0\\#p opt=(calcfc,ts,noeigen) genecp pbe1pbe\\Title Card Required\\1,
1\C,2.4000371211,-1.5146347778,-0.4962773106\C,2.2882258739,-1.0474551
607,0.9676147996\H,1.4518001496,-1.8904109942,-0.875095764\H,3.2739015
444,-2.0900520836,-0.7917010672\H,3.2082637878,-1.0136512164,1.5470285
253\H,1.4255292264,-1.4350026203,1.4983630514\C,1.6407579327,0.5387406
962,0.4635178042\C,2.335639028,1.7506934246,1.0843584819\C,3.655850507
3,1.9034423824,0.3487305267\H,2.481481194,1.5835389405,2.1585413359\H,
1.7113476986,2.6461025825,0.9903891303\C,3.8530292523,0.6706762484,-0.
5356916773\C,3.5718537706,2.0559667464,-1.1237342442\H,2.6054350077,2.
2367990287,-1.5902413622\H,4.4194168484,2.5033010646,-1.6343824397\C,2
.5749342446,-0.0665004407,-0.4243465656\Au,-0.416752707,0.4162283991,0
.3308456346\H,4.5185000345,2.3135529894,0.8633829369\C,5.1367072312,-0
.0239524909,-0.8428043301\C,6.057450767,-0.4579295387,0.1198029887\C,5
.4430543969,-0.2849518214,-2.1857193316\C,7.2331671868,-1.1118483452,-
0.239212777\C,6.6061416204,-0.9461410508,-2.5587375489\H,4.7451312807,
0.0549156702,-2.9472051255\C,7.5058546495,-1.358152885,-1.5798011692\H
,7.9279905122,-1.4240761842,0.5329982295\H,6.813218355,-1.1304344631,-
3.6084454002\H,8.4245400429,-1.8681673615,-1.8538029583\Br,5.772822825
5,-0.1744289276,1.9739711122\C,-4.5168532619,-0.4937874135,-0.19102311
86\C,-4.6339174537,0.8226311958,0.11417233\H,-5.2581834655,-1.24191787
54,-0.425574371\H,-5.499013384,1.4615866187,0.2014183516\C,-2.44120113
57,0.3114137167,0.1575821332\N,-3.3538343408,1.2963309531,0.3232881899
\N,-3.167846294,-0.7868528827,-0.1590311482\C,-3.0334372954,2.65625899
57,0.6664312808\C,-2.9681342153,3.0057634715,2.0245979946\C,-2.8061102
465,3.5708623091,-0.3743798731\C,-2.6467466829,4.330871418,2.327158740
7\C,-2.4894351578,4.8820503822,-0.0130275642\C,-2.4085901132,5.2592981
17,1.3216645093\H,-2.5854701541,4.6395221852,3.3671616378\H,-2.3062497
718,5.6194139793,-0.789760214\H,-2.1639717883,6.2858821043,1.579924640
6\C,-2.5993230769,-2.0797052128,-0.4275047793\C,-2.2331430788,-2.38767
6264,-1.747720749\C,-2.4322457045,-2.9724842461,0.6437018313\C,-1.6605
279167,-3.6413714817,-1.9756662389\C,-1.8545304168,-4.2116469392,0.357
6881897\C,-1.4696968184,-4.5432341092,-0.9358224771\H,-1.3686746291,-3
.9168070358,-2.9855347867\H,-1.7090905848,-4.9299373079,1.1599487483\H
,-1.0284363768,-5.5156921196,-1.1364578819\C,-2.8796046117,3.184755150
8,-1.84009674\C,-1.4988049036,3.2728522405,-2.4981944787\C,-3.90852110
65,4.0307423267,-2.596087489\H,-3.205536354,2.1401648938,-1.9043183557
\H,-0.7768925687,2.6282359487,-1.9828707913\H,-1.5545103995,2.95871054
91,-3.5467726154\H,-1.1135992127,4.2988349033,-2.4766618423\H,-4.90109
05811,3.958997115,-2.1387988883\H,-3.6259985155,5.0891265339,-2.614215
3286\H,-3.9871740637,3.6932957229,-3.6354106113\C,-3.2202118892,2.0128

272344,3.1436989897\C,-4.3809364334,2.4568290848,4.0394329331\C,-1.948
1467626,1.7759614843,3.9641870971\H,-3.5025101991,1.0532340831,2.69520
45789\H,-5.2987643335,2.6096058021,3.4617196666\H,-4.5816362422,1.6981
190688,4.8037820777\H,-4.1537323436,3.394859282,4.5579912607\H,-1.1302
334588,1.4168010944,3.3286752263\H,-1.6145390982,2.6969282968,4.455892
8638\H,-2.1311485743,1.0289500292,4.7450426884\C,-2.4342171962,-1.4261
028209,-2.9045433\C,-3.3478220924,-2.0266638688,-3.9776436839\C,-1.091
322116,-0.9920618048,-3.5005589737\H,-2.9267632135,-0.5250557214,-2.52
18656645\H,-4.3181016899,-2.3175571081,-3.561342741\H,-3.5247746766,-1
.2983146427,-4.7765464172\H,-2.9013427374,-2.9168582407,-4.4344651654\
H,-0.4607929083,-0.5137916875,-2.7415843415\H,-0.5433639156,-1.8477083
052,-3.9121155044\H,-1.2490991678,-0.275587536,-4.3146510363\C,-2.8374
376742,-2.6334845171,2.0664454762\C,-1.6070040353,-2.502642677,2.97003
44158\C,-3.8307913472,-3.6534744946,2.6309983729\H,-3.3397171381,-1.65
94321542,2.0568786567\H,-0.9309148578,-1.7222416147,2.5999466867\H,-1.
9069521398,-2.2359835919,3.9896989219\H,-1.0493602318,-3.4450383173,3.
0214249296\H,-4.7190572972,-3.7420108227,1.9967145126\H,-3.3826898569,
-4.6493154065,2.7189315025\H,-4.1558910751,-3.3499248819,3.6320002501\
\Version=EM64L-G09RevA.01\State=1-A\HF=-4405.9296991\RMSD=5.079e-09\RM
SF=1.892e-06\Dipole=0.1684079,-0.4899627,-0.8219908\Quadrupole=26.5473
199,-8.8650259,-17.682294,-8.197427,-10.6493851,4.0213111\PG=C01 [X(C4
1H49Au1Br1N2)]\\@

IN19

1\1\GINC-A765\FOpt\RPBE1PBE\GenECP\C41H49Au1Br1N2(1+)\ROOT\26-Jan-2016
\0\\#p opt genecp pbe1pbe\\Title Card Required\\1,1\C,-0.1341381823,-0
.3919894646,-0.0252426894\C,-0.0839947939,-0.8131117807,1.4057755332\C
,1.3435901618,-0.6595561032,1.8824172078\H,-0.4143099084,-1.8660578588
,1.4259091977\H,-0.8338654446,-0.2740899982,2.0026971872\C,2.193435377
9,-0.3023253292,0.6783422677\C,1.9670159769,0.7000434809,1.7810634048\
H,1.3336314239,1.5612457988,1.5726021046\H,2.7982212672,0.9037232915,2
.4503458664\Au,-1.842636789,-0.2497704094,-1.0715749139\H,1.7252974058
,-1.3456559237,2.6317136286\C,3.5437647751,-0.8716758052,0.418070997\C
,3.7504852632,-2.2297048781,0.1368725251\C,4.6697113792,-0.0402334001,
0.4226899884\C,5.0178713777,-2.7401775229,-0.121902804\C,5.9440260835,
-0.5333929441,0.1648570037\H,4.5307456403,1.0149665571,0.6445668385\C,
6.1171404248,-1.8869153105,-0.1075374092\H,5.1399076853,-3.7985122552,
-0.3263391214\H,6.7984186856,0.1364405217,0.1801477328\H,7.1070125545,
-2.2872566193,-0.3054281653\C,1.1972486532,-0.1516596912,-0.4592112514
\C,1.5574630749,-0.5016204837,-1.9380709066\C,1.4406331446,0.900565657
6,-1.5940750968\H,2.5435041145,-0.9514840613,-2.0147853517\H,0.7678936
568,-0.9789114517,-2.5079946299\H,0.5704112372,1.4609962613,-1.9202358
418\H,2.3447092643,1.4755111923,-1.4105463964\Br,2.2915064788,-3.44058
7702,0.1084541994\C,-5.7640971934,-0.1504729248,-2.7097523293\C,-5.061

4901432,0.2771700742,-3.7901783271\H,-6.8208137204,-0.3128987955,-2.56
37197775\H,-5.3788677643,0.5641645583,-4.7808051963\C,-3.591012208,-0.
1031955278,-2.128863747\N,-4.8467316653,-0.3770151648,-1.7057212956\N,
-3.7353485271,0.2983943733,-3.4137552027\C,-2.6429023754,0.6900964454,
-4.2636435093\C,-2.0165293777,-0.2966416061,-5.0421312913\C,-2.2532671
716,2.0393230515,-4.2703489174\C,-0.9414045106,0.1072039329,-5.8373050
08\C,-1.1717643478,2.3851798428,-5.0841629871\C,-0.5200311364,1.431114
7494,-5.8562824776\H,-0.4321137384,-0.6261713303,-6.4565419663\H,-0.84
00593989,3.4194560324,-5.1195432991\H,0.3153932674,1.7238357531,-6.486
2516927\C,-5.1796351417,-0.8433291329,-0.3851619352\C,-5.4662734039,0.
1087720227,0.6063328267\C,-5.2105167283,-2.2279378593,-0.1550580602\C,
-5.7801955251,-0.3726375416,1.8791517081\C,-5.5293910753,-2.6513354746
,1.1370298441\C,-5.8098056713,-1.7361569222,2.1439466353\H,-6.00851962
85,0.3326281137,2.6735470593\H,-5.5626173419,-3.7151789122,1.355322144
3\H,-6.0598251315,-2.0883401584,3.1408242216\C,-5.4429593633,1.6035477
714,0.3461612607\C,-4.3350999645,2.2864749848,1.1543664178\C,-6.806623
6669,2.2426488554,0.6269907333\H,-5.2187504153,1.7656132931,-0.7145400
174\H,-3.3532630648,1.8611911947,0.9154688692\H,-4.3069139973,3.359426
5724,0.932976893\H,-4.499330489,2.1730354858,2.2320259315\H,-7.6001779
16,1.7718150942,0.0370114869\H,-7.081677371,2.1557758678,1.6839030263\
H,-6.7844215204,3.3092933616,0.3782739435\C,-4.9122408841,-3.251025808
6,-1.2356237226\C,-6.0976031771,-4.1962357674,-1.4554269986\C,-3.63379
55839,-4.0325576846,-0.9164971872\H,-4.7417781638,-2.7175119812,-2.177
9788974\H,-7.0107566328,-3.644798386,-1.7036487271\H,-5.8832253703,-4.
8882778209,-2.2771157938\H,-6.3038710829,-4.7976918829,-0.5632850335\H
,-2.7750547505,-3.3595128217,-0.8093576629\H,-3.7361837586,-4.60052728
1,0.0151594525\H,-3.412294868,-4.7454949128,-1.7187304958\C,-2.9555209
959,3.1036783124,-3.4474100688\C,-3.5309356288,4.2090750399,-4.3386236
586\C,-2.0246564865,3.6861504896,-2.3792453709\H,-3.7972369583,2.63468
60754,-2.9248897342\H,-4.2136487863,3.8025299678,-5.0922142516\H,-4.08
49084837,4.9342810035,-3.7327553486\H,-2.740011438,4.7545043427,-4.865
1476469\H,-1.6609449203,2.9044480231,-1.7015008681\H,-1.1554995376,4.1
775302989,-2.8317214785\H,-2.5539446878,4.4349655671,-1.7793307352\C,-
2.463279406,-1.7470501334,-5.049969429\C,-1.3764756957,-2.6670446688,-
4.4856274526\C,-2.8892329803,-2.1914803474,-6.452943638\H,-3.339524593
,-1.8416899407,-4.3982566008\H,-1.1130471641,-2.3847088808,-3.45943415
8\H,-1.725400233,-3.7056335564,-4.4714204929\H,-0.4656807439,-2.631442
4375,-5.0945696352\H,-3.6806829084,-1.5496879227,-6.8543485593\H,-2.04
98779533,-2.164706141,-7.1567006905\H,-3.2653139294,-3.2199912374,-6.4
266274735\\Version=ES64L-G09RevD.01\State=1-A\HF=-4405.9406827\RMSD=4.
149e-09\RMSF=2.981e-06\Dipole=-0.01195,0.907575,-0.4649546\Quadrupole=
28.7886802,-18.8868226,-9.9018576,1.2462033,4.9738861,-2.9600792\PG=C0
1 [X(C41H49Au1Br1N2)]\\@

TS18

1\1\GINC-A727\FTS\RPBE1PBE\GenECP\C41H49Au1Br1N2(1+)\ROOT\29-Jan-2016\
0\\#p opt=(calcfc,ts,noeigen) genecp pbe1pbe\\Title Card Required\\1,1
\C,1.684022359,0.1500950369,-0.648231353\H,2.1580596725,1.0566634371,0
.0311048773\C,2.360668737,1.1278580186,-1.404895983\C,3.6835994484,0.7
272179509,-1.8507993121\H,1.9375833706,2.1142374597,-1.5890400091\C,3.
9122896064,-0.6744070642,-1.2455468217\C,3.720037578,-0.5310850345,-2.
7105796604\H,2.7923664235,-0.8813732833,-3.1560025278\H,4.5933542493,-
0.5940657733,-3.3533107665\Au,-0.3143415455,0.169984646,-0.1534861378\
H,4.4806660664,1.4539183292,-1.9666895786\C,5.2348912666,-1.0510751896
,-0.6787354469\C,5.759810468,-0.441940232,0.4689119542\C,5.9898271391,
-2.0696233587,-1.2705407301\C,6.9848518438,-0.8222476474,1.0041002422\
C,7.2159778865,-2.464088513,-0.7468366435\H,5.5965625626,-2.5575048081
,-2.1587734919\C,7.7134449184,-1.8378487881,0.3917388194\H,7.364836837
5,-0.3233772994,1.889252682\H,7.7803722954,-3.2575984298,-1.2269278504
\H,8.6714642847,-2.1336218908,0.8087472126\C,2.6252385147,-1.004844279
2,-0.4951179571\C,2.6062864577,-1.8430922936,0.759359652\C,2.081167082
5,-2.4218789252,-0.5052848465\H,3.5682055114,-2.1920415531,1.123862906
2\H,1.9001279528,-1.5561481306,1.5330508179\H,1.0076540316,-2.54864786
69,-0.6173101664\H,2.6895263558,-3.1589685222,-1.0236663926\Br,4.81779
08467,0.9553875168,1.3388192691\C,-4.4301174604,0.788562403,0.65245147
53\C,-4.3848217054,-0.5623930906,0.7663049488\H,-5.2457200959,1.487457
0849,0.7553240009\H,-5.1529082425,-1.2870337382,0.9881790753\C,-2.3037
510982,0.1481848237,0.2814270127\N,-3.0752937233,-0.9340357963,0.53736
23382\N,-3.1477122923,1.2035760888,0.3545201837\C,-2.7577682746,2.5745
584929,0.1583130873\C,-2.3253501124,3.3105727811,1.2730664816\C,-2.835
6610494,3.113670153,-1.1359541372\C,-1.9418228443,4.6352224392,1.05227
56212\C,-2.4395241916,4.4431341167,-1.2975682241\C,-1.9952019388,5.196
1026395,-0.2176462994\H,-1.6011689004,5.2372779584,1.8901716862\H,-2.4
848073309,4.8958700161,-2.2841378472\H,-1.6964038068,6.2302138706,-0.3
65351958\C,-2.5879284143,-2.2866071795,0.5701991296\C,-2.5625092284,-3
.0174759371,-0.628541627\C,-2.1626010574,-2.8122740553,1.8010377413\C,
-2.0695156027,-4.3230979684,-0.5683190428\C,-1.6773049512,-4.121918605
4,1.8008568047\C,-1.6284654309,-4.8694147348,0.6306778776\H,-2.0371711
447,-4.9222686541,-1.4743166206\H,-1.3359109719,-4.5635589471,2.732977
0364\H,-1.2517156514,-5.8881746659,0.654976133\C,-3.0516581034,-2.4525
567561,-1.9492542396\C,-4.2548712117,-3.2414051351,-2.4761479323\C,-1.
925703158,-2.403431677,-2.9865377231\H,-3.3834302871,-1.4217764106,-1.
7804684558\H,-5.0731680404,-3.2585165856,-1.7484026589\H,-4.6308223382
,-2.7896078808,-3.4006962622\H,-3.9865505409,-4.2800539791,-2.69945983
9\H,-1.0799505342,-1.8073261046,-2.6239181171\H,-1.5564395341,-3.40750
84155,-3.2248314778\H,-2.2866530228,-1.953620888,-3.9183394651\C,-2.20
5727042,-2.018644904,3.0939423044\C,-0.7923431122,-1.7173691636,3.6023
541457\C,-3.0385054709,-2.7298520223,4.1648133233\H,-2.690473497,-1.05

65972671,2.8912237899\H,-0.2188538881,-1.1519590429,2.8585287176\H,-0.
8357992554,-1.1237741232,4.5225469668\H,-0.2441390171,-2.6400055804,3.
8256163243\H,-4.0536968034,-2.9388844446,3.8111186222\H,-2.5865665617,
-3.6824781991,4.4626371434\H,-3.1120899692,-2.1062695619,5.0624288782\
C,-3.3116038435,2.3113671969,-2.3325825101\C,-2.1608146063,2.059333072
3,-3.3122654322\C,-4.4962322748,2.9840855486,-3.0327051854\H,-3.656694
6067,1.3342433289,-1.9751841517\H,-1.3342284588,1.5333162398,-2.820000
4115\H,-2.5020531952,1.4487806155,-4.156023114\H,-1.7720325406,3.00108
10197,-3.7168022629\H,-5.3251619162,3.1610619248,-2.3392575843\H,-4.21
39385171,3.9489455094,-3.4682475719\H,-4.8633313436,2.3507787327,-3.84
76795445\C,-2.2598298558,2.7257984735,2.6717172265\C,-3.1261327032,3.5
201214938,3.6541864374\C,-0.8113143781,2.632840426,3.1622519661\H,-2.6
584781973,1.7053992046,2.6372793564\H,-4.1666950982,3.5770556249,3.317
3263478\H,-3.1129888373,3.0442309409,4.6408271723\H,-2.7591678383,4.54
49373886,3.7780473167\H,-0.2045094287,2.0206344835,2.4847987477\H,-0.3
490111933,3.6244249568,3.2294239847\H,-0.7738884803,2.1794769528,4.159
2815329\\Version=ES64L-G09RevD.01\State=1-A\HF=-4405.9145654\RMSD=3.00
5e-09\RMSF=1.986e-06\Dipole=0.3350855,-0.283764,-0.7788771\Quadrupole=
27.6675606,-7.4316712,-20.2358893,-11.4848905,-8.3633738,0.0874871\PG=
C01 [X(C41H49Au1Br1N2)]\\@

IN21

1\1\GINC-SHI_02\FOpt\RPBE1PBE\GenECP\C41H49Au1Br1N2(1+)\YIN\27-Jan-201
6\0\\#p opt genecp pbe1pbe\\Title Card Required\\1,1\C,-0.1329956832,0
.0556189675,0.655447173\C,0.2827461333,0.4367219495,1.9169027397\C,1.7
672869377,0.5323307452,1.9806715569\H,-0.3301059856,0.3621658938,2.812
674319\C,2.2834030619,0.0573461722,0.6186821676\C,2.481694102,1.478425
1012,1.0449604707\H,1.9092569716,2.2666122368,0.5574992599\H,3.4847333
439,1.769867883,1.3446471081\Au,-0.724612038,2.2514155944,0.9378944306
\H,2.2603903511,0.2506242657,2.9055950727\C,3.4624260795,-0.8428527851
,0.4787706433\C,3.4762645558,-2.1568924857,0.9666886996\C,4.6076782673
,-0.3932389529,-0.1882361684\C,4.582599353,-2.9843468939,0.8052872434\
C,5.7201961526,-1.2088248703,-0.3618812309\H,4.613942319,0.6232610937,
-0.5741586282\C,5.7065719002,-2.50732881,0.1380794731\H,4.5602433482,-
3.9934130274,1.2028301258\H,6.5940786677,-0.831694611,-0.8845127491\H,
6.5694554474,-3.154909616,0.0138307502\C,1.0333820957,-0.2072816713,-0
.2221303583\C,0.9474398914,-1.3511141006,-1.2099046076\C,1.0020180155,
0.0470014984,-1.7156022486\H,1.8337447423,-1.9703001624,-1.3108615216\
H,0.0084348034,-1.8946407077,-1.2654335786\H,0.1024990501,0.4868260267
,-2.1383568866\H,1.9306028148,0.3936683657,-2.1620445536\H,-1.13079124
87,-0.3081296778,0.4140934529\Br,1.976382838,-2.8628652653,1.881586864
3\C,-1.7826903097,6.3061338752,0.4163868656\C,-3.0231084039,5.75563507
83,0.461814303\H,-1.4588303464,7.32927615,0.3037145349\H,-4.0044315826
,6.1994362683,0.3952637174\C,-1.5327262833,4.0900877797,0.6755965397\N

,-2.8482205195,4.3977200562,0.6224069638\N,-0.8835779303,5.2689782105,
0.5467710595\C,-3.9159683987,3.436803447,0.7205222372\C,-4.3851311345,
2.8396045471,-0.4606685794\C,-4.4383141228,3.1483205771,1.991683247\C,
-5.4112361257,1.9008815155,-0.3334909734\C,-5.4624961236,2.2011455063,
2.057303034\C,-5.9425073048,1.5816950217,0.9100174633\H,-5.8032239195,
1.4164583977,-1.2235892066\H,-5.893285382,1.948931966,3.0222178859\H,-
6.742295469,0.8503152305,0.9847679085\C,0.5478778732,5.4203981071,0.54
79158685\C,1.2179698091,5.4173094459,-0.6865543359\C,1.2027506659,5.57
2143745,1.7809496305\C,2.60745266,5.5557296071,-0.6557775053\C,2.59271
45813,5.70686886,1.7496646745\C,3.2885273711,5.6968009547,0.5472348944
\H,3.1633201785,5.5584710443,-1.5893665068\H,3.1365279294,5.8279581607
,2.6824201627\H,4.3691719144,5.8092188379,0.5468532888\C,0.4989222765,
5.2688206232,-2.0146299573\C,0.8939666013,3.9645541718,-2.7141523655\C
,0.7421059467,6.4781342181,-2.9229896667\H,-0.5785653572,5.2187568259,
-1.8194835225\H,0.6828365805,3.0951567776,-2.0811691368\H,0.3363614735
,3.8508410069,-3.6506615472\H,1.9619315366,3.9507424989,-2.9604228282\
H,0.444723717,7.4132763938,-2.4367481091\H,1.7983966849,6.5677216945,-
3.1993009507\H,0.1664098368,6.3777367634,-3.8494568083\C,0.4684654319,
5.6050345123,3.1082713511\C,0.6428073702,6.9591697319,3.8040256823\C,0
.9124435386,4.4567285713,4.0199422036\H,-0.602229287,5.4734540686,2.91
28841028\H,0.3064068544,7.784868824,3.1679931293\H,0.0623712137,6.9864
36628,4.7326538567\H,1.6910823807,7.1441636597,4.0635214629\H,0.766397
7764,3.4835543698,3.5367791807\H,1.9723697972,4.5431224175,4.284233185
9\H,0.3368238038,4.467289787,4.9522480682\C,-3.8308462882,3.1757090169
,-1.8327548307\C,-4.9192089587,3.738904451,-2.7524045006\C,-3.14858324
28,1.95961408,-2.4673816187\H,-3.0672850931,3.9536826104,-1.7163076557
\H,-5.3961857639,4.6227133287,-2.3157034961\H,-4.4889594435,4.02595357
33,-3.7180108246\H,-5.7035210508,2.9991262189,-2.9466709741\H,-2.34018
86134,1.5830915965,-1.8290225792\H,-3.8594659298,1.1413735452,-2.62867
17461\H,-2.7214297157,2.2266356547,-3.4405481624\C,-3.9346644644,3.811
3315685,3.2604201082\C,-3.2396776477,2.7955048632,4.1728207274\C,-5.06
10309709,4.5384850743,4.001653703\H,-3.1886456282,4.5653365426,2.98285
25129\H,-2.4005760319,2.3123944811,3.6581203095\H,-2.8516783322,3.2901
538865,5.0703534027\H,-3.9329971192,2.0112250387,4.4970562056\H,-5.554
1413405,5.2760839558,3.3597226701\H,-5.8268968401,3.8402562091,4.35654
56111\H,-4.6613084936,5.0614120365,4.8771613833\\Version=EM64L-G09RevA
.01\State=1-A\HF=-4405.9605428\RMSD=9.070e-09\RMSF=1.735e-06\Dipole=-0
.4545588,1.5514469,-0.4801754\Quadrupole=17.9237655,0.1013768,-18.0251
422,-12.3428027,-4.546082,1.9521265\PG=C01 [X(C41H49Au1Br1N2)]\\@

15. Reference

[1] Vasu, D.; Das, A.; Liu, R.-S. *Chem. Commun.* **2010**, *46*, 4115-4117.

[2] (a) Buckman, B.; Nicholas, J. B.; Serebryany, V.; Seiwert, S. D. *PCT Int. Appl.* **2011**, WO2011075607 A1 20110623. (b) Stark, T.; Suhartono, M.; Goebel, M. W.; Lautens, M. *Synlett* **2013**, *24*, 2730-2734. (c) Castanet, A.-S.; Colobert, F.; Broutin, P.-E. *Tetrahedron Lett.* **2002**, *43*, 5047-5048.

[3] Kozhushkov, S. I.; Wagner-Gillen, K.; Khlebnikov, A. F.; de Meijere, A. *Synthesis* **2010**, 3967-3973.

[4] Piel, I.; Steinmetz, M.; Hirano, K.; Frohlich, R.; Grimme, S.; Glorius, F. *Angew. Chem., Int. Ed.* **2011**, *50*, 4983-4987.

[5] Ma, S.; He, Q. *Tetrahedron* **2006**, *62*, 2769-2778.

[6] Zheng, H.; Felix, R. J.; Gagné, M. R. *Org. Lett.* **2014**, *16*, 2272-2275.

[7] Deeter, G. A.; Venkataraman, D.; Kampf, J. W.; Moore, J. S. *Macromolecules* **1994**, *27*, 2647.
